# Supplementary material for: Synthesis of meta-carbonyl phenols and anilines
Source: Nat Commun. 2024 Mar 18;15:2415. doi: 10.1038/s41467-024-46576-2 (PMC10948751; doi:10.1038/s41467-024-46576-2)
Supplement: Supplementary file 1 — Supplementary Information [file 41467_2024_46576_MOESM1_ESM.pdf]

Supplementary Information

Synthesis of *meta*-carbonyl phenols and anilines

Bao-Yin Zhao[1], Qiong Jia[1], and Yong-Qiang Wang[1]*

[1]Key Laboratory of Synthetic and Natural Functional Molecule Chemistry of Ministry of Education, College of Chemistry & Materials Science, School of Foreign Languages, Northwest University; Xi'an 710069, China.

*Corresponding author. Email: wangyq@nwu.edu.cn

Table of Contents

1. General Information S3
2. Reaction Optimization S4
   2.1 Condition optimization for synthesis of *meta*-carbonyl phenols S4
   2.2 Condition optimization for synthesis of *meta*-carbonyl phenols S8
3. Procedure for the Synthesis of Starting Materials and Products S10
   3.1 Procedure for the synthesis of starting materials S10
   3.2 General procedure for the synthesis of *meta*-carbonyl phenols S13
   3.3 Gram-scale for the synthesis of *meta*-carbonyl phenols S14
   3.4 General procedure for the synthesis of *meta*-carbonyl anilines S14
4. Characterization Data of Starting Materials and Products S15
   4.1 Characterization data of starting materials S15
   4.2 Characterization data of products S22
5. Preliminary Mechanistic Studies S51
   5.1 ^{18}O-labeled water experiment S51
   5.2 Deuterium-labeling experiments S51
      5.2.1 H/D exchange experiments–investigations of γ-C−H activation S51
      5.2.2 H/D exchange experiments – investigations of α-C−H activation S52
   5.3 KIE experiments S53
   5.4 Intermediate traping experiments and kinetic profiles S56
      5.4.1 Intermediate traping experiments S56
      5.4.2 Kinetic profiles S60
   5.5 Kinetic studies S61
      5.5.1 Determination of kinetic rate constants of the first step: k_1 .... S61
      5.5.2 Determination of kinetic rate constants of the second step: k_2 .... S62
      5.5.3 Determination of kinetic rate constants of the third step: k_3 .... S63
      5.5.4 Determination of kinetic rate constants of the fourth step: k_4 .... S64
   5.6 Proposed mechanism S66
   5.7 Product diversification S67
6. X-Ray Crystallographic Data S70
7. NMR Spectra S75
8. References S209

1. General Information

Unless noted otherwise, commercially available chemicals were used without further purification. Flash chromatography was performed with silica gel (200-300 mesh). Oil bath served as the heat source. NMR spectra were acquired on Bruker 400 MHz (^{1}H at 400 MHz, ^{13}C at 101 MHz) or Jeol 400 MHz (^{1}H at 400 MHz, ^{13}C at 101 MHz, ^{19}F at 376 MHz). Chemical shifts are reported in parts per million (ppm, δ), downfield from tetramethylsilane (TMS, δ = 0.00 ppm). The residual solvent signals were used as references for ^{1}H and ^{13}C NMR spectra ($CDCl_3$: δ_H = 7.26 ppm, δ_C = 77.16 ppm; DMSO-d_6: δ_H = 2.50 ppm, δ_C = 39.52 ppm; Acetone-d_6: δ_H = 2.05 ppm, δ_C = 29.8, 206.3 ppm). Coupling constants, *J* were reported in Hertz unit (Hz). Data for ^{1}H NMR spectra were reported as follows: chemical shift (ppm, referenced to protium, s = singlet, d = doublet, t = triplet, q = quartet, dd = doublet of doublets, td = triplet of doublets, m = multiplet, br = broad, coupling constant (Hz), and integration). Infrared (IR) data were acquired on a Bruker Invenio-R FT-IR spectrometer. Absorbance frequencies are reported in reciprocal centimeters (cm^{-1}). Mass spectra were acquired on a BrukerDaltonics S2 MicroTof-Q II mass spectrometer. X-ray crystal structure analyses were measured on Bruker Smart APEXIICCD instrument using Mo-Kα radiation (λ = 0.71073 Å). The structures were solved and refined using the SHELXTL software package.

2. Reaction Optimization

2.1 Condition optimization for synthesis of *meta*-carbonyl phenols

Supplementary Table 1. Screening of Cu salts[a,b]

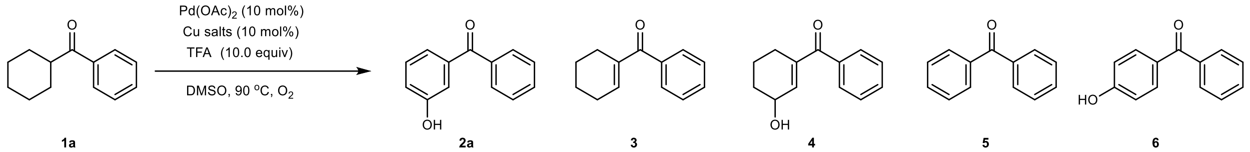

Entry	Catalyst	Cu salts	**2a** (%)	**3** (%)	**4** (%)	**5** (%)	**6** (%)
1	$Pd(OAc)_2$	$Cu(OAc)_2$	6	trace	trace	11	7
2	$Pd(OAc)_2$	CuI	16	trace	trace	7	trace
3	$Pd(OAc)_2$	Cu(OAc)	6	trace	trace	14	6
4	$Pd(OAc)_2$	$CuBr_2$	5	trace	trace	6	trace
5	$Pd(OAc)_2$	CuBr	trace	trace	trace	8	trace
6	$Pd(OAc)_2$	$CuCl_2$	-	-	-	-	-
7	$Pd(OAc)_2$	CuCl	trace	trace	trace	7	trace
8	$Pd(OAc)_2$	$CuSO_4$	6	trace	trace	13	7
9	$Pd(OAc)_2$	$Cu(OTf)_2$	7	trace	trace	16	9
10	$Pd(OAc)_2$	Cu_2O	5	trace	trace	10	7
11	$Pd(OAc)_2$	-	trace	trace	trace	12	5
12	-	CuI	41	trace	trace	<5	n.d.

[a]Reaction conditions: **1a** (0.25 mmol), $Pd(OAc)_2$ (10 mol%), Cu salts (10 mol%), TFA (10.0 equiv), DMSO (1 mL), under O_2 at 90 °C for 60 hours. [b]The yields were determined by ^1H NMR using dibromomethane as the internal standard.

Supplementary Table 2. Screening of Ni salts[a,b]

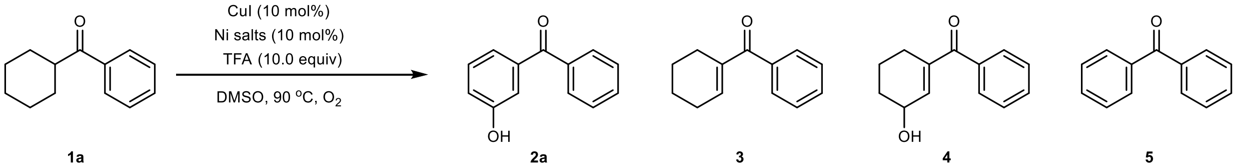

Entry	Ni salts	**2a** (%)	**3** (%)	**4** (%)	**5** (%)
1	NiI_2	19	trace	trace	<5
2	$NiCl_2$	33	trace	trace	<5
3	$NiBr_2$	32	7	trace	<5
4	$Ni(OAc)_2·4H_2O$	35	9	trace	<5
5	$NiBr_2(dme)$	38	8	trace	<5
6	$Ni(OTf)_2$	33	10	trace	<5
7	$Ni(acac)_2$(II)	26	trace	trace	<5
8	$NiCp_2$	29	trace	trace	<5
9	$Ni(PPh_3)_2Cl_2$	36	trace	trace	<5
10	$Ni(PPh_3)_2Br_2$	28	trace	trace	<5
11	$Ni(PCy_3)_2Cl_2$	30	trace	trace	<5

[a]Reaction conditions: **1a** (0.25 mmol), CuI (10 mol%), Ni salts (10 mol%), TFA (10.0 equiv), DMSO (1 mL), under O_2 at 90 °C for 60 hours. [b]The yields were determined by ^1H NMR using dibromomethane as the internal standard.

Supplementary Table 3. Screening of Fe salts[a,b]

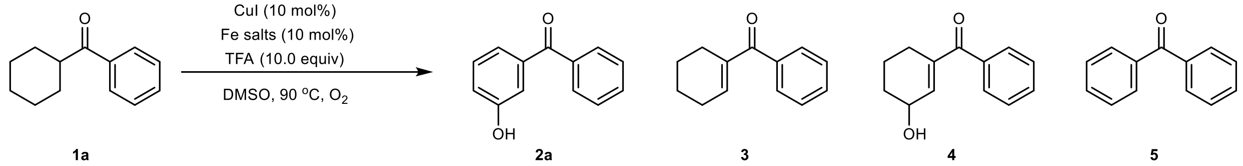

Entry	Fe salts	**2a** (%)	**3** (%)	**4** (%)	**5** (%)
1	Ferrocene	24	6	trace	<5
2	$K_3[Fe(CN)_6]$	21	8	trace	<5
3	$FeCl_2$	26	trace	trace	<5
4	$Fe(CO)_{12}$	19	5	trace	<5
5	$FeSO_4 \cdot 7H_2O$	31	7	trace	<5
6	$FeSO_4$	28	7	11	<5
7	$Fe(acac)_3$ (III)	17	5	7	<5
8	$Fe_2(C_2O_4)_3 \cdot 6H_2O$	25	5	9	<5
9	$Fe_2(CO)_9$	23	trace	8	<5

[a]Reaction conditions: **1a** (0.25 mmol), CuI (10 mol%), Fe salts (10 mol%), TFA (10.0 equiv), DMSO (1 mL), under O_2 at 90 °C for 60 hours. [b]The yields were determined by ^{1}H NMR using dibromomethane as the internal standard.

Supplementary Table 4. Screening of Co salts[a,b]

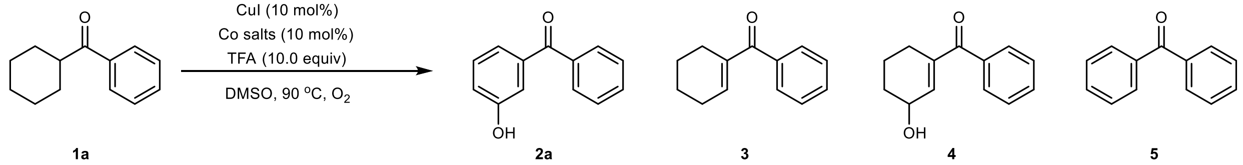

Entry	Co salts	**2a** (%)	**3** (%)	**4** (%)	**5** (%)
1	$Co(OAc)_2$	23	6	trace	<5
2	$Co(acac)_2$	16	5	trace	<5
3	$CoBr_2$	20	5	trace	<5
4	$Co(PPh_3)_3Cl$	21	7	trace	<5
5	$Co(C_2O_4) \cdot 2H_2O$	18	8	trace	<5

[a]Reaction conditions: **1a** (0.25 mmol), CuI (10 mol%), Co salts (10 mol%), TFA (10.0 equiv), DMSO (1 mL), under O_2 at 90 °C for 60 hours. [b]The yields were determined by ^{1}H NMR using dibromomethane as the internal standard.

Supplementary Table 5. Screening of Ag salts[a,b]

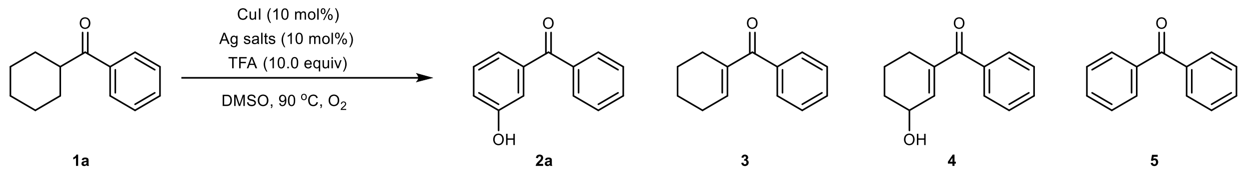

Entry	Catalyst	Ag salts	**2a** (%)	**3** (%)	**4** (%)	**5** (%)
1	CuI	AgOAc	53	17	13	6
2	CuI	AgTFA	47	18	15	7
3	CuI	AgOTf	50	17	14	7
4	CuI	$AgNO_3$	48	19	16	6
5	CuI	Ag_2O	-	-	-	-
6	CuI	Ag_2CO_3	-	-	-	-
7	CuI	Ag_3PO_4	-	-	-	-

[a]Reaction conditions: **1a** (0.25 mmol), CuI (10 mol%), Ag salts (10 mol%), TFA (10.0 equiv), DMSO (1 mL), under O_2 at 90 °C for 60 hours. [b]The yields were determined by ^{1}H NMR using dibromomethane as the internal standard.

Supplementary Table 6. Screening of the ratio of AgOAc to CuI[a,b]

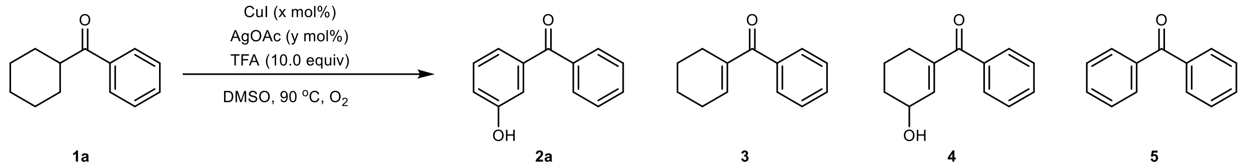

Entry	CuI (mol%)	AgOAc (mol%)	**2a** (%)	**3** (%)	**4** (%)	**5** (%)
1	10	10	53	17	13	6
2	10	11	32	21	14	7
3	10	12	-	-	-	-
4	10	20	-	-	-	-
5	20	10	45	trace	trace	<5
6	10	9	64	15	8	<5
7	10	8	60	13	6	<5
8	10	7	57	12	5	<5
9	10	6	58	10	trace	<5
10	10	5	54	8	trace	<5
11	10	2.5	47	7	trace	<5

[a]Reaction conditions: **1a** (0.25 mmol), CuI (x mol%), $Ag(OAc)_2$ (y mol%), TFA (10.0 equiv), DMSO (1 mL), under O_2 at 90 °C for 60 hours. [b]The yields were determined by ^{1}H NMR using dibromomethane as the internal standard.

Supplementary Table 7. Screening of water additive[a,b]

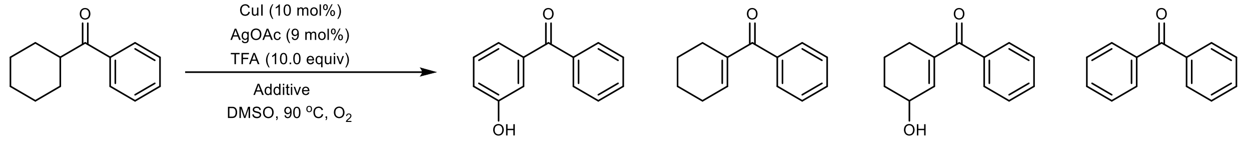

1a 2a 3 4 5

Entry	H_2O (uL)	**2a** (%)	**3** (%)	**4** (%)	**5** (%)
1	25	71	9	trace	<5
2	50	76 (71)[c]	trace	trace	<5
3	75	70	trace	trace	<5
4	100	63	6	trace	<5
5	200	54	17	trace	<5
6	300	51	10	trace	<5

[a]Reaction conditions: **1a** (0.25 mmol), CuI (10 mol%), $Ag(OAc)_2$ (9 mol%), TFA (10.0 equiv), DMSO (1 mL), H_2O (x uL), under O_2 at 90 °C for 60 hours. [b]The yields were determined by ^1H NMR using dibromomethane as the internal standard. [c]Isolated yield.

Supplementary Table 8. Control experiments[a,b]

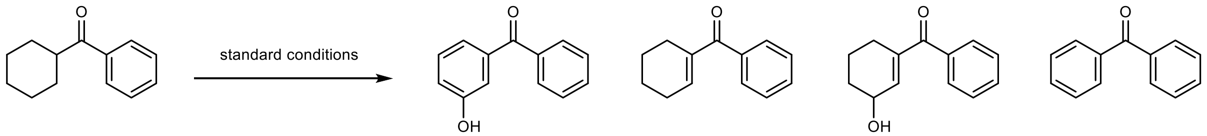

1a 2a 3 4 5

Entry	Catalyst	Oxidant	TFA (equiv)	additive	**2a** (%)	**3** (%)	**4** (%)	**5** (%)
1	CuI	AgOAc	10.0	H_2O	76 (71)[c]	trace	trace	<5
2	CuI	-	10.0	H_2O	54	trace	trace	<5
3	-	AgOAc	10.0	H_2O	-	-	-	-
4	CuI	AgOAc	-	H_2O	-	-	-	-
5	CuI	AgOAc	10.0	-	64	15	8	<5
6[d]	CuI	AgOAc	10.0	H_2O	68	trace	8	<5
7[e]	CuI	TBHP	10.0	-	73	trace	trace	<5

[a]Reaction conditions: **1a** (0.25 mmol), CuI (10 mol%), $Ag(OAc)_2$ (9 mol%), TFA (10.0 equiv), DMSO (1 mL), H_2O (50 uL), under O_2 at 90 °C for 60 hours. [b]The yields were determined by ^1H NMR using dibromomethane as the internal standard. [c]Isolated yield. [d]Under air. [e]TBHP (2.2 equiv) instead of AgOAc and O_2.

2.2 Condition optimization for synthesis of *meta*-carbonyl phenols

Supplementary Table 9. Screening of the ratio of aniline to TFA[a,b]

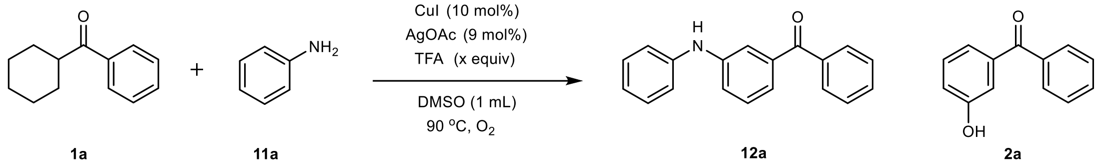

Entry	Aniline (equiv)	TFA (equiv)	**12a** (%)	**2a** (%)
1	1	1.0	14	5
2	1	2.0	21	9
3	1	3.0	39	12
4	1	4.0	43	15
5	1	5.0	48	13
6	1	6.0	45	10
7	1	8.0	31	<5
8	1.5	5.0	trace	-
9	5	10.0	nr	-
10	4	10.0	nr	-

[a]Reaction conditions: Reaction conditions: **1a** (0.25 mmol), **11a** (y equiv), CuI (10 mol%), $Ag(OAc)_2$ (9 mol%), TFA (x equiv), DMSO (1 mL), under O_2 at 90 °C for 60 hours. [b]The yields were determined by ^{1}H NMR using dibromomethane as the internal standard.

Supplementary Table 10. Screening of the ratio of aniline to TFA under silver-free conditions[a,b]

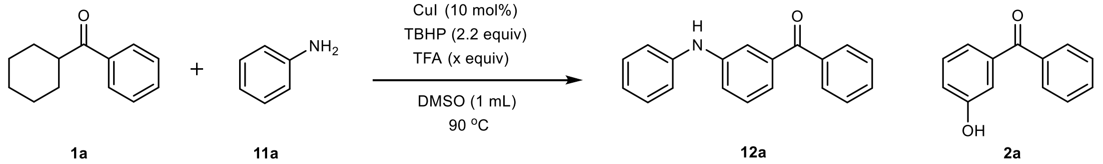

Entry	Aniline (equiv)	TFA (equiv)	**12a** (%)	**2a** (%)
1	1	10	34	9
2	1.5	10	59	<5
3	2	10	78 (73)[c]	trace
4	3	10	62	trace
5	4	10	60	trace
6	5	10	51	trace
7	1.5	5	53	trace
8	2	5	61	trace
9	2.5	5	36	trace
10	3	5	22	trace

[a]Reaction conditions: **1a** (0.25 mmol), **11a** (y equiv), CuI (10 mol%), TBHP (2.2 equiv), TFA (x equiv), DMSO (1 mL), at 90 °C for 60 hours. [b]The yields were determined by ^{1}H NMR using dibromomethane as the internal standard. [c]Isolated yield.

Supplementary Table 11. Screening of the TFA loading[a,b]

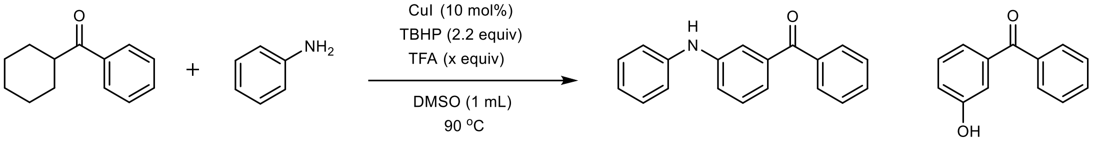

1a 11a 12a 2a

Entry	Aniline (equiv)	TFA (equiv)	**12a** (%)	**2a** (%)
1	2	2	trace	-
2	2	4	58	trace
3	2	6	62	trace
4	2	7	65	trace
5	2	8	67	trace
6	2	9	71	trace
7	2	10	78 (73)[c]	trace

[a]Reaction conditions: **1a** (0.25 mmol), **11a** (0.5 mmol), CuI (10 mol%), TBHP (2.2 equiv), TFA (x equiv), DMSO (1 mL), at 90 °C for 60 hours. [b]The yields were determined by ^1H NMR using dibromomethane as the internal standard. [c]Isolated yield.

Supplementary Table 12. Screening of the TBHP loading[a,b]

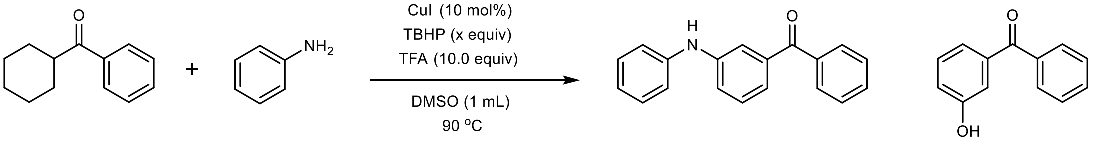

1a 11a 12a 2a

Entry	Aniline (equiv)	TBHP (equiv)	**12a** (%)	**2a** (%)
1	2	2.2	78 (73)[c]	trace
2	2	1.65	67	trace
3	2	1.1	62	trace
4	2	0.55	57	trace

[a]Reaction conditions: **1a** (0.25 mmol), **11a** (0.5 mmol), CuI (10 mol%), TBHP (x equiv), TFA (10.0 equiv), DMSO (1 mL), at 90 °C for 60 hours. [b]The yields were determined by ^1H NMR using dibromomethane as the internal standard. [c]Isolated yield.

3. Procedure for the Synthesis of Starting Materials and Products

3.1 Procedure for the synthesis of starting materials

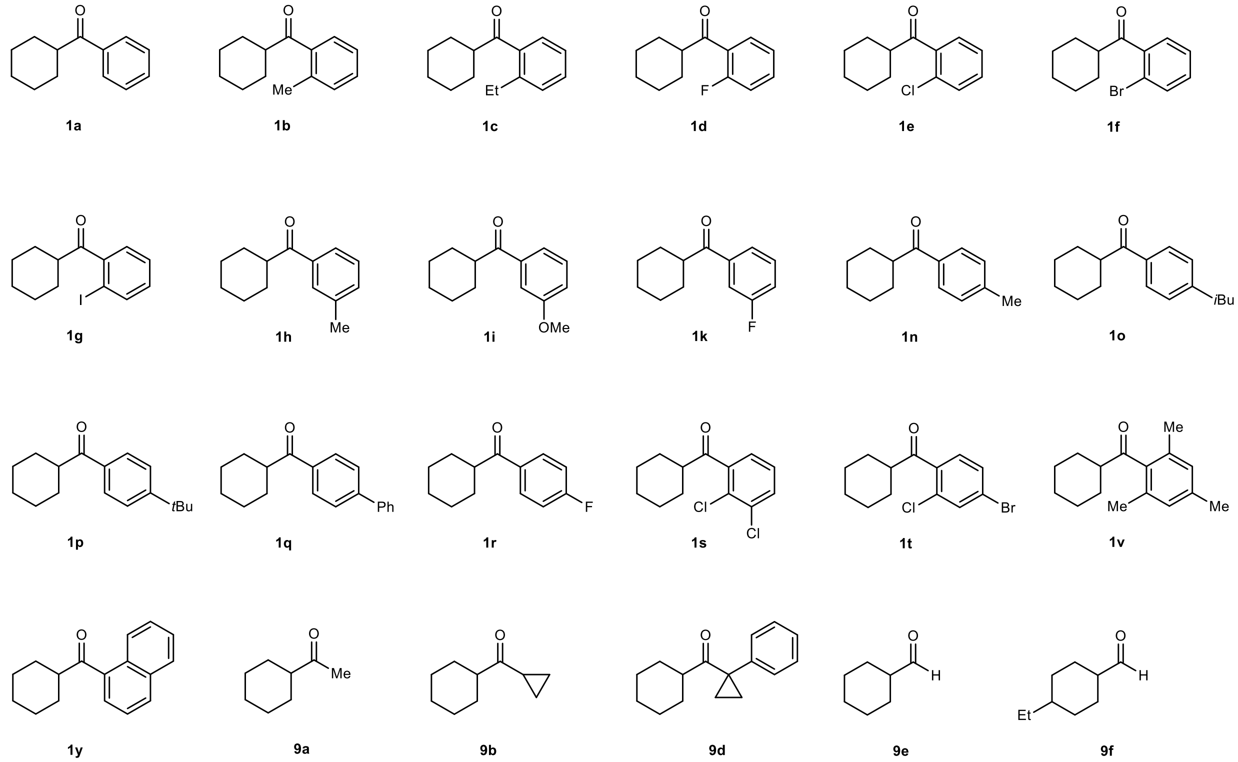

Supplementary Figure 1. Synthesis of starting materials according to general procedure A.

1a, **9a**, **9e** are commercially available. **1b-1i**, **1k**, **1n-1t**, **1v**, **1y**, **9b**, **9d** were prepared according to reported procedure[1]. **1b**[2], **1c**[3], **1d**[4], **1e-1g**[2], **1h**[5], **1i**[6], **1k**[7], **1n**[2], **1p**[8], **1q**[5], **1r**[2], **1v**[9], **1y**[10], **9b**[7], **9d**[11], **9f**[12] are known compounds and their characterization data were consistent with these reported in the literature. Other ketones were prepared by general procedure A.

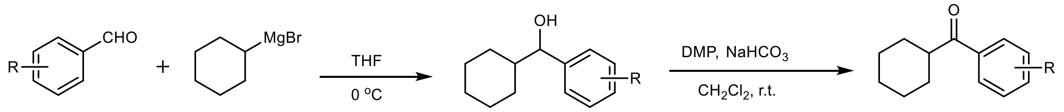

1st Step: To a solution of the corresponding aldehyde (15 mmol, 1.0 equiv) in anhydrous THF (25 mL) at 0 °C was added cyclohexylmagnesium bromide (1.0 M in THF, 19.5 mmol, 19.5 mL, 1.3 equiv) dropwise under argon. Upon completion, the mixture was quenched with saturated NH_4Cl (20 mL), extracted with EtOAc (3 × 50 mL). The combined extracts were washed with brine, dried over anhydrous Na_2SO_4, and concentrated to give the residue, which was further purified by silica gel column chromatography to afford the desired corresponding alcohols.

2nd Step[1]**:** To a solution of the preceding alcohols in dry DCM (30 mL) were added solid $NaHCO_3$ (2 equiv) and Dess-Martin periodinane (DMP, 1.5 equiv). The solution was stirred at room temperature and consumption of starting material was monitored by TLC. Upon

completion, the reaction was quenched by adding saturated $NaHCO_3$ (30 mL) and saturated $Na_2S_2O_3$ (30 mL) and stirred for 2 h. The mixture was extracted with CH_2Cl_2 (3 × 60 mL). The combined organic extracts were dried with Na_2SO_4, and concentrated to give the residue, which was further purified by silica gel column chromatography to afford the desired ketones.

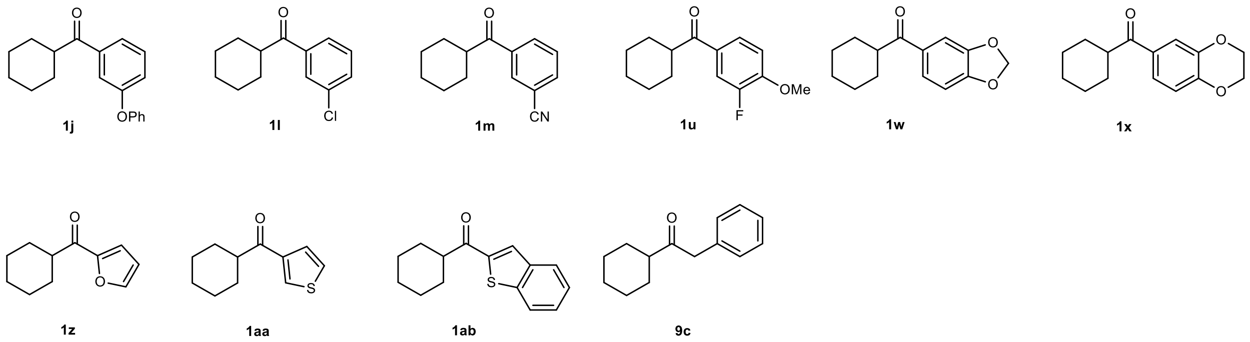

Supplementary Figure 2. Synthesis of starting materials according to general procedure B.

1j, **1l**, **1m**, **1u**, **1w**, **1x**, **1z, 1aa, 1ab, 9c** were prepared according to reported procedure[13]. **1l**[14], **1w**[7], **1z**[7], **1aa**[6], **1ab**[15], **9c**[16] are known compounds and their characterization data were consistent with these reported in the literature. Other ketones were prepared by general procedure B.

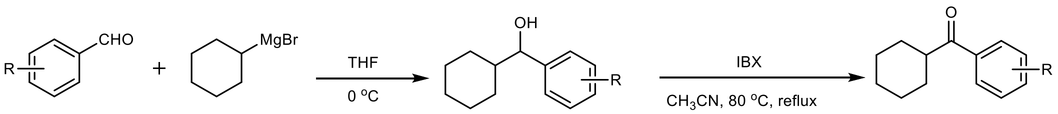

1st Step: To a solution of the corresponding aldehyde (15 mmol, 1.0 equiv) in anhydrous THF (25 mL) at 0 °C was added cyclohexylmagnesium bromide (1.0 M in THF, 19.5 mmol, 19.5 mL, 1.3 equiv) dropwise under argon. Upon completion, the mixture was quenched with saturated NH_4Cl (20 mL), extracted with EtOAc (3 × 50 mL). The combined extracts were washed with brine, dried over anhydrous Na_2SO_4, and concentrated to give the residue, which was further purified by silica gel column chromatography to afford the desired corresponding alcohols.

2nd Step[13]: To a solution of the preceding alcohols in CH_3CN (0.25 M) were added 2-Iodoxybenzoic acid (IBX, 2.0 equiv), then the mixture was refluxed and stirred for 1 h. Upon completion, the reaction mixture was cooled to room temperature. The mixture was filtered through a short path of silica gel and washed with EtOAc. Then the solution was concentrated by evaporation to give the residue, which was further purified by silica gel column chromatography to afford the desired ketones.

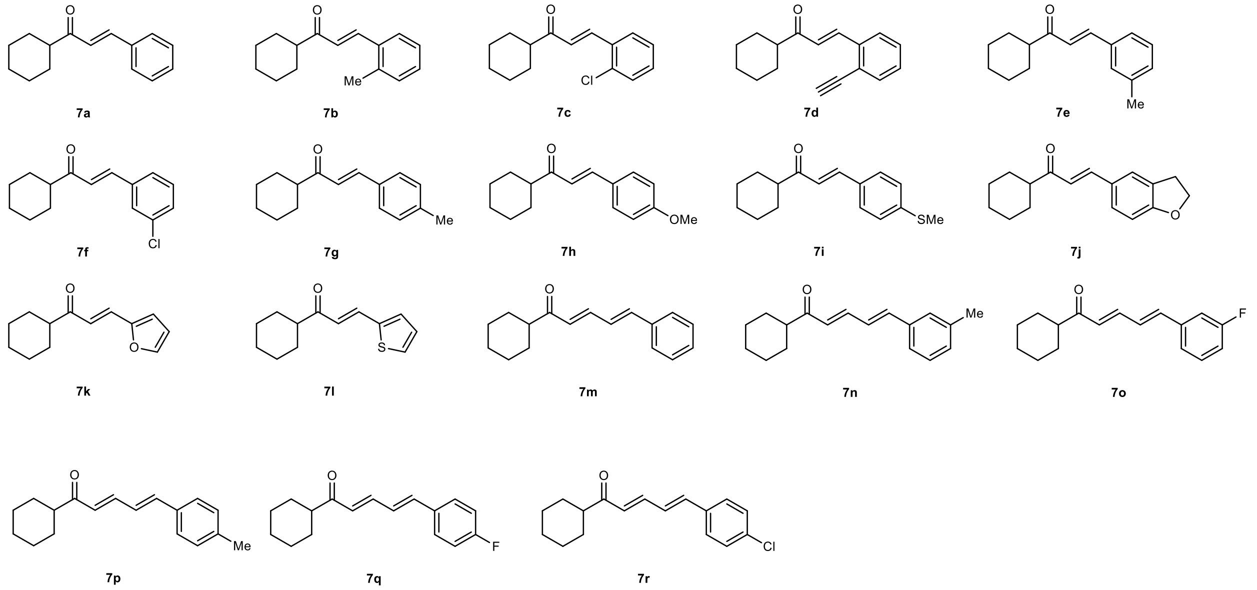

Supplementary Figure 3. Synthesis of starting materials according to general procedure C.

7a-7r were prepared according to reported procedure[17]. **7a**[5], **7b**[18], **7e**[18], **7g**[18], **7h**[19], **7l**[18] are known compounds and their characterization data were consistent with these reported in the literature. Other ketones were prepared by general procedure C.

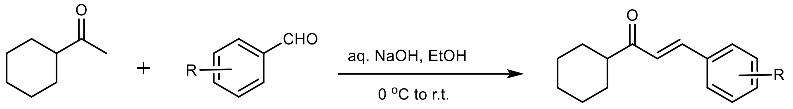

To a solution of 1-cyclohexylethan-1-one (554 mg, 4.4 mmol, 1.1 equiv) in EtOH (3 mL) at 0 °C was added dropwise a solution of NaOH (320 mg, 8 mmol, 2.0 equiv) in water (2 mL) over 15 min. The resulting solution was stirred for 30 min at 0 °C before the corresponding aldehyde (4 mmol, 1.0 equiv) in EtOH (1 mL) was added dropwise. The reaction mixture was warmed to room temperature gradually and stirred overnight. The solid product was collected by suction filtration on a Buchner funnel and washed repeatedly with cold ethanol. Recrystallization from ethanol or purification by silica gel chromatography for liquid products.

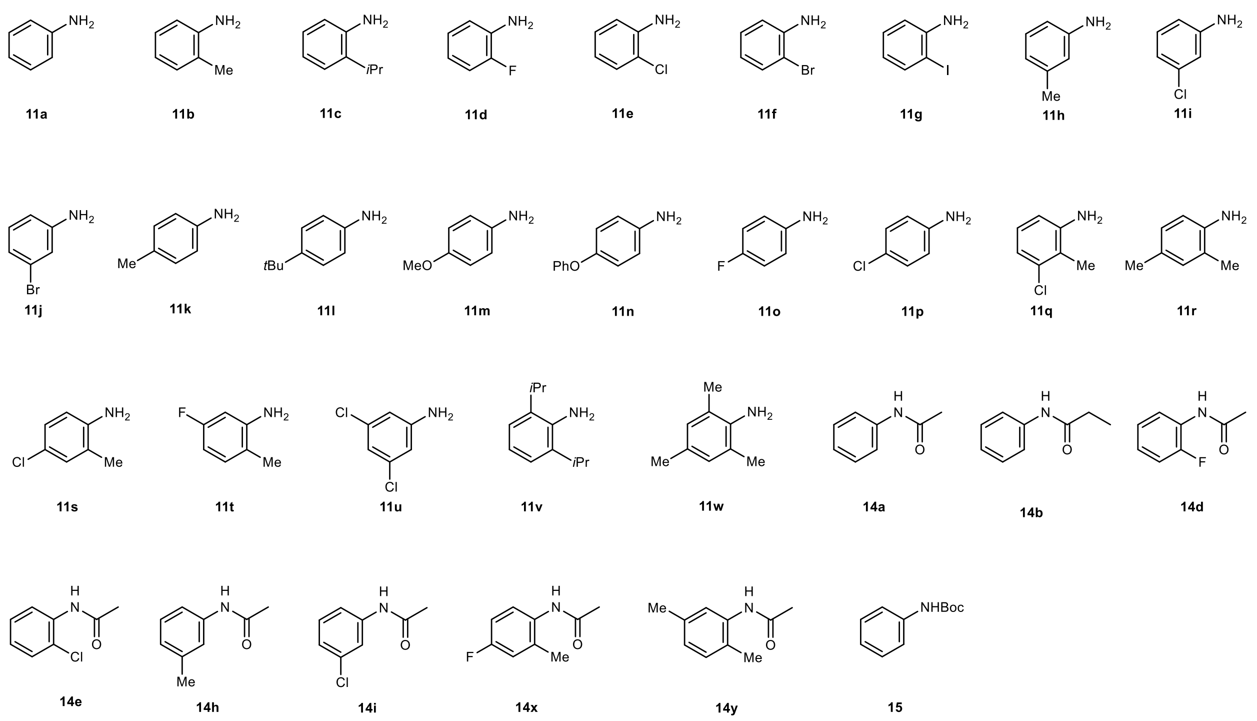

Supplementary Figure 4. Substrates of anilines.

11a-11w, **14a**, **14b**, **14d**, **14e**, **14h**, **14i**, **14x**, **15** are commercially available. **14y** was prepared according to reported procedure[20]. **14y** is known compounds and their characterization data were consistent with these reported in the literature[21].

Note: the NMR spectroscopy of new compounds **1j**, **1m**, **1o**, **1s**, **1t**, **1u**, **1x**, **7c**, **7d**, **7f**, **7i**, **7j**, **7k**, **7m**, **7n**, **7o**, **7p**, **7q**, **7r** were offered.

3.2 General procedure for the synthesis of *meta*-carbonyl phenols

General procedure D: A 15 mL sealed tube containing a magnetic stir bar was charged with ketones (0.25 mmol), CuI (4.8 mg, 10 mol%), AgOAc (3.7 mg, 9 mol%) and DMSO (1 mL) sequentially. The tube was evacuated and backfilled with O_2 three times. Subsequently, H_2O (50 uL, 11.1 equiv) and TFA (186 uL, 10.0 equiv) was added. The tube was sealed and the mixture was stirred at 90 °C for 60 h. After cooling to room temperature, the reaction mixture was diluted with EtOAc (50 mL) and washed with H_2O (3 × 1 mL) and brine (3 × 1 mL). The combined organic layer was dried over anhydrous Na_2SO_4, and concentrated to give the residue, which was further purified by silica gel column chromatography to afford the desired product.

Procedure for the synthesis of 10a

A 15 mL sealed tube containing a magnetic stir bar was charged with **9a** (0.25 mmol), CuI (4.8 mg, 10 mol%), $Pd(OAc)_2$ (8.4 mg, 15 mol%) and DMSO (1 mL) sequentially. The tube was evacuated and backfilled with O_2 three times. Subsequently, H_2O (50 uL, 11.1 equiv) and TFA (186 uL, 10.0 equiv) was added. The tube was sealed and the mixture was stirred at 90 °C for

60 h. After cooling to room temperature, the reaction mixture was diluted with EtOAc (50 mL) and washed with H_2O (3 × 1 mL) and brine (3 × 1 mL). The combined organic layer was dried over anhydrous Na_2SO_4, and concentrated to give the residue, which was further purified by silica gel column chromatography to afford the desired product.

3.3 Gram-scale for the synthesis of *meta*-carbonyl phenols

A 48 mL sealed tube containing a magnetic stir bar was charged with **1a** (5.5 mmol), CuI (105.6 mg, 10 mol%), AgOAc (81.4 mg, 9 mol%) and DMSO (22 mL) sequentially. The tube was evacuated and backfilled with O_2 three times. Subsequently, H_2O (1.1 mL, 11.1 equiv) and TFA (4.08 mL, 10.0 equiv) was added. The tube was sealed and the mixture was stirred at 90 °C for 72 h. After cooling to room temperature, the reaction mixture was diluted with EtOAc (800 mL) and washed with H_2O (3 × 15 mL) and brine (3 × 15 mL). The combined organic layer was dried over anhydrous Na_2SO_4, and concentrated to give the residue, which was further purified by silica gel column chromatography to afford the desired product.

3.4 General procedure for the synthesis of *meta*-carbonyl anilines

General procedure E: A 15 mL sealed tube containing a magnetic stir bar was charged with ketones (0.25 mmol), anilines (0.5 mmol), CuI (4.8 mg, 10 mol%), DMSO (1 mL) and TFA (186 uL, 10.0 equiv) sequentially. Subsequently, TBHP (100 uL, 2.2 equiv) was added. The tube was sealed and the mixture was stirred at 90 °C for 60 h. After cooling to room temperature, the reaction mixture was diluted with EtOAc (50 mL) and washed with H_2O (3 × 1 mL) and brine (3 × 1 mL). The combined organic layer was dried over anhydrous Na_2SO_4, and concentrated to give the residue, which was further purified by silica gel column chromatography to afford the desired product.

4. Characterization Data of Starting Materials and Products

4.1 Characterization data of starting materials

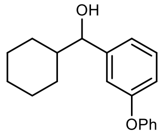

cyclohexyl(3-phenoxyphenyl)methanol (1j-1). Prepared according to general procedure B and purified by flash column chromatography to afford the product as a yellow liquid (2.83 g, 67% yield). Eluant: ethyl acetate/petroleum ether (1:8, R_f = 0.30).
^{1}H NMR (400 MHz, $CDCl_3$) δ 7.36 – 7.29 (m, 2H), 7.29 – 7.24 (m, 1H), 7.12 – 7.06 (m, 1H), 7.05 – 7.00 (m, 2H), 7.00 – 6.96 (m, 2H), 6.89 (dd, *J* = 8.1, 2.5 Hz, 1H), 4.33 (d, *J* = 7.0 Hz, 1H), 1.98 – 1.86 (m, 2H), 1.79 – 1.72 (m, 1H), 1.71 – 1.52 (m, 3H), 1.44 – 1.36 (m, 1H), 1.27 – 0.87 (m, 5H). ^{13}C NMR (101 MHz, $CDCl_3$) δ 157.4, 157.2, 145.9, 129.9 (2C), 129.6, 123.3, 121.6, 118.9 (2C), 117.8, 117.3, 79.1, 45.1, 29.4, 28.8, 26.5, 26.2, 26.1.
IR: 3372, 2921, 2850, 1581, 1484, 1443, 1239, 1212, 1072, 1022, 748, 691 cm^{-1}.
HRMS (ESI) m/z: $[M + Na]^+$ Calcd for $C_{19}H_{22}O_2Na$ 305.1512; Found 305.1507.

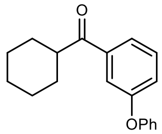

cyclohexyl(3-phenoxyphenyl)methanone (1j). Prepared according to general procedure B and purified by flash column chromatography to afford the product as a colorless liquid (2.48 g, 59% yield for two steps). Eluant: ethyl acetate/petroleum ether (1:25, R_f = 0.30).
^{1}H NMR (400 MHz, $CDCl_3$) δ 7.69 – 7.64 (m, 1H), 7.57 (dd, *J* = 2.5, 1.7 Hz, 1H), 7.44 – 7.38 (m, 1H), 7.38 – 7.31 (m, 2H), 7.22 – 7.10 (m, 2H), 7.05 – 6.99 (m, 2H), 3.19 (tt, *J* = 11.5, 3.2 Hz, 1H), 1.93 – 1.79 (m, 4H), 1.76 – 1.68 (m, 1H), 1.54 – 1.19 (m, 5H). ^{13}C NMR (101 MHz, $CDCl_3$) δ 203.3, 157.9, 156.8, 138.3, 130.0 (3C), 123.9, 123.1, 123.0, 119.2 (2C), 118.4, 45.9, 29.5 (2C), 26.0, 25.9 (2C).
IR: 3065, 2929, 2853, 1680, 1579, 1488, 1434, 1253, 1230, 910, 751, 692 cm^{-1}.
HRMS (ESI) m/z: $[M + Na]^+$ Calcd for $C_{19}H_{20}O_2Na$ 303.1356; Found 303.1362.

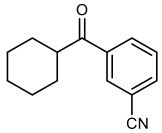

3-(cyclohexanecarbonyl)benzonitrile (1m). Prepared according to general procedure B and purified by flash column chromatography to afford the product as a yellow liquid (1.95 g, 61% yield for two steps). Eluant: ethyl acetate/petroleum ether (1:20, R_f = 0.30).
^{1}H NMR (400 MHz, $CDCl_3$) δ 8.23 – 8.20 (m, 1H), 8.19 – 8.14 (m, 1H), 7.86 – 7.81 (m, 1H), 7.65 – 7.59 (m, 1H), 3.22 (tt, *J* = 11.2, 3.1 Hz, 1H), 1.93 – 1.83 (m, 4H), 1.80 – 1.72 (m, 1H), 1.56 – 1.21 (m, 5H). ^{13}C NMR (101 MHz, $CDCl_3$) δ 201.8, 137.2, 135.7, 132.4, 132.1, 129.8, 118.2, 113.2, 45.8, 29.3 (2C), 25.9, 25.8 (2C).
IR: 3072, 2929, 2854, 2231, 1684, 1598, 1578, 1449, 1255, 1154, 983, 811, 753 cm^{-1}.
HRMS (ESI) m/z: $[M + Na]^+$ Calcd for $C_{14}H_{15}NONa$ 236.1046; Found 236.1056.

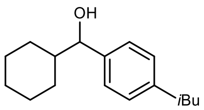

cyclohexyl(4-isobutylphenyl)methanol (1o-1). Prepared according to general procedure A and purified by flash column chromatography to afford the product as a white solid (2.62 g, 71% yield). m.p. = 52 – 54 °C. Eluant: ethyl acetate/petroleum ether (1:10, R_f = 0.30).
^{1}H NMR (400 MHz, $CDCl_3$) δ 7.22 – 7.17 (m, 2H), 7.13 – 7.08 (m, 2H), 4.31 (d, *J* = 7.3 Hz, 1H), 2.46 (d, *J* = 7.1 Hz, 2H), 2.05 – 1.96 (m, 1H), 1.92 – 1.80 (m, 2H), 1.79 – 1.71 (m, 1H), 1.70 – 1.55 (m, 3H), 1.39 – 1.31 (m, 1H), 1.29 – 0.94 (m, 5H), 0.90 (d, *J* = 6.6 Hz, 6H). ^{13}C NMR (101 MHz, $CDCl_3$) δ 141.04, 141.01, 129.1 (2C), 126.5 (2C), 79.5, 45.3, 45.0, 30.4, 29.5, 29.1, 26.6, 26.23, 26.15, 22.5 (2C).
IR: 3380, 2952, 2923, 2851, 1464, 1450, 1015, 847 cm^{-1}.
HRMS (ESI) m/z: [M + Na]$^+$ Calcd for $C_{17}H_{26}ONa$ 269.1876; Found 269.1881.

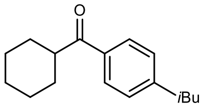

cyclohexyl(4-isobutylphenyl)methanone (1o). Prepared according to general procedure A and purified by flash column chromatography to afford the product as a colorless liquid (2.38 g, 65% yield for two steps). Eluant: ethyl acetate/petroleum ether (1:30, R_f = 0.30).
^{1}H NMR (400 MHz, $CDCl_3$) δ 7.90 – 7.84 (m, 2H), 7.25 – 7.18 (m, 2H), 3.25 (tt, *J* = 11.5, 3.2 Hz, 1H), 2.52 (d, *J* = 7.2 Hz, 2H), 1.96 – 1.79 (m, 5H), 1.78 – 1.67 (m, 1H), 1.58 – 1.19 (m, 5H), 0.91 (d, *J* = 6.6 Hz, 6H). ^{13}C NMR (101 MHz, $CDCl_3$) δ 203.7, 147.3, 134.2, 129.4 (2C), 128.4 (2C), 45.7, 45.5, 30.2, 29.6 (2C), 26.1, 26.0 (2C), 22.5 (2C).
IR: 2929, 2854, 1678, 1606, 1464, 1449, 1414, 1251, 1172, 975 cm^{-1}.
HRMS (ESI) m/z: [M + Na]$^+$ Calcd for $C_{17}H_{24}ONa$ 267.1719; Found 267.1722.

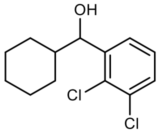

cyclohexyl(2,3-dichlorophenyl)methanol (1s-1). Prepared according to general procedure A and purified by flash column chromatography to afford the product as a white solid (2.67 g, 69% yield). m.p. = 68 – 70 °C. Eluant: ethyl acetate/petroleum ether (1:8, R_f = 0.30).
^{1}H NMR (400 MHz, $CDCl_3$) δ 7.41 (dd, *J* = 7.8, 1.6 Hz, 1H), 7.37 (dd, *J* = 7.9, 1.7 Hz, 1H), 7.24 – 7.19 (m, 1H), 4.94 (d, *J* = 5.8 Hz, 1H), 2.03 (br, 1H), 1.83 – 1.70 (m, 3H), 1.69 – 1.57 (m, 2H), 1.51 – 1.43 (m, 1H), 1.27 – 1.06 (m, 5H). ^{13}C NMR (101 MHz, $CDCl_3$) δ 143.7, 133.0, 130.6, 129.1, 127.3, 126.4, 75.5, 43.8, 29.7, 27.4, 26.5, 26.4, 26.1.
IR: 3370, 2924, 2850, 1448, 1419, 1177, 1154, 1043, 1016, 782, 739, 727 cm^{-1}.
HRMS (ESI) m/z: [M + Na]$^+$ Calcd for $C_{13}H_{16}OCl_2Na$ 281.0470; Found 281.0456.

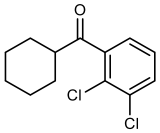

cyclohexyl(2,3-dichlorophenyl)methanone (1s). Prepared according to general procedure A and purified by flash column chromatography to afford the product as a white solid (2.38 g, 62% yield for two steps). m.p. = 51 – 53 °C. Eluant: ethyl acetate/petroleum ether (1:25, R_f = 0.30).

^{1}H NMR (400 MHz, $CDCl_3$) δ 7.51 (dd, J = 8.0, 1.6 Hz, 1H), 7.29 – 7.25 (m, 1H), 7.17 (dd, J = 7.6, 1.6 Hz, 1H), 2.98 (tt, J = 11.3, 3.5 Hz, 1H), 1.97 – 1.88 (m, 2H), 1.85 – 1.76 (m, 2H), 1.73 – 1.64 (m, 1H), 1.52 – 1.38 (m, 2H), 1.36 – 1.17 (m, 3H). ^{13}C NMR (101 MHz, $CDCl_3$) δ 206.8, 142.4, 134.0, 131.6, 128.8, 127.7, 126.1, 50.3, 28.4 (2C), 25.9, 25.7 (2C).
IR: 2927, 2853, 1700, 1448, 1408, 1248, 1180, 1107, 983, 796, 746, 734 cm^{-1}.
HRMS (ESI) m/z: [M + Na]$^+$ Calcd for $C_{13}H_{14}OCl_2Na$ 279.0314; Found 279.0322.

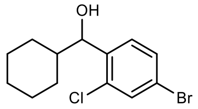

(4-bromo-2-chlorophenyl)(cyclohexyl)methanol (1t-1). Prepared according to general procedure A and purified by flash column chromatography to afford the product as a white solid (3.08 g, 68% yield). m.p. = 66 – 68 °C. Eluant: ethyl acetate/petroleum ether (1:10, R_f = 0.30).
^{1}H NMR (400 MHz, $CDCl_3$) δ 7.48 (d, J = 1.9 Hz, 1H), 7.42 – 7.34 (m, 2H), 4.85 (d, J = 6.2 Hz, 1H), 2.00 (br, 1H), 1.88 – 1.68 (m, 3H), 1.68 – 1.57 (m, 2H), 1.47 – 1.39 (m, 1H), 1.23 – 1.03 (m, 5H). ^{13}C NMR (101 MHz, $CDCl_3$) δ 140.4, 133.3, 131.9, 130.1, 129.6, 121.1, 74.6, 44.0, 29.5, 27.6, 26.44, 26.36, 26.1.
IR: 3369, 2922, 2850, 1582, 1556, 1466, 1448, 1378, 1080, 1047, 1015, 819, 743 cm^{-1}.
HRMS (ESI) m/z: [M + Na]$^+$ Calcd for $C_{13}H_{16}OClBrNa$ 324.9965; Found 324.9973.

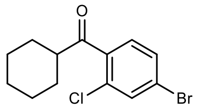

(4-bromo-2-chlorophenyl)(cyclohexyl)methanone (1t). Prepared according to general procedure A and purified by flash column chromatography to afford the product as a colorless liquid (2.70 g, 60% yield for two steps). Eluant: ethyl acetate/petroleum ether (1:50, R_f = 0.30).
^{1}H NMR (400 MHz, $CDCl_3$) δ 7.58 (d, J = 1.8 Hz, 1H), 7.45 (dd, J = 8.2, 1.8 Hz, 1H), 7.21 (d, J = 8.2 Hz, 1H), 3.02 (tt, J = 11.2, 3.4 Hz, 1H), 1.96 – 1.85 (m, 2H), 1.84 – 1.75 (m, 2H), 1.72 – 1.62 (m, 1H), 1.51 – 1.37 (m, 2H), 1.36 – 1.17 (m, 3H). ^{13}C NMR (101 MHz, $CDCl_3$) δ 206.4, 138.7, 133.0, 131.8, 130.2, 129.7, 124.5, 50.0, 28.5 (2C), 25.9, 25.7 (2C).
IR: 2926, 2852, 1694, 1576, 1549, 1463, 1447, 1366, 1201, 1083, 1065, 816, 788, 759 cm^{-1}.
HRMS (ESI) m/z: [M + Na]$^+$ Calcd for $C_{13}H_{14}OClBrNa$ 322.9809; Found 322.9805.

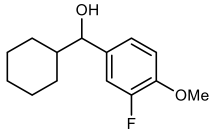

cyclohexyl(3-fluoro-4-methoxyphenyl)methanol (1u-1). Prepared according to general procedure B and purified by flash column chromatography to afford the product as a white solid (2.71 g, 76% yield). m.p. = 57 – 59 °C. Eluant: ethyl acetate/petroleum ether (1:5, R_f = 0.30).
^{1}H NMR (400 MHz, $CDCl_3$) δ 7.04 (dd, J = 12.3, 2.1 Hz, 1H), 7.00 – 6.95 (m, 1H), 6.94 – 6.87 (m, 1H), 4.29 (d, J = 7.1 Hz, 1H), 3.88 (s, 3H), 2.02 – 1.90 (m, 2H), 1.82 – 1.72 (m, 1H), 1.71 – 1.60 (m, 2H), 1.59 – 1.48 (m, 1H), 1.41 – 1.32 (m, 1H), 1.31 – 0.83 (m, 5H). ^{13}C NMR (101 MHz, $CDCl_3$) δ 152.3 (C-F, $^{1}J_{C-F}$ = 245.7 Hz), 146.9 (C-F, $^{2}J_{C-F}$ = 10.8 Hz), 136.9 (C-F, $^{3}J_{C-F}$ = 5.4 Hz), 122.4 (C-F, $^{3}J_{C-F}$ = 3.6 Hz), 114.4 (C-F, $^{2}J_{C-F}$ = 18.4 Hz), 113.0 (C-F, $^{4}J_{C-F}$ = 2.1 Hz), 78.6, 56.4, 45.0, 29.3, 28.9, 26.5, 26.13, 26.05. ^{19}F NMR (376 MHz, $CDCl_3$) δ -135.2 (dd, J = 12.2, 7.7 Hz).
IR: 3376, 2922, 2850, 1622, 1586, 1511, 1443, 1267, 1220, 1118, 1025, 813, 752 cm^{-1}.
HRMS (ESI) m/z: [M + Na]$^+$ Calcd for $C_{14}H_{19}O_2FNa$ 261.1261; Found 261.1252.

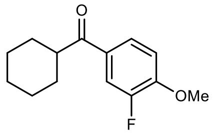

cyclohexyl(3-fluoro-4-methoxyphenyl)methanone (1u). Prepared according to general procedure B and purified by flash column chromatography to afford the product as a white solid (2.45 g, 69% yield for two steps). m.p. = 79 – 81 °C. Eluant: ethyl acetate/petroleum ether (1:15, R_f = 0.30).

^{1}H NMR (400 MHz, $CDCl_3$) δ 7.78 – 7.72 (m, 1H), 7.69 (dd, *J* = 12.0, 2.1 Hz, 1H), 7.04 – 6.97 (m, 1H), 3.95 (s, 3H), 3.17 (tt, *J* = 11.4, 3.2 Hz, 1H), 1.91 – 1.80 (m, 4H), 1.78 – 1.69 (m, 1H), 1.56 – 1.19 (m, 5H). ^{13}C NMR (101 MHz, $CDCl_3$) δ 201.6 (C-F, $^4J_{C-F}$ = 1.8 Hz), 152.2 (C-F, $^1J_{C-F}$ = 248.4 Hz), 151.7 (C-F, $^2J_{C-F}$ = 11.0 Hz), 129.6 (C-F, $^3J_{C-F}$ = 4.7 Hz), 125.5 (C-F, $^3J_{C-F}$ = 3.3 Hz), 116.1 (C-F, $^2J_{C-F}$ = 18.8 Hz), 112.4 (C-F, $^4J_{C-F}$ = 1.9 Hz), 56.4, 45.5, 29.6 (2C), 26.03, 25.95 (2C). ^{19}F NMR (376 MHz, $CDCl_3$) δ -134.3 (dd, *J* = 12.5, 8.2 Hz).

IR: 2929, 2853, 1672, 1609, 1580, 1514, 1430, 1314, 1267, 1114, 817, 761 cm^{-1}.

HRMS (ESI) m/z: [M + Na]$^+$ Calcd for $C_{14}H_{17}O_2FNa$ 259.1105; Found 259.1117.

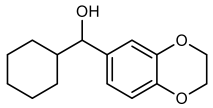

cyclohexyl(2,3-dihydrobenzo[*b*][1,4]dioxin-6-yl)methanol (1x-1). Prepared according to general procedure B and purified by flash column chromatography to afford the product as a colorless liquid (2.86 g, 77% yield). Eluant: ethyl acetate/petroleum ether (1:6, R_f = 0.30).

^{1}H NMR (400 MHz, $CDCl_3$) δ 6.83 – 6.78 (m, 2H), 6.75 (dd, *J* = 8.3, 2.0 Hz, 1H), 4.27 – 4.20 (m, 5H), 2.03 – 1.94 (m, 1H), 1.87 (br, 1H), 1.80 – 1.71 (m, 1H), 1.70 – 1.59 (m, 2H), 1.59 – 1.49 (m, 1H), 1.41 – 1.33 (m, 1H), 1.30 – 0.82 (m, 5H). ^{13}C NMR (101 MHz, $CDCl_3$) δ 143.4, 142.9, 137.3, 119.8, 117.0, 115.6, 79.1, 64.50, 64.46, 45.0, 29.4, 29.1, 26.6, 26.2, 26.1.

IR: 3400, 2921, 2850, 1590, 1503, 1449, 1432, 1281, 1255, 1100, 816, 736 cm^{-1}.

HRMS (ESI) m/z: [M + Na]$^+$ Calcd for $C_{15}H_{20}O_3Na$ 271.1305; Found 271.1299.

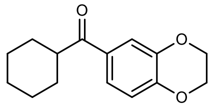

cyclohexyl(2,3-dihydrobenzo[*b*][1,4]dioxin-6-yl)methanone (1x). Prepared according to general procedure B and purified by flash column chromatography to afford the product as a colorless liquid (2.58 g, 70% yield for two steps). Eluant: ethyl acetate/petroleum ether (1:10, R_f = 0.30).

^{1}H NMR (400 MHz, $CDCl_3$) δ 7.53 – 7.46 (m, 2H), 6.93 – 6.88 (m, 1H), 4.34 – 4.25 (m, 4H), 3.17 (tt, *J* = 11.5, 3.2 Hz, 1H), 1.90 – 1.79 (m, 4H), 1.77 – 1.68 (m, 1H), 1.55 – 1.19 (m, 5H). ^{13}C NMR (101 MHz, $CDCl_3$) δ 202.4, 147.8, 143.4, 130.2, 122.4, 117.9, 117.3, 64.8, 64.2, 45.4, 29.7 (2C), 26.1, 26.0 (2C).

IR: 2926, 2852, 1668, 1603, 1579, 1504, 1449, 1427, 1282, 1256, 1064, 884, 775, 734 cm^{-1}.

HRMS (ESI) m/z: [M + Na]$^+$ Calcd for $C_{15}H_{18}O_3Na$ 269.1148; Found 269.1154.

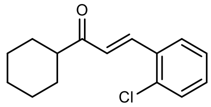

(*E*)-3-(2-chlorophenyl)-1-cyclohexylprop-2-en-1-one (7c). Prepared according to general procedure C and purified by flash column chromatography to afford the product as a white solid (0.91 g, 92% yield). m.p. = 62 – 64 °C. Eluant: ethyl acetate/petroleum ether (1:20, R_f = 0.30).
^{1}H NMR (400 MHz, $CDCl_3$) δ 7.99 (d, *J* = 16.1 Hz, 1H), 7.65 (dd, *J* = 7.3, 2.2 Hz, 1H), 7.42 (dd, *J* = 7.4, 1.9 Hz, 1H), 7.36 – 7.24 (m, 2H), 6.76 (d, *J* = 16.1 Hz, 1H), 2.71 (tt, *J* = 11.2, 3.4 Hz, 1H), 1.96 – 1.88 (m, 2H), 1.88 – 1.79 (m, 2H), 1.76 – 1.65 (m, 1H), 1.52 – 1.17 (m, 5H). ^{13}C NMR (101 MHz, $CDCl_3$) δ 203.1, 138.1, 135.4, 133.2, 131.1, 130.3, 127.7, 127.4, 127.2, 49.2, 28.9 (2C), 26.0, 25.8 (2C).
IR: 2926, 2852, 1686, 1659, 1605, 1468, 1441, 1143, 1009, 976, 752 cm^{-1}.
HRMS (ESI) m/z: [M + Na]$^+$ Calcd for $C_{15}H_{17}OClNa$ 271.0860; Found 271.0865.

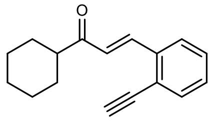

(*E*)-1-cyclohexyl-3-(2-ethynylphenyl)prop-2-en-1-one (7d). Prepared according to general procedure C and purified by flash column chromatography to afford the product as a yellow solid (0.84 g, 88% yield). m.p. = 65 – 67 °C. Eluant: ethyl acetate/petroleum ether (1:20, R_f = 0.30).
^{1}H NMR (400 MHz, $CDCl_3$) δ 8.09 (d, *J* = 16.2 Hz, 1H), 7.66 (dd, *J* = 7.6, 1.7 Hz, 1H), 7.54 (dd, *J* = 7.4, 1.7 Hz, 1H), 7.42 – 7.29 (m, 2H), 6.85 (d, *J* = 16.1 Hz, 1H), 3.44 (s, 1H), 2.71 (tt, *J* = 11.2, 3.4 Hz, 1H), 1.96 – 1.88 (m, 2H), 1.87 – 1.79 (m, 2H), 1.76 – 1.66 (m, 1H), 1.50 – 1.17 (m, 5H). ^{13}C NMR (101 MHz, $CDCl_3$) δ 203.3, 139.8, 136.9, 133.6, 129.8, 129.2, 126.7, 126.1, 123.2, 83.5, 81.3, 49.3, 28.9 (2C), 26.0, 25.9 (2C).
IR: 3296, 3241, 2926, 2851, 1683, 1656, 1606, 1592, 1474, 1447, 1318, 978, 754 cm^{-1}.
HRMS (ESI) m/z: [M + Na]$^+$ Calcd for $C_{17}H_{18}ONa$ 261.1250; Found 261.1245.

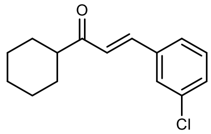

(*E*)-3-(3-chlorophenyl)-1-cyclohexylprop-2-en-1-one (7f). Prepared according to general procedure C and purified by flash column chromatography to afford the product as a white solid (0.90 g, 91% yield). m.p. = 52 – 54 °C. Eluant: ethyl acetate/petroleum ether (1:20, R_f = 0.30).
^{1}H NMR (400 MHz, $CDCl_3$) δ 7.59 – 7.48 (m, 2H), 7.42 (dt, *J* = 6.9, 1.7 Hz, 1H), 7.38 – 7.29 (m, 2H), 6.81 (d, *J* = 16.0 Hz, 1H), 2.63 (tt, *J* = 11.2, 3.4 Hz, 1H), 1.97 – 1.87 (m, 2H), 1.87 – 1.79 (m, 2H), 1.77 – 1.67 (m, 1H), 1.50 – 1.17 (m, 5H). ^{13}C NMR (101 MHz, $CDCl_3$) δ 202.8, 140.7, 136.8, 135.0, 130.3, 130.2, 127.9, 126.7, 125.9, 49.8, 28.7 (2C), 26.0, 25.9 (2C).
IR: 2925, 2852, 1686, 1657, 1609, 1563, 1197, 1144, 1010, 979, 781 cm^{-1}.
HRMS (ESI) m/z: [M + Na]$^+$ Calcd for $C_{15}H_{17}OClNa$ 271.0860; Found 271.0866.

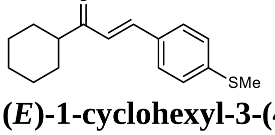

(*E*)-1-cyclohexyl-3-(4-(methylthio)phenyl)prop-2-en-1-one (7i). Prepared according to general procedure C and purified by flash column chromatography to afford the product as a yellow solid (0.90 g, 87% yield). m.p. = 79 – 81 °C. Eluant: ethyl acetate/petroleum ether (1:15, R_f = 0.30).

^{1}H NMR (400 MHz, $CDCl_3$) δ 7.54 (d, *J* = 16.0 Hz, 1H), 7.49 – 7.44 (m, 2H), 7.25 – 7.20 (m, 2H), 6.77 (d, *J* = 16.0 Hz, 1H), 2.64 (tt, *J* = 11.3, 3.3 Hz, 1H), 2.50 (s, 3H), 1.94 – 1.86 (m, 2H), 1.86 – 1.78 (m, 2H), 1.75 – 1.67 (m, 1H), 1.49 – 1.16 (m, 5H). ^{13}C NMR (101 MHz, $CDCl_3$) δ 203.2, 142.1, 141.8, 131.3, 128.7 (2C), 126.1 (2C), 123.8, 49.5, 28.9 (2C), 26.0, 25.9 (2C), 15.3.
IR: 2924, 2853, 1678, 1600, 1589, 1493, 1404, 1320, 1082, 1014, 983, 810, 747 cm^{-1}.
HRMS (ESI) m/z: [M + Na]$^+$ Calcd for $C_{16}H_{20}OSNa$ 283.1127; Found 283.1134.

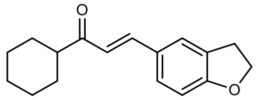

(*E*)-1-cyclohexyl-3-(2,3-dihydrobenzofuran-5-yl)prop-2-en-1-one (7j). Prepared according to general procedure C and purified by flash column chromatography to afford the product as a yellow solid (0.94 g, 92% yield). m.p. = 110 – 112 °C. Eluant: ethyl acetate/petroleum ether (1:15, R_f = 0.30).
^{1}H NMR (400 MHz, $CDCl_3$) δ 7.55 (d, *J* = 16.0 Hz, 1H), 7.46 – 7.43 (m, 1H), 7.34 (dd, *J* = 8.4, 1.9 Hz, 1H), 6.78 (d, *J* = 8.2 Hz, 1H), 6.67 (d, *J* = 15.9 Hz, 1H), 4.62 (t, *J* = 8.7 Hz, 2H), 3.23 (t, *J* = 8.7 Hz, 2H), 2.63 (tt, *J* = 11.3, 3.3 Hz, 1H), 1.93 – 1.78 (m, 4H), 1.77 – 1.65 (m, 1H), 1.52 – 1.17 (m, 5H). ^{13}C NMR (101 MHz, $CDCl_3$) δ 203.4, 162.5, 142.6, 130.0, 128.2, 127.6, 124.8, 122.0, 109.8, 72.0, 49.5, 29.4, 29.0 (2C), 26.0, 25.9 (2C).
IR: 2927, 2853, 1678, 1649, 1586, 1490, 1442, 1241, 981, 815 cm^{-1}.
HRMS (ESI) m/z: [M + Na]$^+$ Calcd for $C_{17}H_{20}O_2Na$ 279.1356; Found 279.1361.

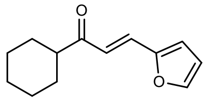

(*E*)-1-cyclohexyl-3-(furan-2-yl)prop-2-en-1-one (7k). Prepared according to general procedure C and purified by flash column chromatography to afford the product as a brown solid (0.63 g, 77% yield). m.p. = 56 – 58 °C. Eluant: ethyl acetate/petroleum ether (1:20, R_f = 0.30).
^{1}H NMR (400 MHz, $CDCl_3$) δ 7.48 (d, *J* = 1.4 Hz, 1H), 7.36 (d, *J* = 15.7 Hz, 1H), 6.73 (d, *J* = 15.7 Hz, 1H), 6.65 (d, *J* = 3.4 Hz, 1H), 6.48 (dd, *J* = 3.4, 1.8 Hz, 1H), 2.57 (tt, *J* = 11.3, 3.4 Hz, 1H), 1.98 – 1.86 (m, 2H), 1.86 – 1.78 (m, 2H), 1.74 – 1.66 (m, 1H), 1.49 – 1.16 (m, 5H). ^{13}C NMR (101 MHz, $CDCl_3$) δ 202.9, 151.5, 144.8, 128.6, 122.1, 115.6, 112.6, 50.0, 28.8 (2C), 26.0, 25.9 (2C).
IR: 2927, 2853, 1680, 1607, 1553, 1449, 1315, 1199, 1144, 1013, 973, 746 cm^{-1}.
HRMS (ESI) m/z: [M + Na]$^+$ Calcd for $C_{13}H_{16}O_2Na$ 227.1043; Found 227.1048.

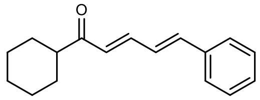

(2*E*,4*E*)-1-cyclohexyl-5-phenylpenta-2,4-dien-1-one (7m). Prepared according to general procedure C and purified by flash column chromatography to afford the product as a yellow solid (0.51 g, 53% yield). m.p. = 43 – 45 °C. Eluant: ethyl acetate/petroleum ether (1:20, R_f = 0.30).
^{1}H NMR (400 MHz, $CDCl_3$) δ 7.50 – 7.44 (m, 2H), 7.41 – 7.28 (m, 4H), 7.01 – 6.83 (m, 2H), 6.35 (d, *J* = 15.2 Hz, 1H), 2.59 (tt, *J* = 11.4, 3.3 Hz, 1H), 1.91 – 1.77 (m, 4H), 1.74 – 1.69 (m, 1H), 1.47 – 1.16 (m, 5H). ^{13}C NMR (101 MHz, $CDCl_3$) δ 203.6, 142.4, 141.3, 136.3, 129.2, 129.0 (2C), 128.4, 127.3 (2C), 127.0, 49.3, 28.9 (2C), 26.0, 25.9 (2C).
IR: 2925, 2851, 1677, 1650, 1614, 1584, 1447, 1120, 1064, 997, 751, 690 cm^{-1}.
HRMS (ESI) m/z: [M + Na]$^+$ Calcd for $C_{17}H_{20}ONa$ 263.1406; Found 263.1401.

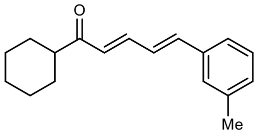

(2*E*,4*E*)-1-cyclohexyl-5-(m-tolyl)penta-2,4-dien-1-one (7n). Prepared according to general procedure C and purified by flash column chromatography to afford the product as a yellow solid (0.79 g, 78% yield). m.p. = 51 – 53 °C. Eluant: ethyl acetate/petroleum ether (1:20, R_f = 0.30).
^{1}H NMR (400 MHz, $CDCl_3$) δ 7.41 – 7.32 (m, 1H), 7.31 – 7.24 (m, 3H), 7.15 – 7.10 (m, 1H), 6.96 – 6.82 (m, 2H), 6.34 (d, *J* = 15.3 Hz, 1H), 2.59 (tt, *J* = 11.3, 3.3 Hz, 1H), 2.36 (s, 3H), 1.90 – 1.78 (m, 4H), 1.74 – 1.66 (m, 1H), 1.47 – 1.15 (m, 5H). ^{13}C NMR (101 MHz, $CDCl_3$) δ 203.6, 142.5, 141.4, 138.6, 136.2, 130.1, 128.8, 128.2, 128.0, 126.8, 124.5, 49.3, 28.9 (2C), 26.0, 25.9 (2C), 21.5.
IR: 2924, 2852, 1701, 1604, 1585, 1449, 1245, 1141, 998, 779, 689 cm^{-1}.
HRMS (ESI) m/z: [M + Na]$^+$ Calcd for $C_{18}H_{22}ONa$ 277.1563; Found 277.1556.

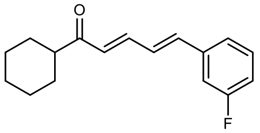

(2*E*,4*E*)-1-cyclohexyl-5-(3-fluorophenyl)penta-2,4-dien-1-one (7o). Prepared according to general procedure C and purified by flash column chromatography to afford the product as a yellow liquid (0.86 g, 83% yield). Eluant: ethyl acetate/petroleum ether (1:15, R_f = 0.30).
^{1}H NMR (400 MHz, $CDCl_3$) δ 7.39 – 7.29 (m, 2H), 7.25 – 7.20 (m, 1H), 7.19 – 7.13 (m, 1H), 7.04 – 6.97 (m, 1H), 6.96 – 6.82 (m, 2H), 6.38 (d, *J* = 15.3 Hz, 1H), 2.58 (tt, *J* = 11.3, 3.3 Hz, 1H), 1.92 – 1.76 (m, 4H), 1.74 – 1.66 (m, 1H), 1.48 – 1.16 (m, 5H). ^{13}C NMR (101 MHz, $CDCl_3$) δ 203.4, 163.2 (C-F, $^1J_{C\text{-}F}$ = 246.2 Hz), 141.7, 139.6 (C-F, $^4J_{C\text{-}F}$ = 2.9 Hz), 138.5 (C-F, $^3J_{C\text{-}F}$ = 7.7 Hz), 130.4 (C-F, $^3J_{C\text{-}F}$ = 8.4 Hz), 129.1, 128.2, 123.3 (C-F, $^4J_{C\text{-}F}$ = 2.8 Hz), 116.0 (C-F, $^2J_{C\text{-}F}$ = 21.6 Hz), 113.4 (C-F, $^2J_{C\text{-}F}$ = 22.0 Hz), 49.4, 28.8 (2C), 26.0, 25.9 (2C). ^{19}F NMR (376 MHz, $CDCl_3$) δ -112.8 (q, *J* = 8.9 Hz).
IR: 2927, 2854, 1698, 1613, 1584, 1486, 1447, 1246, 1142, 966, 782, 750 cm^{-1}.
HRMS (ESI) m/z: [M + H]$^+$ Calcd for $C_{17}H_{20}OF$ 259.1493; Found 259.1499.

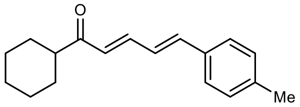

(2*E*,4*E*)-1-cyclohexyl-5-(*p*-tolyl)penta-2,4-dien-1-one (7p). Prepared according to general procedure C and purified by flash column chromatography to afford the product as a yellow solid (0.81 g, 80% yield). m.p. = 92 – 94 °C. Eluant: ethyl acetate/petroleum ether (1:20, R_f = 0.30).
^{1}H NMR (400 MHz, $CDCl_3$) δ 7.40 – 7.32 (m, 3H), 7.19 – 7.14 (m, 2H), 6.96 – 6.78 (m, 2H), 6.33 (d, *J* = 15.3 Hz, 1H), 2.58 (tt, *J* = 11.4, 3.3 Hz, 1H), 2.36 (s, 3H), 1.90 – 1.77 (m, 4H), 1.73 – 1.69 (m, 1H), 1.46 – 1.15 (m, 5H). ^{13}C NMR (101 MHz, $CDCl_3$) δ 203.6, 142.7, 141.4, 139.5, 133.5, 129.7 (2C), 127.8, 127.3 (2C), 126.0, 49.3, 28.9 (2C), 26.0, 25.9 (2C), 21.5.
IR: 2923, 2851, 1673, 1582, 1446, 1140, 1063, 1000, 833, 802 cm^{-1}.
HRMS (ESI) m/z: [M + Na]$^+$ Calcd for $C_{18}H_{22}ONa$ 277.1563; Found 277.1553.

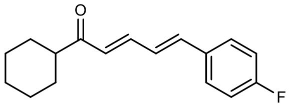

(2*E*,4*E*)-1-cyclohexyl-5-(4-fluorophenyl)penta-2,4-dien-1-one (7q). Prepared according to general procedure C and purified by flash column chromatography to afford the product as a yellow solid (0.74 g, 72% yield). m.p. = 62 – 64 °C. Eluant: ethyl acetate/petroleum ether (1:15, R_f = 0.30).

^{1}H NMR (400 MHz, $CDCl_3$) δ 7.47 – 7.41 (m, 2H), 7.35 (dd, *J* = 15.2, 10.7 Hz, 1H), 7.09 – 7.01 (m, 2H), 6.94 – 6.75 (m, 2H), 6.35 (d, *J* = 15.3 Hz, 1H), 2.58 (tt, *J* = 11.4, 3.3 Hz, 1H), 1.95 – 1.78 (m, 4H), 1.74 – 1.69 (m, 1H), 1.47 – 1.16 (m, 5H). ^{13}C NMR (101 MHz, $CDCl_3$) δ 203.5, 163.2 (C-F, $^1J_{C-F}$ = 249.9 Hz), 142.2, 139.8, 132.5 (C-F, $^4J_{C-F}$ = 3.5 Hz), 129.0 (C-F, $^3J_{C-F}$ = 8.2 Hz, 2C), 128.3, 126.7 (C-F, $^5J_{C-F}$ = 2.5 Hz), 116.0 (C-F, $^2J_{C-F}$ = 21.8 Hz, 2C), 49.4, 28.9 (2C), 26.0, 25.9 (2C). ^{19}F NMR (376 MHz, $CDCl_3$) δ -111.4 (m).

IR: 2926, 2853, 1677, 1618, 1581, 1507, 1449, 1231, 1157, 1141, 997, 840 cm^{-1}.

HRMS (ESI) m/z: $[M + Na]^+$ Calcd for $C_{17}H_{19}OFNa$ 281.1312; Found 281.1307.

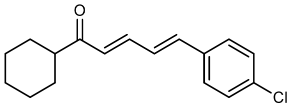

(2*E*,4*E*)-5-(4-chlorophenyl)-1-cyclohexylpenta-2,4-dien-1-one (7r). Prepared according to general procedure C and purified by flash column chromatography to afford the product as a yellow solid (0.94 g, 86% yield). m.p. = 124 – 126 °C. Eluant: ethyl acetate/petroleum ether (1:20, R_f = 0.30).

^{1}H NMR (400 MHz, $CDCl_3$) δ 7.42 – 7.28 (m, 5H), 6.93 – 6.78 (m, 2H), 6.36 (d, *J* = 15.3 Hz, 1H), 2.58 (tt, *J* = 11.4, 3.3 Hz, 1H), 1.90 – 1.77 (m, 4H), 1.75 – 1.69 (m, 1H), 1.48 – 1.14 (m, 5H). ^{13}C NMR (101 MHz, $CDCl_3$) δ 203.4, 142.0, 139.7, 134.9, 134.7, 129.2 (2C), 128.7, 128.4 (2C), 127.5, 49.4, 28.8 (2C), 26.0, 25.9 (2C).

IR: 2923, 2852, 1675, 1583, 1490, 1448, 1239, 1141, 1087, 1062, 998, 837, 809 cm^{-1}.

HRMS (ESI) m/z: $[M + Na]^+$ Calcd for $C_{17}H_{19}OClNa$ 297.1017; Found 297.1026.

4.2 Characterization data of products

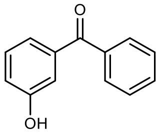

(3-hydroxyphenyl)(phenyl)methanone (2a). Prepared according to general procedure D and purified by flash column chromatography to afford the product as a yellow solid (35.1 mg, 71% yield). m.p. = 108 – 110 °C. Eluant: ethyl acetate/petroleum ether (1:5, R_f = 0.30).

^{1}H NMR (400 MHz, $CDCl_3$) δ 7.85 – 7.76 (m, 2H), 7.63 – 7.55 (m, 1H), 7.50 – 7.43 (m, 2H), 7.42 – 7.38 (m, 1H), 7.36 – 7.27 (m, 2H), 7.13 – 7.08 (m, 1H), 6.42 (br, 1H). ^{13}C NMR (101 MHz, $CDCl_3$) δ 197.4, 156.2, 138.9, 137.5, 132.8, 130.3 (2C), 129.7, 128.5 (2C), 123.0, 120.2, 116.7.

IR: 3352, 3061, 2924, 1643, 1594, 1581, 1448, 1319, 1288, 1234, 843, 725, 707 cm^{-1}.

HRMS (ESI) m/z: $[M + Na]^+$ Calcd for $C_{13}H_{10}O_2Na$ 221.0573; Found 221.0566.

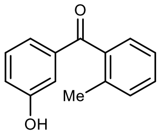

(3-hydroxyphenyl)(*o*-tolyl)methanone (2b). Prepared according to general procedure D and purified by flash column chromatography to afford the product as a yellow solid (42.9 mg, 81% yield). m.p. = 99 – 101 °C. Eluant: ethyl acetate/petroleum ether (1:5, R_f = 0.3).
^{1}H NMR (400 MHz, $CDCl_3$) δ 7.43 – 7.36 (m, 2H), 7.34 – 7.21 (m, 5H), 7.11 – 7.06 (m, 1H), 5.77 (br, 1H), 2.33 (s, 3H). ^{13}C NMR (101 MHz, $CDCl_3$) δ 199.3, 156.3, 139.2, 138.5, 137.0, 131.2, 130.6, 129.9, 128.8, 125.3, 123.3, 120.9, 116.4, 20.1.
IR: 3329, 3062, 2925, 1646, 1595, 1583, 1479, 1448, 1296, 1229, 768, 742 cm^{-1}.
HRMS (ESI) m/z: [M + Na]$^+$ Calcd for $C_{14}H_{12}O_2Na$ 235.0730; Found 235.0722.

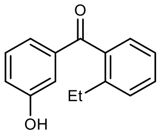

(2-ethylphenyl)(3-hydroxyphenyl)methanone (2c). Prepared according to general procedure D and purified by flash column chromatography to afford the product as a light yellow solid (46.9 mg, 83% yield). m.p. = 48 – 50 °C. Eluant: ethyl acetate/petroleum ether (1:5, R_f = 0.30).
^{1}H NMR (400 MHz, $CDCl_3$) δ 7.47 – 7.43 (m, 1H), 7.41 (dd, *J* = 7.2, 1.7 Hz, 1H), 7.35 – 7.30 (m, 1H), 7.30 – 7.21 (m, 4H), 7.12 – 7.07 (m, 1H), 6.32 (br, 1H), 2.66 (q, *J* = 7.6 Hz, 2H), 1.15 (t, *J* = 7.6 Hz, 3H). ^{13}C NMR (101 MHz, $CDCl_3$) δ 199.4, 156.3, 143.2, 139.3, 138.3, 130.6, 129.8, 129.5, 128.5, 125.3, 123.4, 121.0, 116.4, 26.5, 16.0.
IR: 3328, 3063, 2967, 2932, 1646, 1594, 1582, 1479, 1447, 1291, 1224, 847, 750 cm^{-1}.
HRMS (ESI) m/z: [M + Na]$^+$ Calcd for $C_{15}H_{14}O_2Na$ 249.0886; Found 249.0882.

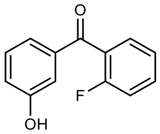

(2-fluorophenyl)(3-hydroxyphenyl)methanone (2d). Prepared according to general procedure D and purified by flash column chromatography to afford the product as a light yellow solid (38.3 mg, 71% yield). m.p. = 110 – 112 °C. Eluant: ethyl acetate/petroleum ether (1:4, R_f = 0.30).
^{1}H NMR (400 MHz, $CDCl_3$) δ 7.57 – 7.49 (m, 2H), 7.43 – 7.39 (m, 1H), 7.36 – 7.30 (m, 2H), 7.29 – 7.23 (m, 1H), 7.19 – 7.09 (m, 2H), 5.83 (br, 1H). ^{13}C NMR (101 MHz, $CDCl_3$) δ 193.8, 160.3 (C-F, $^1J_{C-F}$ = 253.1 Hz), 156.1, 138.9, 133.3 (C-F, $^3J_{C-F}$ = 8.4 Hz), 130.9 (C-F, $^4J_{C-F}$ = 2.8 Hz), 129.9, 127.0 (C-F, $^2J_{C-F}$ = 14.5 Hz), 124.4 (C-F, $^3J_{C-F}$ = 3.7 Hz), 123.0, 121.1, 116.5 (C-F, $^2J_{C-F}$ = 21.6 Hz), 116.1. ^{19}F NMR (376 MHz, $CDCl_3$) δ -111.2 (m).
IR: 3354, 2925, 2853, 1651, 1610, 1597, 1584, 1482, 1451, 1301, 1222, 754 cm^{-1}.
HRMS (ESI) m/z: [M + Na]$^+$ Calcd for $C_{13}H_9O_2FNa$ 239.0479; Found 239.0471.

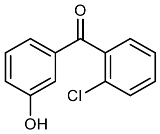

(2-chlorophenyl)(3-hydroxyphenyl)methanone (2e). Prepared according to general procedure D and purified by flash column chromatography to afford the product as a light yellow solid (38.9 mg, 67% yield). m.p. = 112 – 114 °C. Eluant: ethyl acetate/petroleum ether (1:5, R_f = 0.30).

^{1}H NMR (400 MHz, $CDCl_3$) δ 7.49 – 7.41 (m, 2H), 7.40 – 7.35 (m, 3H), 7.34 – 7.28 (m, 2H), 7.13 – 7.08 (m, 1H), 5.44 (br, 1H). ^{13}C NMR (101 MHz, $CDCl_3$) δ 195.5, 156.2, 138.5, 138.0, 131.5, 131.4, 130.3, 130.1, 129.3, 126.8, 123.3, 121.3, 116.2.
IR: 3352, 2924, 2853, 1655, 1586, 1449, 1434, 1294, 1226, 1058, 767, 748 cm^{-1}.
HRMS (ESI) m/z: $[M + Na]^+$ Calcd for $C_{13}H_9O_2ClNa$ 255.0183; Found 255.0173.

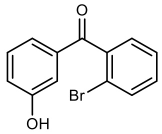

(2-bromophenyl)(3-hydroxyphenyl)methanone (2f). Prepared according to general procedure D and purified by flash column chromatography to afford the product as a light yellow solid (49.5 mg, 72% yield). m.p. = 90 – 92 °C. Eluant: ethyl acetate/petroleum ether (1:5, R_f = 0.30).
^{1}H NMR (400 MHz, $CDCl_3$) δ 7.64 (d, *J* = 7.7 Hz, 1H), 7.44 – 7.38 (m, 2H), 7.38 – 7.27 (m, 4H), 7.13 – 7.09 (m, 1H), 5.75 (br, 1H). ^{13}C NMR (101 MHz, $CDCl_3$) δ 196.3, 156.3, 140.5, 137.6, 133.4, 131.4, 130.1, 129.1, 127.3, 123.5, 121.5, 119.7, 116.3.
IR: 3357, 2926, 1654, 1585, 1449, 1294, 1225, 1048, 1026, 971, 746 cm^{-1}.
HRMS (ESI) m/z: $[M + Na]^+$ Calcd for $C_{13}H_9O_2BrNa$ 298.9678; Found 298.9687.

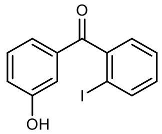

(3-hydroxyphenyl)(2-iodophenyl)methanone (2g). Prepared according to general procedure D and purified by flash column chromatography to afford the product as a light yellow solid (58.9 mg, 73% yield). m.p. = 92 – 94 °C. Eluant: ethyl acetate/petroleum ether (1:8, R_f = 0.3).
^{1}H NMR (400 MHz, Acetone-d_6) δ 8.81 (br, 1H), 8.00 (dd, *J* = 7.9, 1.1 Hz, 1H), 7.61 – 7.55 (m, 1H), 7.41 – 7.35 (m, 2H), 7.34 – 7.28 (m, 1H), 7.27 – 7.24 (m, 1H), 7.23 – 7.18 (m, 1H), 7.18 – 7.13 (m, 1H). ^{13}C NMR (101 MHz, Acetone-d_6) δ ^{13}C NMR (101 MHz, Acetone-d_6) δ 197.2, 158.6, 145.9, 140.3, 138.0, 132.0, 130.8, 129.0, 128.9, 122.5, 121.8, 117.0, 92.4.
IR: 3320, 2923, 2852, 1650, 1580, 1447, 1428, 1289, 1215, 1135, 1016, 970, 841, 742 cm^{-1}.
HRMS (ESI) m/z: $[M + Na]^+$ Calcd for $C_{13}H_9O_2INa$ 346.9539; Found 346.9549.

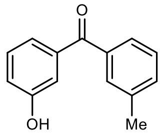

(3-hydroxyphenyl)(*m*-tolyl)methanone (2h). Prepared according to general procedure D and purified by flash column chromatography to afford the product as a light yellow solid (41.3 mg, 78% yield). m.p. = 65 – 67 °C. Eluant: ethyl acetate/petroleum ether (1:5, R_f = 0.30).
^{1}H NMR (400 MHz, $CDCl_3$) δ 7.64 – 7.61 (m, 1H), 7.60 – 7.56 (m, 1H), 7.44 – 7.29 (m, 5H), 7.11 – 7.07 (m, 1H), 5.68 (br, 1H), 2.42 (s, 3H). ^{13}C NMR (101 MHz, $CDCl_3$) δ 197.4, 156.1, 139.1, 138.3, 137.5, 133.6, 130.7, 129.6, 128.3, 127.6, 123.0, 120.0, 116.7, 21.5.
IR: 3349, 2923, 2854, 1642, 1594, 1580, 1479, 1447, 1295, 1230, 1193, 784, 743 cm^{-1}.
HRMS (ESI) m/z: $[M + Na]^+$ Calcd for $C_{14}H_{12}O_2Na$ 235.0730; Found 235.0736.

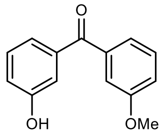

(3-hydroxyphenyl)(3-methoxyphenyl)methanone (2i). Prepared according to general procedure D and purified by flash column chromatography to afford the product as a yellow solid (41.0 mg, 72% yield). m.p. = 96 – 98 °C. Eluant: ethyl acetate/petroleum ether (1:4, R_f = 0.30).

^{1}H NMR (400 MHz, $CDCl_3$) δ 7.39 – 7.37 (m, 1H), 7.37 – 7.29 (m, 5H), 7.15 – 7.08 (m, 2H), 6.54 (br, 1H), 3.84 (s, 3H). ^{13}C NMR (101 MHz, $CDCl_3$) δ 197.4, 159.6, 156.3, 138.8, 138.7, 129.6, 129.4, 123.2, 122.8, 120.3, 119.3, 116.8, 114.6, 55.6.

IR: 3313, 2923, 2837, 1638, 1577, 1482, 1447, 1429, 1286, 1257, 1208, 1040, 994, 782, 747, 701 cm^{-1}.

HRMS (ESI) m/z: $[M + Na]^+$ Calcd for $C_{14}H_{12}O_3Na$ 251.0679; Found 251.0690.

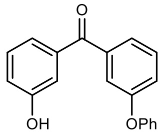

(3-hydroxyphenyl)(3-phenoxyphenyl)methanone (2j). Prepared according to general procedure D and purified by flash column chromatography to afford the product as a light yellow solid (54.4 mg, 75% yield). m.p. = 94 – 96 °C. Eluant: ethyl acetate/petroleum ether (1:4, R_f = 0.30).

^{1}H NMR (400 MHz, Acetone-d_6) δ 8.76 (br, 1H), 7.60 – 7.48 (m, 2H), 7.45 – 7.39 (m, 2H), 7.39 – 7.33 (m, 2H), 7.32 – 7.22 (m, 3H), 7.21 – 7.15 (m, 1H), 7.14 – 7.07 (m, 3H). ^{13}C NMR (101 MHz, Acetone-d_6) δ 195.6, 158.4, 158.3, 157.5, 140.4, 139.6, 131.0 (2C), 130.9, 130.4, 125.4, 124.8, 123.2, 122.0, 120.6, 120.1 (2C), 119.8, 117.0.

IR: 3329, 3064, 2926, 1642, 1577, 1487, 1436, 1288, 1250, 1216, 1161, 747 cm^{-1}.

HRMS (ESI) m/z: $[M + Na]^+$ Calcd for $C_{19}H_{14}O_3Na$ 313.0835; Found 313.0827.

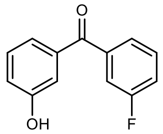

(3-fluorophenyl)(3-hydroxyphenyl)methanone (2k). Prepared according to general procedure D and purified by flash column chromatography to afford the product as a light yellow solid (44.3 mg, 82% yield). m.p. = 110 – 112 °C. Eluant: ethyl acetate/petroleum ether (1:4, R_f = 0.30).

^{1}H NMR (400 MHz, $CDCl_3$) δ 7.61 – 7.56 (m, 1H), 7.53 – 7.42 (m, 2H), 7.40 – 7.27 (m, 4H), 7.13 – 7.09 (m, 1H), 5.68 (br, 1H). ^{13}C NMR (101 MHz, $CDCl_3$) δ 195.7 (C-F, $^{4}J_{C\text{-}F}$ = 2.0 Hz), 162.6 (C-F, $^{1}J_{C\text{-}F}$ = 248.1 Hz), 156.2, 139.5 (C-F, $^{3}J_{C\text{-}F}$ = 6.4 Hz), 138.4, 130.2 (C-F, $^{3}J_{C\text{-}F}$ = 7.6 Hz), 129.8, 126.0 (C-F, $^{4}J_{C\text{-}F}$ = 3.0 Hz), 123.0, 120.5, 119.8 (C-F, $^{2}J_{C\text{-}F}$ = 21.4 Hz), 117.0 (C-F, $^{2}J_{C\text{-}F}$ = 22.4 Hz), 116.6. ^{19}F NMR (376 MHz, $CDCl_3$) δ -130.1 (dd, J = 12.0, 7.9 Hz).

IR: 3368, 2925, 1649, 1583, 1480, 1445, 1303, 1254, 1192, 749 cm^{-1}.

HRMS (ESI) m/z: $[M + Na]^+$ Calcd for $C_{13}H_9O_2FNa$ 239.0479; Found 239.0484.

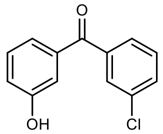

(3-chlorophenyl)(3-hydroxyphenyl)methanone (2l). Prepared according to general procedure D and purified by flash column chromatography to afford the product as a yellow solid (51.0 mg, 88% yield). m.p. = 97 – 99 °C. Eluant: ethyl acetate/petroleum ether (1:5, R_f = 0.30).
^{1}H NMR (400 MHz, Acetone-d_6) δ 8.87 (br, 1H), 7.78 – 7.75 (m, 1H), 7.73 – 7.67 (m, 2H), 7.63 – 7.56 (m, 1H), 7.43 – 7.37 (m, 1H), 7.29 – 7.22 (m, 2H), 7.18 – 7.13 (m, 1H). ^{13}C NMR (101 MHz, Acetone-d_6) δ 195.0, 158.4, 140.6, 139.2, 134.8, 132.9, 131.0, 130.5, 130.0, 129.0, 122.1, 120.9, 117.0.
IR: 3330, 3065, 2926, 1644, 1581, 1567, 1473, 1446, 1418, 1287, 1227, 1165, 1077, 741 cm^{-1}.
HRMS (ESI) m/z: [M + Na]$^+$ Calcd for $C_{13}H_9O_2ClNa$ 255.0183; Found 255.0191.

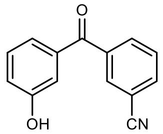

3-(3-hydroxybenzoyl)benzonitrile (2m). Prepared according to general procedure D and purified by flash column chromatography to afford the product as a light yellow solid (42.4 mg, 76% yield). m.p. = 109 – 111 °C. Eluant: ethyl acetate/petroleum ether (1:4, R_f = 0.30).
^{1}H NMR (400 MHz, Acetone-d_6) δ 8.88 (br, 1H), 8.15 – 8.11 (m, 1H), 8.10 – 8.03 (m, 2H), 7.84 – 7.76 (m, 1H), 7.45 – 7.38 (m, 1H), 7.31 – 7.22 (m, 2H), 7.20 – 7.14 (m, 1H). ^{13}C NMR (101 MHz, Acetone-d_6) δ 194.6, 158.5, 139.8, 138.8, 136.2, 134.6, 133.8, 130.7, 130.6, 122.2, 121.1, 118.7, 117.0, 113.5.
IR: 3366, 3069, 2925, 1655, 1595, 1580, 1476, 1448, 1424, 1294, 1224, 1182, 1018, 745 cm^{-1}.
HRMS (ESI) m/z: [M + Na]$^+$ Calcd for $C_{14}H_9NO_2Na$ 246.0525; Found 246.0519.

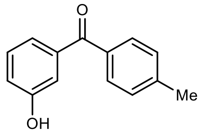

(3-hydroxyphenyl)(*p*-tolyl)methanone (2n). Prepared according to general procedure D and purified by flash column chromatography to afford the product as a yellow solid (33.4 mg, 63% yield). m.p. = 110 – 113 °C. Eluant: ethyl acetate/petroleum ether (1:5, R_f = 0.30).
^{1}H NMR (400 MHz, $CDCl_3$) δ 7.72 (d, J = 7.8 Hz, 2H), 7.41 – 7.34 (m, 1H), 7.33 – 7.25 (m, 4H), 7.12 – 7.05 (m, 1H), 6.05 (br, 1H), 2.44 (s, 3H). ^{13}C NMR (101 MHz, $CDCl_3$) δ 196.9, 156.1, 143.7, 139.4, 134.8, 130.6 (2C), 129.6, 129.2 (2C), 122.8, 119.8, 116.6, 21.8.
IR: 3320, 2923, 2853, 1638, 1594, 1581, 1446, 1313, 1288, 1225, 1181, 749 cm^{-1}.
HRMS (ESI) m/z: [M + Na]$^+$ Calcd for $C_{14}H_{12}O_2Na$ 235.0730; Found 235.0725.

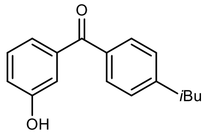

(3-hydroxyphenyl)(4-isobutylphenyl)methanone (2o). Prepared according to general procedure D and purified by flash column chromatography to afford the product as a yellow solid (38.7 mg, 61% yield). m.p. = 70 – 72 °C. Eluant: ethyl acetate/petroleum ether (1:5, R_f = 0.30).

^1H NMR (400 MHz, $CDCl_3$) δ 7.77 – 7.71 (m, 2H), 7.38 – 7.29 (m, 3H), 7.26 – 7.20 (m, 2H), 7.12 – 7.06 (m, 1H), 5.79 (br, 1H), 2.56 (d, J = 7.2 Hz, 2H), 1.99 – 1.86 (m, 1H), 0.93 (d, J = 6.6 Hz, 6H). ^{13}C NMR (101 MHz, $CDCl_3$) δ 197.2, 156.2, 147.5, 139.3, 135.0, 130.5 (2C), 129.6, 129.2 (2C), 122.8, 119.9, 116.7, 45.6, 30.3, 22.5 (2C).
IR: 3349, 2955, 2925, 1640, 1595, 1582, 1447, 1313, 1288, 1237, 1182, 753 cm^{-1}.
HRMS (ESI) m/z: $[M + Na]^+$ Calcd for $C_{17}H_{18}O_2Na$ 277.1199; Found 277.1205.

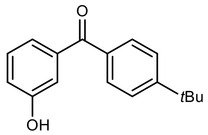

(4-(*tert*-butyl)phenyl)(3-hydroxyphenyl)methanone (2p). Prepared according to general procedure D and purified by flash column chromatography to afford the product as a yellow solid (36.8 mg, 58% yield). m.p. = 98 – 100 °C. Eluant: ethyl acetate/petroleum ether (1:5, R_f = 0.30).
^1H NMR (400 MHz, $CDCl_3$) δ 7.82 – 7.74 (m, 2H), 7.53 – 7.46 (m, 2H), 7.39 – 7.30 (m, 3H), 7.12 – 7.06 (m, 1H), 5.70 (br, 1H), 1.36 (s, 9H). ^{13}C NMR (101 MHz, $CDCl_3$) δ 197.0, 156.7, 156.2, 139.3, 134.7, 130.4 (2C), 129.6, 125.4 (2C), 122.9, 119.9, 116.7, 35.3, 31.3 (3C).
IR: 3322, 2958, 2924, 1638, 1594, 1581, 1446, 1316, 1287, 1105, 973, 766, 716 cm^{-1}.
HRMS (ESI) m/z: $[M + H]^+$ Calcd for $C_{17}H_{19}O_2$ 255.1380; Found 255.1375.

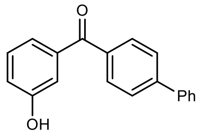

[1,1'-biphenyl]-4-yl(3-hydroxyphenyl)methanone (2q). Prepared according to general procedure D and purified by flash column chromatography to afford the product as a yellow solid (34.9 mg, 51% yield). m.p. = 156 – 158 °C. Eluant: ethyl acetate/petroleum ether (1:4, R_f = 0.30).
^1H NMR (400 MHz, Acetone-d_6) δ 8.77 (br, 1H), 7.93 – 7.82 (m, 4H), 7.81 – 7.74 (m, 2H), 7.56 – 7.49 (m, 2H), 7.48 – 7.36 (m, 2H), 7.33 – 7.24 (m, 2H), 7.17 – 7.12 (m, 1H). ^{13}C NMR (101 MHz, Acetone-d_6) δ 196.0, 158.3, 145.7, 140.6, 140.1, 137.4, 131.3 (2C), 130.4, 129.9 (2C), 129.1, 128.0 (2C), 127.6 (2C), 122.0, 120.4, 117.0.
IR: 3343, 2954, 2924, 1639, 1595, 1580, 1447, 1403, 1315, 1294, 1236, 746 cm^{-1}.
HRMS (ESI) m/z: $[M + Na]^+$ Calcd for $C_{19}H_{14}O_2Na$ 297.0886; Found 297.0874.

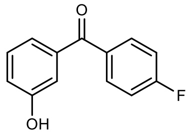

(4-fluorophenyl)(3-hydroxyphenyl)methanone (2r). Prepared according to general procedure D and purified by flash column chromatography to afford the product as a light yellow solid (41.0 mg, 76% yield). m.p. = 102 –104 °C. Eluant: ethyl acetate/petroleum ether (1:5, R_f = 0.3).
^1H NMR (400 MHz, $CDCl_3$) δ 7.90 – 7.81 (m, 2H), 7.39 – 7.31 (m, 2H), 7.30 – 7.26 (m, 1H), 7.20 – 7.13 (m, 2H), 7.12 – 7.07 (m, 1H), 5.88 (br, 1H). ^{13}C NMR (101 MHz, $CDCl_3$) δ 196.0, 165.7 (C-F, $^1J_{C-F}$ = 254.9 Hz), 156.3, 138.8, 133.6 (C-F, $^4J_{C-F}$ = 3.0 Hz), 133.0 (C-F, $^3J_{C-F}$ = 9.2 Hz, 2C), 129.7, 122.7, 120.3, 116.6, 115.7 (C-F, $^2J_{C-F}$ = 21.8 Hz, 2C). ^{19}F NMR (376 MHz, $CDCl_3$) δ -105.5 (m).
IR: 3350, 2954, 2924, 1643, 1595, 1504, 1446, 1409, 1306, 1286, 1233, 1156, 872, 758 cm^{-1}.
HRMS (ESI) m/z: $[M + Na]^+$ Calcd for $C_{13}H_9O_2FNa$ 239.0479; Found 239.0485.

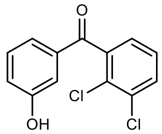

(2,3-dichlorophenyl)(3-hydroxyphenyl)methanone (2s). Prepared according to general procedure D and purified by flash column chromatography to afford the product as a yellow solid (57.0 mg, 86% yield). m.p. = 154 – 156 °C. Eluant: ethyl acetate/petroleum ether (1:4, R_f = 0.30).

^{1}H NMR (400 MHz, Acetone-d_6) δ 8.84 (br, 1H), 7.77 (dd, *J* = 8.0, 1.5 Hz, 1H), 7.57 – 7.51 (m, 1H), 7.43 (dd, *J* = 7.6, 1.6 Hz, 1H), 7.41 – 7.35 (m, 1H), 7.29 – 7.26 (m, 1H), 7.25 – 7.21 (m, 1H), 7.19 – 7.15 (m, 1H). ^{13}C NMR (101 MHz, Acetone-d_6) δ 194.0, 158.8, 142.2, 138.3, 133.9, 132.5, 131.0, 129.33, 129.30, 128.0, 122.24, 122.22, 116.6.

IR: 3364, 2926, 2853, 1655, 1596, 1583, 1448, 1410, 1291, 1227, 1194, 1148, 760, 745 cm^{-1}.

HRMS (ESI) m/z: [M + Na]$^+$ Calcd for $C_{13}H_8O_2Cl_2Na$ 288.9794; Found 288.9785.

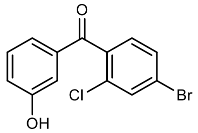

(4-bromo-2-chlorophenyl)(3-hydroxyphenyl)methanone (2t). Prepared according to general procedure D and purified by flash column chromatography to afford the product as a yellow solid (58.7 mg, 76% yield). m.p. = 129 – 131 °C. Eluant: ethyl acetate/petroleum ether (1:4, R_f = 0.30).

^{1}H NMR (400 MHz, Acetone-d_6) δ 8.83 (br, 1H), 7.81 (d, *J* = 1.8 Hz, 1H), 7.72 (dd, *J* = 8.2, 1.8 Hz, 1H), 7.44 (d, *J* = 8.1 Hz, 1H), 7.41 – 7.35 (m, 1H), 7.28 – 7.25 (m, 1H), 7.25 – 7.21 (m, 1H),7.19 – 7.15 (m, 1H). ^{13}C NMR (101 MHz, Acetone-d_6) δ 194.2, 158.7, 139.0, 138.4, 133.2, 132.5, 131.3, 131.2, 131.0, 124.6, 122.2, 122.1, 116.6.

IR: 3356, 2925, 2852, 1655, 1597, 1579, 1449, 1369, 1291, 1225, 1135, 1083, 851, 756 cm^{-1}.

HRMS (ESI) m/z: [M + Na]$^+$ Calcd for $C_{13}H_8O_2ClBrNa$ 332.9288; Found 332.9276.

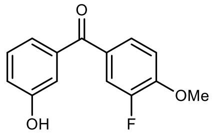

(3-fluoro-4-methoxyphenyl)(3-hydroxyphenyl)methanone (2u). Prepared according to general procedure D and purified by flash column chromatography to afford the product as a yellow solid (44.3 mg, 72% yield). m.p. = 178 – 180 °C. Eluant: ethyl acetate/petroleum ether (1:3, R_f = 0.30).

^{1}H NMR (400 MHz, Acetone-d_6) δ 8.75 (br, 1H), 7.66 – 7.56 (m, 2H), 7.42 – 7.34 (m, 1H), 7.33 – 7.25 (m, 1H), 7.24 – 7.16 (m, 2H), 7.15 – 7.07 (m, 1H), 4.01 (s, 3H). ^{13}C NMR (101 MHz, Acetone-d_6) δ 194.2, 158.3, 152.5 (C-F, $^1J_{C-F}$ = 247.6 Hz), 152.4 (C-F, $^2J_{C-F}$ = 10.7 Hz), 140.0, 131.2 (C-F, $^3J_{C-F}$ = 5.1 Hz), 130.4, 128.6 (C-F, $^3J_{C-F}$ = 3.3 Hz), 121.7, 120.1, 117.7 (C-F, $^2J_{C-F}$ = 19.2 Hz), 116.8, 113.6 (C-F, $^4J_{C-F}$ = 1.7 Hz), 56.8. ^{19}F NMR (376 MHz, Acetone-d_6) δ -112.9 (m).

IR: 3353, 2923, 2852, 1657, 1608, 1580, 1517, 1443, 1282, 1107, 1023, 754 cm^{-1}.

HRMS (ESI) m/z: [M + Na]$^+$ Calcd for $C_{14}H_{11}O_3FNa$ 269.0584; Found 269.0575.

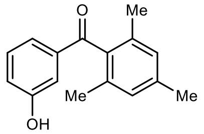

(3-hydroxyphenyl)(mesityl)methanone (2v). Prepared according to general procedure D and purified by flash column chromatography to afford the product as a yellow solid (33.6 mg, 56% yield). m.p. = 122 – 124 °C. Eluant: ethyl acetate/petroleum ether (1:5, R_f = 0.30).
^{1}H NMR (400 MHz, $CDCl_3$) δ 7.40 – 7.35 (m, 1H), 7.33 – 7.27 (m, 2H), 7.10 – 7.04 (m, 1H), 6.89 (s, 2H), 5.67 (br, 1H), 2.32 (s, 3H), 2.08 (s, 6H). ^{13}C NMR (101 MHz, $CDCl_3$) δ 201.0, 156.4, 139.0, 138.7, 136.9, 134.4 (2C), 130.3, 128.5 (2C), 122.5, 121.1, 115.5, 21.3, 19.5 (2C).
IR: 3321, 2953, 2923, 1649, 1610, 1595, 1583, 1478, 1448, 1286, 1170, 851, 833, 759 cm^{-1}.
HRMS (ESI) m/z: $[M + Na]^+$ Calcd for $C_{16}H_{16}O_2Na$ 263.1043; Found 263.1033.

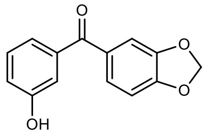

benzo[*d*][1,3]dioxol-5-yl(3-hydroxyphenyl)methanone (2w). Prepared according to general procedure D and purified by flash column chromatography to afford the product as a yellow solid (47.8 mg, 79% yield). m.p. = 144 – 146 °C. Eluant: ethyl acetate/petroleum ether (1:3, R_f = 0.30).
^{1}H NMR (400 MHz, Acetone-d_6) δ 8.70 (br, 1H), 7.40 – 7.33 (m, 2H), 7.29 (d, J = 1.7 Hz, 1H), 7.22 – 7.15 (m, 2H), 7.12 – 7.06 (m, 1H), 6.98 (d, J = 8.1 Hz, 1H), 6.15 (s, 2H). ^{13}C NMR (101 MHz, Acetone-d_6) δ 194.7, 158.2, 152.5, 149.0, 140.5, 132.8, 130.2, 127.3, 121.6, 119.9, 116.8, 109.9, 108.4, 103.1.
IR: 3300, 2923, 2853, 1637, 1580, 1503, 1485, 1440, 1354, 1293, 1255, 1037, 806, 754 cm^{-1}.
HRMS (ESI) m/z: $[M + Na]^+$ Calcd for $C_{14}H_{10}O_4Na$ 265.0471; Found 265.0466.

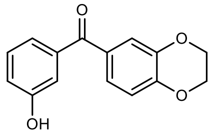

(2,3-dihydrobenzo[*b*][1,4]dioxin-6-yl)(3-hydroxyphenyl)methanone (2x). Prepared according to general procedure D and purified by flash column chromatography to afford the product as a yellow solid (49.3 mg, 77% yield). m.p. = 145 – 147 °C. Eluant: ethyl acetate/petroleum ether (1:3, R_f = 0.30).
^{1}H NMR (400 MHz, Acetone-d_6) δ 8.72 (br, 1H), 7.42 – 7.28 (m, 3H), 7.25 – 7.15 (m, 2H), 7.13 – 7.06 (m, 1H), 6.96 (d, J = 8.1 Hz, 1H), 4.41 – 4.36 (m, 2H), 4.35 – 4.31 (m, 2H). ^{13}C NMR (101 MHz, Acetone-d_6) δ 194.8, 158.1, 148.7, 144.2, 140.4, 131.7, 130.2, 124.8, 121.6, 119.83, 119.81, 117.7, 116.8, 65.6, 65.0.
IR: 3337, 2925, 2853, 1637, 1577, 1504, 1447, 1429, 1287, 1259, 1065, 886, 759 cm^{-1}.
HRMS (ESI) m/z: $[M + Na]^+$ Calcd for $C_{15}H_{12}O_4Na$ 279.0628; Found 279.0620.

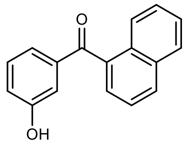

(3-hydroxyphenyl)(naphthalen-1-yl)methanone (2y). Prepared according to general procedure D and purified by flash column chromatography to afford the product as a yellow oil (47.7 mg, 77% yield). Eluant: ethyl acetate/petroleum ether (1:5, R_f = 0.30).

^{1}H NMR (400 MHz, $CDCl_3$) δ 8.09 – 8.03 (m, 1H), 8.00 – 7.94 (m, 1H), 7.91 – 7.85 (m, 1H), 7.58 – 7.41 (m, 5H), 7.31 – 7.20 (m, 2H), 7.10 – 7.05 (m, 1H), 6.58 (br, 1H). ^{13}C NMR (101 MHz, $CDCl_3$) δ 198.8, 156.3, 139.6, 136.1, 133.8, 131.7, 131.0, 129.8, 128.6, 128.2, 127.5, 126.6, 125.7, 124.4, 123.5, 121.1, 116.7.
IR: 3351, 3059, 2925, 1643, 1582, 1508, 1481, 1447, 1289, 1233, 1191, 793, 780, 748 cm^{-1}.
HRMS (ESI) m/z: $[M + Na]^+$ Calcd for $C_{17}H_{12}O_2Na$ 271.0730; Found 271.0738.

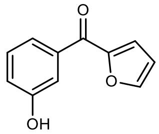

furan-2-yl(3-hydroxyphenyl)methanone (2z). Prepared according to general procedure D and purified by flash column chromatography to afford the product as a yellow solid (33.8 mg, 72% yield). m.p. = 90 – 92 °C. Eluant: ethyl acetate/petroleum ether (1:5, R_f = 0.30).
^{1}H NMR (400 MHz, Acetone-d_6) δ 8.81 (br, 1H), 7.95 (d, J = 1.7 Hz, 1H), 7.49 – 7.45 (m, 1H), 7.44 – 7.42 (m, 1H), 7.42 – 7.36 (m, 1H), 7.32 (d, J = 3.6 Hz, 1H), 7.16 – 7.10 (m, 1H), 6.74 (dd, J = 3.6, 1.7 Hz, 1H). ^{13}C NMR (101 MHz, Acetone-d_6) δ 182.2, 158.3, 153.2, 148.4, 139.7, 130.5, 121.3, 121.1, 120.5, 116.4, 113.1.
IR: 3325, 2933, 2851, 1629, 1593, 1581, 1560, 1461, 1392, 1316, 1030, 819, 754 cm^{-1}.
HRMS (ESI) m/z: $[M + Na]^+$ Calcd for $C_{11}H_8O_3Na$ 211.0366; Found 211.0356.

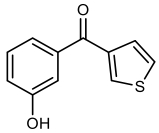

(3-hydroxyphenyl)(thiophen-3-yl)methanone (2aa). Prepared according to general procedure D and purified by flash column chromatography to afford the product as as a yellow solid (41.3 mg, 81% yield). m.p. = 158 – 160 °C. Eluant: ethyl acetate/petroleum ether (1:5, R_f = 0.30).
^{1}H NMR (400 MHz, Acetone-d_6) δ 8.73 (br, 1H), 8.13 (dd, J = 2.9, 1.3 Hz, 1H), 7.62 (dd, J = 5.1, 2.9 Hz, 1H), 7.56 (dd, J = 5.1, 1.3 Hz, 1H), 7.42 – 7.35 (m, 1H), 7.35 – 7.29 (m, 2H), 7.16 – 7.09 (m, 1H). ^{13}C NMR (101 MHz, Acetone-d_6) δ 189.7, 158.3, 142.1, 141.0, 134.8, 130.5, 129.1, 127.5, 121.4, 120.2, 116.5.
IR: 3341, 2924, 2852, 1633, 1581, 1509, 1446, 1412, 1390, 1286, 1243, 745 cm^{-1}.
HRMS (ESI) m/z: $[M + Na]^+$ Calcd for $C_{11}H_8O_2SNa$ 227.0137; Found 227.0129.

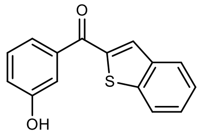

benzo[*b*]thiophen-2-yl(3-hydroxyphenyl)methanone (2ab). Prepared according to general procedure D and purified by flash column chromatography to afford the product as a yellow solid (27.3 mg, 43% yield). m.p. = 169 – 171 °C. Eluant: ethyl acetate/petroleum ether (1:4, R_f = 0.30).
^{1}H NMR (400 MHz, Acetone-d_6) δ 8.83 (br, 1H), 8.12 – 8.03 (m, 3H), 7.60 – 7.54 (m, 1H), 7.53 – 7.47 (m, 1H), 7.46 – 7.42 (m, 2H), 7.41 – 7.37 (m, 1H), 7.22 – 7.13 (m, 1H). ^{13}C NMR (101 MHz, Acetone-d_6) δ 189.6, 158.5, 143.9, 143.2, 140.3, 140.0, 133.3, 130.7, 128.5, 127.3, 126.1, 123.7, 121.3, 120.5, 116.4.
IR: 3350, 2923, 2852, 1626, 1591, 1580, 1510, 1445, 1427, 1298, 1181, 783, 754, 722 cm^{-1}.
HRMS (ESI) m/z: $[M + Na]^+$ Calcd for $C_{15}H_{10}O_2SNa$ 277.0294; Found 277.0303.

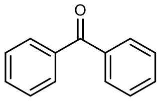

benzophenone (5). ^{1}H NMR (400 MHz, CDCl$_3$) δ 7.87 – 7.76 (m, 4H), 7.64 – 7.56 (m, 2H), 7.54 – 7.44 (m, 4H). ^{13}C NMR (101 MHz, CDCl$_3$) δ 196.9, 137.7 (2C), 132.6 (2C), 130.2 (4C), 128.4 (4C).
IR: 3060, 2927, 2853, 1657, 1598, 1577, 1447, 1317, 1276, 763, 698 cm^{-1}.
HRMS (ESI) m/z: [M + Na]$^+$ Calcd for $C_{13}H_{10}ONa$ 205.0624; Found 205.0615.

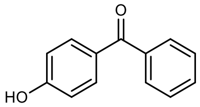

(4-hydroxyphenyl)(phenyl)methanone (6). ^{1}H NMR (400 MHz, CDCl$_3$) δ 8.23 (br, 1H), 7.81 – 7.72 (m, 4H), 7.61 – 7.53 (m, 1H), 7.51 – 7.43 (m, 2H), 7.00 – 6.93 (m, 2H). ^{13}C NMR (101 MHz, CDCl$_3$) δ 197.5, 161.4, 138.0, 133.4 (2C), 132.5, 130.0 (2C), 129.3, 128.4 (2C), 115.6 (2C).
IR: 3250, 2925, 2853, 1634, 1599, 1585, 1572, 1511, 1445, 1319, 1280, 1170, 743, 699 cm^{-1}.
HRMS (ESI) m/z: [M + Na]$^+$ Calcd for $C_{13}H_{10}O_2Na$ 221.0573; Found 221.0582.

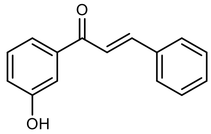

(*E*)-1-(3-hydroxyphenyl)-3-phenylprop-2-en-1-one (8a). Prepared according to general procedure D and purified by flash column chromatography to afford the product as a yellow solid (29.7 mg, 53% yield). m.p. = 116 – 118 °C. Eluant: ethyl acetate/petroleum ether (1:6, R_f = 0.30).
^{1}H NMR (400 MHz, CDCl$_3$) δ 7.82 (d, *J* = 15.7 Hz, 1H), 7.71 – 7.65 (m, 1H), 7.65 – 7.59 (m, 2H), 7.57 (d, *J* = 7.7 Hz, 1H), 7.51 (d, *J* = 15.7 Hz, 1H), 7.45 – 7.33 (m, 4H), 7.18 – 7.11 (m, 1H), 6.99 (br, 1H). ^{13}C NMR (101 MHz, CDCl$_3$) δ 191.2, 156.7, 145.7, 139.6, 134.8, 130.9, 130.1, 129.1 (2C), 128.7 (2C), 122.1, 121.1, 120.8, 115.4.
IR: 3321, 2924, 2853, 1653, 1574, 1494, 1449, 1336, 1304, 1290, 1184, 760 cm^{-1}.
HRMS (ESI) m/z: [M + Na]$^+$ Calcd for $C_{15}H_{12}O_2Na$ 247.0730; Found 247.0721.

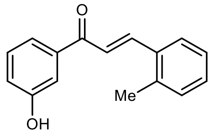

(*E*)-1-(3-hydroxyphenyl)-3-(*o*-tolyl)prop-2-en-1-one (8b). Prepared according to general procedure D and purified by flash column chromatography to afford the product as a yellow solid (47.6 mg, 80% yield). m.p. = 95 – 97 °C. Eluant: ethyl acetate/petroleum ether (1:6, R_f = 0.30).
^{1}H NMR (400 MHz, Acetone-*d*$_6$) δ 8.80 (br, 1H), 8.08 (d, *J* = 15.5 Hz, 1H), 7.94 – 7.89 (m, 1H), 7.73 – 7.63 (m, 2H), 7.59 – 7.56 (m, 1H), 7.43 – 7.37 (m, 1H), 7.36 – 7.25 (m, 3H), 7.16 – 7.10 (m, 1H), 2.48 (s, 3H). ^{13}C NMR (101 MHz, Acetone-*d*$_6$) δ 189.8, 158.7, 142.0, 140.6, 139.0, 134.7, 131.7, 131.1, 130.7, 127.5, 127.3, 123.9, 120.8, 120.7, 115.6, 19.8.
IR: 3285, 3057, 2925, 1649, 1568, 1481, 1445, 1320, 1283, 1180, 975, 757, 731, 698 cm^{-1}.
HRMS (ESI) m/z: [M + Na]$^+$ Calcd for $C_{16}H_{14}O_2Na$ 261.0886; Found 261.0889.

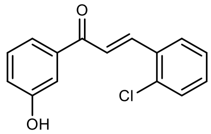

(*E*)-3-(2-chlorophenyl)-1-(3-hydroxyphenyl)prop-2-en-1-one (8c). Prepared according to general procedure D and purified by flash column chromatography to afford the product as a yellow solid (43.2 mg, 67% yield). m.p. = 111 – 113 °C. Eluant: ethyl acetate/petroleum ether (1:6, R_f = 0.30).

^{1}H NMR (400 MHz, Acetone-*d*$_6$) δ 8.78 (br, 1H), 8.17 (d, *J* = 15.6 Hz, 1H), 8.11 (dd, *J* = 7.3, 2.2 Hz, 1H), 7.84 (d, *J* = 15.6 Hz, 1H), 7.71 – 7.65 (m, 1H), 7.62 – 7.57 (m, 1H), 7.56 – 7.51 (m, 1H), 7.49 – 7.37 (m, 3H), 7.19 – 7.12 (m, 1H). ^{13}C NMR (101 MHz, Acetone-*d*$_6$) δ 189.6, 158.7, 140.3, 139.7, 135.7, 133.9, 132.4, 130.9, 130.7, 129.1, 128.4, 125.6, 121.1, 120.9, 115.7.

IR: 3314, 2924, 2852, 1652, 1577, 1468, 1443, 1315, 1277, 1183, 975, 778, 753 cm^{-1}.

HRMS (ESI) m/z: [M + Na]$^+$ Calcd for $C_{15}H_{11}O_2ClNa$ 281.0340; Found 281.0328.

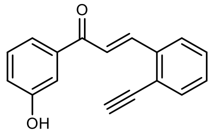

(*E*)-3-(2-ethynylphenyl)-1-(3-hydroxyphenyl)prop-2-en-1-one (8d). Prepared according to general procedure D and purified by flash column chromatography to afford the product as a brown solid (39.7 mg, 64% yield). m.p. = 117 – 119 °C. Eluant: ethyl acetate/petroleum ether (1:6, R_f = 0.30).

^{1}H NMR (400 MHz, $CDCl_3$) δ 8.27 (d, *J* = 15.8 Hz, 1H), 7.73 – 7.68 (m, 1H), 7.66 – 7.62 (m, 1H), 7.61 – 7.51 (m, 3H), 7.41 – 7.30 (m, 3H), 7.16 – 7.11 (m, 1H), 7.06 (br, 1H), 3.43 (s, 1H). ^{13}C NMR (101 MHz, $CDCl_3$) δ 191.4, 156.7, 143.2, 139.4, 136.8, 133.8, 130.2, 130.0, 129.2, 126.7, 124.1, 123.5, 121.2, 120.8, 115.5, 84.0, 81.3.

IR: 3288, 2925, 2853, 1655, 1578, 1475, 1447, 1327, 1277, 1185, 980, 758 cm^{-1}.

HRMS (ESI) m/z: [M + Na]$^+$ Calcd for $C_{17}H_{12}O_2Na$ 271.0730; Found 271.0726.

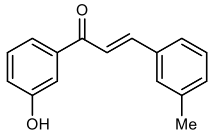

(*E*)-1-(3-hydroxyphenyl)-3-(*m*-tolyl)prop-2-en-1-one (8e). Prepared according to general procedure D and purified by flash column chromatography to afford the product as a yellow solid (42.2 mg, 71% yield). m.p. = 84 – 86 °C. Eluant: ethyl acetate/petroleum ether (1:6, R_f = 0.30).

^{1}H NMR (400 MHz, Acetone-*d*$_6$) δ 8.73 (br, 1H), 7.83 – 7.70 (m, 2H), 7.69 – 7.63 (m, 2H), 7.62 – 7.58 (m, 1H), 7.58 – 7.54 (m, 1H), 7.42 – 7.37 (m, 1H), 7.37 – 7.31 (m, 1H), 7.29 – 7.24 (m, 1H), 7.15 – 7.10 (m, 1H), 2.38 (s, 3H). ^{13}C NMR (101 MHz, Acetone-*d*$_6$) δ 189.8, 158.7, 144.9, 140.7, 139.4, 136.0, 132.1, 130.6, 129.9, 129.7, 126.8, 122.8, 120.8, 120.7, 115.6, 21.3.

IR: 3299, 2920, 2851, 1650, 1570, 1481, 1446, 1315, 1267, 1230, 1185, 980, 777, 684 cm^{-1}.

HRMS (ESI) m/z: [M + Na]$^+$ Calcd for $C_{16}H_{14}O_2Na$ 261.0886; Found 261.0891.

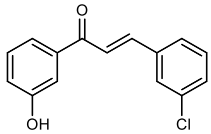

(*E*)-3-(3-chlorophenyl)-1-(3-hydroxyphenyl)prop-2-en-1-one (8f). Prepared according to general procedure D and purified by flash column chromatography to afford the product as a yellow solid (53.5 mg, 83% yield). m.p. = 112 – 114 °C. Eluant: ethyl acetate/petroleum ether (1:6, R_f = 0.30).

^{1}H NMR (400 MHz, Acetone-d_6) δ 8.82 (br, 1H), 7.96 – 7.88 (m, 2H), 7.80 – 7.71 (m, 2H), 7.71 – 7.66 (m, 1H), 7.62 – 7.57 (m, 1H), 7.52 – 7.44 (m, 2H), 7.43 – 7.36 (m, 1H), 7.17 – 7.11 (m, 1H). ^{13}C NMR (101 MHz, Acetone-d_6) δ 189.6, 158.6, 142.9, 140.3, 138.2, 135.3, 131.4, 130.9, 130.7, 128.7, 128.2, 124.5, 121.0, 120.8, 115.7.

IR: 3336, 2926, 1655, 1579, 1473, 1448, 1312, 1185, 979, 779 cm^{-1}.

HRMS (ESI) m/z: [M + Na]$^+$ Calcd for $C_{15}H_{11}O_2ClNa$ 281.0340; Found 281.0332.

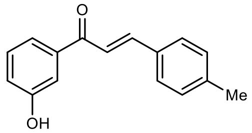

(*E*)-1-(3-hydroxyphenyl)-3-(*p*-tolyl)prop-2-en-1-one (8g). Prepared according to general procedure D and purified by flash column chromatography to afford the product as a yellow solid (39.9 mg, 67% yield). m.p. = 110 – 112 °C. Eluant: ethyl acetate/petroleum ether (1:6, R_f = 0.30).

^{1}H NMR (400 MHz, Acetone-d_6) δ 8.75 (br, 1H), 7.76 (s, 2H), 7.74 – 7.68 (m, 2H), 7.67 – 7.62 (m, 1H), 7.59 – 7.55 (m, 1H), 7.42 – 7.36 (m, 1H), 7.28 (d, *J* = 7.9 Hz, 2H), 7.15 – 7.10 (m, 1H), 2.37 (s, 3H). ^{13}C NMR (101 MHz, Acetone-d_6) δ 189.8, 158.6, 144.8, 141.7, 140.7, 133.3, 130.6, 130.5 (2C), 129.5 (2C), 122.0, 120.73, 120.66, 115.6, 21.4.

IR: 3316, 2923, 2853, 1650, 1577, 1511, 1447, 1332, 1263, 1180, 1030, 982, 812, 791, 737 cm^{-1}.

HRMS (ESI) m/z: [M + Na]$^+$ Calcd for $C_{16}H_{14}O_2Na$ 261.0886; Found 261.0894.

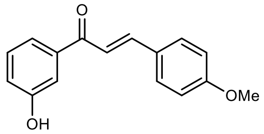

(*E*)-1-(3-hydroxyphenyl)-3-(4-methoxyphenyl)prop-2-en-1-one (8h). Prepared according to general procedure D and purified by flash column chromatography to afford the product as a yellow solid (46.4 mg, 73% yield). m.p. = 115 – 117 °C. Eluant: ethyl acetate/petroleum ether (1:4, R_f = 0.30).

^{1}H NMR (400 MHz, Acetone-d_6) δ 8.75 (br, 1H), 7.85 – 7.72 (m, 3H), 7.71 – 7.62 (m, 2H), 7.59 – 7.54 (m, 1H), 7.42 – 7.35 (m, 1H), 7.15 – 7.09 (m, 1H), 7.04 – 6.98 (m, 2H), 3.86 (s, 3H).

^{13}C NMR (101 MHz, Acetone-d_6) δ 189.7, 162.7, 158.6, 144.7, 140.9, 131.3 (2C), 130.6, 128.6, 120.60, 120.57, 120.5, 115.6, 115.2 (2C), 55.8.

IR: 3277, 2925, 2839, 1647, 1558, 1509, 1445, 1422, 1250, 1168, 1028, 826, 799, 739 cm^{-1}.

HRMS (ESI) m/z: [M + Na]$^+$ Calcd for $C_{16}H_{14}O_3Na$ 277.0835; Found 277.0827.

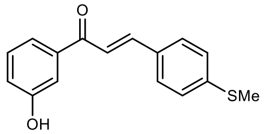

(*E*)-1-(3-hydroxyphenyl)-3-(4-(methylthio)phenyl)prop-2-en-1-one (8i). Prepared according to general procedure D and purified by flash column chromatography to afford the product as a yellow solid (48.6 mg, 72% yield). m.p. = 114 – 116 °C. Eluant: ethyl acetate/petroleum ether (1:5, R_f = 0.30).
^{1}H NMR (400 MHz, Acetone-d_6) δ 8.69 (br, 1H), 7.81 – 7.73 (m, 4H), 7.67 – 7.61 (m, 1H), 7.59 – 7.53 (m, 1H), 7.42 – 7.36 (m, 1H), 7.35 – 7.29 (m, 2H), 7.15 – 7.09 (m, 1H), 2.55 (s, 3H). ^{13}C NMR (101 MHz, Acetone-d_6) δ 189.7, 158.6, 144.3, 143.3, 140.7, 132.4, 130.6, 129.9 (2C), 126.6 (2C), 121.9, 120.73, 120.67, 115.6, 14.8.
IR: 3299, 2922, 2852, 1649, 1575, 1549, 1491, 1446, 1406, 1184, 1091, 815, 777 cm^{-1}.
HRMS (ESI) m/z: [M + Na]$^+$ Calcd for $C_{16}H_{14}O_2SNa$ 293.0607; Found 293.0600.

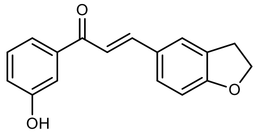

(*E*)-3-(2,3-dihydrobenzofuran-5-yl)-1-(3-hydroxyphenyl)prop-2-en-1-one (8j). Prepared according to general procedure D and purified by flash column chromatography to afford the product as a yellow solid (39.2 mg, 59% yield). m.p. = 179 – 181 °C. Eluant: ethyl acetate/petroleum ether (1:4, R_f = 0.3).
^{1}H NMR (400 MHz, Acetone-d_6) δ 8.74 (br, 1H), 7.79 – 7.71 (m, 2H), 7.68 – 7.60 (m, 2H), 7.59 – 7.53 (m, 2H), 7.41 – 7.34 (m, 1H), 7.13 – 7.07 (m, 1H), 6.81 (d, J = 8.3 Hz, 1H), 4.63 (t, J = 8.7 Hz, 2H), 3.27 (t, J = 8.7 Hz, 2H). ^{13}C NMR (101 MHz, Acetone-d_6) δ 189.7, 163.6, 158.6, 145.2, 141.0, 131.2, 130.5, 129.6, 128.8, 126.0, 120.54, 120.51, 119.8, 115.6, 110.2, 72.7, 29.7.
IR: 3349, 2920, 2850, 1658, 1579, 1491, 1445, 1264, 1242, 736 cm^{-1}.
HRMS (ESI) m/z: [M + Na]$^+$ Calcd for $C_{17}H_{14}O_3Na$ 289.0835; Found 289.0826.

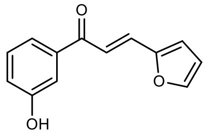

(*E*)-3-(furan-2-yl)-1-(3-hydroxyphenyl)prop-2-en-1-one (8k). Prepared according to general procedure D and purified by flash column chromatography to afford the product as a brown solid (33.7 mg, 63% yield). m.p. = 128 – 130 °C. Eluant: ethyl acetate/petroleum ether (1:6, R_f = 0.30).
^{1}H NMR (400 MHz, Acetone-d_6) δ 8.77 (br, 1H), 7.78 (d, J = 1.8 Hz, 1H), 7.62 – 7.55 (m, 2H), 7.54 – 7.46 (m, 2H), 7.43 – 7.37 (m, 1H), 7.15 – 7.09 (m, 1H), 7.00 (d, J = 3.4 Hz, 1H), 6.65 (dd, J = 3.5, 1.8 Hz, 1H). ^{13}C NMR (101 MHz, Acetone-d_6) δ 189.2, 158.7, 152.6, 146.4, 140.5, 131.0, 130.7, 120.8, 120.5, 120.0, 117.1, 115.5, 113.7.
IR: 3316, 2923, 2852, 1652, 1577, 1549, 1475, 1447, 1287, 1016, 750 cm^{-1}.
HRMS (ESI) m/z: [M + Na]$^+$ Calcd for $C_{13}H_{10}O_3Na$ 237.0522; Found 237.0517.

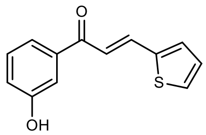

(*E*)-1-(3-hydroxyphenyl)-3-(thiophen-2-yl)prop-2-en-1-one (8l). Prepared according to general procedure D and purified by flash column chromatography to afford the product as a

yellow solid (36.8 mg, 64% yield). m.p. = 109 – 111 °C. Eluant: ethyl acetate/petroleum ether (1:6, R_f = 0.30).
^{1}H NMR (400 MHz, Acetone-d_6) δ 8.73 (br, 1H), 7.93 (d, *J* = 15.3 Hz, 1H), 7.66 (d, *J* = 5.1 Hz, 1H), 7.62 – 7.56 (m, 2H), 7.55 – 7.52 (m, 1H), 7.49 (d, *J* = 15.3 Hz, 1H), 7.43 – 7.35 (m, 1H), 7.18 (dd, *J* = 5.1, 3.6 Hz, 1H), 7.12 (dd, *J* = 8.0, 2.6 Hz, 1H). ^{13}C NMR (101 MHz, Acetone-d_6) δ 189.3, 158.6, 141.1, 140.5, 137.3, 133.2, 130.7, 130.2, 129.4, 121.5, 120.8, 120.6, 115.5.
IR: 3281, 2923, 2852, 1644, 1563, 1512, 1486, 1446, 1284, 1200, 966, 705 cm^{-1}.
HRMS (ESI) m/z: [M + H]$^+$ Calcd for $C_{13}H_{11}O_2S$ 231.0474; Found 231.0468.

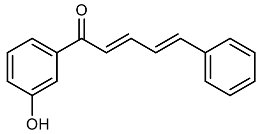

(2*E*,4*E*)-1-(3-hydroxyphenyl)-5-phenylpenta-2,4-dien-1-one (8m). Prepared according to general procedure D and purified by flash column chromatography to afford the product as a yellow solid (45.0 mg, 72% yield). m.p. = 115 – 117 °C. Eluant: ethyl acetate/petroleum ether (1:6, R_f = 0.30).
^{1}H NMR (400 MHz, Acetone-d_6) δ 8.73 (br, 1H), 7.66 – 7.59 (m, 2H), 7.59 – 7.51 (m, 2H), 7.51 – 7.47 (m, 1H), 7.45 – 7.33 (m, 4H), 7.32 – 7.22 (m, 2H), 7.21 – 7.14 (m, 1H), 7.14 – 7.08 (m, 1H). ^{13}C NMR (101 MHz, Acetone-d_6) δ 189.8, 158.7, 145.0, 142.3, 140.7, 137.4, 130.6, 129.9, 129.7 (2C), 128.2, 128.1 (2C), 126.5, 120.7, 120.5, 115.5.
IR: 3322, 2925, 1644, 1566, 1447, 1353, 1290, 1148, 997, 795, 752 cm^{-1}.
HRMS (ESI) m/z: [M + H]$^+$ Calcd for $C_{17}H_{15}O_2$ 251.1067; Found 251.1060.

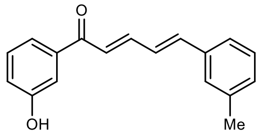

(2*E*,4*E*)-1-(3-hydroxyphenyl)-5-(*m*-tolyl)penta-2,4-dien-1-one (8n). Prepared according to general procedure D and purified by flash column chromatography to afford the product as a yellow solid (46.9 mg, 71% yield). m.p. = 65 – 67 °C. Eluant: ethyl acetate/petroleum ether (1:6, R_f = 0.30).
^{1}H NMR (400 MHz, Acetone-d_6) δ 8.73 (br, 1H), 7.62 – 7.51 (m, 2H), 7.51 – 7.47 (m, 1H), 7.46 – 7.43 (m, 1H), 7.43 – 7.35 (m, 2H), 7.33 – 7.23 (m, 3H), 7.22 – 7.14 (m, 2H), 7.13 – 7.08 (m, 1H), 2.35 (s, 3H). ^{13}C NMR (101 MHz, Acetone-d_6) δ 189.8, 158.6, 145.1, 142.5, 140.7, 139.3, 137.3, 130.7, 130.6, 129.6, 128.8, 128.0, 126.4, 125.4, 120.7, 120.5, 115.5, 21.3.
IR: 3282, 2923, 2854, 1643, 1558, 1486, 1446, 1346, 1284, 1148, 996, 793, 723 cm^{-1}.
HRMS (ESI) m/z: [M + Na]$^+$ Calcd for $C_{18}H_{16}O_2Na$ 287.1043; Found 287.1035.

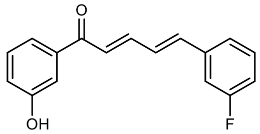

(2*E*,4*E*)-5-(3-fluorophenyl)-1-(3-hydroxyphenyl)penta-2,4-dien-1-one (8o). Prepared according to general procedure D and purified by flash column chromatography to afford the product as a yellow solid (54.9 mg, 82% yield). m.p. = 119 – 121 °C. Eluant: ethyl acetate/petroleum ether (1:5, R_f = 0.30).
^{1}H NMR (400 MHz, Acetone-d_6) δ 8.73 (br, 1H), 7.63 – 7.49 (m, 3H), 7.48 – 7.36 (m, 4H), 7.35 – 7.25 (m, 2H), 7.20 – 7.06 (m, 3H). ^{13}C NMR (101 MHz, Acetone-d_6) δ 189.8, 164.0 (C-F, $^1J_{C-F}$ = 244.1 Hz), 158.6, 144.4, 140.6 (C-F, $^4J_{C-F}$ = 2.9 Hz), 140.5, 139.9 (C-F, $^3J_{C-F}$ = 7.7 Hz),

131.5 (C-F, ^3J C-F = 8.4 Hz), 130.7, 129.6, 127.3, 124.4 (C-F, ^4J C-F = 2.8 Hz), 120.8, 120.5, 116.4 (C-F, ^2J C-F = 21.6 Hz), 115.4, 114.0 (C-F, ^2J C-F = 22.0 Hz). ^{19}F NMR (376 MHz, Acetone-d_6) δ -114.4 (t, J = 11.3 Hz).
IR: 3284, 2924, 2853, 1645, 1562, 1445, 1345, 1276, 1147, 995, 784, 722 cm^{-1}.
HRMS (ESI) m/z: [M + Na]$^+$ Calcd for $C_{17}H_{13}O_2FNa$ 291.0792; Found 291.0784.

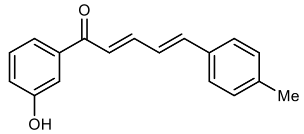

(2*E*,4*E*)-1-(3-hydroxyphenyl)-5-(*p*-tolyl)penta-2,4-dien-1-one (8p). Prepared according to general procedure D and purified by flash column chromatography to afford the product as a yellow solid (50.2 mg, 76% yield). m.p. = 136 – 138 °C. Eluant: ethyl acetate/petroleum ether (1:6, R_f = 0.30).
^{1}H NMR (400 MHz, Acetone-d_6) δ 8.75 (br, 1H), 7.61 – 7.51 (m, 3H), 7.50 – 7.46 (m, 2H), 7.42 – 7.34 (m, 1H), 7.30 – 7.16 (m, 4H), 7.15 – 7.08 (m, 2H), 2.34 (s, 3H). ^{13}C NMR (101 MHz, Acetone-d_6) δ 189.8, 158.6, 145.3, 142.4, 140.7, 140.1, 134.6, 130.6, 130.4 (2C), 128.1 (2C), 127.2, 125.9, 120.6, 120.4, 115.4, 21.3.
IR: 3299, 2923, 2854, 1643, 1561, 1448, 1287, 997, 805, 725 cm^{-1}.
HRMS (ESI) m/z: [M + Na]$^+$ Calcd for $C_{18}H_{16}O_2Na$ 287.1043; Found 287.1030.

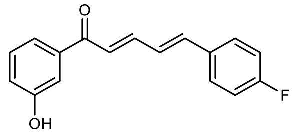

(2*E*,4*E*)-5-(4-fluorophenyl)-1-(3-hydroxyphenyl)penta-2,4-dien-1-one (8q). Prepared according to general procedure D and purified by flash column chromatography to afford the product as a yellow solid (52.9 mg, 79% yield). m.p. = 110 – 112 °C. Eluant: ethyl acetate/petroleum ether (1:5, R_f = 0.30).
^{1}H NMR (400 MHz, Acetone-d_6) δ 8.74 (br, 1H), 7.70 – 7.61 (m, 2H), 7.61 – 7.48 (m, 3H), 7.42 – 7.35 (m, 1H), 7.27 (d, J = 14.8 Hz, 1H), 7.24 – 7.09 (m, 5H). ^{13}C NMR (101 MHz, Acetone-d_6) δ 189.8, 163.9 (C-F, ^1J C-F = 247.8 Hz), 158.6, 144.9, 140.9, 140.6, 133.8 (C-F, ^4J C-F = 3.4 Hz), 130.6, 130.1 (C-F, ^3J C-F = 8.3 Hz, 2C), 128.0 (C-F, ^5J C-F = 2.5 Hz), 126.5, 120.7, 120.5, 116.6 (C-F, ^2J C-F = 22.0 Hz, 2C), 115.5. ^{19}F NMR (376 MHz, Acetone-d_6) δ -113.2 (m).
IR: 3283, 2925, 2853, 1644, 1561, 1506, 1447, 1287, 1230, 1155, 996, 844, 794, 781 cm^{-1}.
HRMS (ESI) m/z: [M + Na]$^+$ Calcd for $C_{17}H_{13}O_2FNa$ 291.0792; Found 291.0783.

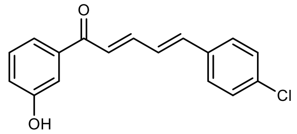

(2*E*,4*E*)-5-(4-chlorophenyl)-1-(3-hydroxyphenyl)penta-2,4-dien-1-one (8r). Prepared according to general procedure D and purified by flash column chromatography to afford the product as a yellow solid (55.4 mg, 78% yield). m.p. = 135 – 137 °C. Eluant: ethyl acetate/petroleum ether (1:6, R_f = 0.30).
^{1}H NMR (400 MHz, Acetone-d_6) δ 8.75 (br, 1H), 7.62 (d, J = 8.2 Hz, 2H), 7.60 – 7.52 (m, 2H), 7.51 – 7.48 (m, 1H), 7.43 (d, J = 8.2 Hz, 2H), 7.41 – 7.35 (m, 1H), 7.34 – 7.20 (m, 2H), 7.19 – 7.08 (m, 2H). ^{13}C NMR (101 MHz, Acetone-d_6) δ 189.8, 158.7, 144.6, 140.64, 140.60, 136.2, 135.0, 130.7, 129.8 (2C), 129.6 (2C), 129.0, 127.0, 120.8, 120.5, 115.5.
IR: 3342, 2925, 2853, 1647, 1568, 1490, 1448, 1283, 1091, 997, 812, 795, 725 cm^{-1}.

HRMS (ESI) m/z: [M + Na]+ Calcd for $C_{17}H_{13}O_2ClNa$ 307.0496; Found 307.0490.

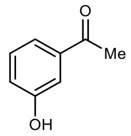

1-(3-hydroxyphenyl)ethan-1-one (10a). Prepared according to procedure for the synthesis of **10a** and purified by flash column chromatography to afford the product as a white solid (21.1 mg, 62% yield). m.p. = 91 – 93 °C. Eluant: ethyl acetate/petroleum ether (1:8, R_f = 0.30).
^{1}H NMR (400 MHz, $CDCl_3$) δ 7.58 – 7.54 (m, 1H), 7.54 – 7.49 (m, 1H), 7.38 – 7.31 (m, 1H), 7.15 – 7.10 (m, 1H), 6.84 (br, 1H), 2.61 (s, 3H). ^{13}C NMR (101 MHz, $CDCl_3$) δ 199.6, 156.5, 138.4, 130.1, 121.2, 121.0, 114.8, 26.9.
IR: 3345, 2924, 2852, 1669, 1598, 1585, 1450, 1360, 1288, 1220, 787 cm^{-1}.
HRMS (ESI) m/z: [M + Na]+ Calcd for $C_8H_8O_2Na$ 159.0417; Found 159.0412.

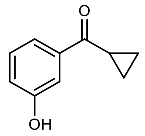

cyclopropyl(3-hydroxyphenyl)methanone (10b). Prepared according to general procedure D and purified by flash column chromatography to afford the product as a yellow oil (22.7 mg, 56% yield). Eluant: ethyl acetate/petroleum ether (1:6, R_f = 0.30).
^{1}H NMR (400 MHz, $CDCl_3$) δ 7.64 – 7.55 (m, 2H), 7.39 – 7.32 (m, 1H), 7.12 – 7.07 (m, 1H), 6.48 (br, 1H), 2.72 – 2.62 (m, 1H), 1.31 – 1.22 (m, 2H), 1.12 – 1.03 (m, 2H). ^{13}C NMR (101 MHz, $CDCl_3$) δ 201.4, 156.4, 139.5, 130.0, 120.8, 120.4, 114.7, 17.5, 12.2 (2C).
IR: 3314, 2925, 2853, 1650, 1582, 1448, 1387, 1276, 1177, 1041, 908, 741 cm^{-1}.
HRMS (ESI) m/z: [M + Na]+ Calcd for $C_{10}H_{10}O_2Na$ 185.0573; Found 185.0578.

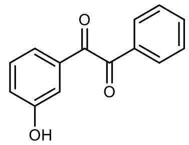

1-(3-hydroxyphenyl)-2-phenylethane-1,2-dione (10c). Prepared according to general procedure D and purified by flash column chromatography to afford the product as a yellow solid (20.3 mg, 36% yield). m.p. = 82 – 84 °C. Eluant: ethyl acetate/petroleum ether (1:6, R_f = 0.30).
^{1}H NMR (400 MHz, $CDCl_3$) δ 8.00 – 7.92 (m, 2H), 7.70 – 7.62 (m, 1H), 7.55 – 7.44 (m, 4H), 7.40 – 7.33 (m, 1H), 7.18 – 7.12 (m, 1H), 5.83 (br, 1H). ^{13}C NMR (101 MHz, $CDCl_3$) δ 194.8, 194.7, 156.5, 135.2, 134.3, 132.9, 130.6, 130.1 (2C), 129.2 (2C), 123.0, 122.7, 115.8.
IR: 3375, 2924, 2853, 1665, 1595, 1582, 1449, 1261, 1229, 749, 717 cm^{-1}.
HRMS (ESI) m/z: [M + Na]+ Calcd for $C_{14}H_{10}O_3Na$ 249.0522; Found 249.0513.

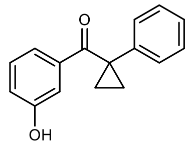

(3-hydroxyphenyl)(1-phenylcyclopropyl)methanone (10d). Prepared according to general procedure D and purified by flash column chromatography to afford the product as a yellow solid (24.4 mg, 41% yield). m.p. = 62 – 64 °C. Eluant: ethyl acetate/petroleum ether (1:6, R_f = 0.30).
^{1}H NMR (400 MHz, $CDCl_3$) δ 7.33 – 7.27 (m, 2H), 7.25 – 7.13 (m, 5H), 7.13 – 7.07 (m, 1H), 6.92 – 6.84 (m, 1H), 5.66 (br, 1H), 1.70 – 1.62 (m, 2H), 1.40 – 1.32 (m, 2H). ^{13}C NMR (101

MHz, $CDCl_3$) δ 200.9, 155.7, 140.9, 138.5, 129.4, 128.8 (2C), 128.0 (2C), 126.8, 122.3, 119.6, 116.0, 35.4, 16.6 (2C).
IR: 3358, 2954, 2924, 1656, 1596, 1583, 1446, 1297, 1279, 1172, 1157, 745, 698 cm^{-1}.
HRMS (ESI) m/z: $[M + Na]^+$ Calcd for $C_{16}H_{14}O_2Na$ 261.0886; Found 261.0897.

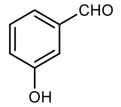

3-hydroxybenzaldehyde (10e). Prepared according to general procedure D and purified by flash column chromatography to afford the product as a yellow solid (26.2 mg, 86% yield). m.p. = 100 – 103 °C. Eluant: ethyl acetate/petroleum ether (1:10, R_f = 0.30).
^{1}H NMR (400 MHz, $CDCl_3$) δ 9.96 (s, 1H), 7.49 – 7.40 (m, 2H), 7.40 – 7.37 (m, 1H), 7.19 – 7.13 (m, 1H), 5.92 (br, 1H). ^{13}C NMR (101 MHz, $CDCl_3$) δ 192.7, 156.6, 137.9, 130.6, 123.7, 122.3, 114.9.
IR: 3210, 2956, 2924, 1667, 1580, 1493, 1281, 1249, 1172, 783 cm^{-1}.
HRMS (ESI) m/z: $[M + Na]^+$ Calcd for $C_7H_6O_2Na$ 145.0260; Found 145.0266.

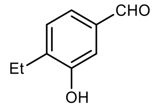

4-ethyl-3-hydroxybenzaldehyde (10f). Prepared according to general procedure D and purified by flash column chromatography to afford the product as a yellow solid (33.4 mg, 89% yield). m.p. = 55 – 57 °C. Eluant: ethyl acetate/petroleum ether (1:10, R_f = 0.30).
^{1}H NMR (400 MHz, Acetone-d_6) δ 9.90 (s, 1H), 8.83 (br, 1H), 7.40 – 7.32 (m, 3H), 2.72 (q, J = 7.5 Hz, 2H), 1.22 (t, J = 7.5 Hz, 3H). ^{13}C NMR (101 MHz, Acetone-d_6) δ 192.5, 156.4, 139.0, 137.0, 130.6, 123.3, 114.4, 24.2, 14.1.
IR: 3355, 2969, 2934, 1679, 1605, 1584, 1433, 1395, 821, 760 cm^{-1}.
HRMS (ESI) m/z: $[M + Na]^+$ Calcd for $C_9H_{10}O_2Na$ 173.0573; Found 173.0569.

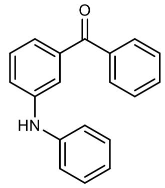

phenyl(3-(phenylamino)phenyl)methanone (12a). Prepared according to general procedure E and purified by flash column chromatography to afford the product as a yellow solid (49.8 mg, 73% yield). m.p. = 72 – 74 °C. Eluant: ethyl acetate/petroleum ether (1:20, R_f = 0.30).
^{1}H NMR (400 MHz, $CDCl_3$) δ 7.85 – 7.78 (m, 2H), 7.61 – 7.53 (m, 1H), 7.51 – 7.43 (m, 3H), 7.38 – 7.25 (m, 5H), 7.15 – 7.06 (m, 2H), 7.00 – 6.93 (m, 1H), 5.89 (br, 1H). ^{13}C NMR (101 MHz, $CDCl_3$) δ 196.9, 143.7, 142.4, 138.9, 137.7, 132.6, 130.2 (2C), 129.6 (2C), 129.3, 128.4 (2C), 122.6, 121.9, 120.9, 118.6 (2C), 118.4.
IR: 3358, 3056, 2923, 2852, 1648, 1590, 1577, 1494, 1449, 1413, 1318, 1275, 986, 750, 714, 693 cm^{-1}.
HRMS (ESI) m/z: $[M + Na]^+$ Calcd for $C_{19}H_{15}NONa$ 296.1046; Found 296.1052.

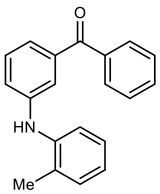

phenyl(3-(*o*-tolylamino)phenyl)methanone (12b). Prepared according to general procedure E and purified by flash column chromatography to afford the product as a yellow oil (43.8 mg, 61% yield). Eluant: ethyl acetate/petroleum ether (1:20, R_f = 0.30).
^{1}H NMR (400 MHz, $CDCl_3$) δ 7.87 – 7.75 (m, 2H), 7.61 – 7.52 (m, 1H), 7.51 – 7.41 (m, 2H), 7.39 – 7.34 (m, 1H), 7.33 – 7.28 (m, 1H), 7.28 – 7.19 (m, 3H), 7.18 – 7.09 (m, 2H), 7.03 – 6.94 (m, 1H), 5.55 (br, 1H), 2.25 (s, 3H). ^{13}C NMR (101 MHz, $CDCl_3$) δ 196.9, 144.6, 140.4, 138.9, 137.7, 132.5, 131.2, 130.2 (2C), 129.4, 129.2, 128.3 (2C), 127.0, 123.0, 122.1, 120.4, 119.9, 118.0, 18.1.
IR: 3368, 3057, 2923, 1649, 1595, 1575, 1514, 1318, 1274, 985, 748, 716, 706 cm^{-1}.
HRMS (ESI) m/z: $[M + Na]^+$ Calcd for $C_{20}H_{17}NONa$ 310.1202; Found 310.1216.

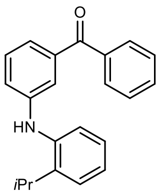

(3-((2-isopropylphenyl)amino)phenyl)(phenyl)methanone (12c). Prepared according to general procedure E and purified by flash column chromatography to afford the product as a yellow oil (55.9 mg, 71% yield). Eluant: ethyl acetate/petroleum ether (1:20, R_f = 0.30).
^{1}H NMR (400 MHz, $CDCl_3$) δ 7.86 – 7.78 (m, 2H), 7.61 – 7.53 (m, 1H), 7.50 – 7.42 (m, 2H), 7.37 – 7.23 (m, 4H), 7.22 – 7.14 (m, 2H), 7.13 – 7.08 (m, 1H), 7.07 – 7.02 (m, 1H), 5.56 (br, 1H), 3.23 – 3.09 (m, 1H), 1.23 (d, J = 6.8 Hz, 6H). ^{13}C NMR (101 MHz, $CDCl_3$) δ 197.0, 145.9, 141.7, 138.9, 138.8, 137.8, 132.5, 130.2 (2C), 129.2, 128.3 (2C), 126.7, 126.4, 124.5, 122.8, 121.6, 119.5, 117.0, 27.9, 23.2 (2C).
IR: 3370, 3059, 2960, 2924, 1650, 1595, 1576, 1509, 1487, 1447, 1318, 1279, 1082, 754, 722, 711 cm^{-1}.
HRMS (ESI) m/z: $[M + Na]^+$ Calcd for $C_{22}H_{21}NONa$ 338.1515; Found 338.1526.

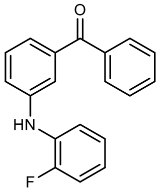

(3-((2-fluorophenyl)amino)phenyl)(phenyl)methanone (12d). Prepared according to general procedure E and purified by flash column chromatography to afford the product as a yellow oil (61.8 mg, 85% yield). Eluant: ethyl acetate/petroleum ether (1:15, R_f = 0.30).
^{1}H NMR (400 MHz, $CDCl_3$) δ 7.86 – 7.79 (m, 2H), 7.62 – 7.55 (m, 1H), 7.54 – 7.50 (m, 1H), 7.50 – 7.44 (m, 2H), 7.41 – 7.27 (m, 4H), 7.14 – 6.99 (m, 2H), 6.93 – 6.85 (m, 1H), 5.94 (br, 1H). ^{13}C NMR (101 MHz, $CDCl_3$) δ 196.7, 153.5 (C-F, $^{1}J_{C-F}$ = 241.9 Hz), 142.6, 139.0, 137.6, 132.6, 130.8 (C-F, $^{2}J_{C-F}$ = 11.1 Hz), 130.2 (2C), 129.3, 128.4 (2C), 124.5 (C-F, $^{3}J_{C-F}$ = 3.7 Hz), 123.4, 121.69 (C-F, $^{3}J_{C-F}$ = 7.3 Hz), 121.68, 119.1, 118.2 (C-F, $^{4}J_{C-F}$ = 2.1 Hz), 115.8 (C-F, $^{2}J_{C-F}$ = 19.3 Hz). ^{19}F NMR (376 MHz, $CDCl_3$) δ -131.0.

IR: 3357, 3059, 2922, 1651, 1618, 1597, 1578, 1320, 1273, 1099, 779, 715 cm^{-1}.
HRMS (ESI) m/z: $[M + Na]^+$ Calcd for $C_{19}H_{14}NOFNa$ 314.0952; Found 314.0960.

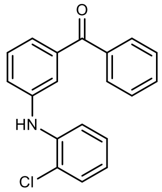

(3-((2-chlorophenyl)amino)phenyl)(phenyl)methanone (12e). Prepared according to general procedure E and purified by flash column chromatography to afford the product as a yellow oil (62.2 mg, 81% yield). Eluant: ethyl acetate/petroleum ether (1:20, R_f = 0.30).
^{1}H NMR (400 MHz, $CDCl_3$) δ 7.98 – 7.78 (m, 2H), 7.64 – 7.53 (m, 2H), 7.52 – 7.44 (m, 2H), 7.43 – 7.28 (m, 5H), 7.19 – 7.09 (m, 1H), 6.88 – 6.80 (m, 1H), 6.20 (br, 1H). ^{13}C NMR (101 MHz, $CDCl_3$) δ 196.5, 142.1, 139.5, 139.0, 137.5, 132.7, 130.2 (2C), 130.0, 129.4, 128.4 (2C), 127.6, 124.1, 123.2, 122.4, 121.4, 120.5, 116.4.
IR: 3358, 3027, 2923, 1652, 1578, 1514, 1446, 1315, 1272, 738, 718, 706 cm^{-1}.
HRMS (ESI) m/z: $[M + Na]^+$ Calcd for $C_{19}H_{14}NOClNa$ 330.0656; Found 330.0665.

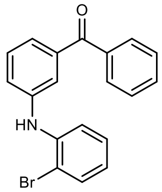

(3-((2-bromophenyl)amino)phenyl)(phenyl)methanone (12f). Prepared according to general procedure E and purified by flash column chromatography to afford the product as a yellow oil (71.9 mg, 82% yield). Eluant: ethyl acetate/petroleum ether (1:20, R_f = 0.30).
^{1}H NMR (400 MHz, $CDCl_3$) δ 7.88 – 7.78 (m, 2H), 7.65 – 7.44 (m, 5H), 7.43 – 7.33 (m, 3H), 7.32 – 7.27 (m, 1H), 7.23 – 7.13 (m, 1H), 6.84 – 6.73 (m, 1H), 6.18 (br, 1H). ^{13}C NMR (101 MHz, $CDCl_3$) δ 196.5, 142.2, 140.7, 139.1, 137.6, 133.3, 132.7, 130.2 (2C), 129.4, 128.4 (2C), 128.3, 124.2, 123.2, 122.0, 120.6, 116.6, 113.1.
IR: 3359, 3060, 2923, 1651, 1577, 1310, 1272, 1022, 745, 716, 703 cm^{-1}.
HRMS (ESI) m/z: $[M + Na]^+$ Calcd for $C_{19}H_{14}NOBrNa$ 374.0151; Found 374.0167.

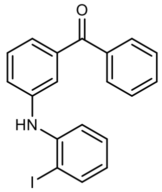

(3-((2-iodophenyl)amino)phenyl)(phenyl)methanone (12g). Prepared according to general procedure E and purified by flash column chromatography to afford the product as a yellow oil (66.8 mg, 67% yield). Eluant: ethyl acetate/petroleum ether (1:25, R_f = 0.30).
^{1}H NMR (400 MHz, $CDCl_3$) δ 7.87 – 7.81 (m, 2H), 7.81 – 7.76 (m, 1H), 7.64 – 7.56 (m, 1H), 7.56 – 7.52 (m, 1H), 7.52 – 7.45 (m, 2H), 7.44 – 7.36 (m, 2H), 7.35 – 7.30 (m, 1H), 7.29 – 7.20 (m, 2H), 6.71 – 6.64 (m, 1H), 6.00 (br, 1H). ^{13}C NMR (101 MHz, $CDCl_3$) δ 196.6, 143.2, 142.6, 139.8, 139.1, 137.6, 132.7, 130.2 (2C), 129.4, 129.3, 128.4 (2C), 124.0, 123.0, 122.9, 120.2, 116.8, 89.8.
IR: 3374, 3058, 2920, 2850, 1651, 1574, 1510, 1484, 1456, 1441, 1312, 1277, 1010, 748, 717, 703 cm^{-1}.

HRMS (ESI) m/z: $[M + Na]^+$ Calcd for $C_{19}H_{14}NOINa$ 422.0012; Found 421.9999.

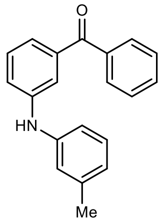

phenyl(3-(*m*-tolylamino)phenyl)methanone (12h). Prepared according to general procedure E and purified by flash column chromatography to afford the product as a yellow oil (48.8 mg, 68% yield). Eluant: ethyl acetate/petroleum ether (1:20, R_f = 0.30).

^{1}H NMR (400 MHz, $CDCl_3$) δ 7.86 – 7.77 (m, 2H), 7.62 – 7.54 (m, 1H), 7.53 – 7.42 (m, 3H), 7.38 – 7.31 (m, 1H), 7.31 – 7.26 (m, 2H), 7.21 – 7.13 (m, 1H), 6.95 – 6.88 (m, 2H), 6.82 – 6.72 (m, 1H), 5.84 (br, 1H), 2.31 (s, 3H). ^{13}C NMR (101 MHz, $CDCl_3$) δ 196.9, 143.7, 142.3, 139.5, 138.9, 137.7, 132.6, 130.2 (2C), 129.4, 129.3, 128.4 (2C), 122.8, 122.5, 121.0, 119.2, 118.5, 115.7, 21.7.

IR: 3362, 3033, 2922, 2853, 1649, 1575, 1484, 1446, 1405, 1319, 1275, 1166, 986, 775, 714, 693 cm^{-1}.

HRMS (ESI) m/z: $[M + Na]^+$ Calcd for $C_{20}H_{17}NONa$ 310.1202; Found 310.1193.

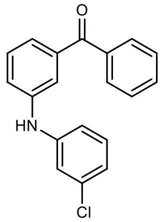

(3-((3-chlorophenyl)amino)phenyl)(phenyl)methanone (12i). Prepared according to general procedure E and purified by flash column chromatography to afford the product as a yellow solid (55.3 mg, 72% yield). m.p. = 68 – 70 °C. Eluant: ethyl acetate/petroleum ether (1:20, R_f = 0.30).

^{1}H NMR (400 MHz, $CDCl_3$) δ 7.88 – 7.76 (m, 2H), 7.64 – 7.55 (m, 1H), 7.54 – 7.44 (m, 3H), 7.42 – 7.33 (m, 2H), 7.32 – 7.27 (m, 1H), 7.21 – 7.12 (m, 1H), 7.11 – 7.04 (m, 1H), 6.98 – 6.85 (m, 2H), 5.97 (br, 1H). ^{13}C NMR (101 MHz, $CDCl_3$) δ 196.7, 144.1, 142.5, 139.0, 137.5, 135.2, 132.7, 130.6, 130.2 (2C), 129.5, 128.5 (2C), 123.6, 122.1, 121.4, 119.6, 117.4, 115.9.

IR: 3354, 2923, 2852, 1650, 1585, 1519, 1479, 1446, 1319, 1277, 1265, 1075, 991, 769, 720, 703 cm^{-1}.

HRMS (ESI) m/z: $[M + Na]^+$ Calcd for $C_{19}H_{14}NOClNa$ 330.0656; Found 330.0649.

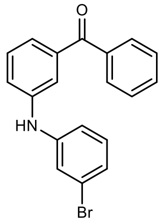

(3-((3-bromophenyl)amino)phenyl)(phenyl)methanone (12j). Prepared according to general procedure E and purified by flash column chromatography to afford the product as a yellow solid (62.3 mg, 71% yield). m.p. = 65 – 67 °C. Eluant: ethyl acetate/petroleum ether (1:20, R_f = 0.30).

^{1}H NMR (400 MHz, $CDCl_3$) δ 7.87 – 7.78 (m, 2H), 7.63 – 7.54 (m, 1H), 7.53 – 7.43 (m, 3H), 7.42 – 7.33 (m, 2H), 7.32 – 7.27 (m, 1H), 7.25 – 7.19 (m, 1H), 7.15 – 7.08 (m, 1H), 7.07 – 7.01 (m, 1H), 7.00 – 6.93 (m, 1H), 5.94 (br, 1H). ^{13}C NMR (101 MHz, $CDCl_3$) δ 196.6, 144.2, 142.5,

139.0, 137.6, 132.7, 130.9, 130.2 (2C), 129.5, 128.5 (2C), 124.3, 123.6, 123.3, 122.1, 120.4, 119.7, 116.4.
IR: 3351, 2923, 2853, 1647, 1574, 1515, 1475, 1444, 1317, 1278, 989, 769, 716 cm^{-1}.
HRMS (ESI) m/z: [M + Na]$^+$ Calcd for $C_{19}H_{14}NOBrNa$ 374.0151; Found 374.0133.

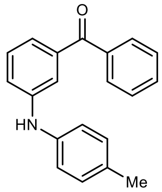

phenyl(3-(*p*-tolylamino)phenyl)methanone (12k). Prepared according to general procedure E and purified by flash column chromatography to afford the product as a yellow solid (59.6 mg, 83% yield). m.p. = 77 – 79 °C. Eluant: ethyl acetate/petroleum ether (1:20, R_f = 0.30).
^{1}H NMR (400 MHz, $CDCl_3$) δ 7.85 – 7.78 (m, 2H), 7.63 – 7.53 (m, 1H), 7.52 – 7.43 (m, 2H), 7.42 – 7.37 (m, 1H), 7.35 – 7.27 (m, 1H), 7.24 – 7.18 (m, 2H), 7.15 – 7.07 (m, 2H), 7.06 – 6.98 (m, 2H), 5.77 (br, 1H), 2.30 (s, 3H). ^{13}C NMR (101 MHz, $CDCl_3$) δ 196.9, 144.4, 139.6, 138.9, 137.8, 132.5, 131.9, 130.2 (2C), 130.1 (2C), 129.2, 128.3 (2C), 122.0, 120.1, 119.6 (2C), 117.6, 20.9.
IR: 3365, 2918, 2849, 1646, 1595, 1515, 1321, 1275, 811, 716 cm^{-1}.
HRMS (ESI) m/z: [M + Na]$^+$ Calcd for $C_{20}H_{17}NONa$ 310.1202; Found 310.1214.

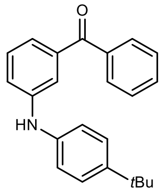

(3-((4-(*tert*-butyl)phenyl)amino)phenyl)(phenyl)methanone (12l). Prepared according to general procedure E and purified by flash column chromatography to afford the product as a yellow solid (65.8 mg, 80% yield). m.p. = 78 – 80 °C. Eluant: ethyl acetate/petroleum ether (1:20, R_f = 0.30).
^{1}H NMR (400 MHz, $CDCl_3$) δ 7.87 – 7.77 (m, 2H), 7.60 – 7.52 (m, 1H), 7.50 – 7.39 (m, 3H), 7.36 – 7.27 (m, 3H), 7.26 – 7.18 (m, 2H), 7.11 – 7.01 (m, 2H), 5.83 (br, 1H), 1.30 (s, 9H). ^{13}C NMR (101 MHz, $CDCl_3$) δ 196.9, 145.1, 144.3, 139.6, 138.9, 137.8, 132.5, 130.2 (2C), 129.2, 128.3 (2C), 126.4 (2C), 122.1, 120.3, 118.9 (2C), 117.8, 34.3, 31.6 (3C).
IR: 3361, 3056, 2958, 2864, 1649, 1594, 1577, 1516, 1482, 1445, 1266, 825, 778, 710 cm^{-1}.
HRMS (ESI) m/z: [M + Na]$^+$ Calcd for $C_{23}H_{23}NONa$ 352.1672; Found 352.1659.

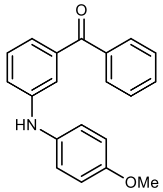

(3-((4-methoxyphenyl)amino)phenyl)(phenyl)methanone (12m). Prepared according to general procedure E and purified by flash column chromatography to afford the product as a yellow solid (43.2 mg, 57% yield). m.p. = 65 – 67 °C. Eluant: ethyl acetate/petroleum ether (1:12, R_f = 0.30).
^{1}H NMR (400 MHz, $CDCl_3$) δ 7.84 – 7.79 (m, 2H), 7.61 – 7.54 (m, 1H), 7.50 – 7.43 (m, 2H), 7.34 – 7.26 (m, 2H), 7.20 – 7.15 (m, 1H), 7.13 – 7.06 (m, 3H), 6.90 – 6.84 (m, 2H), 5.65 (br, 1H),

3.80 (s, 3H). ^{13}C NMR (101 MHz, $CDCl_3$) δ 197.0, 155.9, 145.7, 138.8, 137.8, 134.9, 132.5, 130.2 (2C), 129.2, 128.3 (2C), 123.0 (2C), 121.4, 119.0, 116.4, 114.9 (2C), 55.7.
IR: 3357, 3057, 2929, 2833, 1648, 1594, 1577, 1506, 1483, 1442, 1320, 1232, 1031, 820, 778, 714 cm^{-1}.
HRMS (ESI) m/z: [M + Na]$^+$ Calcd for $C_{20}H_{17}NO_2Na$ 326.1151; Found 326.1137.

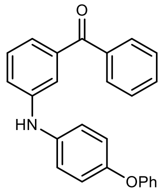

(3-((4-phenoxyphenyl)amino)phenyl)(phenyl)methanone (12n). Prepared according to general procedure E and purified by flash column chromatography to afford the product as a yellow solid (70.3 mg, 77% yield). m.p. = 79 – 81 °C. Eluant: ethyl acetate/petroleum ether (1:15, R_f = 0.30).
^{1}H NMR (400 MHz, $CDCl_3$) δ 7.85 – 7.78 (m, 2H), 7.62 – 7.52 (m, 1H), 7.50 – 7.43 (m, 2H), 7.42 – 7.38 (m, 1H), 7.36 – 7.27 (m, 3H), 7.25 – 7.16 (m, 2H), 7.13 – 7.03 (m, 3H), 7.02 – 6.92 (m, 4H), 5.85 (br, 1H). ^{13}C NMR (101 MHz, $CDCl_3$) δ 196.9, 158.1, 152.1, 144.6, 139.0, 137.9, 137.8, 132.6, 130.2 (2C), 129.8 (2C), 129.3, 128.4 (2C), 123.0, 122.2, 121.4 (2C), 120.6 (2C), 120.0, 118.3 (2C), 117.5.
IR: 3360, 2954, 2922, 1649, 1594, 1578, 1503, 1485, 1320, 1217, 778, 715, 689 cm^{-1}.
HRMS (ESI) m/z: [M + H]$^+$ Calcd for $C_{25}H_{20}NO_2$ 366.1489; Found 366.1482.

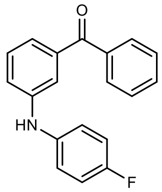

(3-((4-fluorophenyl)amino)phenyl)(phenyl)methanone (12o). Prepared according to general procedure E and purified by flash column chromatography to afford the product as a yellow solid (59.7 mg, 82% yield). m.p. = 69 – 71 °C. Eluant: ethyl acetate/petroleum ether (1:15, R_f = 0.30).
^{1}H NMR (400 MHz, $CDCl_3$) δ 7.84 – 7.77 (m, 2H), 7.62 – 7.53 (m, 1H), 7.51 – 7.41 (m, 2H), 7.41 – 7.35 (m, 1H), 7.34 – 7.27 (m, 1H), 7.26 – 7.20 (m, 1H), 7.19 – 7.13 (m, 1H), 7.12 – 7.03 (m, 2H), 7.02 – 6.92 (m, 2H), 5.83 (br, 1H). ^{13}C NMR (101 MHz, $CDCl_3$) δ 196.9, 158.6 (C-F, $^1J_{C-F}$ = 241.1 Hz), 144.5, 139.0, 138.2 (C-F, $^4J_{C-F}$ = 2.7 Hz), 137.7, 132.6, 130.2 (2C), 129.3, 128.4 (2C), 122.3, 121.5 (C-F, $^3J_{C-F}$ = 7.9 Hz, 2C), 120.0, 117.4, 116.2 (C-F, $^2J_{C-F}$ = 22.4 Hz, 2C). ^{19}F NMR (376 MHz, $CDCl_3$) δ -120.6 (dt, J = 8.9, 4.9 Hz).
IR: 3355, 3058, 2923, 2853, 1648, 1595, 1578, 1503, 1445, 1320, 1274, 822, 778, 713 cm^{-1}.
HRMS (ESI) m/z: [M + Na]$^+$ Calcd for $C_{19}H_{14}NOFNa$ 314.0952; Found 314.0940.

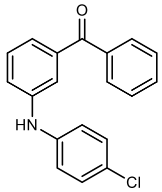

(3-((4-chlorophenyl)amino)phenyl)(phenyl)methanone (12p). Prepared according to general procedure E and purified by flash column chromatography to afford the product as a yellow solid (64.5 mg, 84% yield). m.p. = 97 – 99 °C. Eluant: ethyl acetate/petroleum ether (1:20, R_f = 0.30).
^{1}H NMR (400 MHz, $CDCl_3$) δ 7.86 – 7.77 (m, 2H), 7.63 – 7.55 (m, 1H), 7.51 – 7.42 (m, 3H),

7.38 – 7.32 (m, 1H), 7.31 – 7.27 (m, 1H), 7.26 – 7.18 (m, 3H), 7.07 – 6.97 (m, 2H), 5.88 (br, 1H). ^{13}C NMR (101 MHz, CDCl$_3$) δ 196.7, 143.3, 141.1, 139.1, 137.6, 132.7, 130.2 (2C), 129.6 (2C), 129.4, 128.4 (2C), 126.5, 123.1, 121.2, 119.7 (2C), 118.6.
IR: 3351, 2923, 2852, 1647, 1587, 1576, 1512, 1487, 1445, 1318, 1273, 810, 779, 716 cm^{-1}.
HRMS (ESI) m/z: [M + Na]$^+$ Calcd for $C_{19}H_{14}NOClNa$ 330.0656; Found 330.0643.

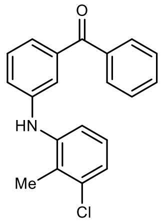

(3-((3-chloro-2-methylphenyl)amino)phenyl)(phenyl)methanone (12q). Prepared according to general procedure E and purified by flash column chromatography to afford the product as a yellow solid (60.2 mg, 75% yield). m.p. = 79 – 81 °C. Eluant: ethyl acetate/petroleum ether (1:20, R_f = 0.30).
^{1}H NMR (400 MHz, CDCl$_3$) δ 7.86 – 7.76 (m, 2H), 7.63 – 7.53 (m, 1H), 7.52 – 7.42 (m, 2H), 7.38 – 7.30 (m, 2H), 7.29 – 7.22 (m, 1H), 7.20 – 7.01 (m, 4H), 5.62 (br, 1H), 2.31 (s, 3H). ^{13}C NMR (101 MHz, CDCl$_3$) δ 196.8, 144.3, 142.0, 139.0, 137.6, 135.6, 132.6, 130.2 (2C), 129.3, 128.4 (2C), 127.9, 127.1, 124.0, 122.6, 120.8, 118.7, 118.3, 14.8.
IR: 3361, 3058, 2923, 2853, 1650, 1594, 1570, 1446, 1317, 1265, 1013, 774, 718 cm^{-1}.
HRMS (ESI) m/z: [M + Na]$^+$ Calcd for $C_{20}H_{16}NOClNa$ 344.0813; Found 344.0807.

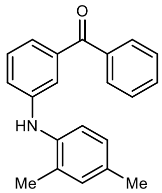

(3-((2,4-dimethylphenyl)amino)phenyl)(phenyl)methanone (12r). Prepared according to general procedure E and purified by flash column chromatography to afford the product as a yellow oil (63.2 mg, 84% yield). Eluant: ethyl acetate/petroleum ether (1:20, R_f = 0.30).
^{1}H NMR (400 MHz, CDCl$_3$) δ 7.85 – 7.77 (m, 2H), 7.61 – 7.53 (m, 1H), 7.51 – 7.42 (m, 2H), 7.31 – 7.25 (m, 2H), 7.21 – 7.11 (m, 2H), 7.08 – 7.00 (m, 2H), 7.00 – 6.94 (m, 1H), 5.45 (br, 1H), 2.30 (s, 3H), 2.22 (s, 3H). ^{13}C NMR (101 MHz, CDCl$_3$) δ 197.0, 145.5, 138.8, 137.8, 137.5, 133.3, 132.5, 131.9, 130.7, 130.2 (2C), 129.1, 128.3 (2C), 127.5, 121.6, 121.4, 119.4, 116.9, 20.9, 18.0.
IR: 3368, 3023, 2920, 2854, 1649, 1595, 1577, 1504, 1318, 1272, 778, 716 cm^{-1}.
HRMS (ESI) m/z: [M + Na]$^+$ Calcd for $C_{21}H_{19}NONa$ 324.1359; Found 324.1347.

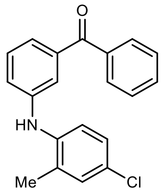

(3-((4-chloro-2-methylphenyl)amino)phenyl)(phenyl)methanone (12s). Prepared according to general procedure E and purified by flash column chromatography to afford the product as a yellow oil (66.6 mg, 83% yield). Eluant: ethyl acetate/petroleum ether (1:20, R_f = 0.30).

^{1}H NMR (400 MHz, $CDCl_3$) δ 7.86 – 7.76 (m, 2H), 7.62 – 7.53 (m, 1H), 7.51 – 7.42 (m, 2H), 7.36 – 7.28 (m, 2H), 7.27 – 7.21 (m, 1H), 7.20 – 7.14 (m, 2H), 7.13 – 7.05 (m, 2H), 5.52 (br, 1H), 2.22 (s, 3H). ^{13}C NMR (101 MHz, $CDCl_3$) δ 196.8, 144.2, 139.1, 139.0, 137.6, 132.6, 131.2, 130.9, 130.1 (2C), 129.3, 128.4 (2C), 127.6, 126.9, 122.5, 121.0, 120.6, 118.0, 17.9.
IR: 3371, 3059, 2924, 2853, 1650, 1595, 1579, 1513, 1485, 1447, 1274, 779, 717, 703 cm^{-1}.
HRMS (ESI) m/z: $[M + Na]^+$ Calcd for $C_{20}H_{16}NOClNa$ 344.0813; Found 344.0799.

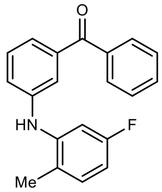

(3-((5-fluoro-2-methylphenyl)amino)phenyl)(phenyl)methanone (12t). Prepared according to general procedure E and purified by flash column chromatography to afford the product as a yellow oil (54.1 mg, 71% yield). Eluant: ethyl acetate/petroleum ether (1:15, R_f = 0.30).
^{1}H NMR (400 MHz, $CDCl_3$) δ 7.88 – 7.78 (m, 2H), 7.63 – 7.55 (m, 1H), 7.54 – 7.46 (m, 2H), 7.45 – 7.42 (m, 1H), 7.41 – 7.31 (m, 2H), 7.28 – 7.22 (m, 1H), 7.15 – 7.08 (m, 1H), 7.00 – 6.92 (m, 1H), 6.65 – 6.56 (m, 1H), 5.58 (br, 1H), 2.21 (s, 3H). ^{13}C NMR (101 MHz, $CDCl_3$) δ 196.7, 162.0 (C-F, $^{1}J_{C-F}$ = 242.5 Hz), 143.1, 142.2 (C-F, $^{3}J_{C-F}$ = 10.0 Hz), 139.0, 137.6, 132.7, 131.9 (C-F, $^{3}J_{C-F}$ = 9.3 Hz), 130.2 (2C), 129.4, 128.4 (2C), 123.4, 122.7 (C-F, $^{4}J_{C-F}$ = 2.9 Hz), 122.0, 119.7, 108.3 (C-F, $^{2}J_{C-F}$ = 21.0 Hz), 104.4 (C-F, $^{2}J_{C-F}$ = 24.9 Hz), 17.4. ^{19}F NMR (376 MHz, $CDCl_3$) δ -115.3 (m).
IR: 3370, 3060, 2923, 2853, 1650, 1595, 1579, 1519, 1414, 1319, 1275, 1156, 779, 715 cm^{-1}.
HRMS (ESI) m/z: $[M + Na]^+$ Calcd for $C_{20}H_{16}NOFNa$ 328.1108; Found 328.1101.

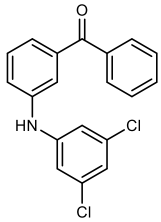

(3-((3,5-dichlorophenyl)amino)phenyl)(phenyl)methanone (12u). Prepared according to general procedure E and purified by flash column chromatography to afford the product as a yellow solid (54.6 mg, 64% yield). m.p. = 76 – 78 °C. Eluant: ethyl acetate/petroleum ether (1:15, R_f = 0.30).
^{1}H NMR (400 MHz, $CDCl_3$) δ 7.87 – 7.78 (m, 2H), 7.64 – 7.56 (m, 1H), 7.54 – 7.47 (m, 3H), 7.46 – 7.39 (m, 2H), 7.36 – 7.29 (m, 1H), 6.95 – 6.89 (m, 2H), 6.89 – 6.85 (m, 1H), 6.04 (br, 1H). ^{13}C NMR (101 MHz, $CDCl_3$) δ 196.6, 145.2, 141.5, 139.1, 137.4, 135.8 (2C), 132.8, 130.2 (2C), 129.7, 128.5 (2C), 124.5, 123.2, 120.84, 120.82, 115.1 (2C).
IR: 3350, 3061, 2922, 2852, 1648, 1571, 1444, 1319, 1274, 1111, 942, 799, 781, 718 cm^{-1}.
HRMS (ESI) m/z: $[M + H]^+$ Calcd for $C_{19}H_{14}NOCl_2$ 342.0447; Found 342.0432.

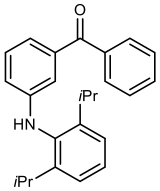

(3-((2,6-diisopropylphenyl)amino)phenyl)(phenyl)methanone (12v). Prepared according to general procedure E and purified by flash column chromatography to afford the product as a yellow solid (70.5 mg, 79% yield). m.p. = 101 – 103 °C. Eluant: ethyl acetate/petroleum ether (1:20, R_f = 0.30).

^{1}H NMR (400 MHz, $CDCl_3$) δ 7.85 – 7.76 (m, 2H), 7.59 – 7.51 (m, 1H), 7.49 – 7.40 (m, 2H), 7.33 – 7.26 (m, 1H), 7.24 – 7.15 (m, 3H), 7.09 – 7.00 (m, 2H), 6.64 – 6.55 (m, 1H), 5.31 (br, 1H), 3.28 – 3.11 (m, 2H), 1.15 (d, J = 6.8 Hz, 12H). ^{13}C NMR (101 MHz, $CDCl_3$) δ 197.2, 148.4, 147.7 (2C), 138.8, 137.9, 134.6, 132.4, 130.2 (2C), 129.1, 128.3 (2C), 127.8, 124.1 (2C), 120.0, 116.5, 114.1, 28.4 (2C), 24.0 (4C).

IR: 3369, 3062, 2961, 2867, 1650, 1600, 1578, 1502, 1469, 1446, 1321, 799, 724, 710 cm^{-1}.

HRMS (ESI) m/z: [M + Na]$^+$ Calcd for $C_{25}H_{27}NONa$ 380.1985; Found 380.1976.

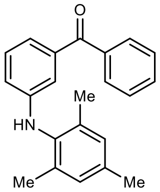

(3-(mesitylamino)phenyl)(phenyl)methanone (12w). Prepared according to general procedure E and purified by flash column chromatography to afford the product as a yellow solid (70.1 mg, 89% yield). m.p. = 103 – 105 °C. Eluant: ethyl acetate/petroleum ether (1:20, R_f = 0.30).

^{1}H NMR (400 MHz, $CDCl_3$) δ 7.85 – 7.76 (m, 2H), 7.60 – 7.51 (m, 1H), 7.49 – 7.40 (m, 2H), 7.22 – 7.15 (m, 1H), 7.09 – 7.04 (m, 1H), 7.03 – 6.99 (m, 1H), 6.93 (s, 2H), 6.63 – 6.57 (m, 1H), 5.27 (br, 1H), 2.29 (s, 3H), 2.18 (s, 6H). ^{13}C NMR (101 MHz, $CDCl_3$) δ 197.2, 147.0, 138.8, 138.0, 136.2 (2C), 136.0, 134.9, 132.3, 130.2 (2C), 129.5 (2C), 129.1, 128.2 (2C), 120.2, 116.7, 114.4, 21.0, 18.4 (2C).

IR: 3368, 2917, 2853, 1649, 1595, 1577, 1500, 1480, 1446, 1320, 1272, 1218, 984, 778, 719, 705 cm^{-1}.

HRMS (ESI) m/z: [M + Na]$^+$ Calcd for $C_{22}H_{21}NONa$ 338.1515; Found 338.1507.

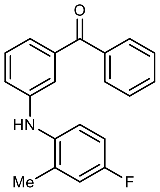

(3-((4-fluoro-2-methylphenyl)amino)phenyl)(phenyl)methanone (12x). Prepared according to general procedure E and purified by flash column chromatography to afford the product as a yellow solid (59.5 mg, 78% yield). m.p. = 58 – 60 °C. Eluant: ethyl acetate/petroleum ether (1:15, R_f = 0.30).

^{1}H NMR (400 MHz, $CDCl_3$) δ 7.88 – 7.76 (m, 2H), 7.62 – 7.53 (m, 1H), 7.52 – 7.42 (m, 2H), 7.32 – 7.26 (m, 1H), 7.24 – 7.21 (m, 1H), 7.21 – 7.15 (m, 2H), 7.00 – 6.92 (m, 2H), 6.91 – 6.83 (m, 1H), 5.41 (br, 1H), 2.24 (s, 3H). ^{13}C NMR (101 MHz, $CDCl_3$) δ 197.0, 159.5 (C-F, $^1J_{C-F}$ = 242.7 Hz), 145.7, 138.9, 137.7, 136.0 (C-F, $^4J_{C-F}$ = 2.8 Hz), 134.0 (C-F, $^3J_{C-F}$ = 7.8 Hz), 132.5,

130.2 (2C), 129.2, 128.3 (2C), 124.2 (C-F, $^{3}J_{C\text{-}F}$ = 8.4 Hz), 121.6, 119.1, 117.7 (C-F, $^{2}J_{C\text{-}F}$ = 22.2 Hz), 116.5, 113.6 (C-F, $^{2}J_{C\text{-}F}$ = 22.1 Hz), 18.2 (C-F, $^{4}J_{C\text{-}F}$ = 1.4 Hz). ^{19}F NMR (376 MHz, $CDCl_3$) δ -119.0 (m).
IR: 3361, 3058, 2922, 2853, 1650, 1596, 1579, 1491, 1447, 1320, 1268, 1148, 779, 716 cm^{-1}.
HRMS (ESI) m/z: $[M + Na]^+$ Calcd for $C_{20}H_{16}NOFNa$ 328.1108; Found 328.1096.

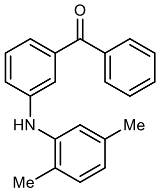

(3-((2,5-dimethylphenyl)amino)phenyl)(phenyl)methanone (12y). Prepared according to general procedure E and purified by flash column chromatography to afford the product as a yellow oil (47.4 mg, 63% yield). Eluant: ethyl acetate/petroleum ether (1:20, R_f = 0.30).
^{1}H NMR (400 MHz, $CDCl_3$) δ 7.88 – 7.77 (m, 2H), 7.61 – 7.53 (m, 1H), 7.52 – 7.43 (m, 2H), 7.38 – 7.29 (m, 2H), 7.27 – 7.22 (m, 1H), 7.16 – 7.03 (m, 3H), 6.84 – 6.75 (m, 1H), 5.49 (br, 1H), 2.28 (s, 3H), 2.21 (s, 3H). ^{13}C NMR (101 MHz, $CDCl_3$) δ 196.9, 144.7, 140.2, 138.9, 137.8, 136.7, 132.5, 131.1, 130.2 (2C), 129.3, 128.3 (2C), 126.3, 123.9, 121.9, 120.6, 120.5, 118.0, 21.3, 17.6.
IR: 3369, 3023, 2921, 2854, 1650, 1596, 1575, 1523, 1496, 1473, 1446, 1319, 1275, 1001, 983, 805, 779, 717, 701 cm^{-1}.
HRMS (ESI) m/z: $[M + Na]^+$ Calcd for $C_{21}H_{19}NONa$ 324.1359; Found 324.1366.

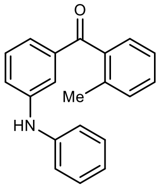

(3-(phenylamino)phenyl)(*o*-tolyl)methanone (13b). Prepared according to general procedure E and purified by flash column chromatography to afford the product as a yellow oil (52.4 mg, 73% yield). Eluant: ethyl acetate/petroleum ether (1:20, R_f = 0.30).
^{1}H NMR (400 MHz, $CDCl_3$) δ 7.52 – 7.47 (m, 1H), 7.42 – 7.21 (m, 9H), 7.13 – 7.04 (m, 2H), 7.01 – 6.92 (m, 1H), 5.86 (br, 1H), 2.34 (s, 3H). ^{13}C NMR (101 MHz, $CDCl_3$) δ 198.7, 143.8, 142.3, 139.1, 138.8, 136.9, 131.1, 130.4, 129.6 (2C), 129.5, 128.6, 125.2, 122.9, 121.9, 121.5, 118.5 (2C), 118.2, 20.1.
IR: 3361, 3056, 2954, 2923, 2853, 1654, 1589, 1515, 1494, 1484, 1451, 1307, 1263, 985, 738, 703 cm^{-1}.
HRMS (ESI) m/z: $[M + Na]^+$ Calcd for $C_{20}H_{17}NONa$ 310.1202; Found 310.1196.

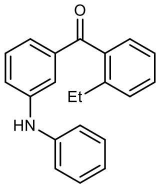

(2-ethylphenyl)(3-(phenylamino)phenyl)methanone (13c). Prepared according to general procedure E and purified by flash column chromatography to afford the product as a yellow oil (53.4 mg, 71% yield). Eluant: ethyl acetate/petroleum ether (1:20, R_f = 0.30).

^{1}H NMR (400 MHz, $CDCl_3$) δ 7.54 – 7.49 (m, 1H), 7.45 – 7.37 (m, 1H), 7.36 – 7.20 (m, 8H), 7.13 – 7.04 (m, 2H), 7.01 – 6.93 (m, 1H), 5.85 (br, 1H), 2.68 (q, *J* = 7.6 Hz, 2H), 1.17 (t, *J* = 7.5 Hz, 3H). ^{13}C NMR (101 MHz, $CDCl_3$) δ 198.8, 143.8, 143.1, 142.3, 139.2, 138.5, 130.4, 129.6 (2C), 129.5, 129.4, 128.4, 125.2, 123.0, 121.9, 121.6, 118.5 (2C), 118.2, 26.5, 16.1.
IR: 3363, 3059, 2961, 2871, 1654, 1589, 1515, 1484, 1452, 1307, 1265, 986, 747, 704 cm^{-1}.
HRMS (ESI) m/z: [M + Na]$^+$ Calcd for $C_{21}H_{19}NONa$ 324.1359; Found 324.1347.

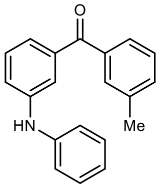

(3-(phenylamino)phenyl)(*m*-tolyl)methanone (13h). Prepared according to general procedure E and purified by flash column chromatography to afford the product as a yellow solid (42.3 mg, 59% yield). m.p. = 51 – 53 °C. Eluant: ethyl acetate/petroleum ether (1:20, R_f = 0.30).
^{1}H NMR (400 MHz, $CDCl_3$) δ 7.64 (s, 1H), 7.62 – 7.56 (m, 1H), 7.49 – 7.44 (m, 1H), 7.42 – 7.32 (m, 3H), 7.31 – 7.23 (m, 4H), 7.15 – 7.06 (m, 2H), 7.01 – 6.92 (m, 1H), 5.90 (br, 1H), 2.42 (s, 3H). ^{13}C NMR (101 MHz, $CDCl_3$) δ 197.1, 143.5, 142.4, 139.0, 138.2, 137.7, 133.4, 130.6, 129.6 (2C), 129.3, 128.2, 127.5, 122.6, 121.8, 120.9, 118.49, 118.46 (2C), 21.5.
IR: 3356, 3037, 2922, 2853, 1647, 1590, 1578, 1517, 1494, 1309, 1277, 992, 787, 739 cm^{-1}.
HRMS (ESI) m/z: [M + Na]$^+$ Calcd for $C_{20}H_{17}NONa$ 310.1202; Found 310.1211.

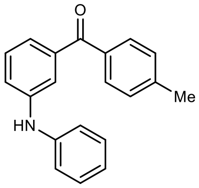

(3-(phenylamino)phenyl)(*p*-tolyl)methanone (13n). Prepared according to general procedure E and purified by flash column chromatography to afford the product as a yellow solid (40.9 mg, 57% yield). m.p. = 78 – 80 °C. Eluant: ethyl acetate/petroleum ether (1:20, R_f = 0.30).
^{1}H NMR (400 MHz, $CDCl_3$) δ 7.79 – 7.70 (m, 2H), 7.48 – 7.41 (m, 1H), 7.38 – 7.22 (m, 7H), 7.15 – 7.06 (m, 2H), 7.01 – 6.92 (m, 1H), 5.88 (br, 1H), 2.43 (s, 3H). ^{13}C NMR (101 MHz, $CDCl_3$) δ 196.6, 143.5, 143.4, 142.4, 139.3, 134.9, 130.4 (2C), 129.6 (2C), 129.2, 129.1 (2C), 122.5, 121.8, 120.7, 118.5 (2C), 118.4, 21.8.
IR: 3352, 3032, 2922, 2852, 1645, 1590, 1579, 1494, 1313, 1275, 1179, 834, 791, 744 cm^{-1}.
HRMS (ESI) m/z: [M + Na]$^+$ Calcd for $C_{20}H_{17}NONa$ 310.1202; Found 310.1207.

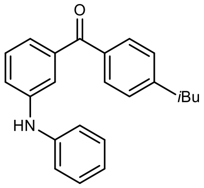

(4-isobutylphenyl)(3-(phenylamino)phenyl)methanone (13o). Prepared according to general procedure E and purified by flash column chromatography to afford the product as a yellow solid (50.2 mg, 61% yield). m.p. = 76 – 78 °C. Eluant: ethyl acetate/petroleum ether (1:20, R_f = 0.30).
^{1}H NMR (400 MHz, $CDCl_3$) δ 7.82 – 7.71 (m, 2H), 7.47 – 7.43 (m, 1H), 7.37 – 7.21 (m, 7H), 7.15 – 7.06 (m, 2H), 7.00 – 6.92 (m, 1H), 5.88 (br, 1H), 2.55 (d, *J* = 7.2 Hz, 2H), 2.00 – 1.83 (m, 1H), 0.93 (d, *J* = 6.6 Hz, 6H). ^{13}C NMR (101 MHz, $CDCl_3$) δ 196.7, 147.1, 143.6, 142.4, 139.3,

135.2, 130.3 (2C), 129.6 (2C), 129.2, 129.1 (2C), 122.5, 121.8, 120.7, 118.5 (2C), 118.4, 45.5, 30.3, 22.5 (2C).
IR: 3357, 3049, 2954, 2867, 1647, 1591, 1580, 1495, 1313, 1264, 1180, 986, 790, 735 cm^{-1}.
HRMS (ESI) m/z: $[M + Na]^+$ Calcd for $C_{23}H_{23}NONa$ 352.1672; Found 352.1684.

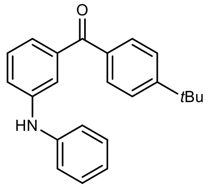

(4-(*tert*-butyl)phenyl)(3-(phenylamino)phenyl)methanone (13p). Prepared according to general procedure E and purified by flash column chromatography to afford the product as a yellow solid (51.0 mg, 62% yield). m.p. = 68 – 70 °C. Eluant: ethyl acetate/petroleum ether (1:20, R_f = 0.30).
^{1}H NMR (400 MHz, $CDCl_3$) δ 7.82 – 7.74 (m, 2H), 7.52 – 7.47 (m, 2H), 7.47 – 7.43 (m, 1H), 7.36 – 7.25 (m, 5H), 7.14 – 7.07 (m, 2H), 7.00 – 6.92 (m, 1H), 5.91 (br, 1H), 1.36 (s, 9H). ^{13}C NMR (101 MHz, $CDCl_3$) δ 196.6, 156.3, 143.5, 142.4, 139.3, 134.9, 130.3 (2C), 129.6 (2C), 129.2, 125.3 (2C), 122.6, 121.8, 120.7, 118.5, 118.4 (2C), 35.2, 31.3 (3C).
IR: 3355, 3037, 2959, 2867, 1645, 1591, 1518, 1495, 1451, 1315, 1266, 798, 739, 716 cm^{-1}.
HRMS (ESI) m/z: $[M + Na]^+$ Calcd for $C_{23}H_{23}NONa$ 352.1672; Found 352.1664.

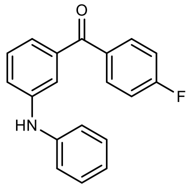

(4-fluorophenyl)(3-(phenylamino)phenyl)methanone (13r). Prepared according to general procedure E and purified by flash column chromatography to afford the product as a yellow solid (45.1 mg, 62% yield). m.p. = 53 – 55 °C. Eluant: ethyl acetate/petroleum ether (1:15, R_f = 0.30).
^{1}H NMR (400 MHz, $CDCl_3$) δ 7.91 – 7.82 (m, 2H), 7.45 – 7.40 (m, 1H), 7.38 – 7.20 (m, 5H), 7.19 – 7.07 (m, 4H), 7.01 – 6.94 (m, 1H), 5.89 (br, 1H). ^{13}C NMR (101 MHz, $CDCl_3$) δ 195.4, 165.5 (C-F, $^1J_{C-F}$ = 254.3 Hz), 143.8, 142.2, 138.8, 133.9 (C-F, $^4J_{C-F}$ = 3.3 Hz), 132.8 (C-F, $^3J_{C-F}$ = 9.2 Hz, 2C), 129.6 (2C), 129.4, 122.3, 122.0, 120.9, 118.7 (2C), 118.1, 115.5 (C-F, $^2J_{C-F}$ = 21.8 Hz, 2C). ^{19}F NMR (376 MHz, $CDCl_3$) δ -105.8 (m).
IR: 3360, 3059, 2953, 2923, 2853, 1649, 1590, 1579, 1494, 1451, 1410, 1310, 1274, 1226, 1154, 987, 750, 699 cm^{-1}.
HRMS (ESI) m/z: $[M + Na]^+$ Calcd for $C_{19}H_{14}NOFNa$ 314.0952; Found 314.0941.

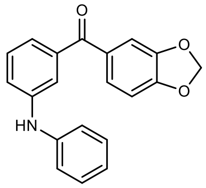

benzo[*d*][1,3]dioxol-5-yl(3-(phenylamino)phenyl)methanone (13w). Prepared according to general procedure E and purified by flash column chromatography to afford the product as a yellow oil (45.2 mg, 57% yield). Eluant: ethyl acetate/petroleum ether (1:10, R_f = 0.30).
^{1}H NMR (400 MHz, $CDCl_3$) δ 7.45 – 7.36 (m, 3H), 7.36 – 7.20 (m, 5H), 7.16 – 7.07 (m, 2H), 7.01 – 6.93 (m, 1H), 6.86 (d, J = 8.1 Hz, 1H), 6.07 (s, 2H), 5.86 (br, 1H). ^{13}C NMR (101 MHz,

$CDCl_3$) δ 195.3, 151.7, 148.0, 143.5, 142.4, 139.5, 132.0, 129.6 (2C), 129.2, 127.0, 122.2, 121.9, 120.6, 118.6 (2C), 118.2, 110.0, 107.8, 102.0.
IR: 3351, 3055, 2922, 2852, 1644, 1590, 1579, 1495, 1483, 1437, 1257, 1035, 748, 721 cm^{-1}.
HRMS (ESI) m/z: $[M + Na]^+$ Calcd for $C_{20}H_{15}NO_3Na$ 340.0944; Found 340.0936.

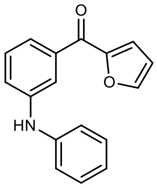

furan-2-yl(3-(phenylamino)phenyl)methanone (13z). Prepared according to general procedure E and purified by flash column chromatography to afford the product as a yellow oil (38.1 mg, 58% yield). Eluant: ethyl acetate/petroleum ether (1:20, R_f = 0.30).
^{1}H NMR (400 MHz, $CDCl_3$) δ 7.72 – 7.67 (m, 1H), 7.63 – 7.58 (m, 1H), 7.51 – 7.45 (m, 1H), 7.40 – 7.33 (m, 1H), 7.33 – 7.26 (m, 3H), 7.24 (d, J = 3.5 Hz, 1H), 7.15 – 7.08 (m, 2H), 7.02 – 6.95 (m, 1H), 6.61 – 6.56 (m, 1H), 5.88 (br, 1H). ^{13}C NMR (101 MHz, $CDCl_3$) δ 182.7, 152.4, 147.3, 143.8, 142.4, 138.6, 129.6 (2C), 129.5, 122.0, 121.7, 121.2, 120.7, 118.7 (2C), 117.8, 112.3.
IR: 3346, 3054, 2954, 2922, 2852, 1634, 1591, 1578, 1494, 1460, 1307, 1171, 1026, 744 cm^{-1}.
HRMS (ESI) m/z: $[M + Na]^+$ Calcd for $C_{17}H_{13}NO_2Na$ 286.0838; Found 286.0845.

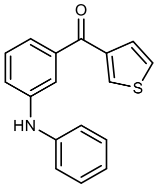

(3-(phenylamino)phenyl)(thiophen-3-yl)methanone (13aa). Prepared according to general procedure E and purified by flash column chromatography to afford the product as a yellow oil (37.0 mg, 53% yield). Eluant: ethyl acetate/petroleum ether (1:20, R_f = 0.30).
^{1}H NMR (400 MHz, $CDCl_3$) δ 7.99 – 7.93 (m, 1H), 7.60 (d, J = 5.1 Hz, 1H), 7.53 – 7.46 (m, 1H), 7.43 – 7.33 (m, 3H), 7.32 – 7.27 (m, 3H), 7.17 – 7.07 (m, 2H), 7.03 – 6.94 (m, 1H), 5.89 (br, 1H). ^{13}C NMR (101 MHz, $CDCl_3$) δ 190.1, 143.8, 142.3, 141.4, 140.0, 134.2, 129.6 (2C), 129.4, 128.7, 126.3, 122.0, 121.8, 120.8, 118.7 (2C), 117.7.
IR: 3352, 3054, 2921, 2851, 1635, 1590, 1578, 1509, 1494, 1451, 1412, 1265, 790, 740 cm^{-1}.
HRMS (ESI) m/z: $[M + Na]^+$ Calcd for $C_{17}H_{13}NOSNa$ 302.0610; Found 302.0619.

5. Preliminary Mechanistic Studies

5.1 ^{18}O-labeled water experiment

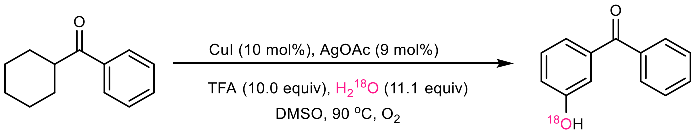

A 15 mL sealed tube containing a magnetic stir bar was charged with ketone **1a** (47.0 mg, 0.25 mmol), CuI (4.8 mg, 10 mol%), AgOAc (3.7 mg, 9 mol%) and DMSO (1 mL) sequentially. The tube was evacuated and backfilled with O_2 three times. Subsequently, $H_2{}^{18}O$ (50 uL) and TFA (186 uL, 10.0 equiv) was added. The tube was sealed and the mixture was stirred at 90 °C for 60 h. After cooling to room temperature, the reaction mixture was diluted with EtOAc (50 mL) and washed with H_2O (3 × 1 mL) and brine (3 × 1 mL). The combined organic layer was dried over anhydrous Na_2SO_4, and concentrated to give the residue, which was further purified by silica gel column chromatography to afford the desired product. HRMS (ESI) m/z: $[M + Na]^+$ Calcd for $C_{13}H_{10}O^{18}ONa$ 223.0615; Found 223.0610.

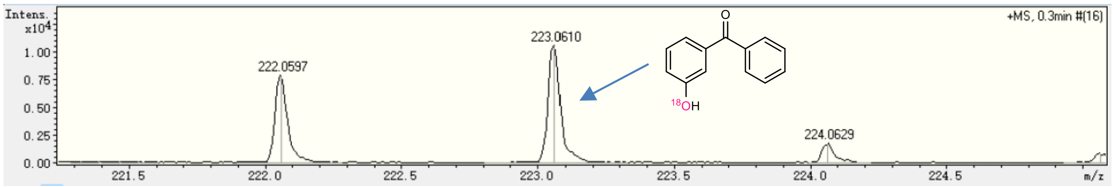

Supplementary Figure 5. HRMS spectrum of the ^{18}O Compound

5.2 Deuterium-labeling experiments

5.2.1 H/D exchange experiments–investigations of *γ*-C−H activation

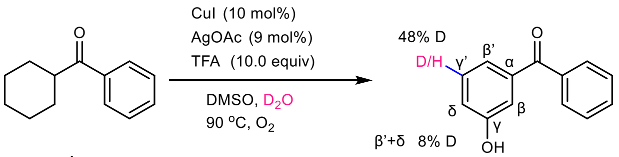

A 15 mL sealed tube containing a magnetic stir bar was charged with ketone **1a** (47.0 mg, 0.25 mmol), CuI (4.8 mg, 10 mol%), AgOAc (3.7 mg, 9 mol%) and DMSO (1 mL) sequentially. The tube was evacuated and backfilled with O_2 three times. Subsequently, D_2O (50 uL) and TFA (186 uL, 10.0 equiv) was added. The tube was sealed and the mixture was stirred at 90 °C for 60 h. After cooling to room temperature, the reaction mixture was diluted with EtOAc (50 mL) and washed with H_2O (3 × 1 mL) and brine (3 × 1 mL). The combined organic layer was dried over anhydrous Na_2SO_4, and concentrated to give the residue, which was further purified by silica gel column chromatography to afford the deuterium-labeling product which was characterized by ^{1}H NMR spectroscopy. The deuterium-labeling product was found to contain 48% D incorporation at the *γ′* position.

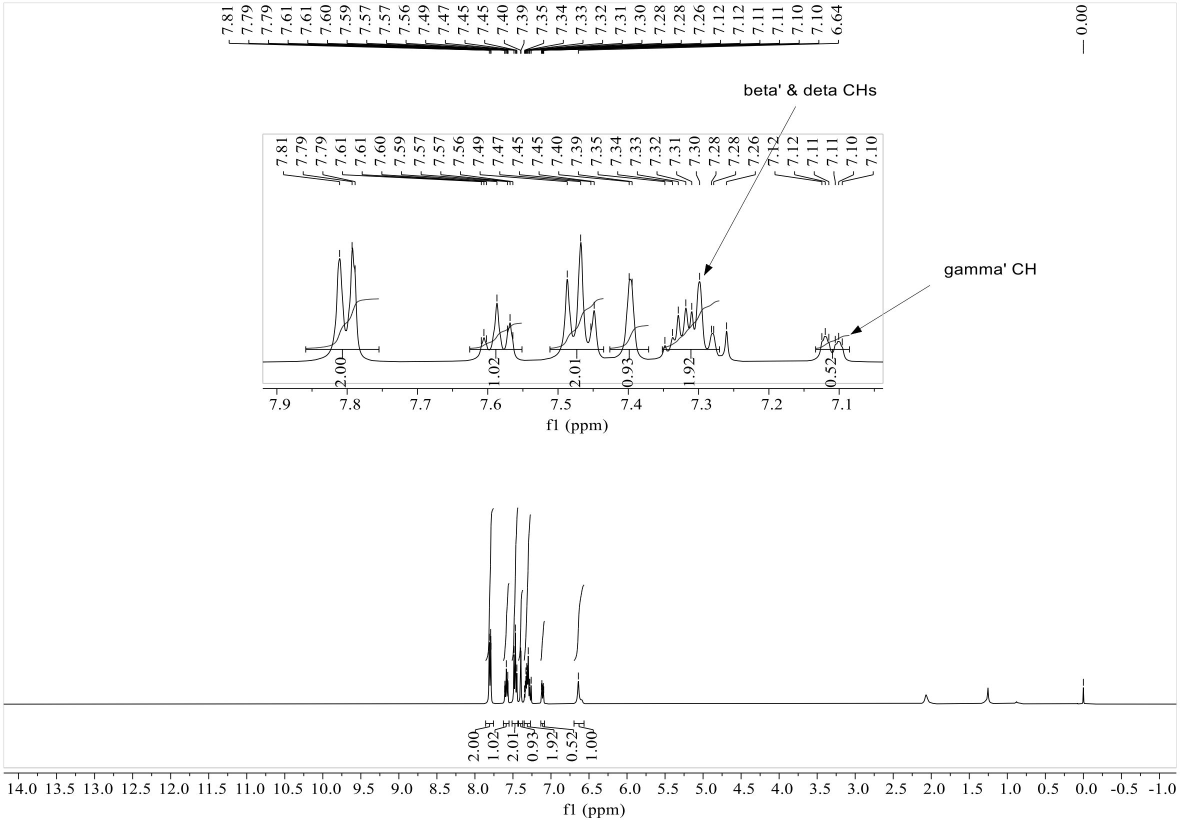

Supplementary Figure 6. ^{1}H NMR spectrum of compound deuterium-labeling **2a** (400 MHz, $CDCl_3$)

5.2.2 H/D exchange experiments – investigations of *α*-C−H activation

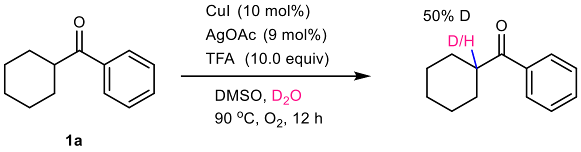

A 15 mL sealed tube containing a magnetic stir bar was charged with ketone **1a** (47.0 mg, 0.25 mmol), CuI (4.8 mg, 10 mol%), AgOAc (3.7 mg, 9 mol%) and DMSO (1 mL) sequentially. The tube was evacuated and backfilled with O_2 three times. Subsequently, D_2O (50 uL) and TFA (186 uL, 10.0 equiv) was added. The tube was sealed and the mixture was stirred at 90 °C for 12 h. After cooling to room temperature, the reaction mixture was diluted with EtOAc (50 mL) and washed with H_2O (3 × 1 mL) and brine (3 × 1 mL). The combined organic layer was dried over anhydrous Na_2SO_4, and concentrated to give the residue, which was further purified by silica gel column chromatography to afford the recovered **1a** which was characterized by ^{1}H NMR spectroscopy. The recovered **1a** was found to contain 50% D at the *α* position.

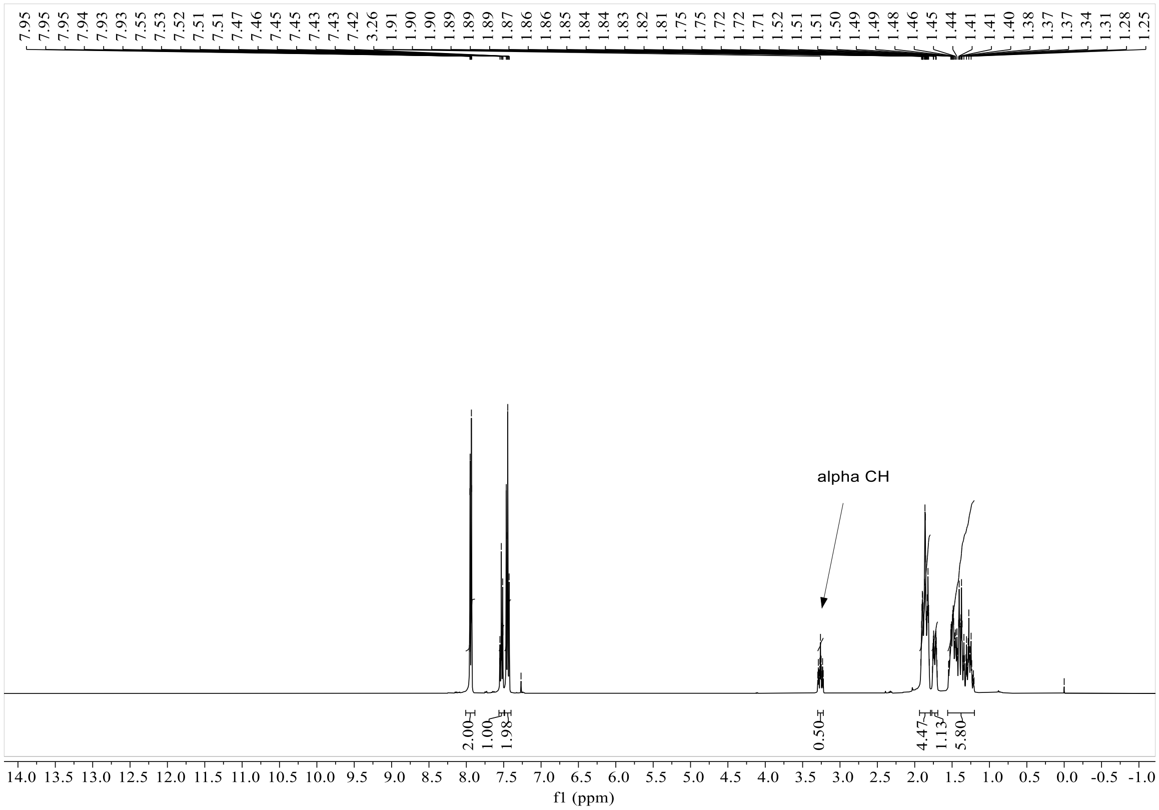

Supplementary Figure 7. ^{1}H NMR spectrum of the recovered **1a** (400 MHz, $CDCl_3$)

5.3 KIE experiments

Deuterated-substratep preparation: Synthesis of **(cyclohexyl-1-*d*)(phenyl)methanone (1a-*d*)**.[23]

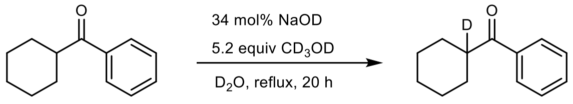

(cyclohexyl-1-*d*)(phenyl)methanone (1a-*d*). An oven-dried 50 mL round bottom flask was placed 8.5 mL D_2O, cyclohexyl(phenyl)methanone (1.88 g, 10.0 mmol), CD_3OD (1.82 g, 52.0 mmol) and a solution of 40% NaOD in D_2O (0.3 mL, 3.4 mmol) under argon atmosphere. The reaction mixture was stirred at reflux for 20 h. After cooling to room temperature, 8 mL diethyl ether was added via syringe. After stirring for 1 h, the layers were separated and the aqueous layer was washed with ether (2 × 20 mL). The combined organic layers were washed with water (2 × 20 mL) and brine (2 × 20 mL), dried over Na_2SO_4 and concentrated by evaporation. The crude product was shown to be 98% deuterated material.

^{1}H NMR (400 MHz, $CDCl_3$) δ 8.00 – 7.89 (m, 2H), 7.58 – 7.51 (m, 1H), 7.50 – 7.41 (m, 2H), 1.96 – 1.80 (m, 4H), 1.79 – 1.66 (m, 1H), 1.58 – 1.19 (m, 5H). ^{13}C NMR (101 MHz, $CDCl_3$) δ 204.1, 136.4, 132.9, 128.7 (2C), 128.4 (2C), 45.3 (t, *J* = 19.3 Hz), 29.4 (2C), 26.1, 26.0 (2C).

HRMS (ESI) m/z: $[M + Na]^+$ Calcd for $C_{13}H_{15}DONa$ 212.1156; Found 212.1148.

IR: 2928, 2856, 1666, 1595, 1578, 1444, 1286, 1251, 1180, 980, 763, 699 cm^{-1}.

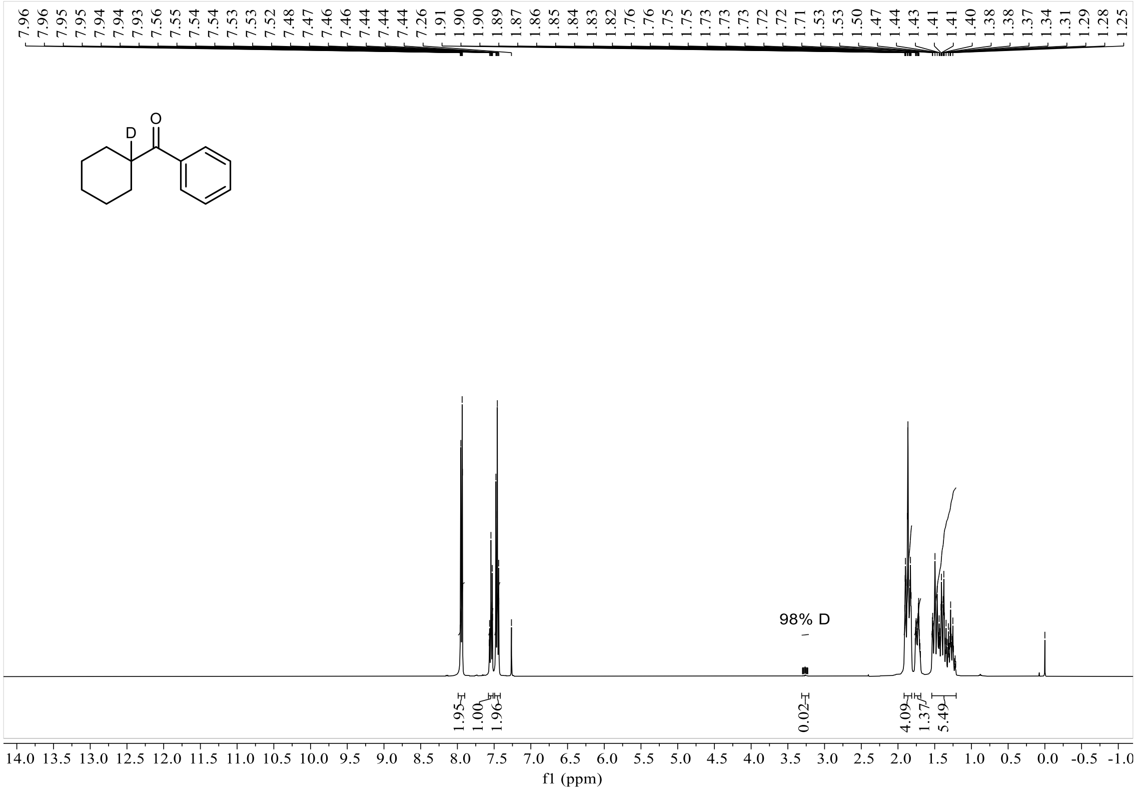

Supplementary Figure 8. ^{1}H NMR spectrum of compound **1a**-*d* (400 MHz, $CDCl_3$)

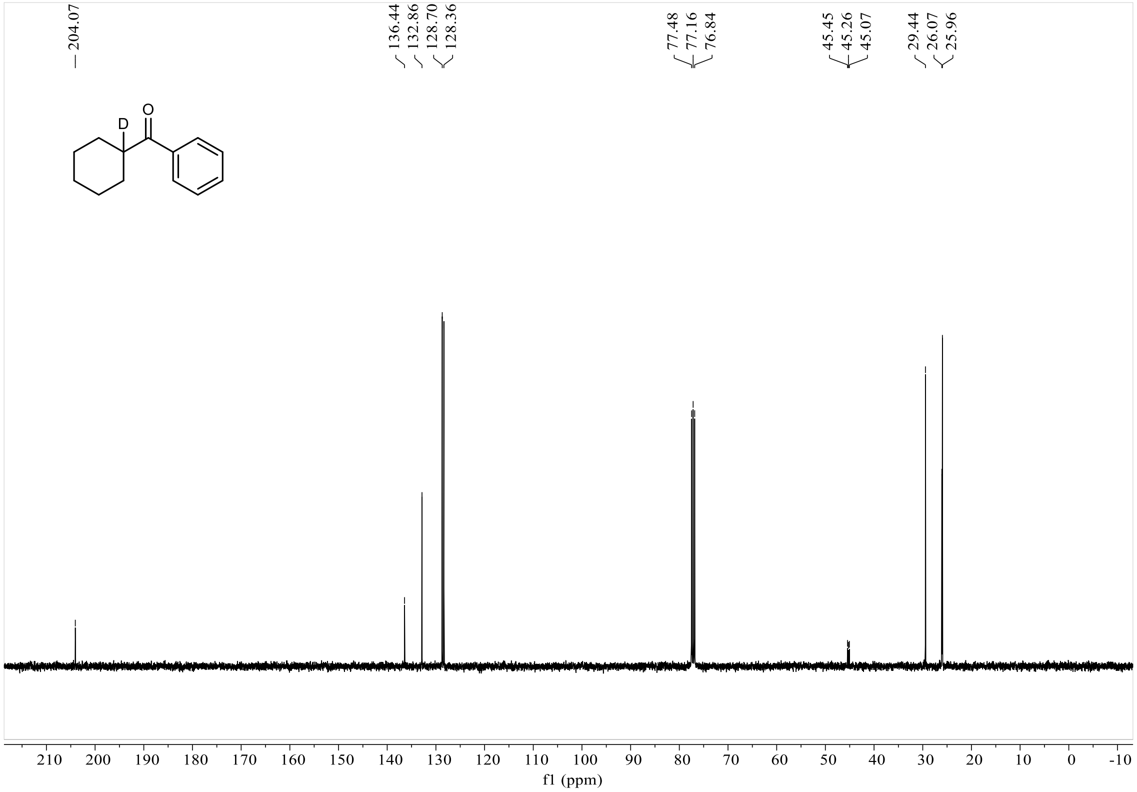

Supplementary Figure 9. ^{13}C NMR spectrum of compound **1a**-*d* (101 MHz, $CDCl_3$)

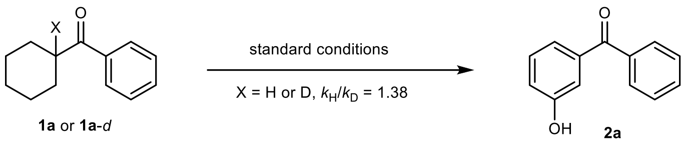

KIE experiment: We use **1a** and deuterated **1a**-*d* as starting materials and two parallel reactions were carried out. A 15 mL sealed tube containing a magnetic stir bar was charged with **1a** (0.25 mmol) or **1a**-*d* (0.25 mmol), CuI (4.8 mg, 10 mol%), AgOAc (3.7 mg, 9 mol%) and DMSO (1 mL) sequentially. The tubes were evacuated and backfilled with O_2 three times. Subsequently, H_2O (50 uL, 11.1 equiv) and TFA (186 uL, 10.0 equiv) was added. The tubes were sealed and the mixture was stirred at 90 °C. A 200 uL reaction mixture was taken at 5 h, 7 h, 8 h, 9 h, 12 h. ^{1}H NMR was taken to determine the amount of **2a** using dibromomethane as the internal standard. The obtained yields were plotted as concentration vs. time and the following initial rates were calculated.

Supplementary Table 13. Conversion of the reaction of **1a** and **1a**-*d*

Reaction time (h)	H-Concentration (M)	D-Concentration (M)
5	0.0015	0.0015
7	0.00375	0.00375
8	0.0055	0.00475
9	0.00625	0.00525
12	0.00925	0.00725

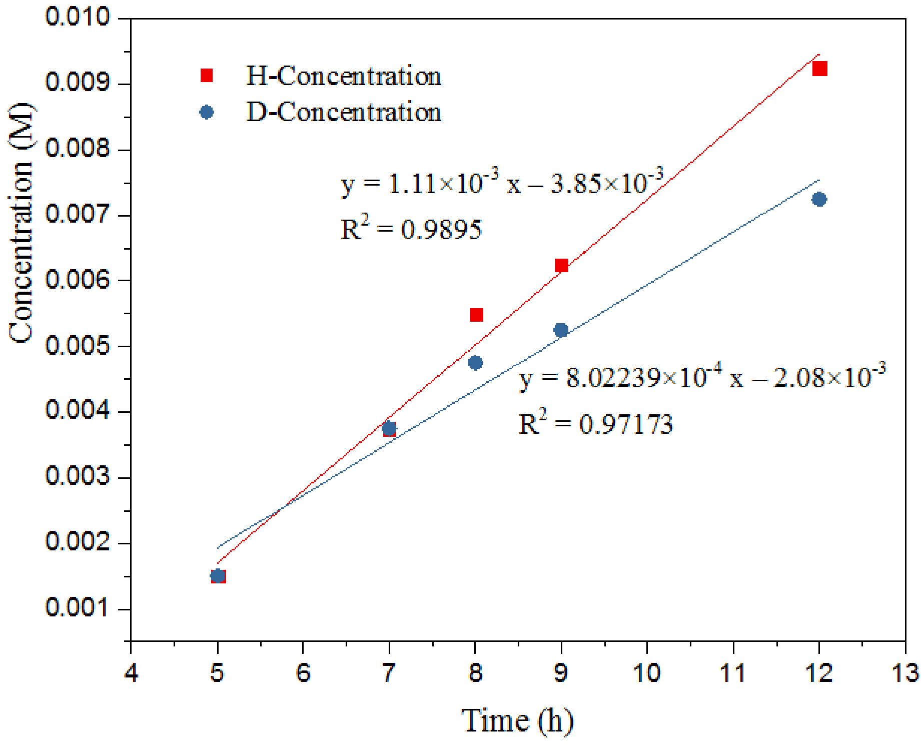

Supplementary Figure 10. Concentration versus time

5.4 Intermediate traping experiments and kinetic profiles

5.4.1 Intermediate traping experiments

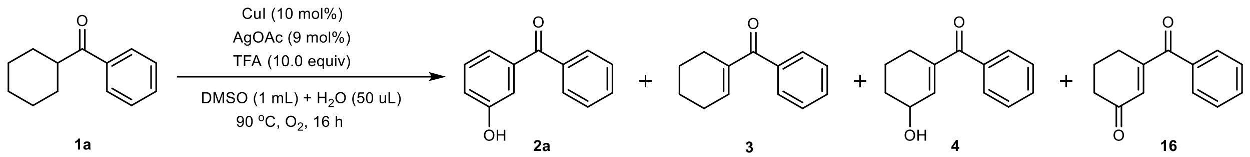

A 15 mL sealed tube containing a magnetic stir bar was charged with ketone **1a** (47.0 mg, 0.25 mmol), CuI (4.8 mg, 10 mol%), AgOAc (3.7 mg, 9 mol%) and DMSO (1 mL) sequentially. The tube was evacuated and backfilled with O_2 three times. Subsequently, H_2O (50 uL, 11.1 equiv) and TFA (186 uL, 10.0 equiv) was added. The tube was sealed and the mixture was stirred at 90 °C for 16 h. A 200 uL reaction mixture was taken and immediately diluted with internal standard CH_2Br_2 (1 uL) in DMSO-d_6 (400 uL). The reaction mixture was found to contain compounds **2a**, **3**, **4** and **16**. The desired product **2a**, compounds **3**, **4**, and **16** were also isolated.

3: ^{1}H NMR (400 MHz, $CDCl_3$) δ 7.65 – 7.60 (m, 2H), 7.52 – 7.46 (m, 1H), 7.44 – 7.38 (m, 2H), 6.61 – 6.56 (m, 1H), 2.46 – 2.39 (m, 2H), 2.30 – 2.23 (m, 2H), 1.78 – 1.63 (m, 4H). ^{13}C NMR (101 MHz, $CDCl_3$) δ 198.4, 144.3, 138.8 (2C), 131.4, 129.3 (2C), 128.1 (2C), 26.2, 24.1, 22.1, 21.8.
IR: 3058, 2929, 2858, 1643, 1598, 1577, 1446, 1276, 1255, 700 cm^{-1}.
HRMS (ESI) m/z: $[M + Na]^+$ Calcd for $C_{13}H_{14}ONa$ 209.0937; Found 209.0945.

4: ^{1}H NMR (400 MHz, $CDCl_3$) δ 7.71 – 7.63 (m, 2H), 7.55 – 7.47 (m, 1H), 7.46 – 7.37 (m, 2H), 6.45 – 6.40 (m, 1H), 4.47 – 4.38 (m, 1H), 2.48 – 2.24 (m, 3H), 2.06 – 1.82 (m, 2H), 1.75 – 1.56 (m, 2H). ^{13}C NMR (101 MHz, $CDCl_3$) δ 198.4, 142.8, 140.0, 137.8, 132.1, 129.4 (2C), 128.3 (2C), 66.3, 31.4, 24.3, 19.2.
IR: 3392, 3059, 2931, 2861, 1633, 1597, 1576, 1446, 1303, 1265, 1243, 1123, 958, 750 cm^{-1}.
HRMS (ESI) m/z: $[M + H]^+$ Calcd for $C_{13}H_{15}O_2$ 203.1067; Found 203.1076.

16: ^{1}H NMR (400 MHz, $CDCl_3$) δ 7.83 – 7.76 (m, 2H), 7.65 – 7.58 (m, 1H), 7.53 – 7.45 (m, 2H), 6.27 (t, J = 1.8 Hz, 1H), 2.71 (td, J = 6.0, 1.8 Hz, 2H), 2.59 – 2.51 (m, 2H), 2.22 – 2.13 (m, 2H). ^{13}C NMR (101 MHz, $CDCl_3$) δ 200.3, 197.2, 156.0, 135.7, 133.6, 132.6, 129.8 (2C), 128.8 (2C), 38.0, 25.7, 22.4.
IR: 3057, 2922, 2850, 1677, 1655, 1597, 1448, 1254, 1233, 964, 733, 704 cm^{-1}.
HRMS (ESI) m/z: $[M + Na]^+$ Calcd for $C_{13}H_{12}O_2Na$ 223.0730; Found 223.0735.

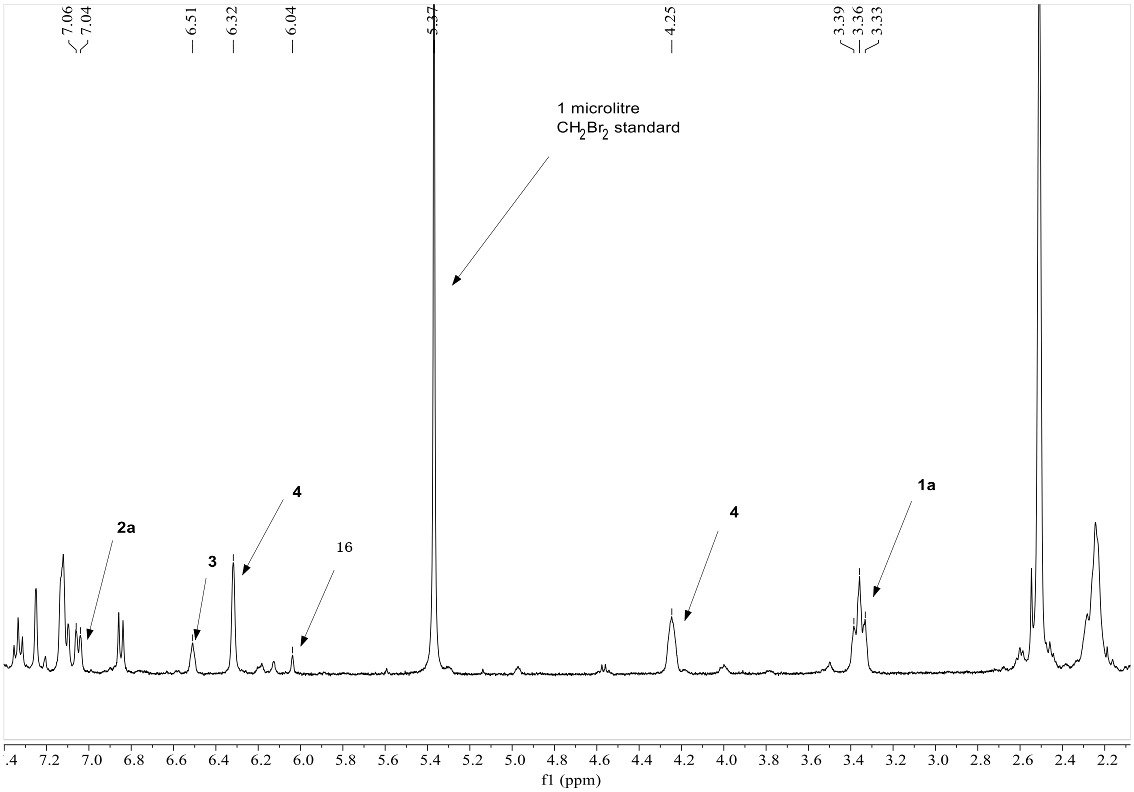

Supplementary Figure 11. Intermediate traping experiments

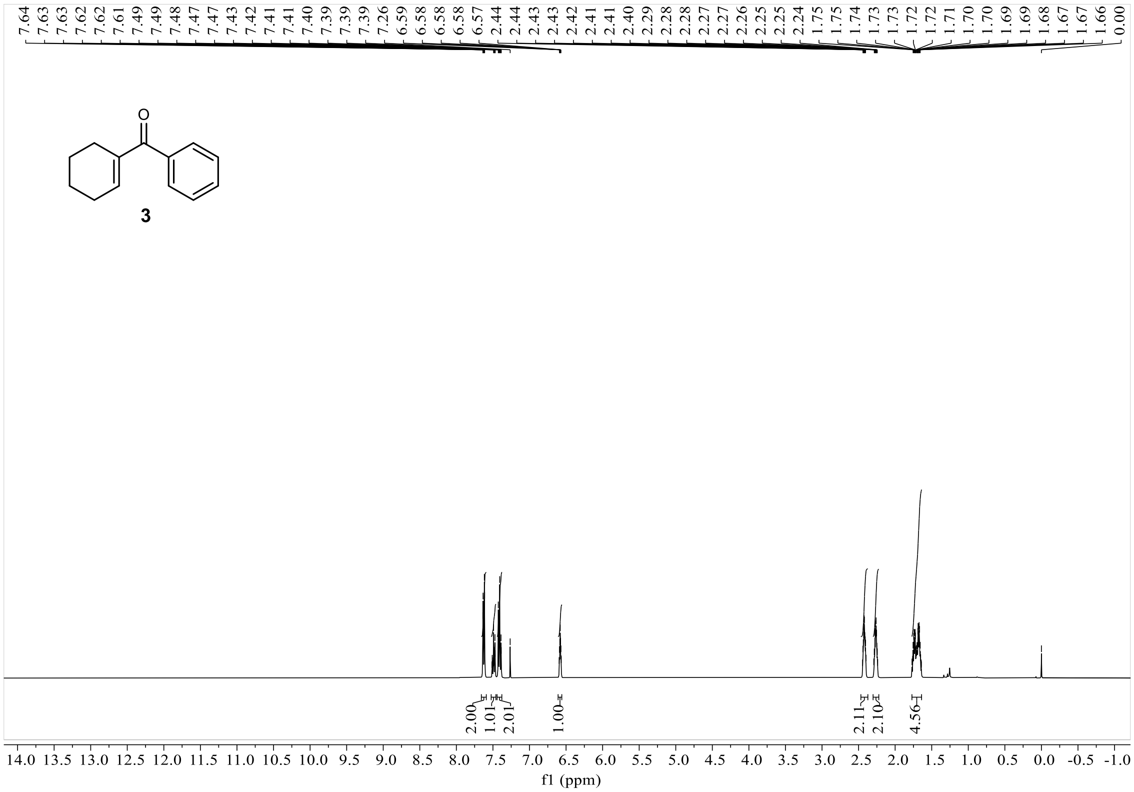

Supplementary Figure 12. ^{1}H NMR spectrum of compound **3** (400 MHz, $CDCl_3$)

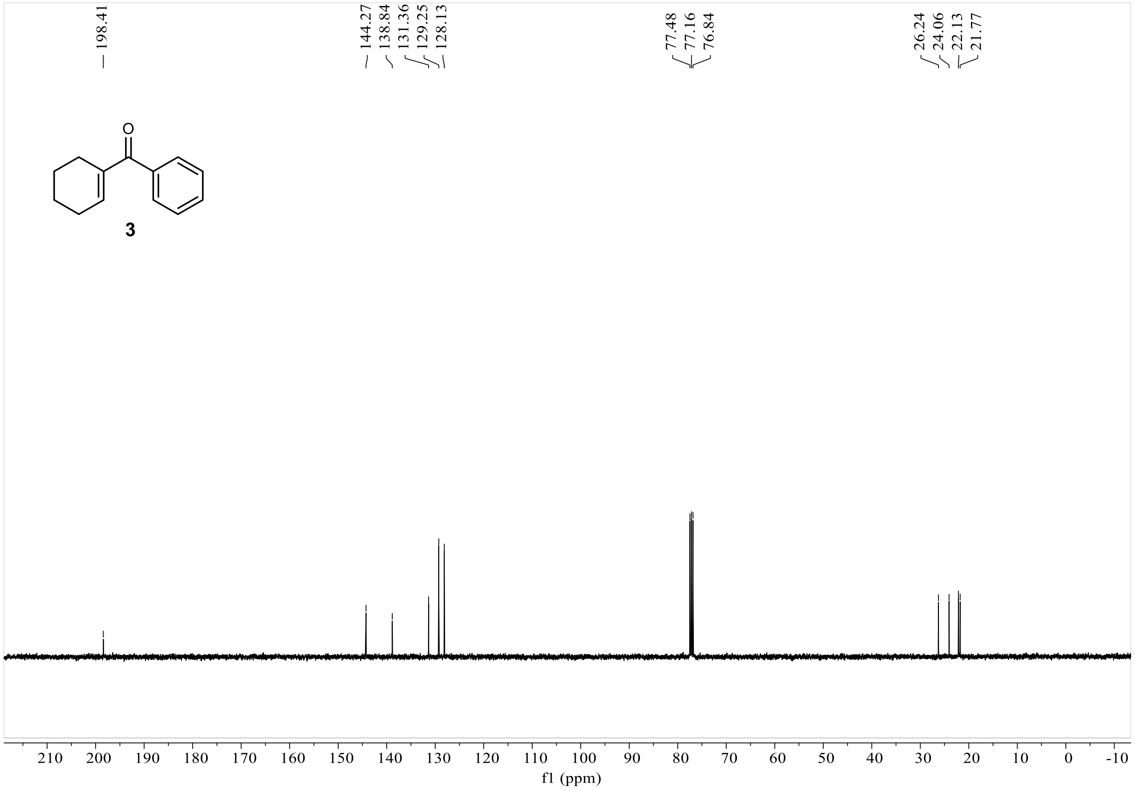

Supplementary Figure 13. ^{13}C NMR spectrum of compound **3** (101 MHz, $CDCl_3$)

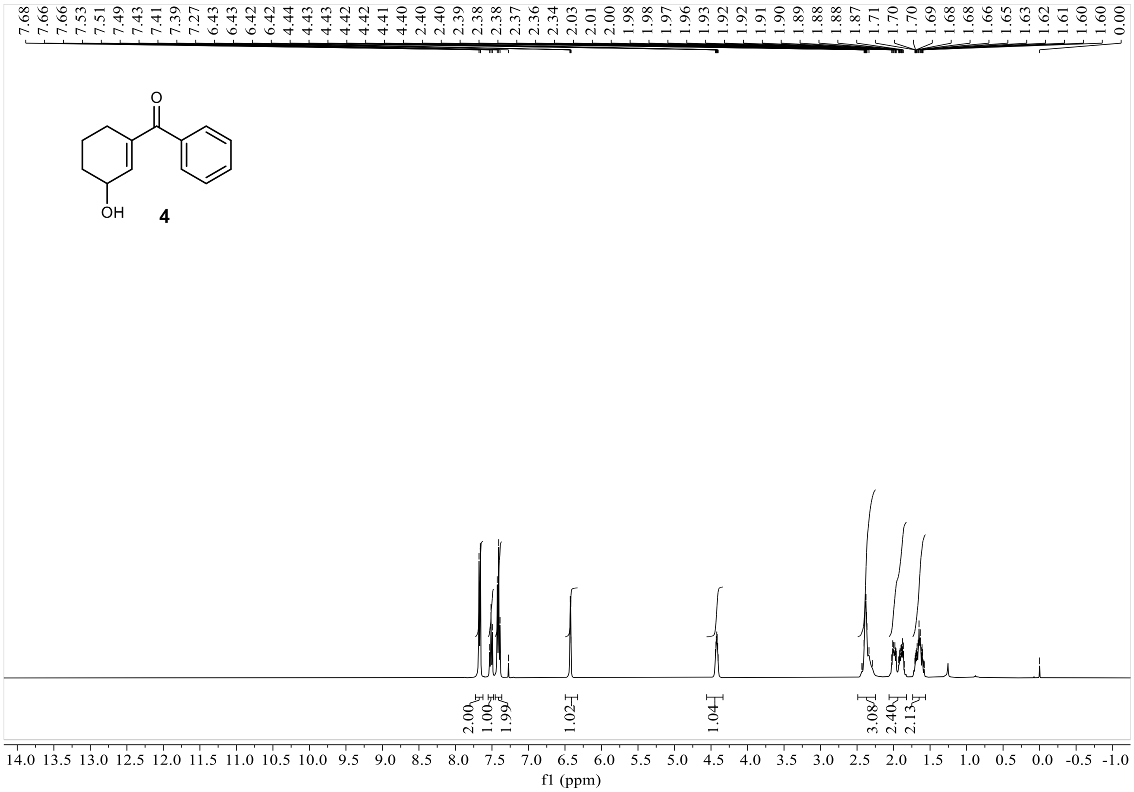

Supplementary Figure 14. ^{1}H NMR spectrum of compound **4** (400 MHz, $CDCl_3$)

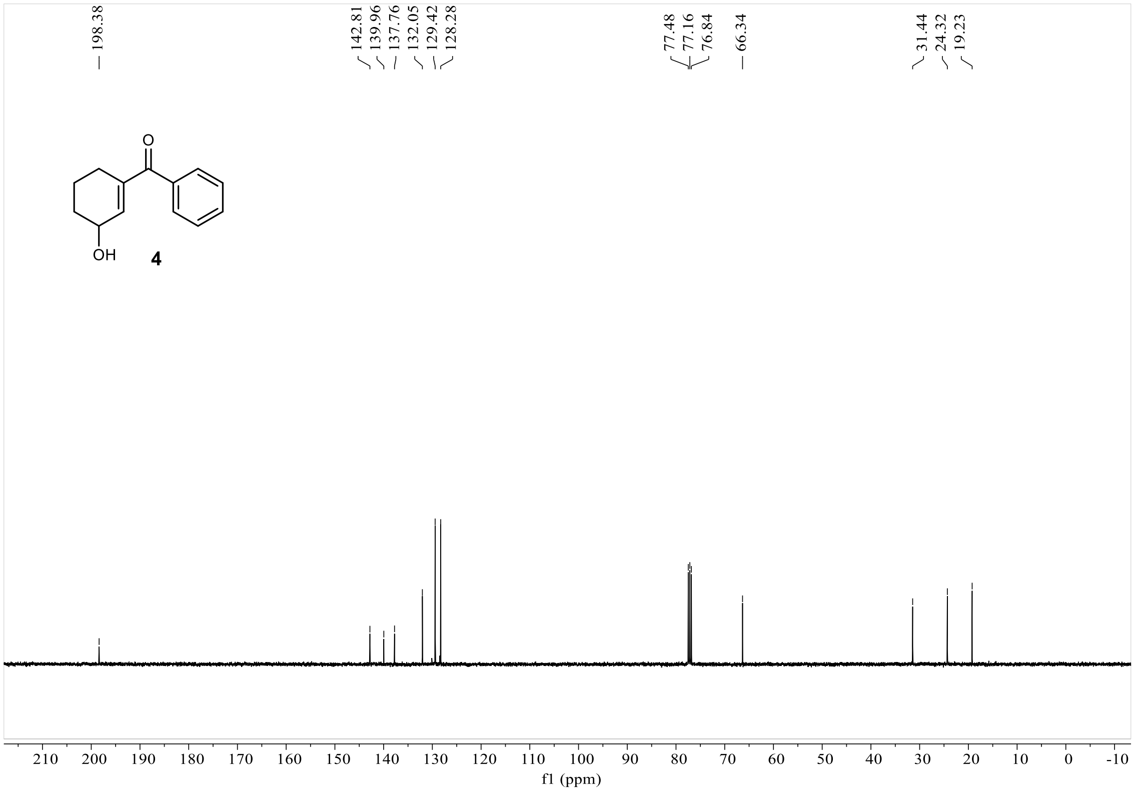

Supplementary Figure 15. ^{13}C NMR spectrum of compound **4** (101 MHz, $CDCl_3$)

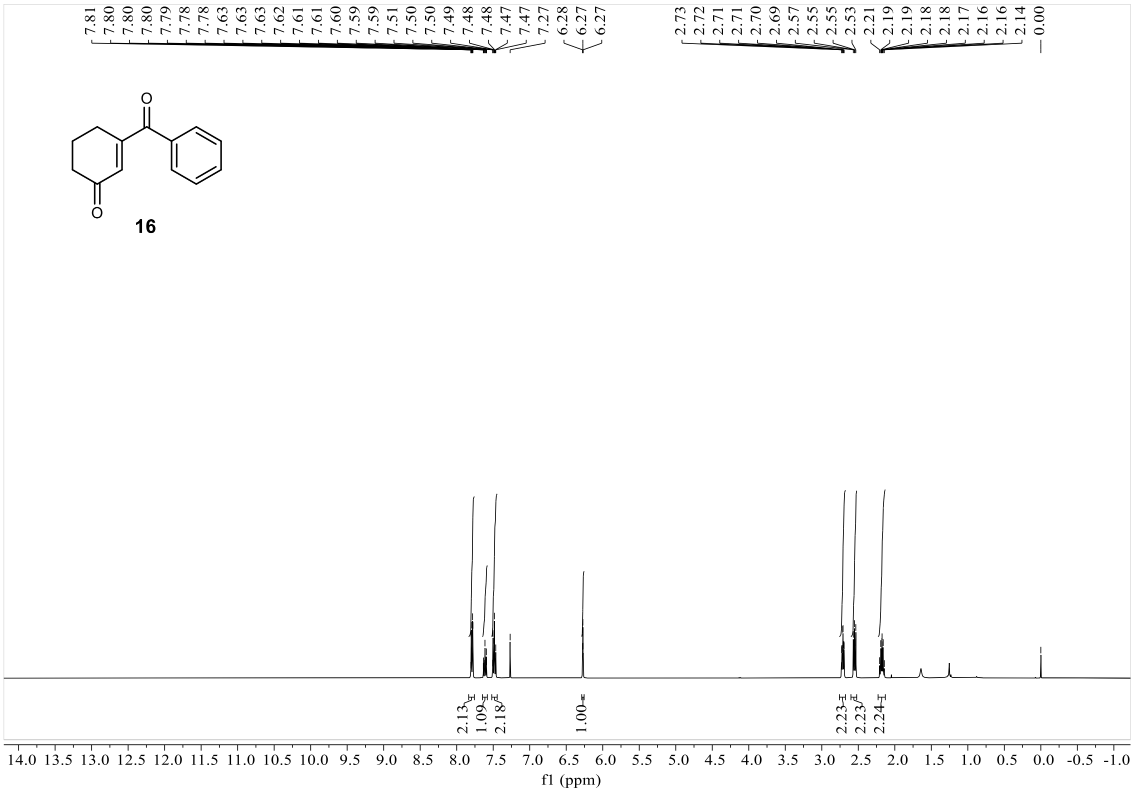

Supplementary Figure 16. ^{1}H NMR spectrum of compound **16** (400 MHz, $CDCl_3$)

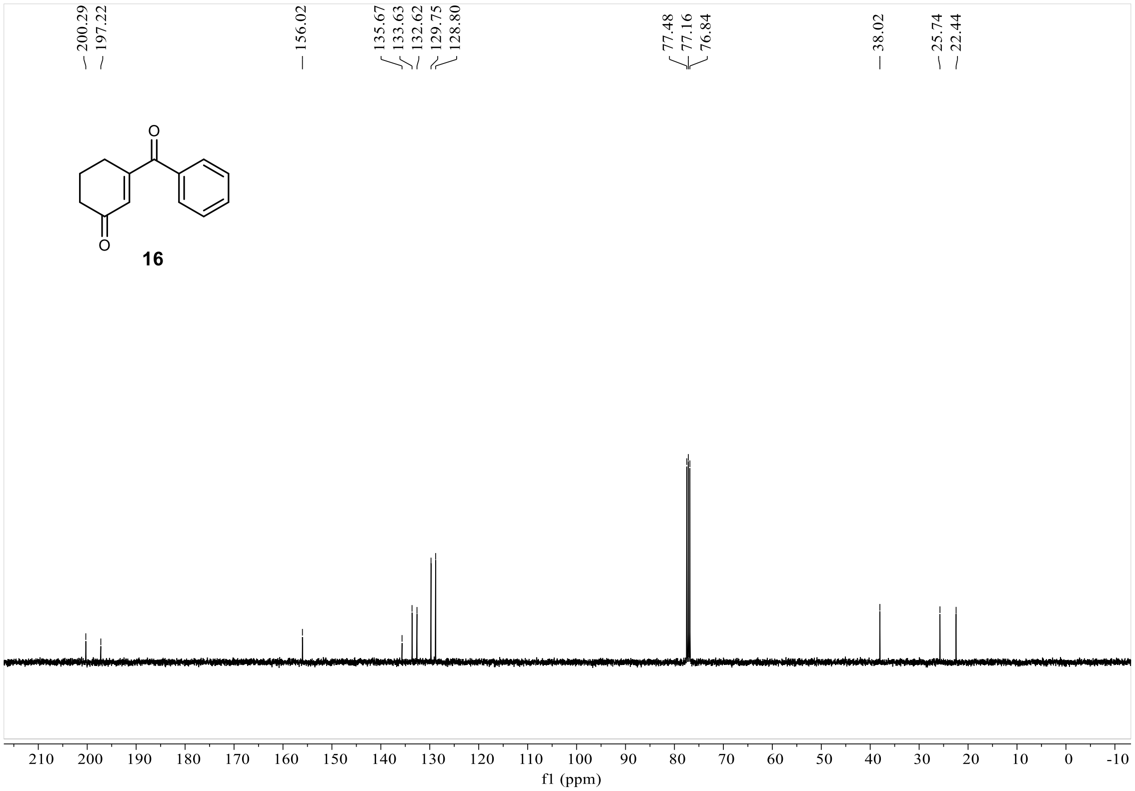

Supplementary Figure 17. ^{13}C NMR spectrum of compound **16** (101 MHz, $CDCl_3$)

5.4.2 Kinetic profiles

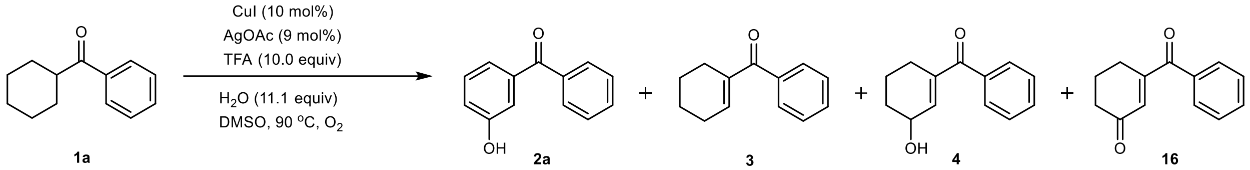

A 15 mL sealed tube containing a magnetic stir bar was charged with ketones (47.0 mg, 0.25 mmol), CuI (4.8 mg, 10 mol%), AgOAc (3.7 mg, 9 mol%) and DMSO (1 mL) sequentially. The tube was evacuated and backfilled with O_2 three times. Subsequently, H_2O (50 uL, 11.1 equiv) and TFA (186 uL, 10.0 equiv) was added. The tube was sealed and the mixture was stirred at 90 °C. The reaction aliquot was periodically sampled and analyzed by ^1H NMR.

Supplementary Table 14. Concentration of **1a**, **2a**, **3**, **4** and **16**.

Time (h)	Concentration (M)				
	**1a**	**2a**	**3**	**4**	**16**
0	0.25	0	0	0	0
1	0.213		0.01175		
2			0.01675	0.00625	
3	0.176		0.02025	0.01275	
4	0.167		0.02225		

5			0.0215		
6	0.14	0.003	0.01975	0.02575	0.00325
12	0.095	0.01225	0.016	0.035	0.0045
16	0.076	0.026	0.01425	0.0415	0.00525
20	0.062	0.0365	0.012	0.0395	0.005
60	0.0025	0.19	0.00525	0.00375	0

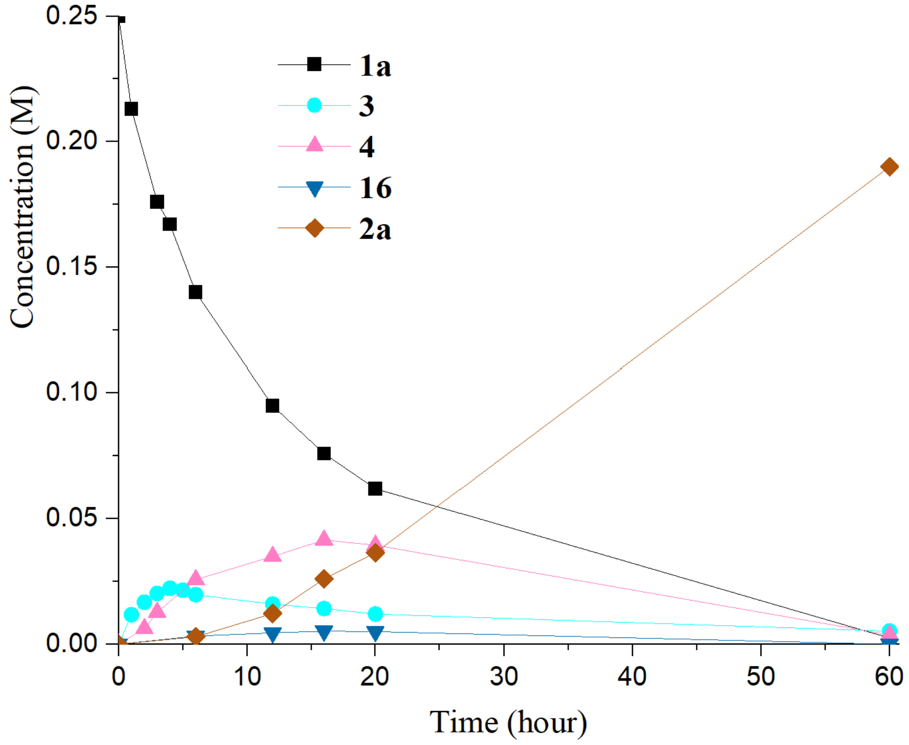

Supplementary Figure 18. Analysis of the reaction profile.

5.5 Kinetic studies

5.5.1 Determination of kinetic rate constants of the first step: k_1

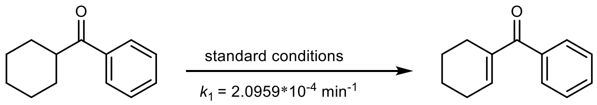

1a **3**

A 15 mL sealed tube containing a magnetic stir bar was charged with **1a** (47.0 mg, 0.25 mmol), CuI (4.8 mg, 10 mol%), AgOAc (3.7 mg, 9 mol%) and DMSO (1 mL) sequentially. The tube was evacuated and backfilled with O_2 three times. Subsequently, H_2O (50 uL, 11.1 equiv) and TFA (186 uL, 10.0 equiv) was added. The tube was sealed and the mixture was stirred at 90 °C. A 200 uL reaction mixture was taken at 20 min, 30 min, 40 min, 60 min, 90 min, 110 min, 130 min, 150 min. ^{1}H NMR was taken to determine the amount of **3** using dibromomethane as the internal standard. The obtained yields were plotted as concentration vs. time and the following initial rates were calculated.

Supplementary Table 15. Conversion of the reaction of **1a**.

Reaction time (min)	Concentration (M)
20	0.00425
30	0.006
40	0.0085
60	0.012
90	0.01875
110	0.02225
130	0.027
150	0.03175

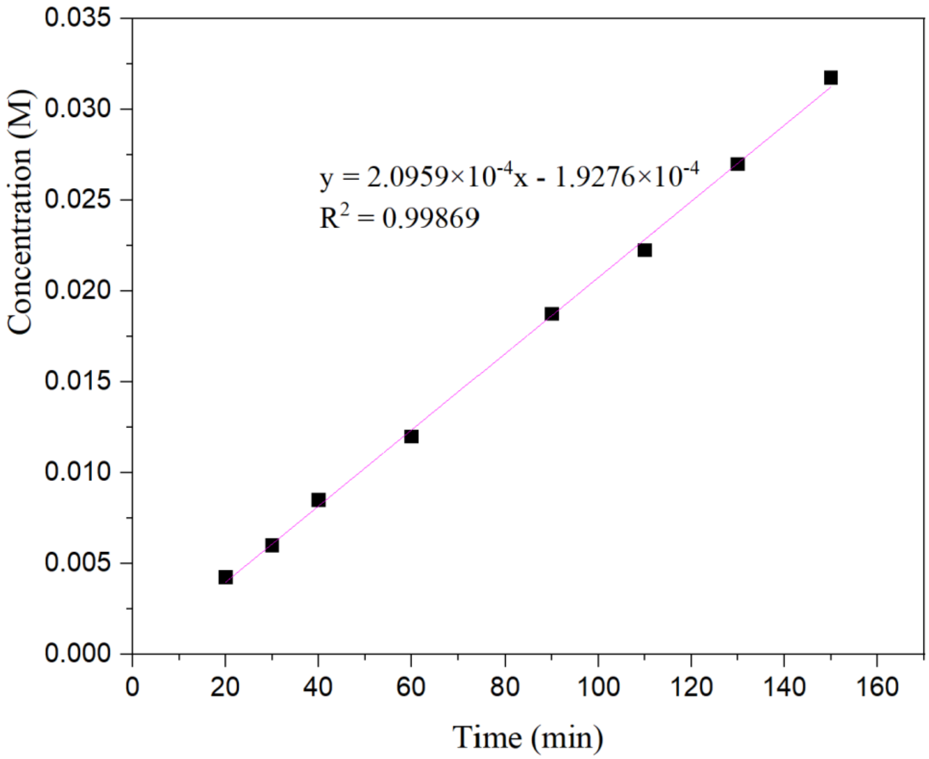

Supplementary Figure 19. Concentration versus time

5.5.2 Determination of kinetic rate constants of the second step: k_2

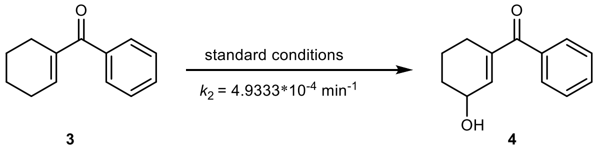

3 **4**

A 15 mL sealed tube containing a magnetic stir bar was charged with **3** (46.5 mg, 0.25 mmol), CuI (4.8 mg, 10 mol%), AgOAc (3.7 mg, 9 mol%) and DMSO (1 mL) sequentially. The tube was evacuated and backfilled with O_2 three times. Subsequently, H_2O (50 uL, 11.1 equiv) and TFA (186 uL, 10.0 equiv) was added. The tube was sealed and the mixture was stirred at 90 °C. A 200 uL reaction mixture was taken at 15 min, 30 min, 45 min, 60 min, 75 min. ^{1}H NMR was

taken to determine the amount of **4** using dibromomethane as the internal standard. The obtained yields were plotted as concentration vs. time and the following initial rates were calculated.

Supplementary Table 16. Conversion of the reaction of **3**.

Reaction time (min)	Concentration (M)
15	0.01425
30	0.02275
45	0.03025
60	0.03575
75	0.04475

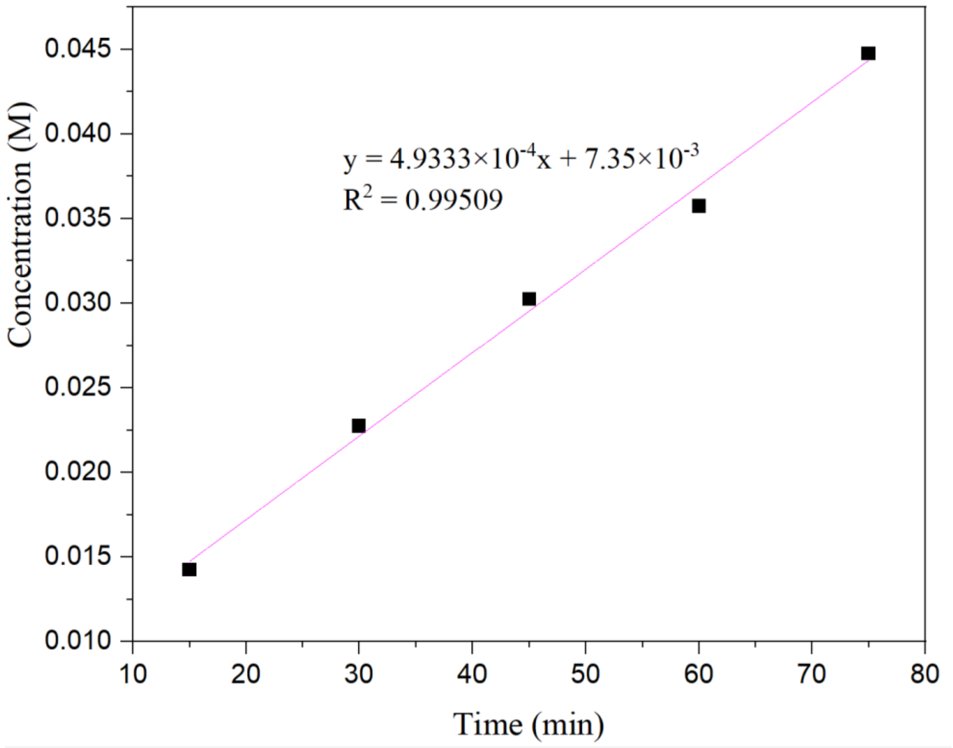

Supplementary Figure 20. Concentration versus time

5.5.3 Determination of kinetic rate constants of the third step: k_3

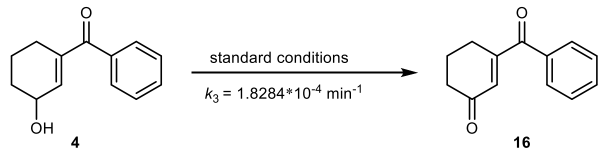

A 15 mL sealed tube containing a magnetic stir bar was charged with **4** (50.5 mg, 0.25 mmol), CuI (4.8 mg, 10 mol%), AgOAc (3.7 mg, 9 mol%) and DMSO (1 mL) sequentially. The tube was evacuated and backfilled with O_2 three times. Subsequently, H_2O (50 uL, 11.1 equiv) and TFA (186 uL, 10.0 equiv) was added. The tube was sealed and the mixture was stirred at 90 °C.

A 200 uL reaction mixture was taken at 30 min, 60 min, 90 min, 120 min, 150 min, 180 min, 210 min, 240 min. ^{1}H NMR was taken to determine the amount of **16** using dibromomethane as the internal standard. The obtained yields were plotted as concentration vs. time and the following initial rates were calculated.

Supplementary Table 17. Conversion of the reaction of **4**.

Reaction time (min)	Concentration (M)
30	0.00475
60	0.009
90	0.01375
120	0.0215
150	0.0265
180	0.032
210	0.03775
240	0.0415

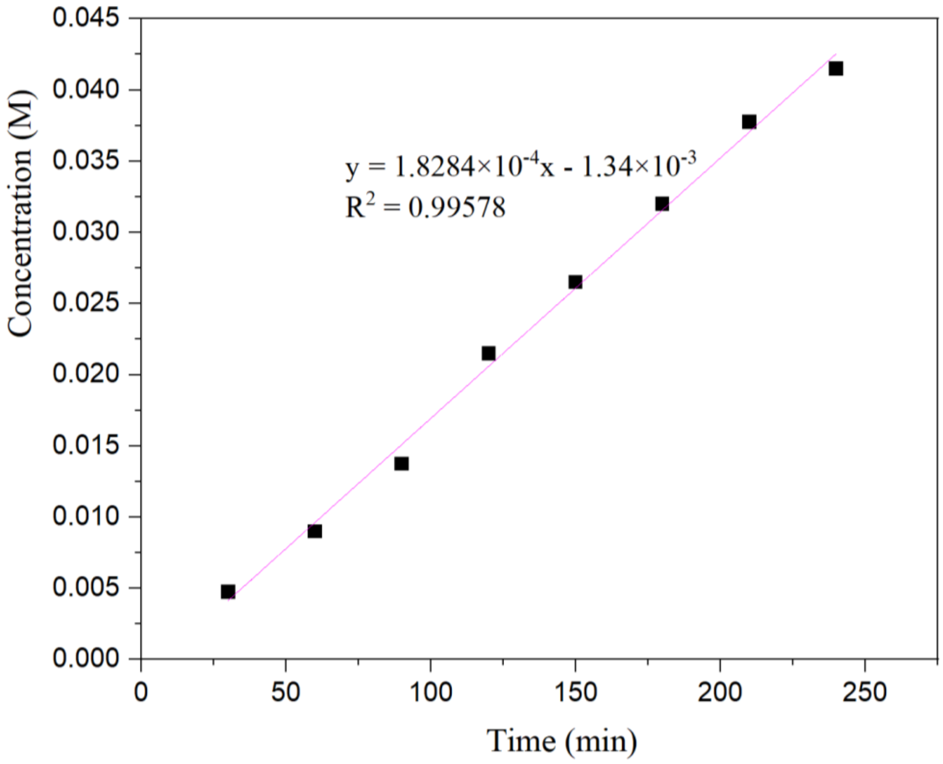

Supplementary Figure 21. Concentration versus time

5.5.4 Determination of kinetic rate constants of the fourth step: k_4

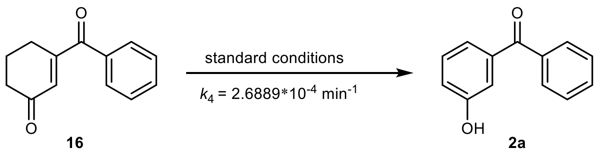

A 15 mL sealed tube containing a magnetic stir bar was charged with **16** (50 mg, 0.25 mmol), CuI (4.8 mg, 10 mol%), AgOAc (3.7 mg, 9 mol%) and DMSO (1 mL) sequentially. The tube was evacuated and backfilled with O_2 three times. Subsequently, H_2O (50 uL, 11.1 equiv) and TFA (186 uL, 10.0 equiv) was added. The tube was sealed and the mixture was stirred at 90 °C. A 200 uL reaction mixture was taken at 30 min, 45 min, 60 min, 75 min, 90 min, 105 min, 120 min, 135 min, 150min. ^{1}H NMR was taken to determine the amount of **2a** using dibromomethane as the internal standard. The obtained yields were plotted as concentration vs. time and the following initial rates were calculated.

Supplementary Table 18. Conversion of the reaction of **16**.

Reaction time (min)	Concentration (M)
30	0.00725
45	0.01175
60	0.01575
75	0.0185
90	0.02275
105	0.02675
120	0.031
135	0.0355
150	0.04025

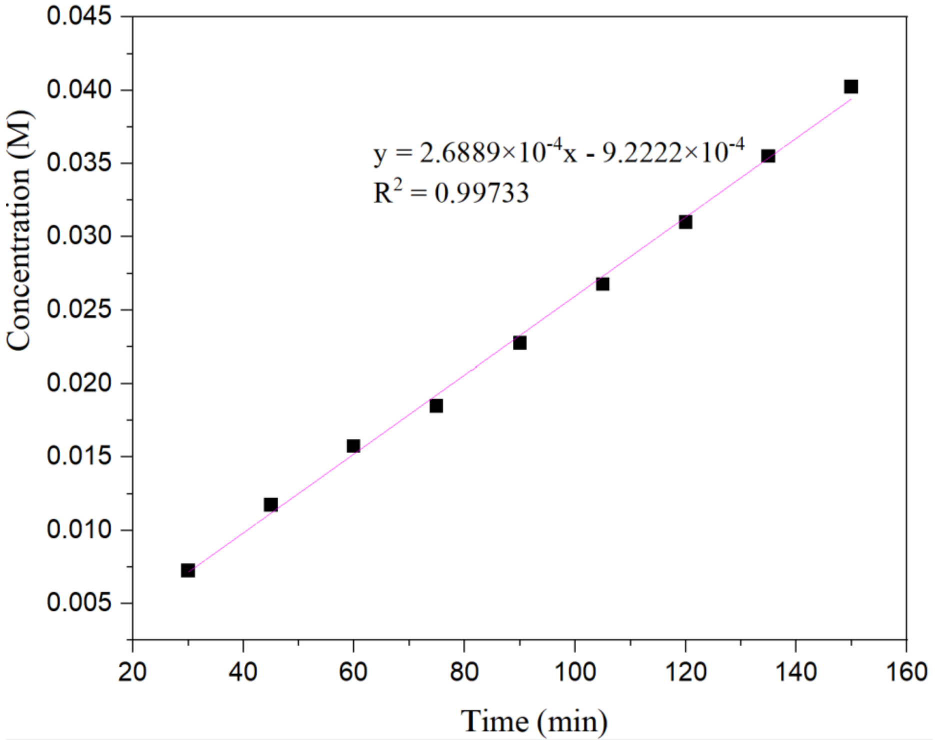

Supplementary Figure 22. Concentration versus time

The rate constants for each step were determined as follows: k_1 = 2.0959×10^{-4} min^{-1}, k_2 = 4.9333×10^{-4} min^{-1}, k_3 = 1.8284×10^{-4} min^{-1}, k_4 = 2.6889×10^{-4} min^{-1}, giving a ratio of $k_1/k_2/k_3/k_4$ = 1.15 : 2.70 : 1 : 1.47.

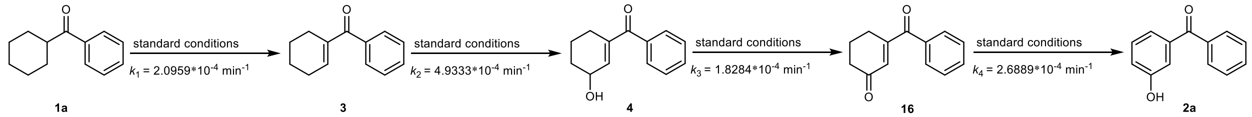

5.6 Proposed mechanism

Based on the experimental results and the related literature, a plausible reaction mechanism for synthesis of *meta*-carbonyl phenols or anilines was proposed in Supplementary Figure 23. First, Cu^{I} species is oxidized in situ by AgOAc in the presence of TFA under oxygen atmosphere, generating $Cu^{II}(O_2CCF_3)_2$; meanwhile, the ketone suffers from the enolization. The formation of copper(II) enolate followed by the oxidation or the disproportionation gives copper(III) enolate that undergoes β-hydride elimination to deliver the α,β-unsaturated ketone **3** along with a Cu^{III}-hydride intermediate. The Cu^{III}-hydride species eliminates a TFA, resulting in $Cu^{I}O_2CCF_3$ that is reoxidized to $Cu^{II}(O_2CCF_3)_2$ by AgOAc and O_2 in the presence of TFA. Subsequently, α,β-unsaturated ketone **3** isomerizes into diene **E**. The terminal C=C double bond of diene is activated by $Cu^{II}(O_2CCF_3)_2$, and then delivers into Cu^{II} species **F**, meanwhile losing a TFA. Cu^{II} species **F** can be detected by HRMS (HRMS (ESI) m/z: $[M + Na]^+$ Calcd for $C_{15}H_{13}F_3O_3CuNa$ 384.0005; Found 383.9997). Cu^{II} species **F** undergoes the oxidation or the disproportionation to give Cu^{III} intermediate **G** which proceeds a reductive elimination to generate intermediate **H** and $Cu^{I}O_2CCF_3$ that proceeds the same process as above to regenerate the active $Cu^{II}(O_2CCF_3)_2$. Intermediate **H** can also be detected by HRMS (HRMS (ESI) m/z: $[M + Na]^+$ Calcd for $C_{15}H_{13}F_3O_3Na$ 321.0709; Found 321.0694). Then intermediate **H** is hydrolyzed to furnish the product **4**. **4** is oxidized producing 1,4-enedione **16** which then undergoes the similar procedure like **1a** to **3**, affording the targeted product **2a**.

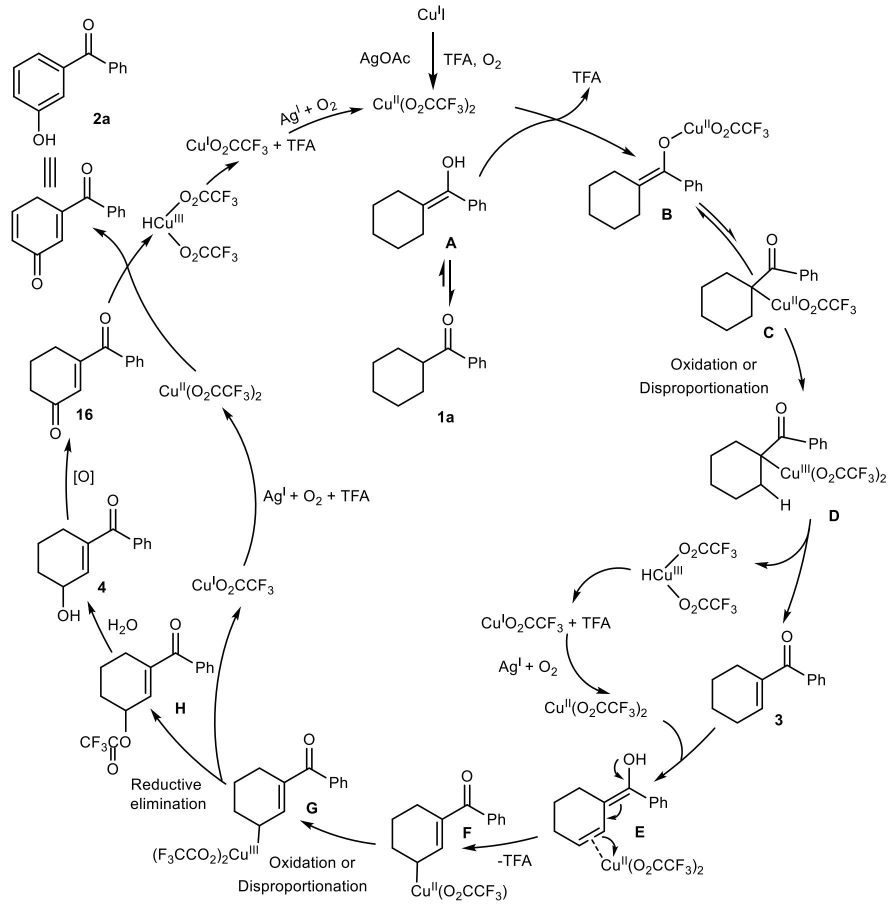

Supplementary Figure 23. Proposed mechanism.

5.7 Product diversification

Synthesis of 3-benzylphenol[23]

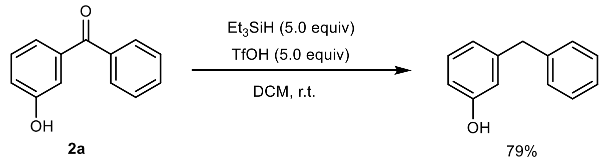

To a solution of **2a** (79.2 mg, 0.4 mmol, 1.0 equiv) in CH_2Cl_2 (5 mL) was added Et_3SiH (0.33 mL, 2 mmol, 5.0 equiv) and TfOH (0.18 mL, 2 mmol, 5.0 equiv) at room temperature. Upon completion, the mixture was poured into a pre-cooled saturated aqueous $NaHCO_3$ solution (5 mL), The organic layers were separated and the aqueous layer was extracted with CH_2Cl_2 (2 × 15 mL). The combined organic layers were washed with brine (5 mL), dried over anhydrous Na_2SO_4, and concentrated to give the residue, which was further purified by silica gel column chromatography to afford product **3-benzylphenol** (58.1 mg, 79%).

^{1}H NMR (400 MHz, $CDCl_3$) δ 7.31 – 7.25 (m, 2H), 7.22 – 7.11 (m, 4H), 6.79 – 6.75 (m, 1H), 6.67 – 6.63 (m, 1H), 6.63 – 6.60 (m, 1H), 4.93 (br, 1H), 3.92 (s, 2H). ^{13}C NMR (101 MHz, $CDCl_3$) δ 155.7, 143.2, 140.9, 129.8, 129.1 (2C), 128.6 (2C), 126.3, 121.6, 116.0, 113.2, 41.9.

The spectroscopic data matches the previously reported data[24].

Synthesis of 3-(hydroxymethyl)phenol[25]

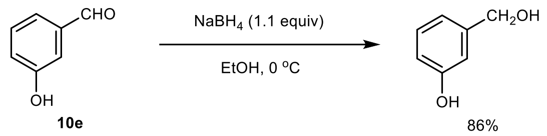

To a stirred solution of **10e** (366.4 mg, 3 mmol, 1.0 equiv) in anhydrous EtOH (6 mL) at 0 °C was added $NaBH_4$ (124.8 mg, 3.3 mmol, 1.1 equiv) slowly. The reaction mixture was stirred at 0 °C for 10 min and then quenched with saturated aqueous NH_4Cl (3 mL). The organic layers were separated and the aqueous layer was extracted with EtOAc (3 × 10 mL). The combined organic layers were washed with brine (3 mL), dried over anhydrous Na_2SO_4, and concentrated to give the residue, which was further purified by silica gel column chromatography to afford product **3-(hydroxymethyl)phenol** (320.3 mg, 86%).
^{1}H NMR (400 MHz, DMSO-d_6) δ 9.28 (br, 1H), 7.12 – 7.06 (m, 1H), 6.76 – 6.73 (m, 1H), 6.73 – 6.69 (m, 1H), 6.63 – 6.59 (m, 1H), 5.11 (t, *J* = 5.7 Hz, 1H), 4.41 (d, *J* = 5.4 Hz, 2H). ^{13}C NMR (101 MHz, DMSO-d_6) δ 157.3, 144.2, 129.1, 117.0, 113.6, 113.3, 62.9.
The spectroscopic data matches the previously reported data[26].

Synthesis of 3-hydroxybenzoic acid

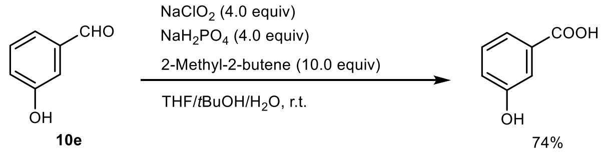

To a 25 mL round bottom flask containing a magnetic stir bar was charged with **10e** (366.4 mg, 3 mmol, 1.0 equiv), THF (3 mL), *t*BuOH (3 mL), and H_2O (1 mL) sequentially. Subsequently, NaH_2PO_4 (1.44 g, 12 mmol, 4.0 equiv), 2-Methyl-2-butene (2.10 g, 30 mmol, 10.0 equiv), and $NaClO_2$ (1.09 g, 12 mmol, 4.0 equiv) was added. After stirring for 2 h at room temperature, additional NaH_2PO_4 (0.72 g, 6 mmol, 2.0 equiv) and $NaClO_2$ (0.54 g, 6 mmol, 2.0 equiv) was added again. Upon completion, the reaction mixture was diluted with CH_2Cl_2 (30 mL) and washed with H_2O (3 × 2 mL) and brine (2 mL). The combined organic layers were dried over anhydrous Na_2SO_4, and concentrated to give the residue, which was further purified by silica gel column chromatography to afford product **3-hydroxybenzoic acid** (306.4 mg, 74%).
^{1}H NMR (400 MHz, DMSO-d_6) δ 12.86 (br, 1H), 9.78 (br, 1H), 7.43 – 7.39 (m, 1H), 7.38 – 7.35 (m, 1H), 7.33 – 7.27 (m, 1H), 7.02 (dd, *J* = 8.4, 2.6 Hz, 1H). ^{13}C NMR (101 MHz, DMSO-d_6) δ 167.4, 157.5, 132.1, 129.7, 120.1, 119.9, 115.9.
The spectroscopic data matches the previously reported data[27].

Synthesis of (*E*)-3-(3-hydroxyphenyl)-1-phenylprop-2-en-1-one[1]

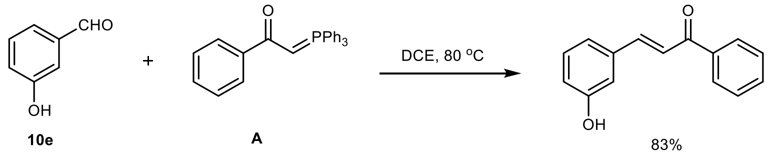

To a 50 mL round bottom flask containing a magnetic stir bar was charged with **10e** (0.37 g, 3 mmol, 1.0 equiv.) and **A** (1.37 g, 3.6 mmol, 1.2 equiv) using 1,2-dichloroethane (6 mL) as solvent, was heated at 80 °C in an oil bath. After completion of the reaction, the solution was concentrated by evaporation to give the residue, which was further purified by silica gel column chromatography to give *α,β*-unsaturated ketone (0.56 g, 83%).
^{1}H NMR (400 MHz, DMSO-d_6) δ 9.67 (br, 1H), 8.19 – 8.12 (m, 2H), 7.89 – 7.82 (m, 1H), 7.72 – 7.64 (m, 2H), 7.62 – 7.54 (m, 2H), 7.36 – 7.31 (m, 1H), 7.30 – 7.23 (m, 2H), 6.90 (dd, *J* = 7.9, 2.5 Hz, 1H). ^{13}C NMR (101 MHz, DMSO-d_6) δ 189.3, 157.8, 144.4, 137.6, 136.0, 133.2, 129.9, 128.8 (2C), 128.6 (2C), 121.9, 119.9, 117.9, 115.3.
The spectroscopic data matches the previously reported data[28].

Synthesis of (3-aminophenyl)(phenyl)methanone[29]

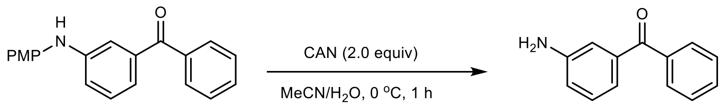

12m 46%

To a solution of **12m** (15.1 mg, 0.05 mmol, 1.0 equiv) in acetonitrile (1 mL) was added dropwise a solution of cerium ammonium nitrate (54.8 mg, 0.1 mmol, 2.0 equiv) in water (0.5 mL) at 0 °C over 20 min. After stirring for 1 h, the reaction mixture was quenched by adding 5% aqueous $NaHCO_3$ solution until pH = 6. The mixture was further added sodium sulfite until it becomes a brown suspension, then extracted with EtOAc (3 × 20 mL). The combined organic layers were dried over anhydrous Na_2SO_4, filtered, and concentrated under vacuum. The residue was purified by silica gel flash column chromatography to afford the *meta*-substituted aniline (4.5 mg, 46%).
^{1}H NMR (400 MHz, $CDCl_3$) δ 7.84 – 7.78 (m, 2H), 7.61 – 7.54 (m, 1H), 7.51 – 7.43 (m, 2H), 7.28 – 7.21 (m, 1H), 7.16 – 7.09 (m, 2H), 6.92 – 6.86 (m, 1H), 3.72 (br, 2H). ^{13}C NMR (101 MHz, $CDCl_3$) δ 197.1, 146.6, 138.8, 137.9, 132.4, 130.2 (2C), 129.2, 128.3 (2C), 120.8, 119.1, 116.1.
The spectroscopic data matches the previously reported data[30].

6. X-Ray Crystallographic Data

The single crystal for compound **2k, 8a, 12i** and **12k** were prepared from a mixture solvent of dichloromethane and Petroleum ether (v/v = 1:4). The data were collected on a Bruker Smart APEXIICCD instrument using Mo-Kα radiation (λ = 0.71073 Å) at 296 K. The crystal structures were solved and refined using the SHELXTL software package. All non-hydrogen atoms were refined anisotropically. Hydrogen atoms were added in the riding model and refined with isotropic thermal parameters. The crystallographic data have already been deposited at the Cambridge Crystallographic Data Centre. CCDC numbers: 2264711 (**2k**), 2264714 (**8a**), 2264715 (**12i**) and 2264716 (**12k**).

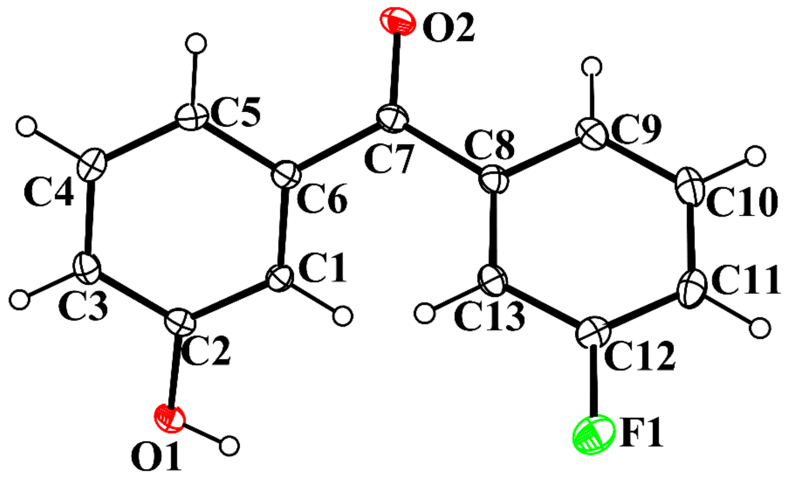

Supplementary Figure 24. X-ray derived ORTEP of **2k** with thermal ellipsoids shown at the 30% probability level

Supplementary Table 19. Crystal data and structure refinement for **2k**

Identification code	**2k**
Empirical formula	C13 H9 F O2
Formula weight	216.20
Temperature	150.0 K
Wavelength	1.34139 Å
Crystal system	Orthorhombic
Space group	Pbca
Unit cell dimensions	a = 7.8303(9) Å α = 90°.
	b = 12.3029(13) Å β = 90°.
	c = 20.903(2) Å γ = 90°.
Volume	2013.7(4) Å^3
Z	8
Calculated density	1.426 Mg/m^3
Absorption coefficient	0.581 mm^{-1}

F(000)	896
Crystal size	0.32 x 0.24 x 0.22 mm^3
Theta range for data collection	3.679 to 54.944°.
Index ranges	-8<=h<=9, -14<=k<=14, -25<=l<=25
Reflections collected	19376
Independent reflections	1915 [R(int) = 0.0619]
Completeness to theta = 53.594°	99.9 %
Absorption correction	Semi-empirical from equivalents
Max. and min. transmission	0.7508 and 0.6351
Refinement method	Full-matrix least-squares on F^2
Data / restraints / parameters	1915 / 0 / 146
Goodness-of-fit on F^2	1.110
Final R indices [I>2sigma(I)]	R1 = 0.0454, wR2 = 0.1348
R indices (all data)	R1 = 0.0483, wR2 = 0.1374
Largest diff. peak and hole	0.749 and -0.316 e.Å^{-3}

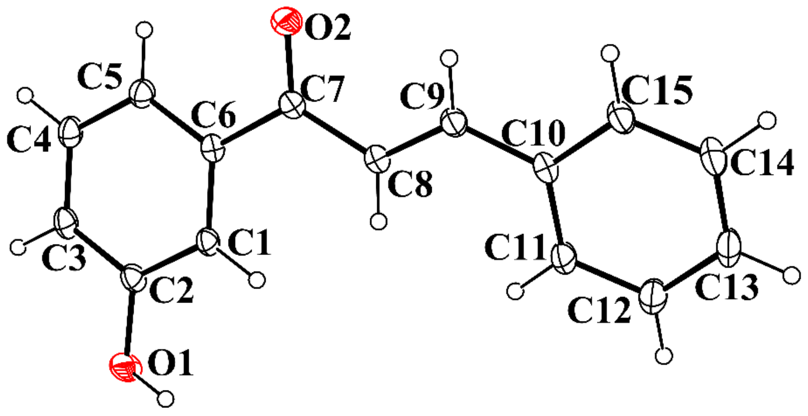

Supplementary Figure 25. X-ray derived ORTEP of **8a** with thermal ellipsoids shown at the 30% probability level

Supplementary Table 20. Crystal data and structure refinement for **8a**

Type or paste caption here. Create a page break and paste in the table above the caption.

Identification code	**8a**	
Empirical formula	C15 H12 O2	
Formula weight	224.25	
Temperature	150.0 K	
Wavelength	1.34138 Å	
Crystal system	Monoclinic	
Space group	P 1 21/c 1	
Unit cell dimensions	a = 12.6299(9) Å	α = 90°.

	b = 12.0299(7) Å β = 102.285(4)°.
	c = 7.6700(4) Å γ = 90°.
Volume	1138.67(12) $Å^3$
Z	4
Calculated density	1.308 Mg/m^3
Absorption coefficient	0.443 mm^{-1}
F(000)	472
Crystal size	0.18 x 0.14 x 0.12 mm^3
Theta range for data collection	4.465 to 56.949°.
Index ranges	-15<=h<=15, -15<=k<=14, -9<=l<=8
Reflections collected	9018
Independent reflections	2286 [R(int) = 0.0635]
Completeness to theta = 53.594°	98.2 %
Absorption correction	Semi-empirical from equivalents
Max. and min. transmission	0.7512 and 0.3608
Refinement method	Full-matrix least-squares on F^2
Data / restraints / parameters	2286 / 0 / 158
Goodness-of-fit on F^2	1.087
Final R indices [I>2sigma(I)]	R1 = 0.0703, wR2 = 0.1805
R indices (all data)	R1 = 0.0725, wR2 = 0.1846
Largest diff. peak and hole	0.393 and -0.421 e.$Å^{-3}$

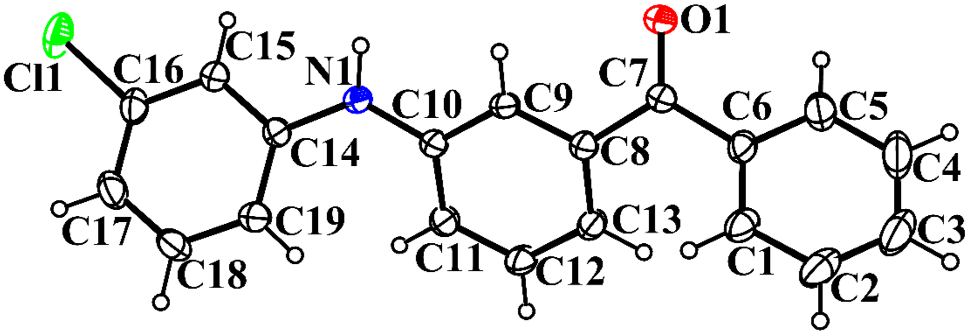

Supplementary Figure 26. X-ray derived ORTEP of **12i** with thermal ellipsoids shown at the 30% probability level

Supplementary Table 21. Crystal data and structure refinement for **12i**

Identification code	**12i**
Empirical formula	C19 H14 Cl N O
Formula weight	307.76
Temperature	260.0 K
Wavelength	1.34139 Å

Crystal system	Monoclinic
Space group	P 1 21/n 1
Unit cell dimensions	a = 7.4040(6) Å α = 90°.
	b = 14.9600(13) Å β = 92.581(4)°.
	c = 13.7178(12) Å γ = 90°.
Volume	1517.9(2) Å^3
Z	4
Calculated density	1.347 Mg/m^3
Absorption coefficient	1.448 mm^{-1}
F(000)	640
Crystal size	0.20 x 0.15 x 0.12 mm^3
Theta range for data collection	3.806 to 57.270°.
Index ranges	-9<=h<=9, -18<=k<=18, -16<=l<=17
Reflections collected	12632
Independent reflections	3089 [R(int) = 0.0802]
Completeness to theta = 53.594°	99.1 %
Absorption correction	Semi-empirical from equivalents
Max. and min. transmission	0.7512 and 0.4088
Refinement method	Full-matrix least-squares on F^2
Data / restraints / parameters	3089 / 0 / 199
Goodness-of-fit on F^2	1.149
Final R indices [I>2sigma(I)]	R1 = 0.0582, wR2 = 0.1464
R indices (all data)	R1 = 0.0695, wR2 = 0.1593
Largest diff. peak and hole	0.333 and -0.353 e.Å^{-3}

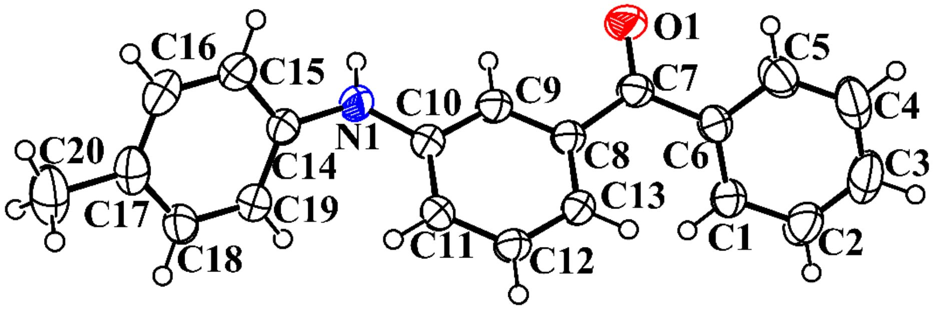

Supplementary Figure 27. X-ray derived ORTEP of **12k** with thermal ellipsoids shown at the 30% probability level

Supplementary Table 22. Crystal data and structure refinement for **12k**

Identification code	**12k**
Empirical formula	C20 H17 N O

Formula weight	287.36
Temperature	260.0 K
Wavelength	1.34139 Å
Crystal system	Monoclinic
Space group	P 1 21/n 1
Unit cell dimensions	a = 15.8980(8) Å α = 90°.
	b = 7.6272(4) Å β = 94.789(2)°.
	c = 25.4492(12) Å γ = 90°.
Volume	3075.1(3) $Å^3$
Z	4
Calculated density	1.241 Mg/m^3
Absorption coefficient	0.382 mm^{-1}
F(000)	1216
Crystal size	0.21 x 0.20 x 0.19 mm^3
Theta range for data collection	3.723 to 57.098°.
Index ranges	-19<=h<=19, -7<=k<=9, -31<=l<=30
Reflections collected	34502
Independent reflections	6283 [R(int) = 0.0773]
Completeness to theta = 53.594°	99.7 %
Absorption correction	Semi-empirical from equivalents
Max. and min. transmission	0.7512 and 0.5284
Refinement method	Full-matrix least-squares on F^2
Data / restraints / parameters	6283 / 0 / 399
Goodness-of-fit on F^2	1.042
Final R indices [I>2sigma(I)]	R1 = 0.0544, wR2 = 0.1429
R indices (all data)	R1 = 0.0901, wR2 = 0.1645
Largest diff. peak and hole	0.314 and -0.288 e.$Å^{-3}$

7. NMR Spectra

^{1}H NMR (400 MHz, $CDCl_3$) and ^{13}C NMR (101 MHz, $CDCl_3$) spectra of **1j-1**

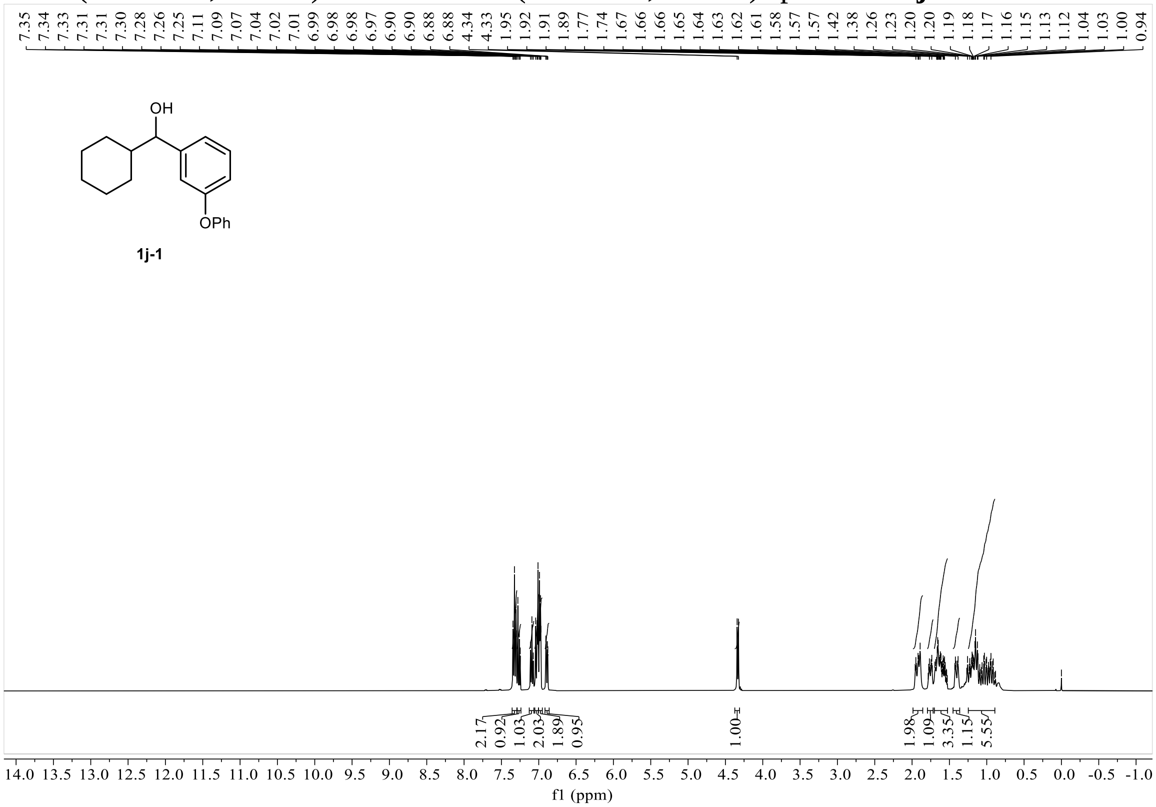

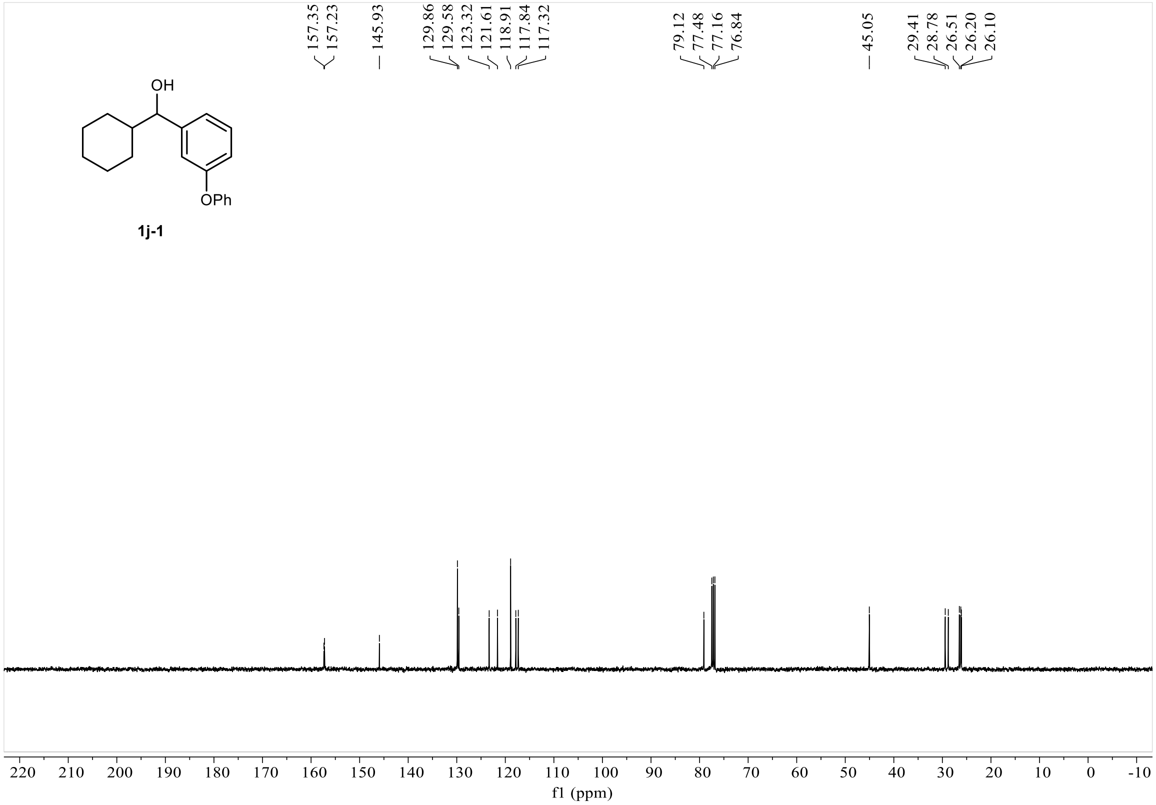

^{1}H NMR (400 MHz, $CDCl_3$) and ^{13}C NMR (101 MHz, $CDCl_3$) spectra of **1j**

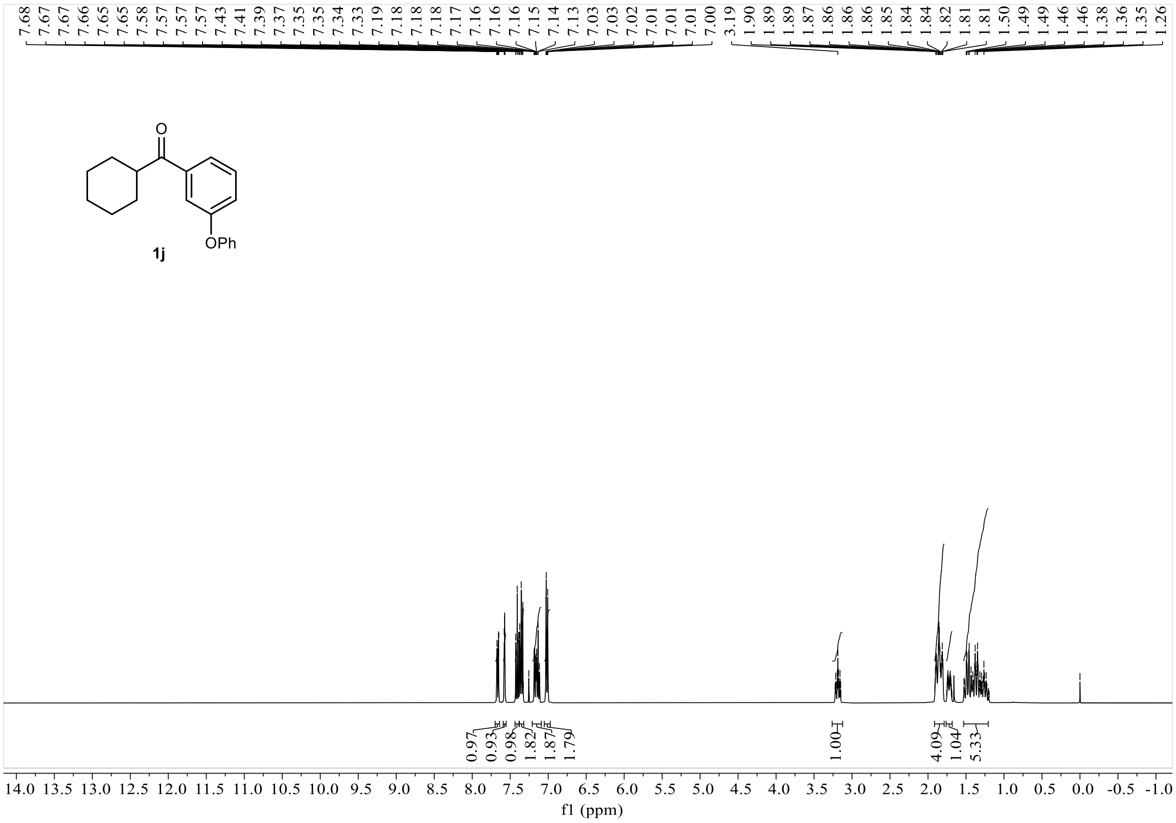

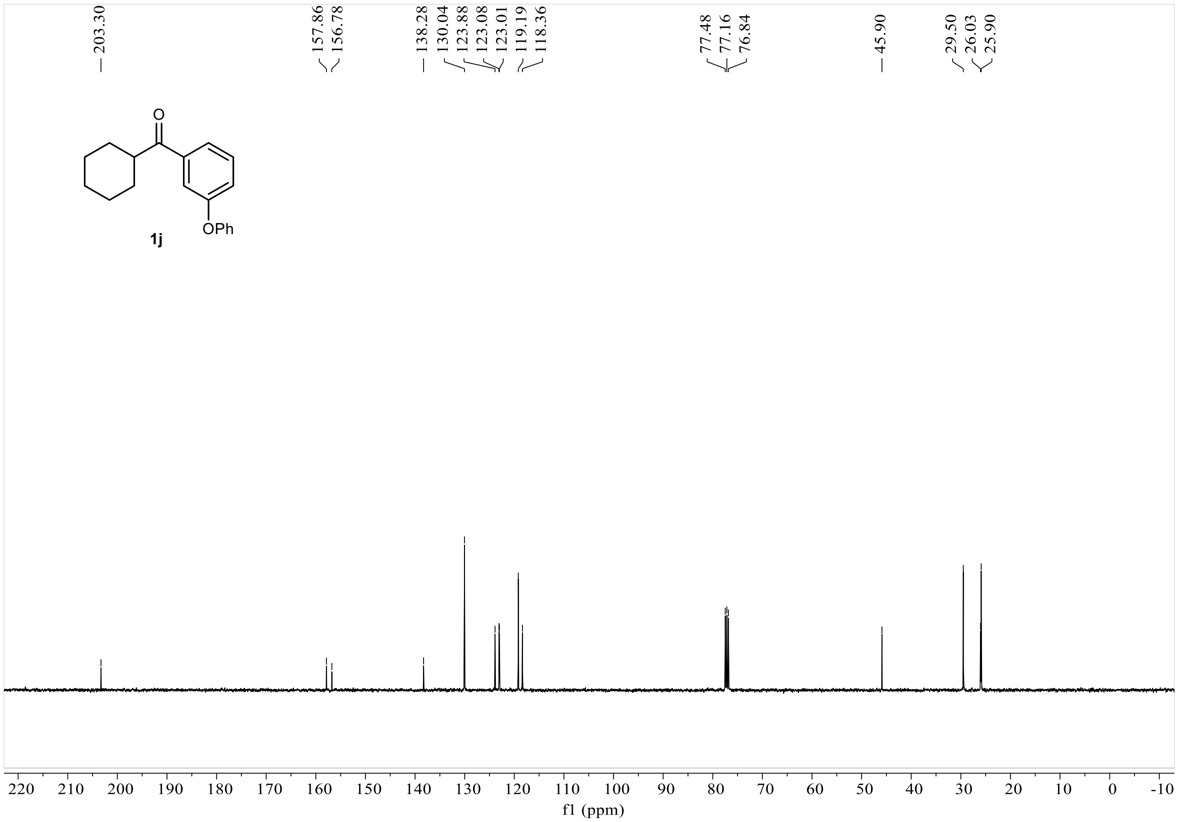

^{1}H NMR (400 MHz, $CDCl_3$) and ^{13}C NMR (101 MHz, $CDCl_3$) spectra of **1m**

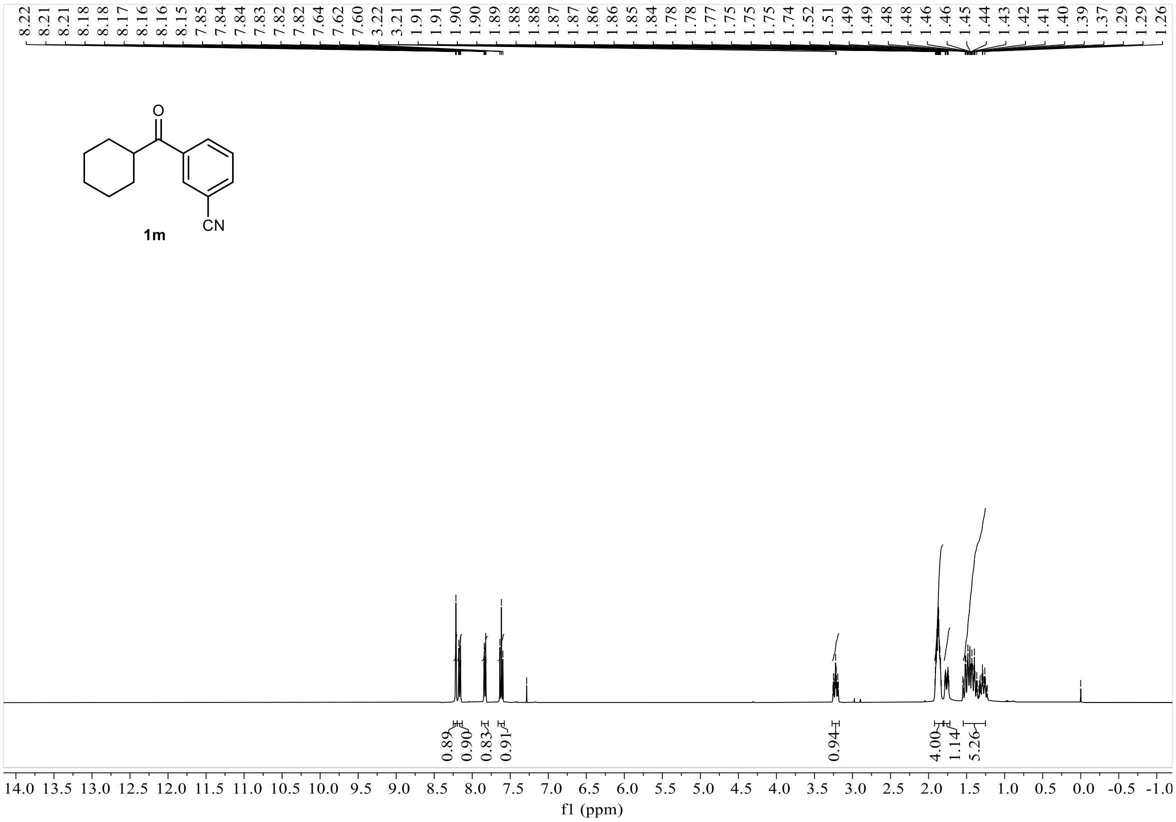

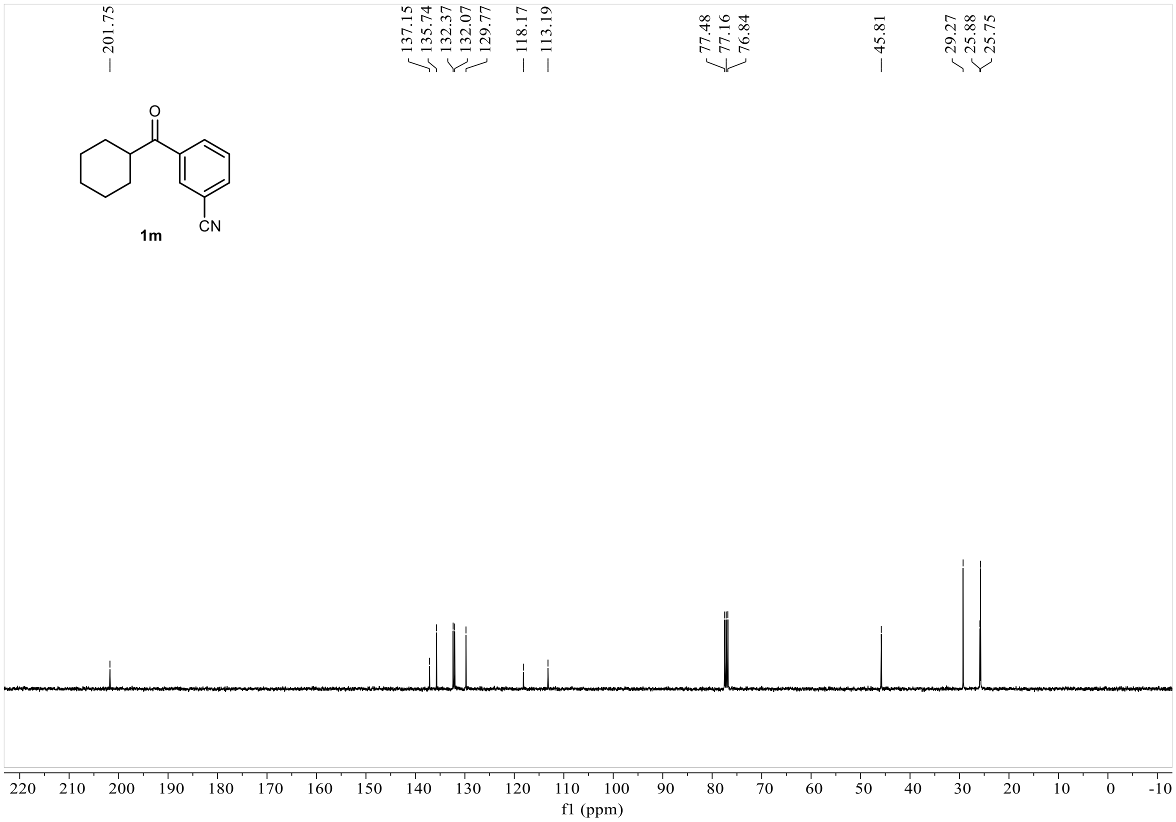

^{1}H NMR (400 MHz, $CDCl_3$) and ^{13}C NMR (101 MHz, $CDCl_3$) spectra of **1o-1**

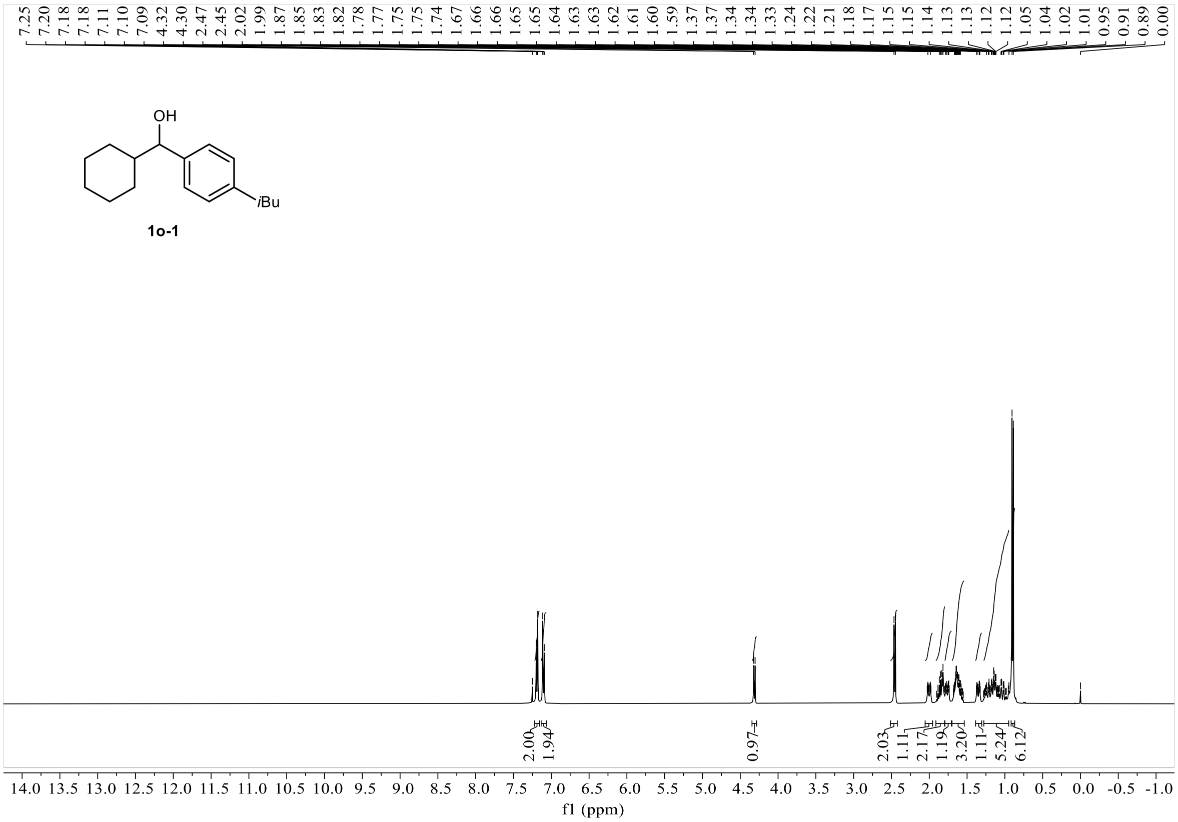

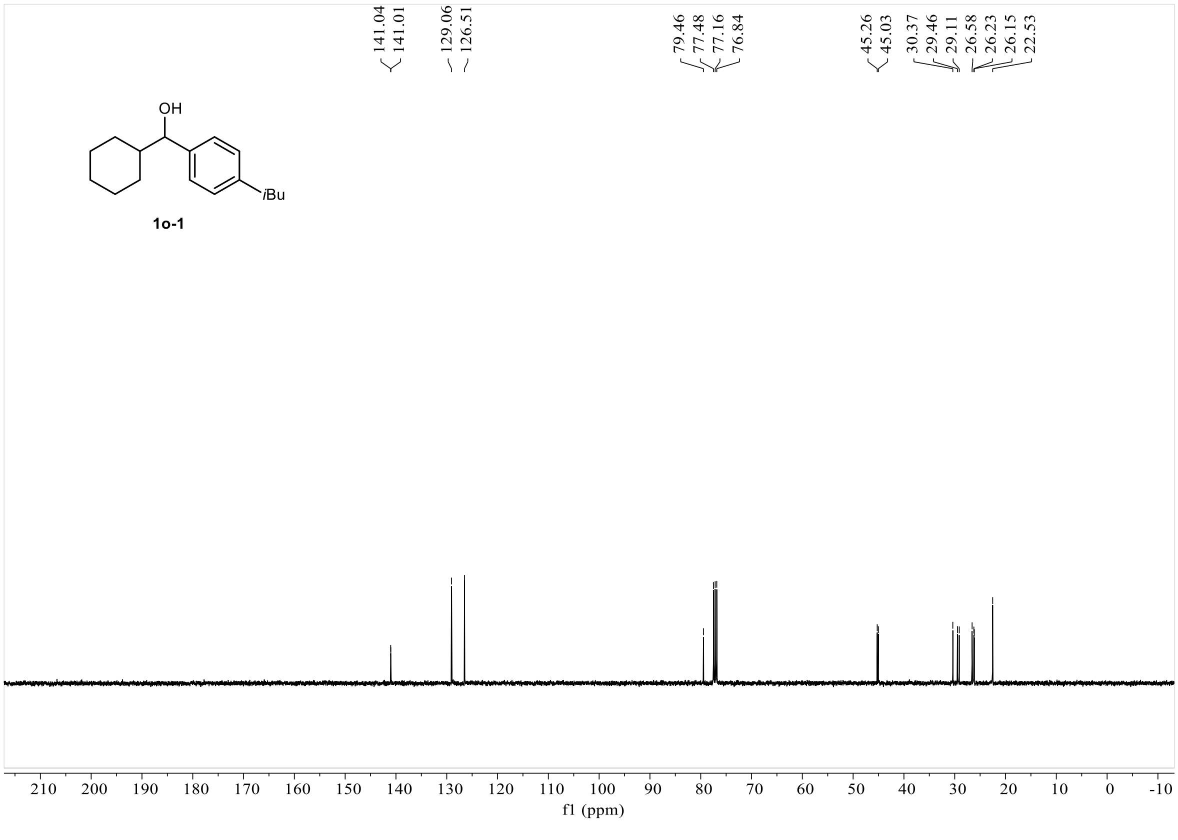

^1H NMR (400 MHz, $CDCl_3$) and ^{13}C NMR (101 MHz, $CDCl_3$) spectra of **1o**

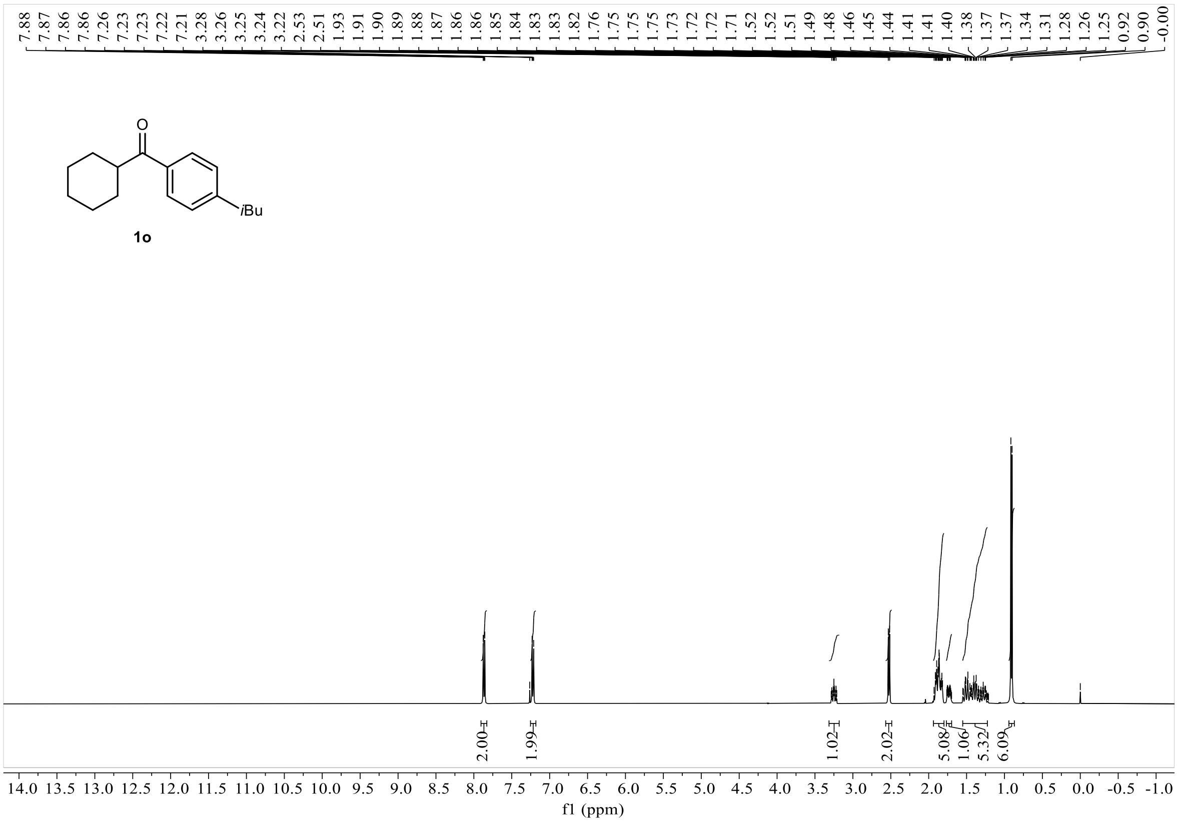

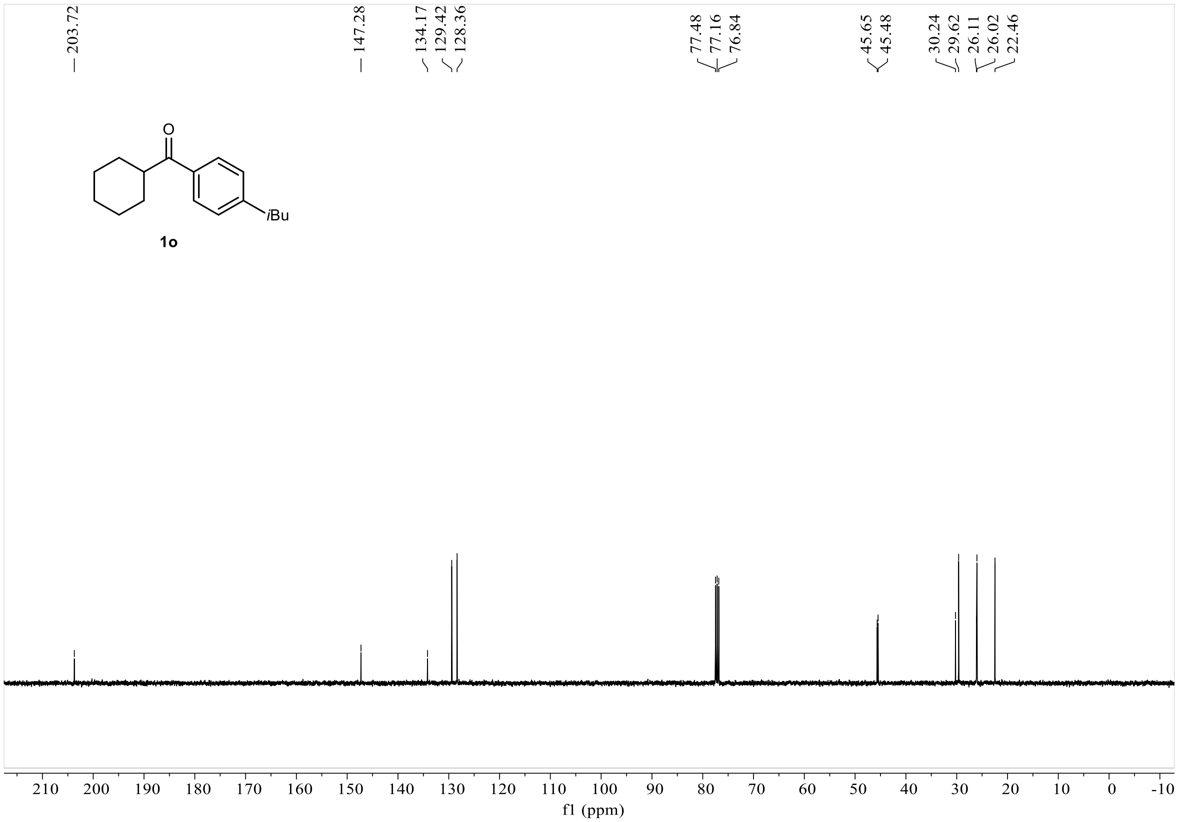

^{1}H NMR (400 MHz, $CDCl_3$) and ^{13}C NMR (101 MHz, $CDCl_3$) spectra of **1s-1**

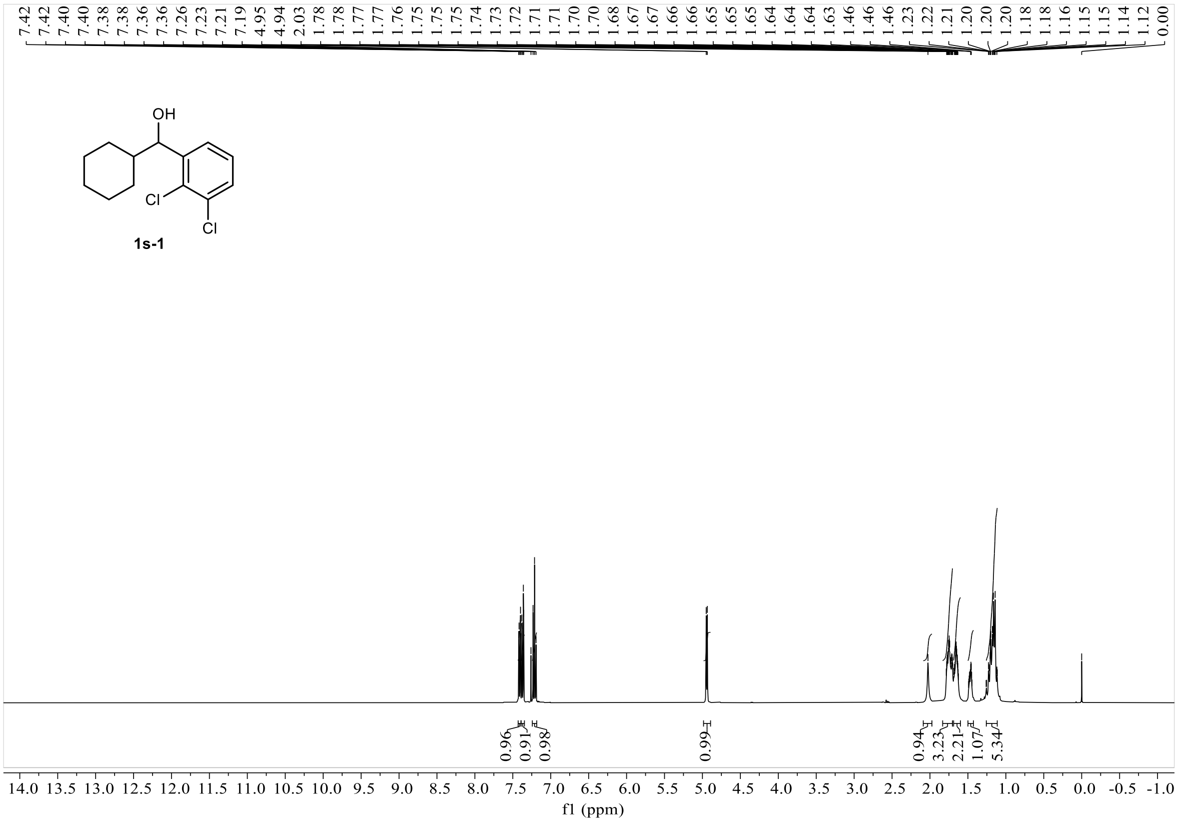

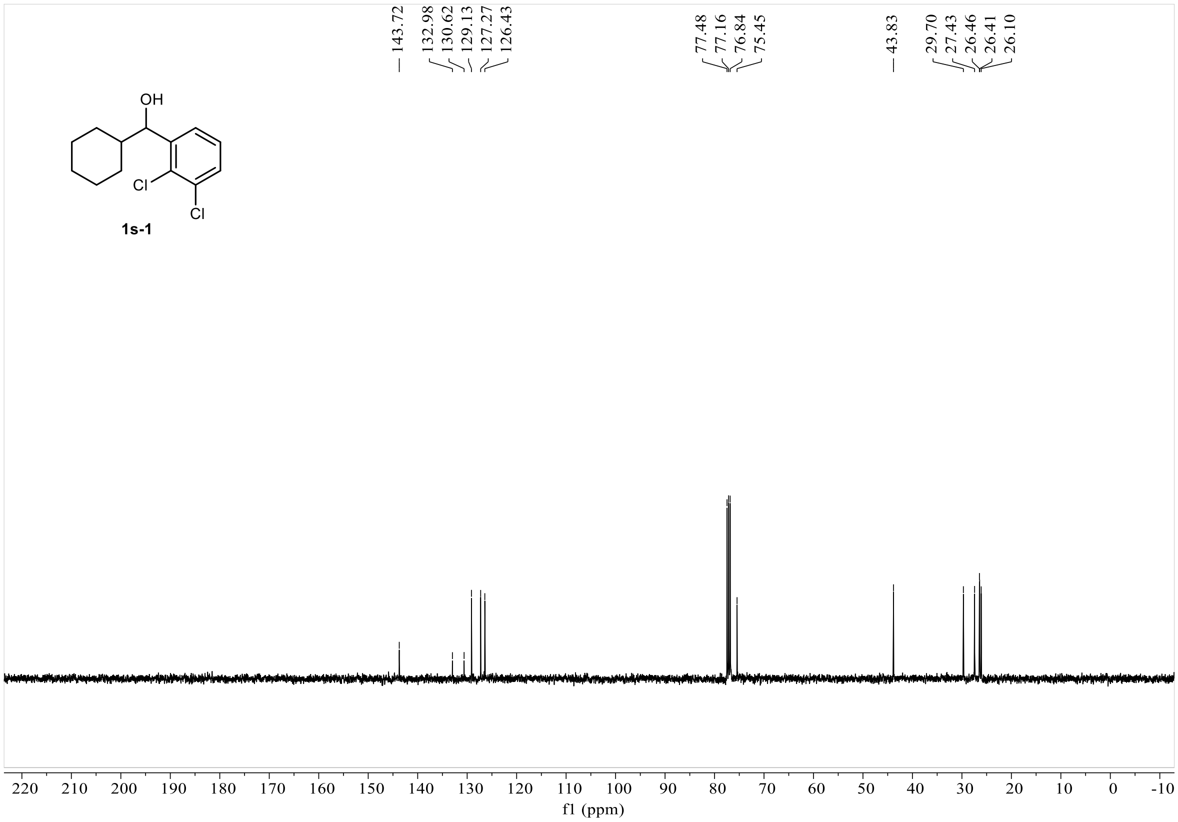

^{1}H NMR (400 MHz, $CDCl_3$) and ^{13}C NMR (101 MHz, $CDCl_3$) spectra of **1s**

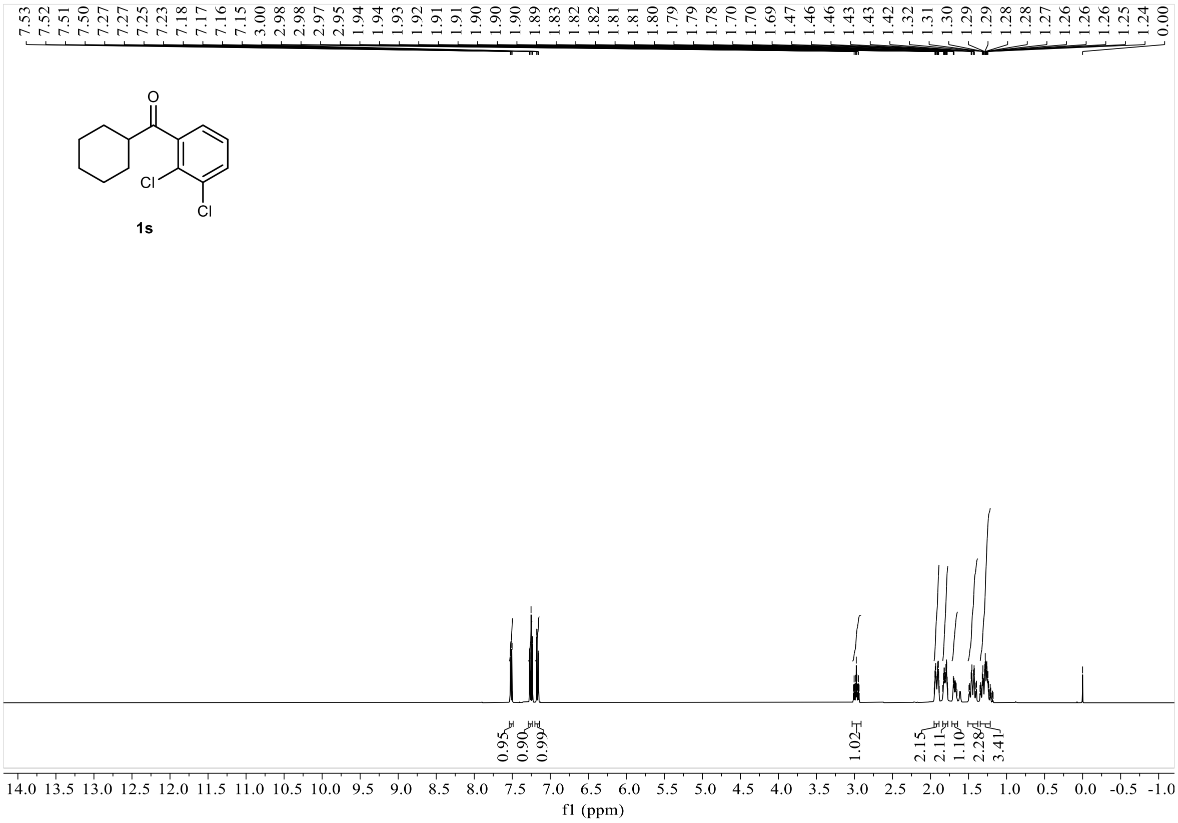

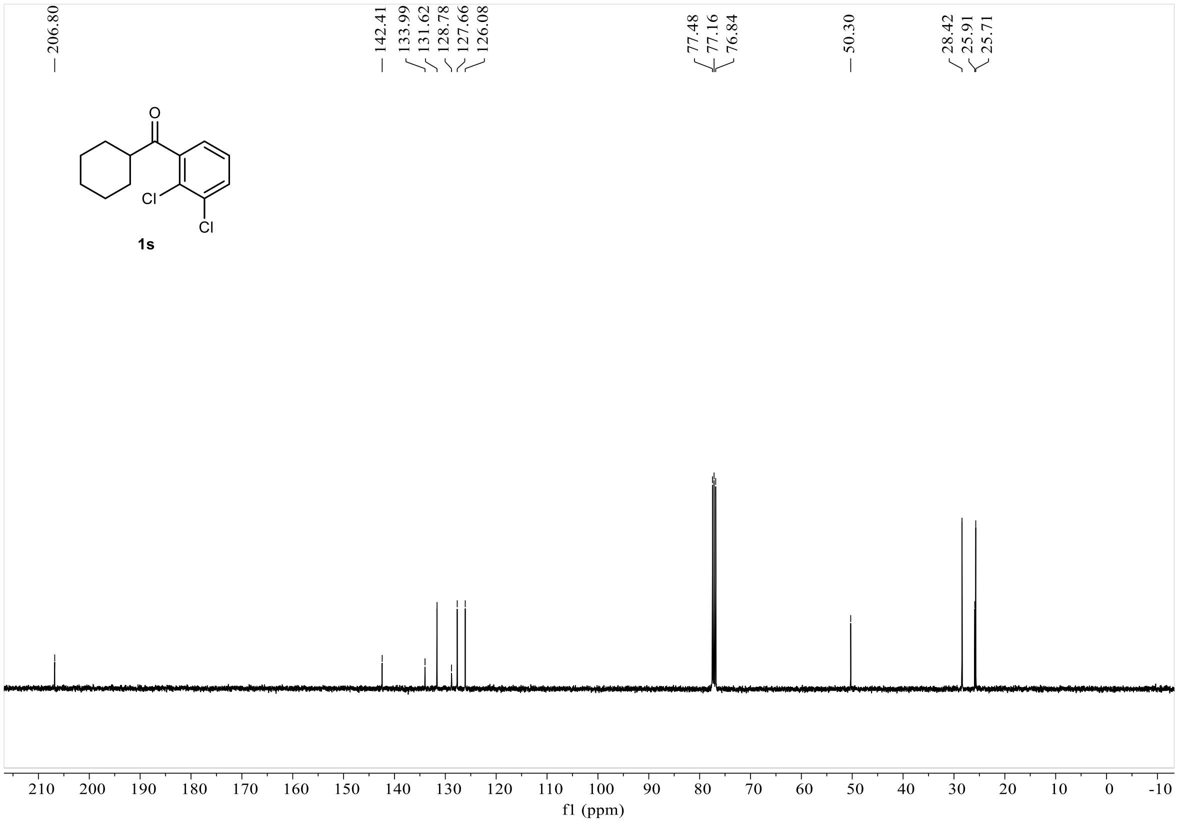

^{1}H NMR (400 MHz, $CDCl_3$) and ^{13}C NMR (101 MHz, $CDCl_3$) spectra of **1t-1**

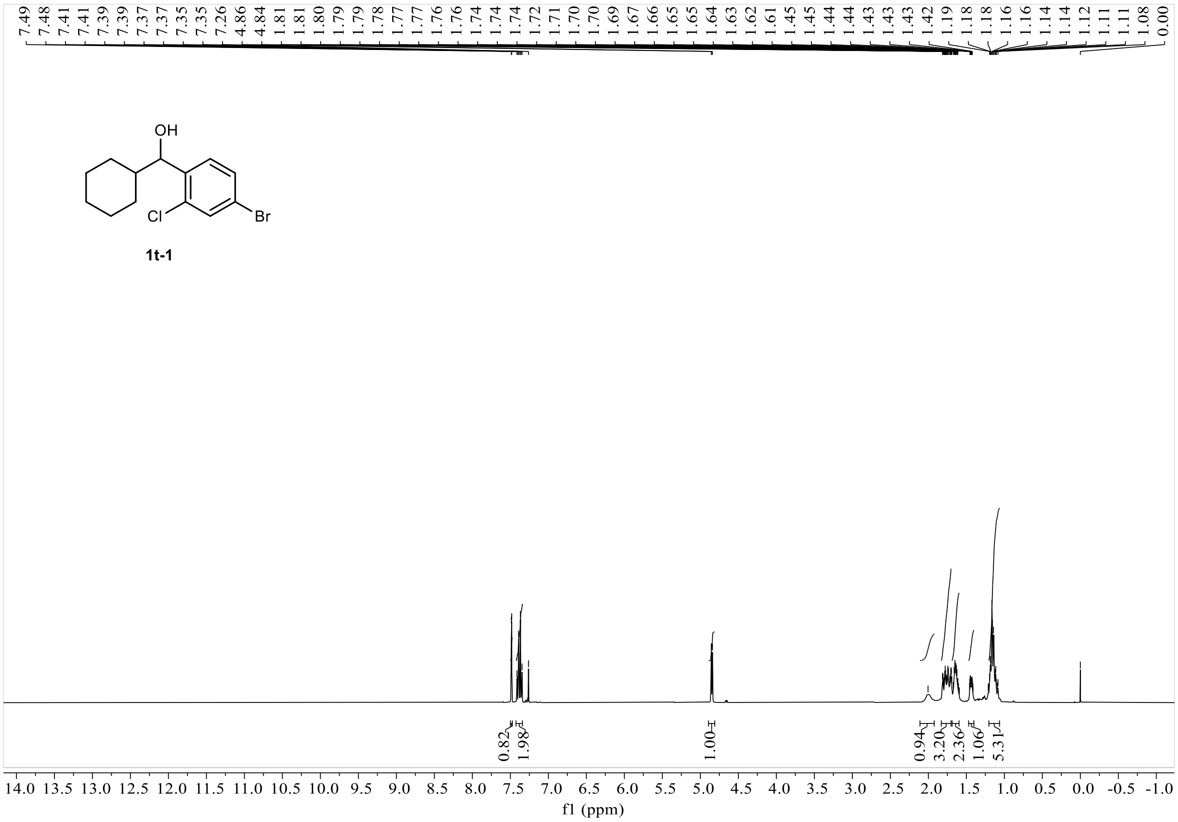

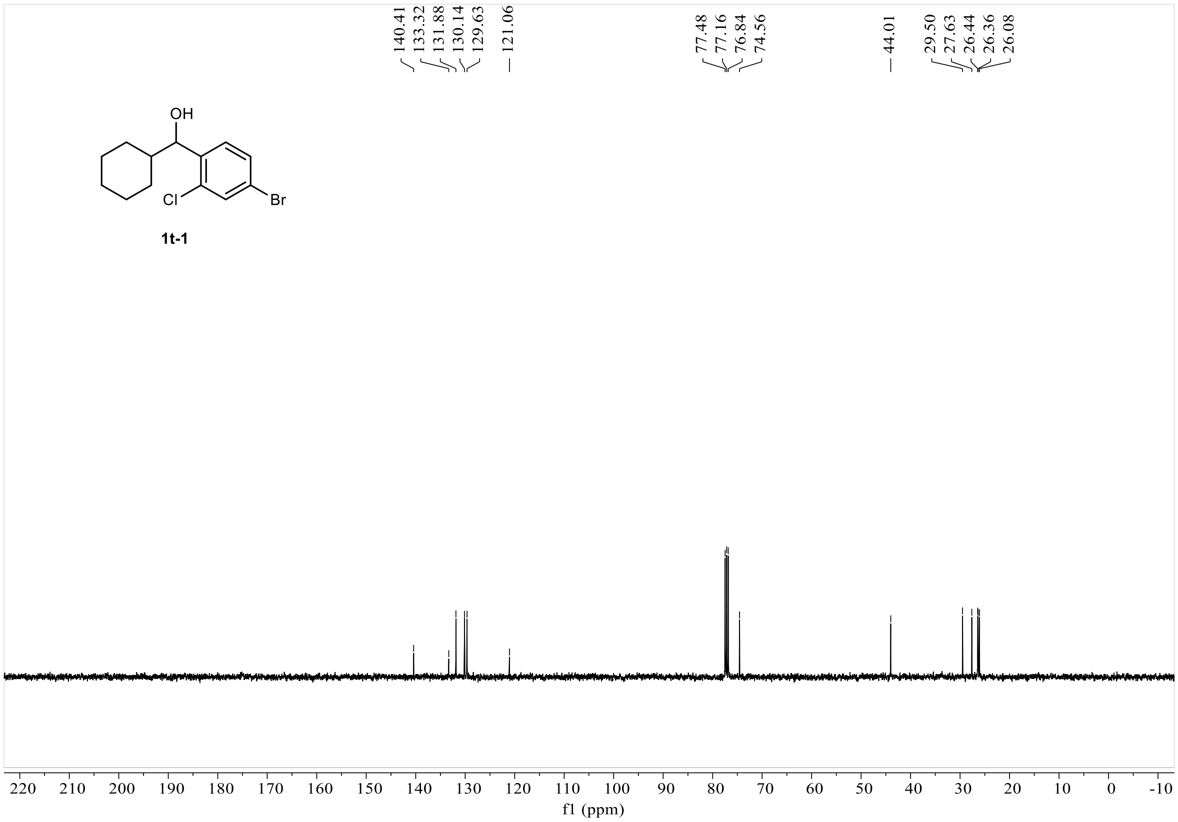

^{1}H NMR (400 MHz, $CDCl_3$) and ^{13}C NMR (101 MHz, $CDCl_3$) spectra of **1t**

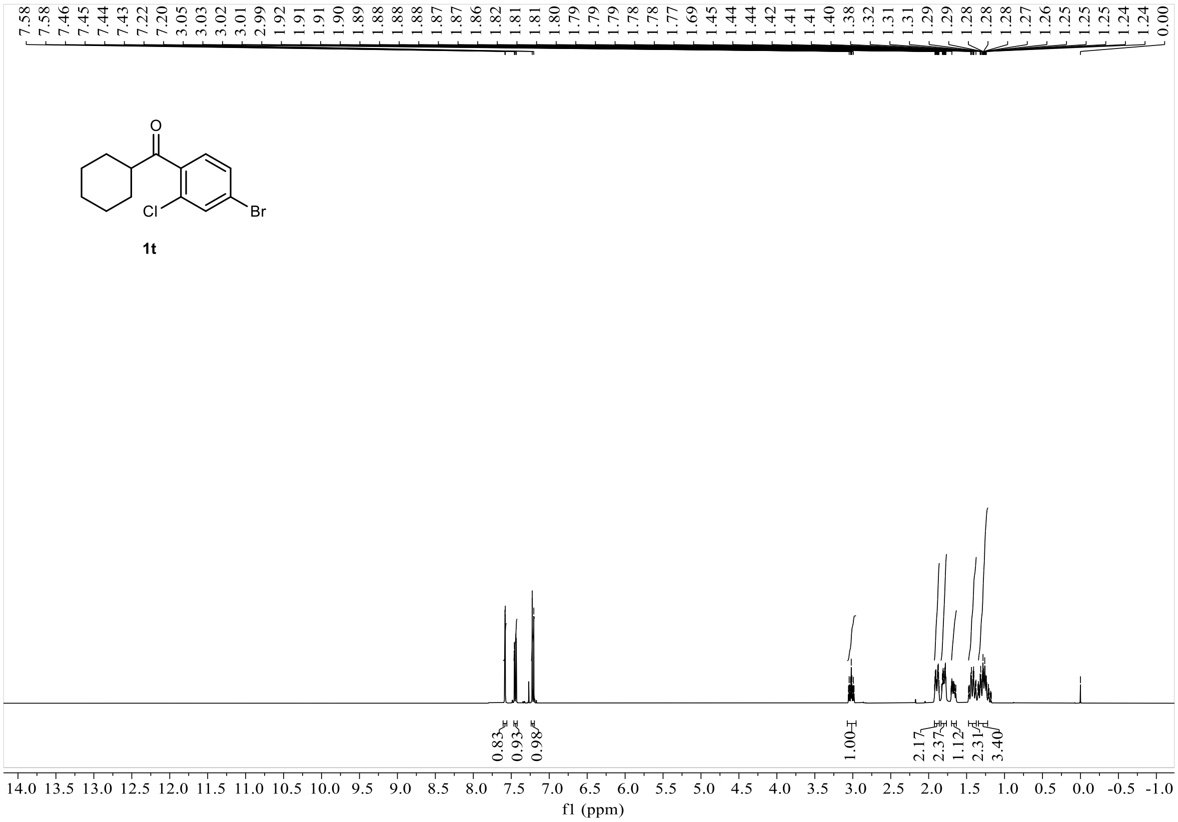

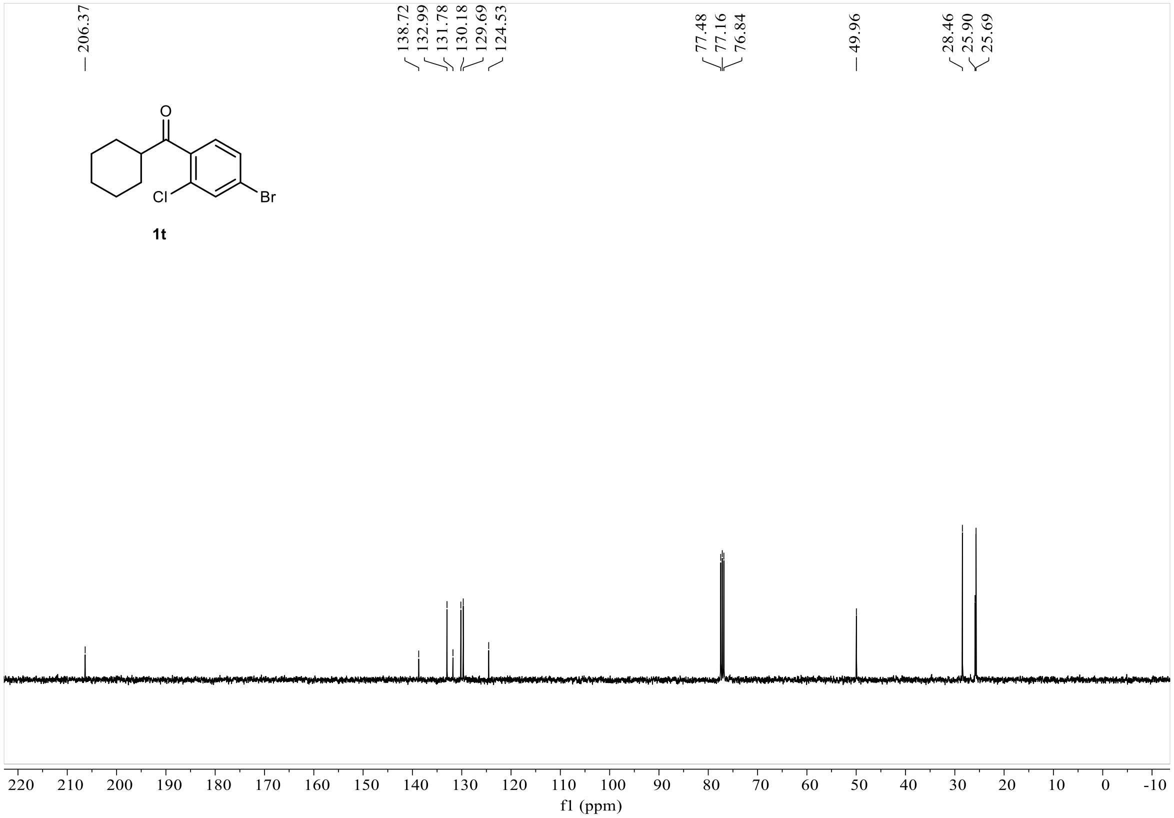

^{1}H NMR (400 MHz, $CDCl_3$) and ^{13}C NMR (101 MHz, $CDCl_3$) spectra of **1u-1**

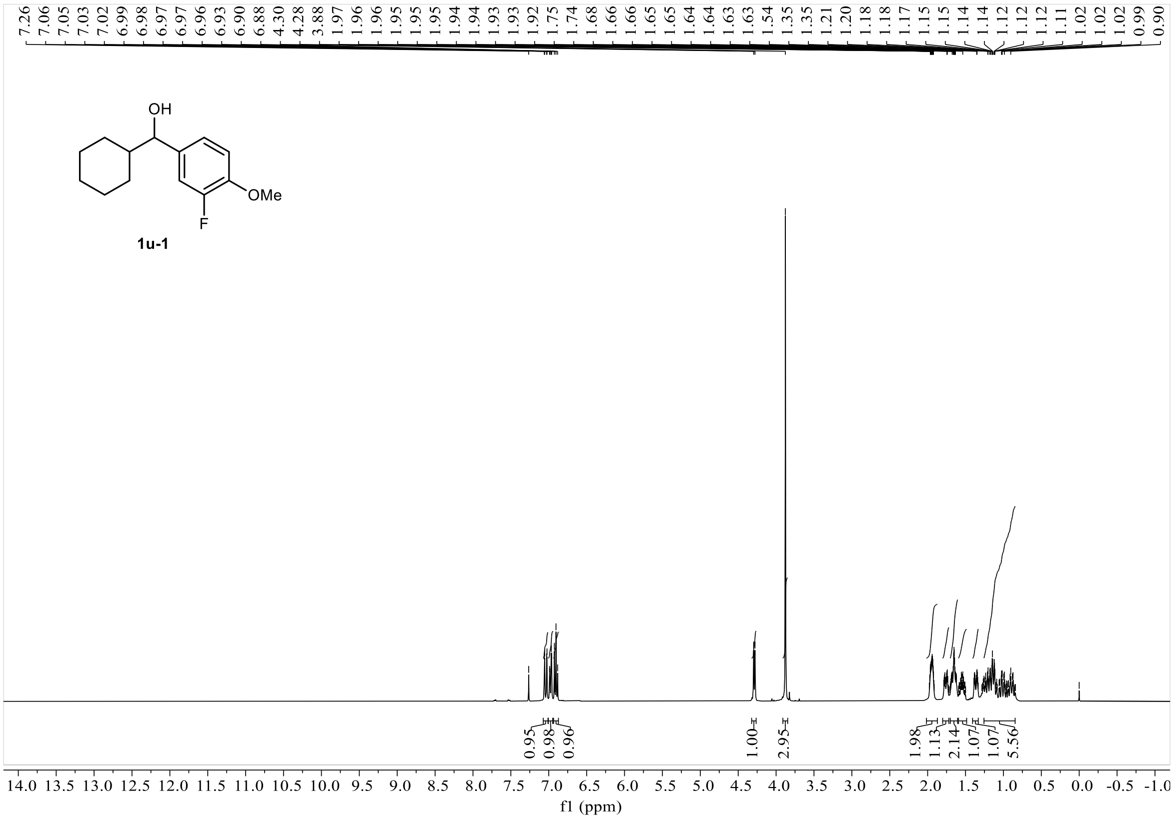

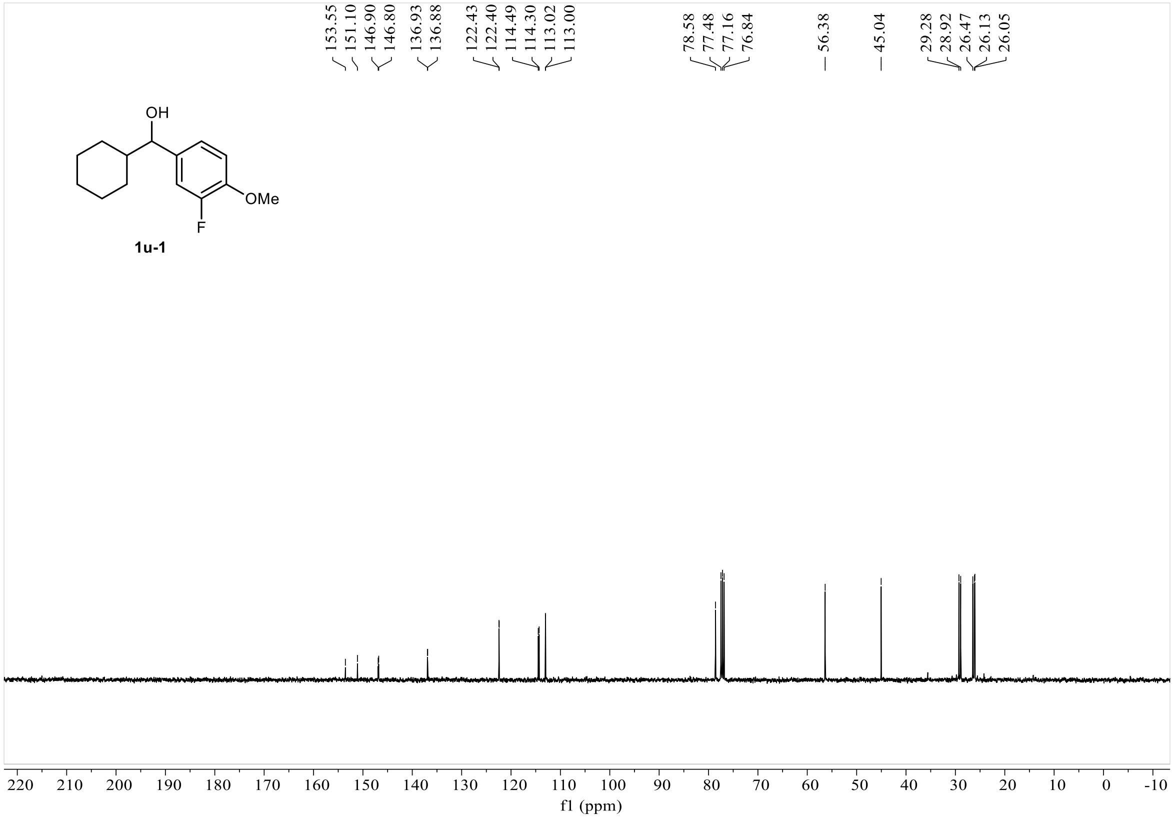

^{19}F NMR (376 MHz, $CDCl_3$) spectrum of **1u-1**

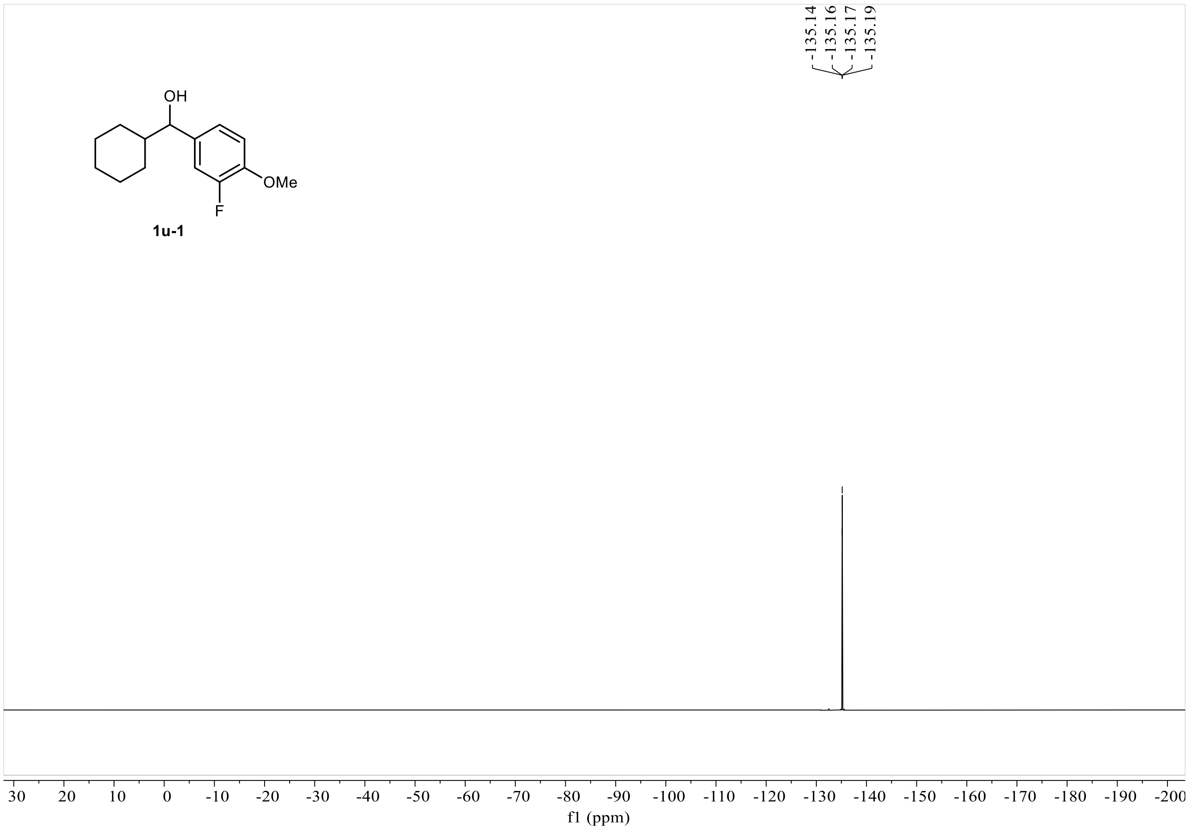

^{1}H NMR (400 MHz, $CDCl_3$) and ^{13}C NMR (101 MHz, $CDCl_3$) spectra of **1u**

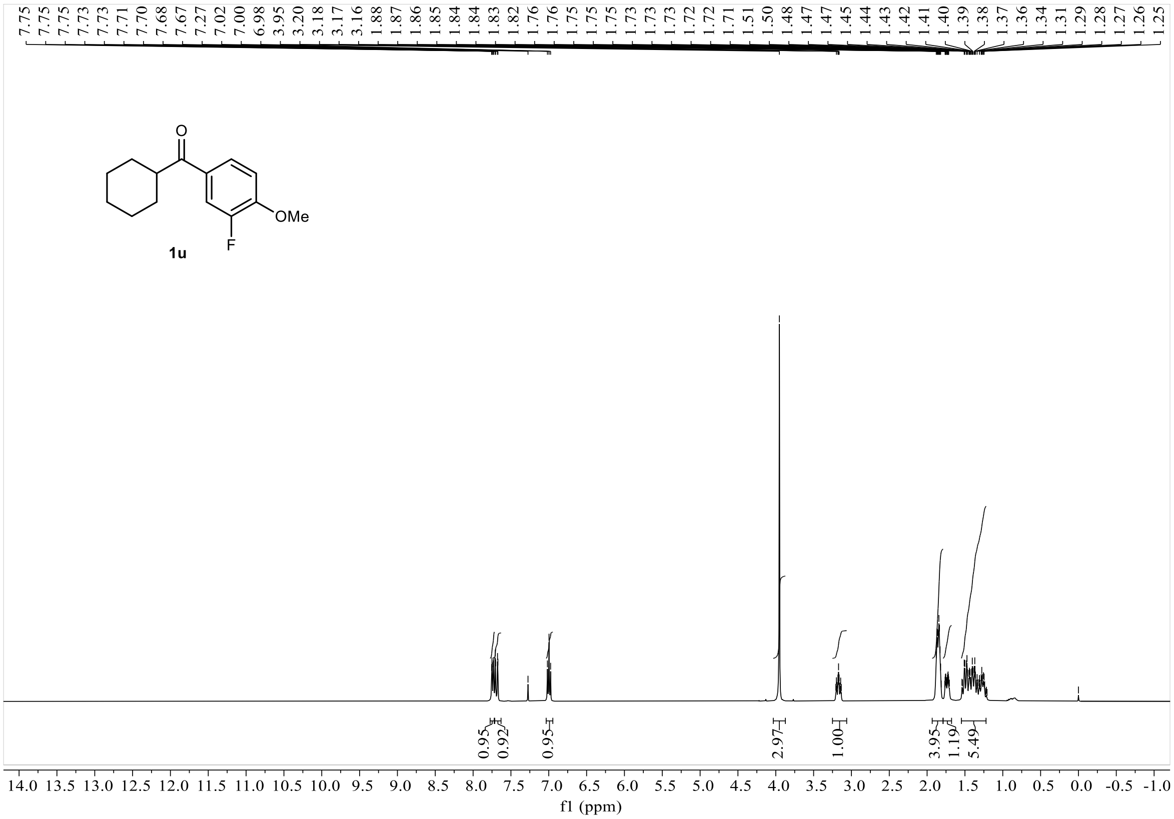

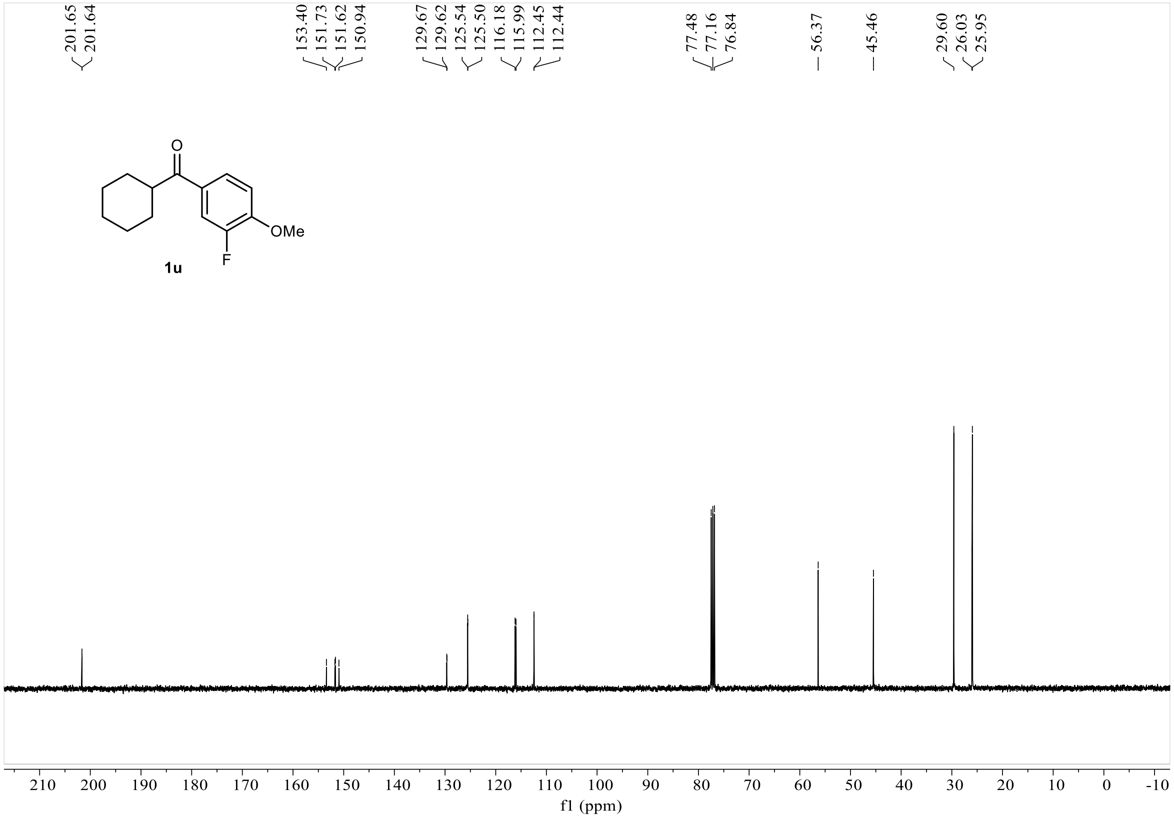

^{19}F NMR (376 MHz, $CDCl_3$) spectrum of **1u**

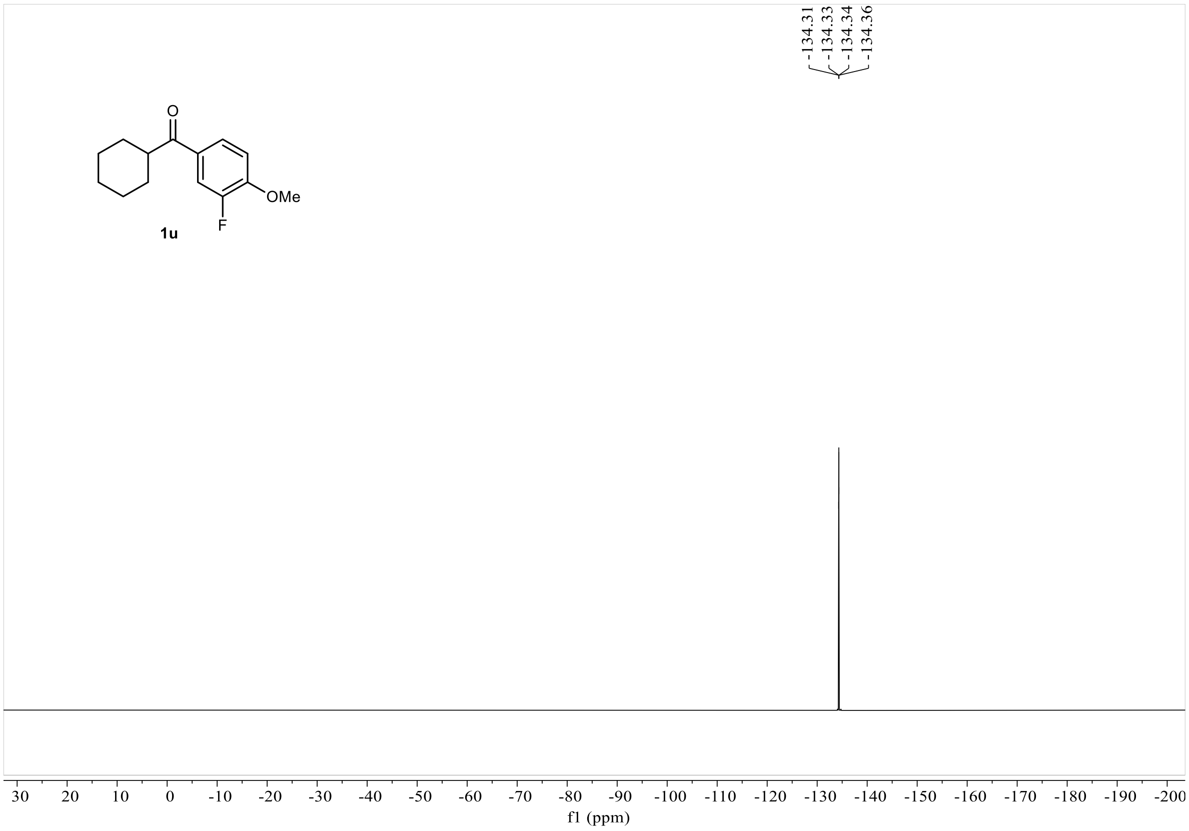

^{1}H NMR (400 MHz, $CDCl_3$) and ^{13}C NMR (101 MHz, $CDCl_3$) spectra of **1x-1**

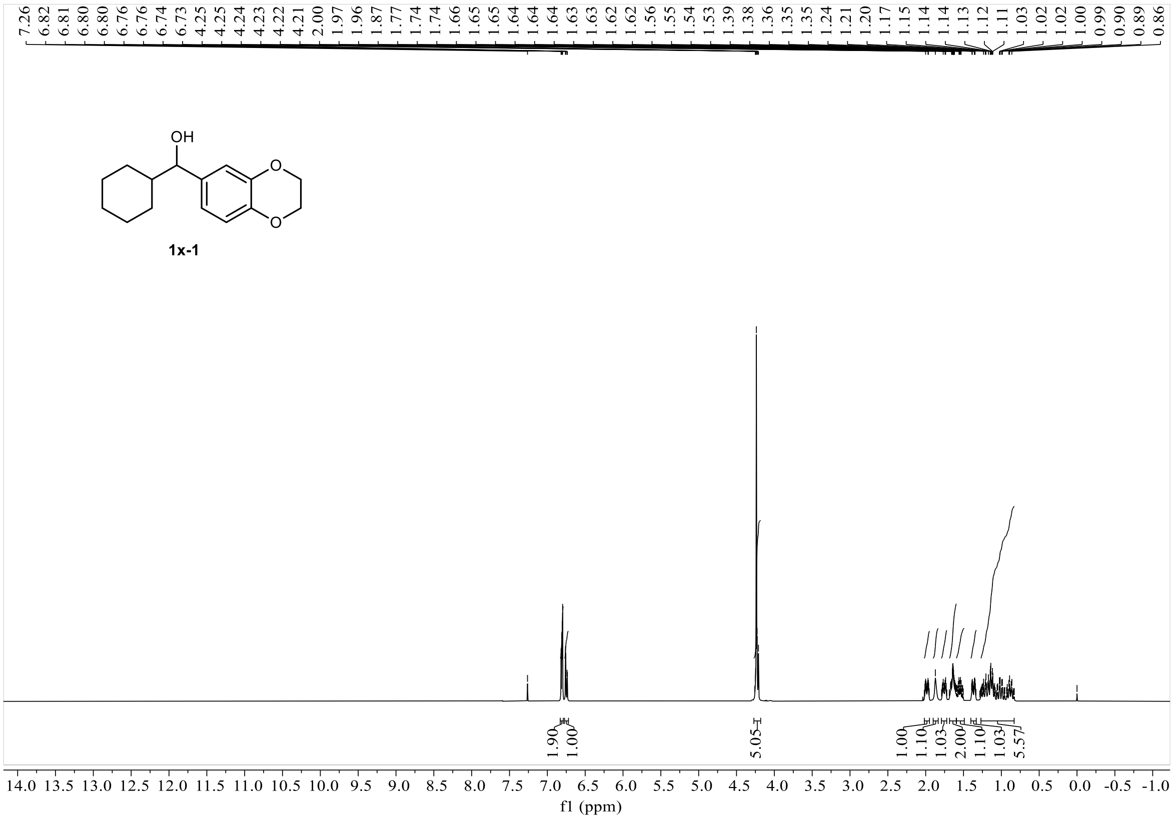

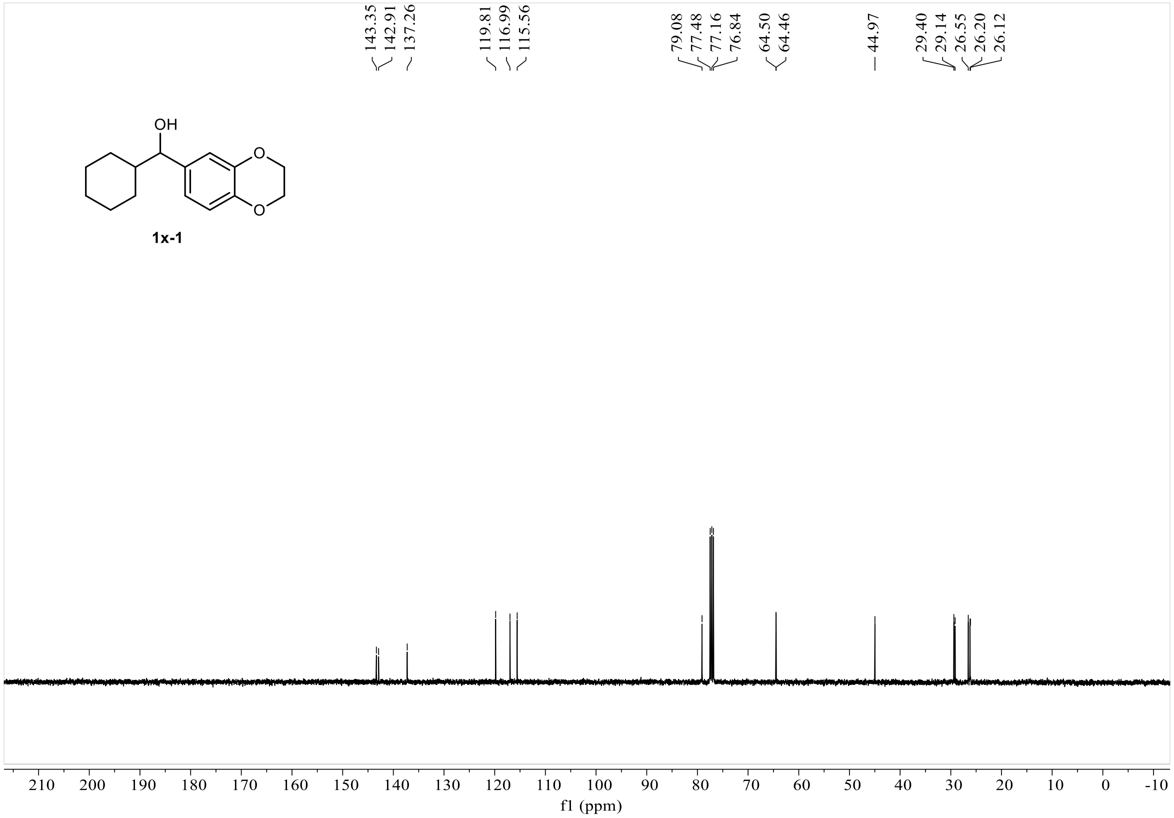

^{1}H NMR (400 MHz, $CDCl_3$) and ^{13}C NMR (101 MHz, $CDCl_3$) spectra of **1x**

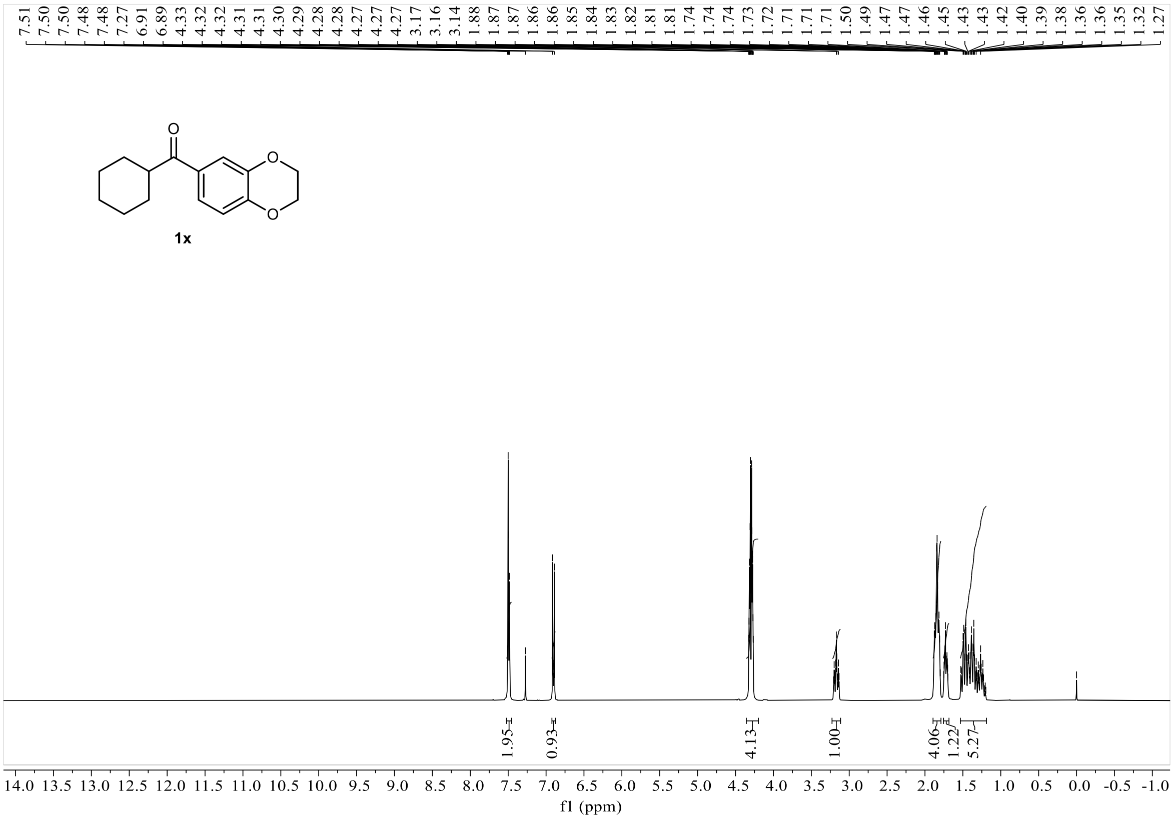

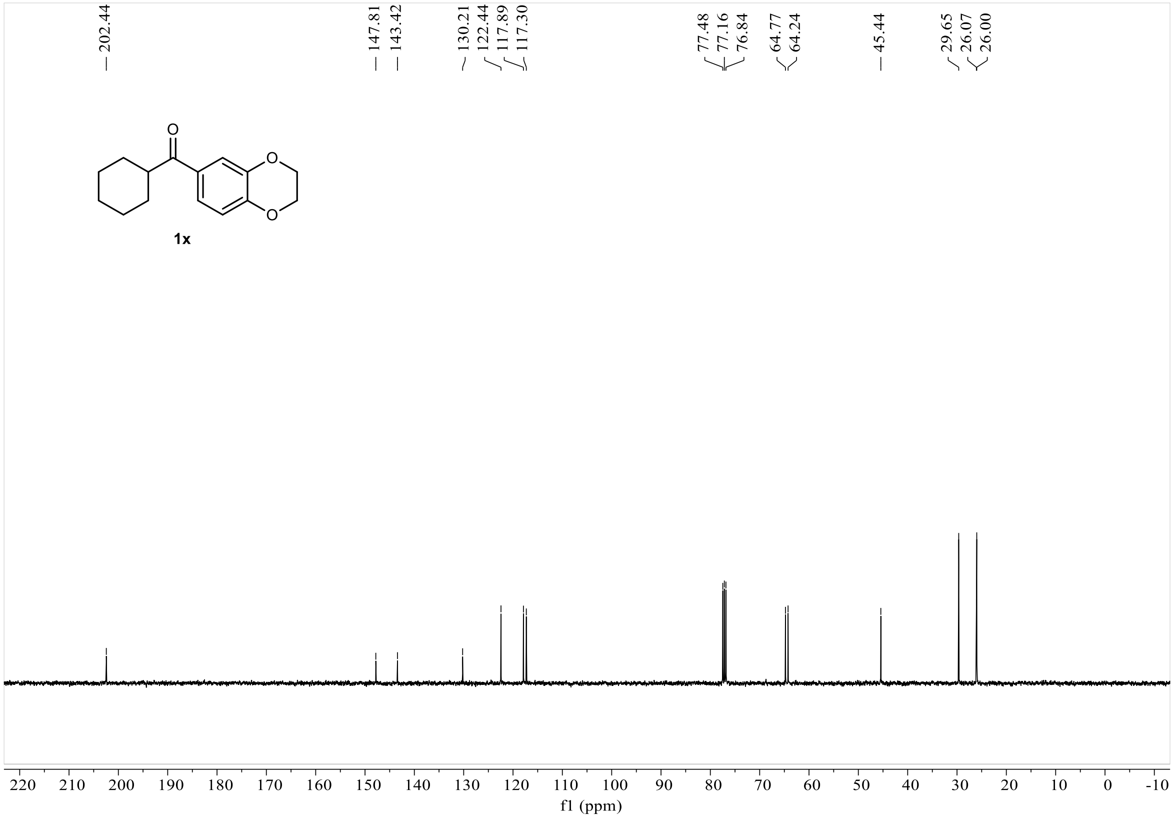

^{1}H NMR (400 MHz, $CDCl_3$) and ^{13}C NMR (101 MHz, $CDCl_3$) spectra of **7c**

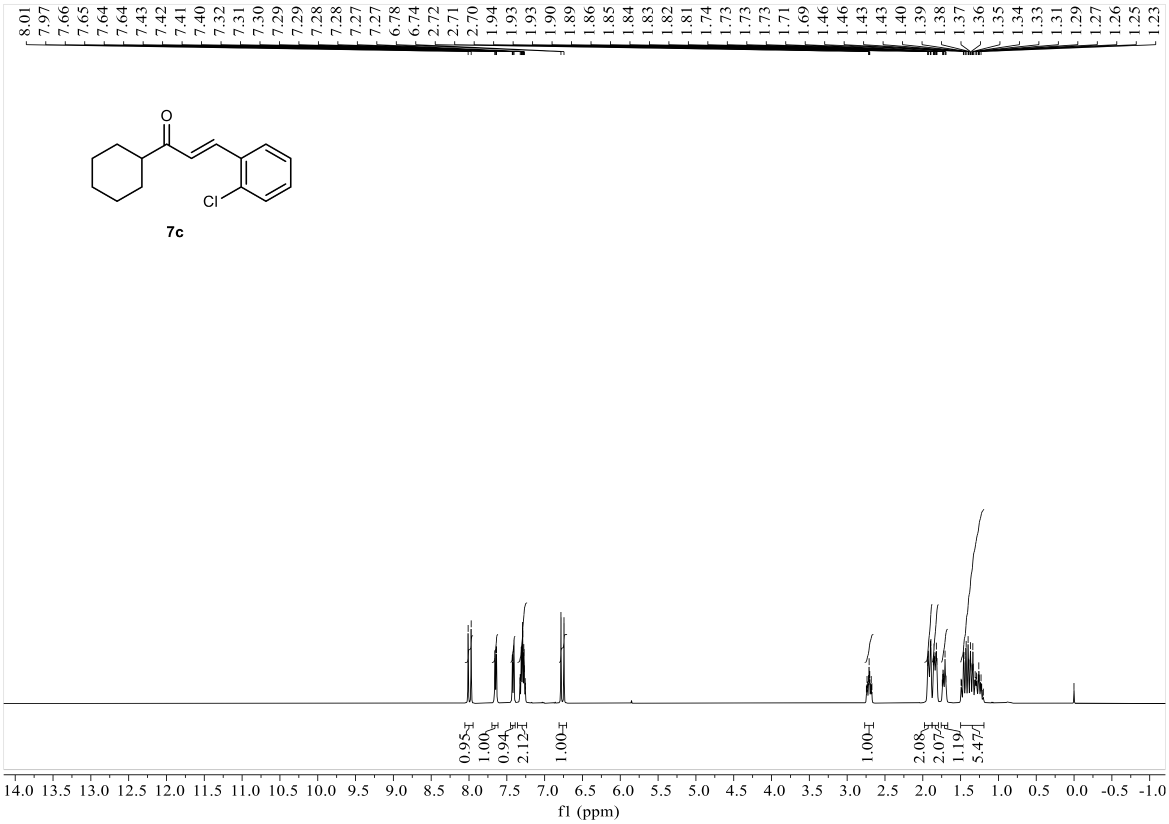

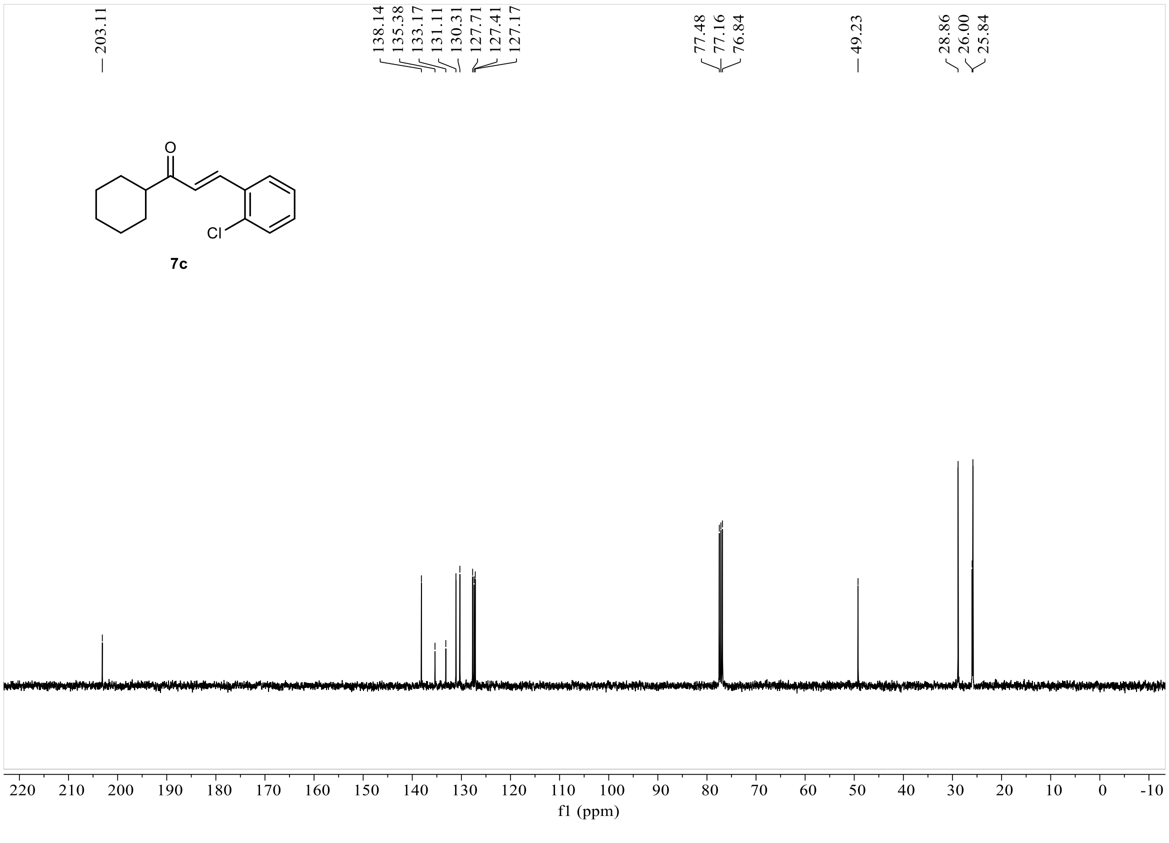

^{1}H NMR (400 MHz, $CDCl_3$) and ^{13}C NMR (101 MHz, $CDCl_3$) spectra of **7d**

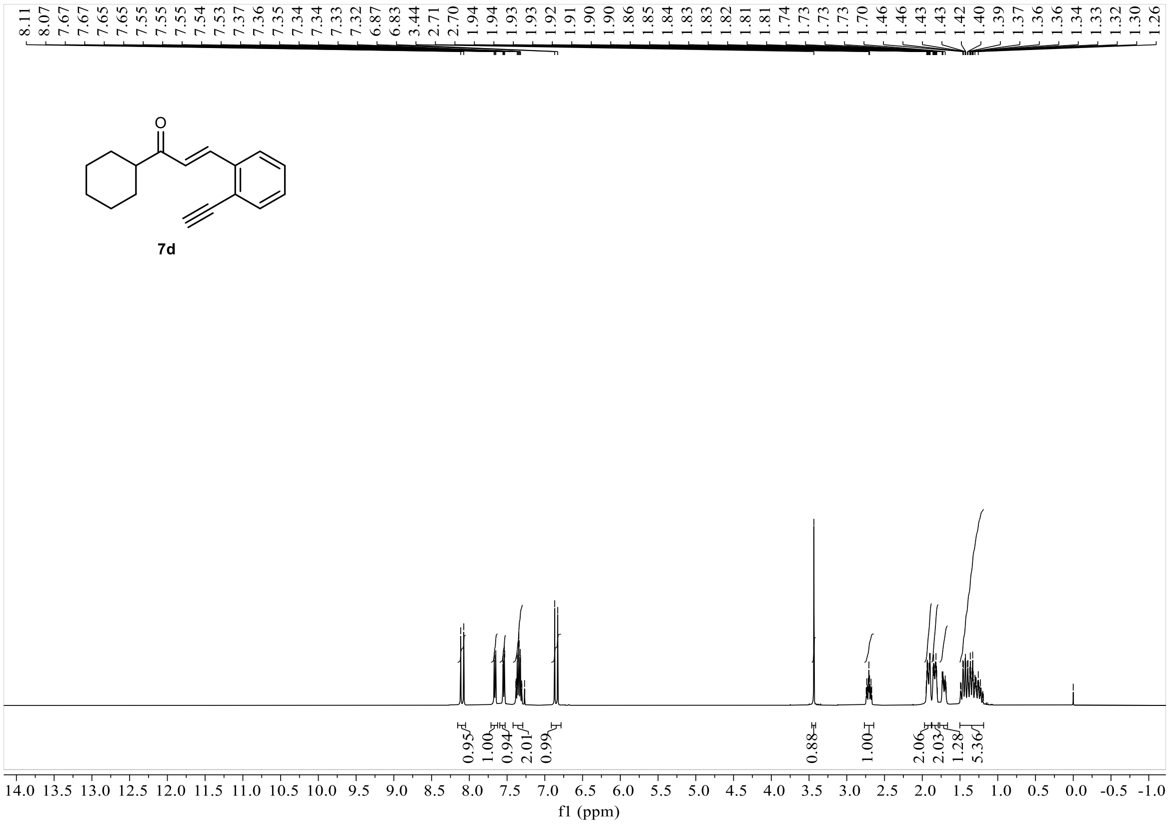

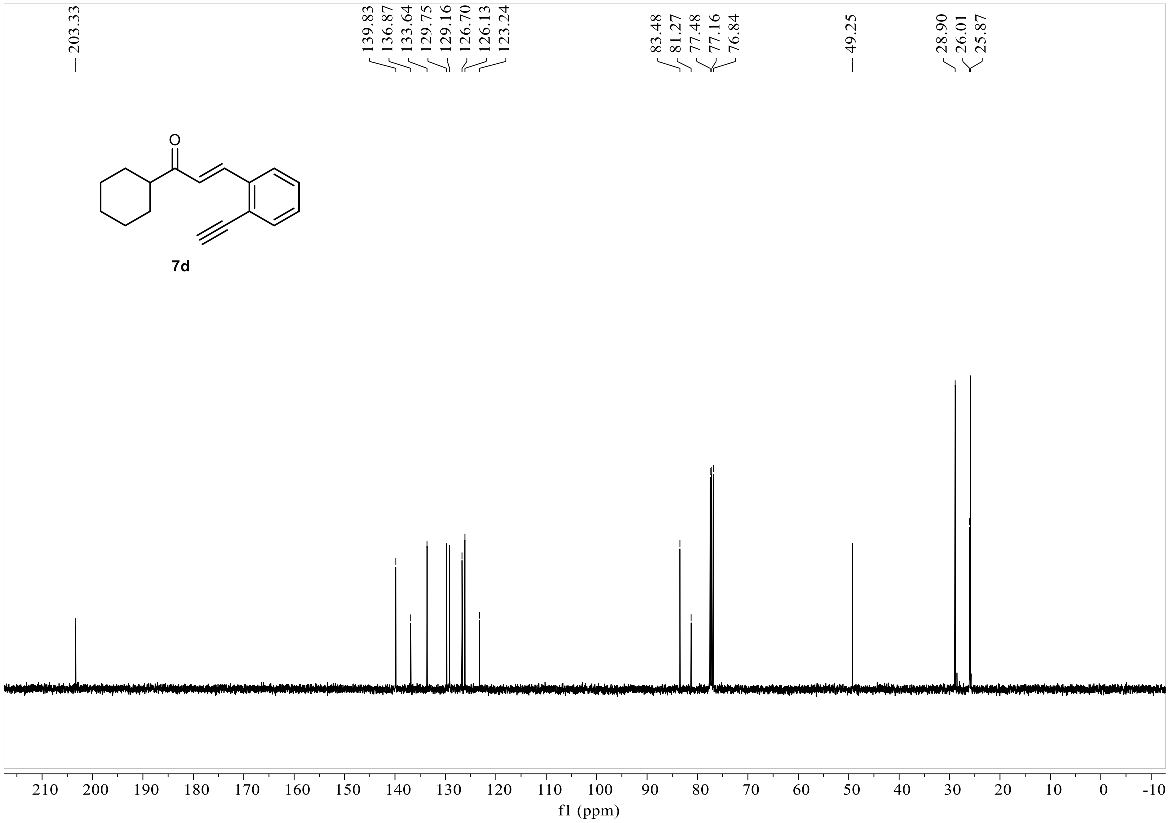

^{1}H NMR (400 MHz, $CDCl_3$) and ^{13}C NMR (101 MHz, $CDCl_3$) spectra of **7f**

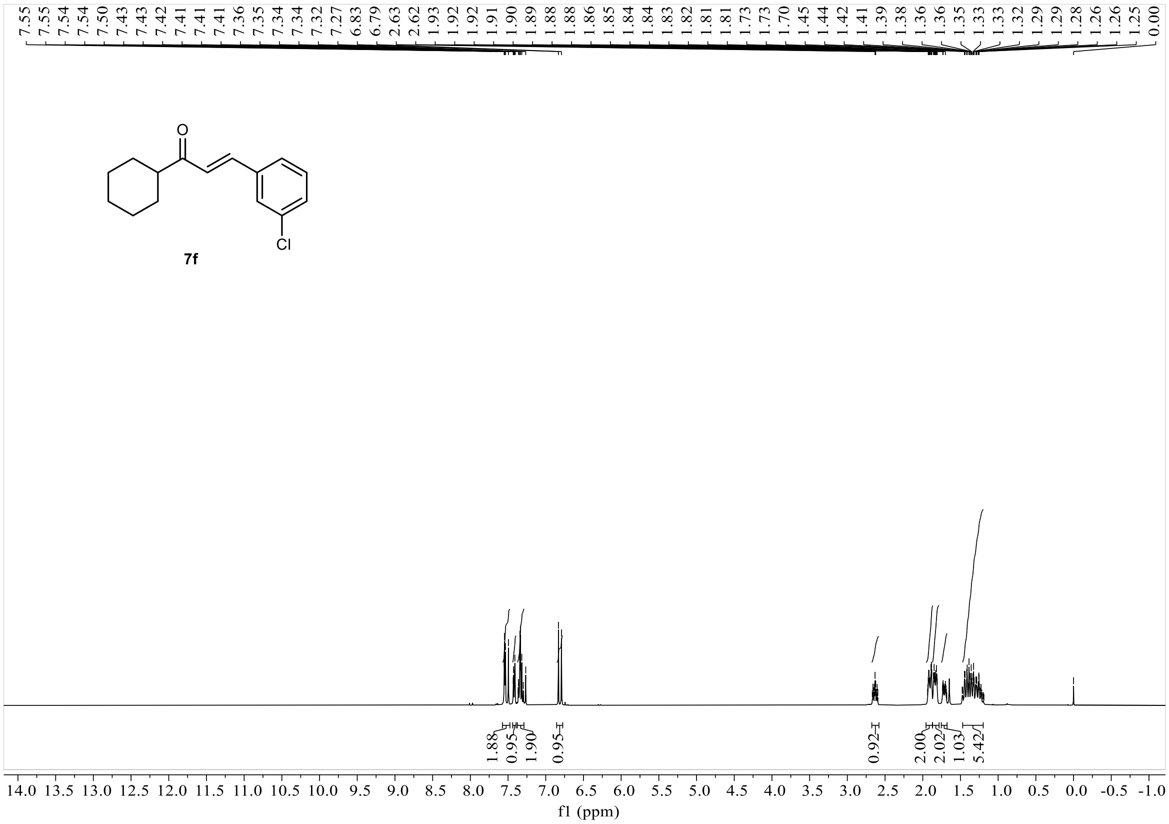

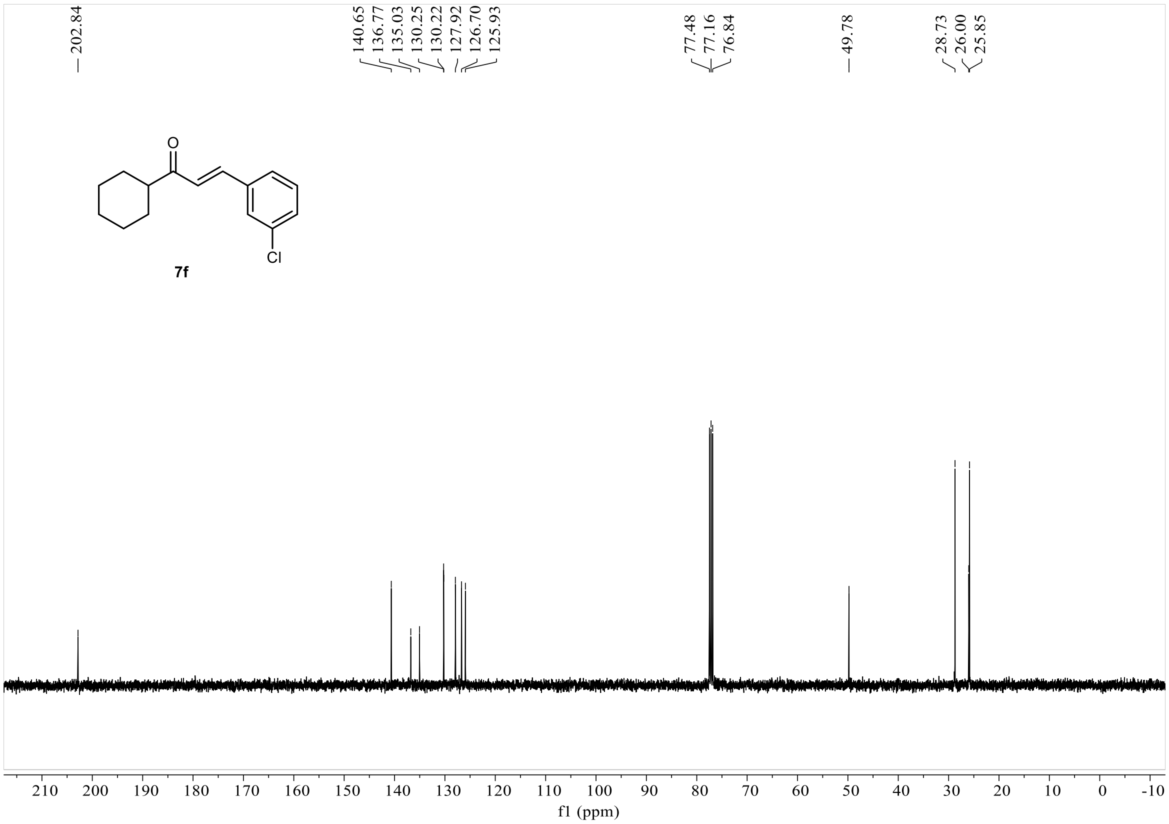

^{1}H NMR (400 MHz, $CDCl_3$) and ^{13}C NMR (101 MHz, $CDCl_3$) spectra of **7i**

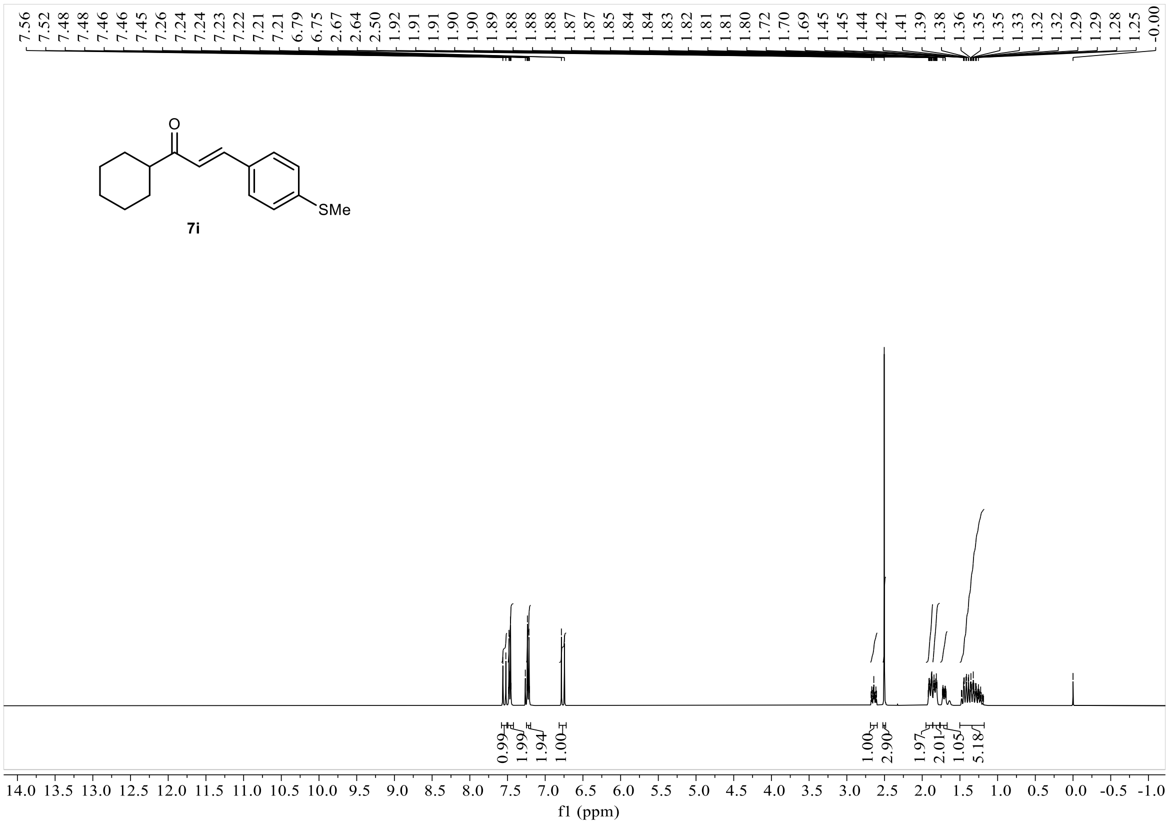

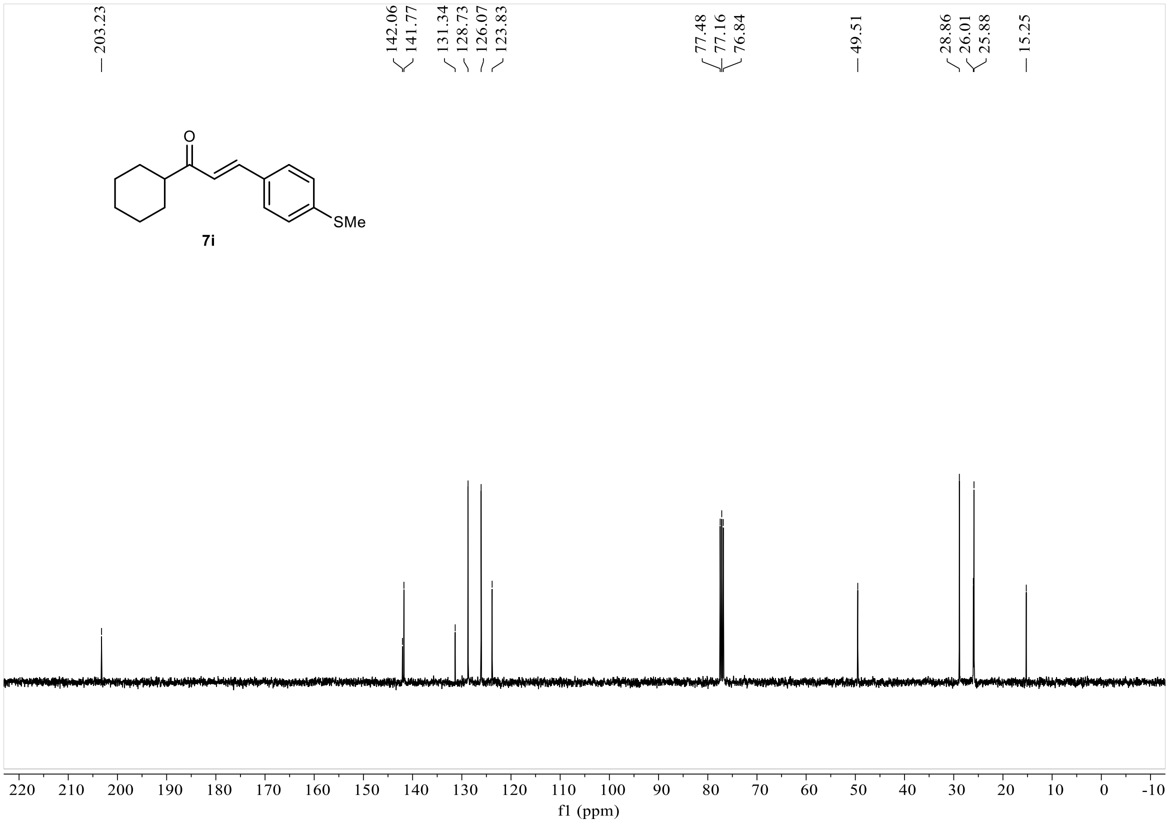

^{1}H NMR (400 MHz, $CDCl_3$) and ^{13}C NMR (101 MHz, $CDCl_3$) spectra of **7j**

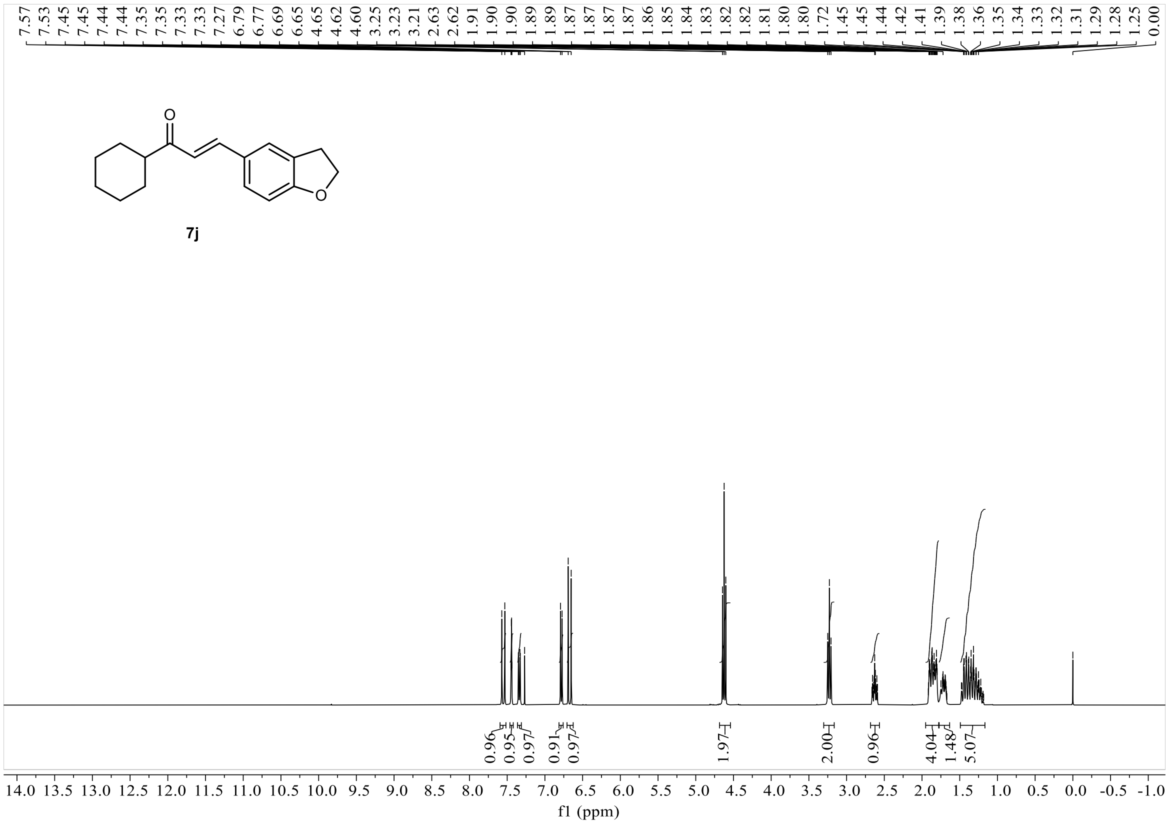

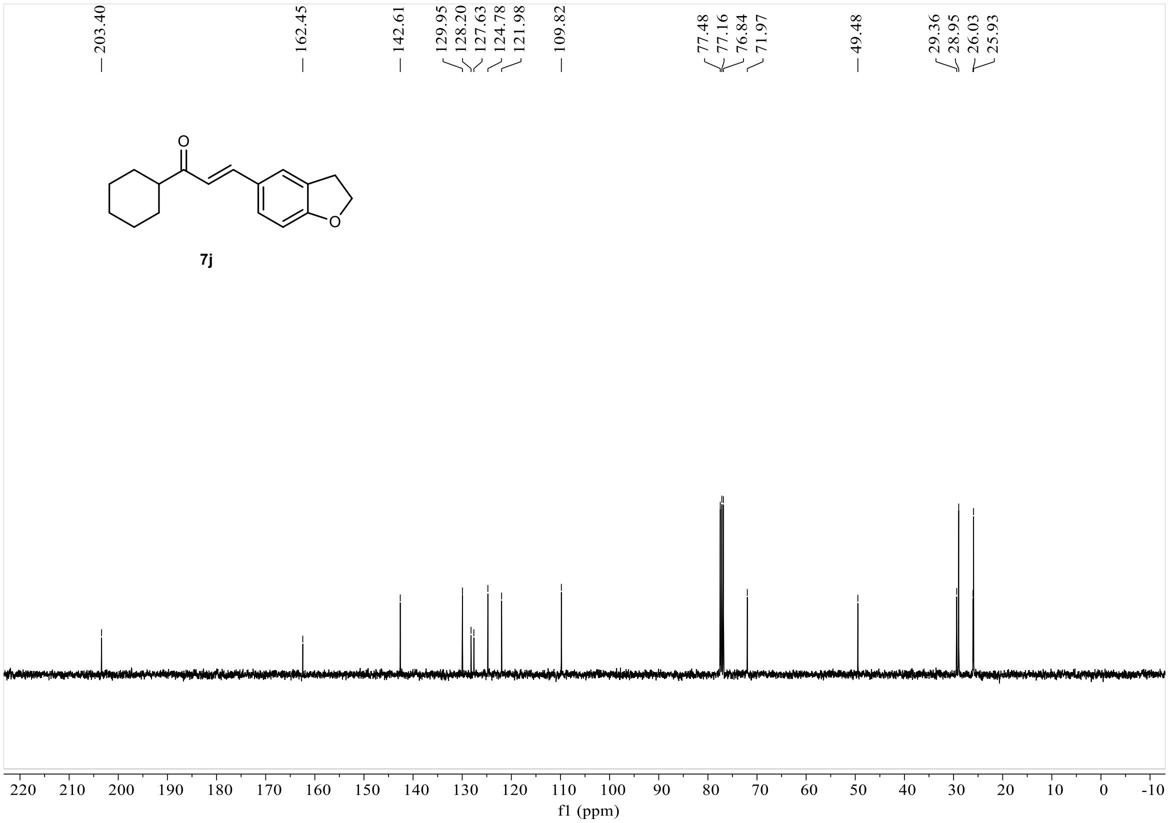

^{1}H NMR (400 MHz, $CDCl_3$) and ^{13}C NMR (101 MHz, $CDCl_3$) spectra of **7k**

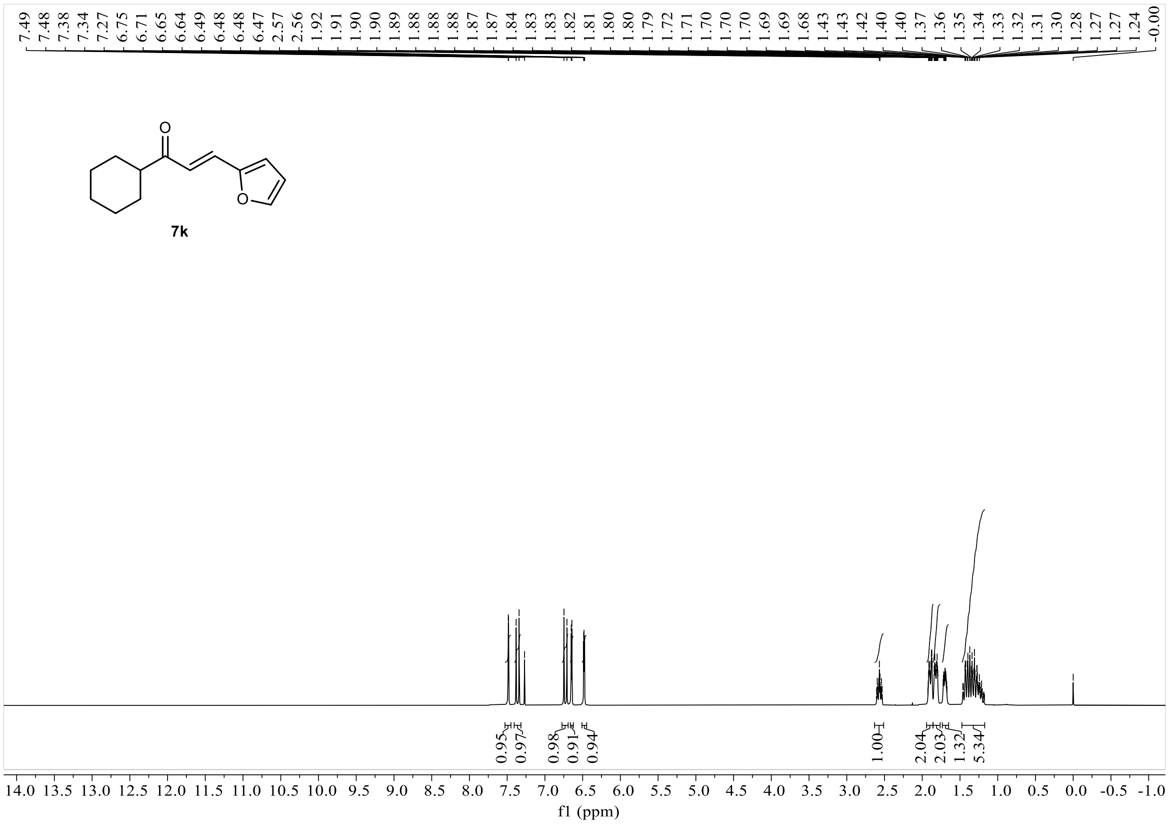

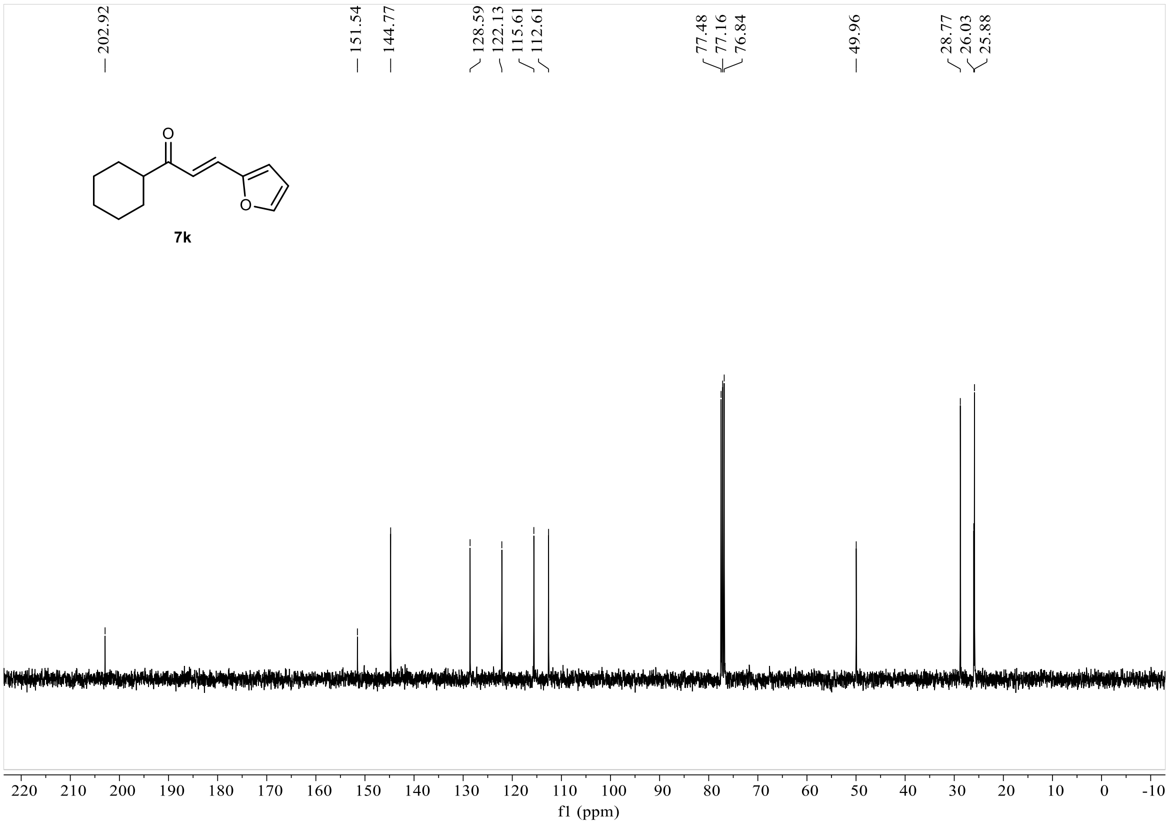

^{1}H NMR (400 MHz, $CDCl_3$) and ^{13}C NMR (101 MHz, $CDCl_3$) spectra of **7m**

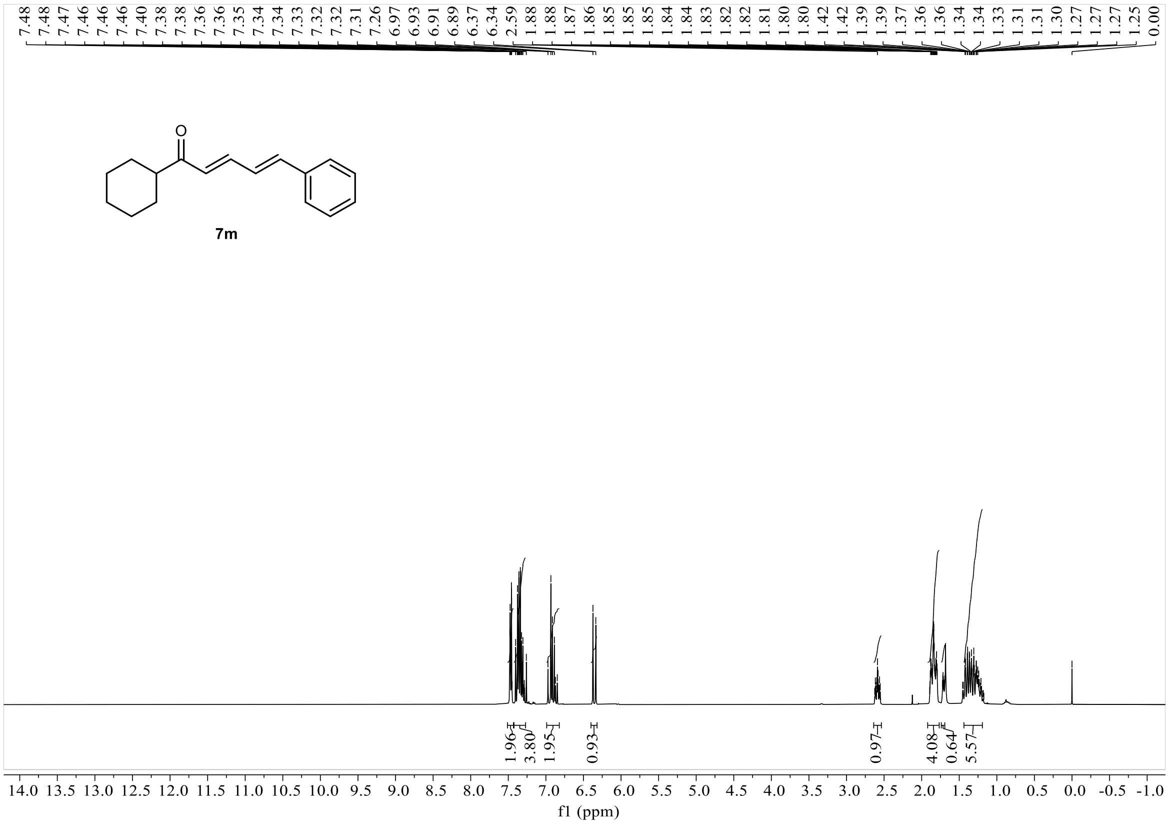

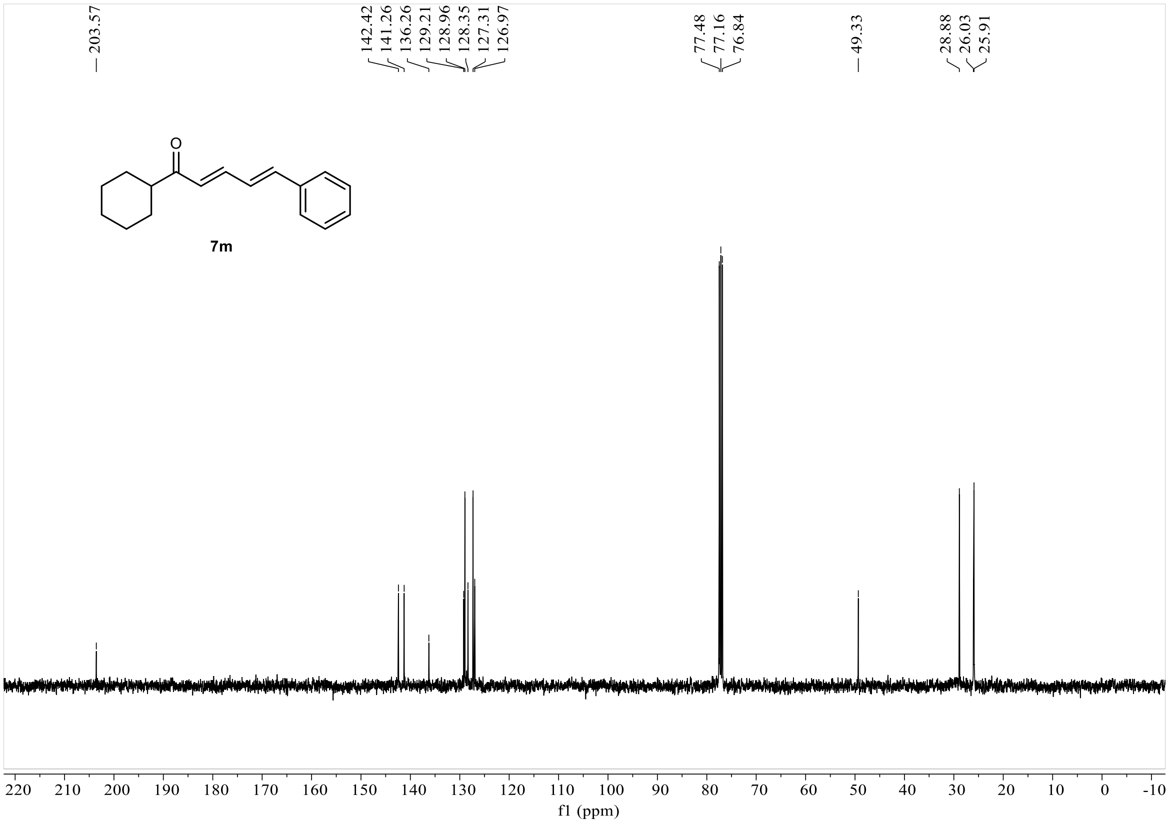

^{1}H NMR (400 MHz, $CDCl_3$) and ^{13}C NMR (101 MHz, $CDCl_3$) spectra of **7n**

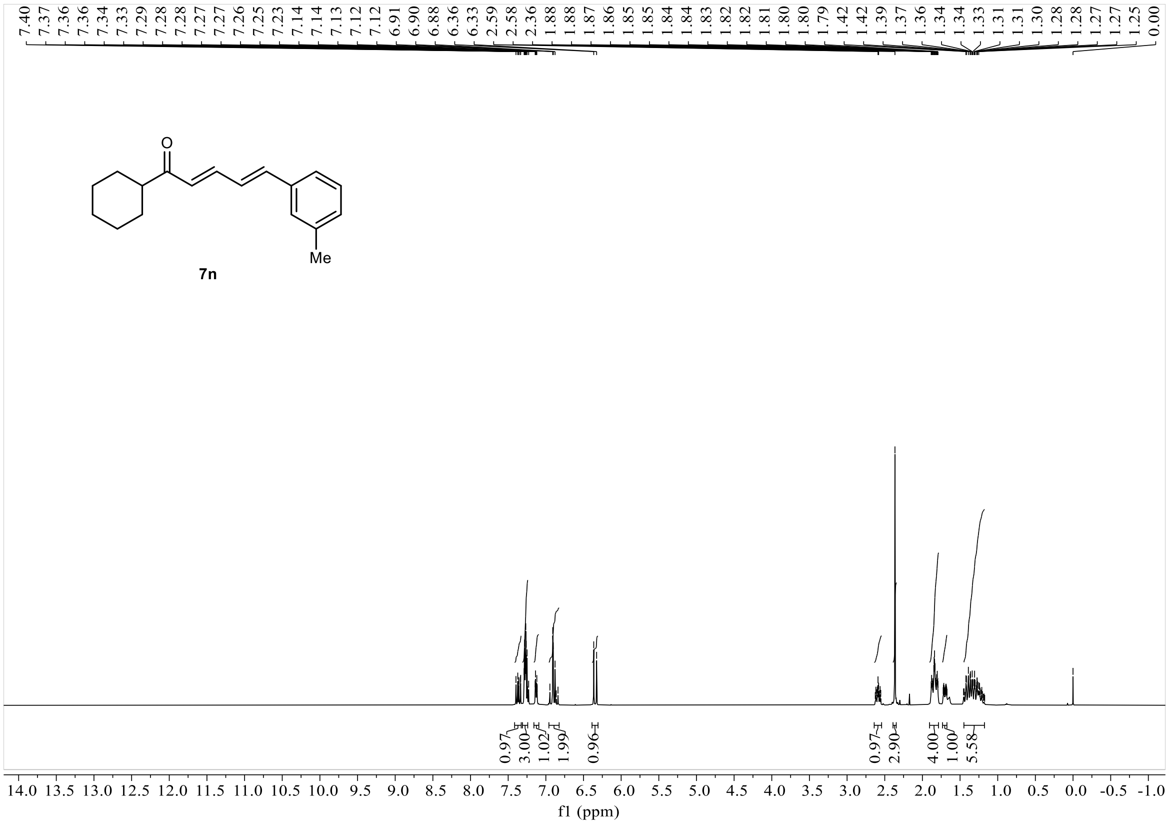

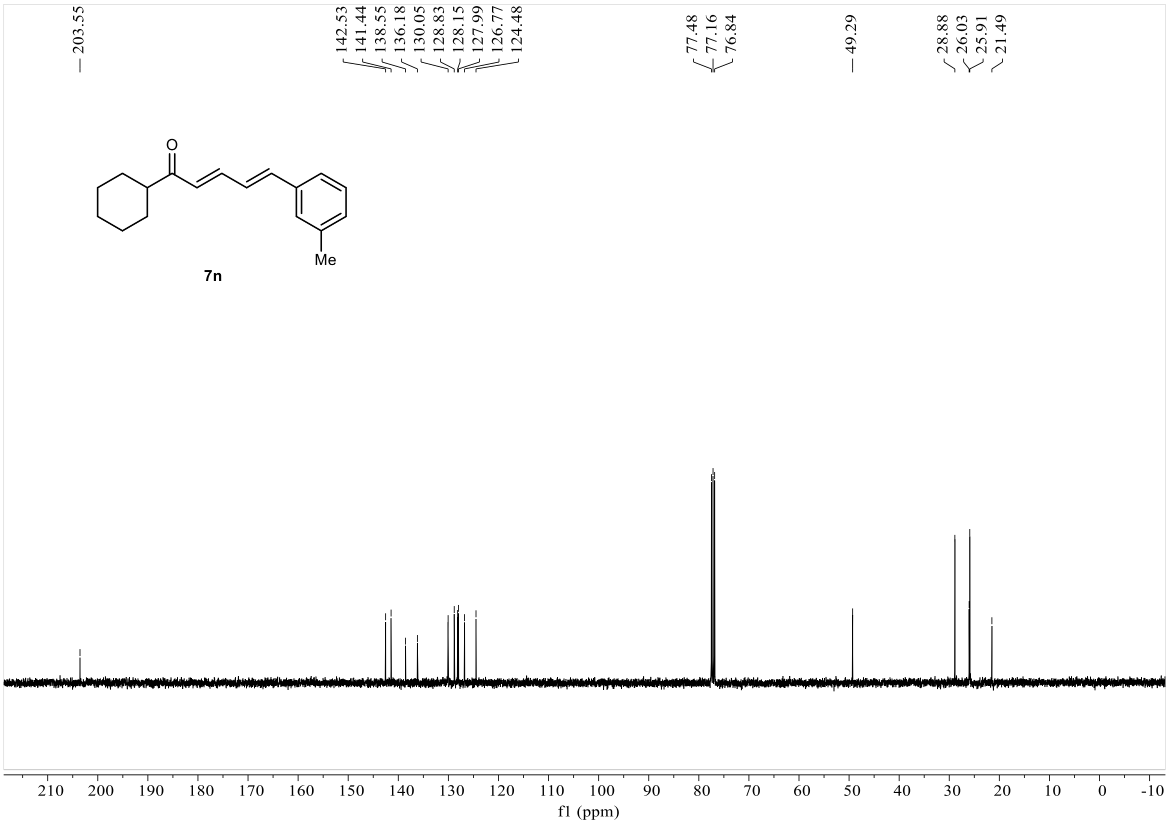

^{1}H NMR (400 MHz, $CDCl_3$) and ^{13}C NMR (101 MHz, $CDCl_3$) spectra of **7o**

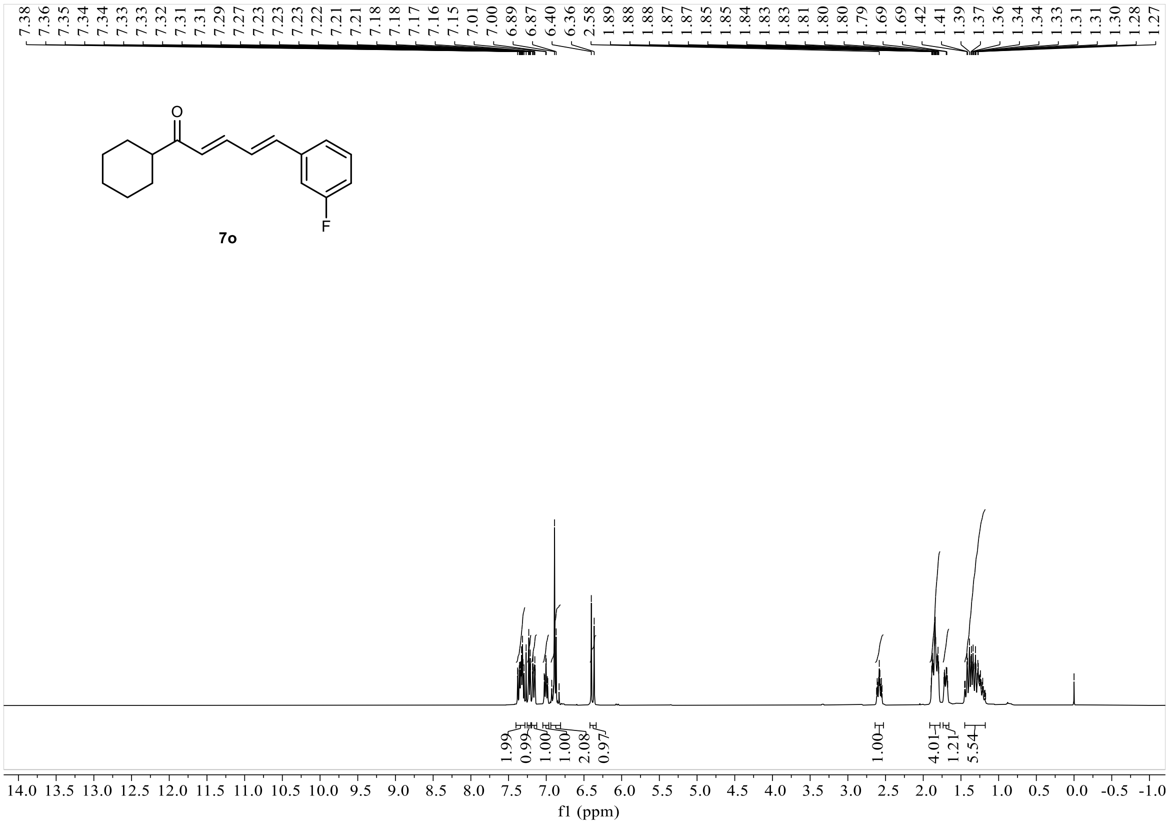

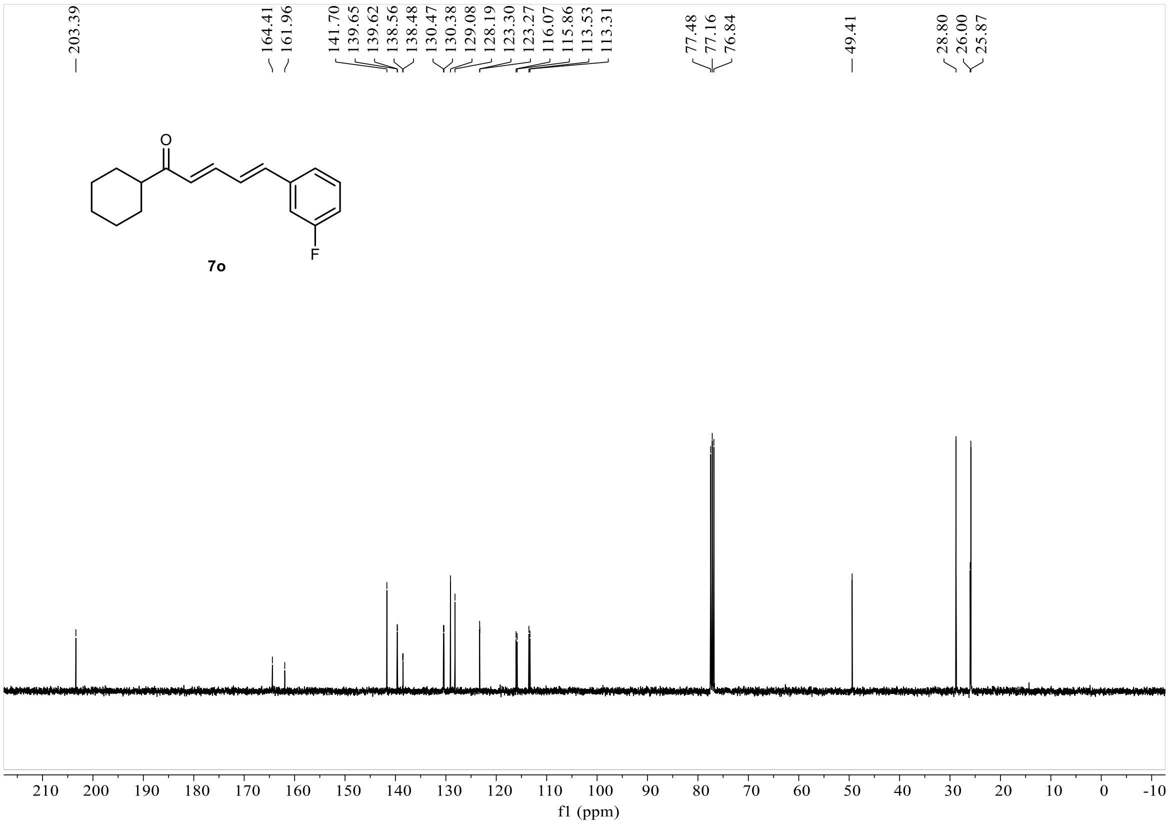

^{19}F NMR (376 MHz, $CDCl_3$) spectrum of **7o**

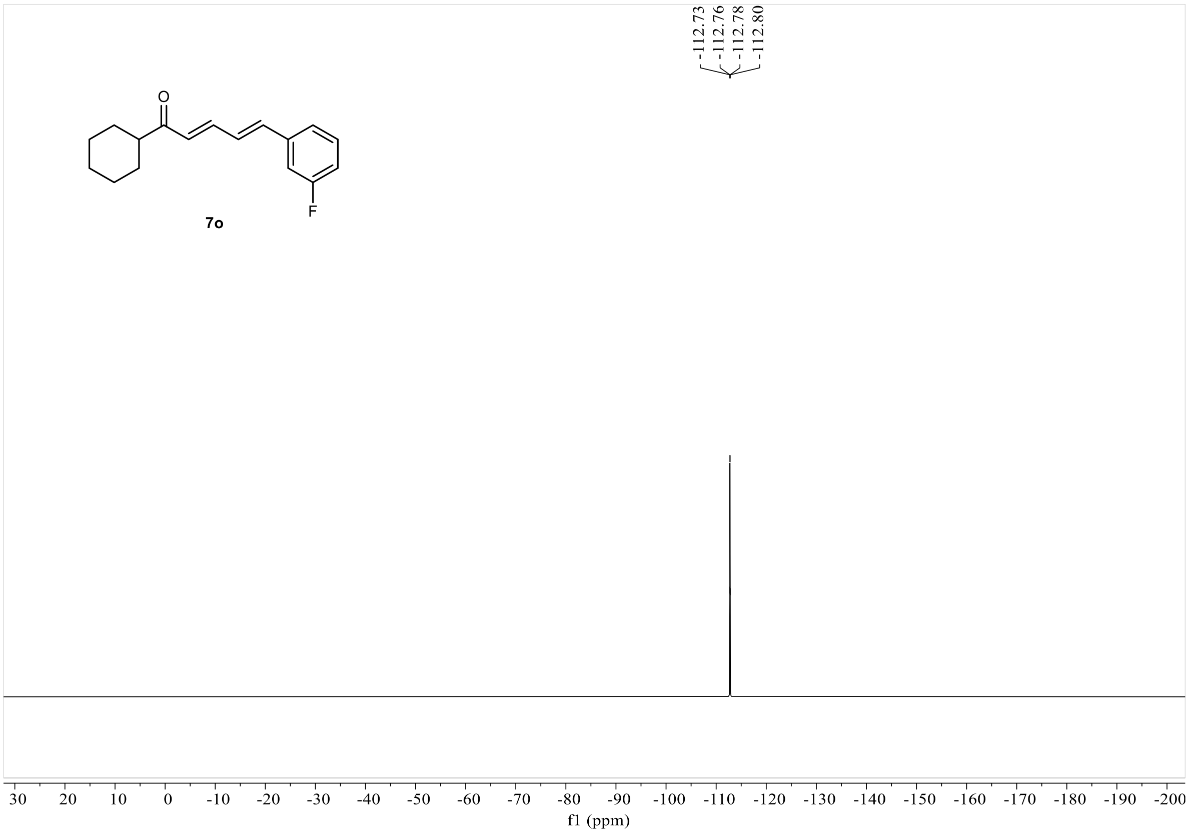

^{1}H NMR (400 MHz, $CDCl_3$) and ^{13}C NMR (101 MHz, $CDCl_3$) spectra of **7p**

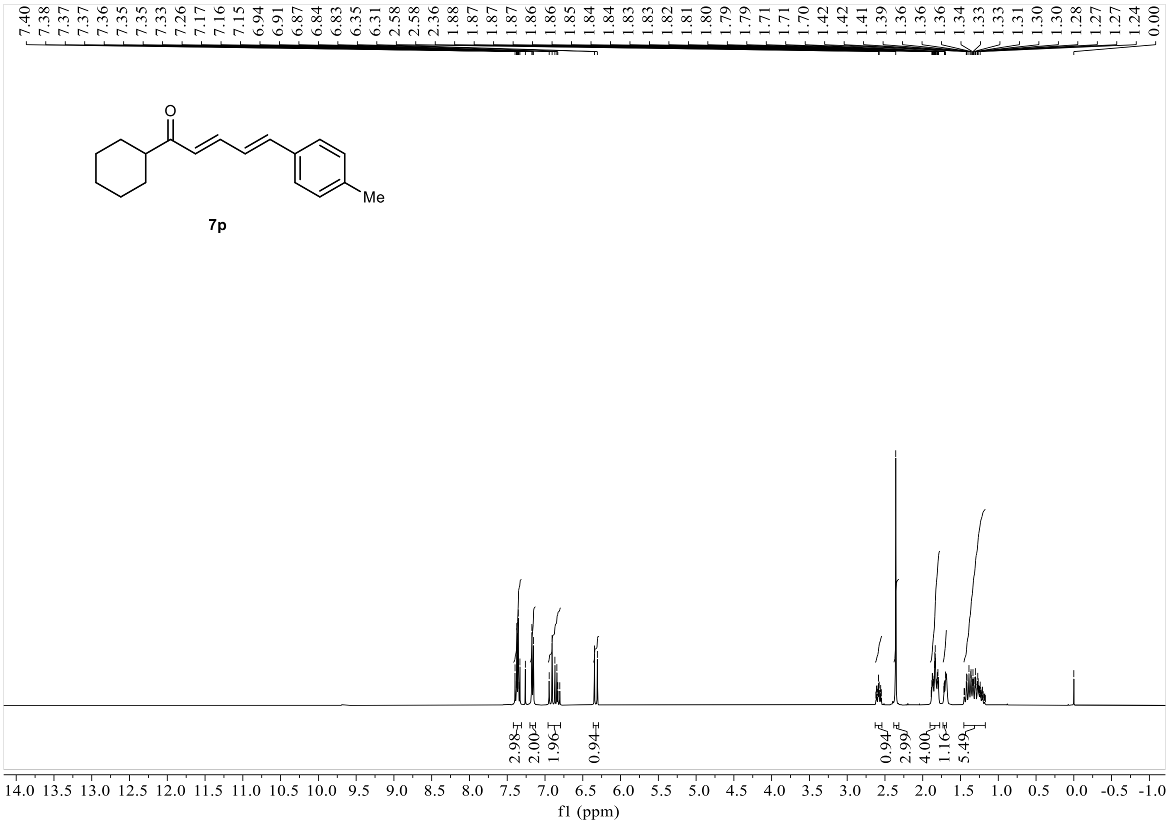

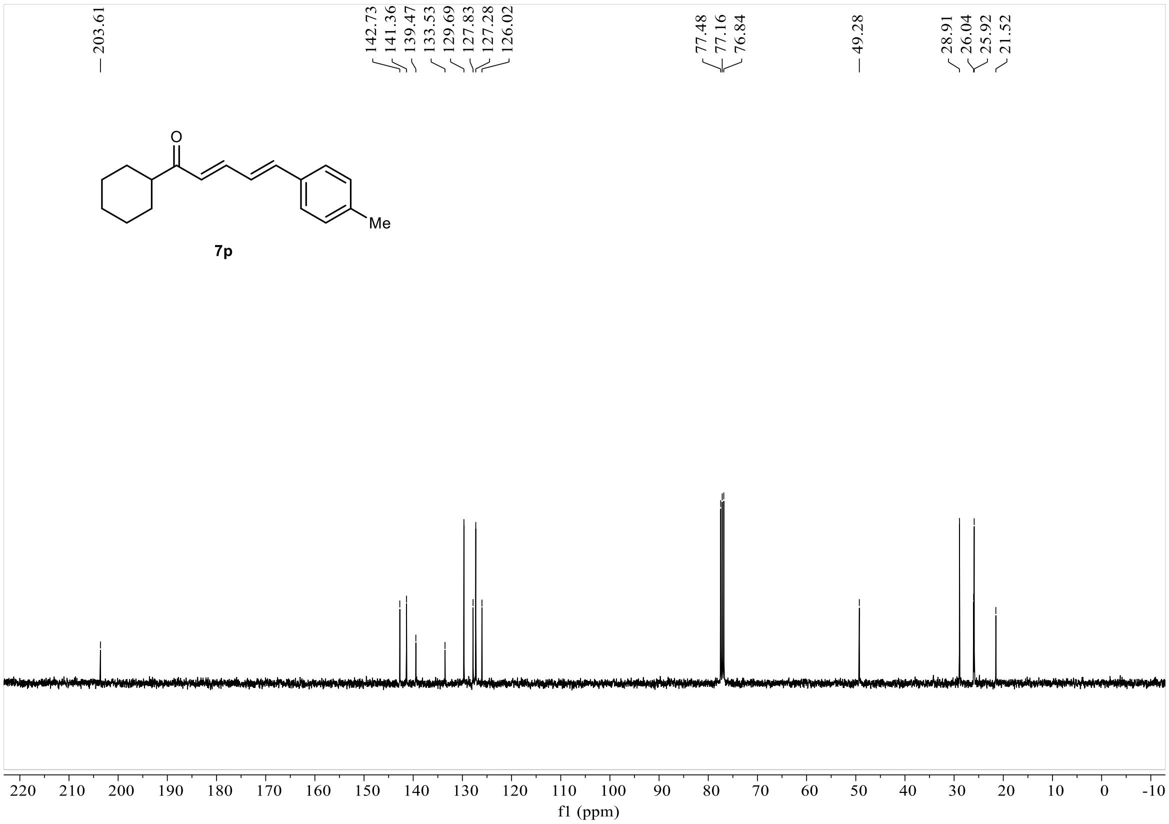

^{1}H NMR (400 MHz, $CDCl_3$) and ^{13}C NMR (101 MHz, $CDCl_3$) spectra of **7q**

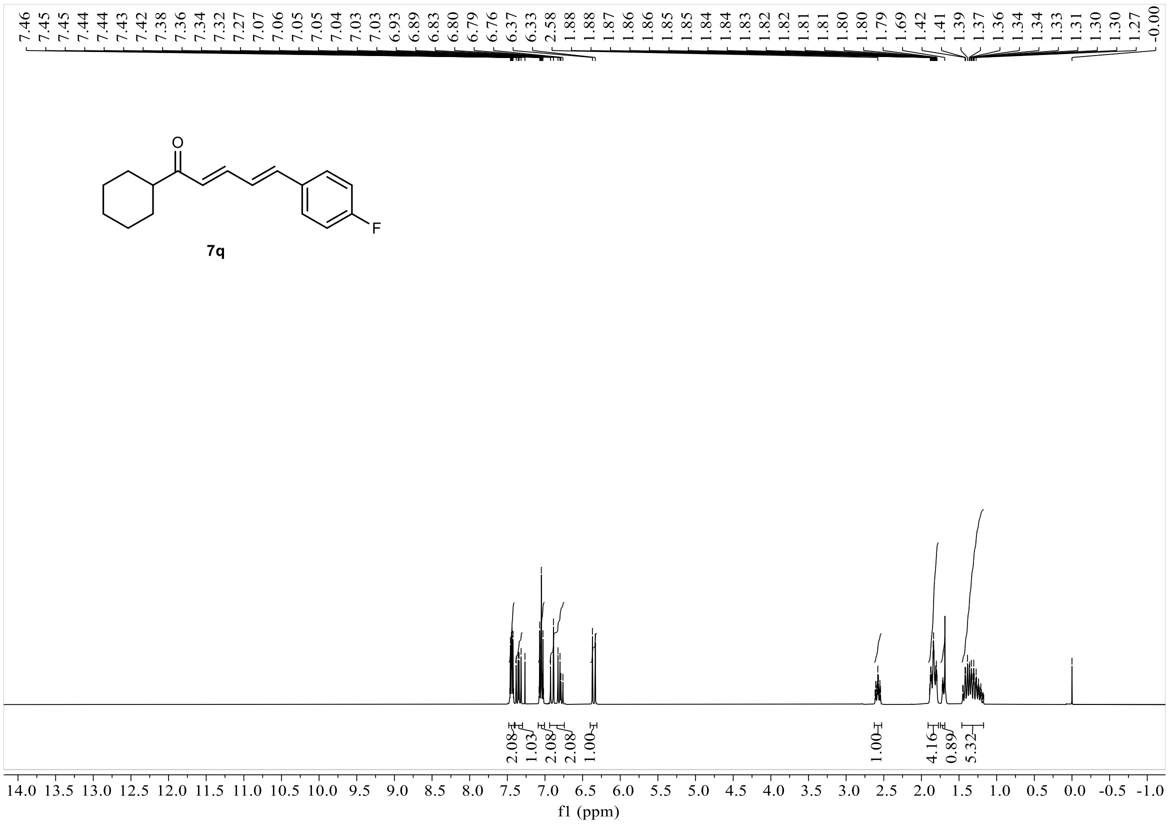

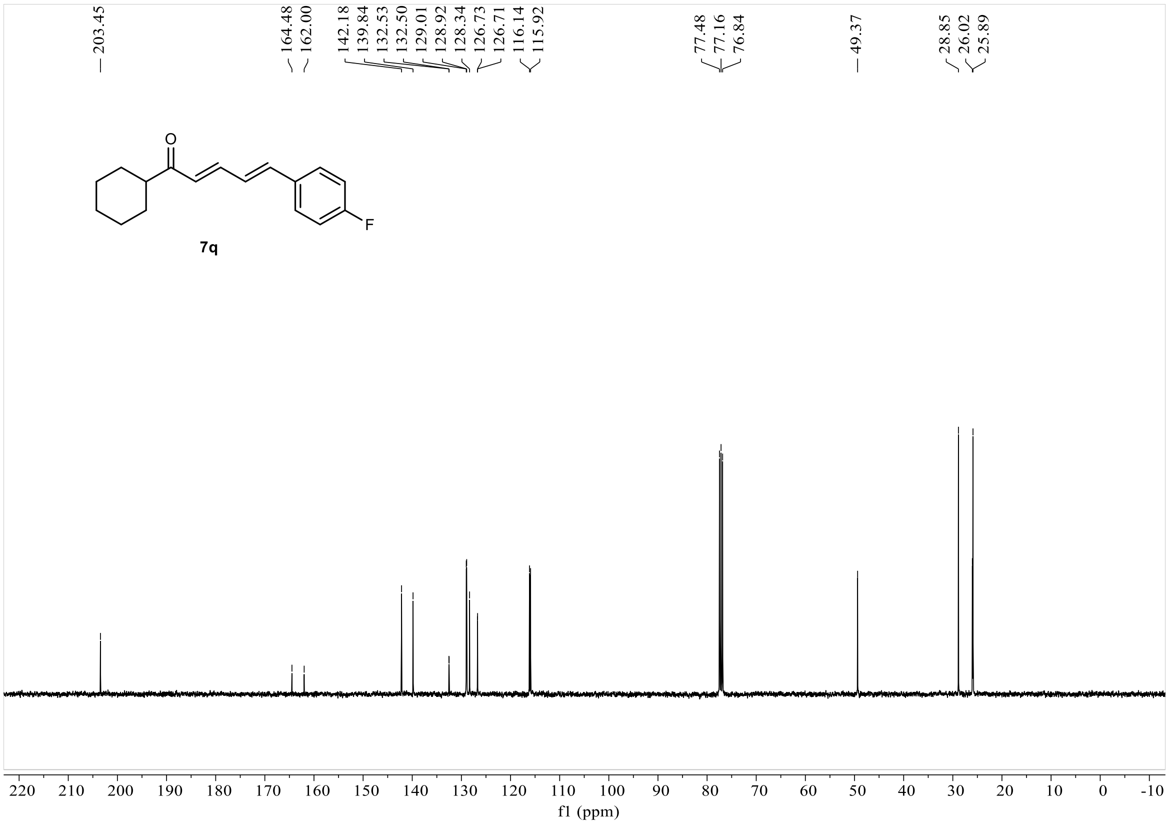

^{19}F NMR (376 MHz, $CDCl_3$) spectrum of **7q**

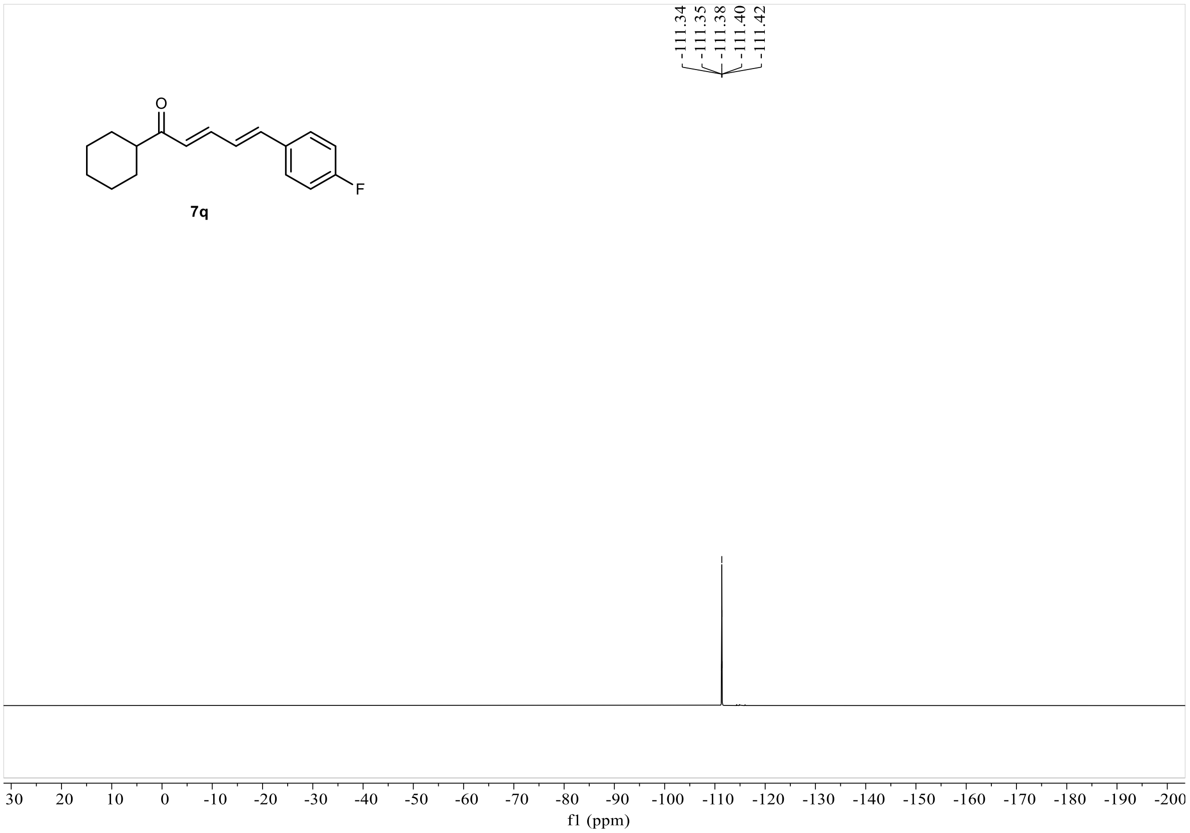

^{1}H NMR (400 MHz, $CDCl_3$) and ^{13}C NMR (101 MHz, $CDCl_3$) spectra of **7r**

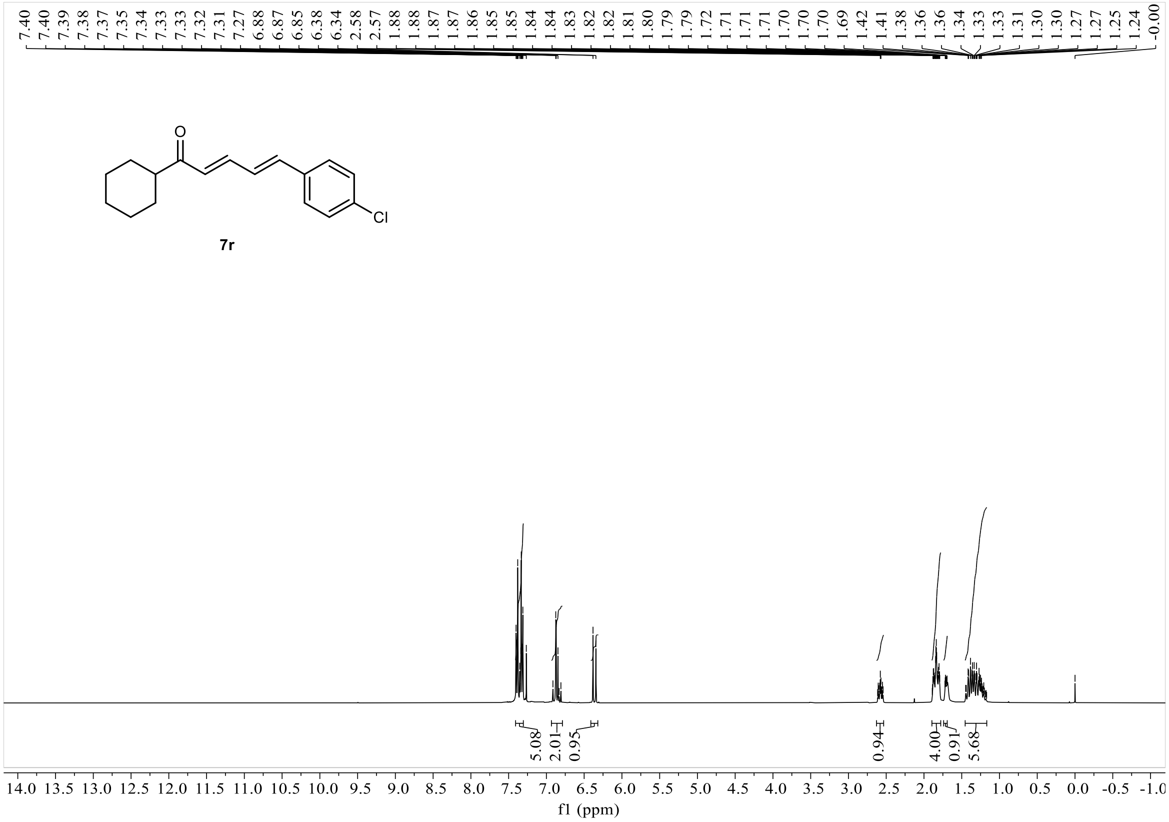

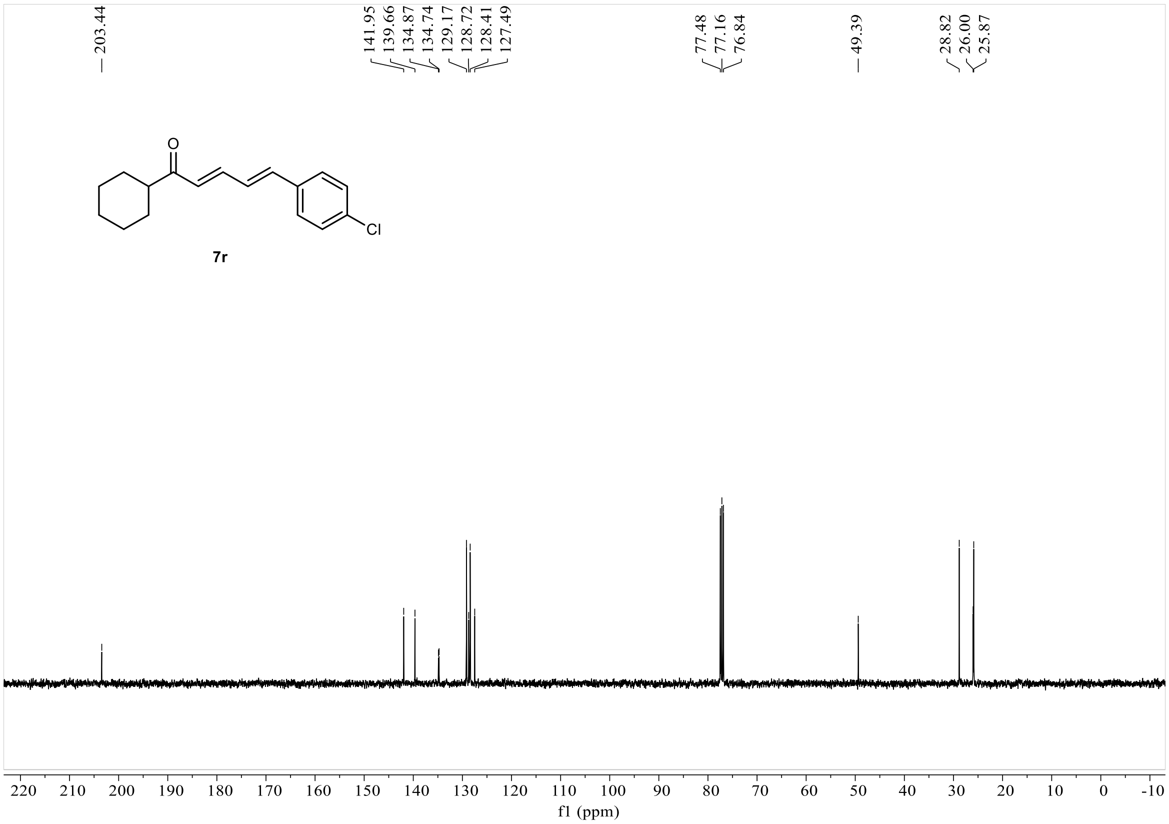

^{1}H NMR (400 MHz, $CDCl_3$) and ^{13}C NMR (101 MHz, $CDCl_3$) spectra of **2a**

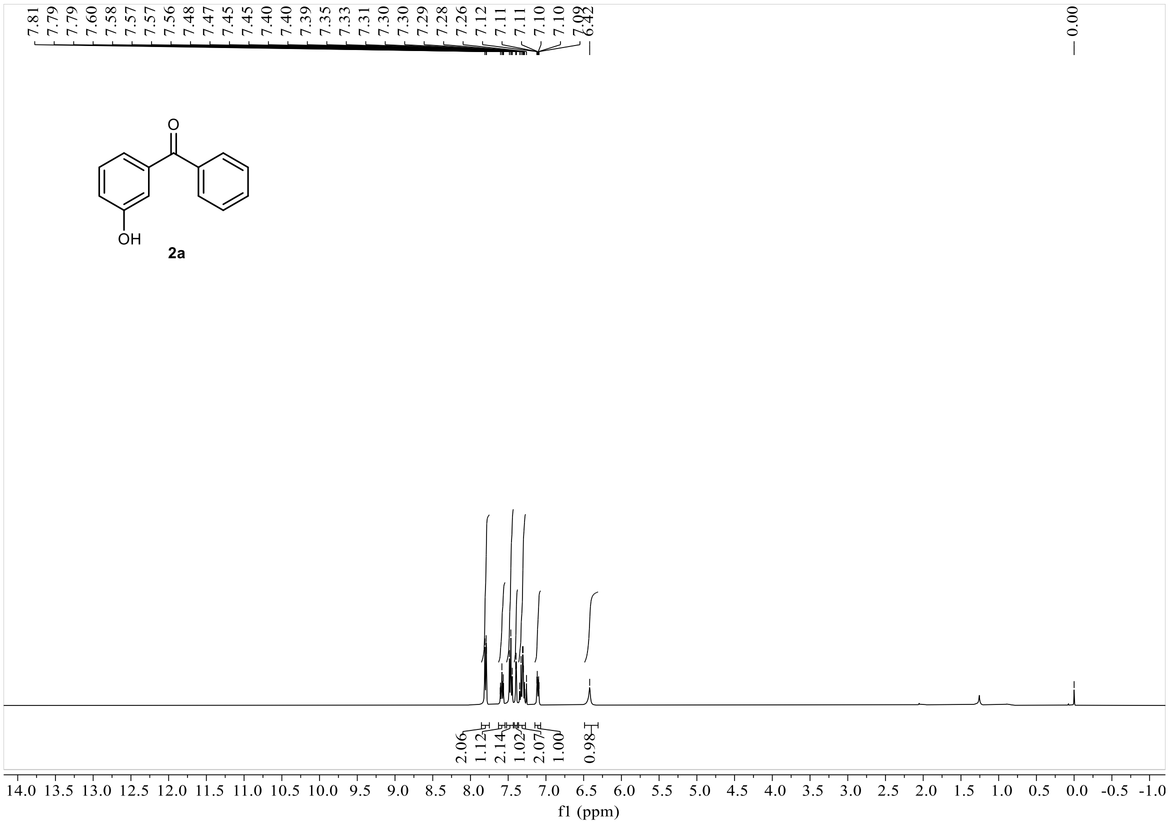

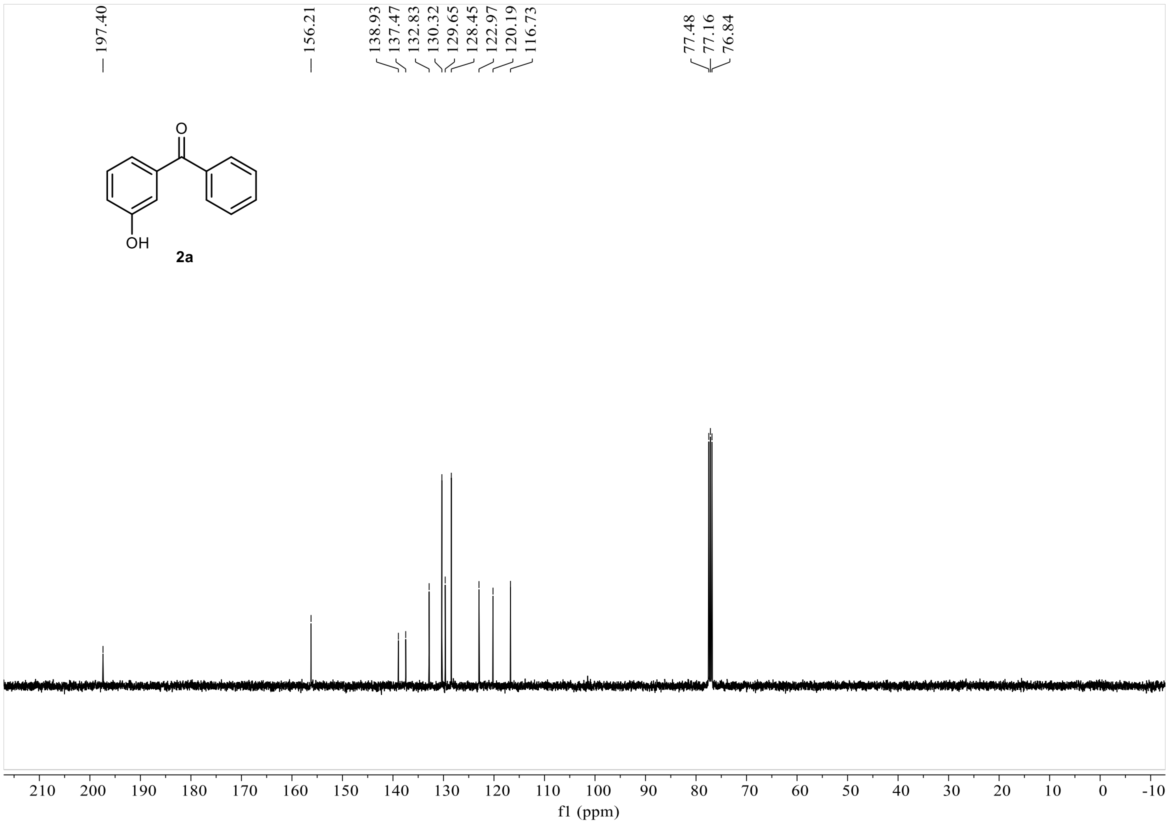

^{1}H NMR (400 MHz, $CDCl_3$) and ^{13}C NMR (101 MHz, $CDCl_3$) spectra of **2b**

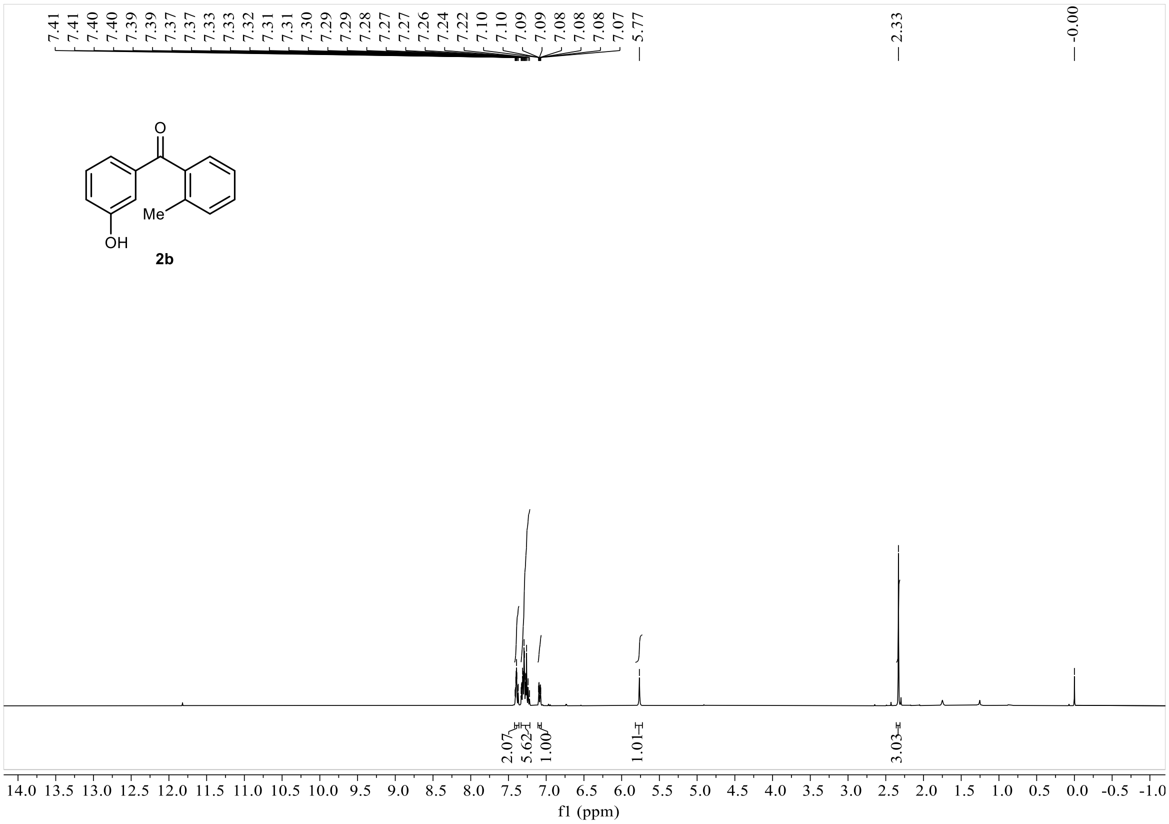

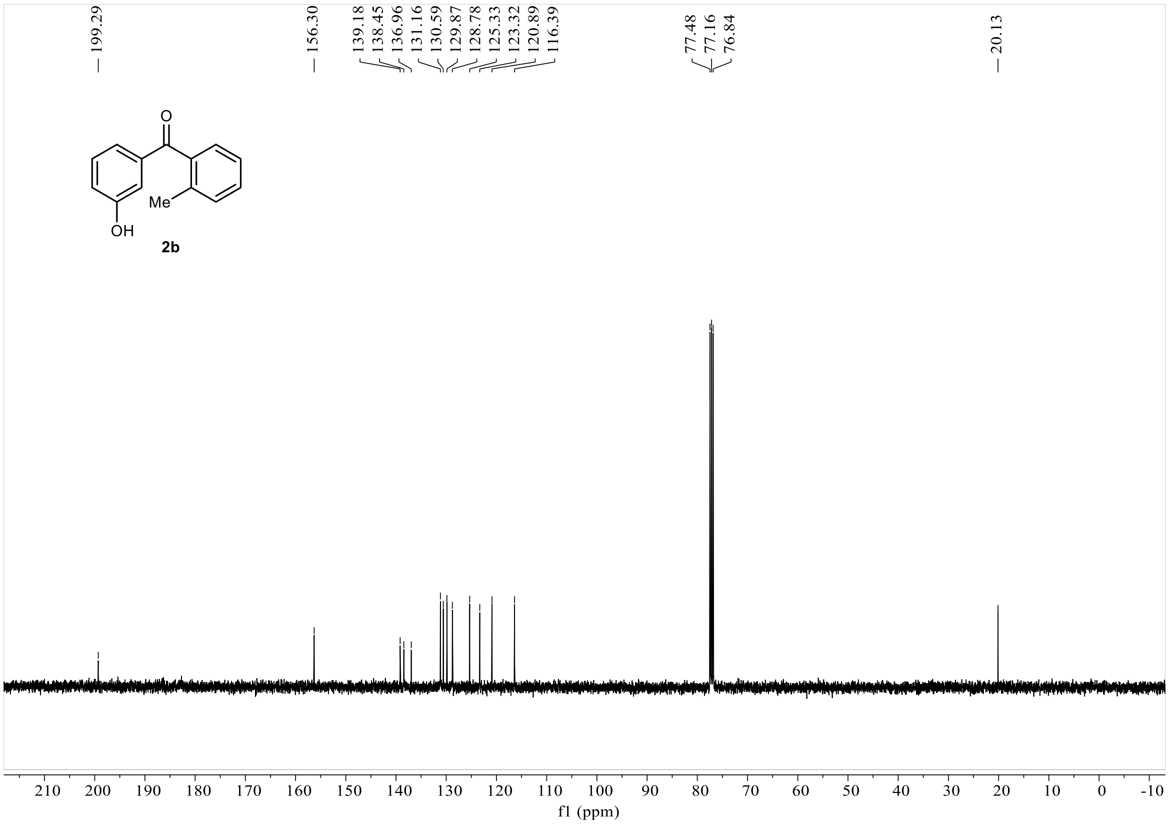

^1H NMR (400 MHz, $CDCl_3$) and ^{13}C NMR (101 MHz, $CDCl_3$) spectra of **2c**

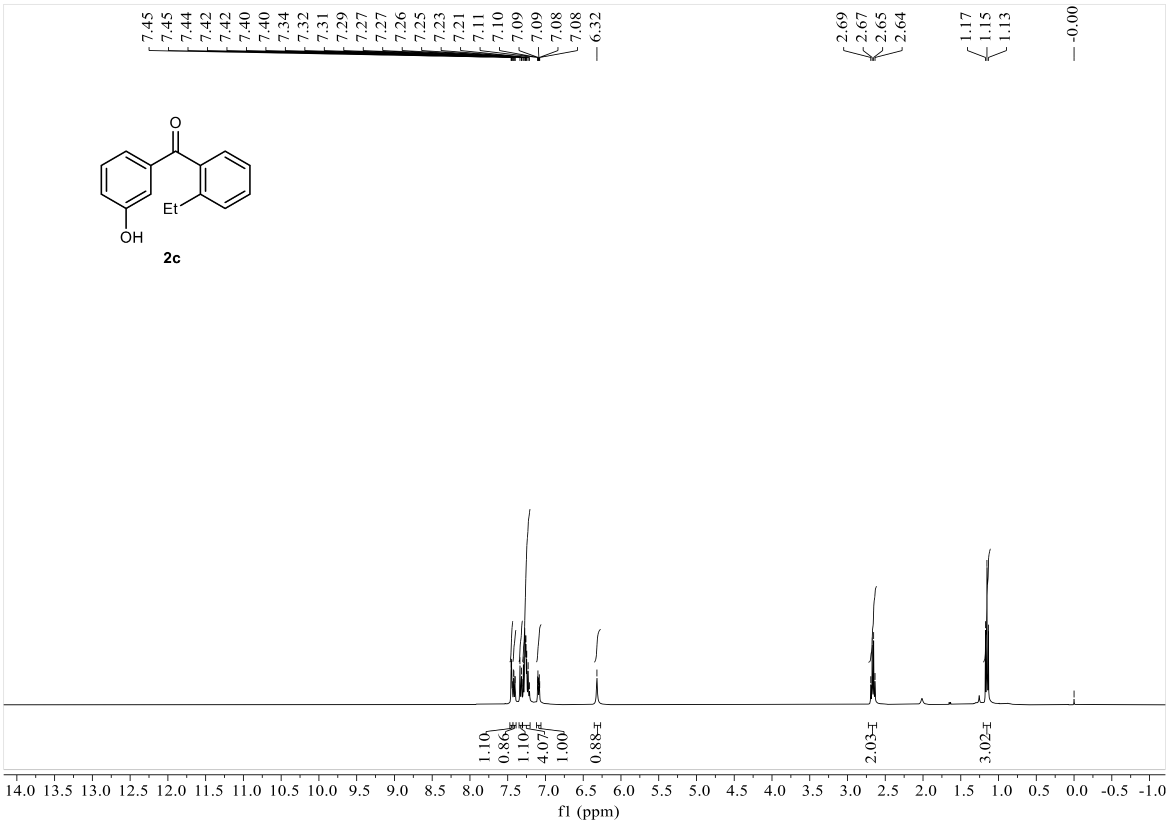

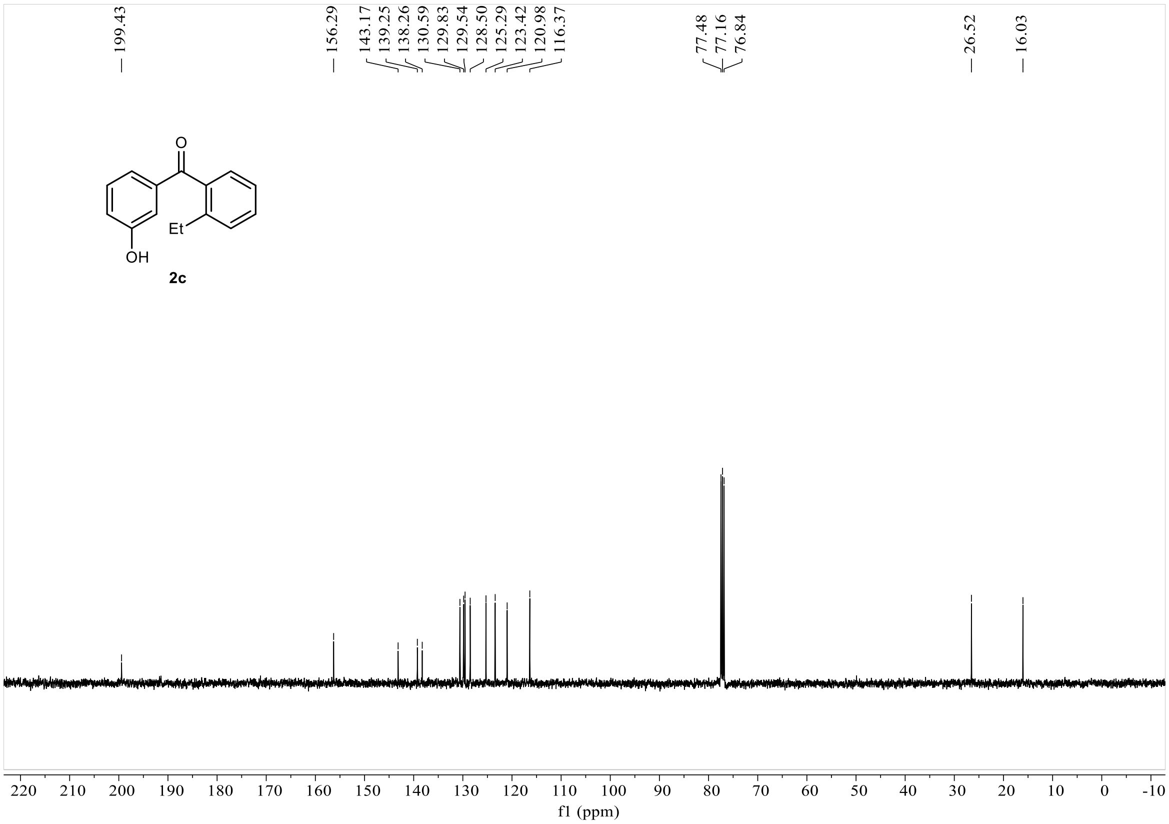

^{1}H NMR (400 MHz, $CDCl_3$) and ^{13}C NMR (101 MHz, $CDCl_3$) spectra of **2d**

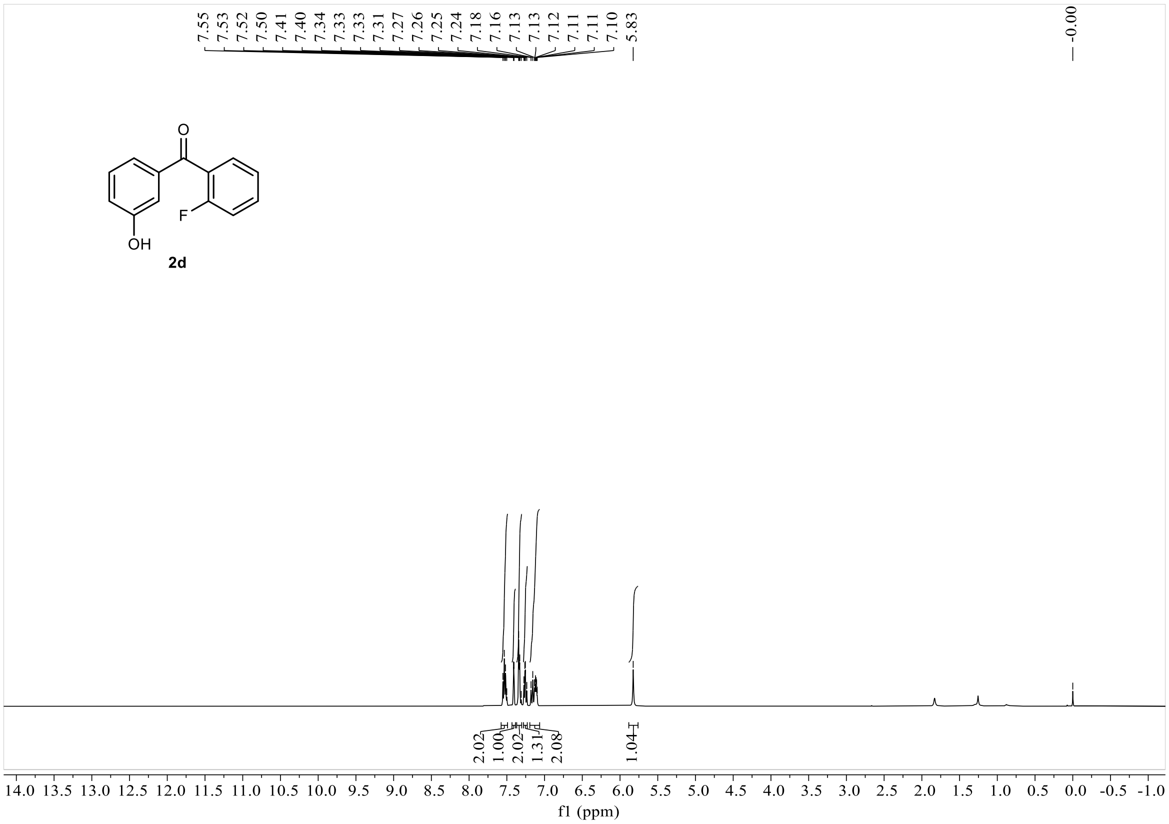

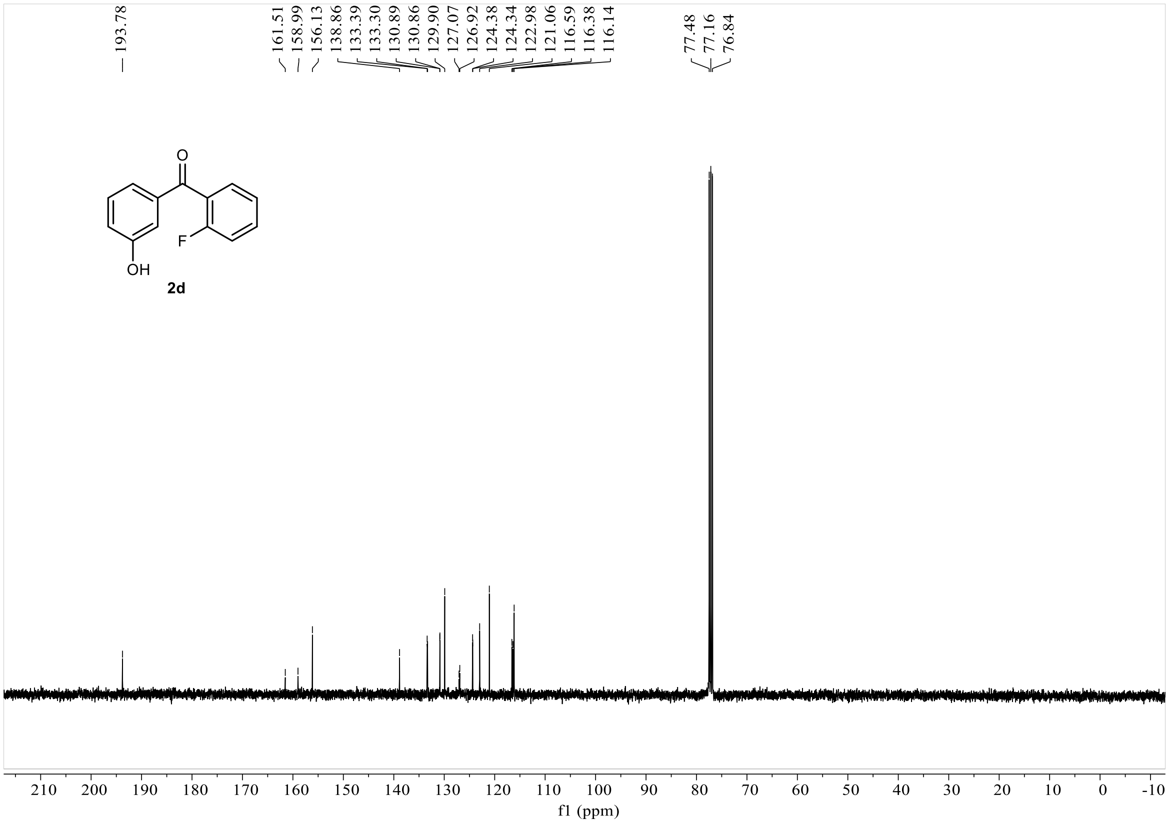

^{19}F NMR (376 MHz, $CDCl_3$) spectrum of **2d**

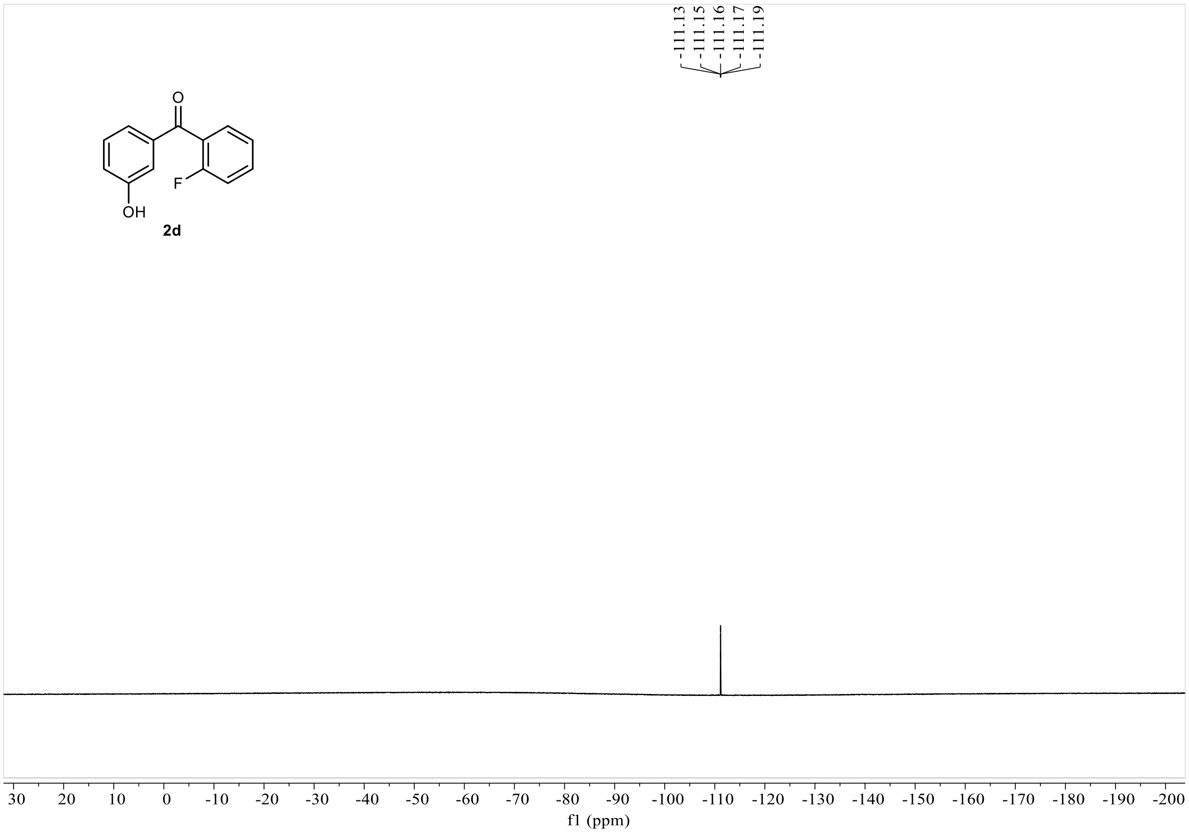

^{1}H NMR (400 MHz, $CDCl_3$) and ^{13}C NMR (101 MHz, $CDCl_3$) spectra of **2e**

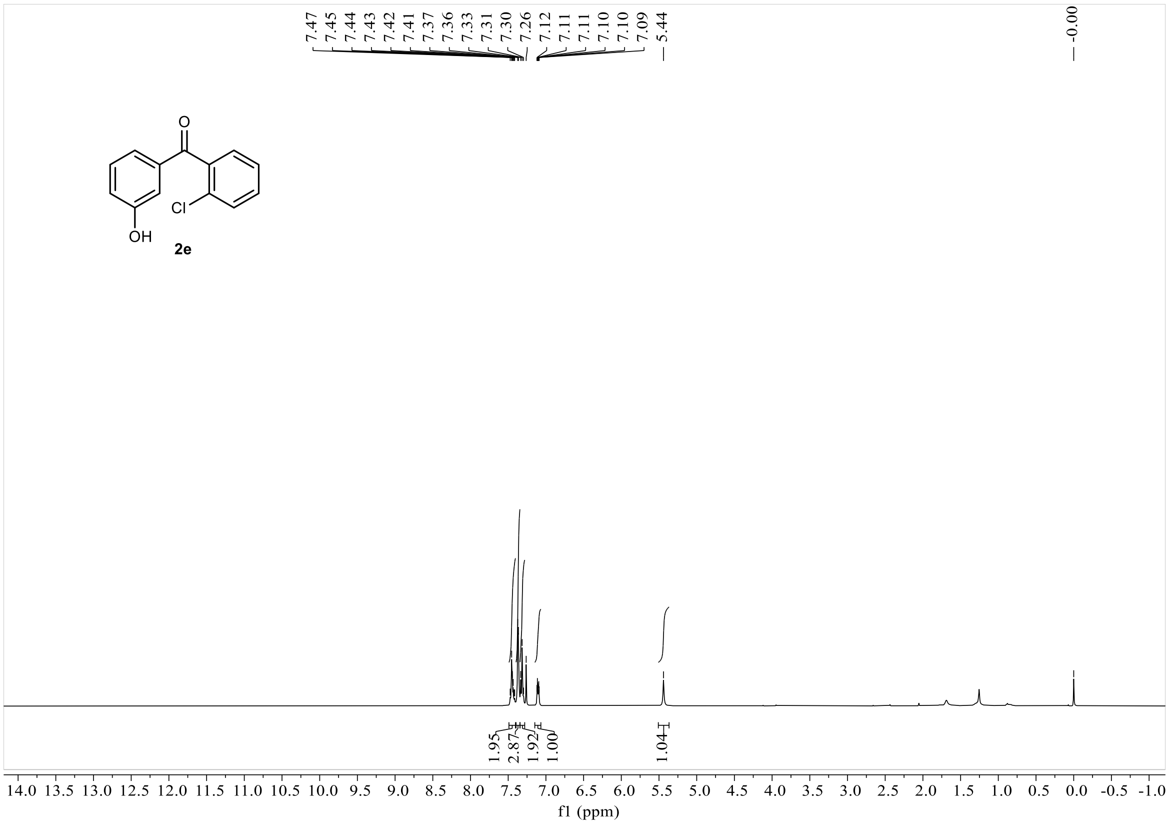

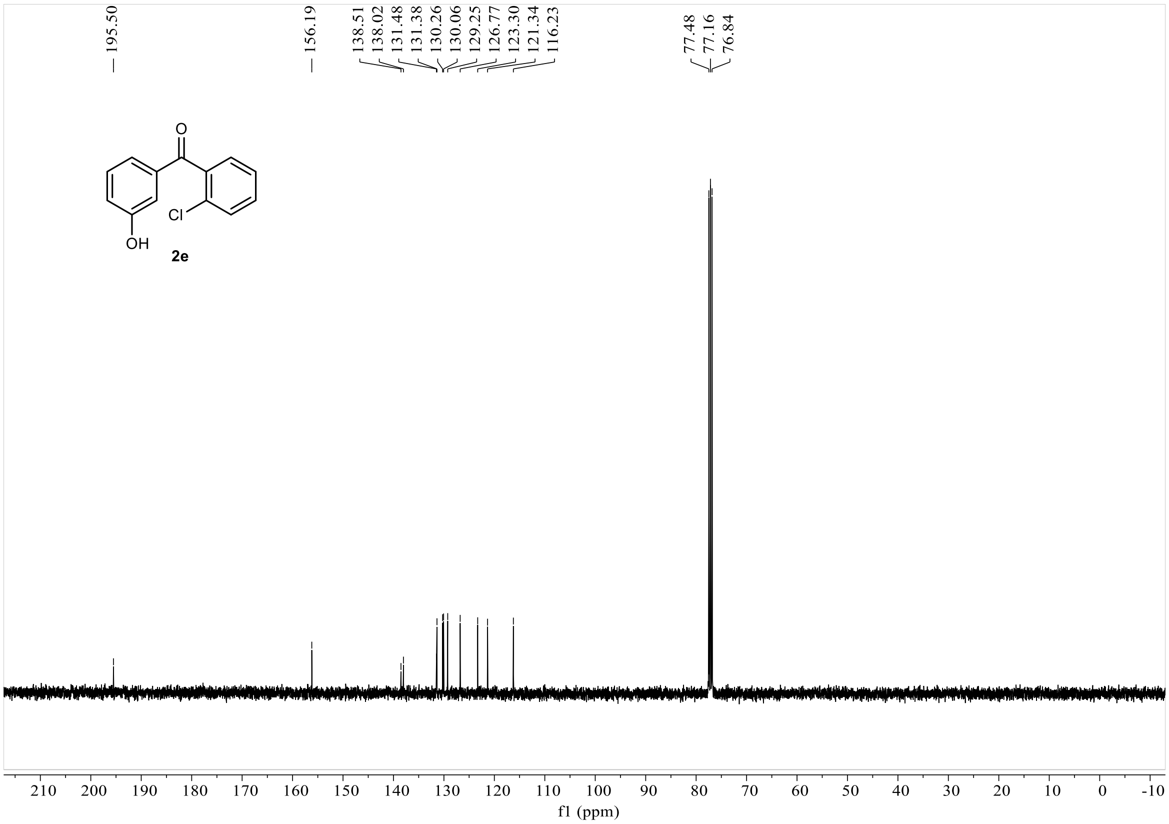

^{1}H NMR (400 MHz, $CDCl_3$) and ^{13}C NMR (101 MHz, $CDCl_3$) spectra of **2f**

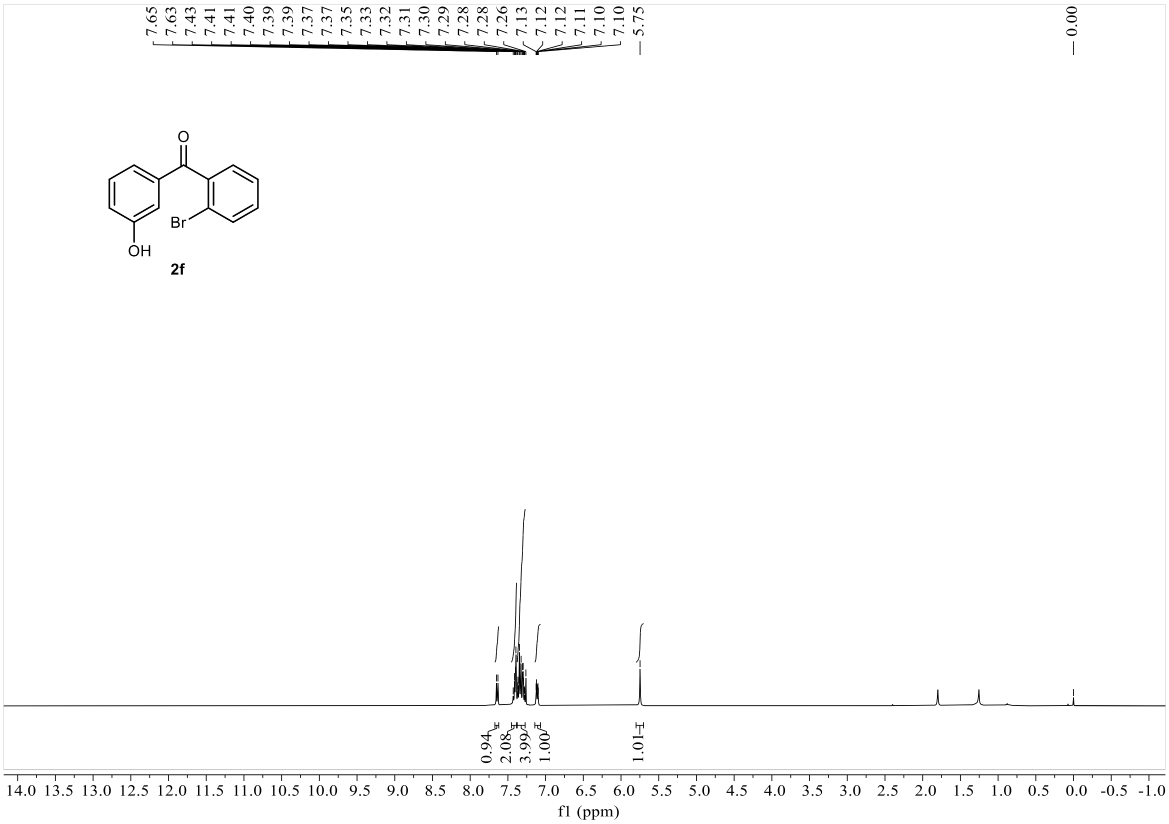

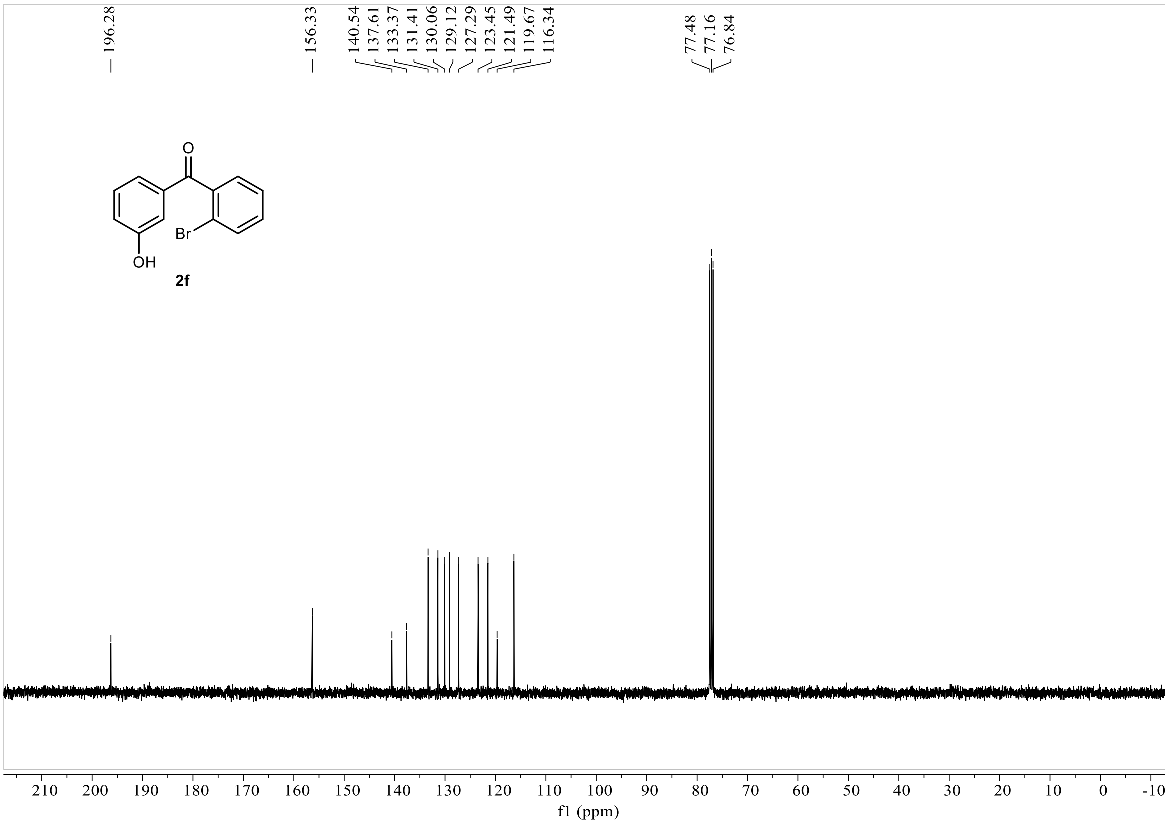

^{1}H NMR (400 MHz, Acetone-d_6) and ^{13}C NMR (101 MHz, Acetone-d_6) spectra of **2g**

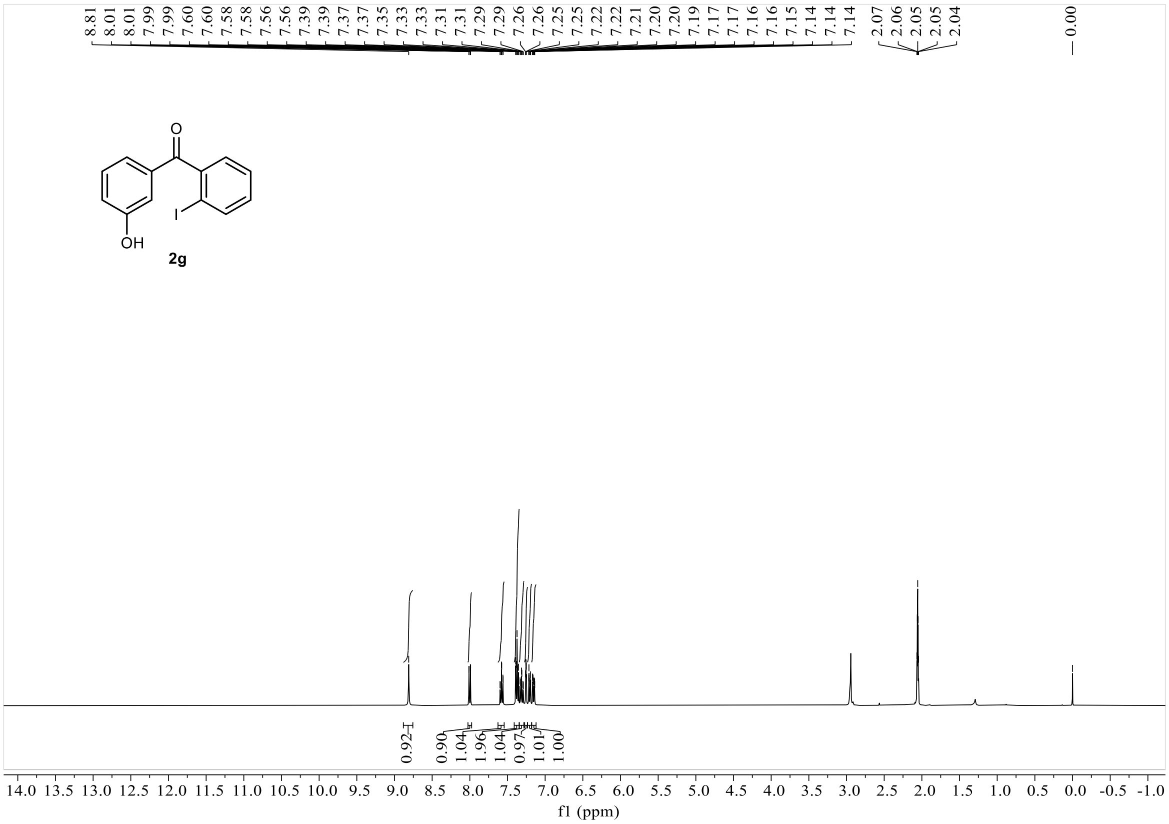

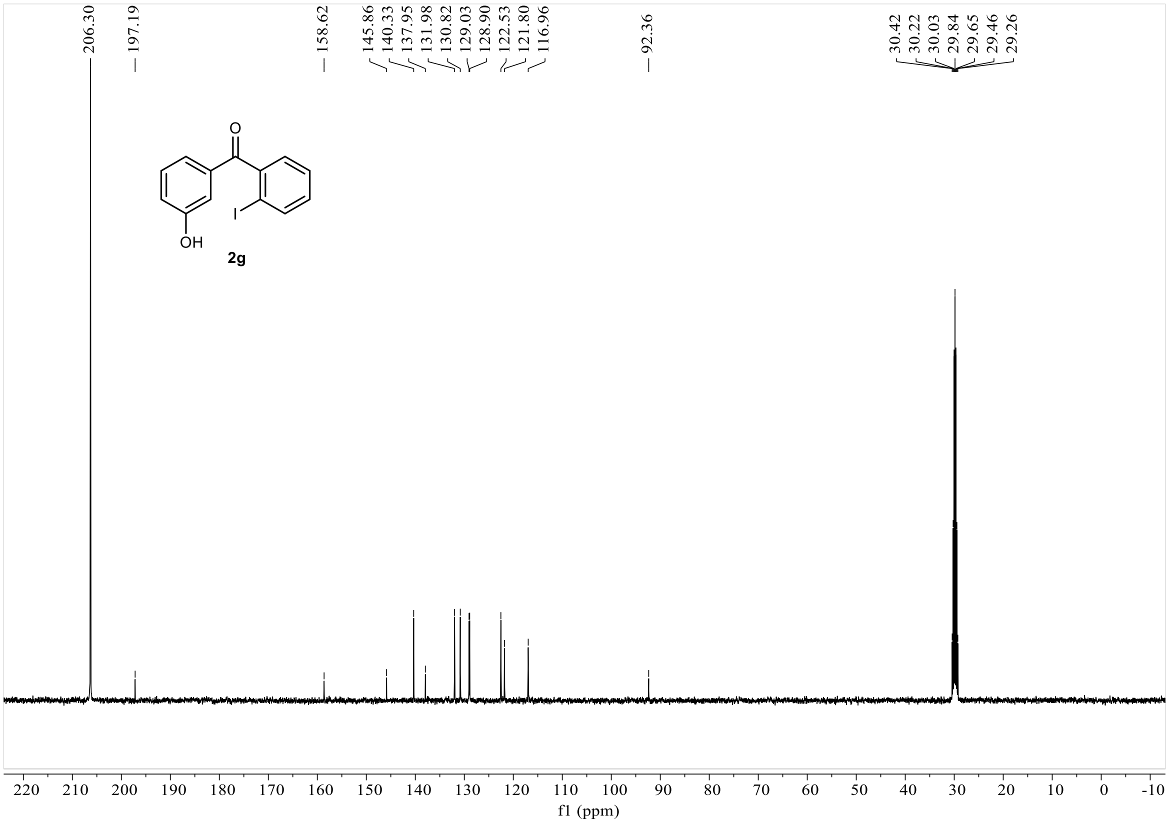

^{1}H NMR (400 MHz, $CDCl_3$) and ^{13}C NMR (101 MHz, $CDCl_3$) spectra of **2h**

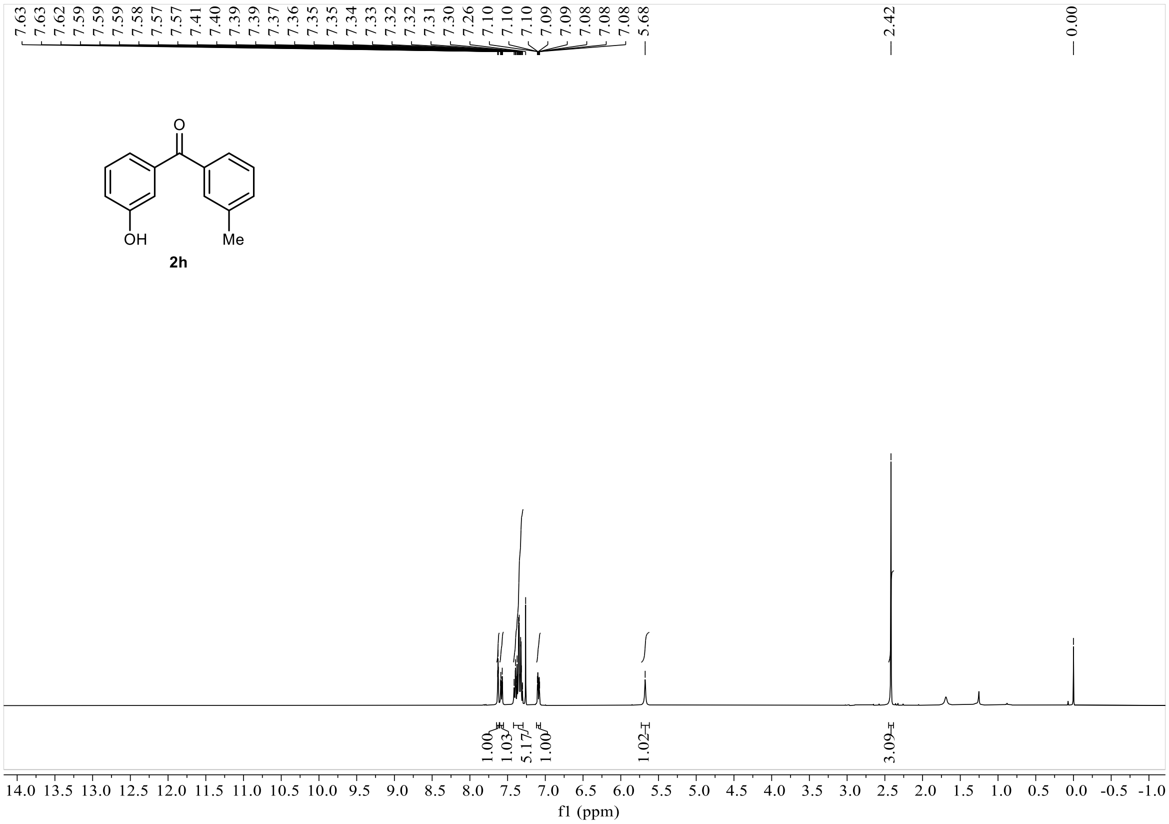

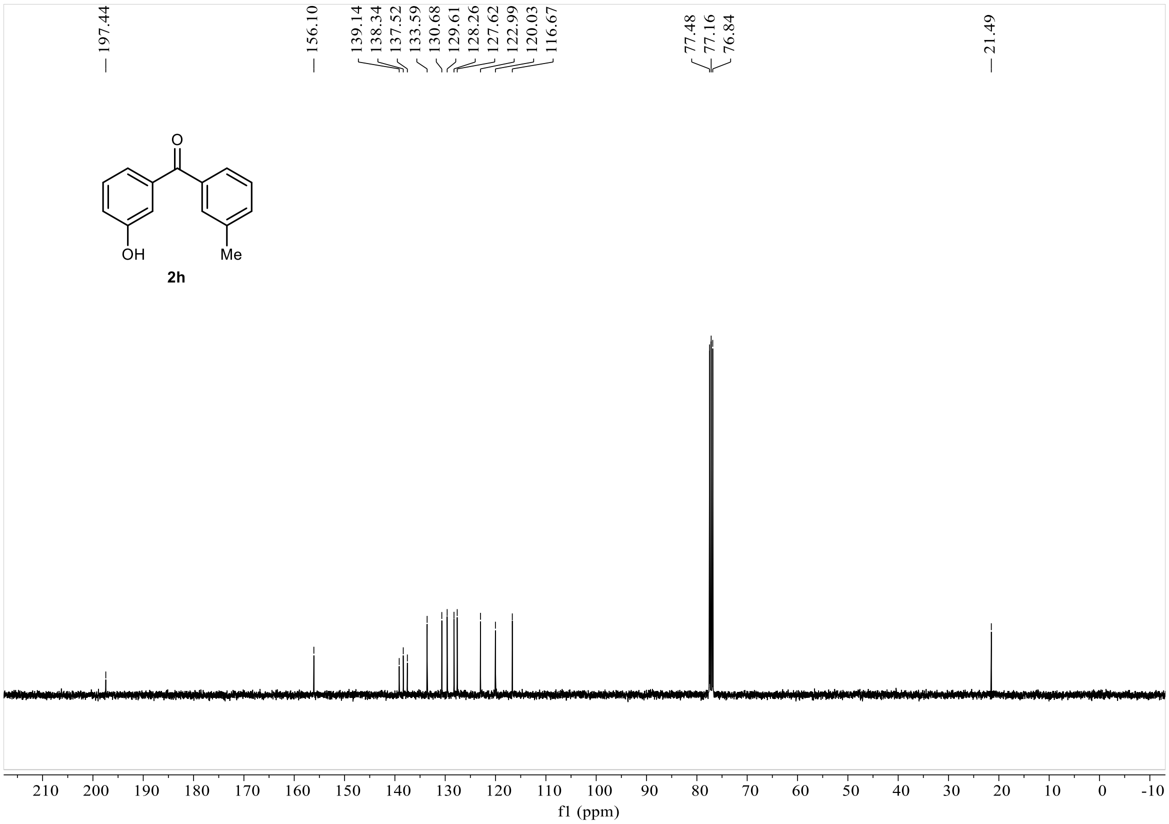

^{1}H NMR (400 MHz, $CDCl_3$) and ^{13}C NMR (101 MHz, $CDCl_3$) spectra of **2i**

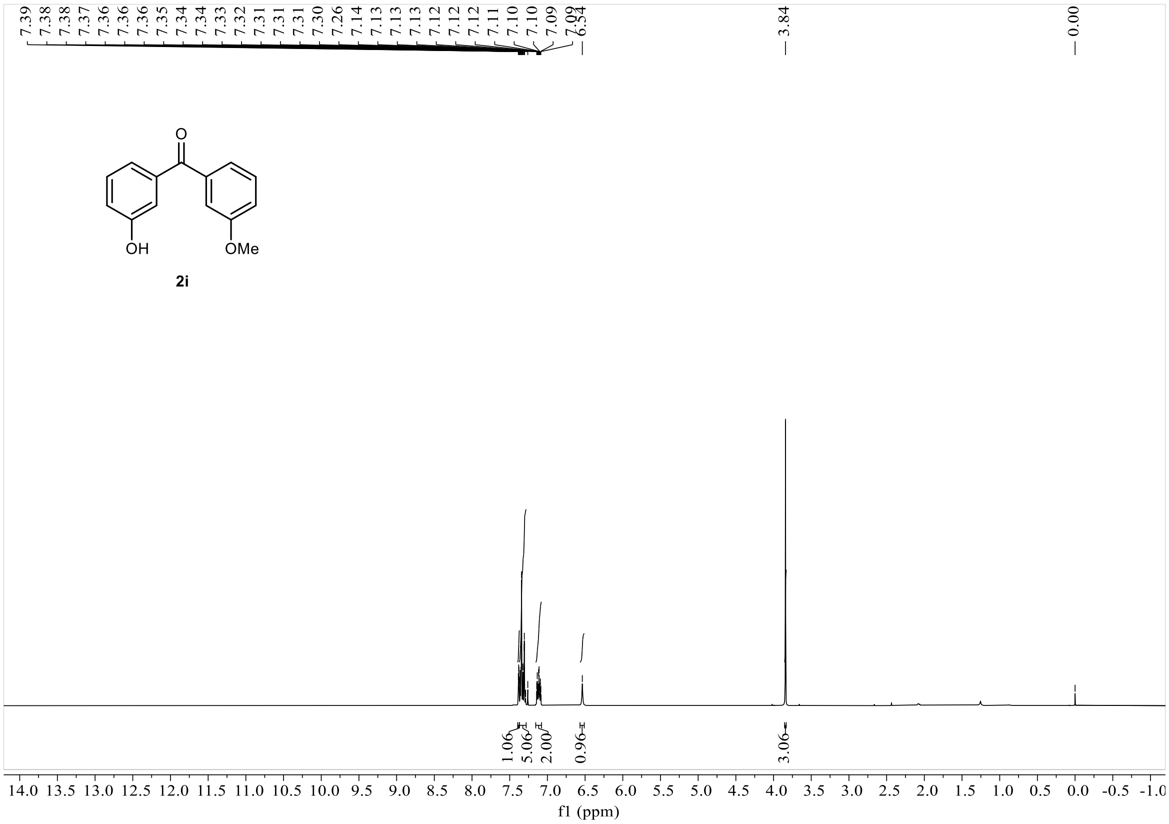

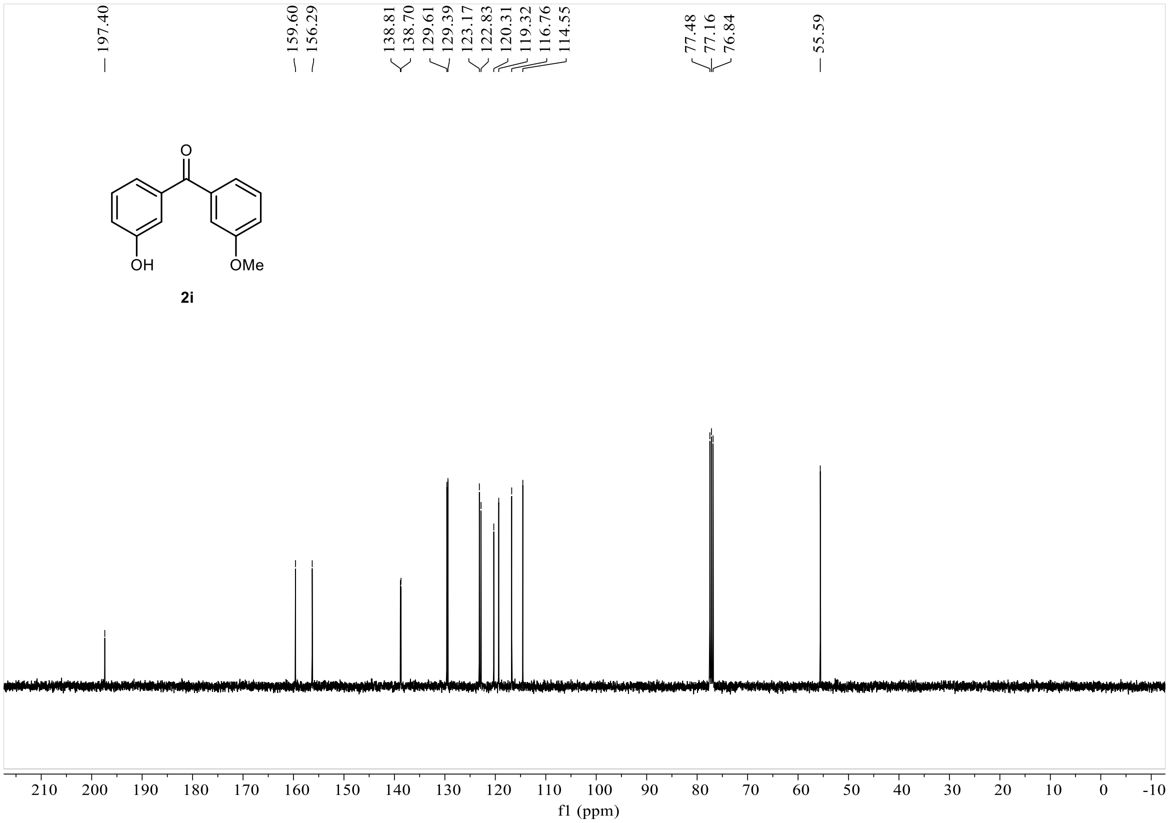

^{1}H NMR (400 MHz, Acetone-d_6) and ^{13}C NMR (101 MHz, Acetone-d_6) spectra of **2j**

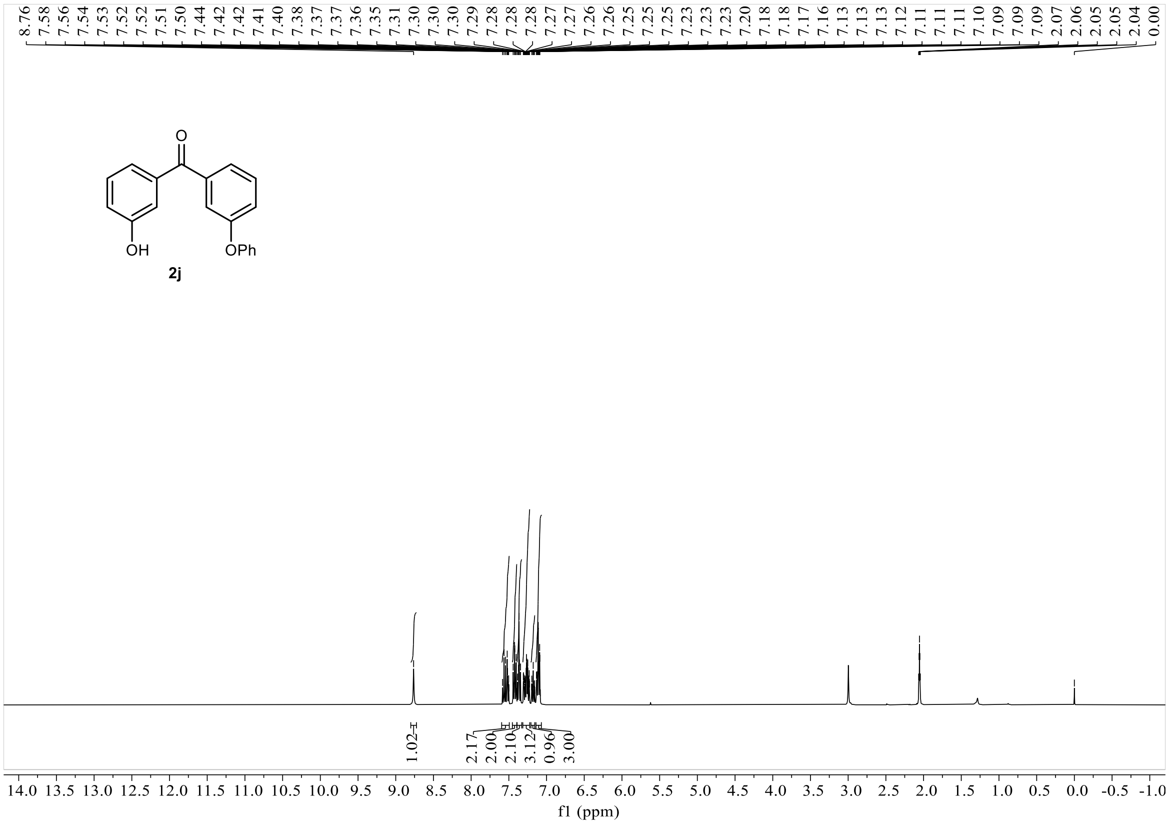

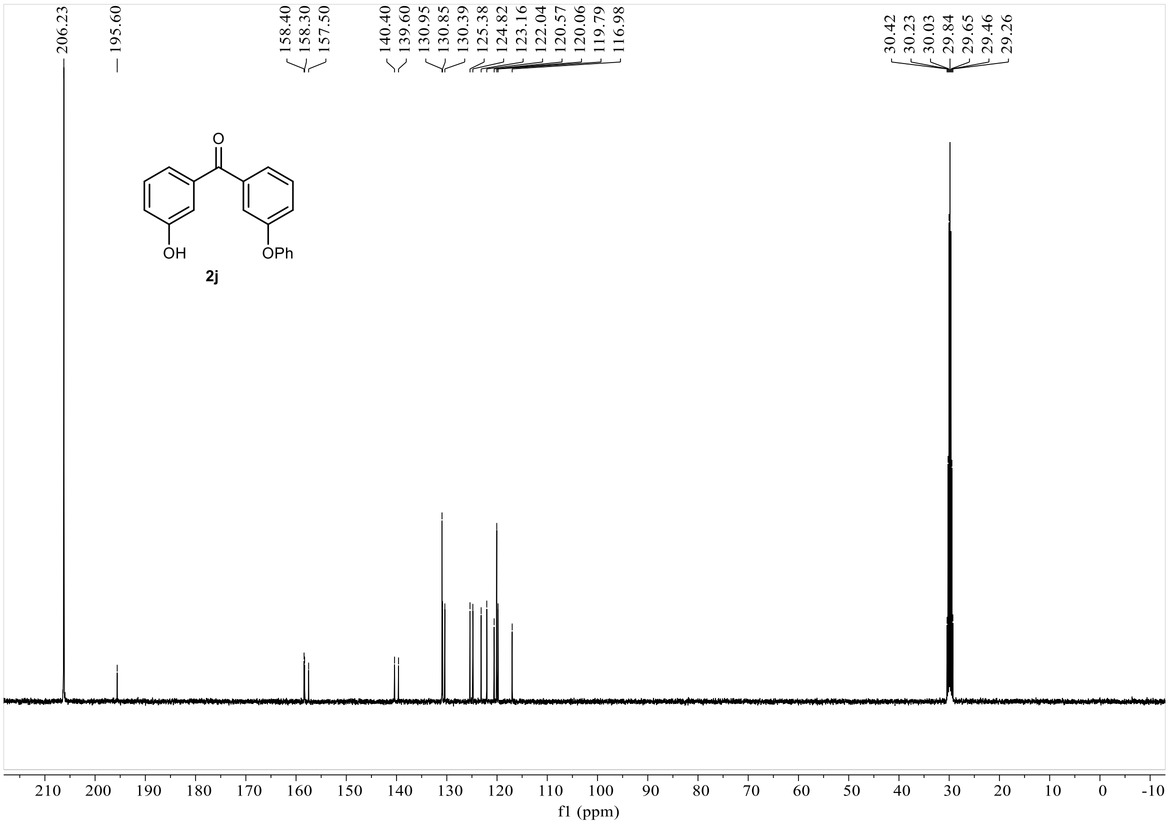

^{1}H NMR (400 MHz, $CDCl_3$) and ^{13}C NMR (101 MHz, $CDCl_3$) spectra of **2k**

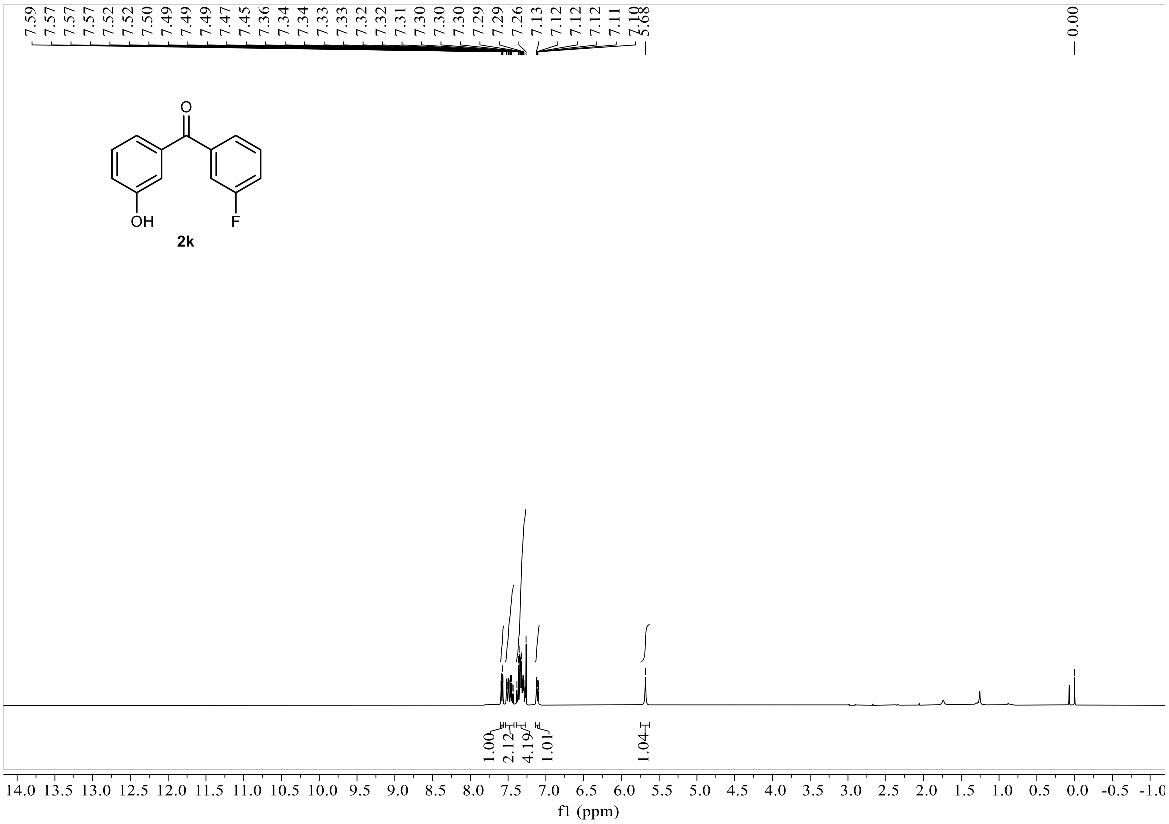

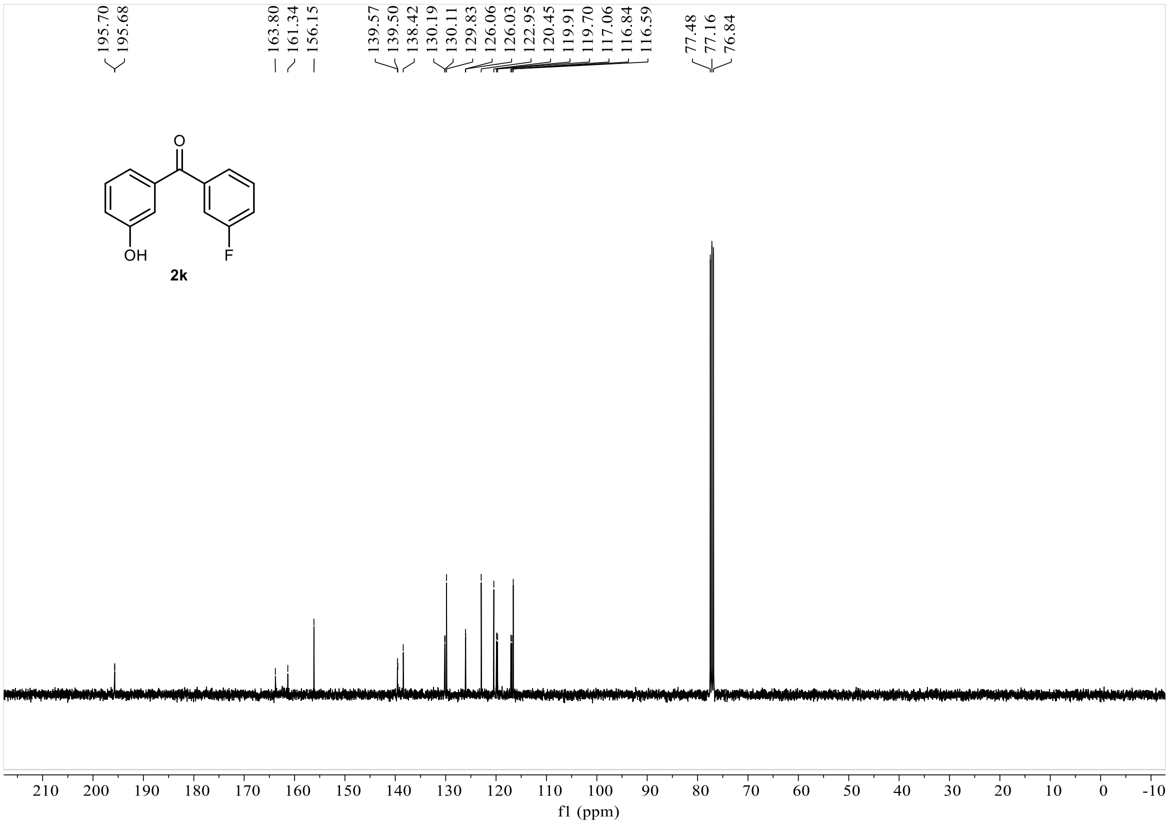

^{19}F NMR (376 MHz, $CDCl_3$) spectrum of **2k**

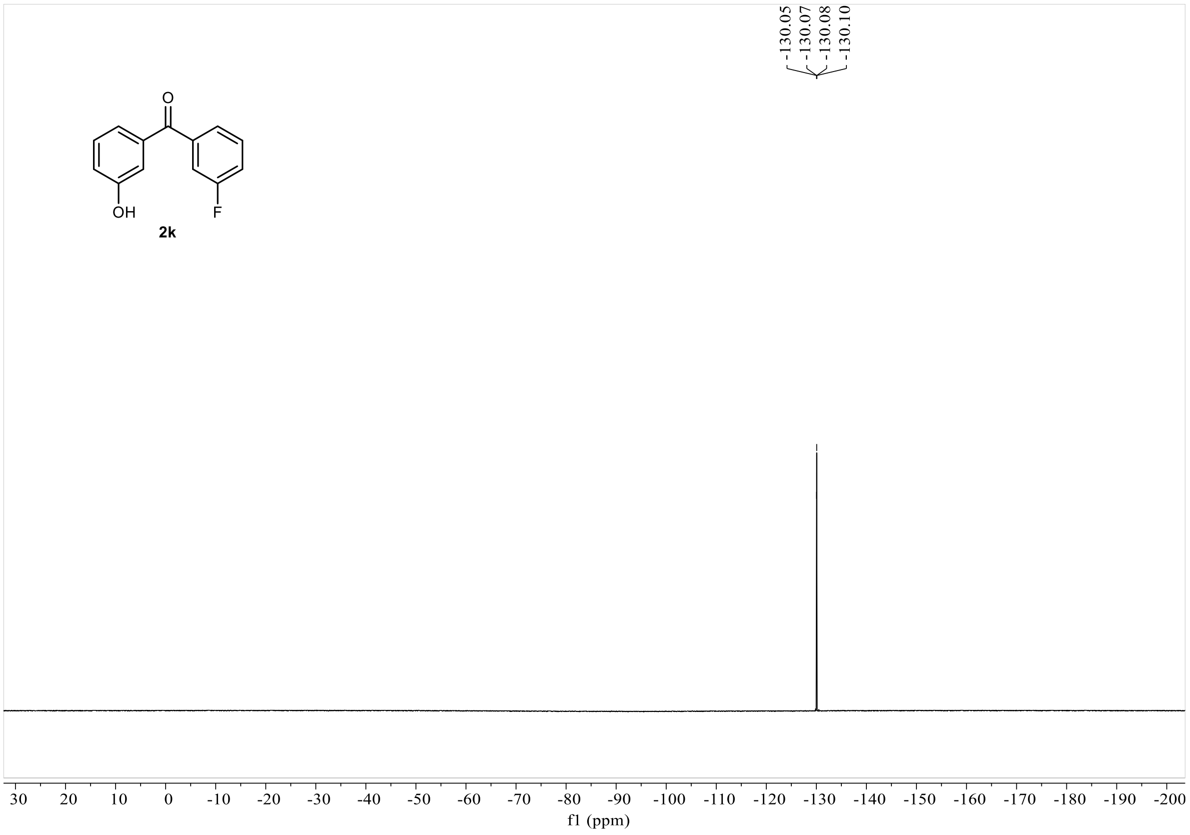

^{1}H NMR (400 MHz, Acetone-d_6) and ^{13}C NMR (101 MHz, Acetone-d_6) spectra of **2l**

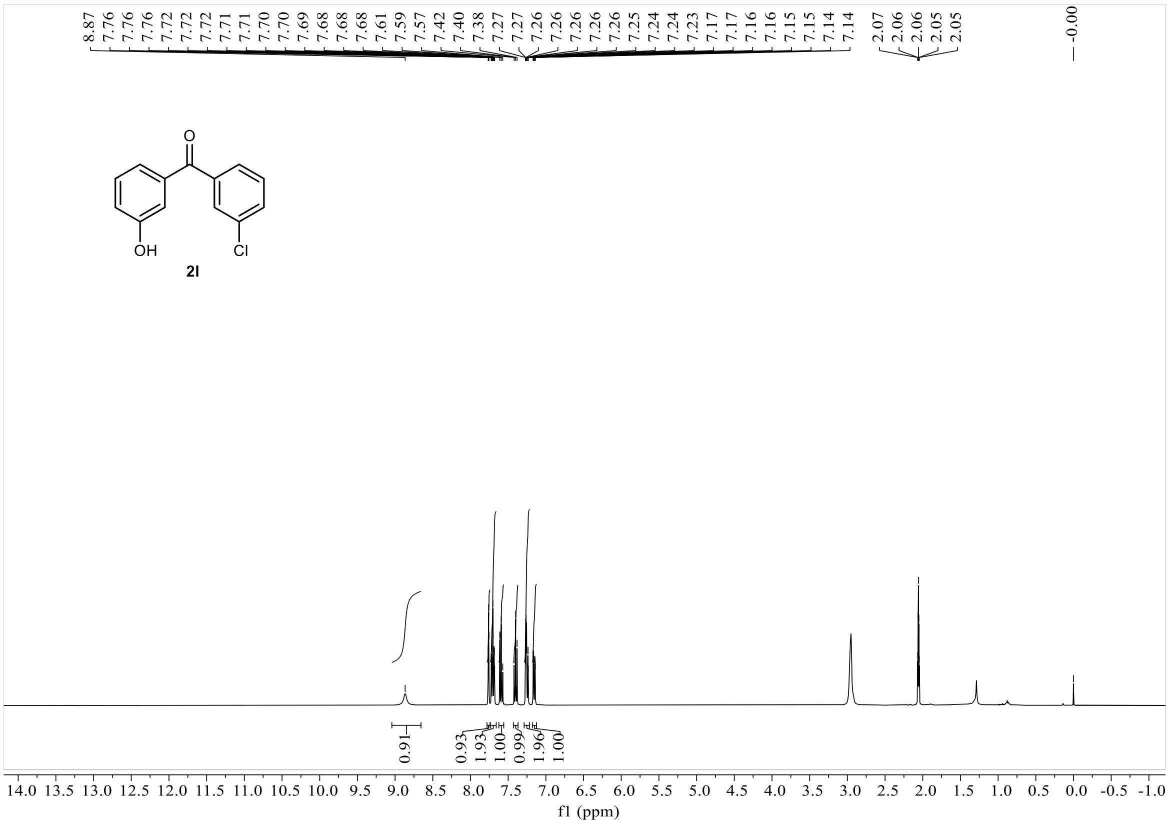

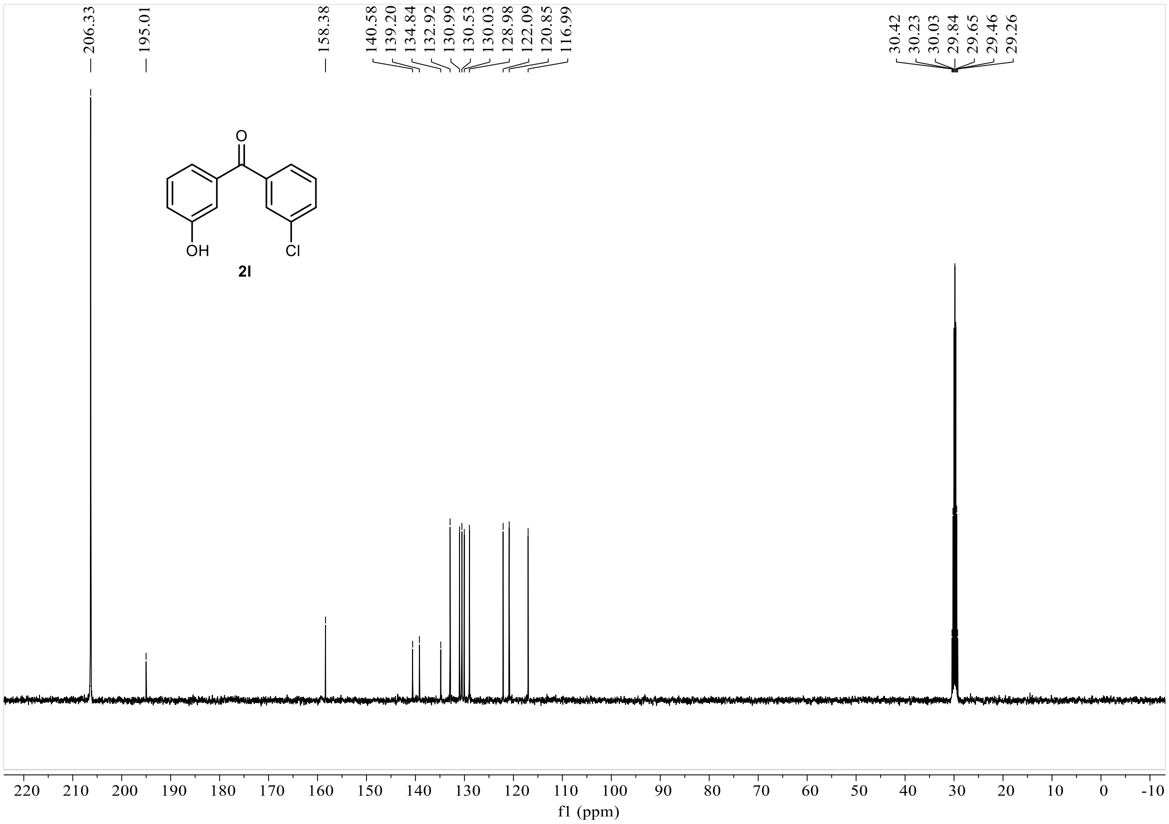

^{1}H NMR (400 MHz, Acetone-d_6) and ^{13}C NMR (101 MHz, Acetone-d_6) spectra of **2m**

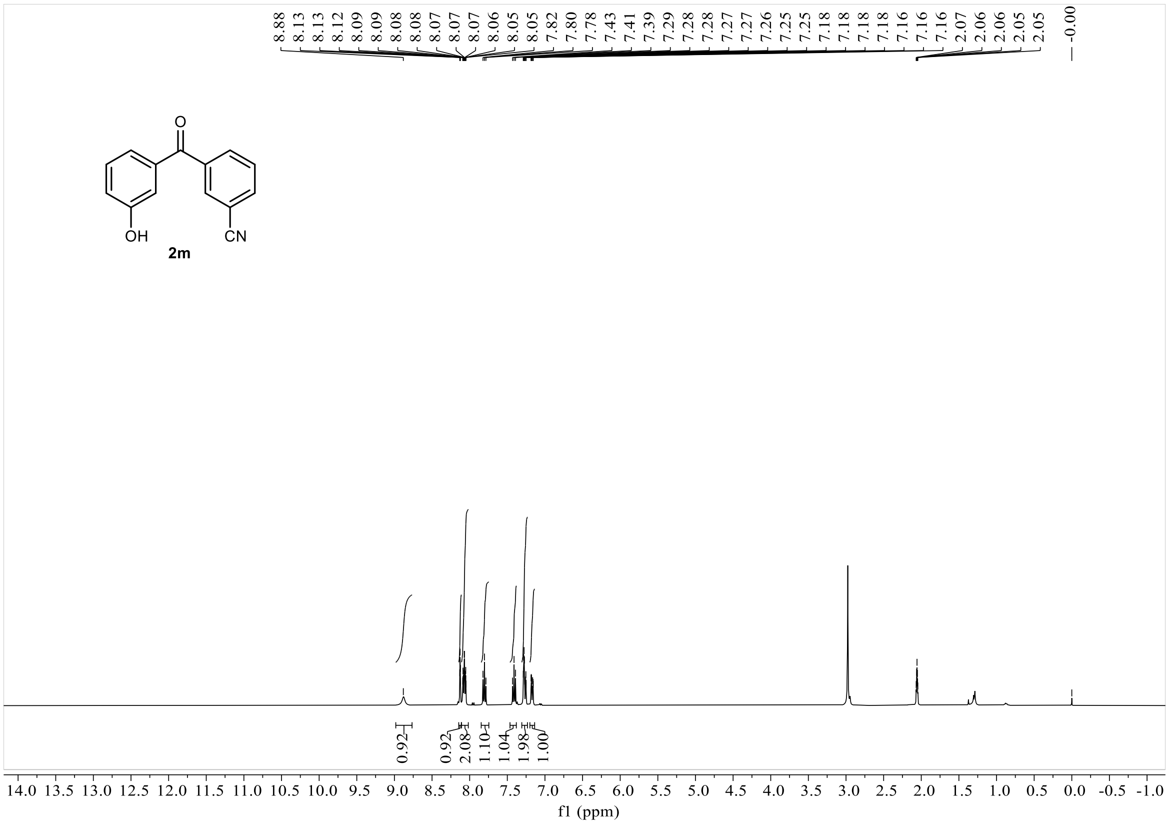

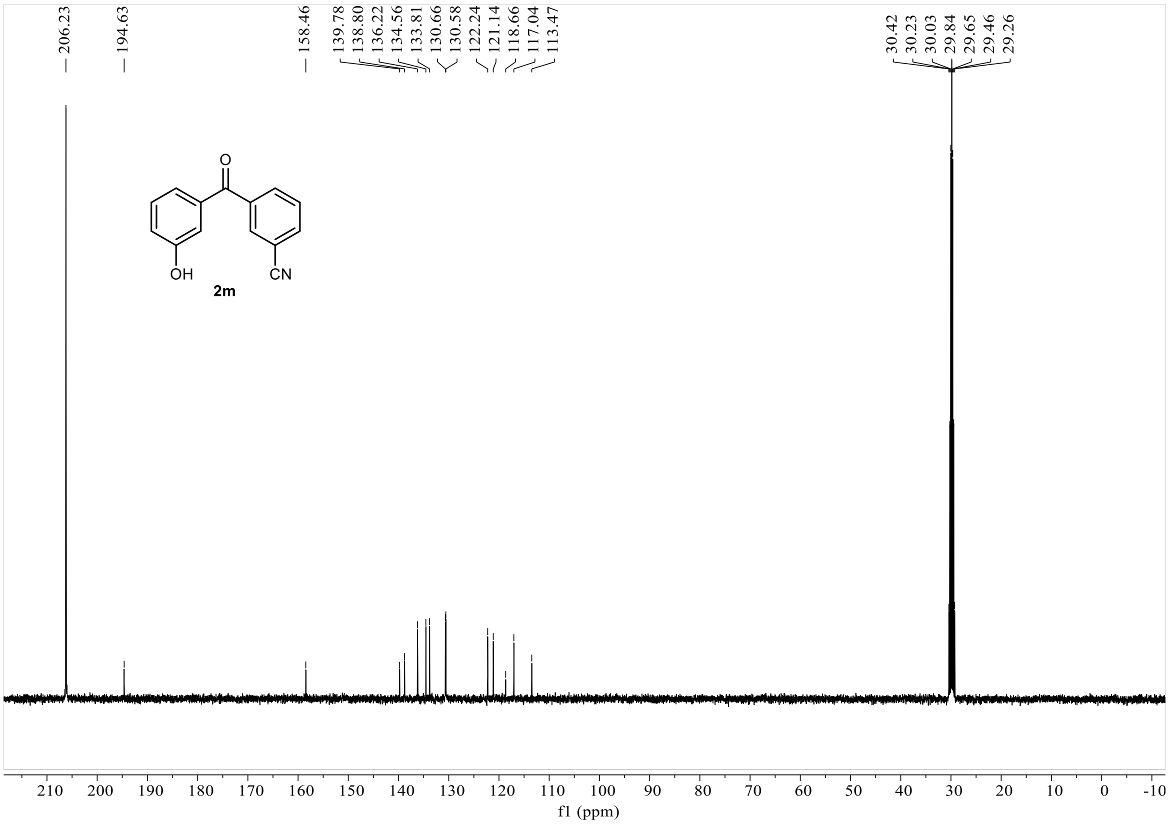

^1H NMR (400 MHz, $CDCl_3$) and ^{13}C NMR (101 MHz, $CDCl_3$) spectra of **2n**

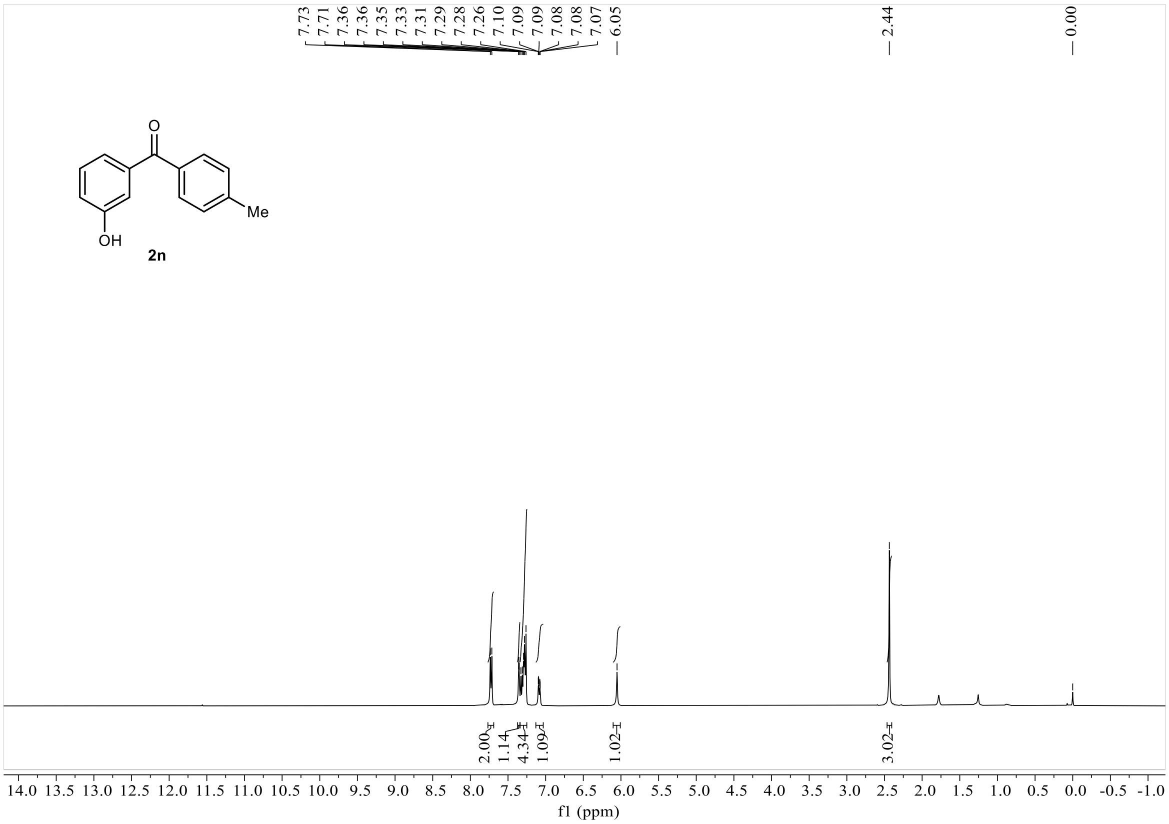

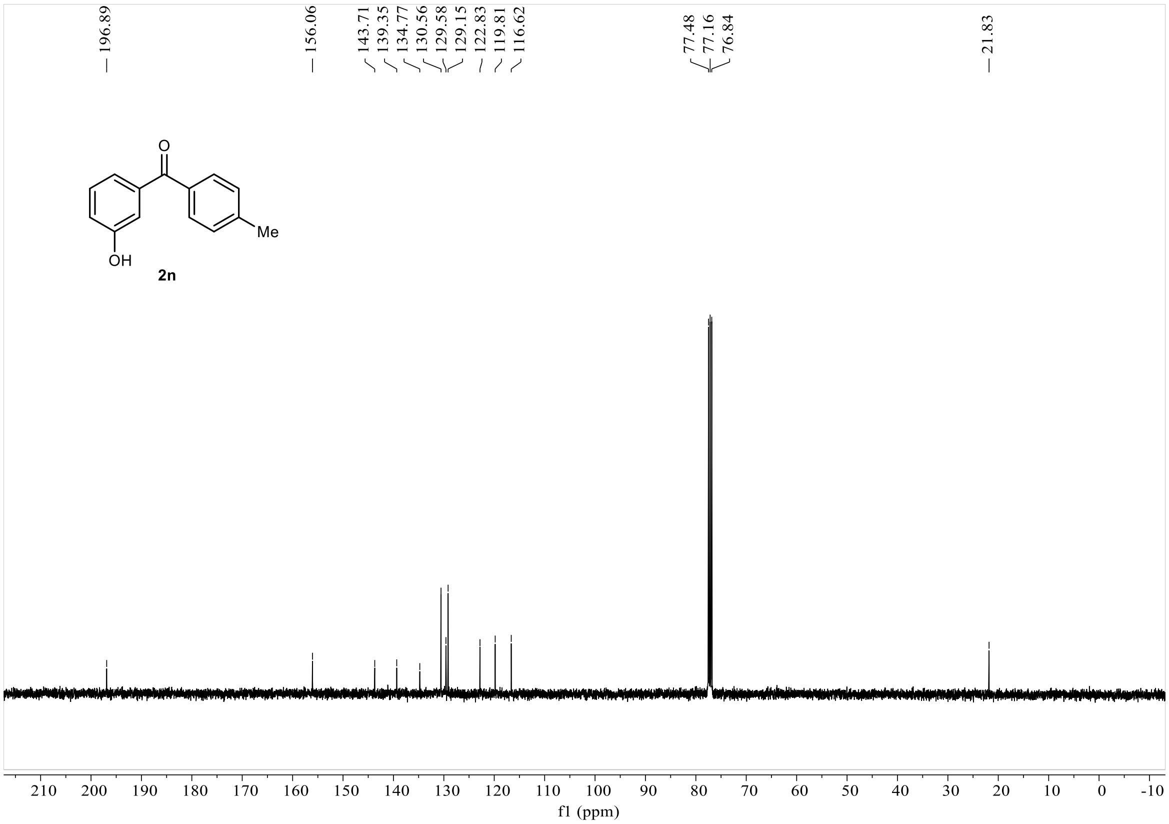

^{1}H NMR (400 MHz, $CDCl_3$) and ^{13}C NMR (101 MHz, $CDCl_3$) spectra of **2o**

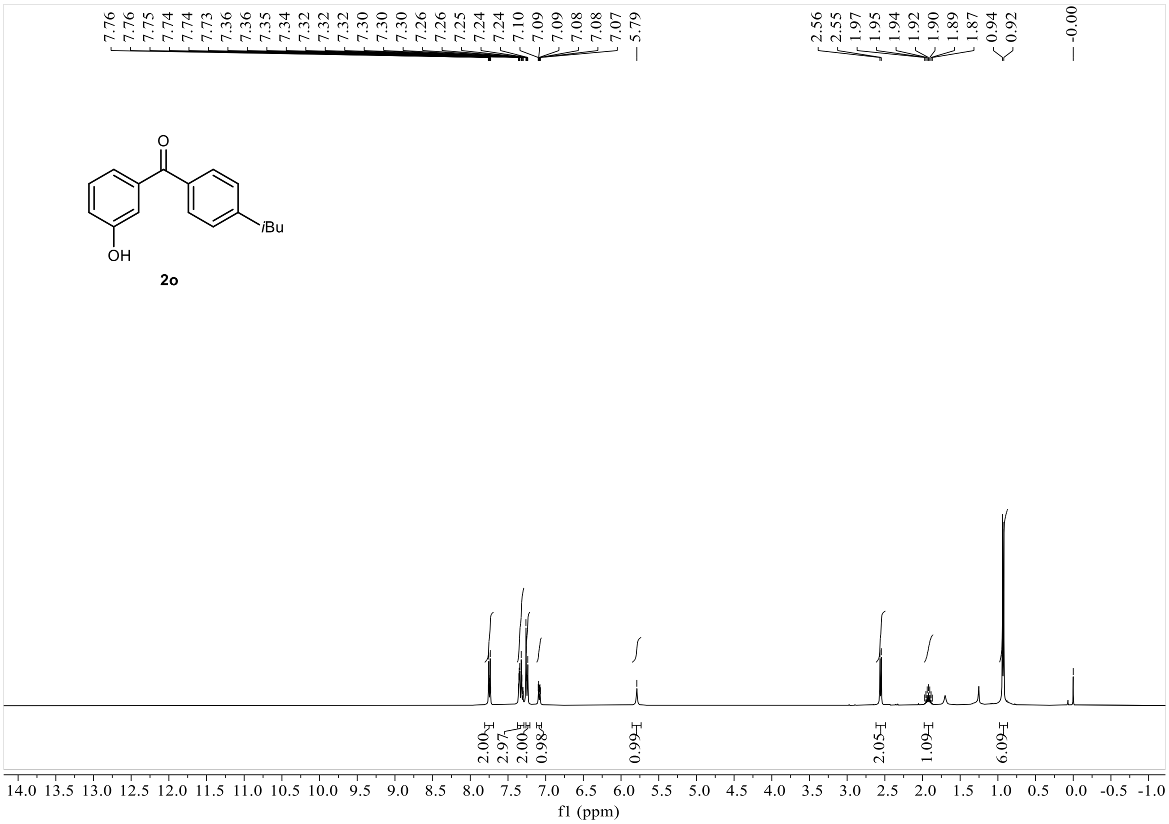

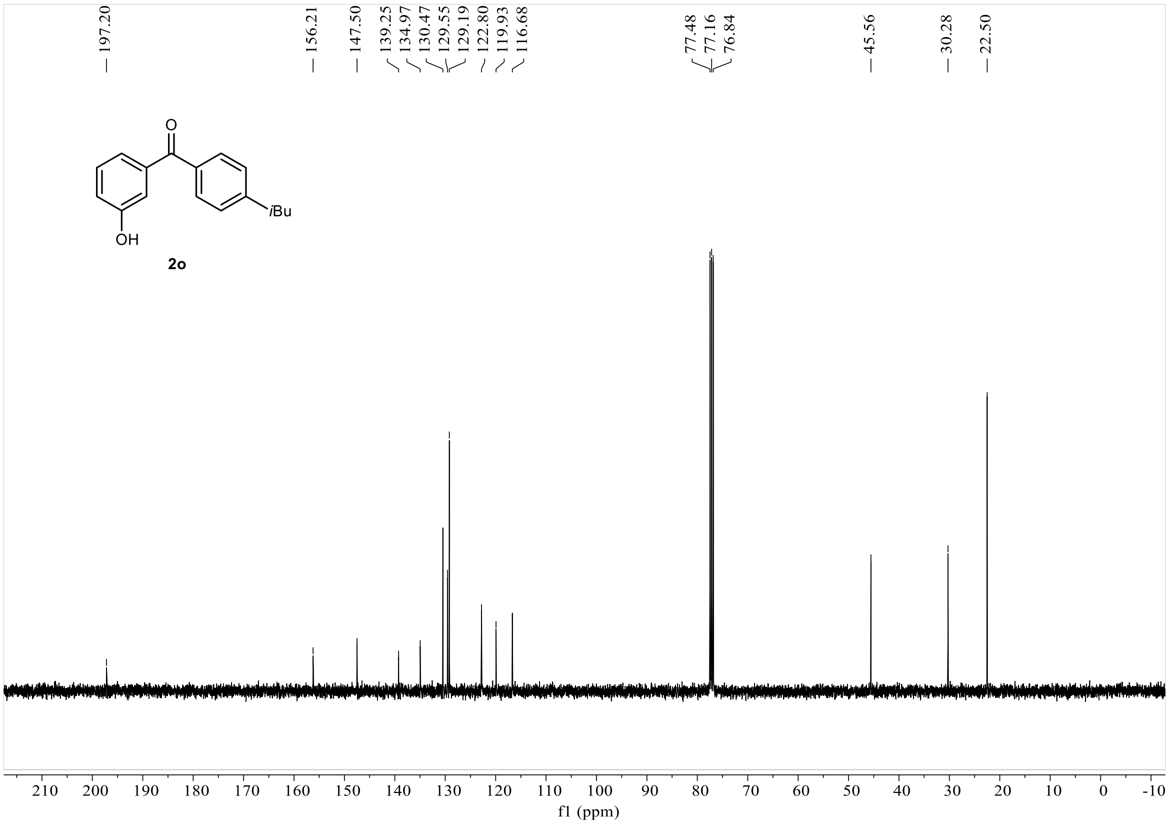

^{1}H NMR (400 MHz, $CDCl_3$) and ^{13}C NMR (101 MHz, $CDCl_3$) spectra of **2p**

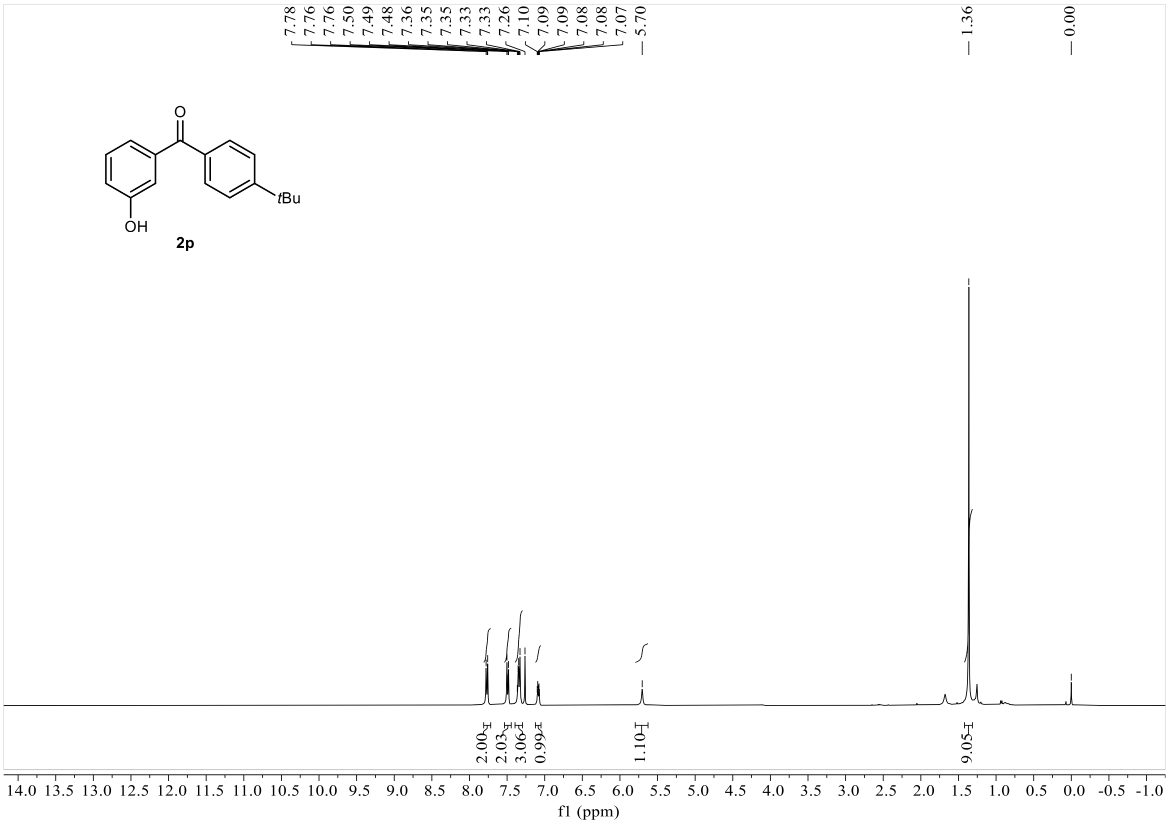

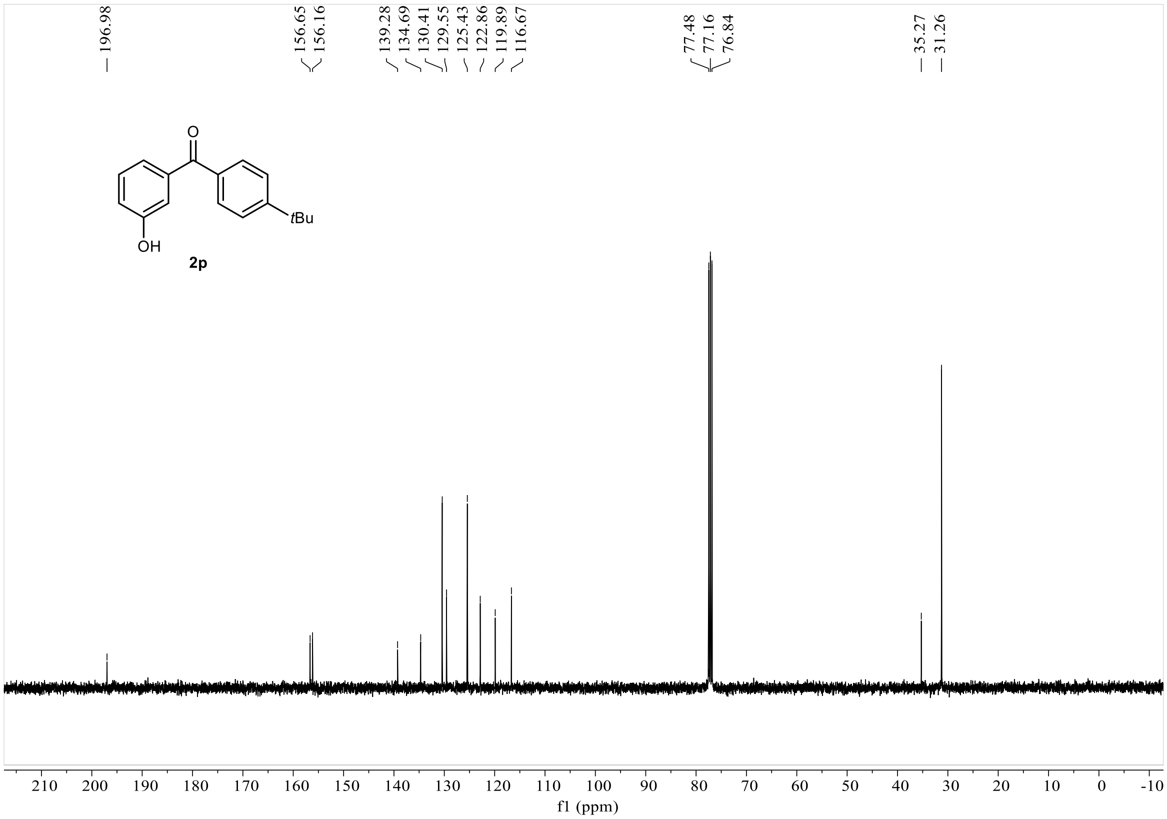

^{1}H NMR (400 MHz, Acetone-d_6) and ^{13}C NMR (101 MHz, Acetone-d_6) spectra of **2q**

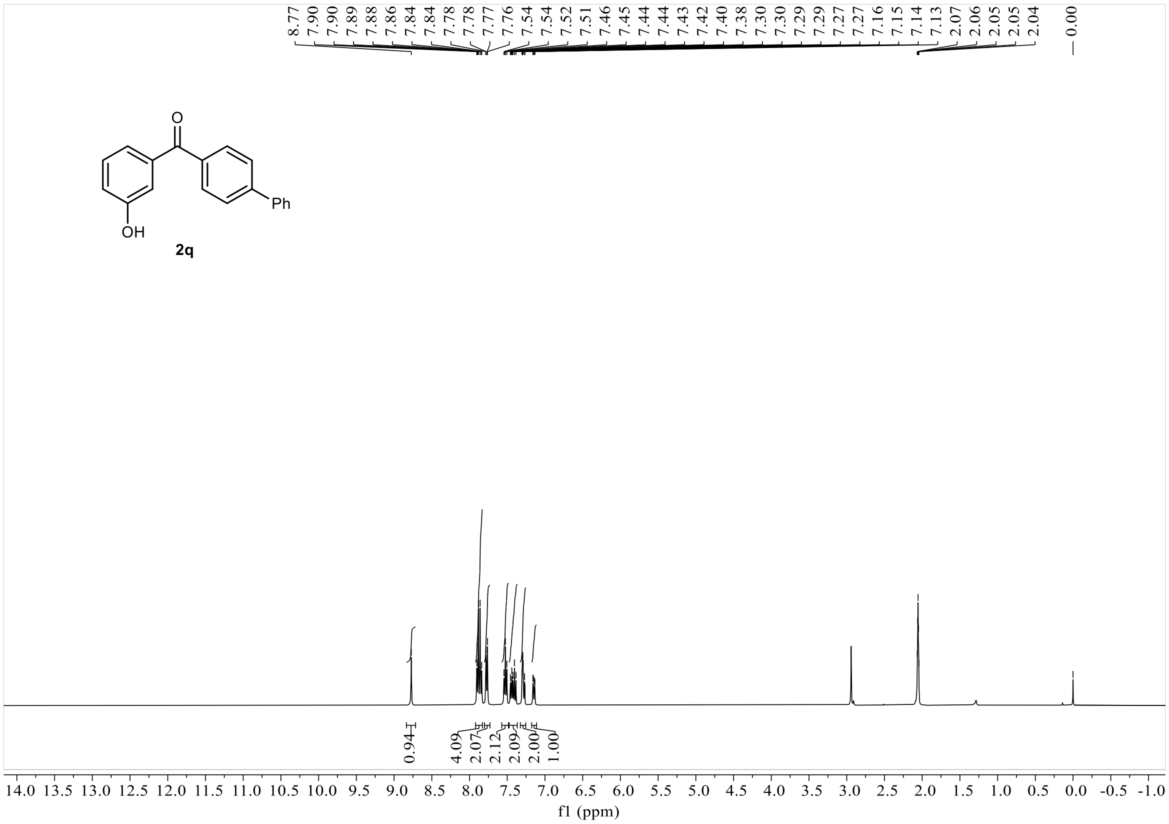

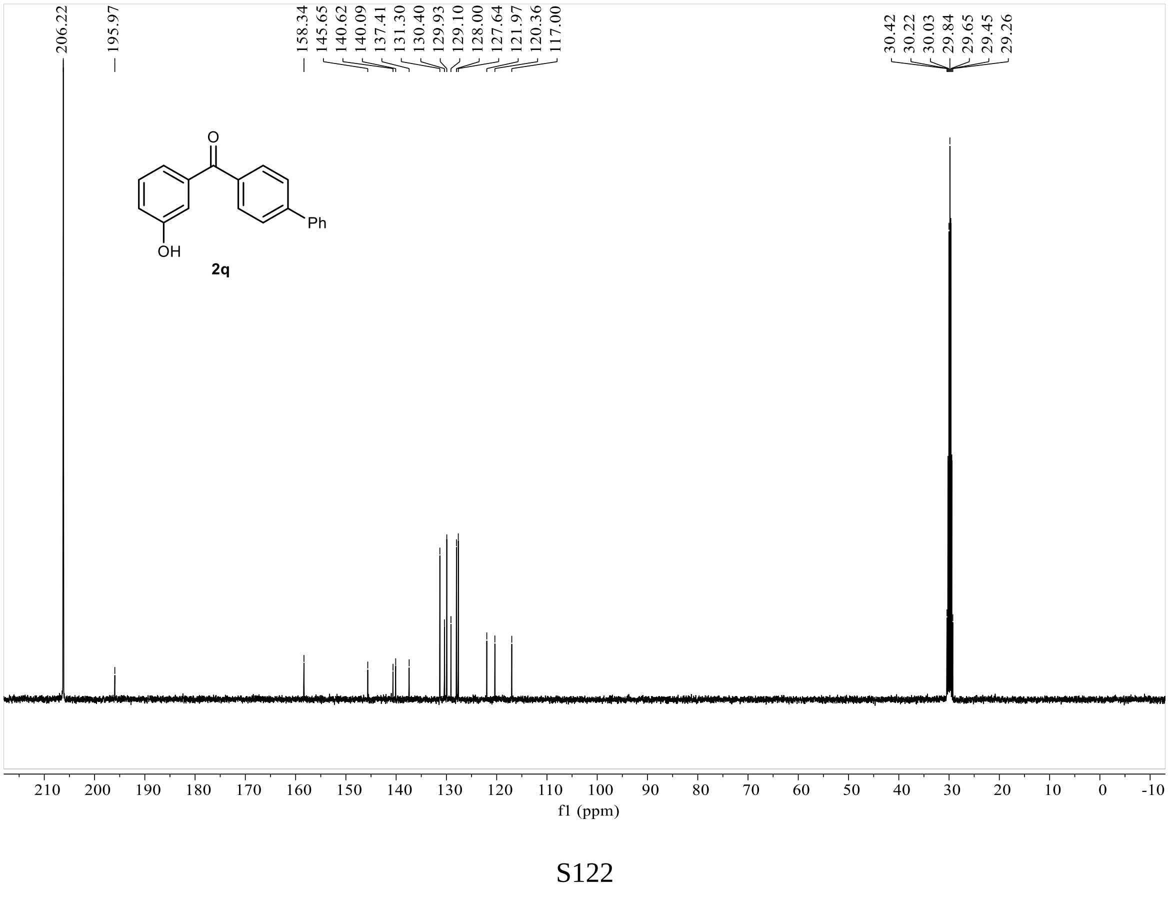

^{1}H NMR (400 MHz, $CDCl_3$) and ^{13}C NMR (101 MHz, $CDCl_3$) spectra of **2r**

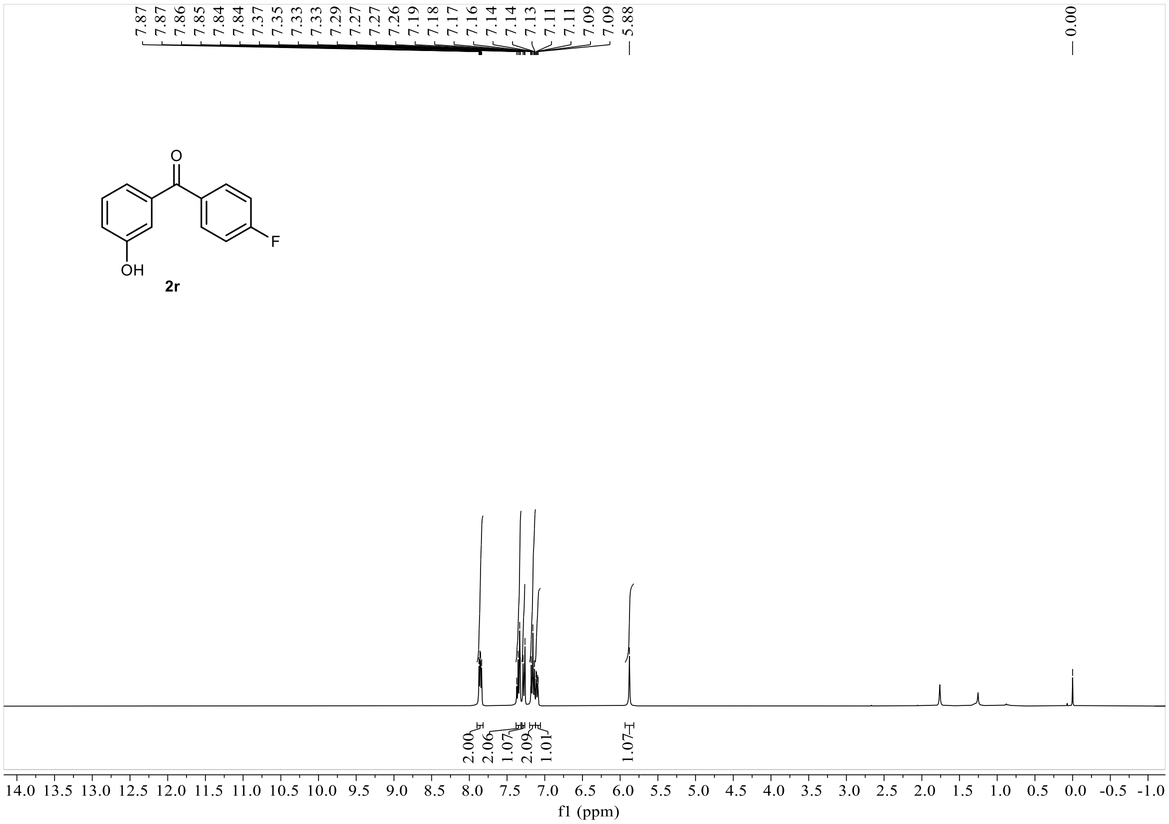

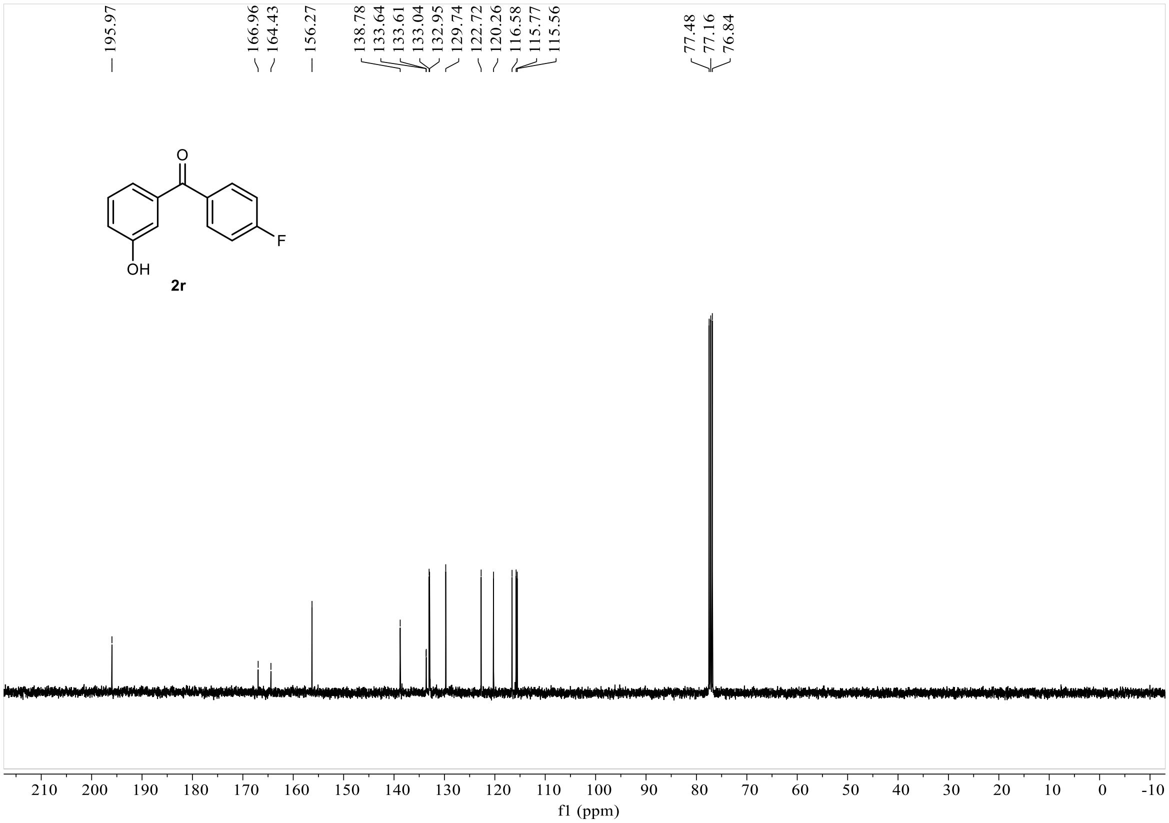

^{19}F NMR (376 MHz, $CDCl_3$) spectrum of **2r**

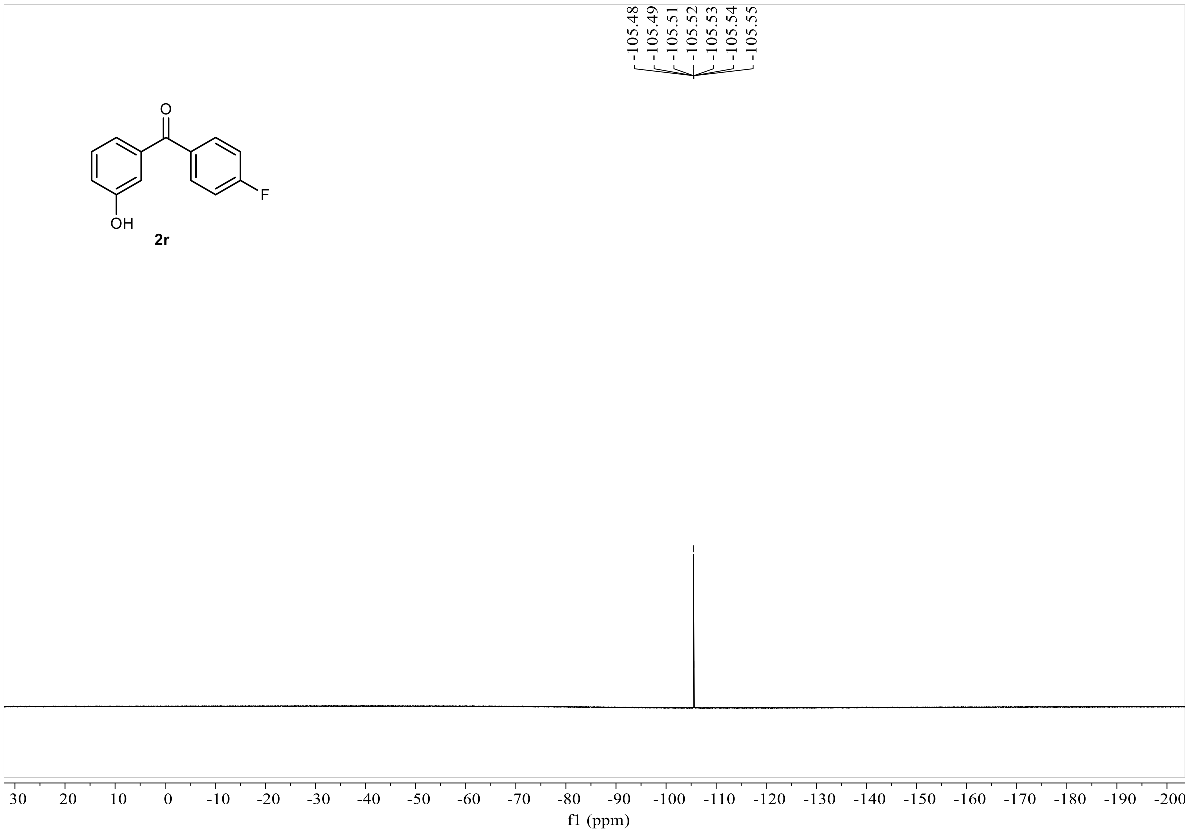

^{1}H NMR (400 MHz, Acetone-d_6) and ^{13}C NMR (101 MHz, Acetone-d_6) spectra of **2s**

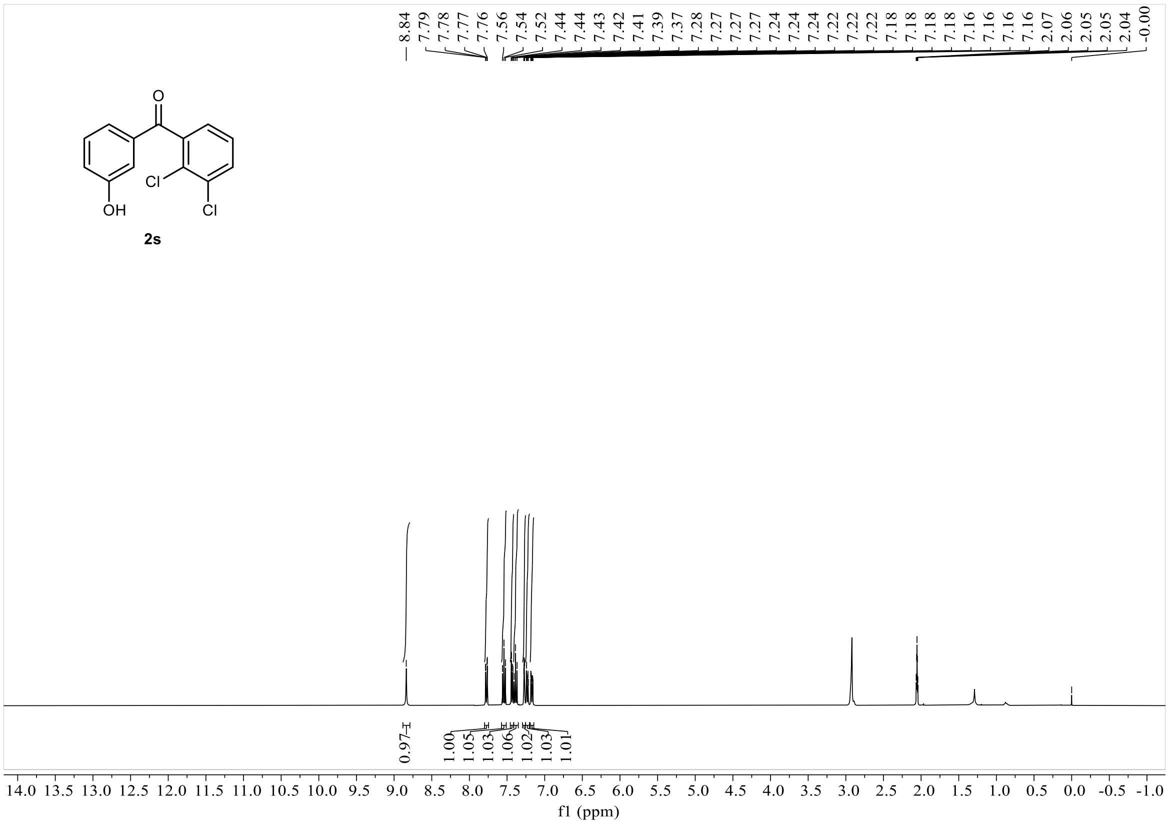

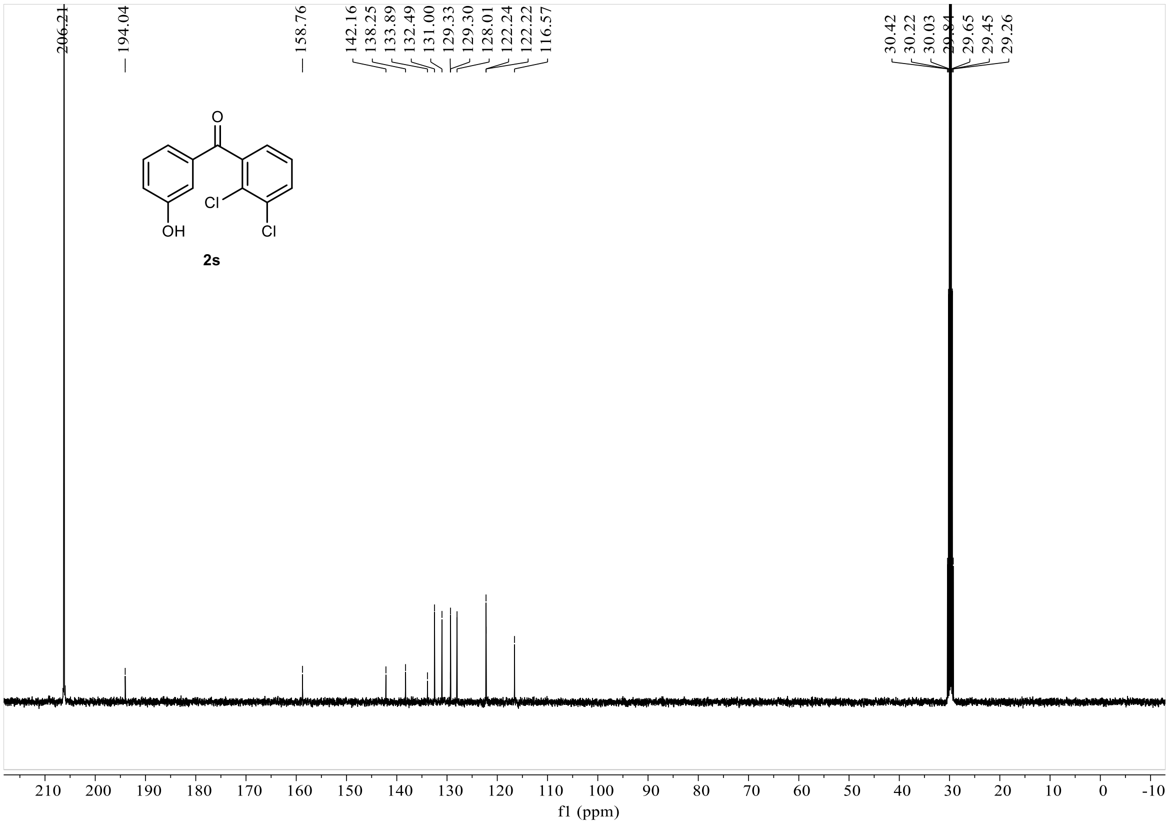

^{1}H NMR (400 MHz, Acetone-d_6) and ^{13}C NMR (101 MHz, Acetone-d_6) spectra of **2t**

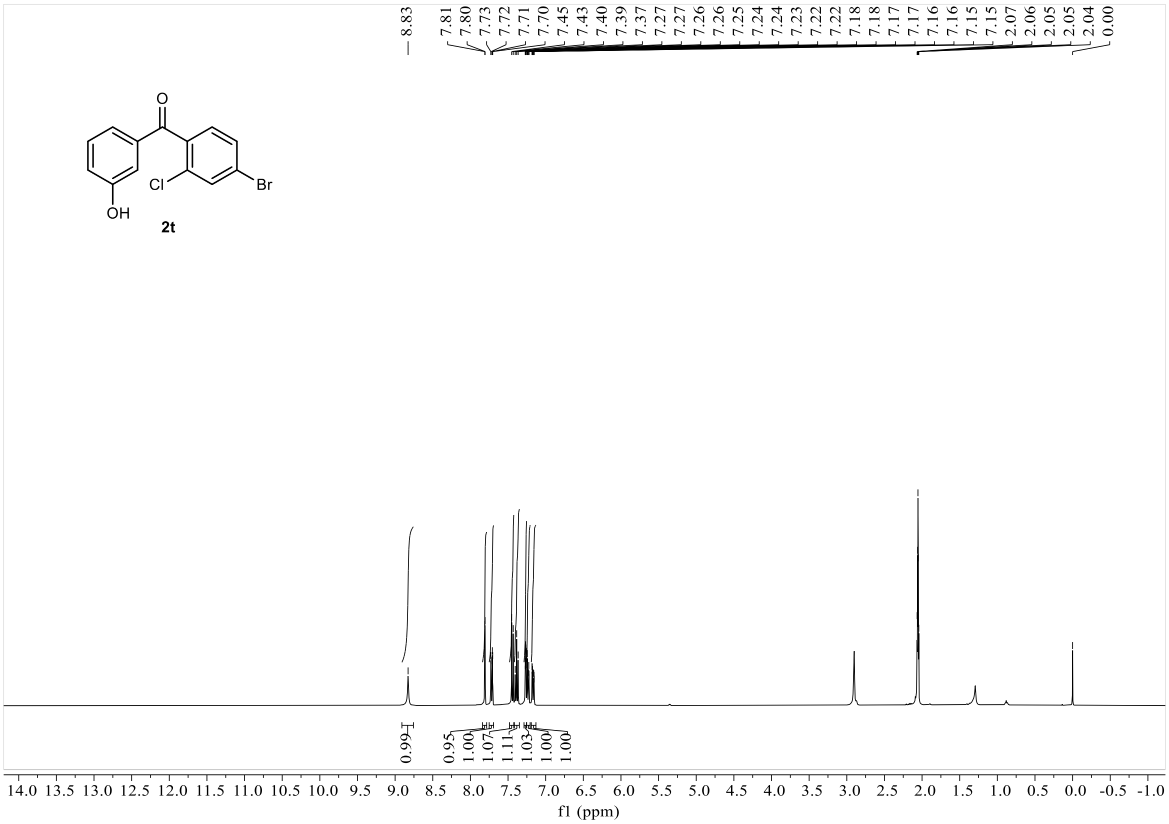

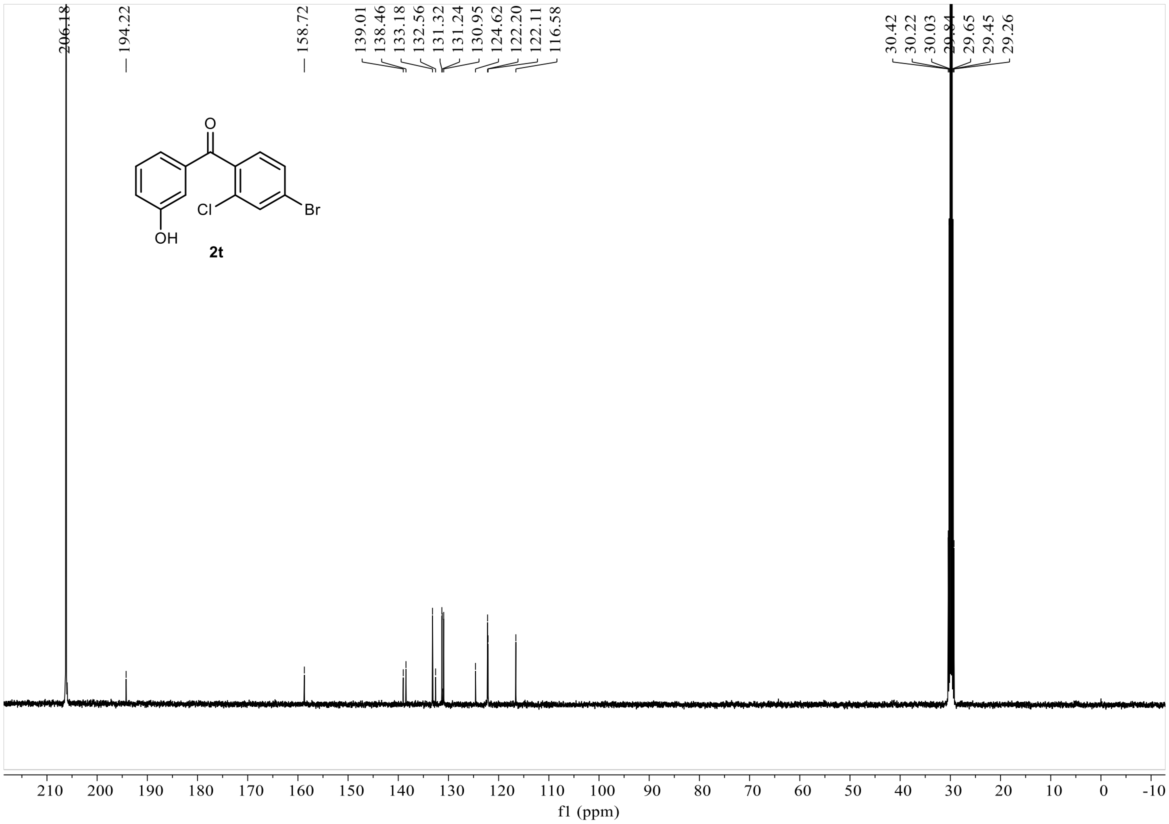

^{1}H NMR (400 MHz, Acetone-d_6) and ^{13}C NMR (101 MHz, Acetone-d_6) spectra of **2u**

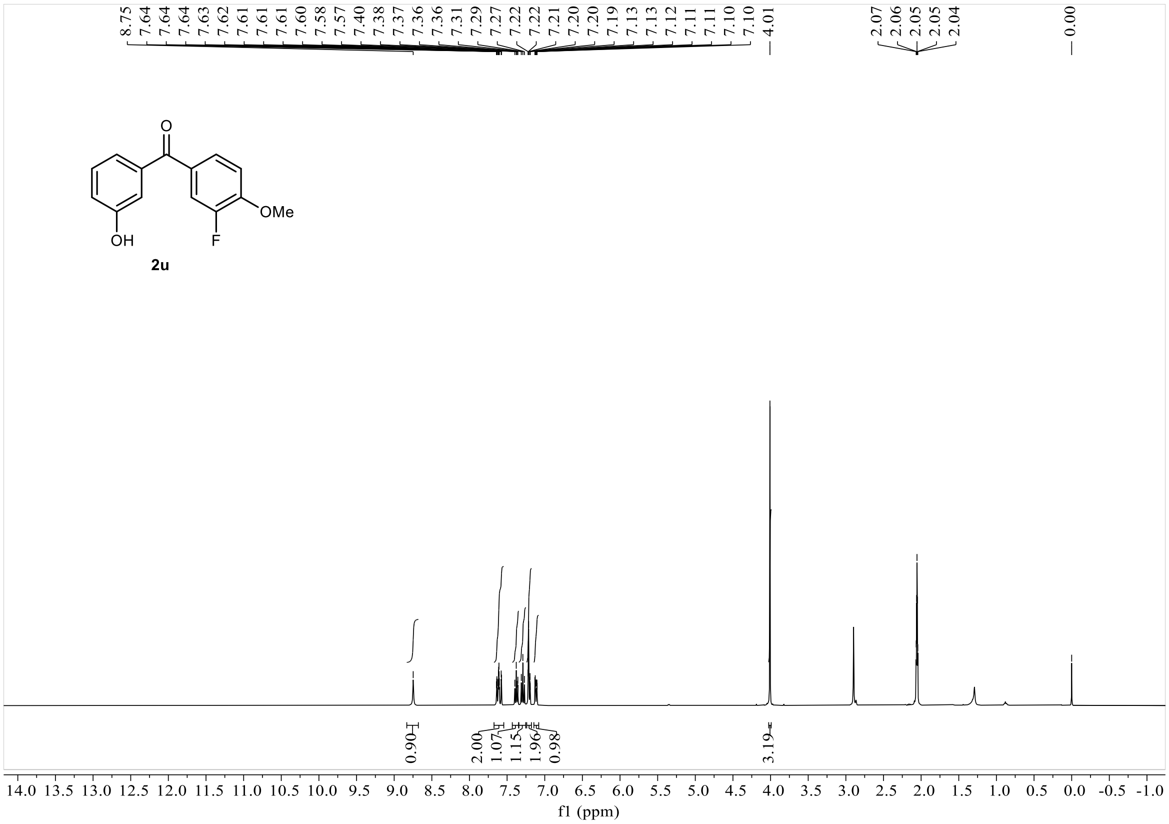

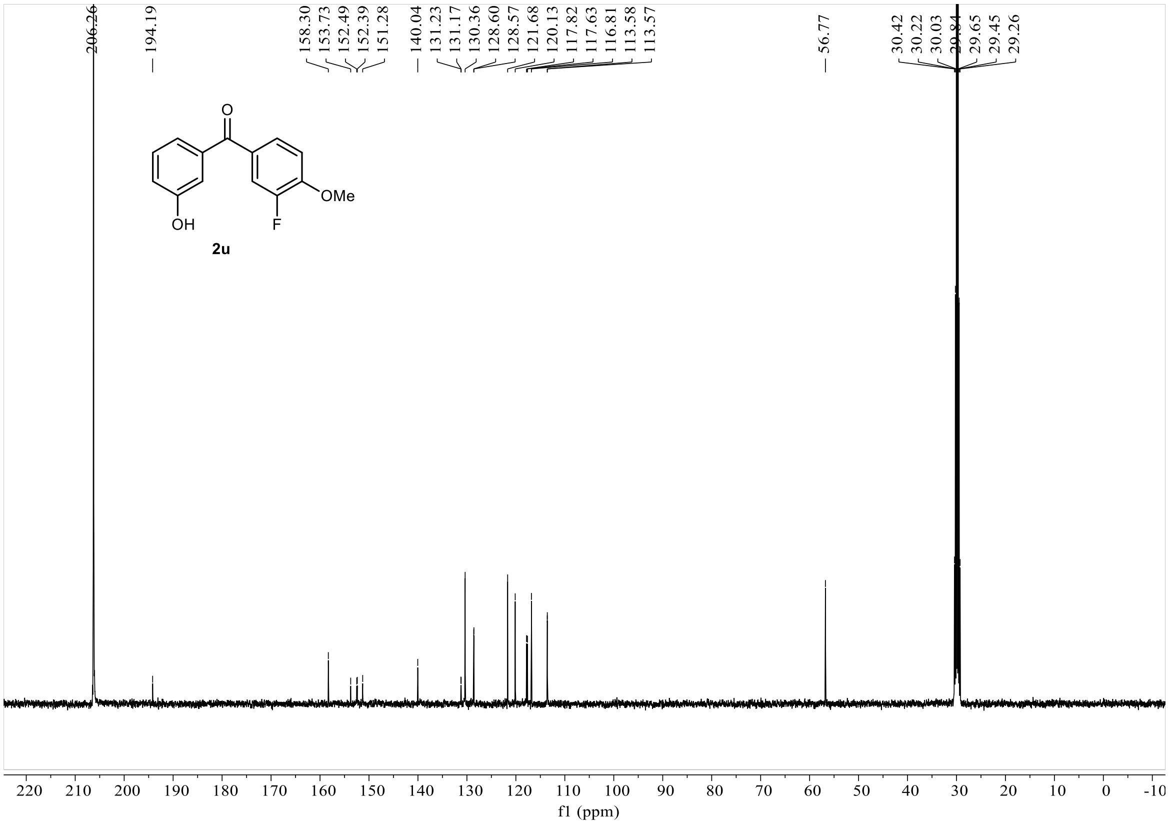

^{19}F NMR (376 MHz, Acetone-d_6) spectrum of **2u**

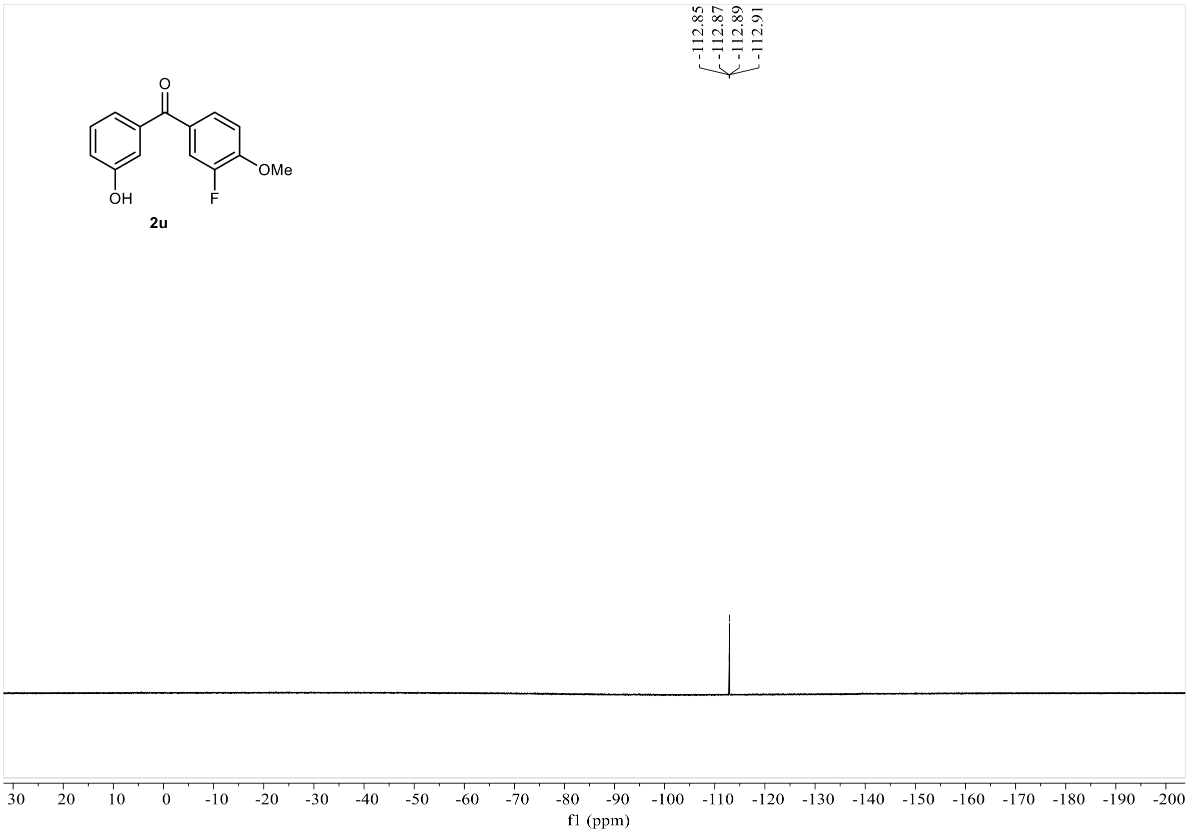

^{1}H NMR (400 MHz, $CDCl_3$) and ^{13}C NMR (101 MHz, $CDCl_3$) spectra of **2v**

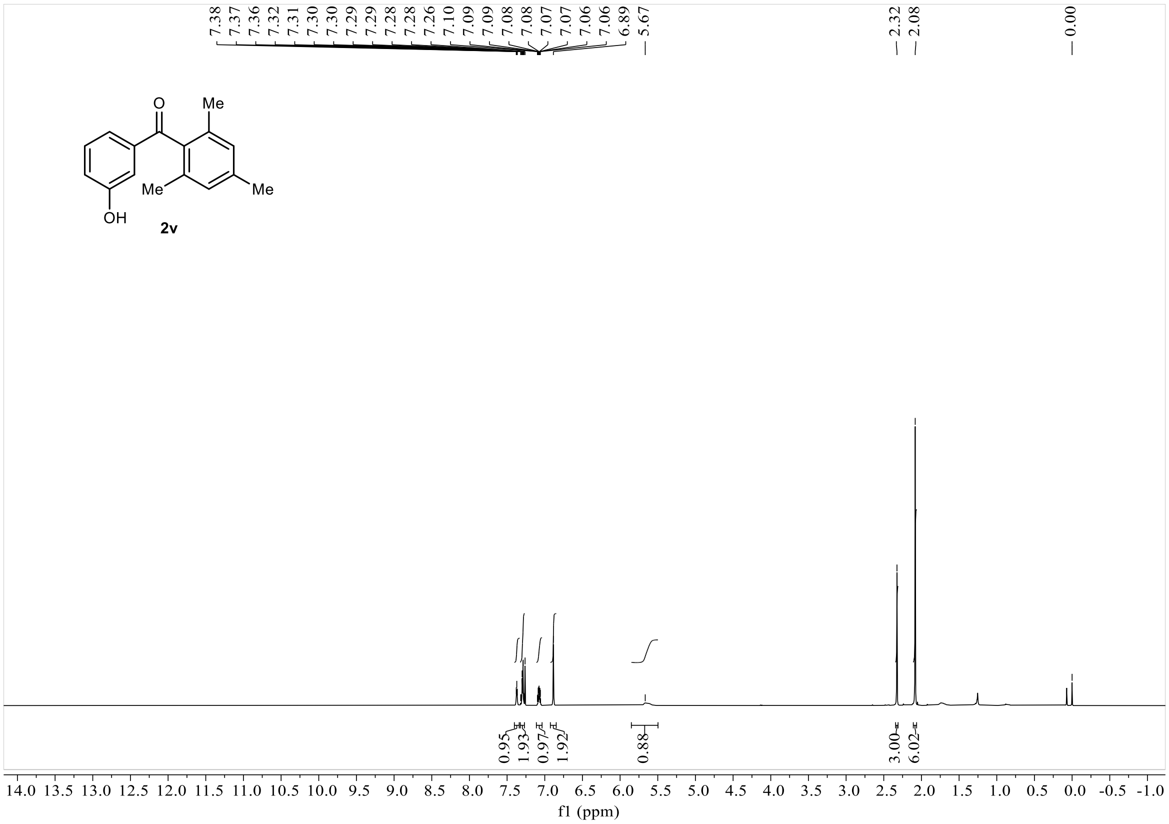

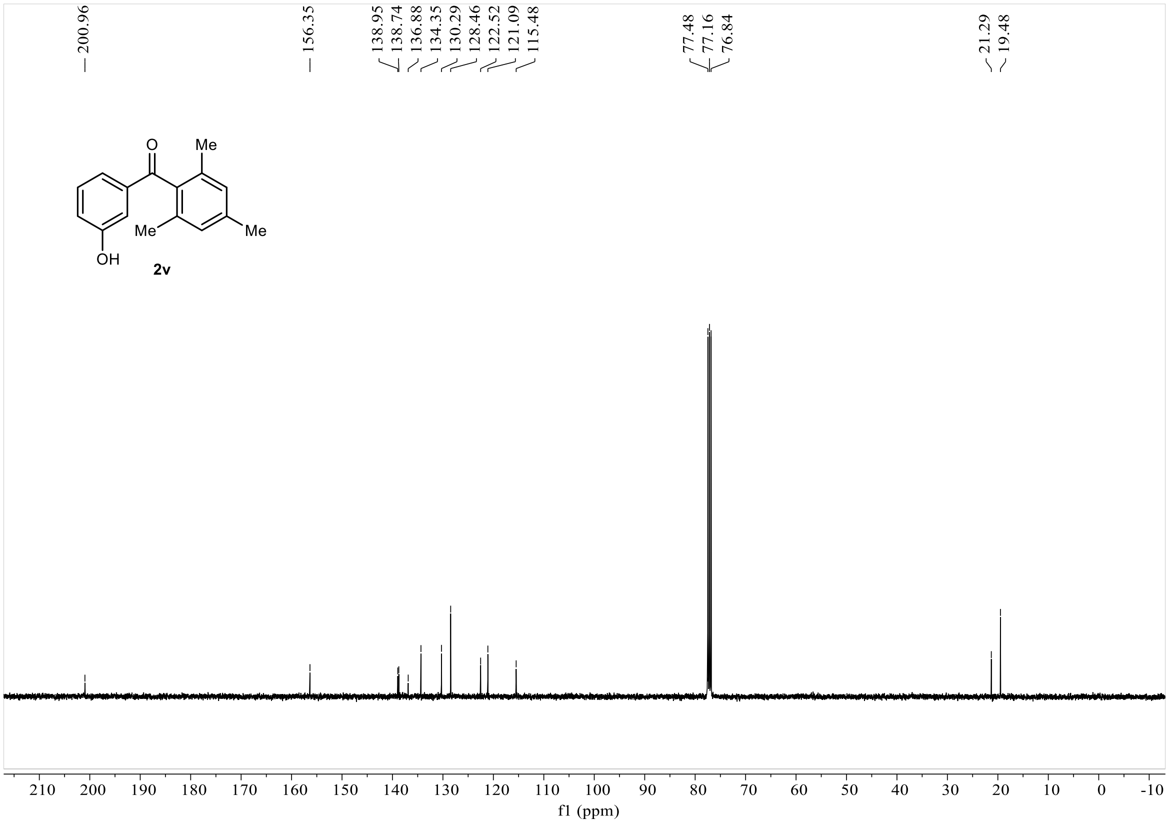

^{1}H NMR (400 MHz, Acetone-d_6) and ^{13}C NMR (101 MHz, Acetone-d_6) spectra of **2w**

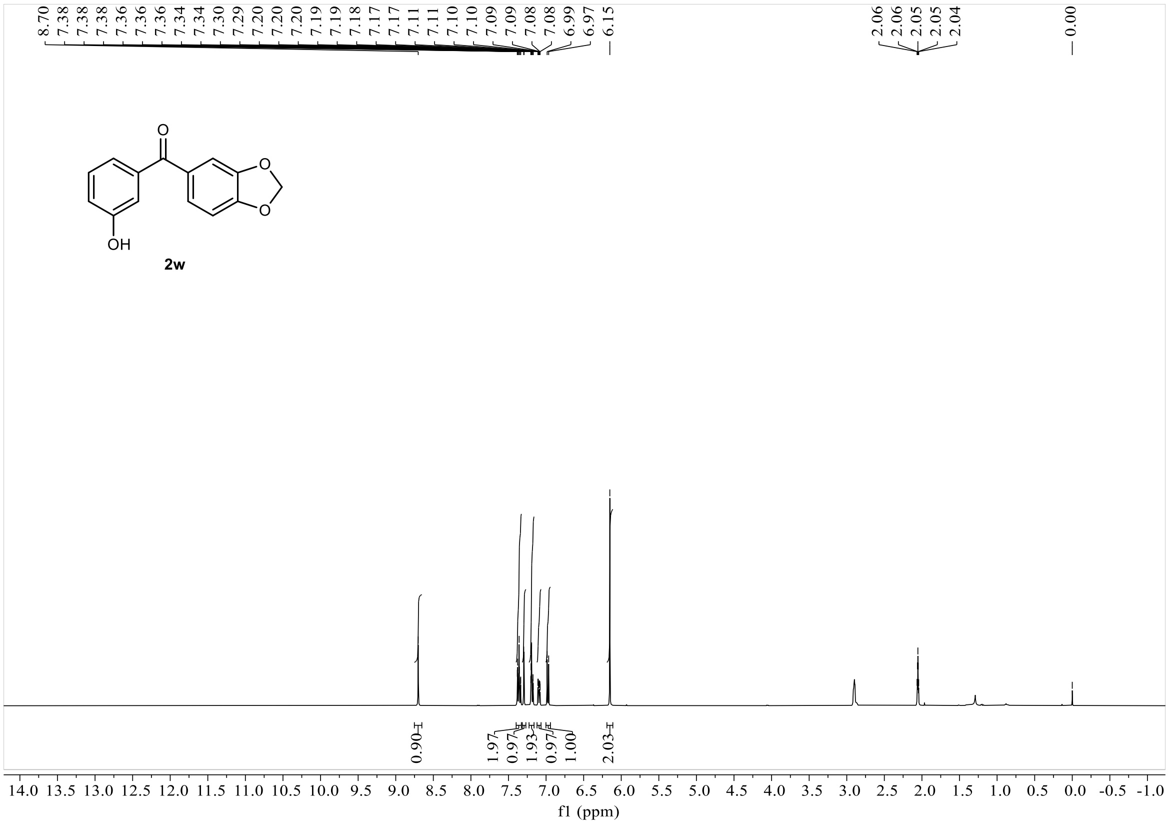

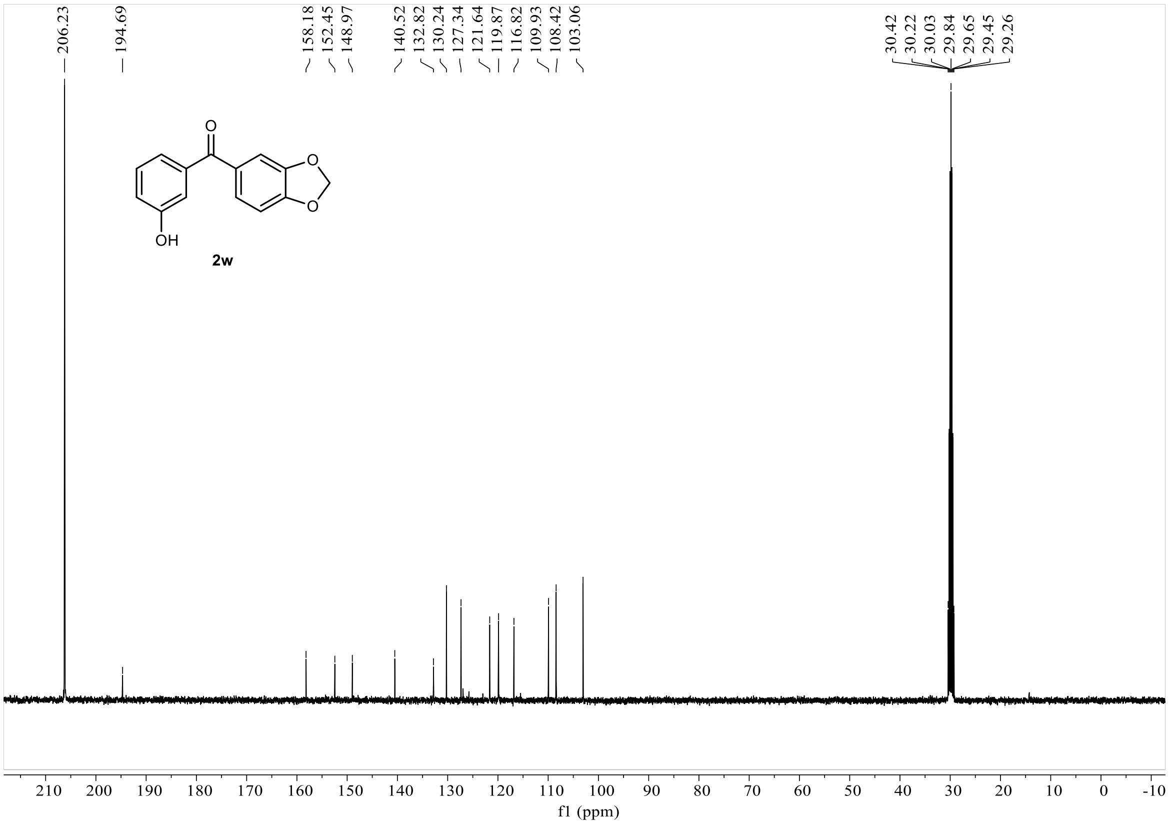

^{1}H NMR (400 MHz, Acetone-d_6) and ^{13}C NMR (101 MHz, Acetone-d_6) spectra of **2x**

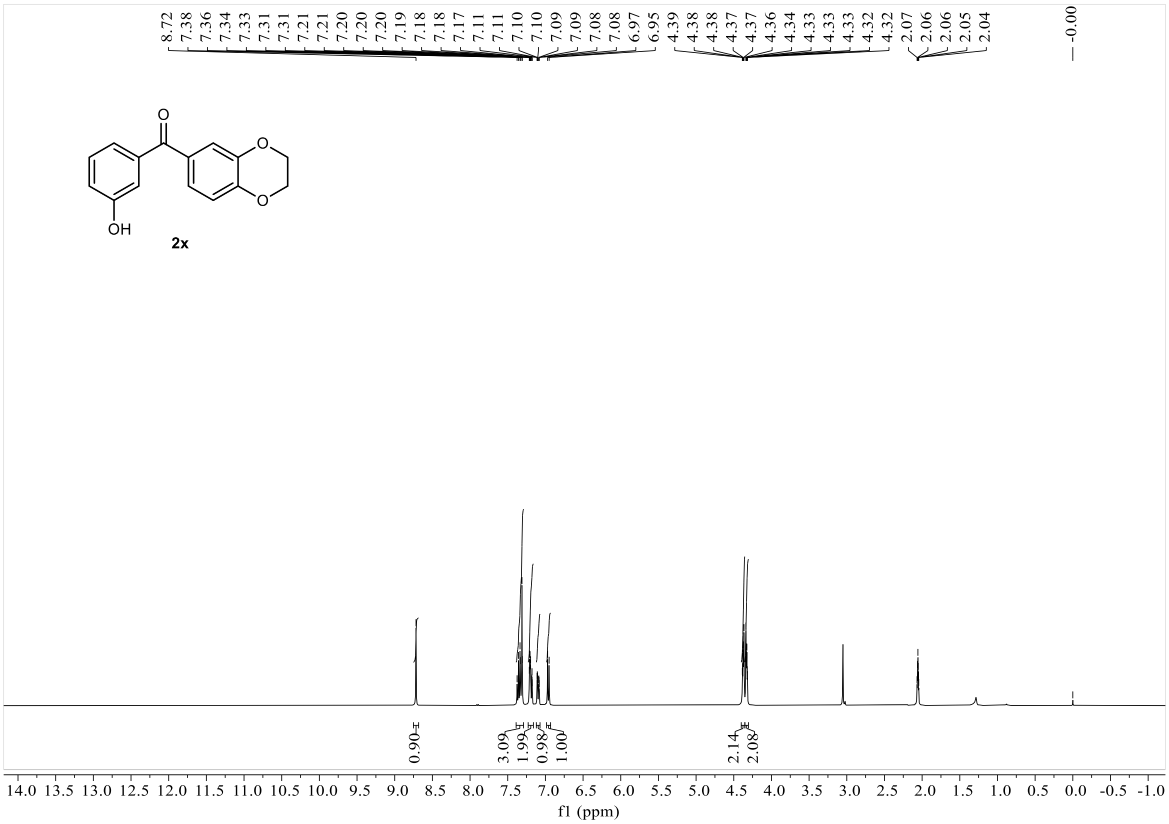

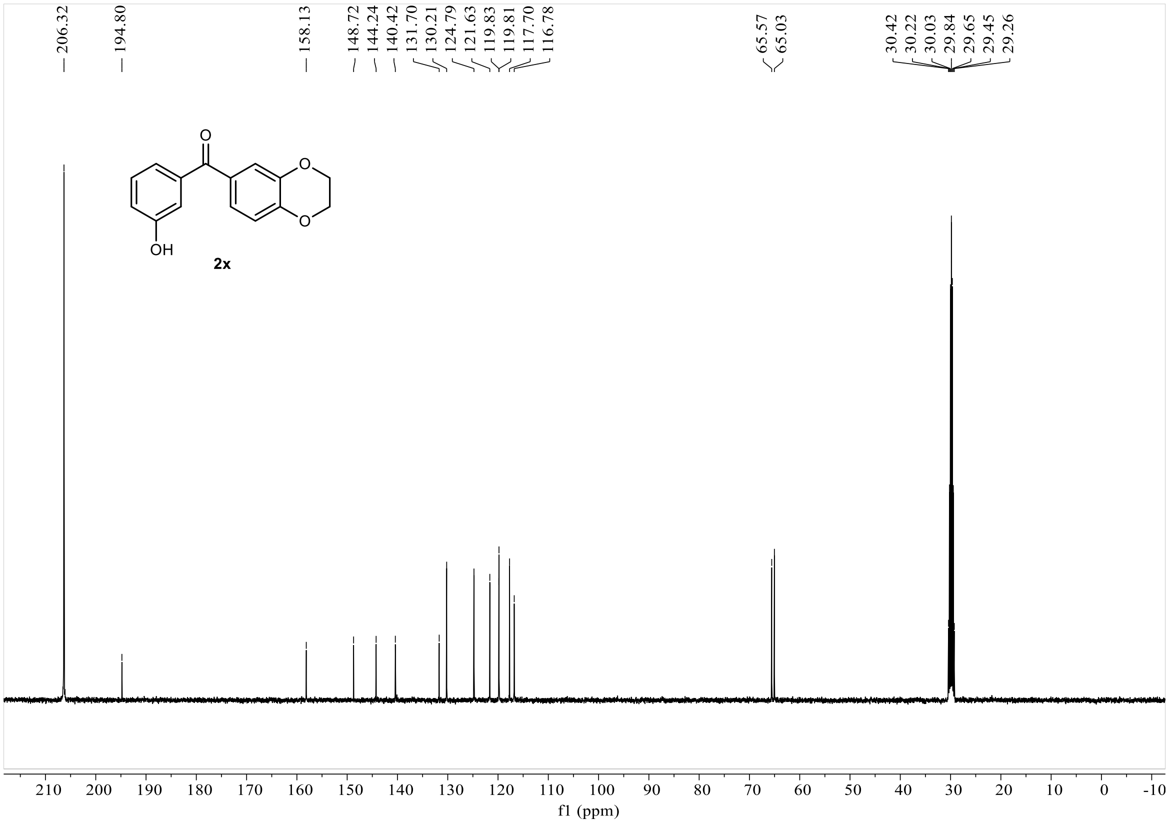

^{1}H NMR (400 MHz, $CDCl_3$) and ^{13}C NMR (101 MHz, $CDCl_3$) spectra of **2y**

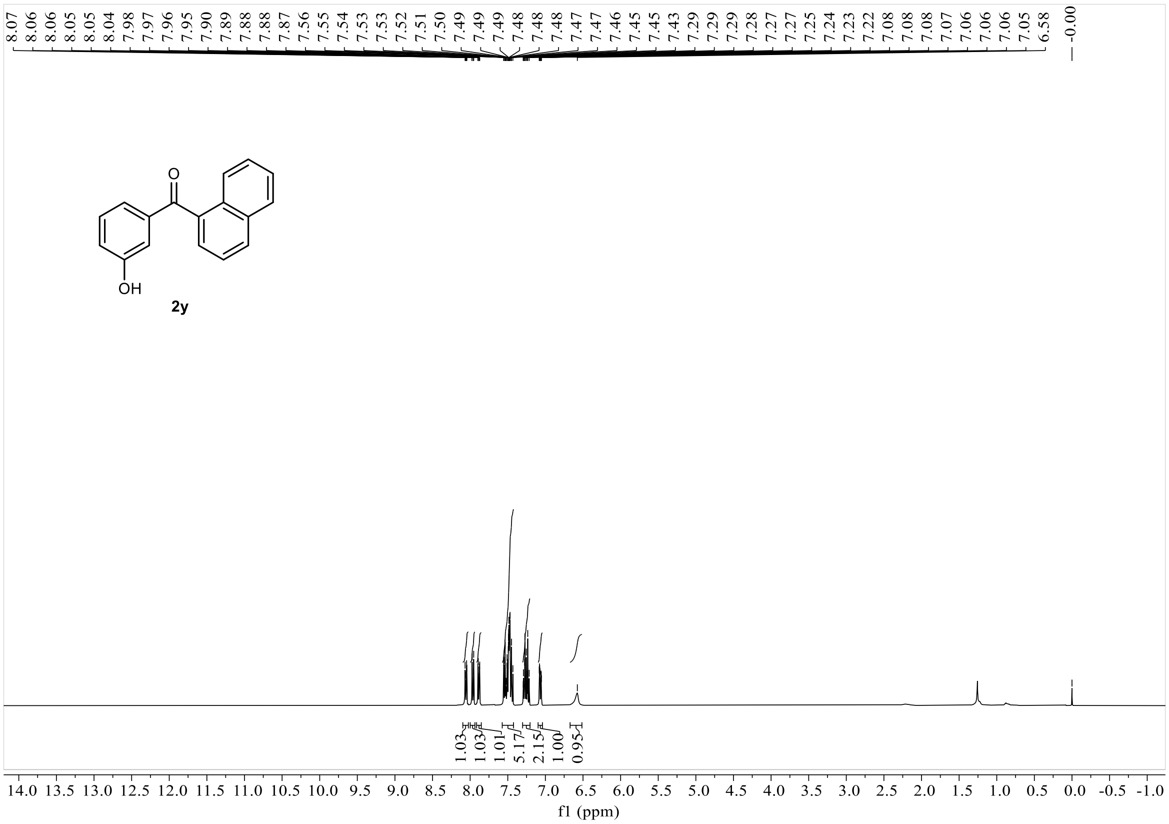

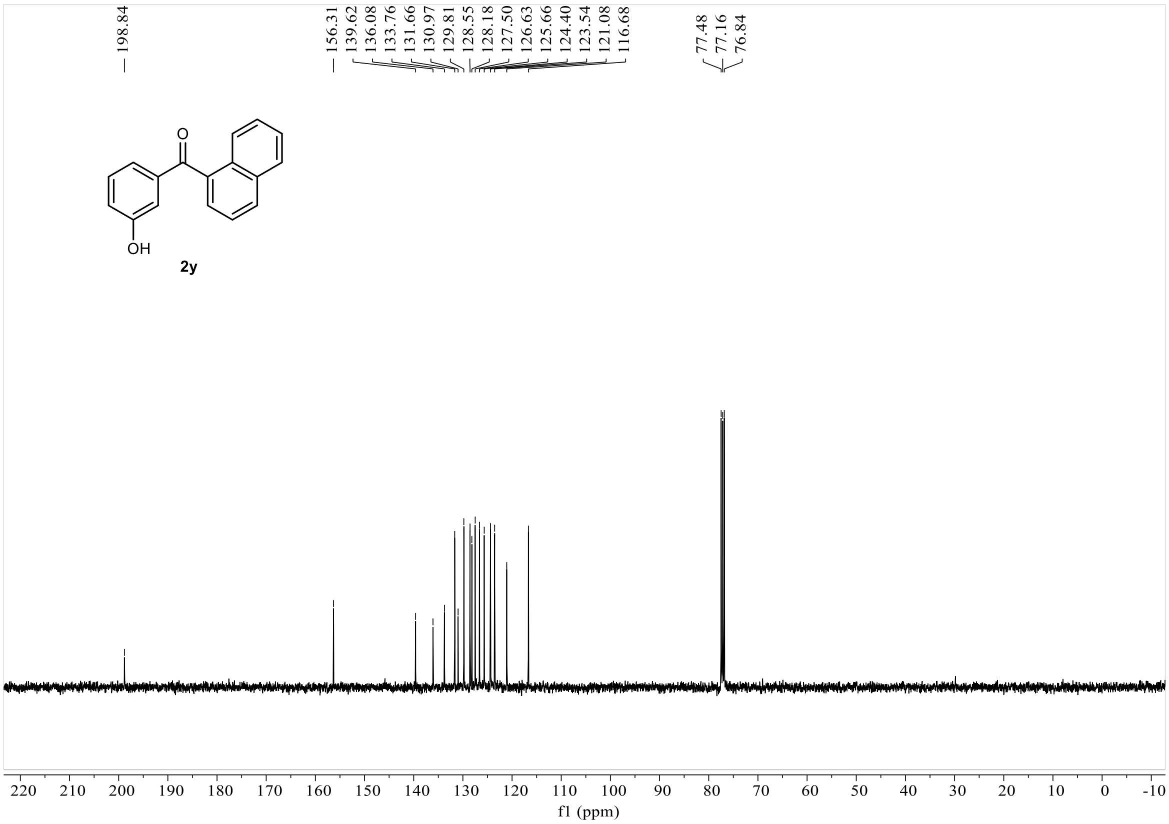

^{1}H NMR (400 MHz, Acetone-d_6) and ^{13}C NMR (101 MHz, Acetone-d_6) spectra of **2z**

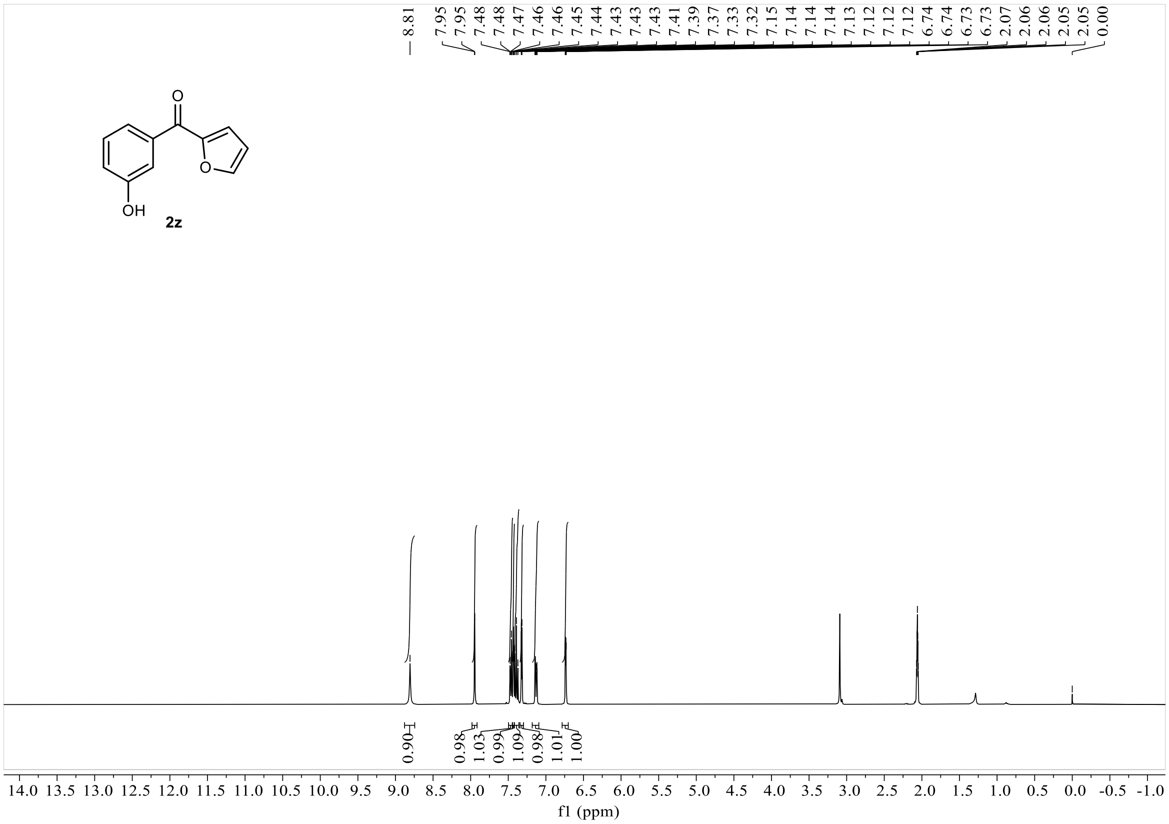

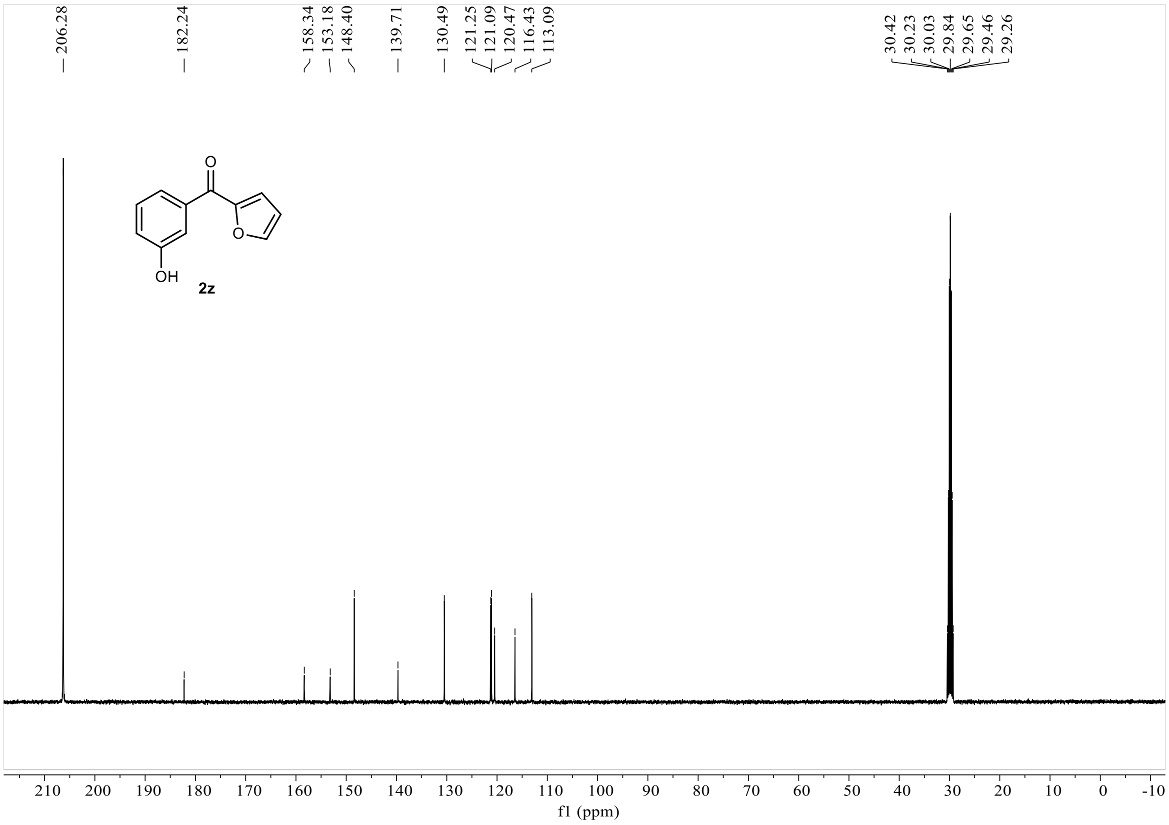

^{1}H NMR (400 MHz, Acetone-d_6) and ^{13}C NMR (101 MHz, Acetone-d_6) spectra of **2aa**

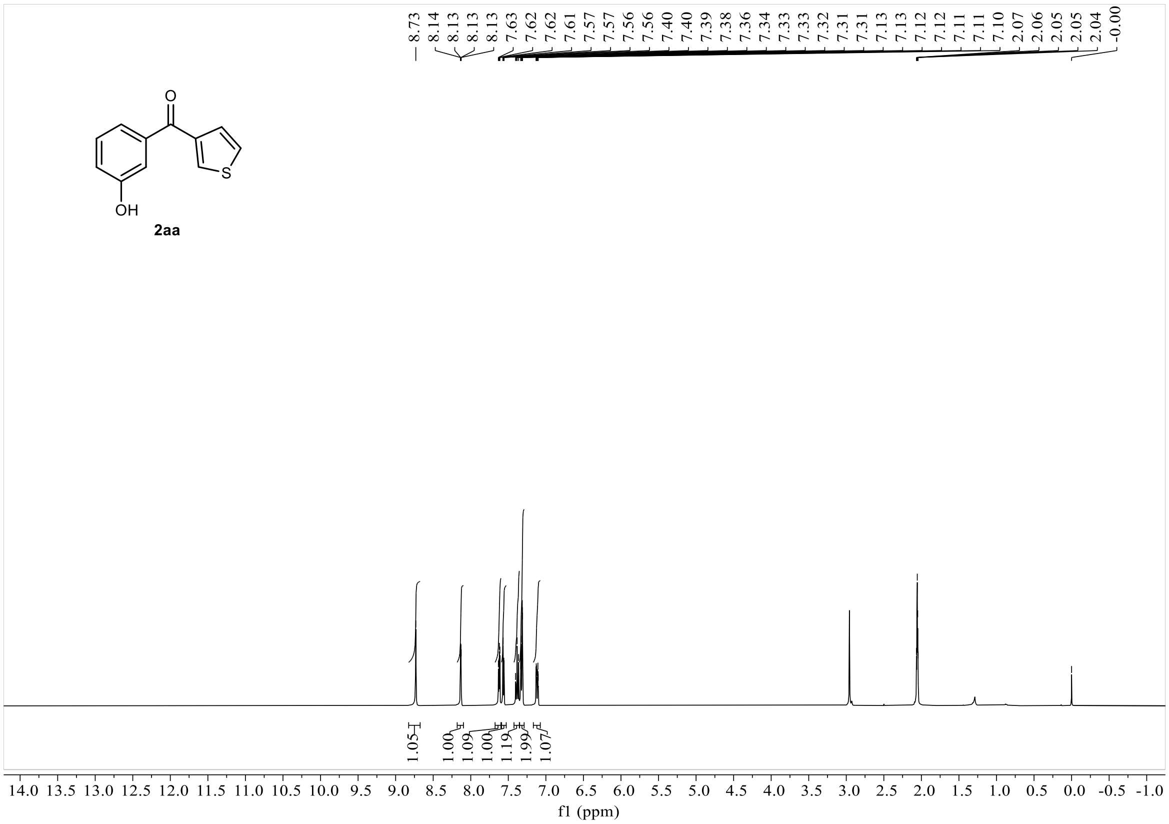

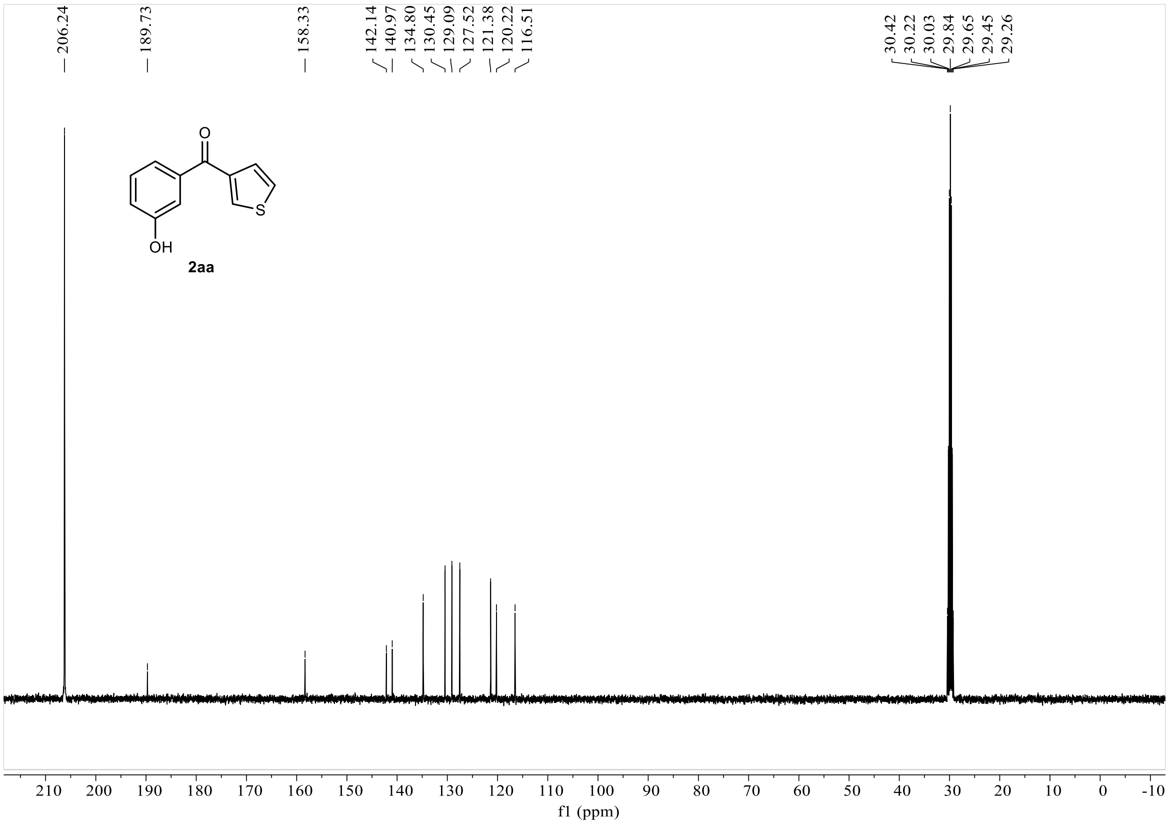

^{1}H NMR (400 MHz, Acetone-d_6) and ^{13}C NMR (101 MHz, Acetone-d_6) spectra of **2ab**

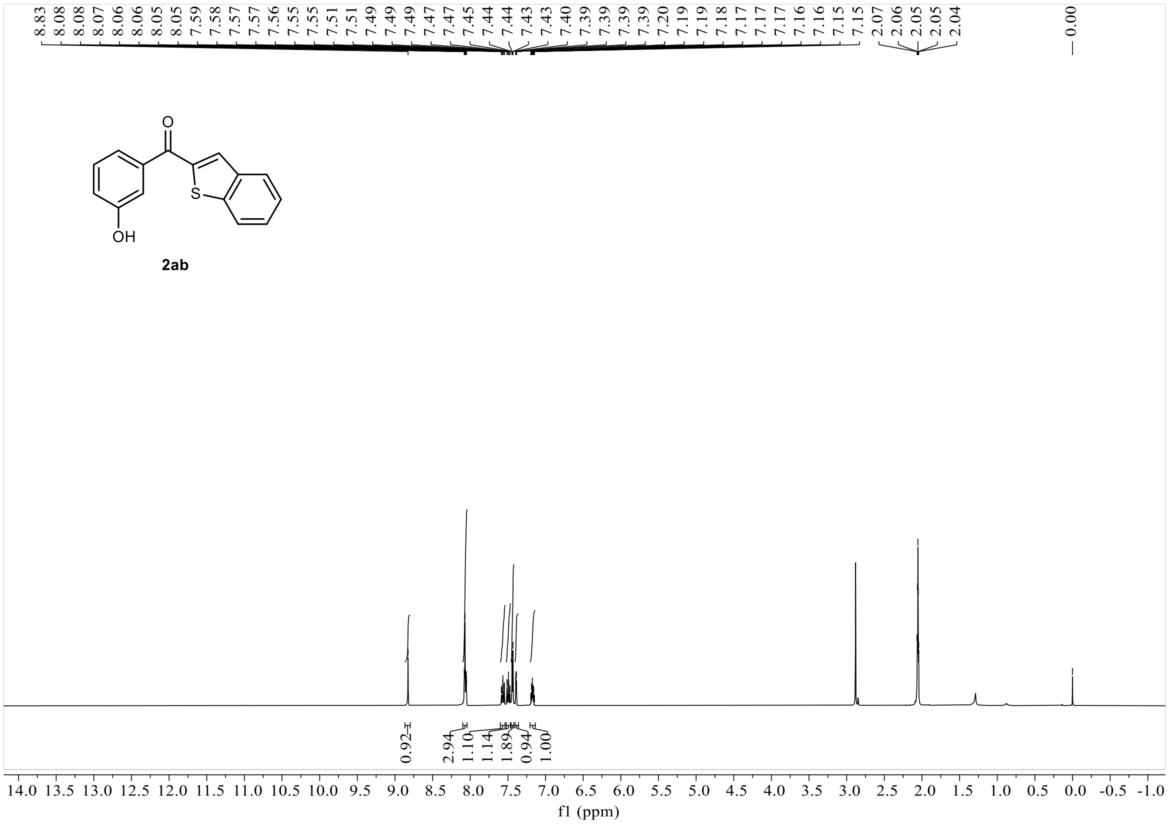

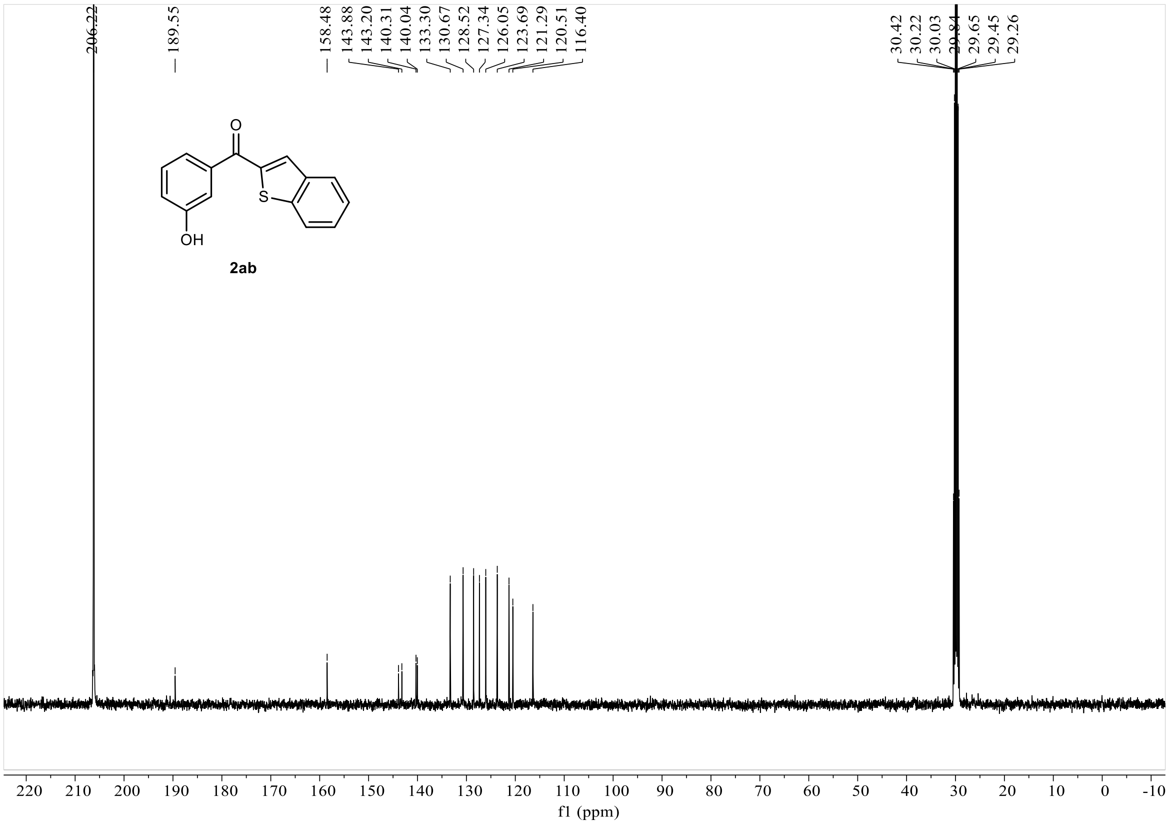

^{1}H NMR (400 MHz, $CDCl_3$) and ^{13}C NMR (101 MHz, $CDCl_3$) spectra of **5**

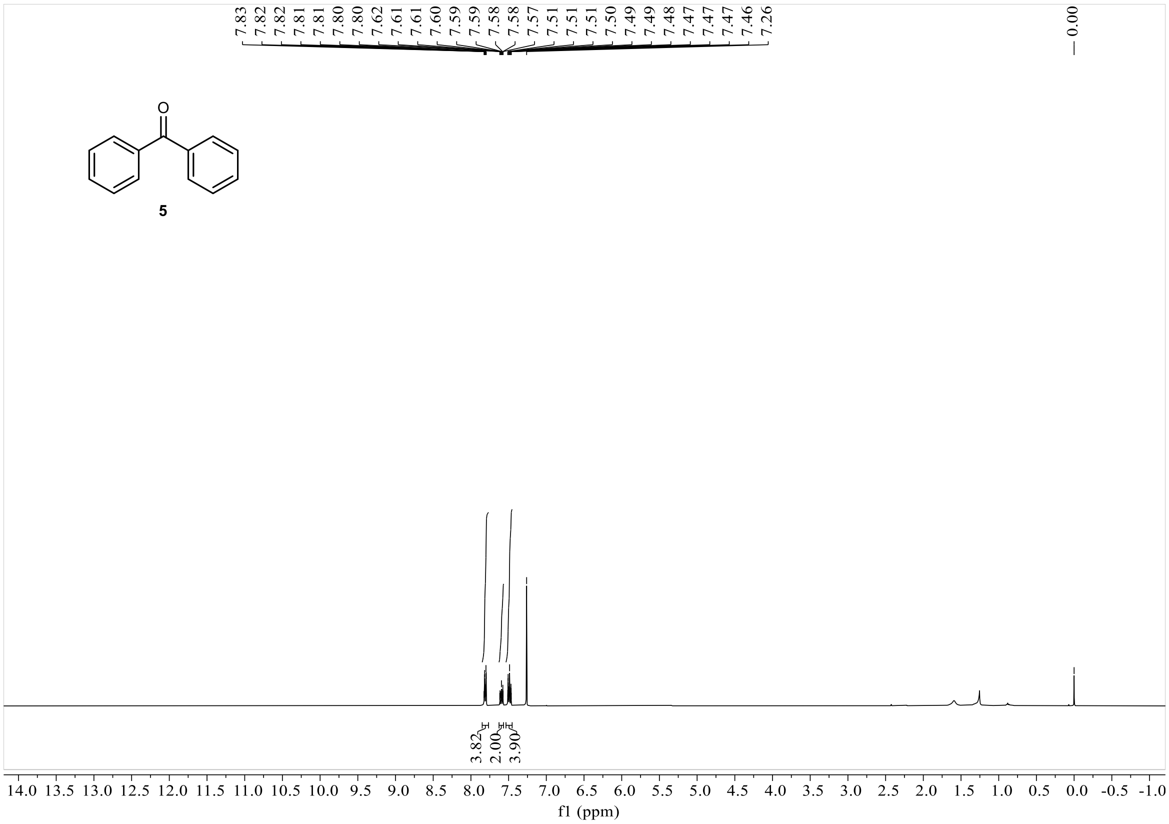

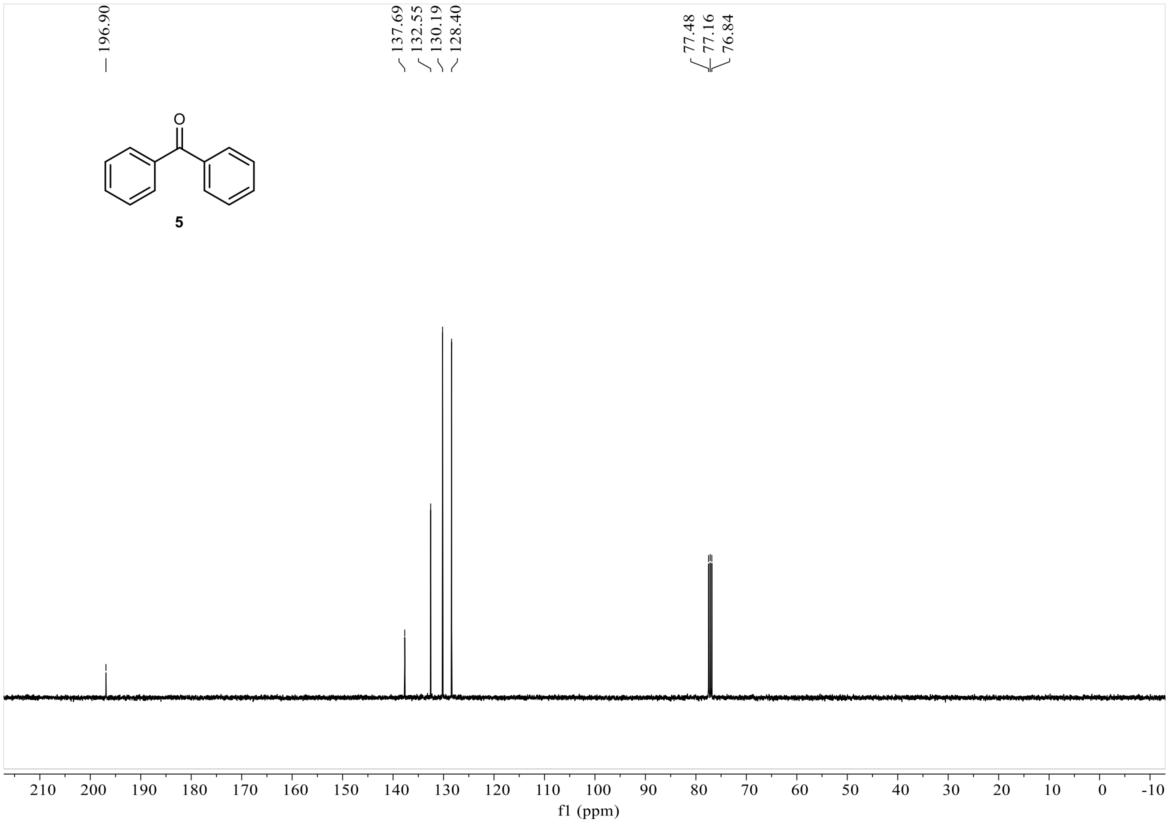

^{1}H NMR (400 MHz, $CDCl_3$) and ^{13}C NMR (101 MHz, $CDCl_3$) spectra of **6**

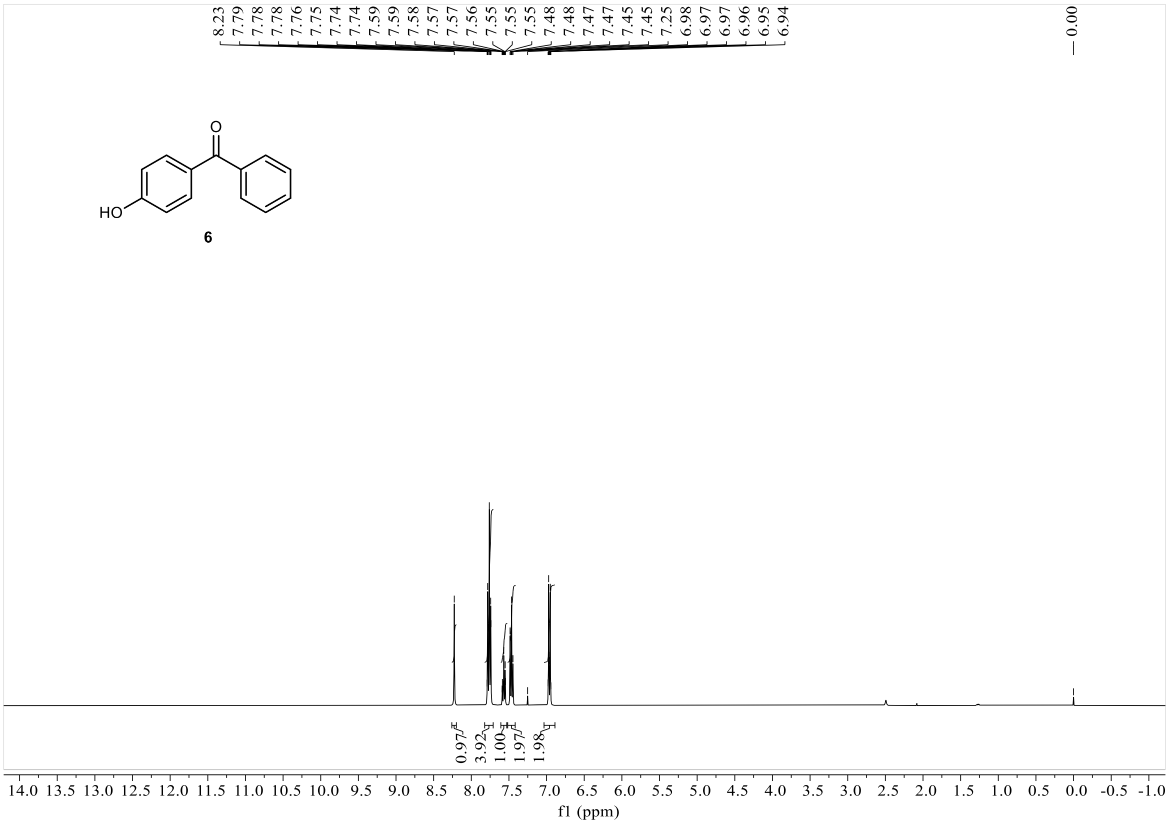

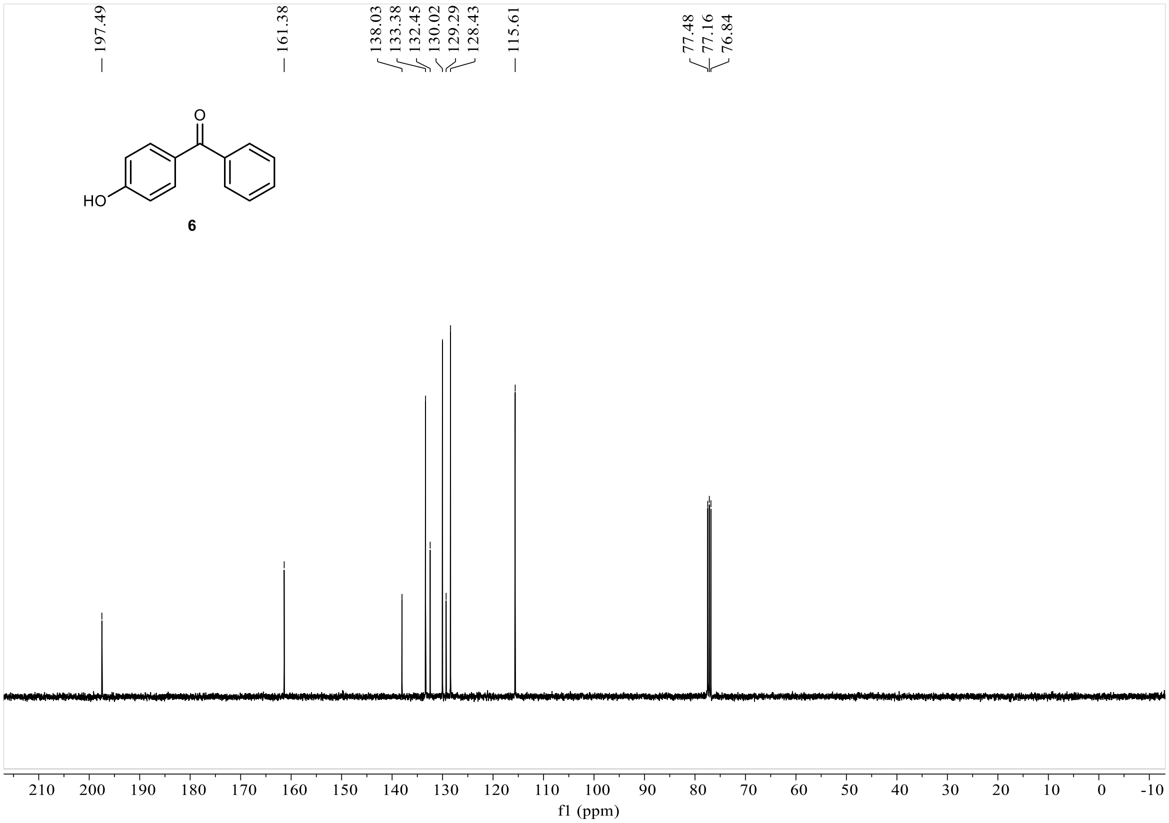

^{1}H NMR (400 MHz, $CDCl_3$) and ^{13}C NMR (101 MHz, $CDCl_3$) spectra of **8a**

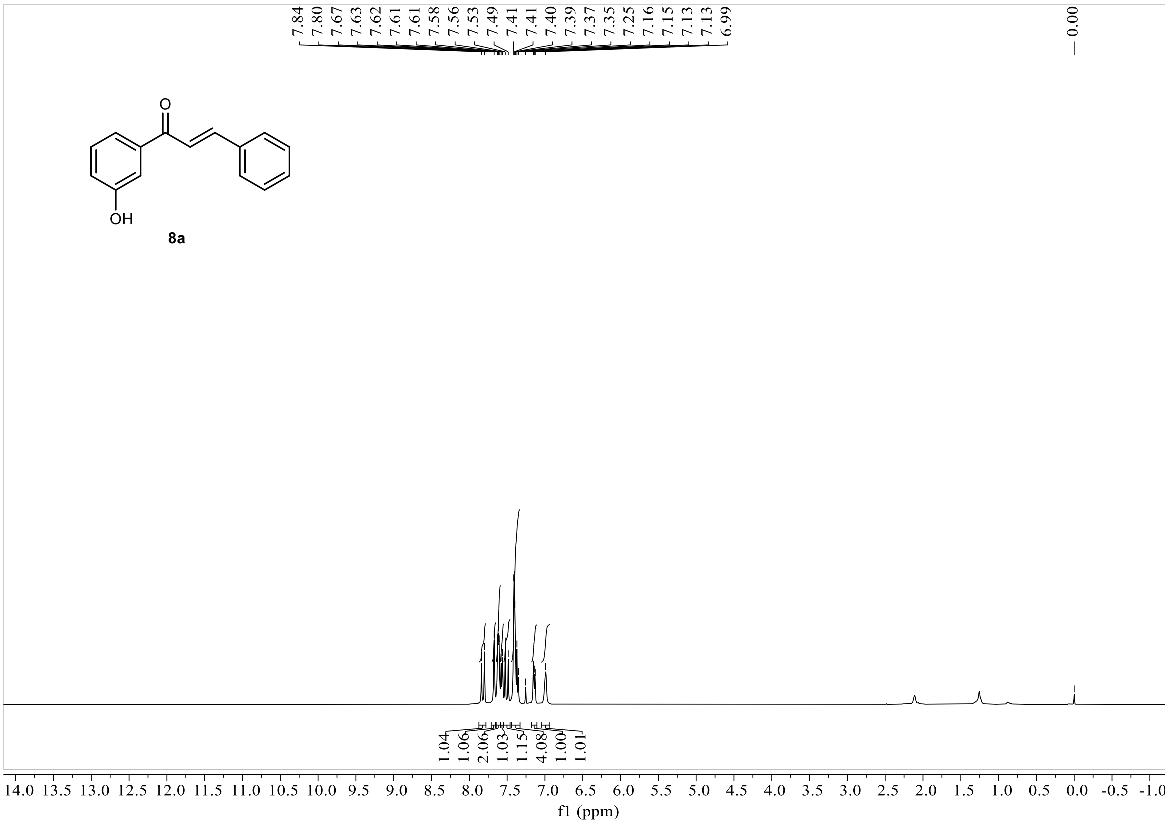

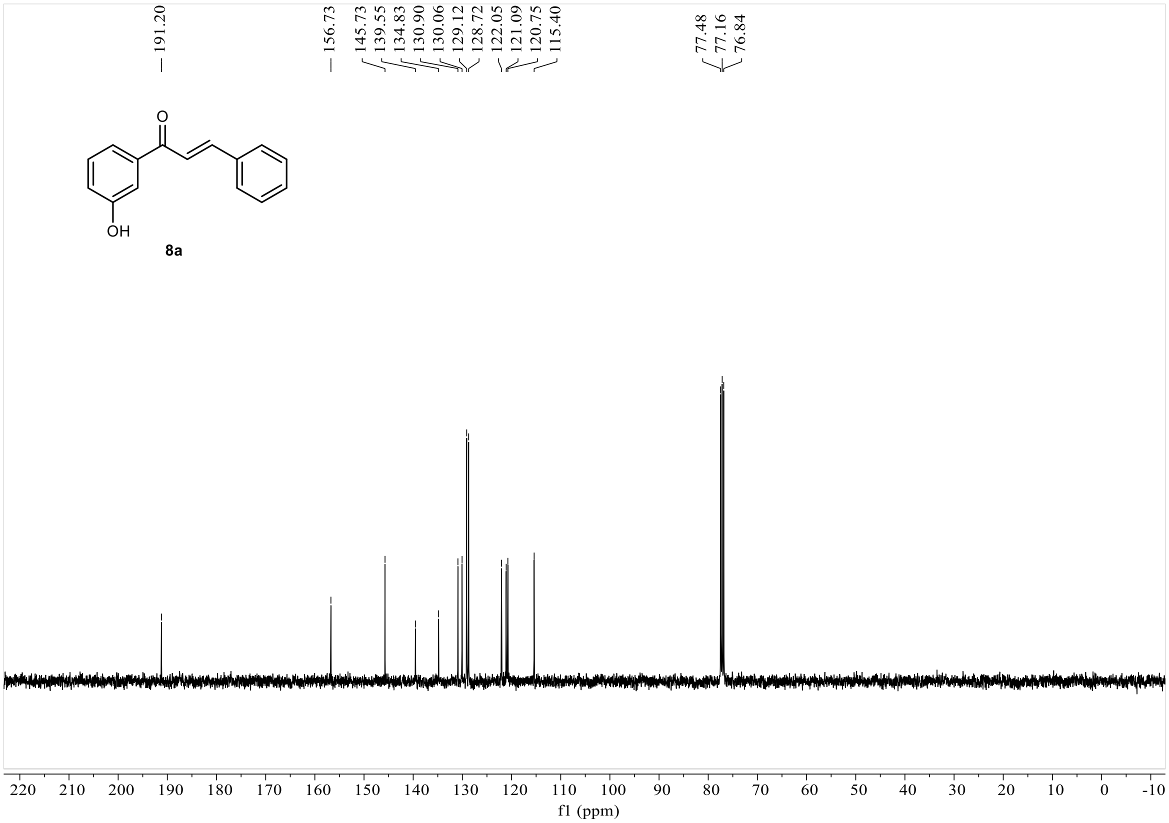

^{1}H NMR (400 MHz, Acetone-d_6) and ^{13}C NMR (101 MHz, Acetone-d_6) spectra of **8b**

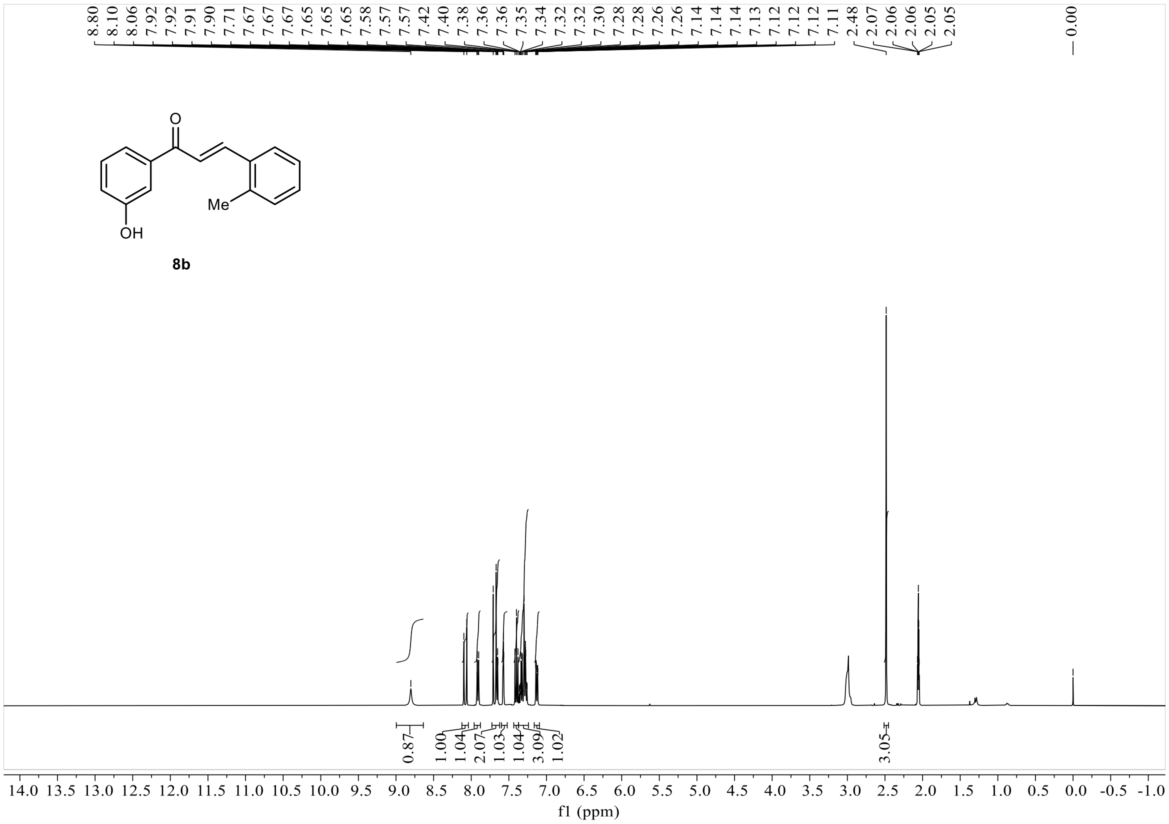

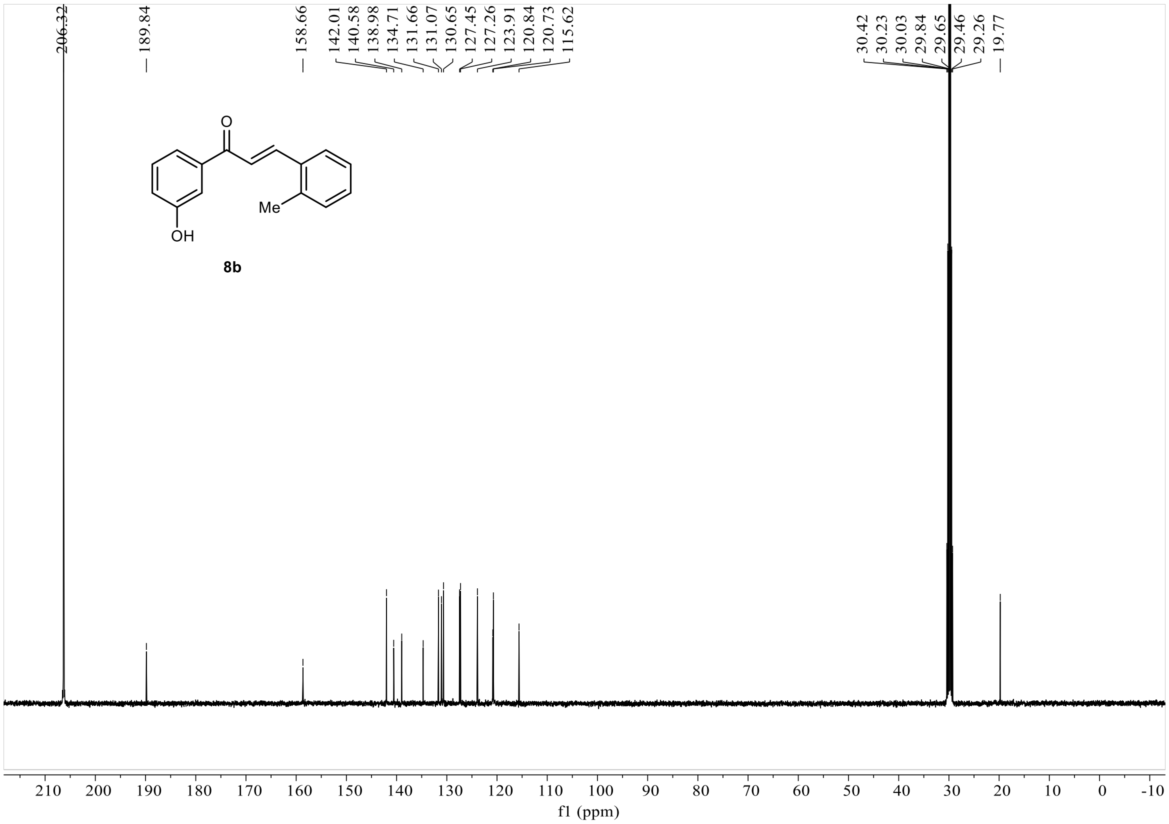

^{1}H NMR (400 MHz, Acetone-d_6) and ^{13}C NMR (101 MHz, Acetone-d_6) spectra of **8c**

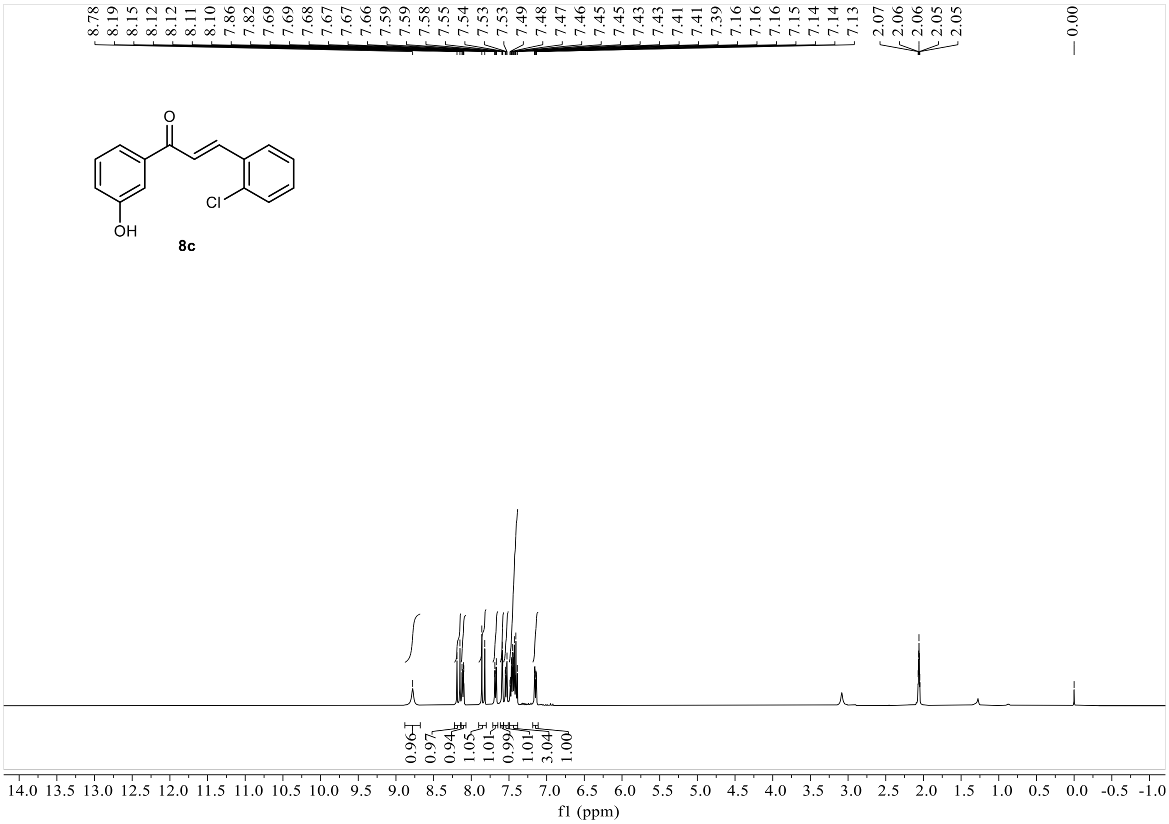

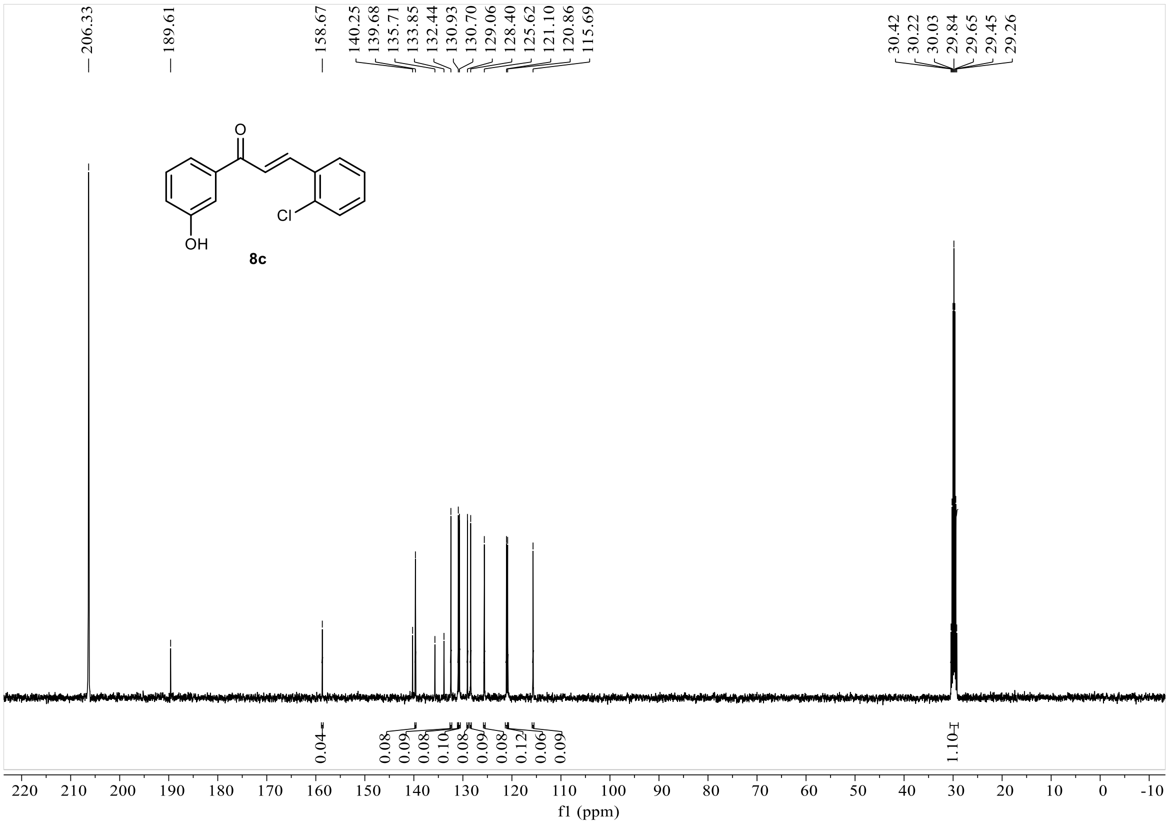

^{1}H NMR (400 MHz, $CDCl_3$) and ^{13}C NMR (101 MHz, $CDCl_3$) spectra of **8d**

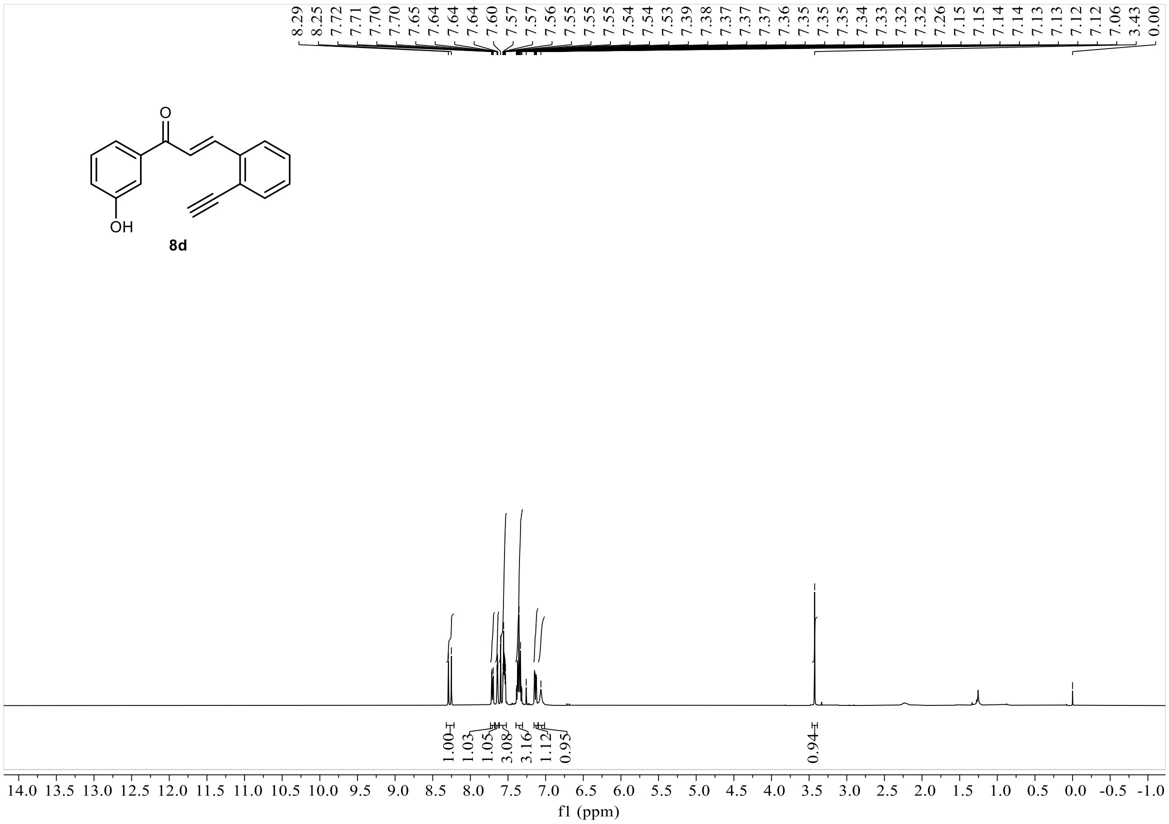

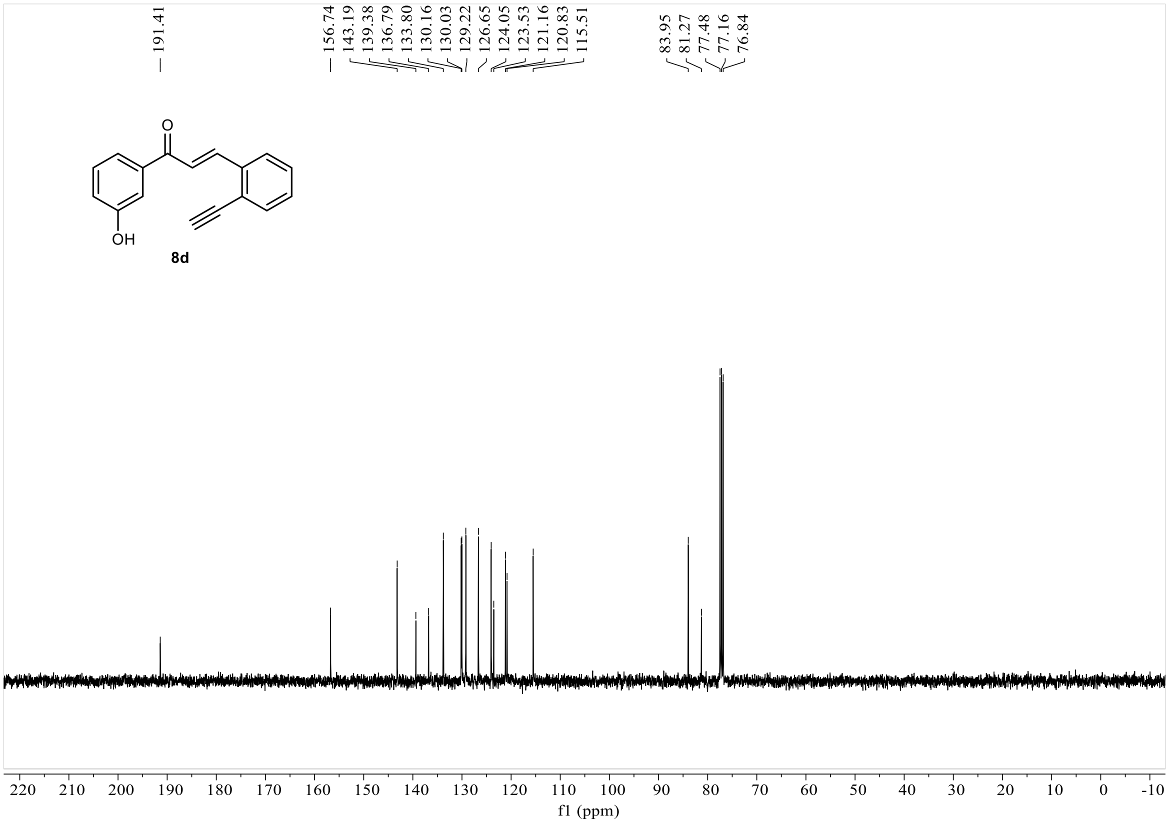

^{1}H NMR (400 MHz, Acetone-d_6) and ^{13}C NMR (101 MHz, Acetone-d_6) spectra of **8e**

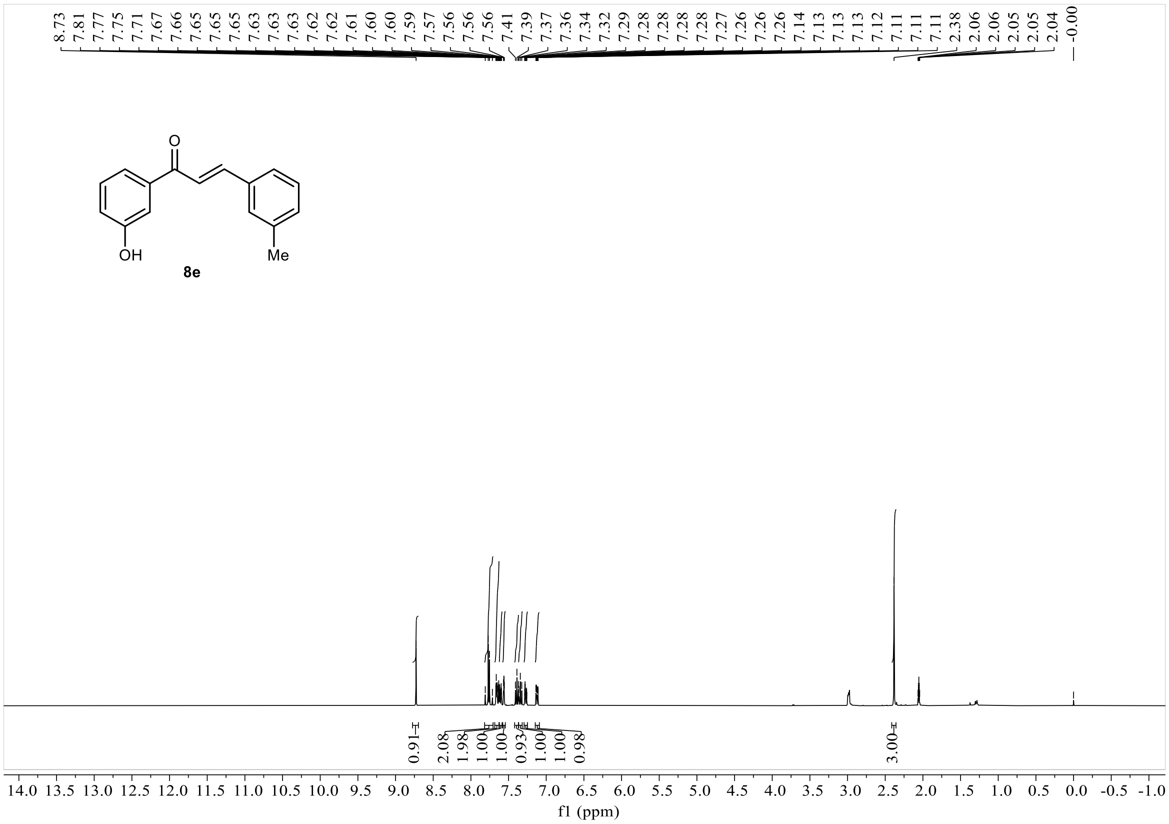

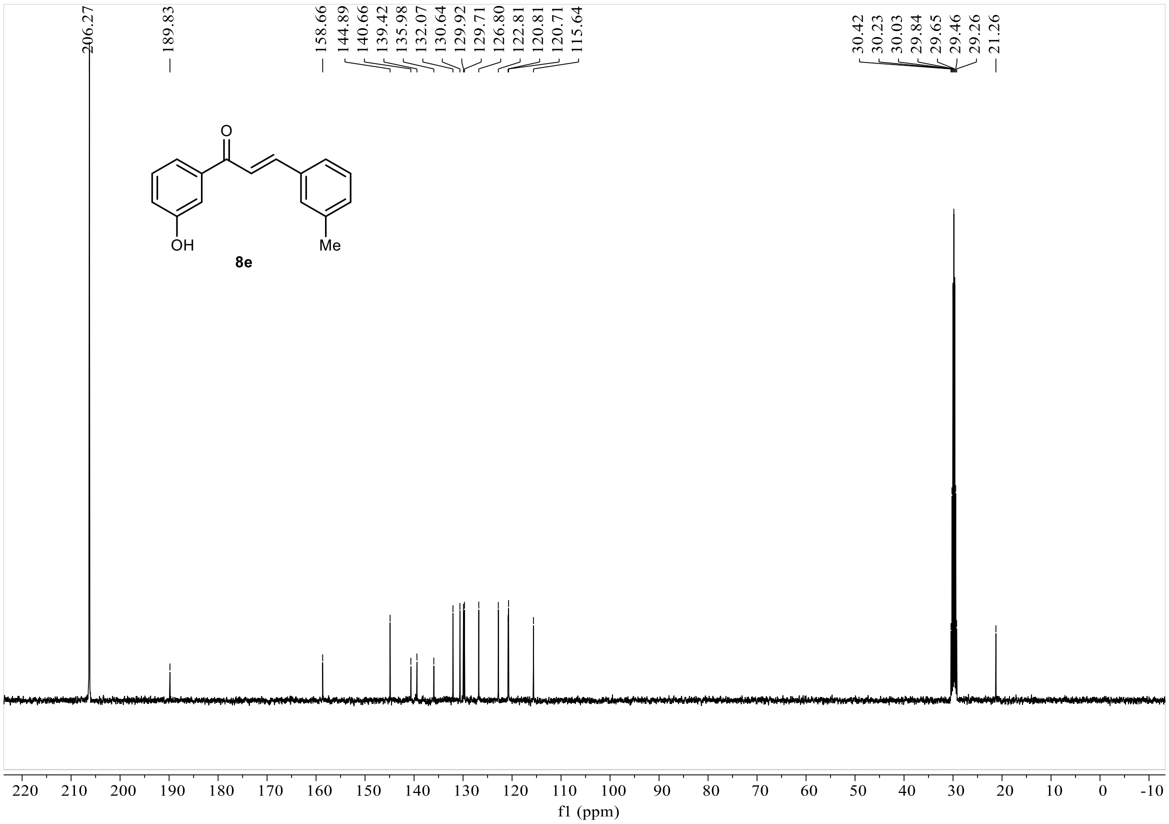

^{1}H NMR (400 MHz, Acetone-d_6) and ^{13}C NMR (101 MHz, Acetone-d_6) spectra of **8f**

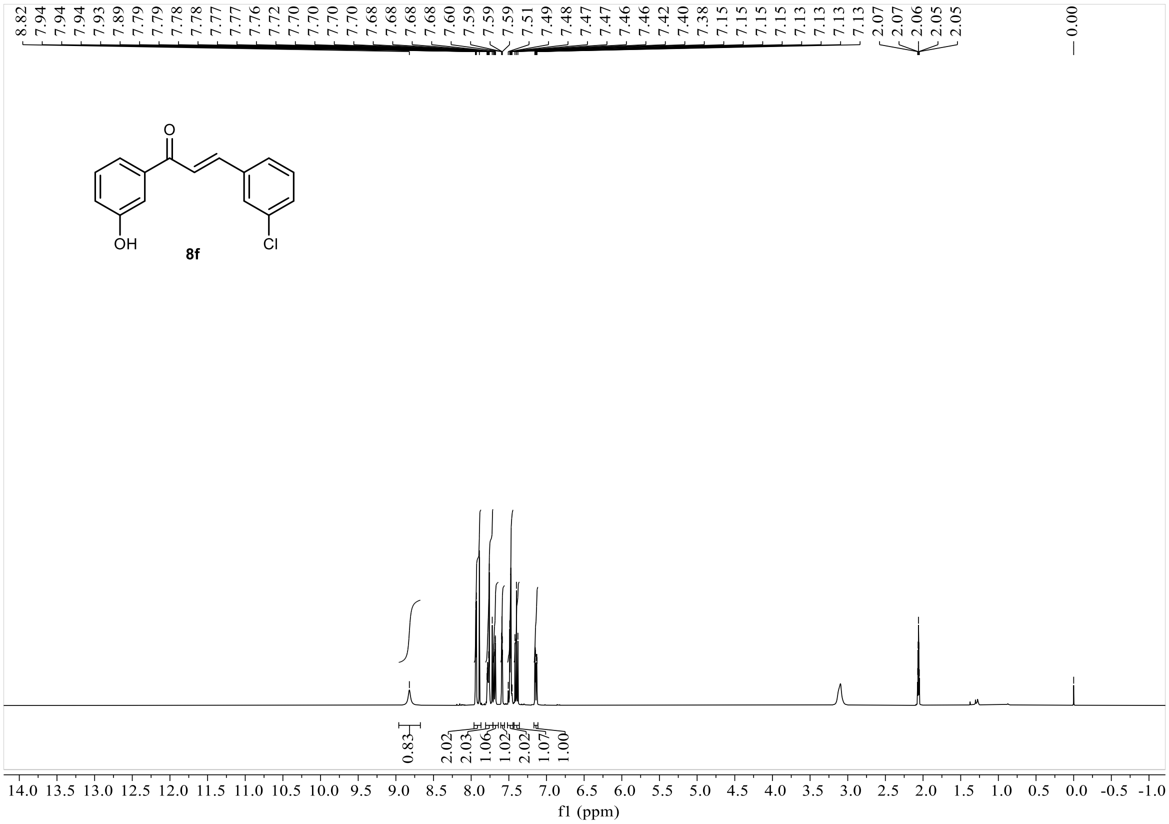

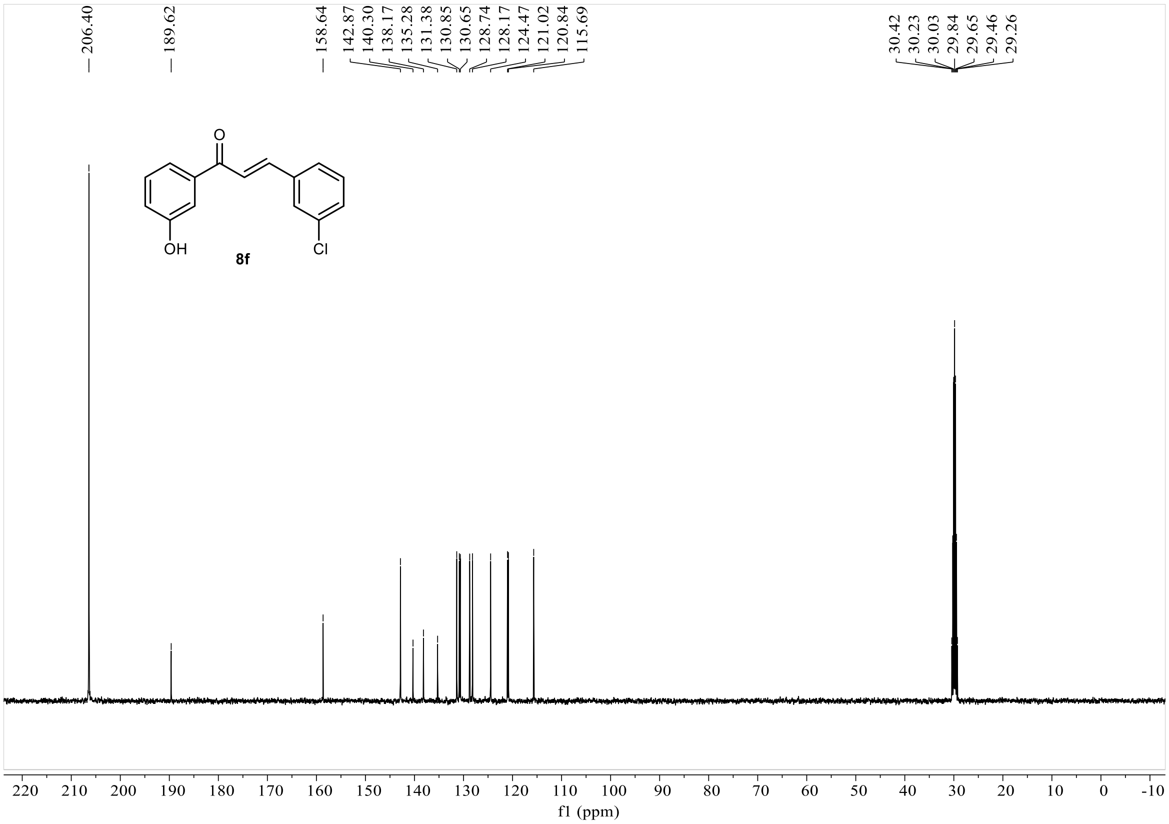

^1H NMR (400 MHz, Acetone-d_6) and ^{13}C NMR (101 MHz, Acetone-d_6) spectra of **8g**

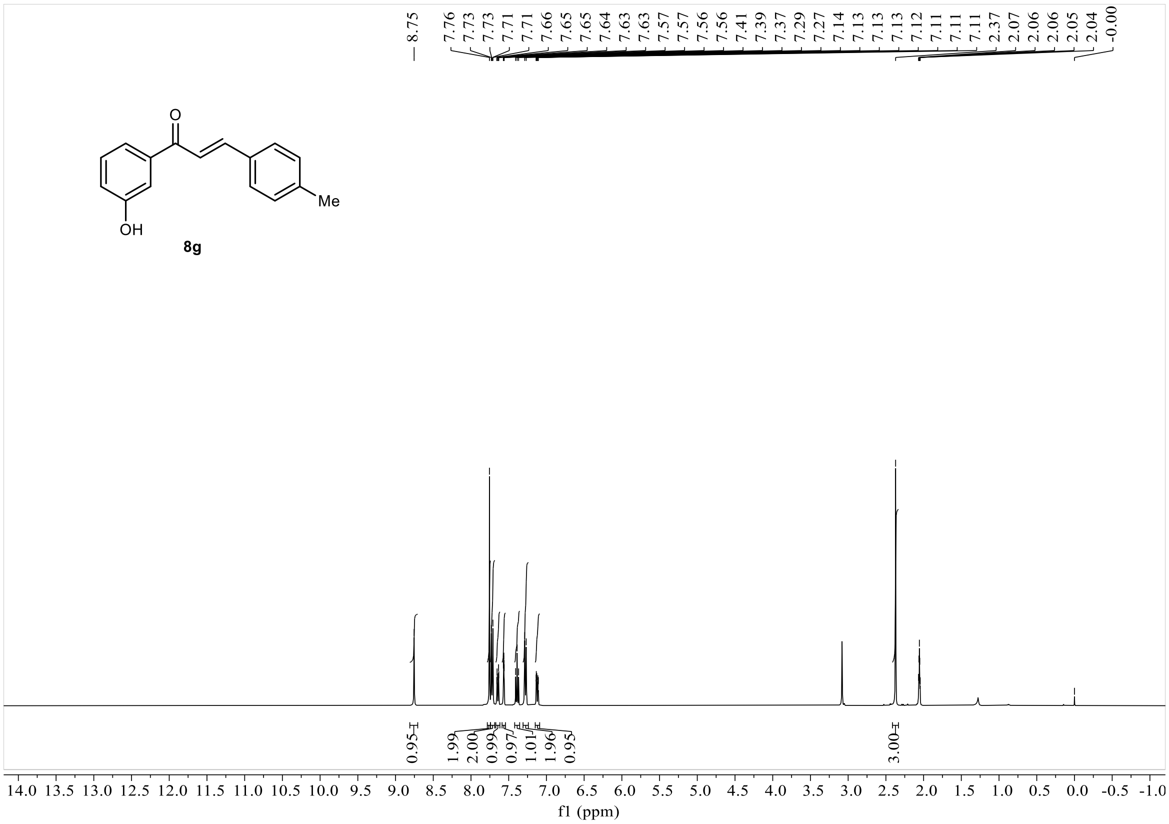

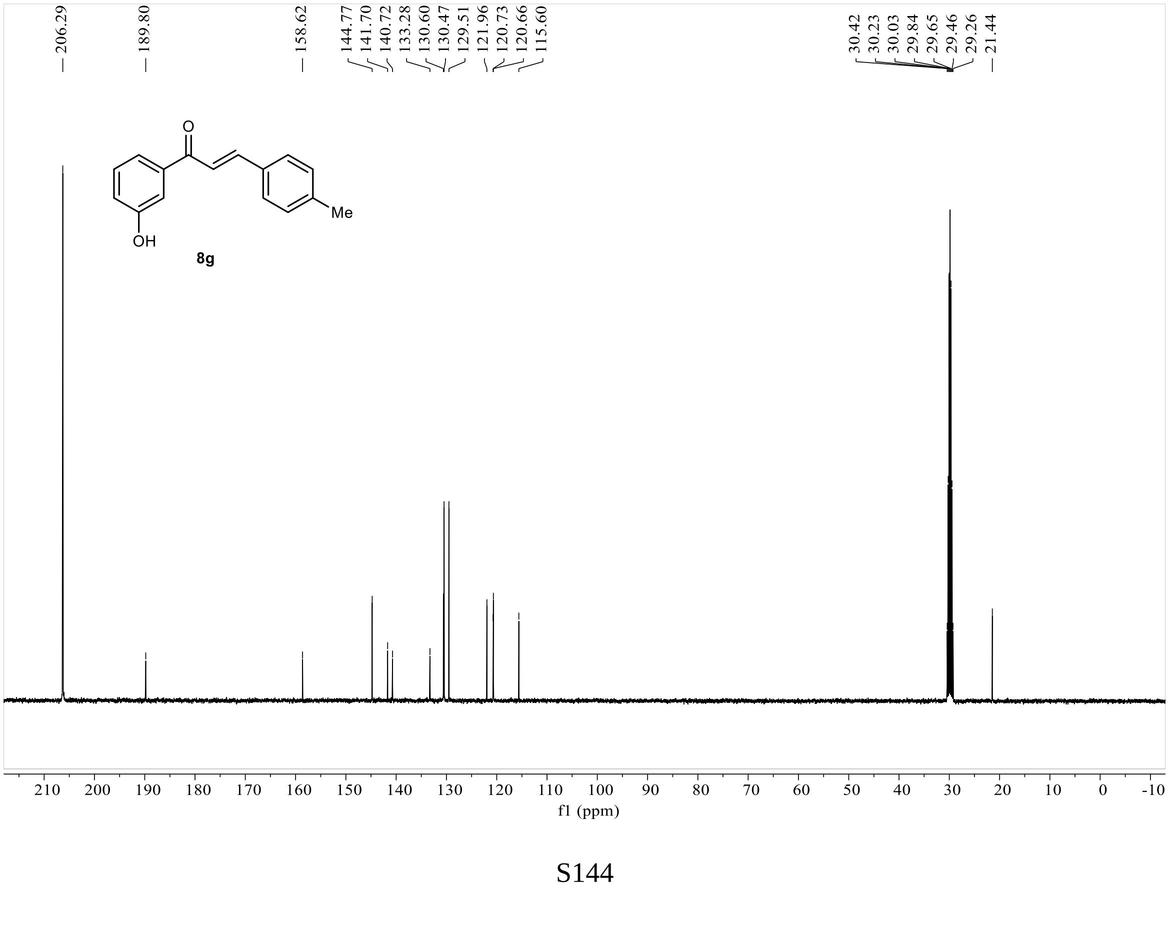

^{1}H NMR (400 MHz, Acetone-d_6) and ^{13}C NMR (101 MHz, Acetone-d_6) spectra of **8h**

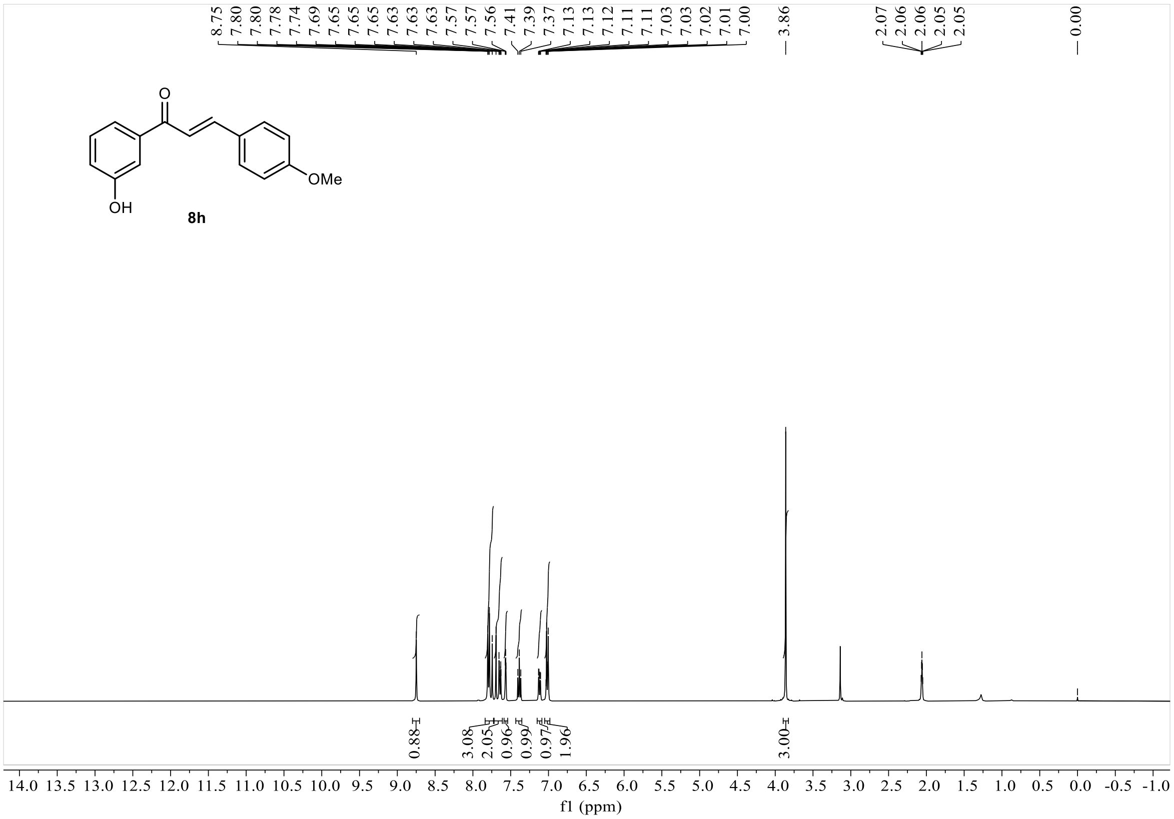

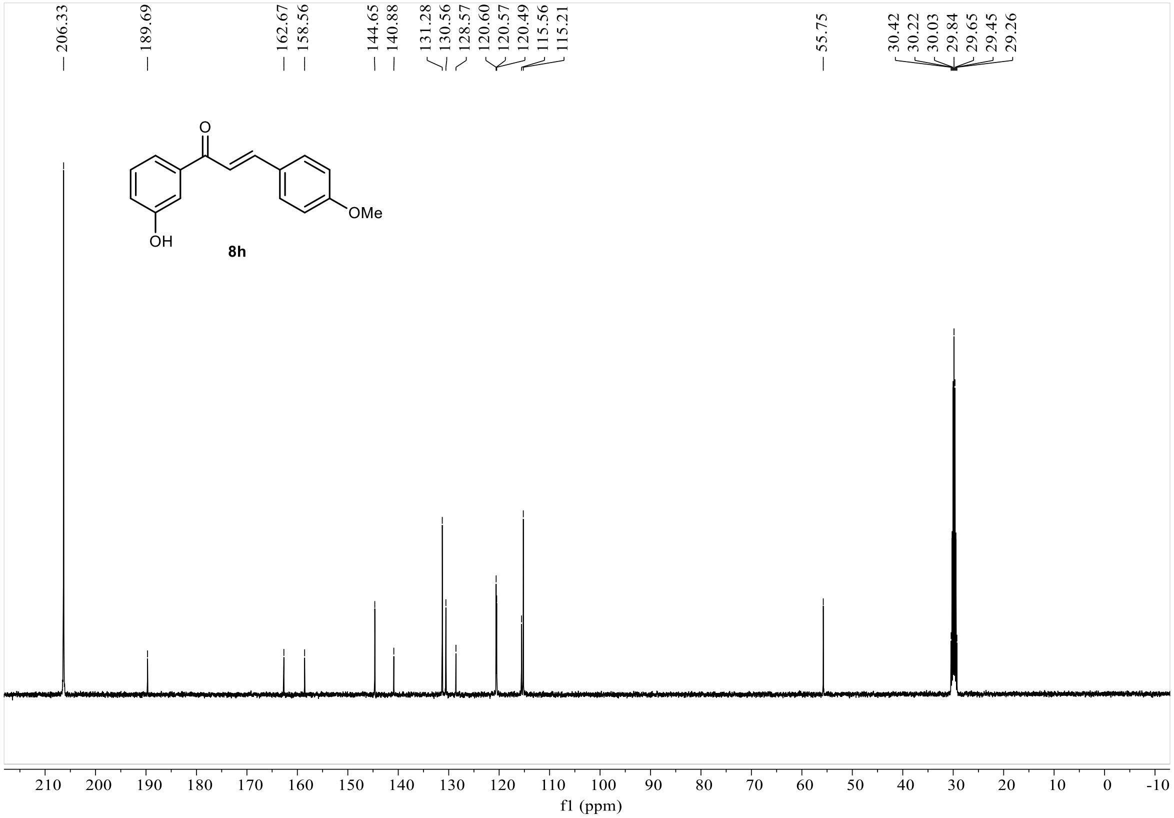

^{1}H NMR (400 MHz, Acetone-d_6) and ^{13}C NMR (101 MHz, Acetone-d_6) spectra of **8i**

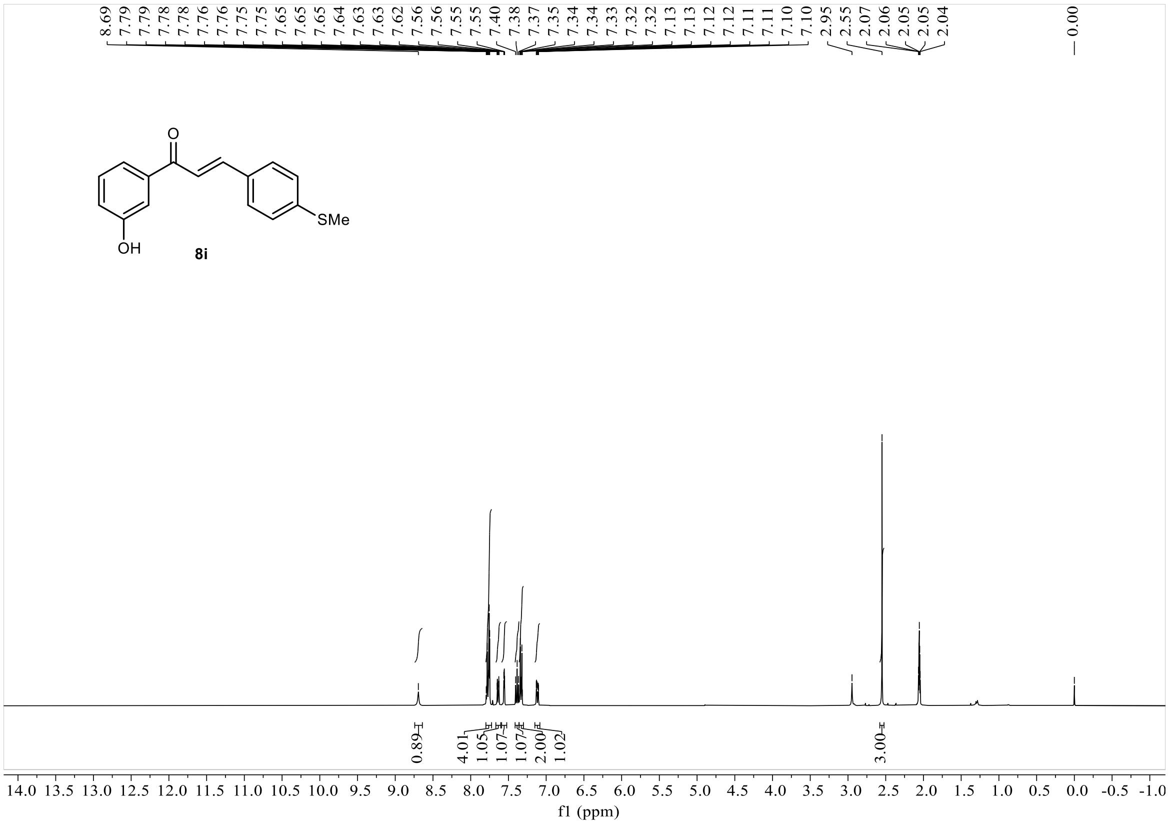

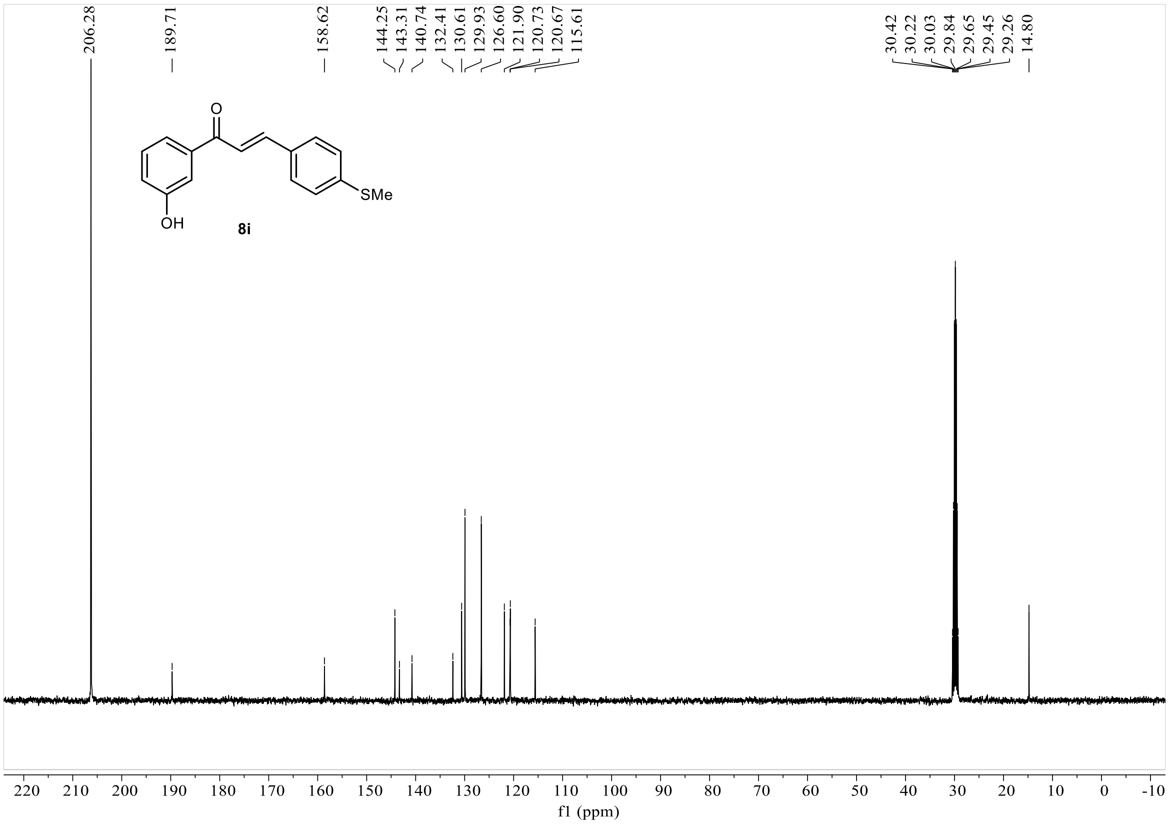

^{1}H NMR (400 MHz, Acetone-d_6) and ^{13}C NMR (101 MHz, Acetone-d_6) spectra of **8j**

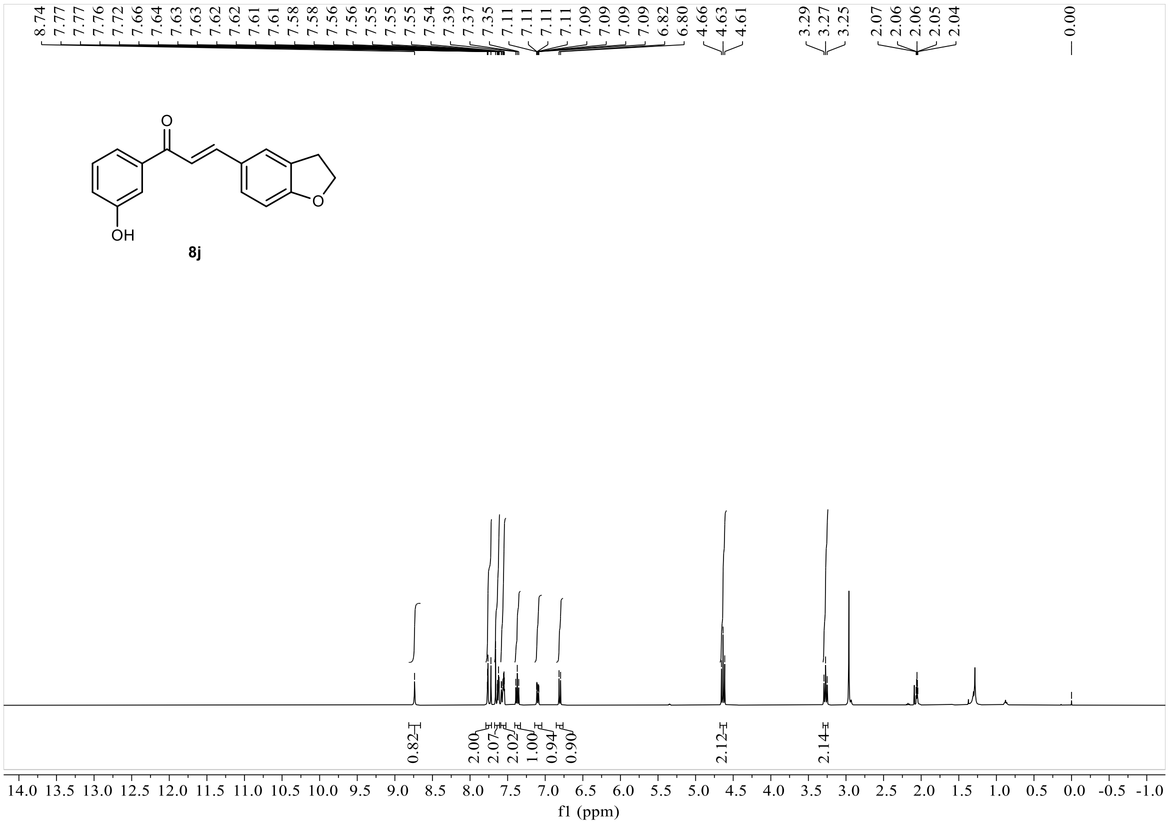

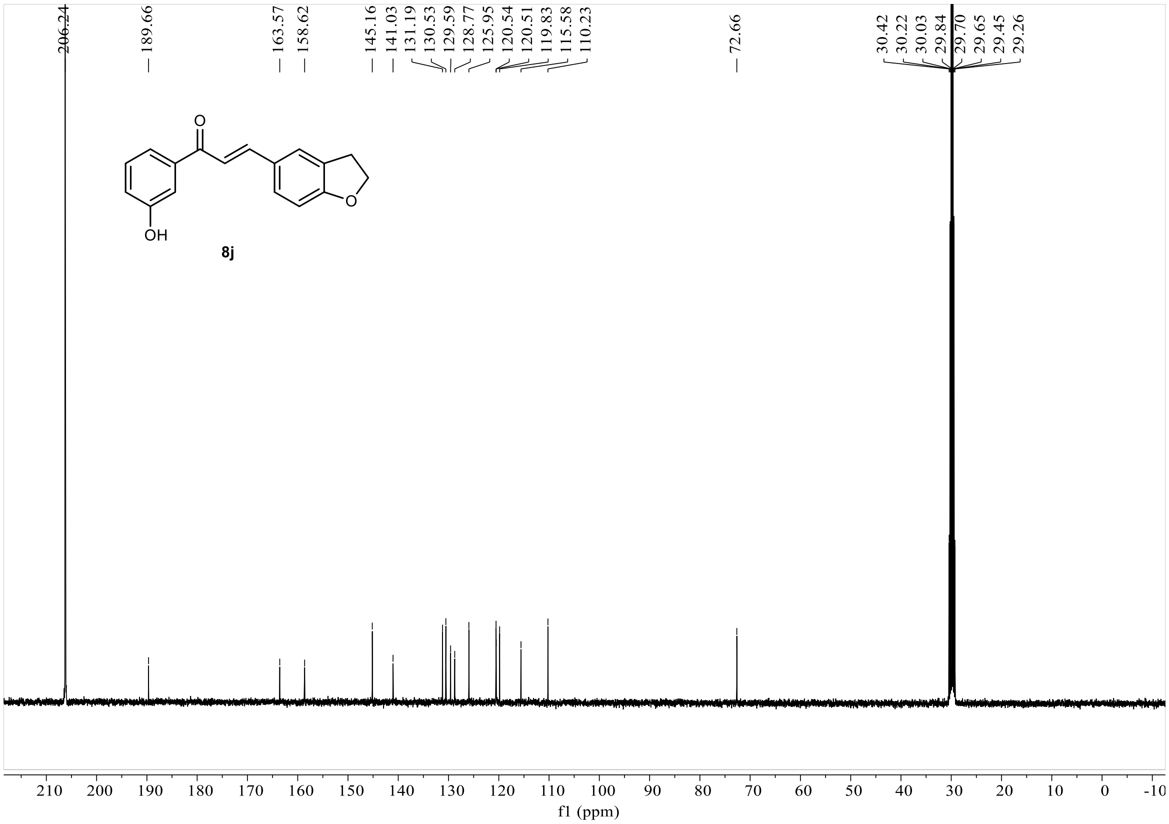

^{1}H NMR (400 MHz, Acetone-d_6) and ^{13}C NMR (101 MHz, Acetone-d_6) spectra of **8k**

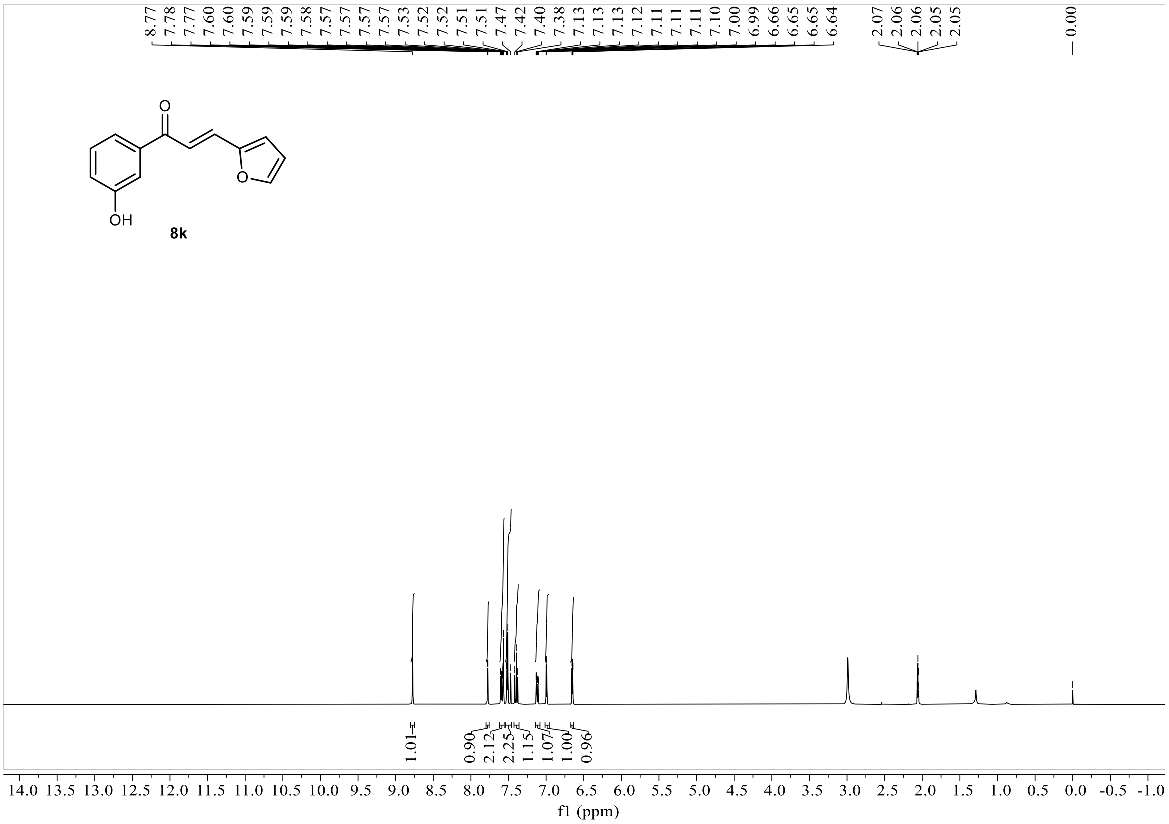

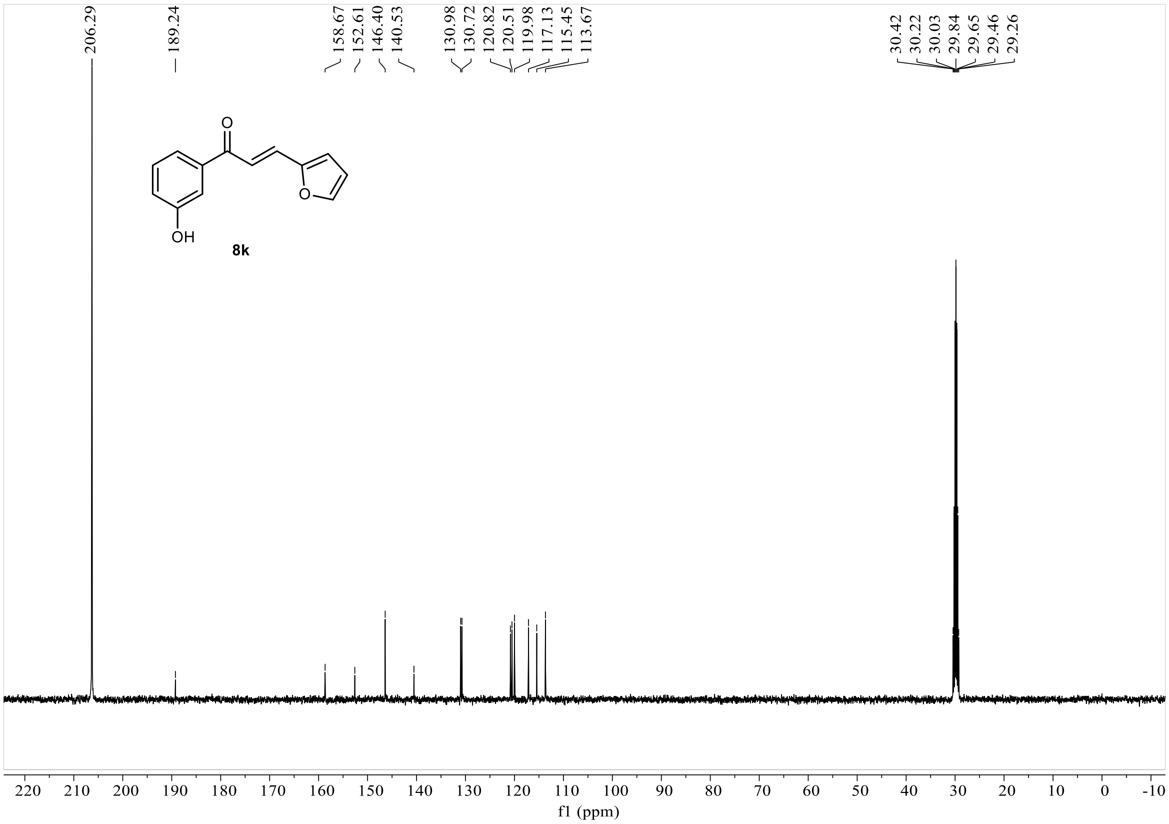

^{1}H NMR (400 MHz, Acetone-d_6) and ^{13}C NMR (101 MHz, Acetone-d_6) spectra of **8l**

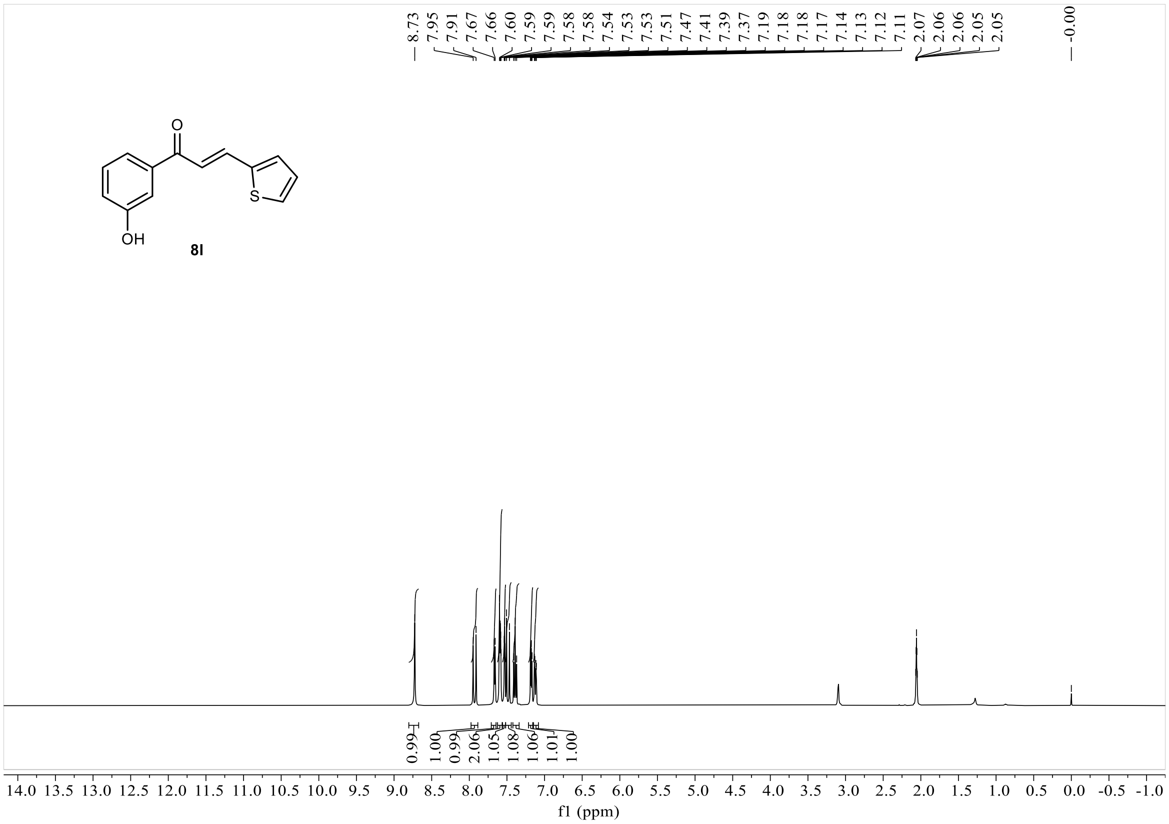

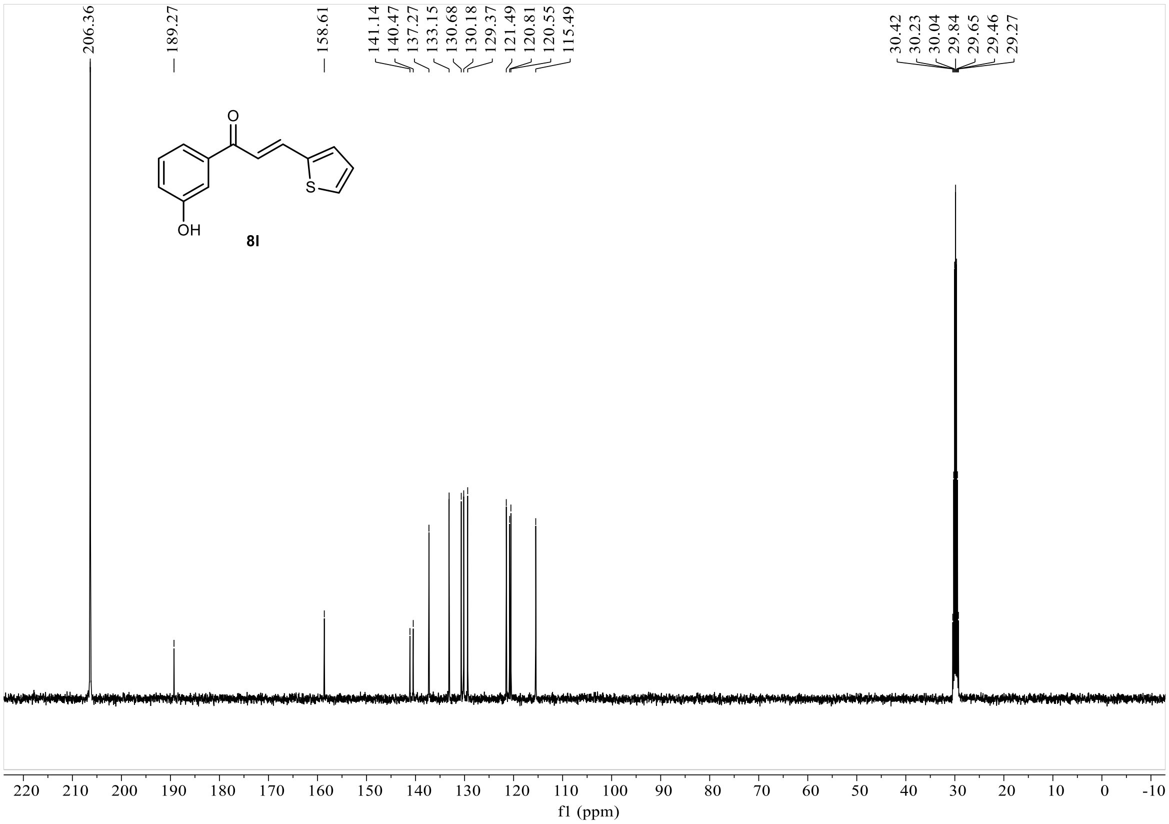

^{1}H NMR (400 MHz, Acetone-d_6) and ^{13}C NMR (101 MHz, Acetone-d_6) spectra of **8m**

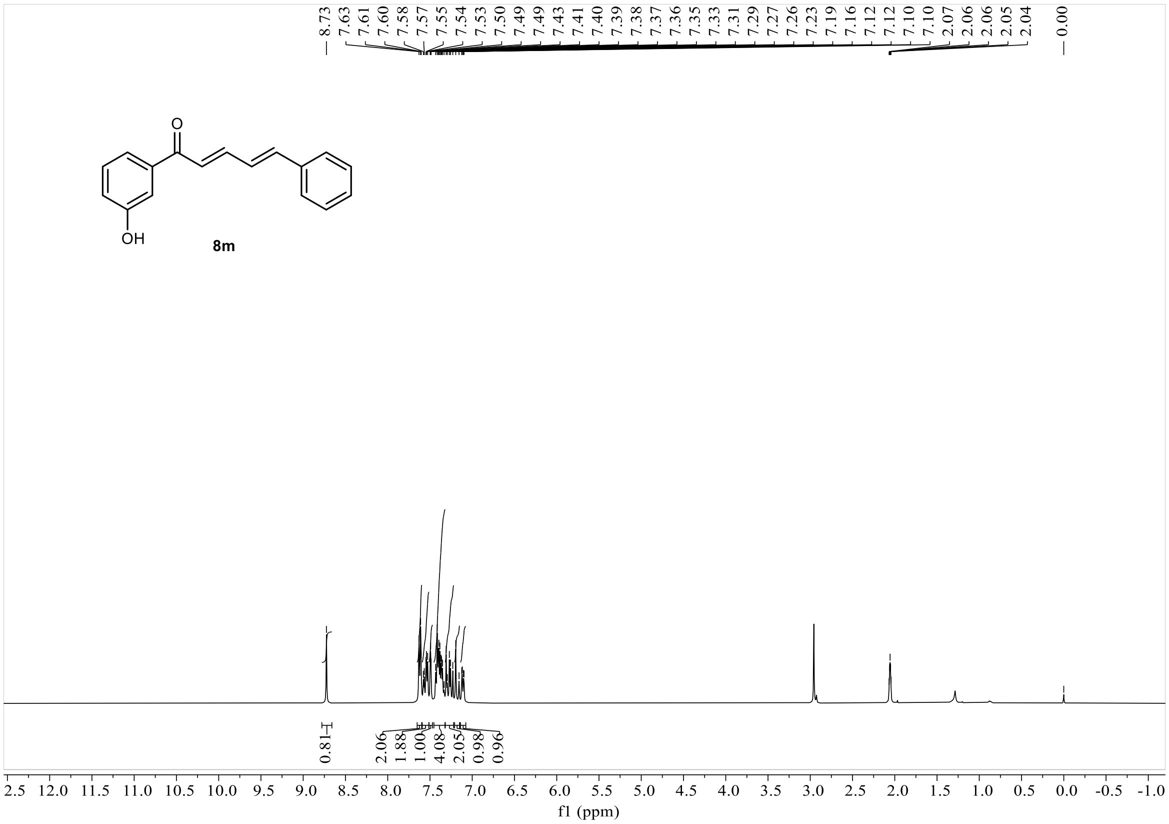

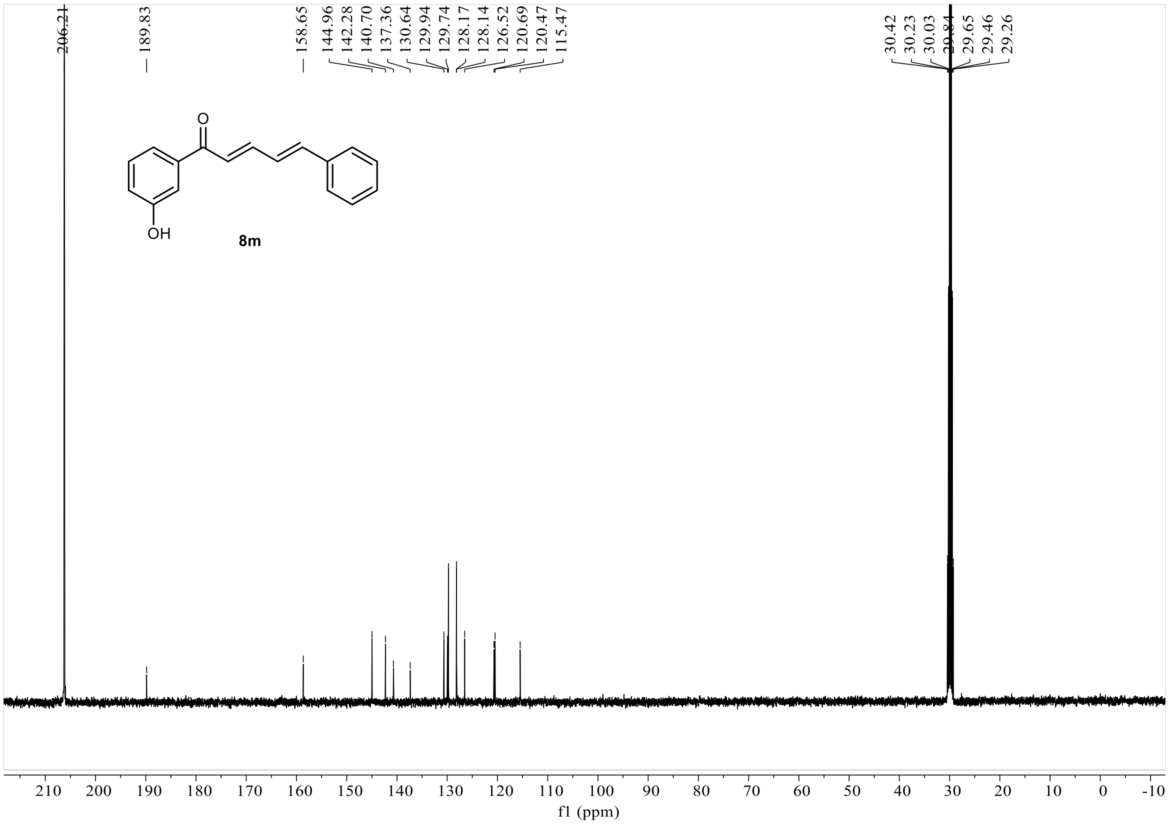

^{1}H NMR (400 MHz, Acetone-d_6) and ^{13}C NMR (101 MHz, Acetone-d_6) spectra of **8n**

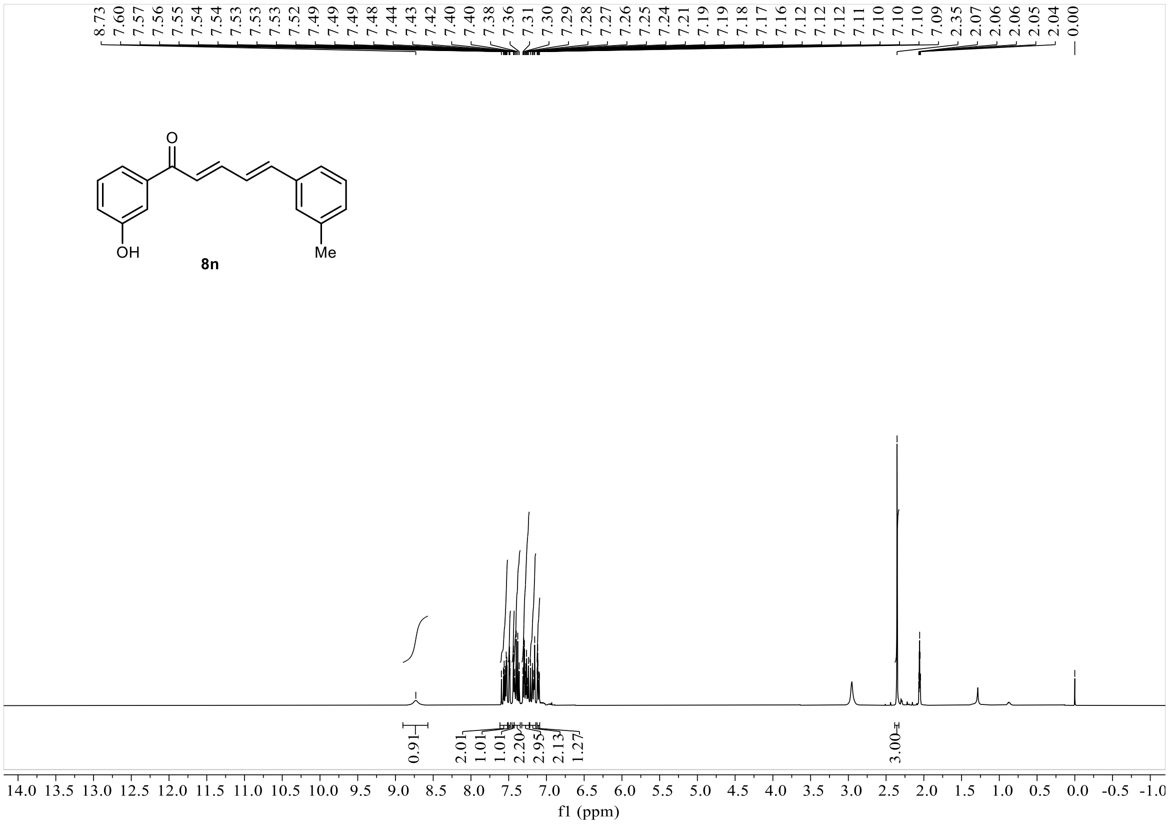

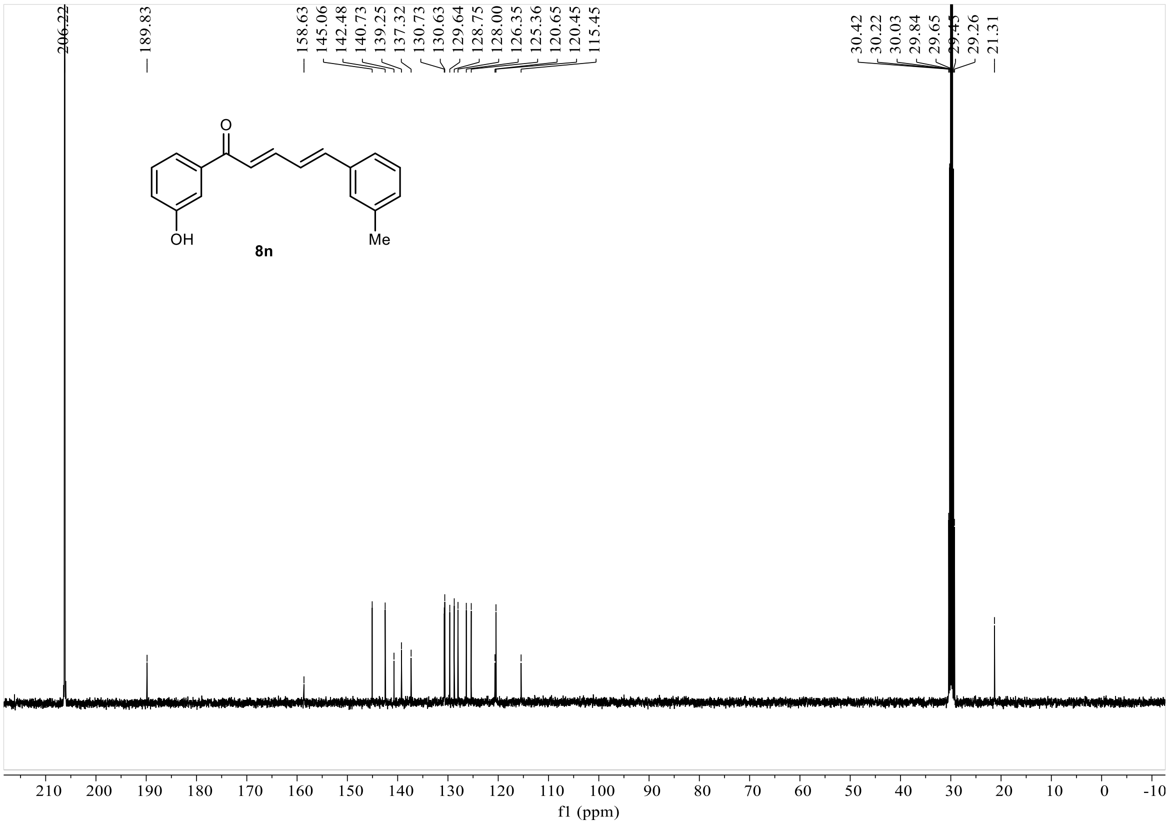

^{1}H NMR (400 MHz, Acetone-d_6) and ^{13}C NMR (101 MHz, Acetone-d_6) spectra of **8o**

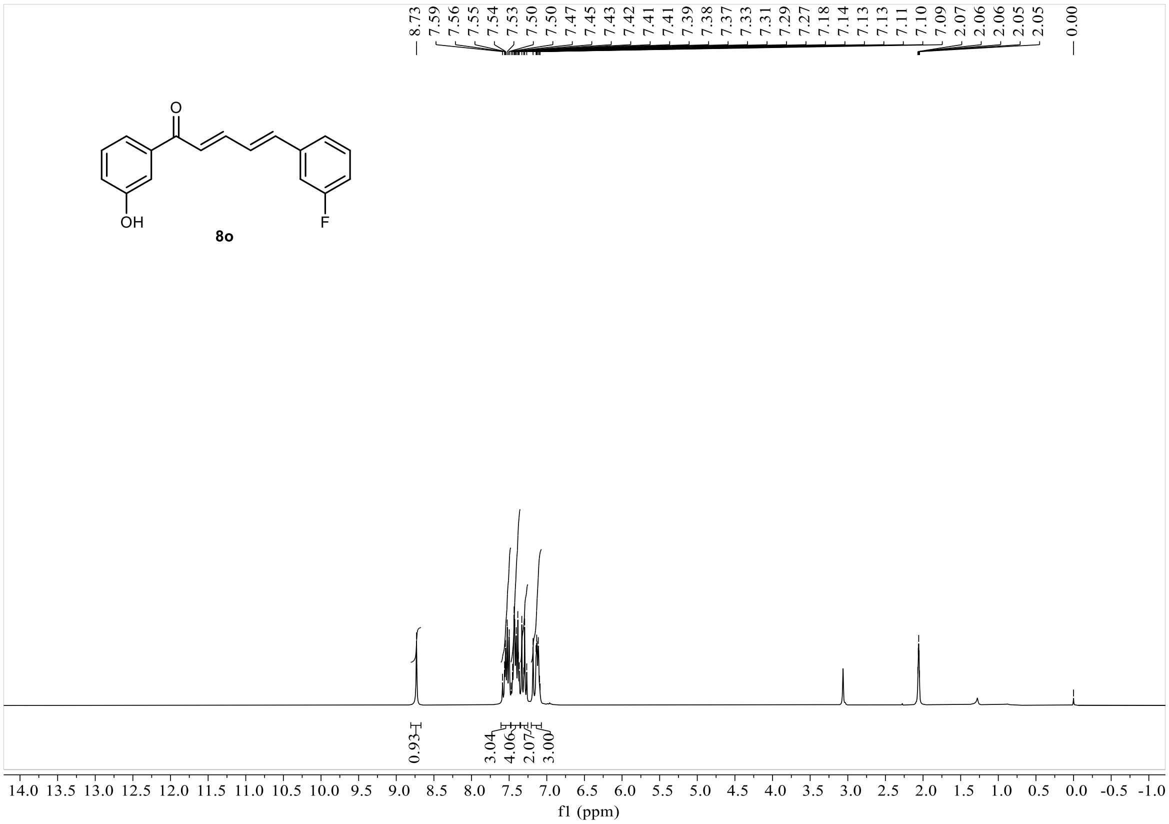

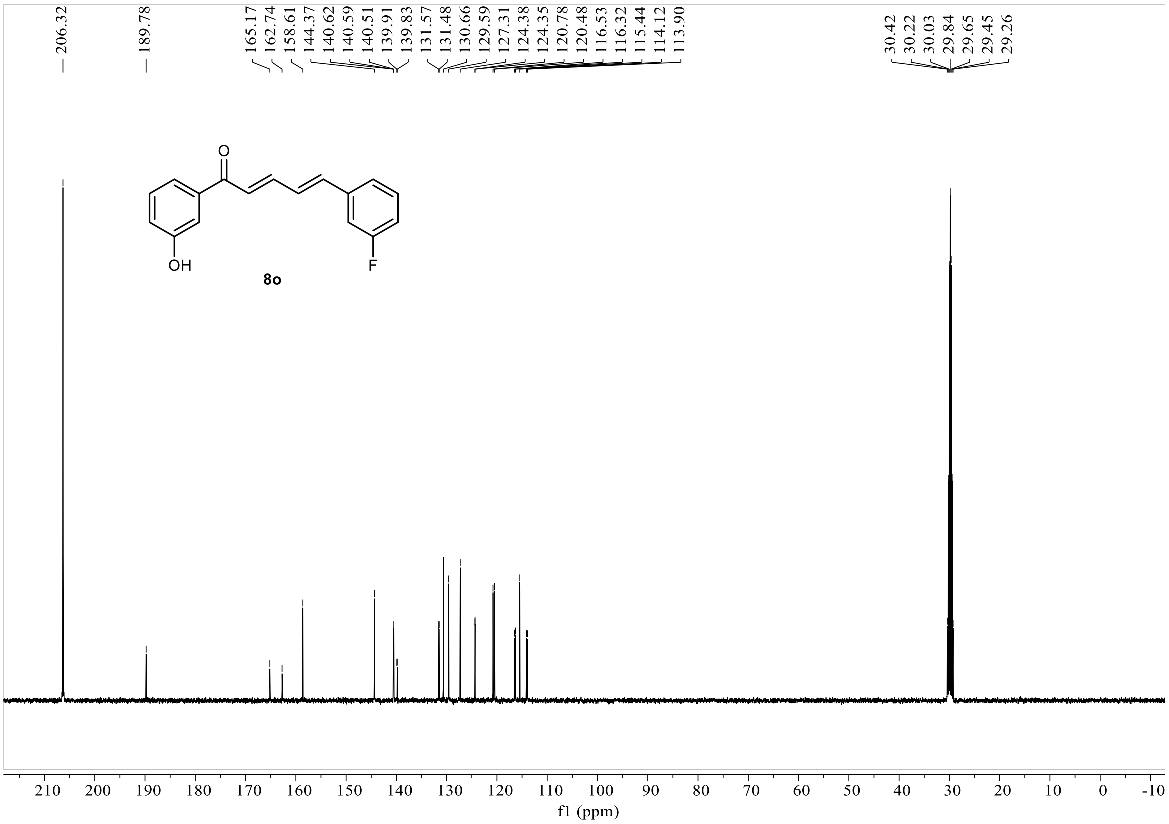

^{19}F NMR (376 MHz, Acetone-d_6) spectrum of **8o**

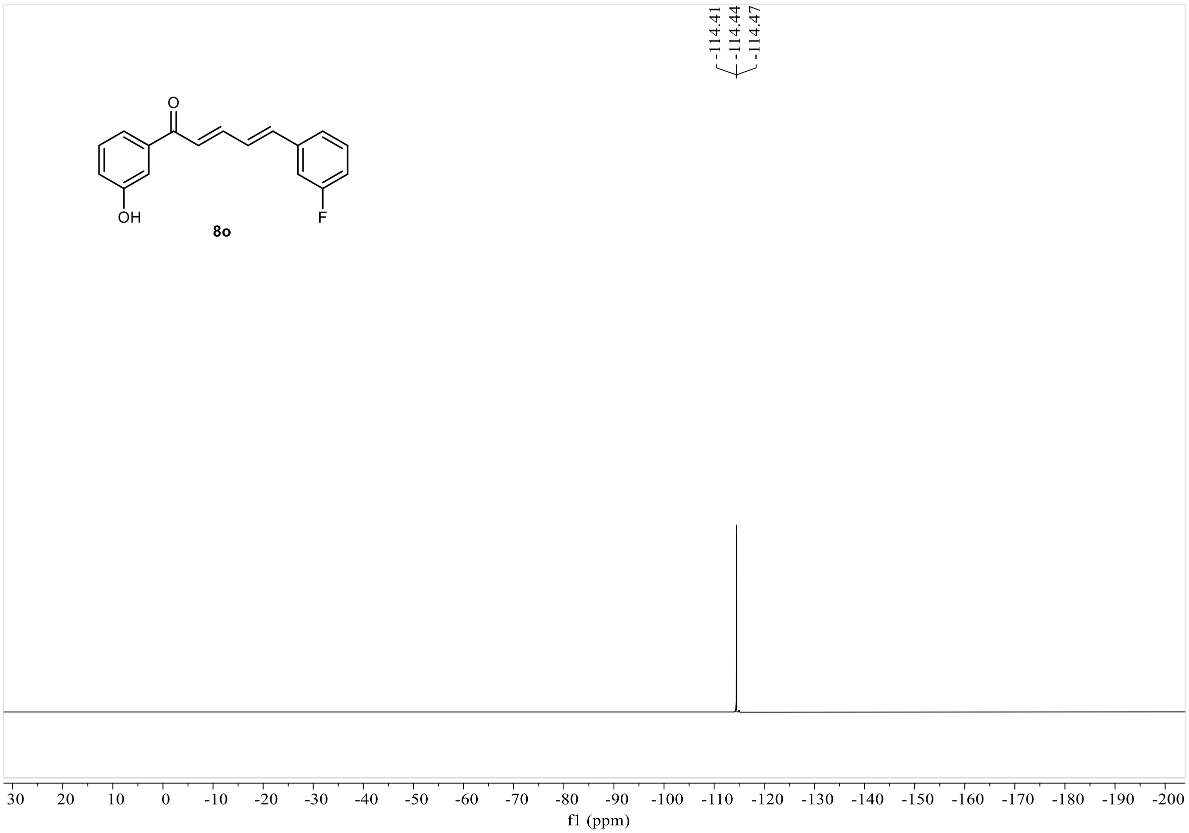

^{1}H NMR (400 MHz, Acetone-d_6) and ^{13}C NMR (101 MHz, Acetone-d_6) spectra of **8p**

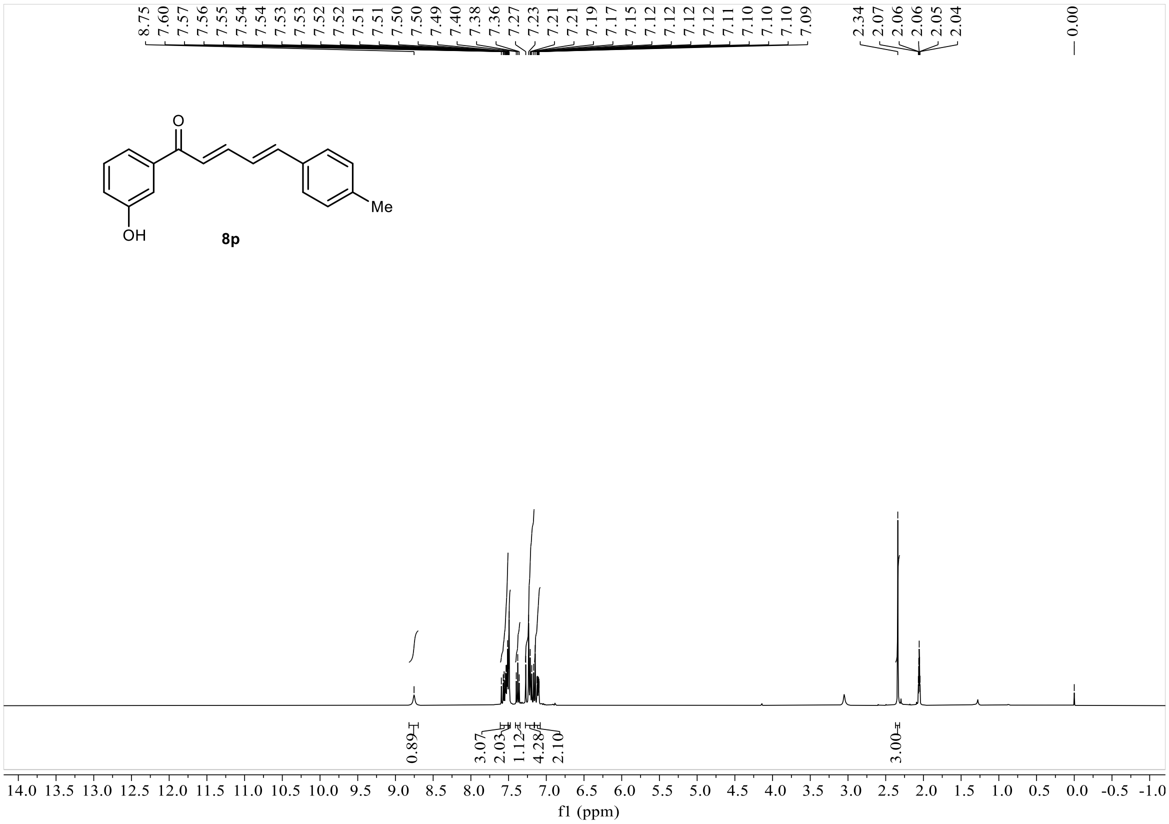

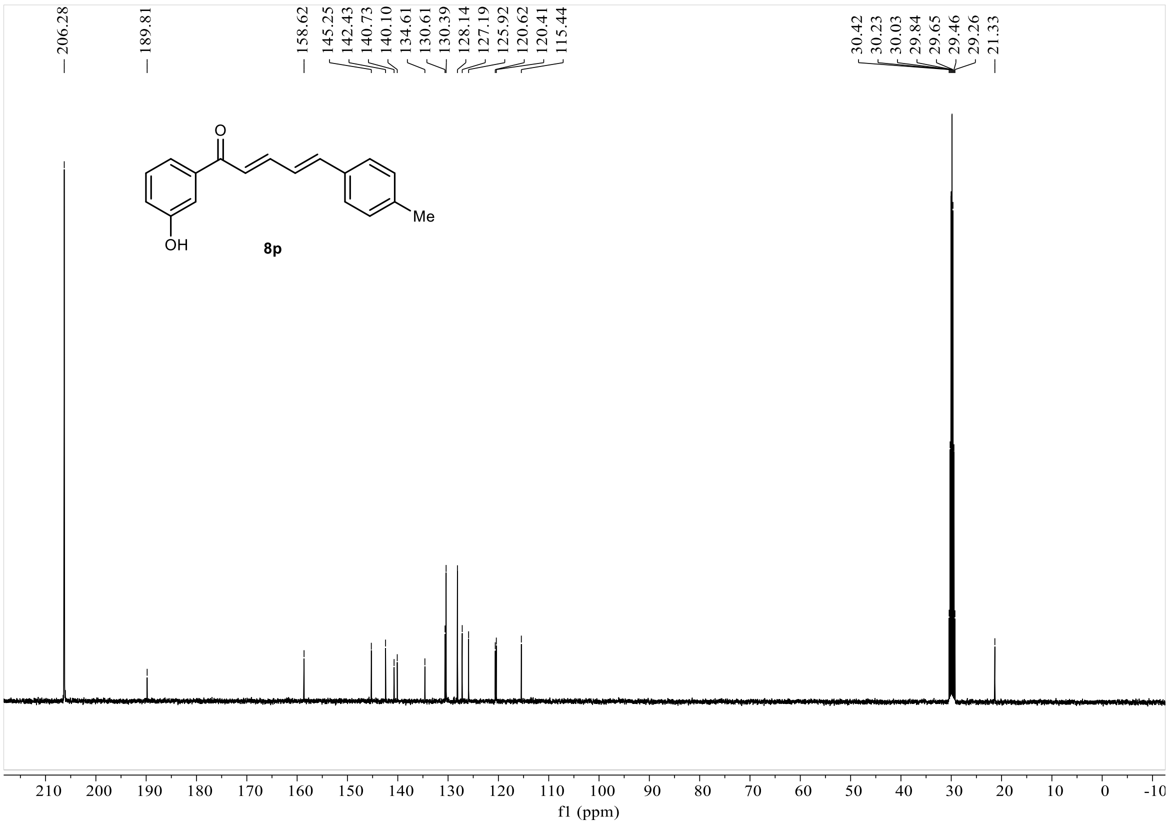

^{1}H NMR (400 MHz, Acetone-d_6) and ^{13}C NMR (101 MHz, Acetone-d_6) spectra of **8q**

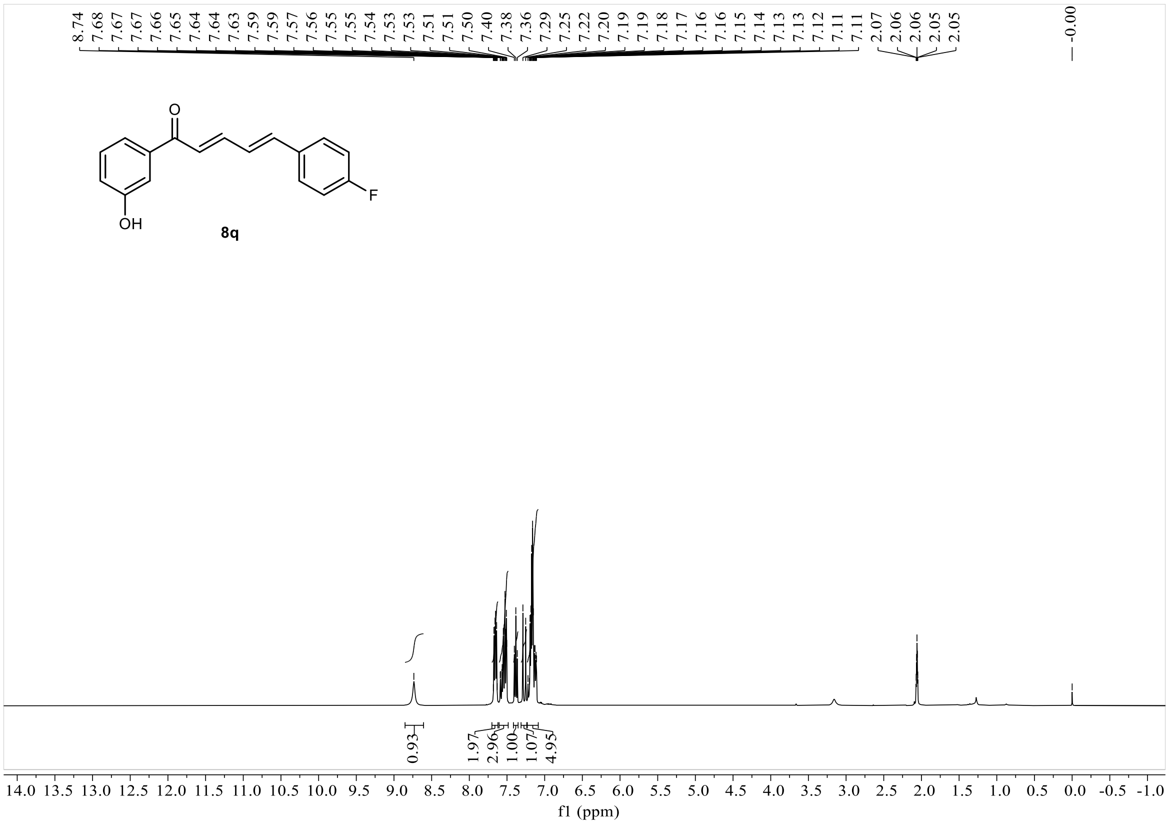

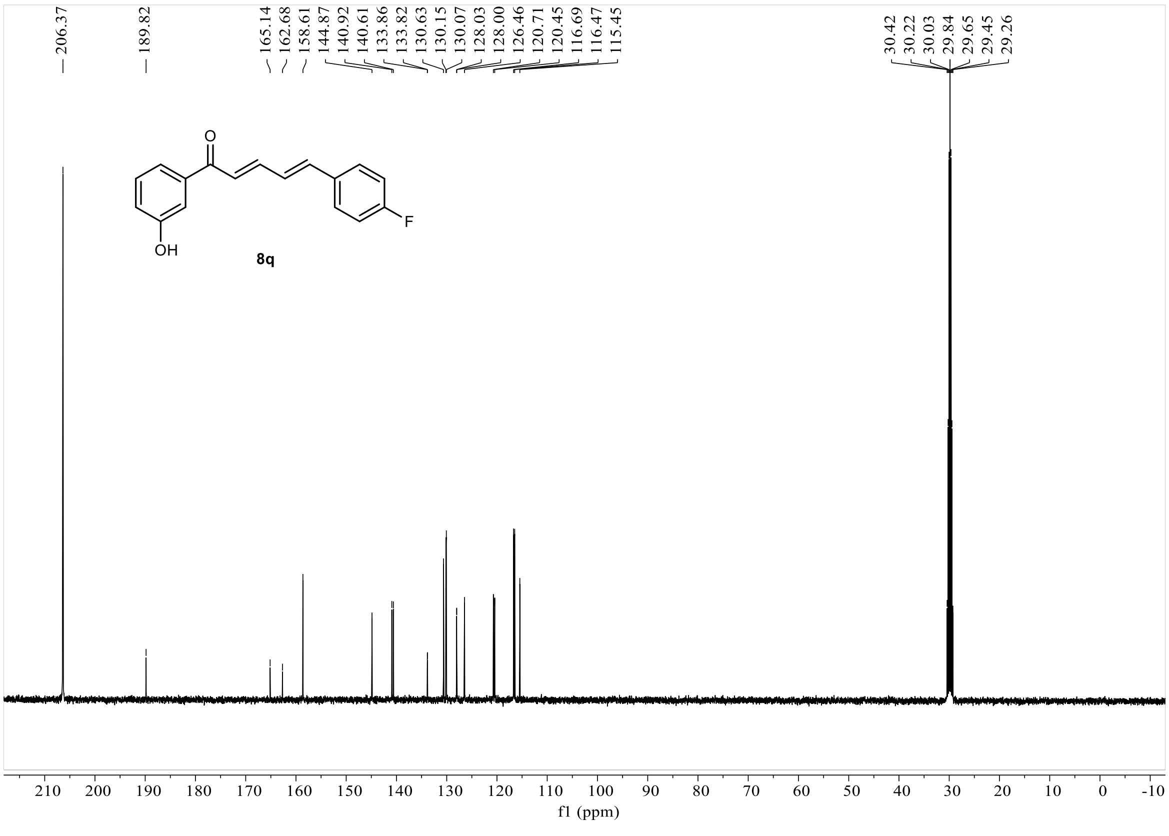

^{19}F NMR (376 MHz, Acetone-d_6) spectrum of **8q**

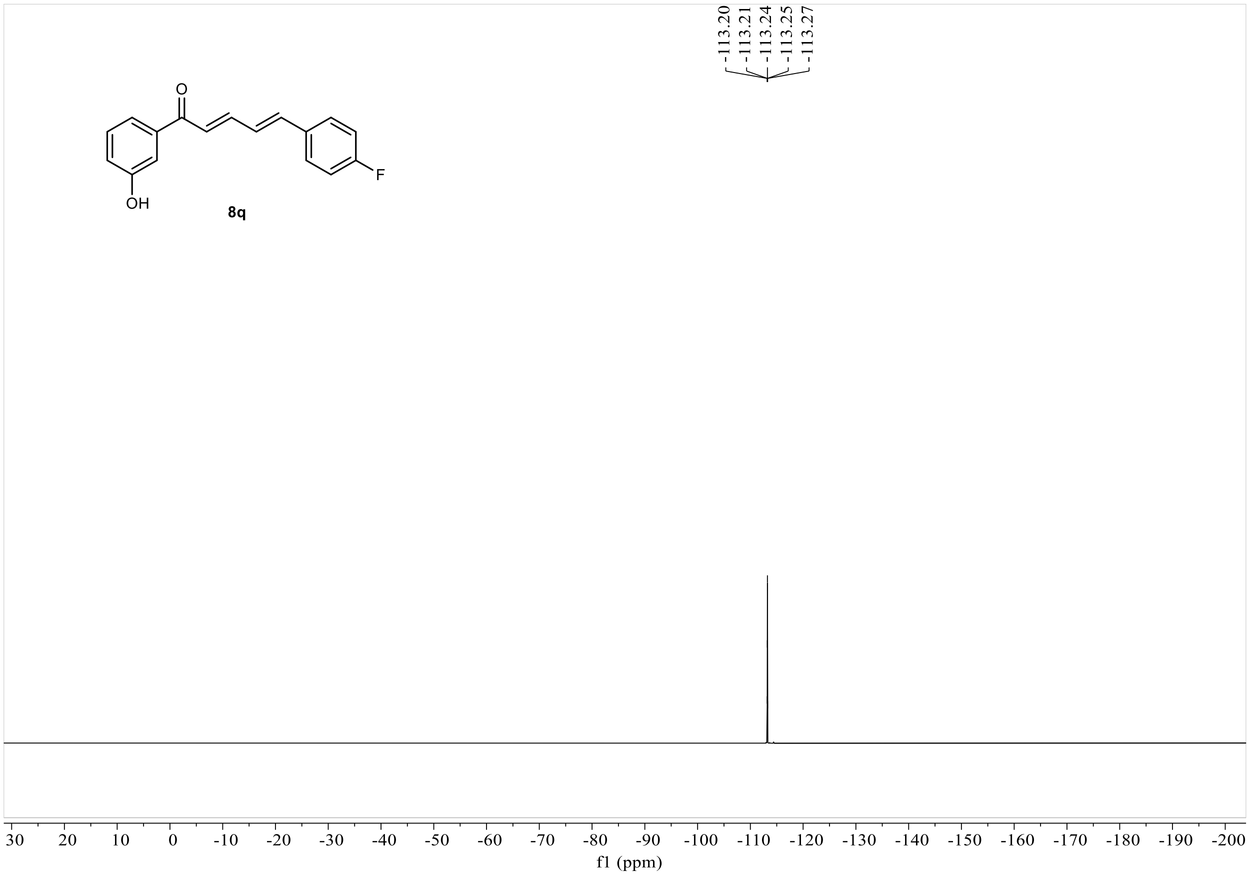

^{1}H NMR (400 MHz, Acetone-d_6) and ^{13}C NMR (101 MHz, Acetone-d_6) spectra of **8r**

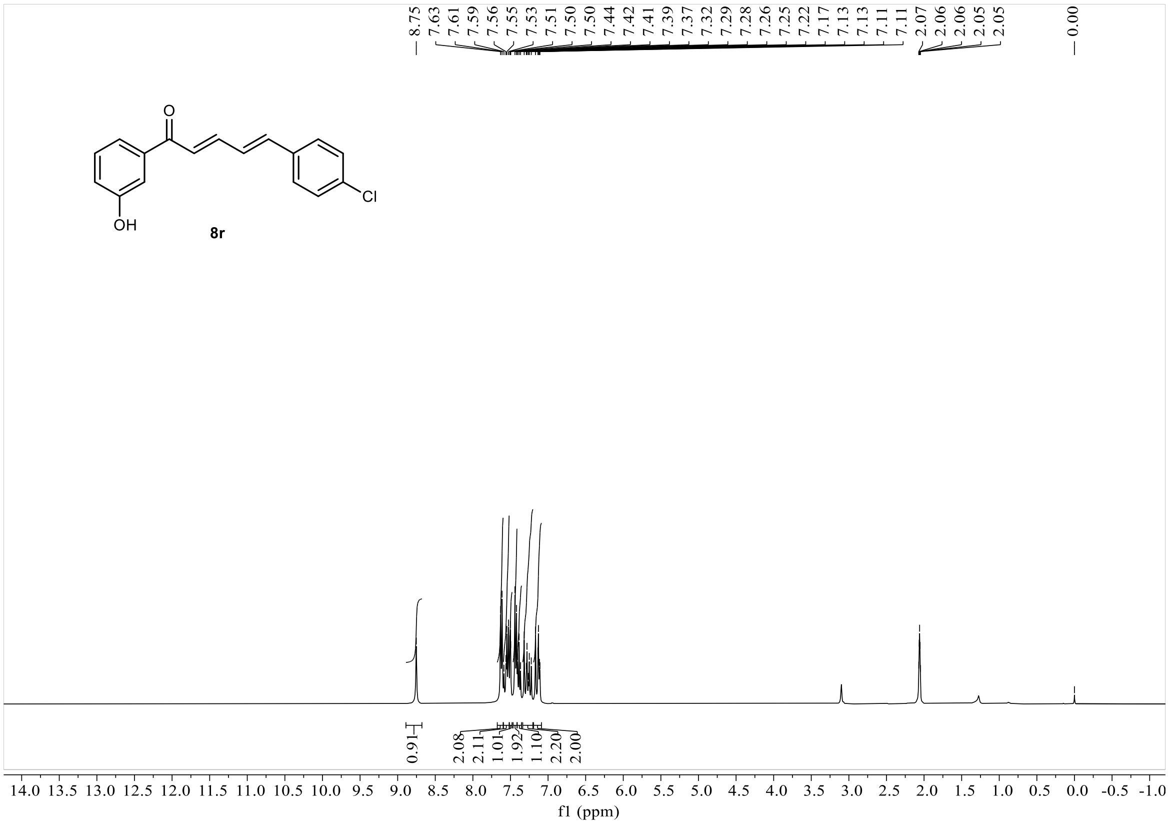

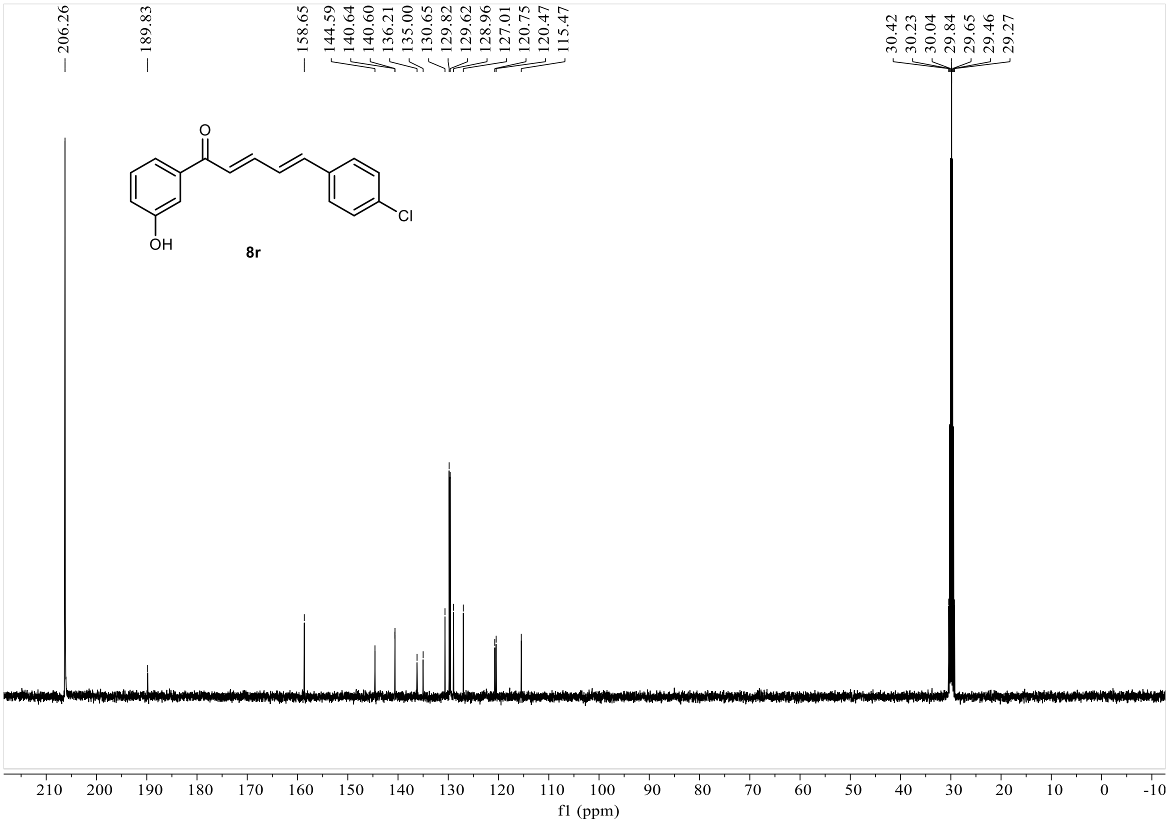

^{1}H NMR (400 MHz, $CDCl_3$) and ^{13}C NMR (101 MHz, $CDCl_3$) spectra of **10a**

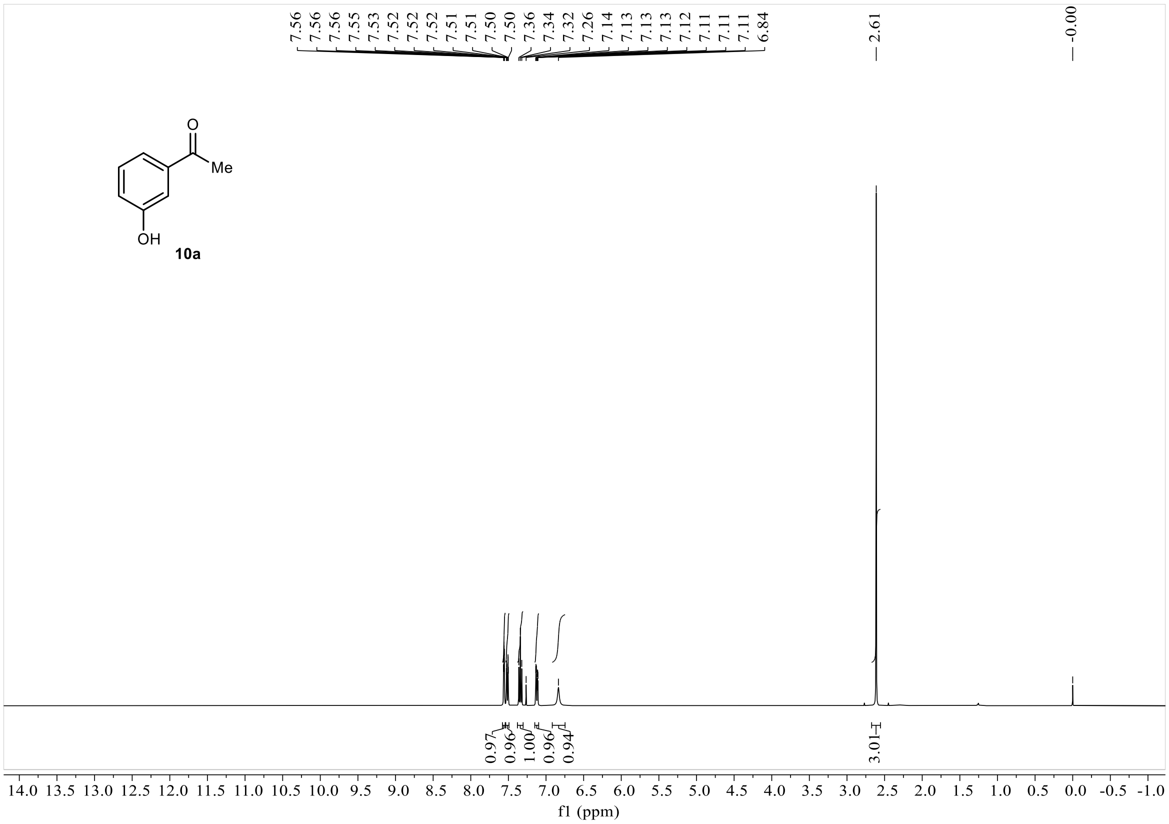

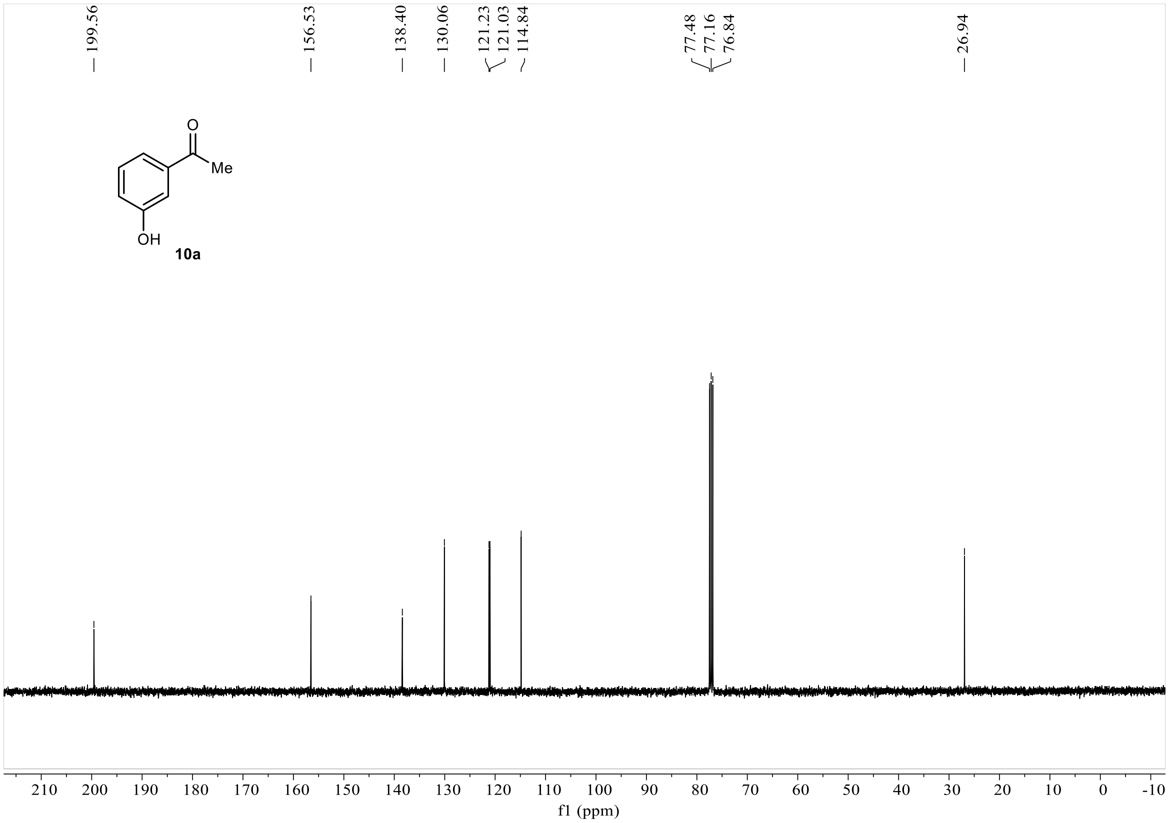

^{1}H NMR (400 MHz, $CDCl_3$) and ^{13}C NMR (101 MHz, $CDCl_3$) spectra of **10b**

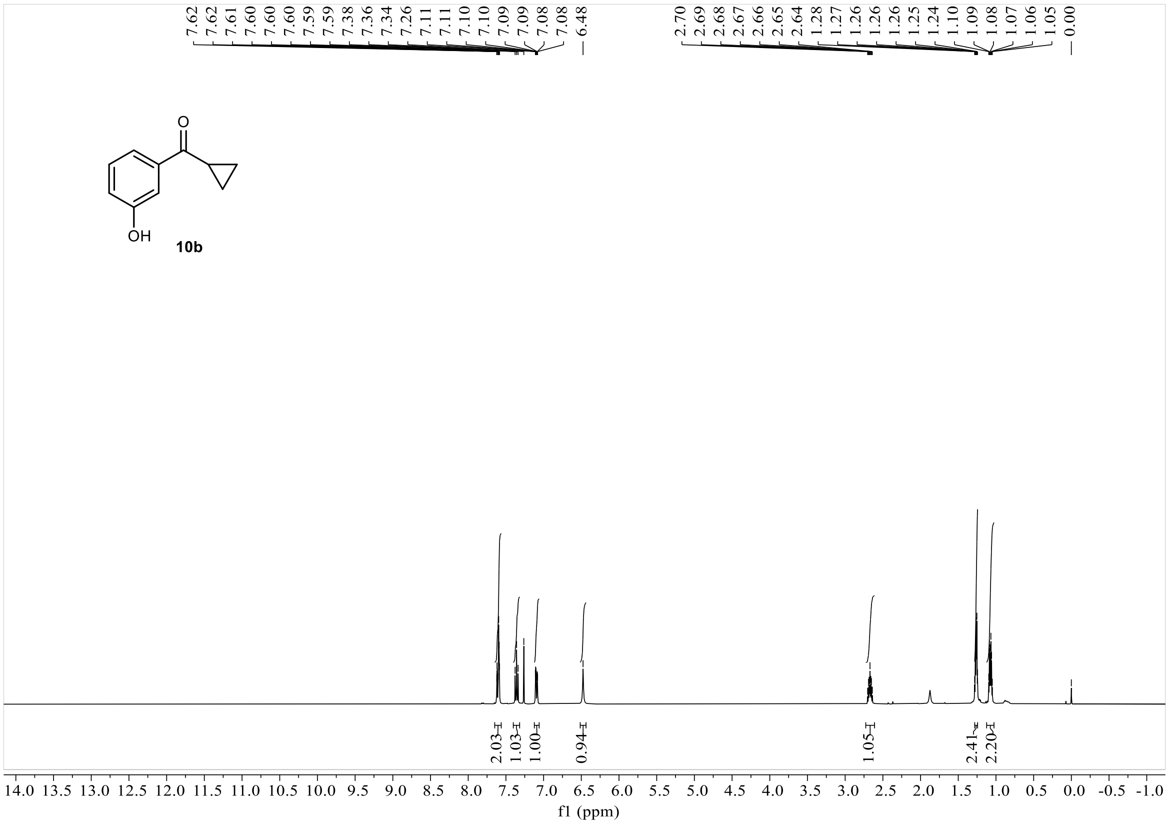

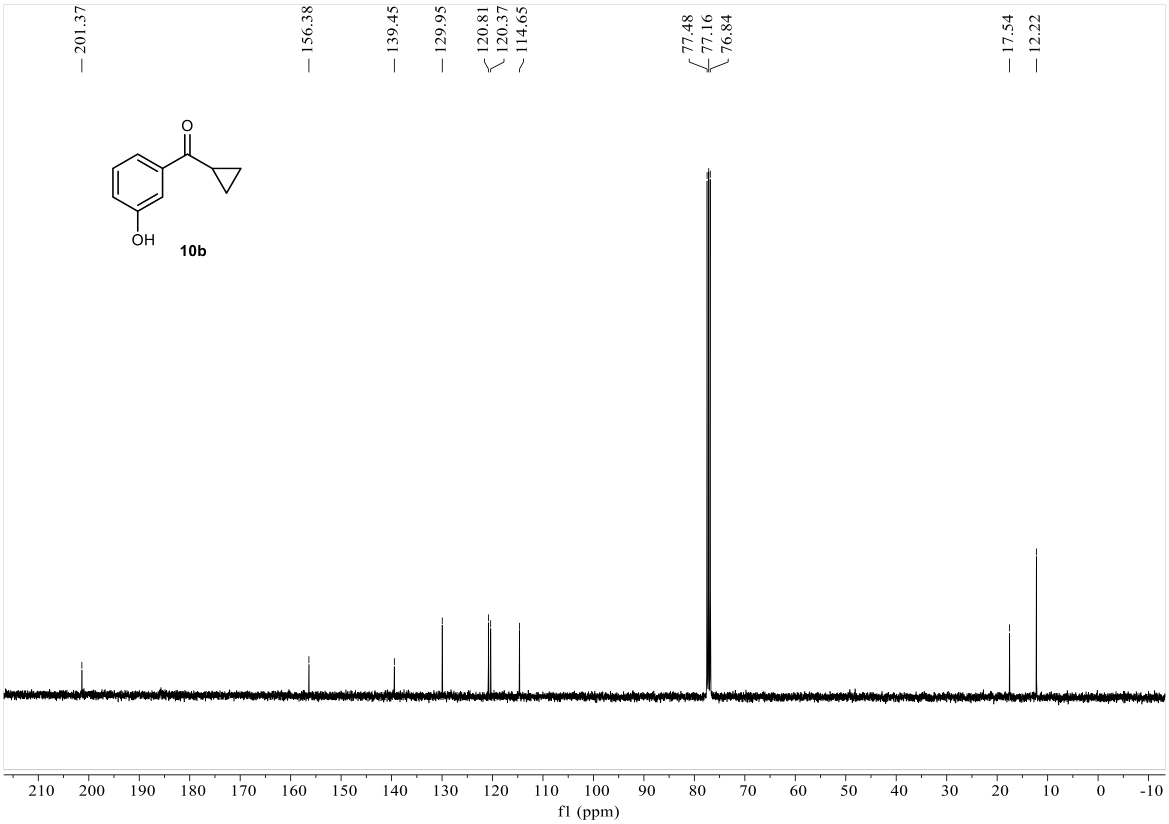

^{1}H NMR (400 MHz, $CDCl_3$) and ^{13}C NMR (101 MHz, $CDCl_3$) spectra of **10c**

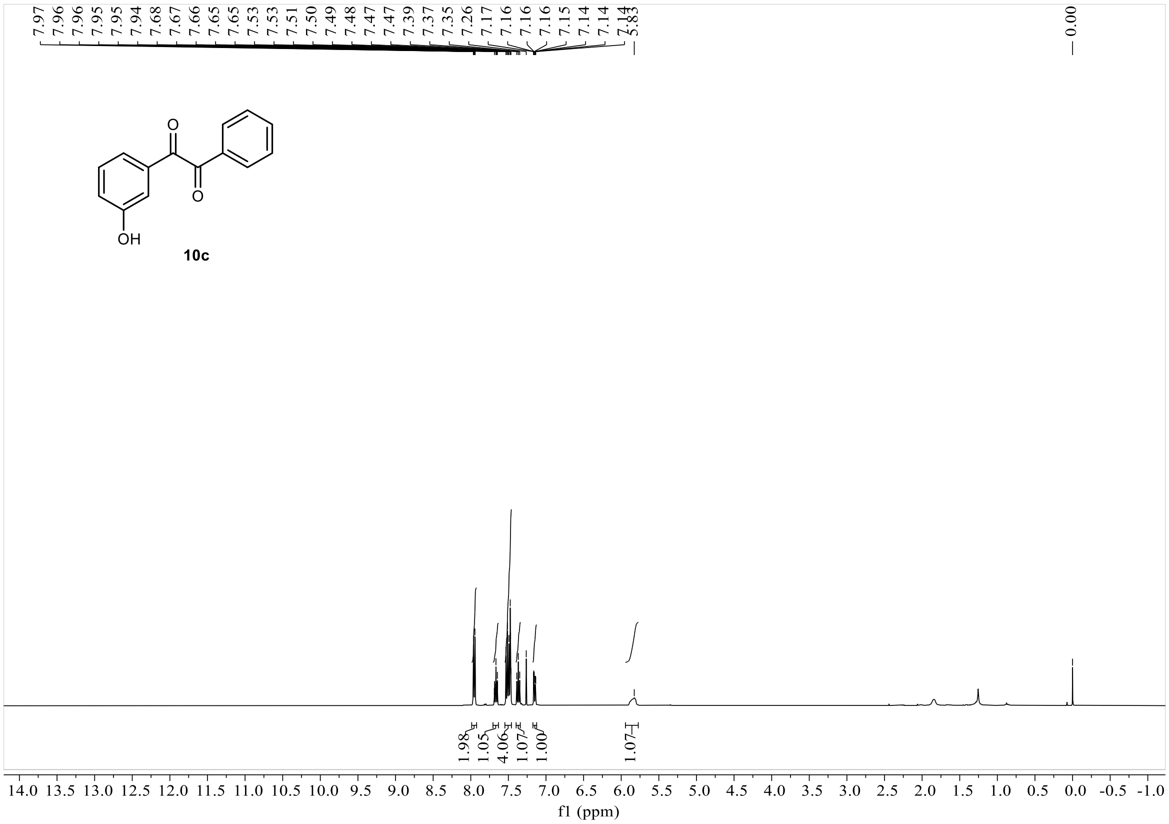

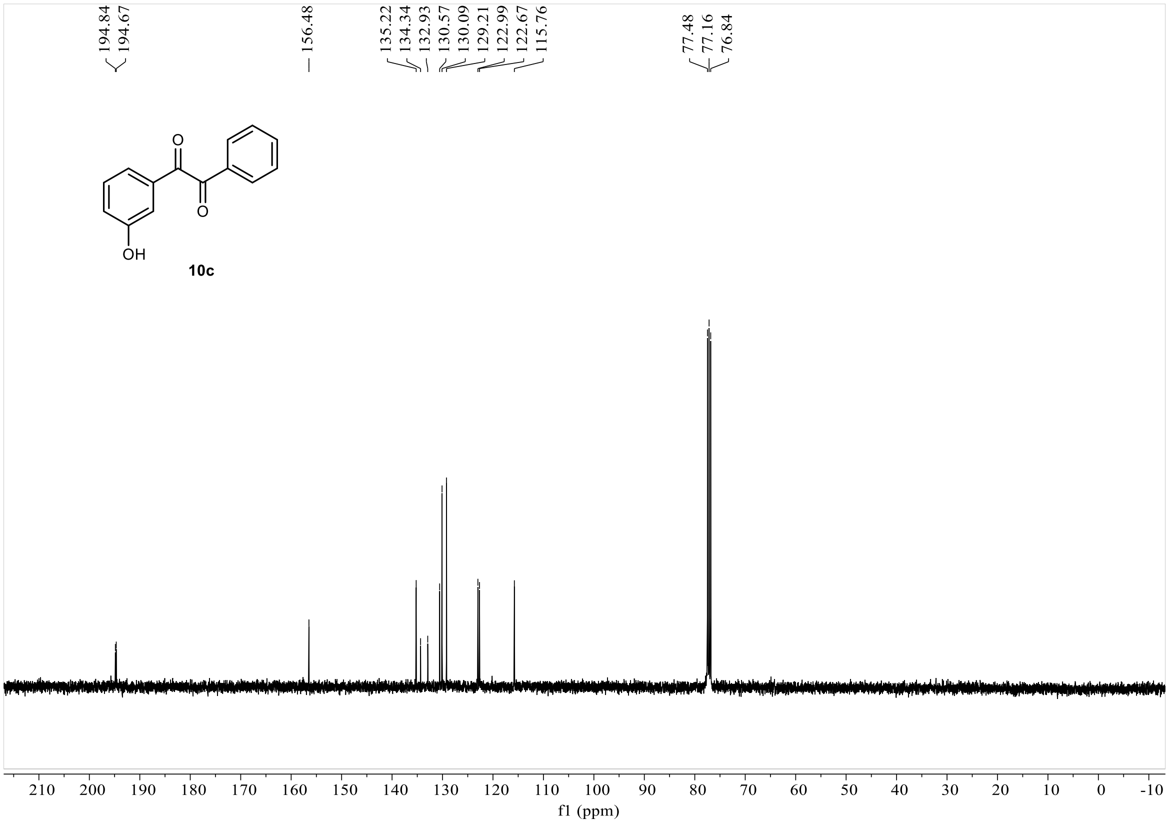

^{1}H NMR (400 MHz, $CDCl_3$) and ^{13}C NMR (101 MHz, $CDCl_3$) spectra of **10d**

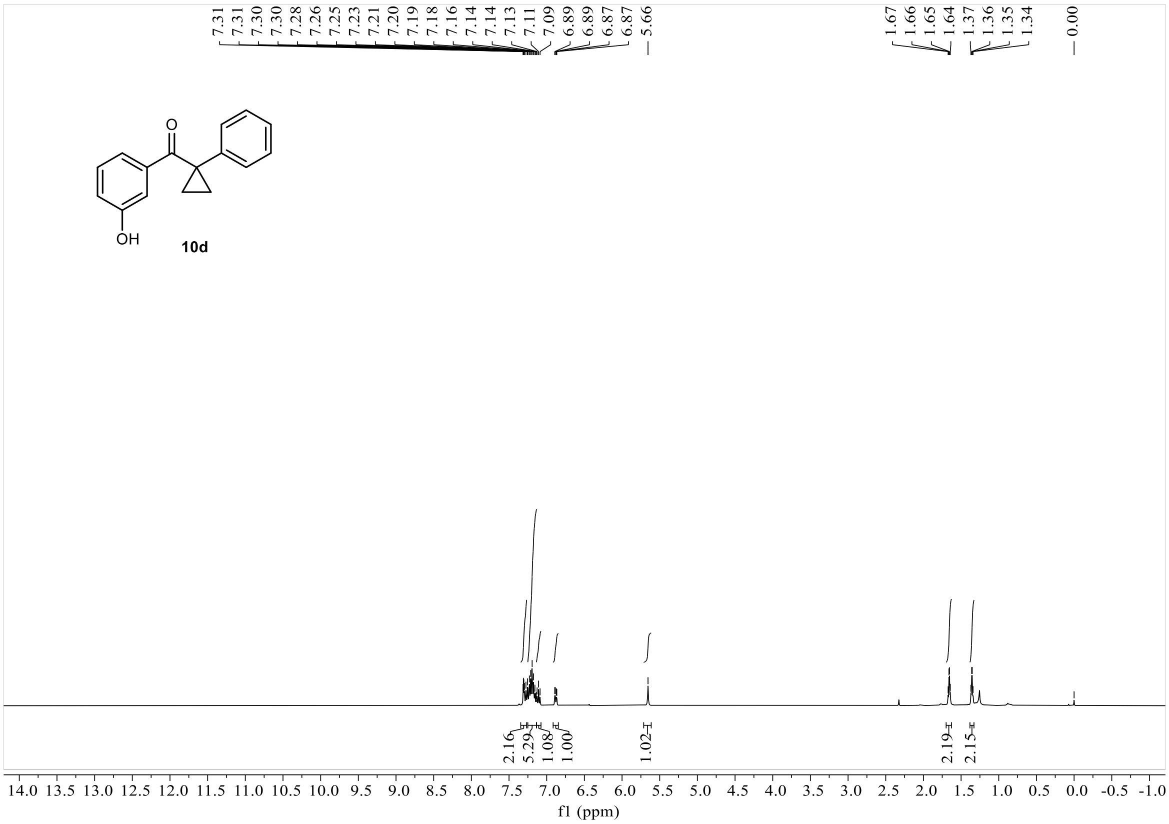

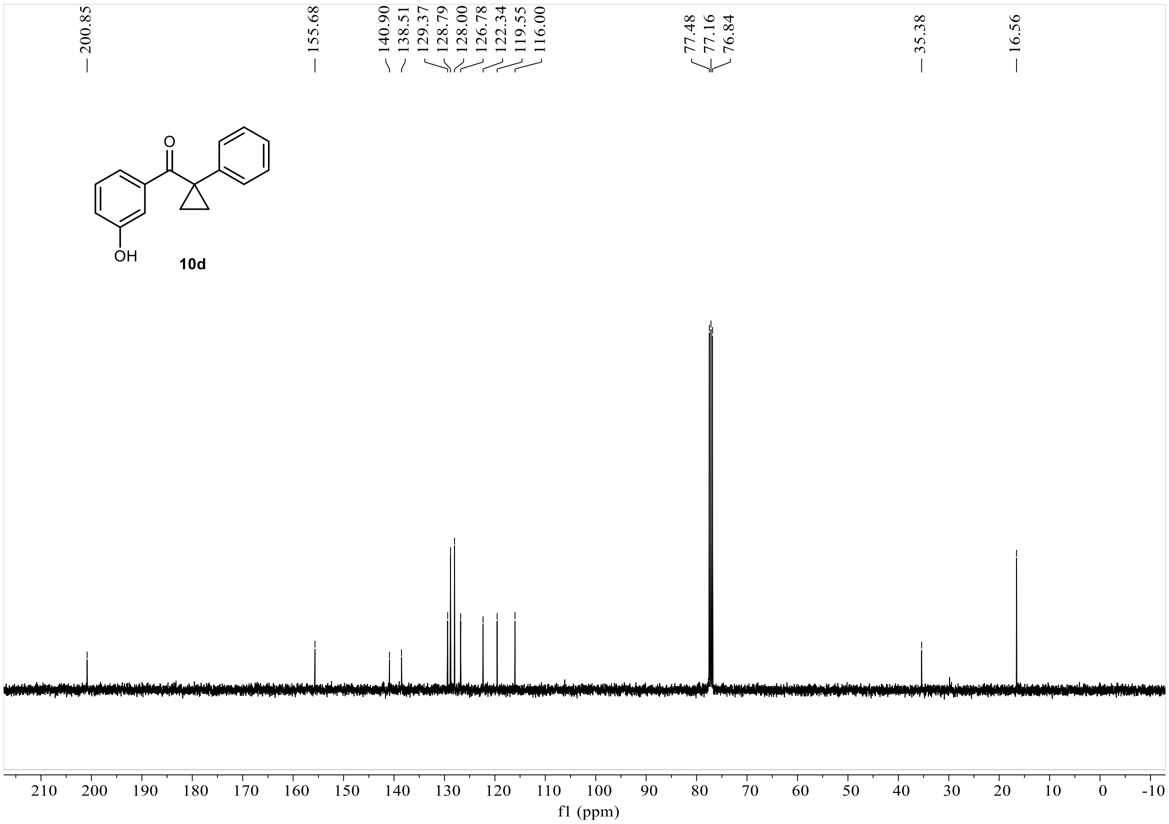

^{1}H NMR (400 MHz, $CDCl_3$) and ^{13}C NMR (101 MHz, $CDCl_3$) spectra of **10e**

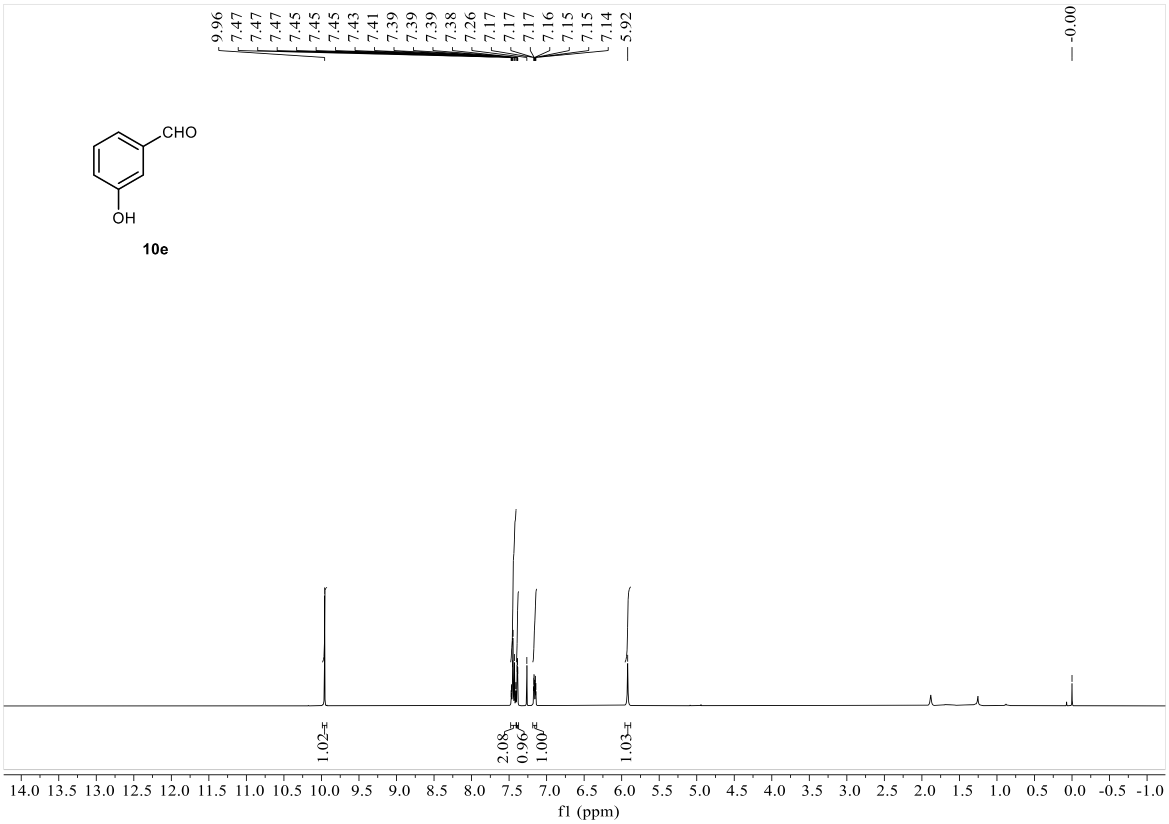

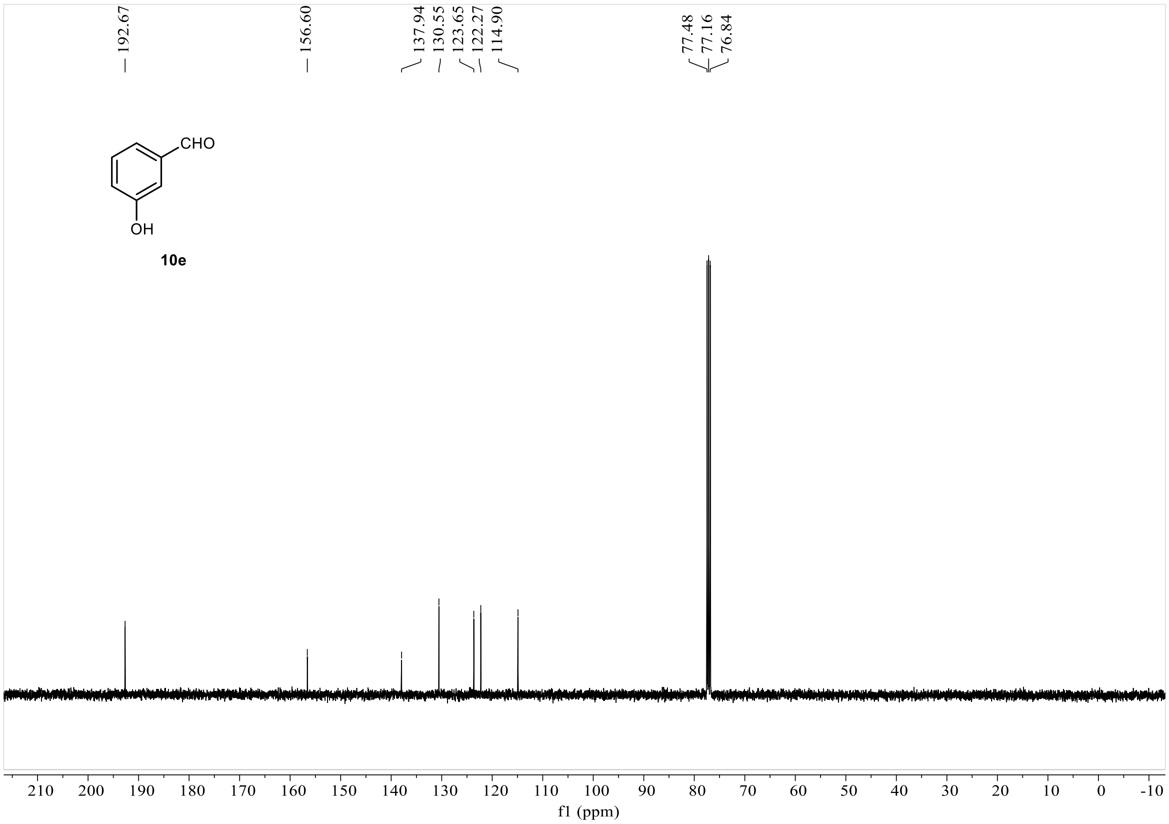

^{1}H NMR (400 MHz, Acetone-d_6) and ^{13}C NMR (101 MHz, Acetone-d_6) spectra of **10f**

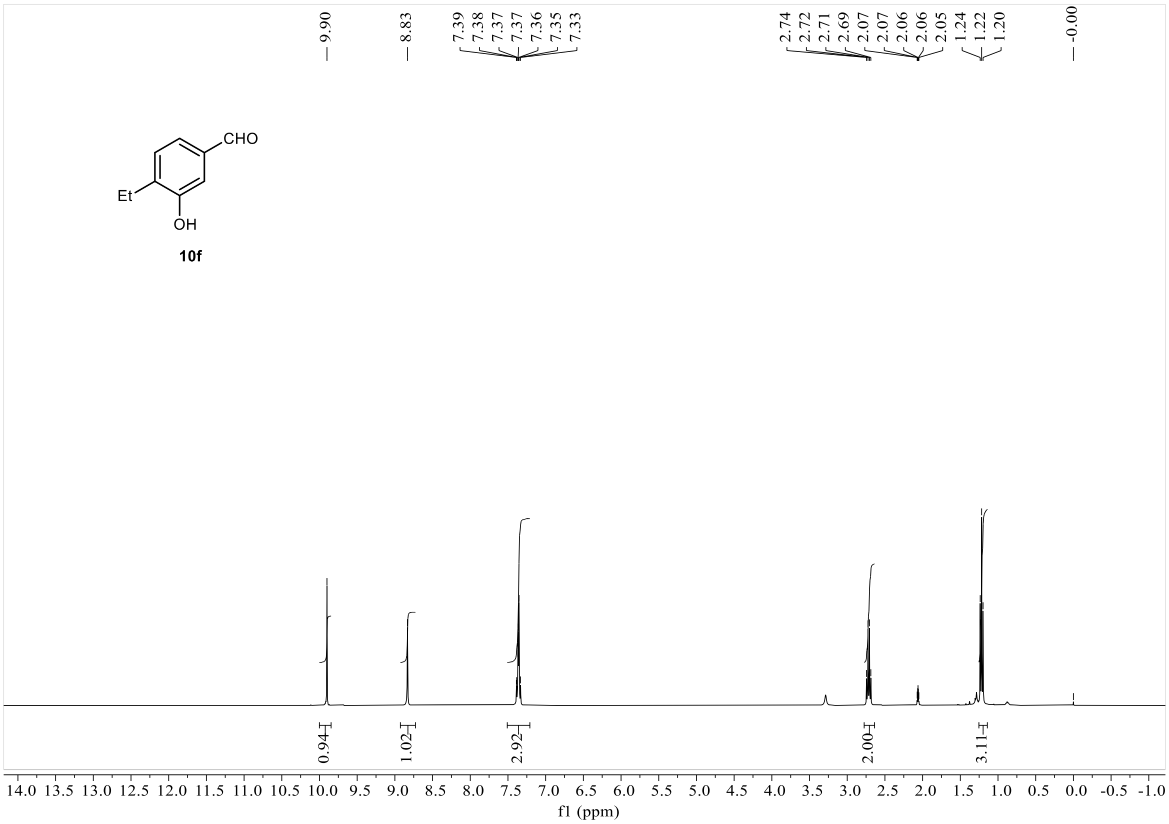

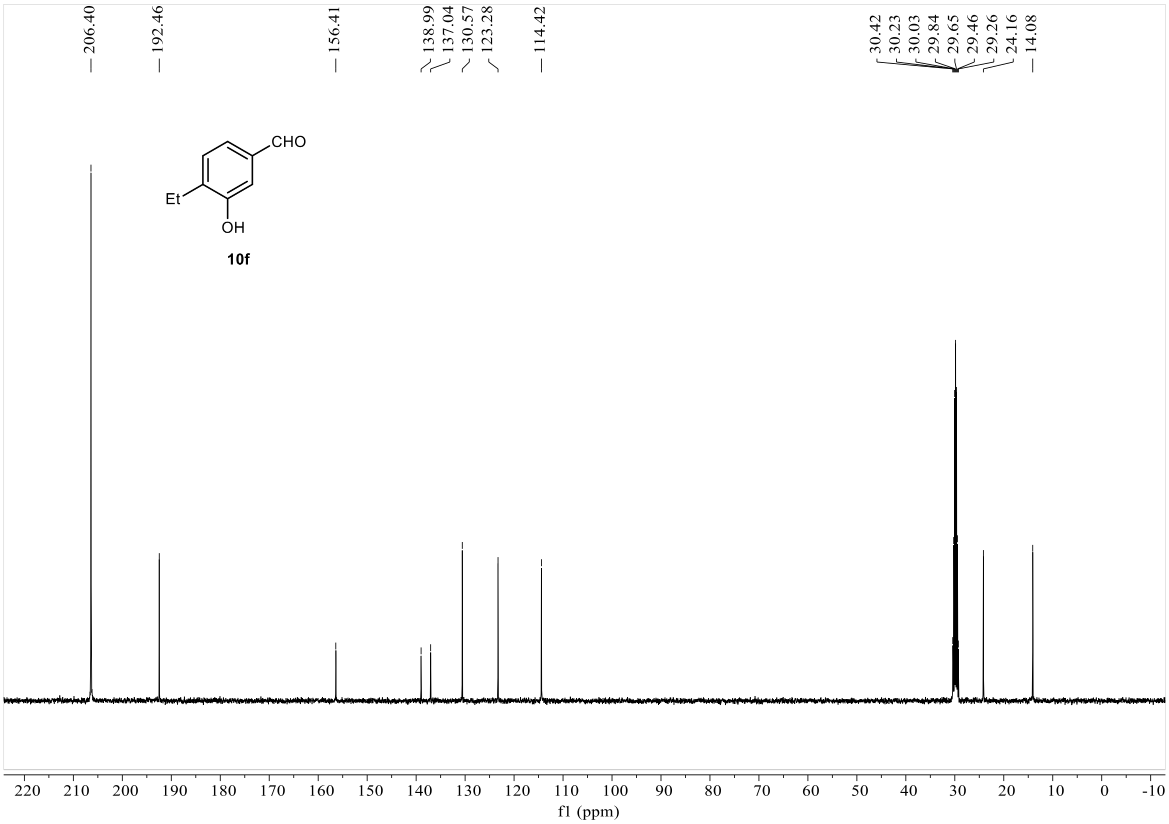

^{1}H NMR (400 MHz, $CDCl_3$) and ^{13}C NMR (101 MHz, $CDCl_3$) spectra of **12a**

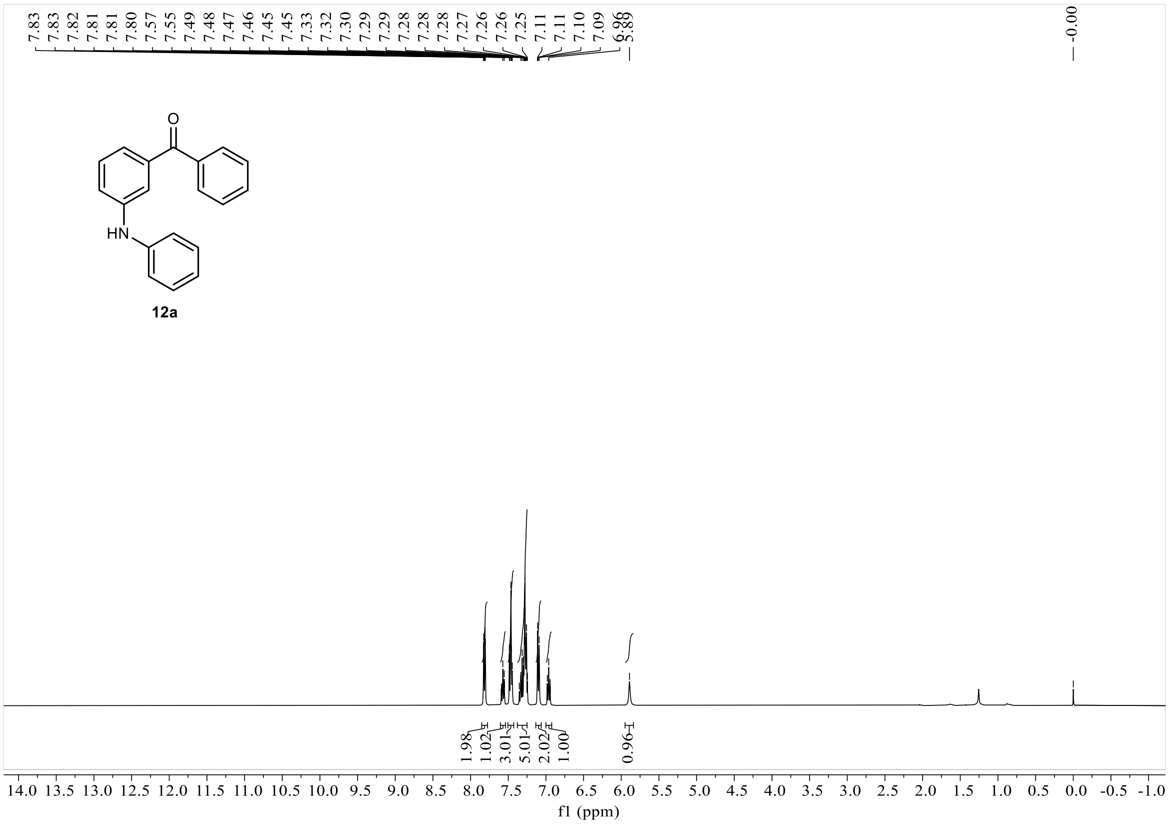

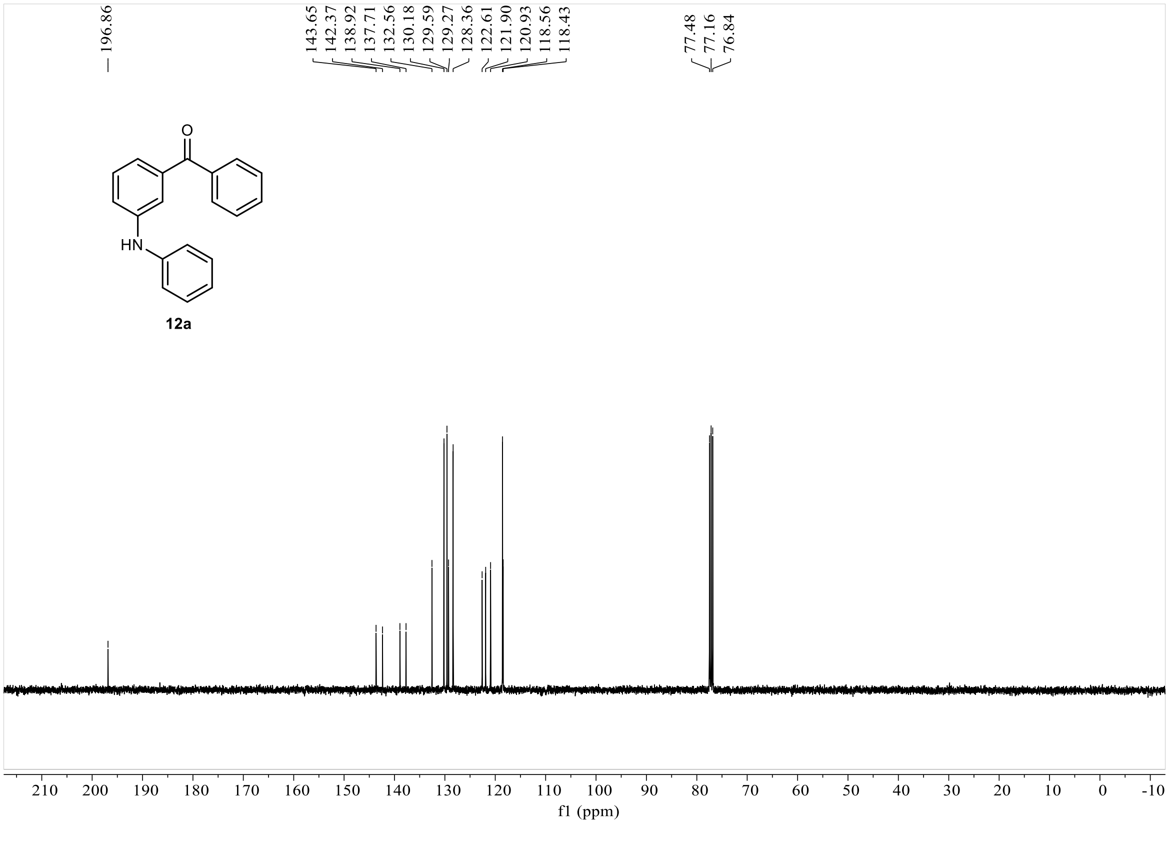

^{1}H NMR (400 MHz, $CDCl_3$) and ^{13}C NMR (101 MHz, $CDCl_3$) spectra of **12b**

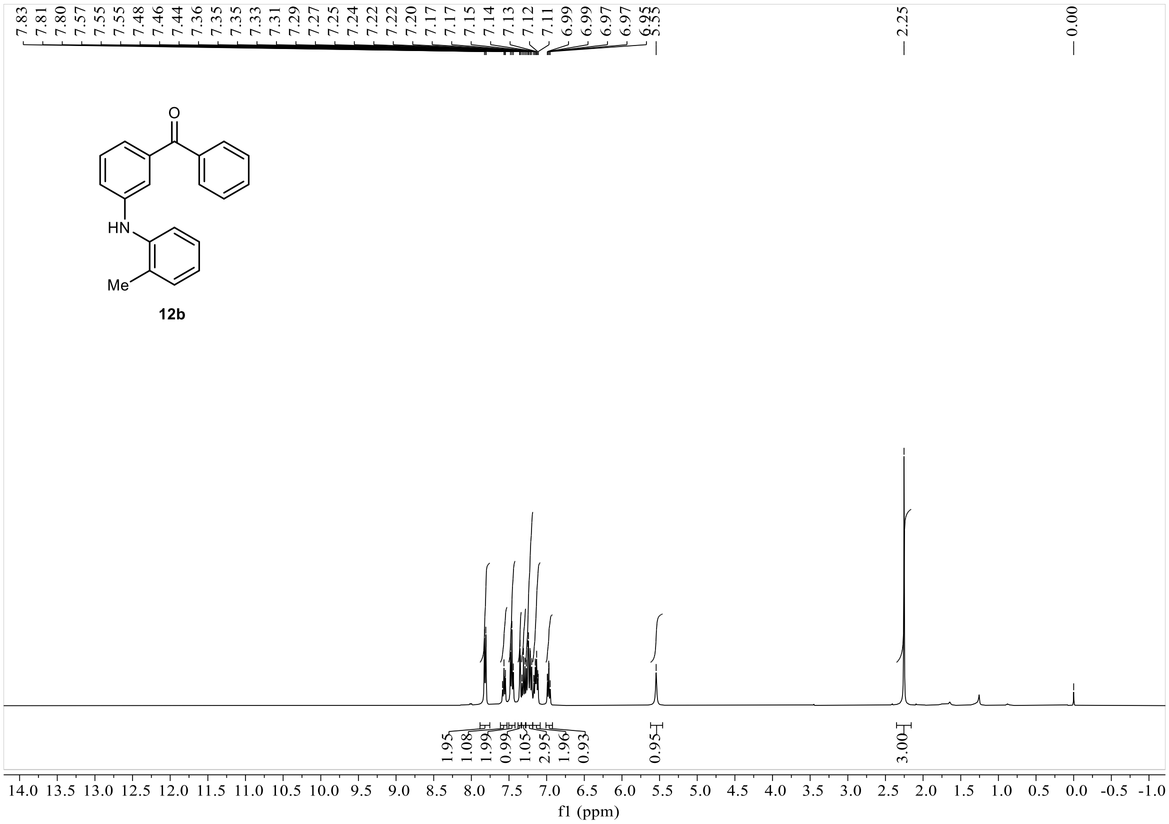

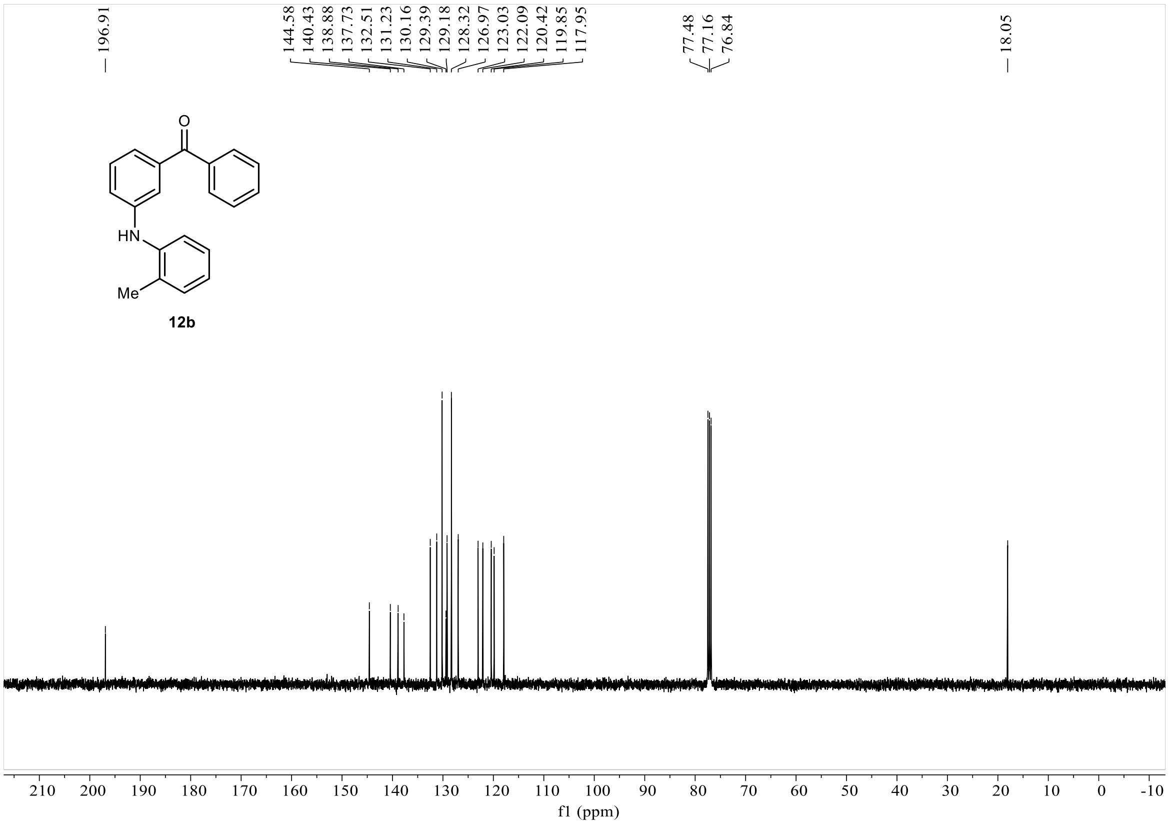

^{1}H NMR (400 MHz, $CDCl_3$) and ^{13}C NMR (101 MHz, $CDCl_3$) spectra of **12c**

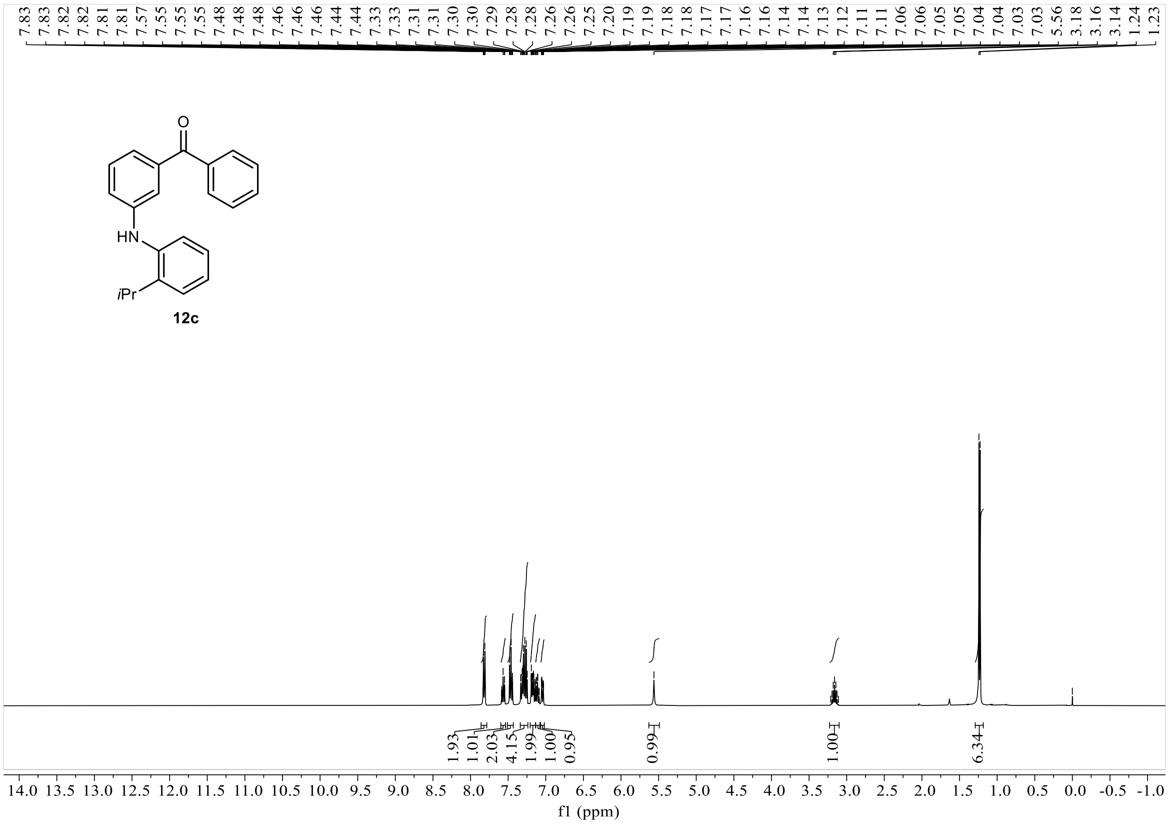

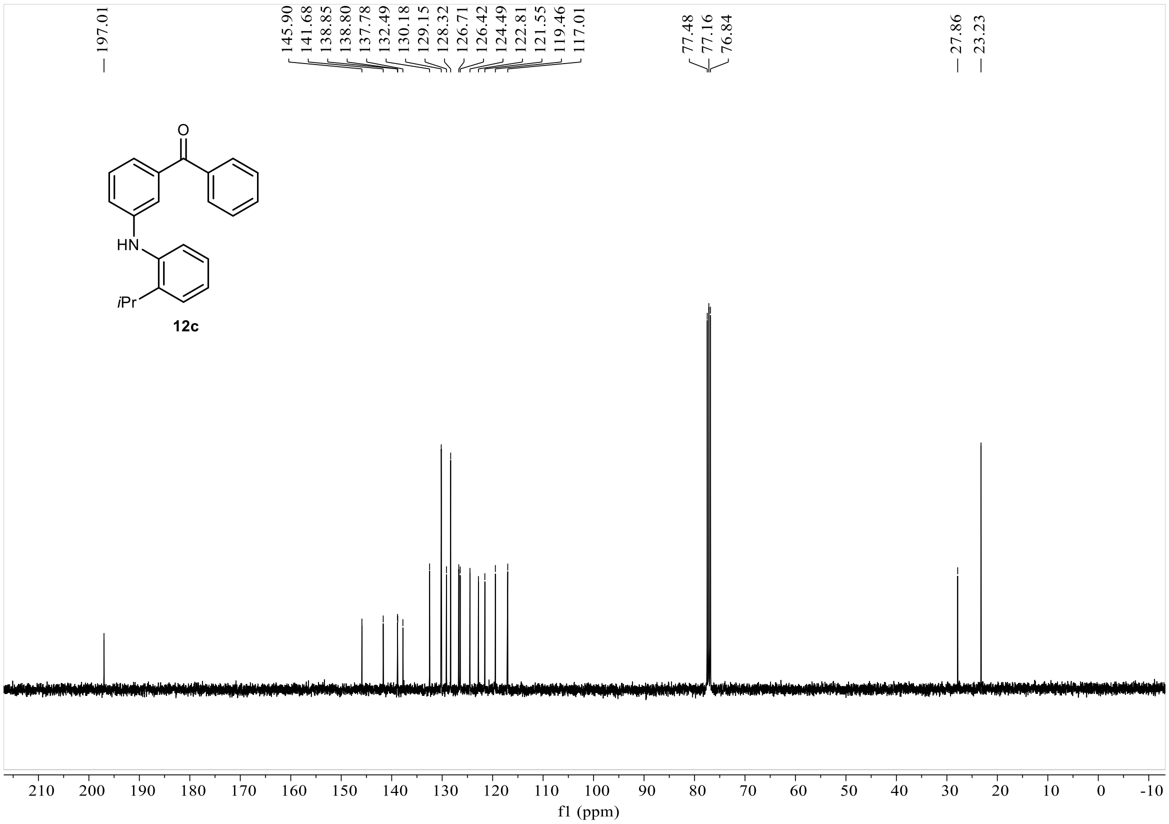

^{1}H NMR (400 MHz, $CDCl_3$) and ^{13}C NMR (101 MHz, $CDCl_3$) spectra of **12d**

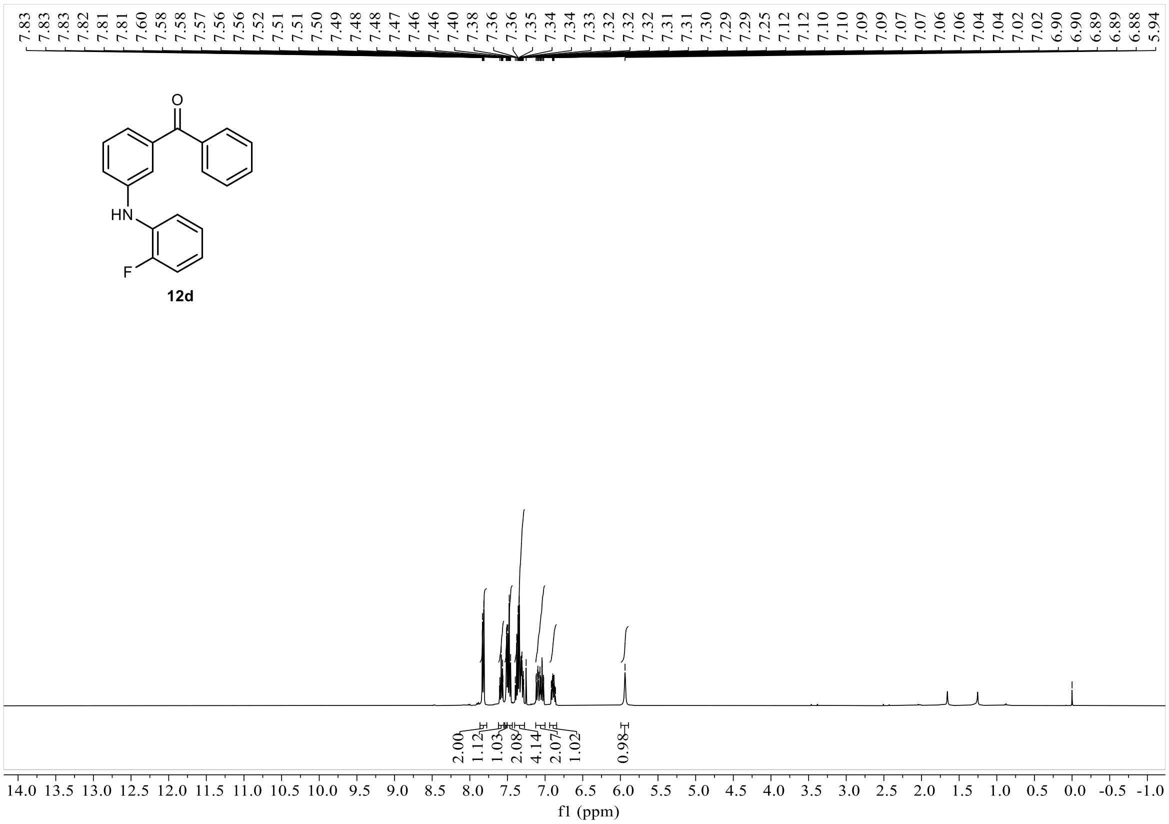

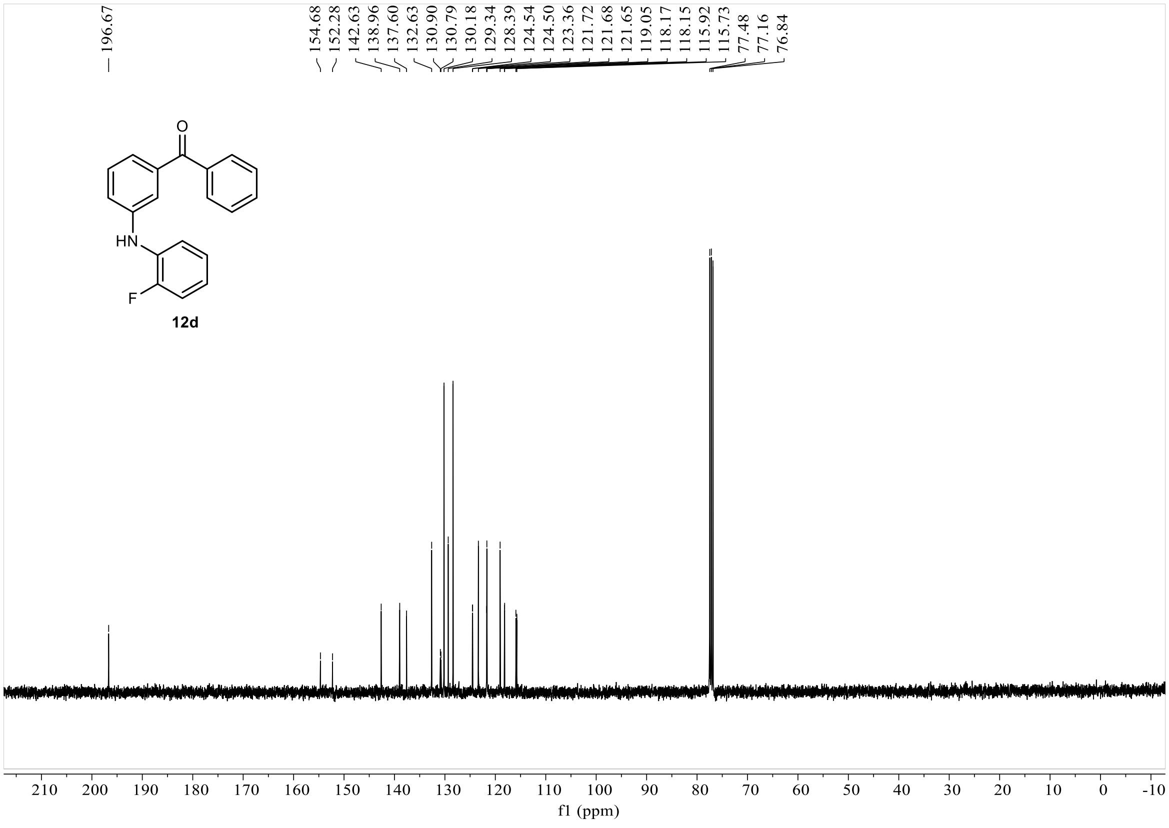

^{19}F NMR (376 MHz, $CDCl_3$) spectrum of **12d**

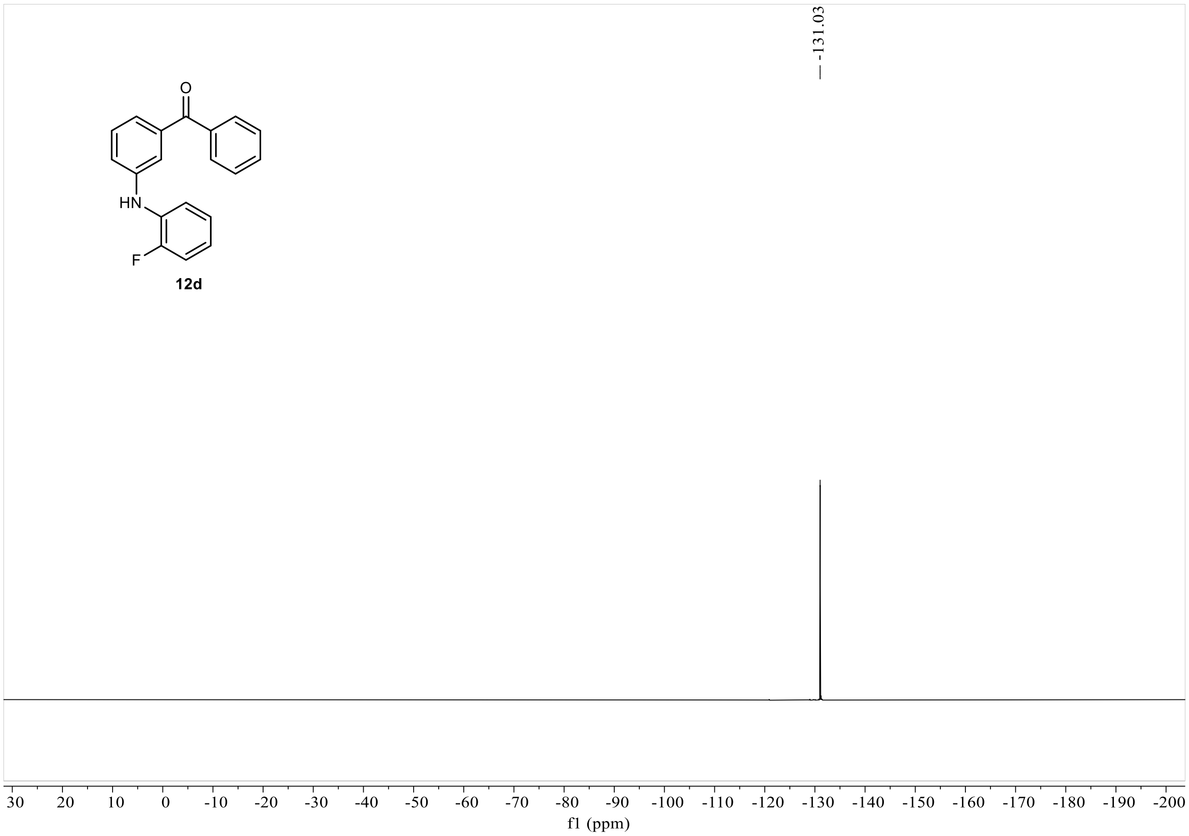

^{1}H NMR (400 MHz, $CDCl_3$) and ^{13}C NMR (101 MHz, $CDCl_3$) spectra of **12e**

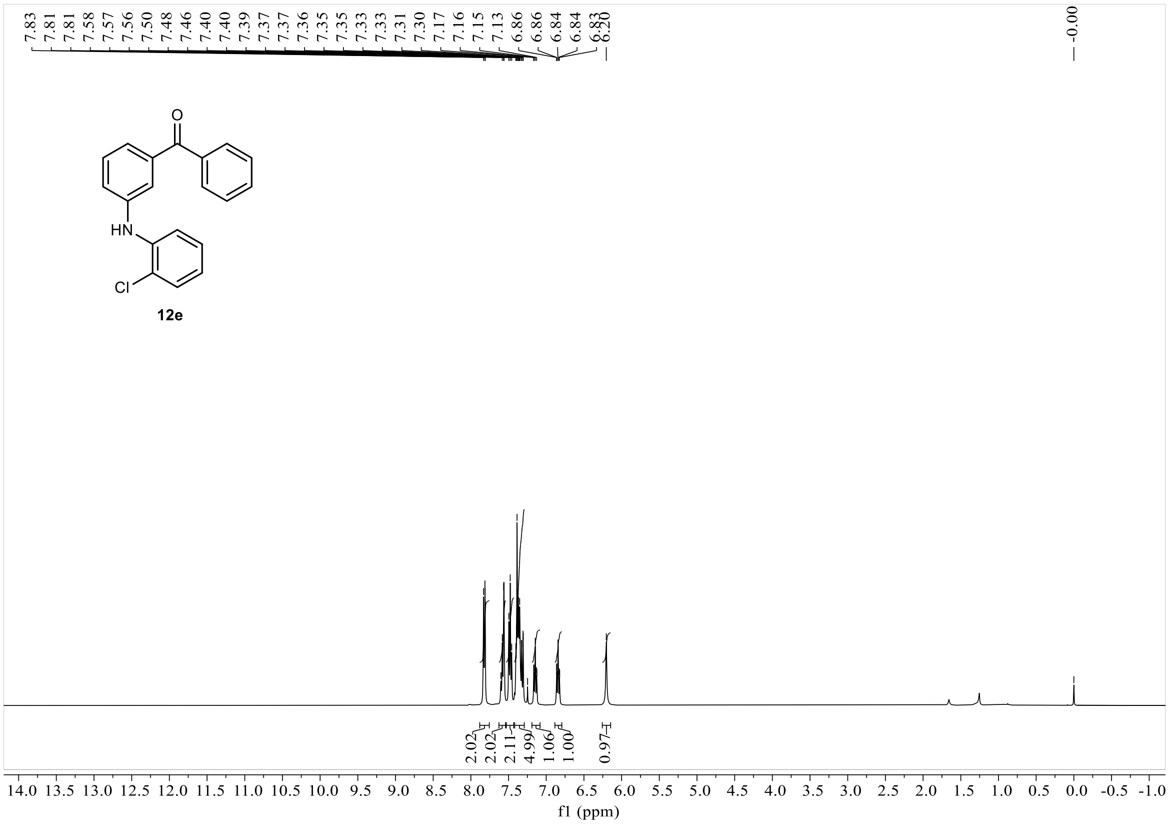

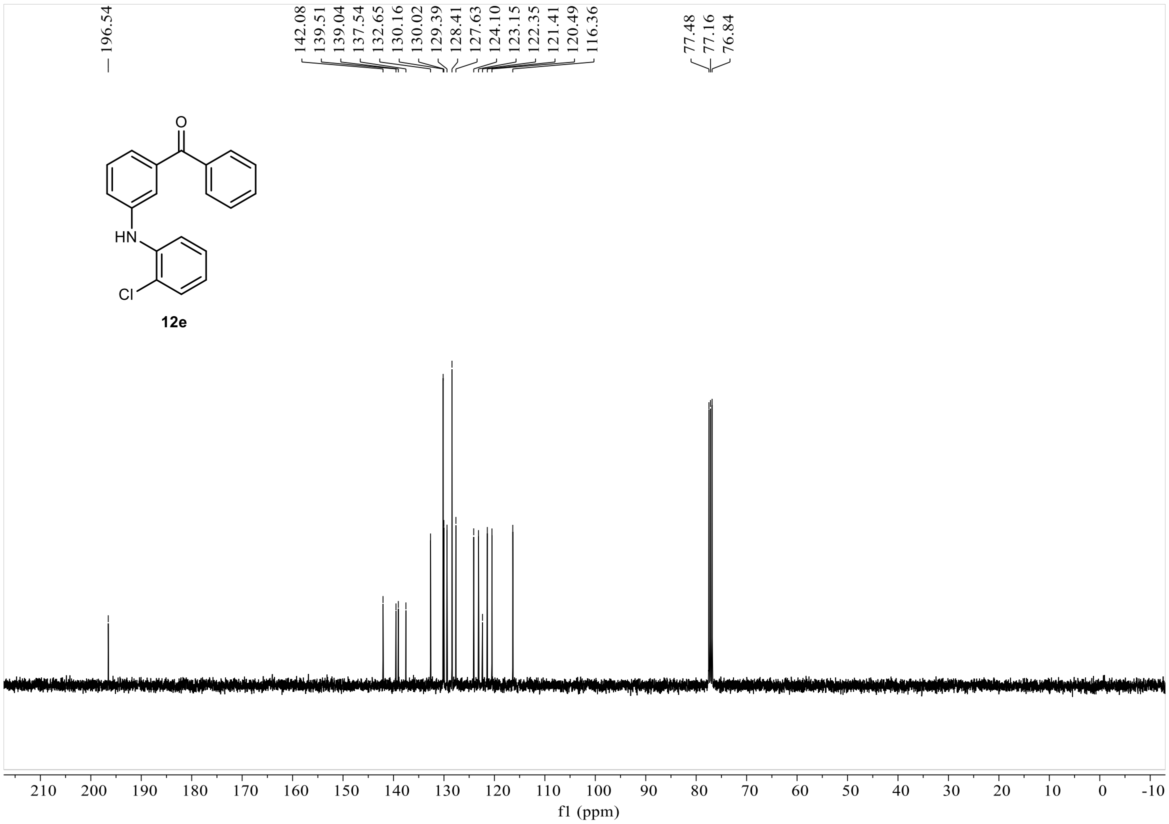

^{1}H NMR (400 MHz, $CDCl_3$) and ^{13}C NMR (101 MHz, $CDCl_3$) spectra of **12f**

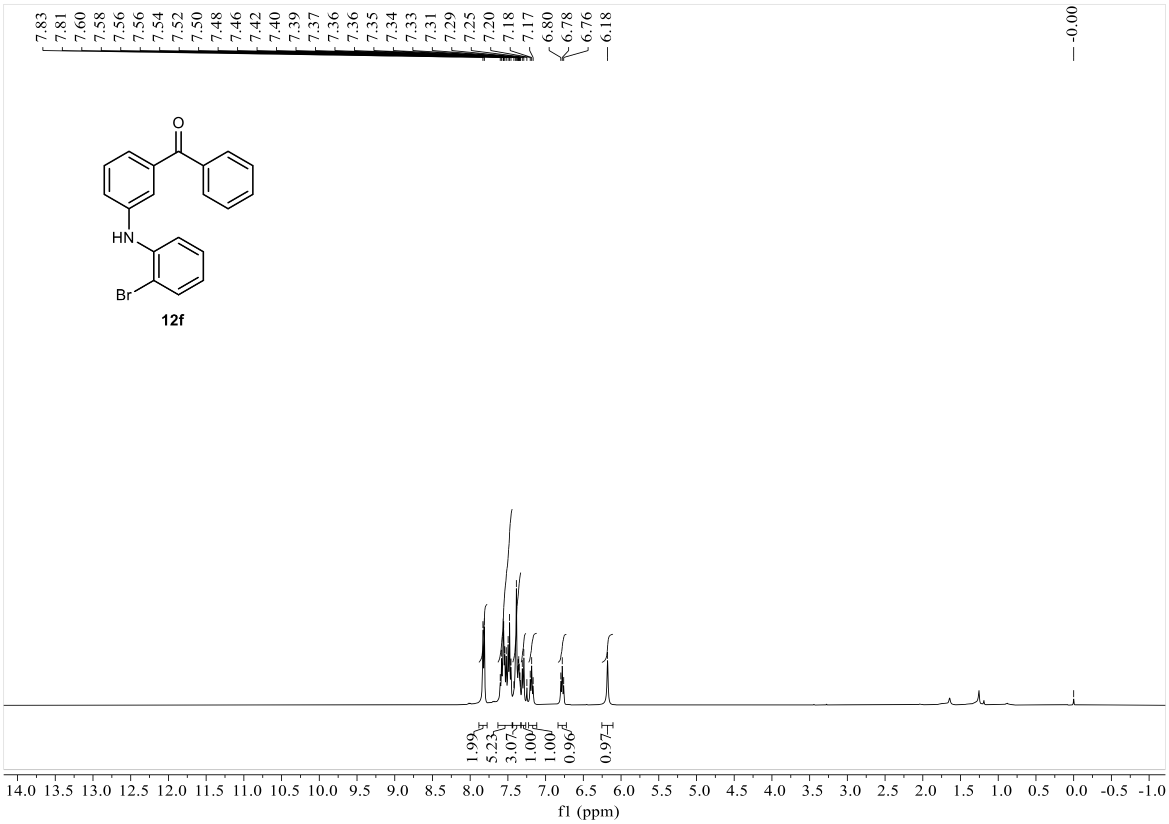

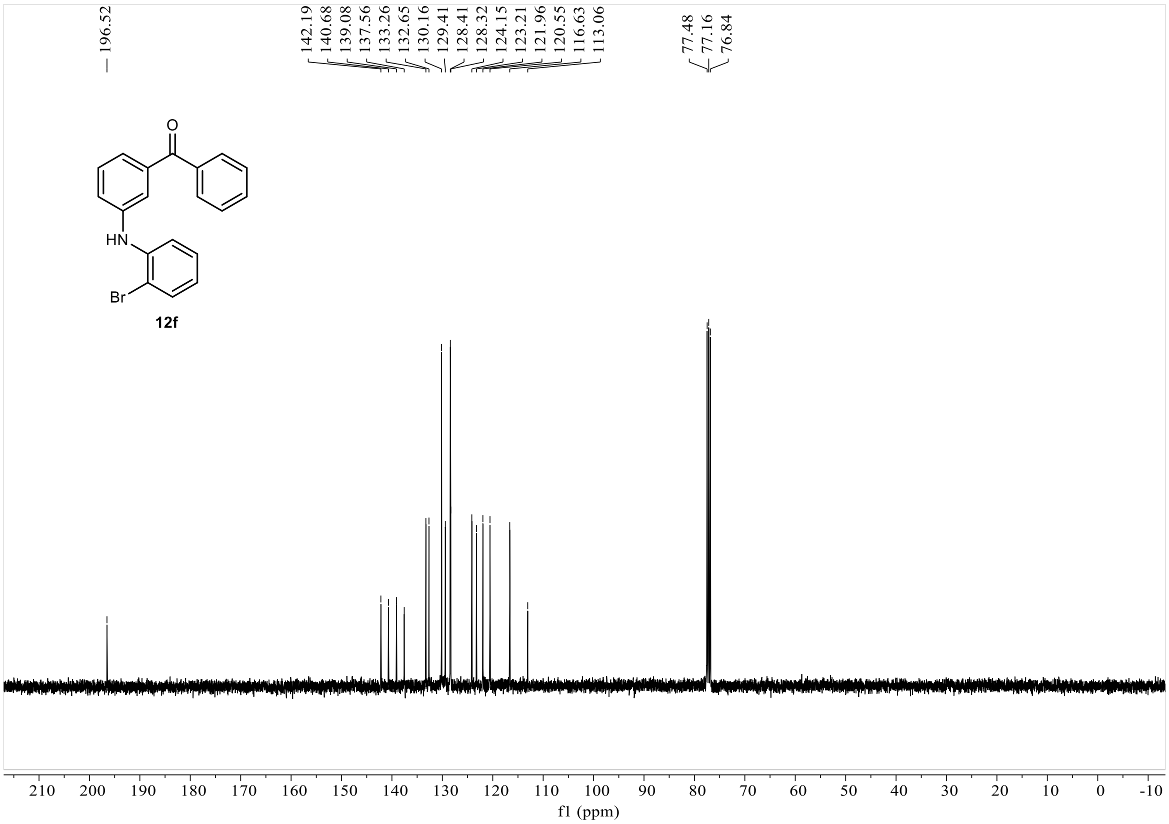

^{1}H NMR (400 MHz, $CDCl_3$) and ^{13}C NMR (101 MHz, $CDCl_3$) spectra of **12g**

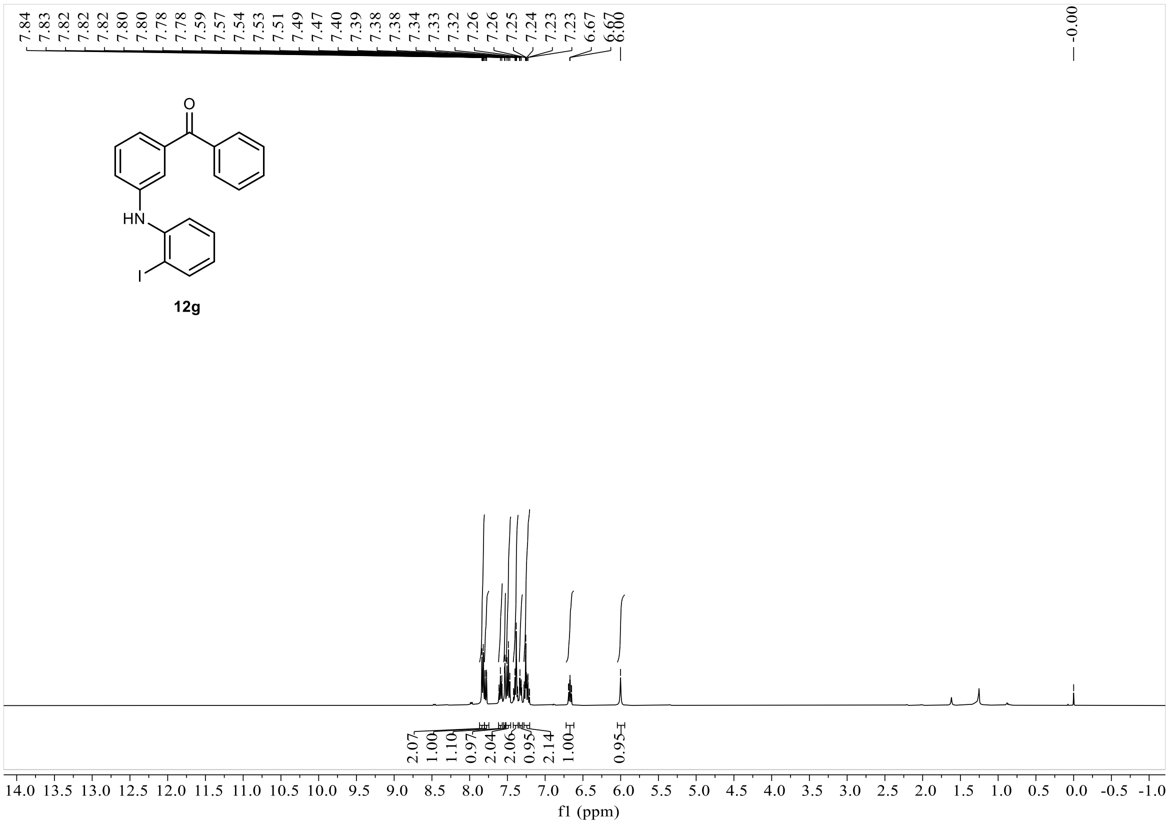

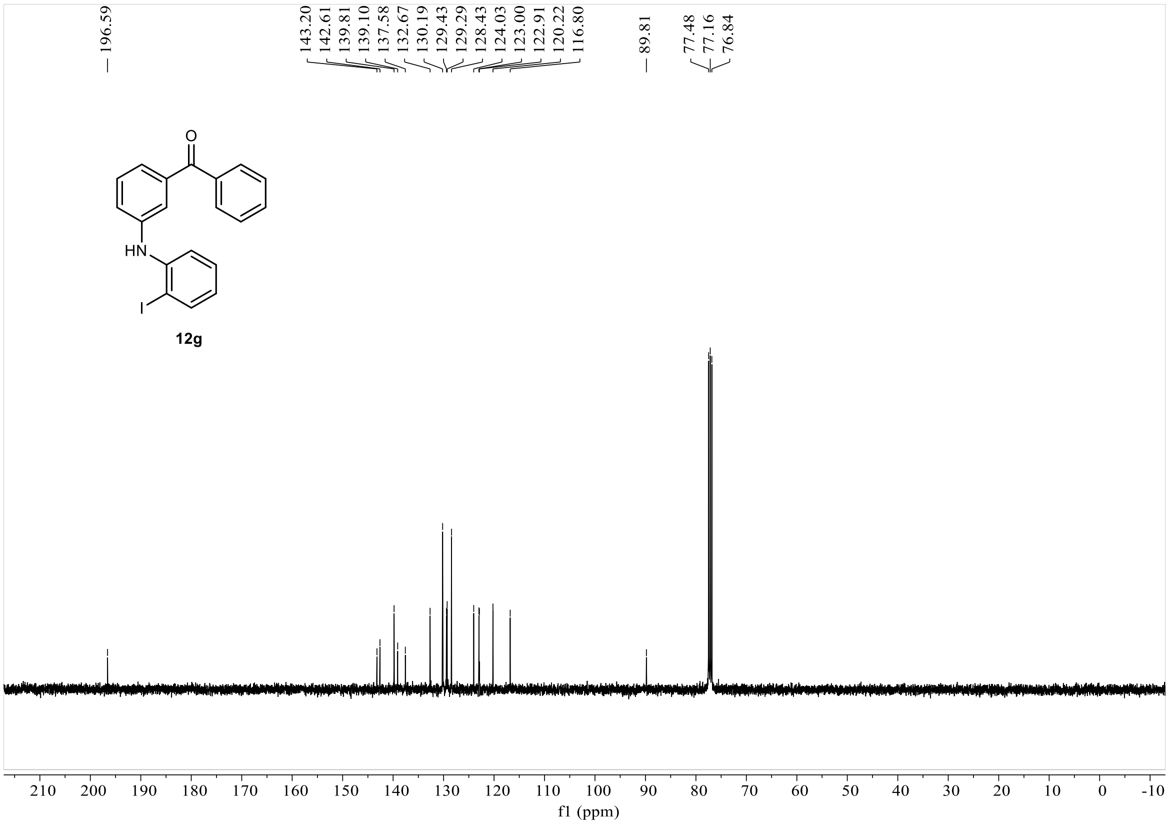

^{1}H NMR (400 MHz, $CDCl_3$) and ^{13}C NMR (101 MHz, $CDCl_3$) spectra of **12h**

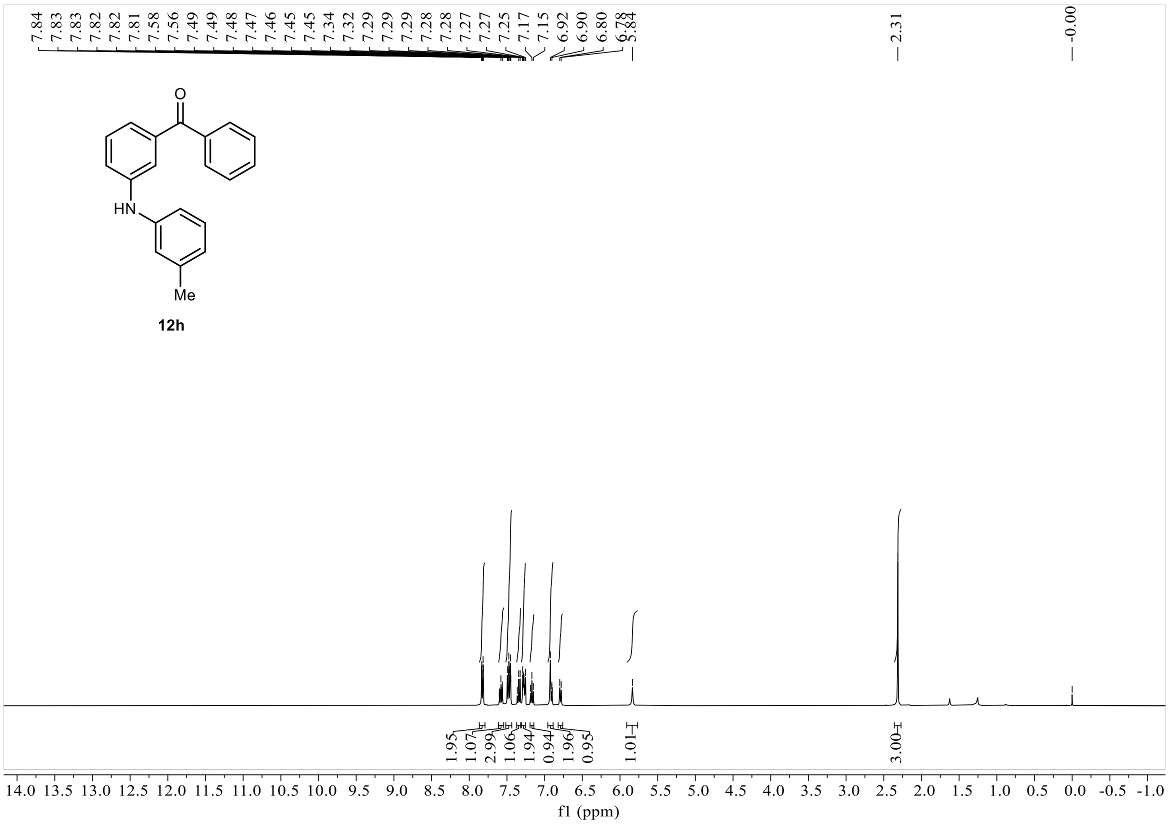

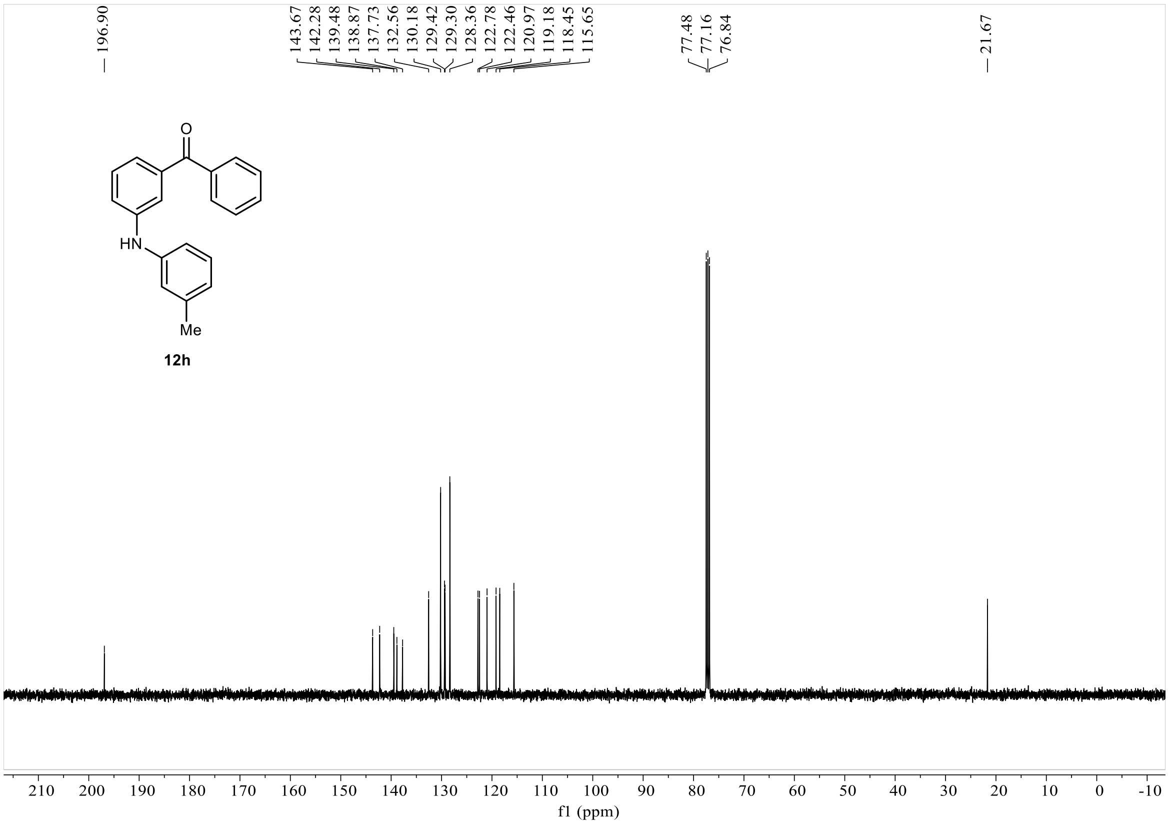

^{1}H NMR (400 MHz, $CDCl_3$) and ^{13}C NMR (101 MHz, $CDCl_3$) spectra of **12i**

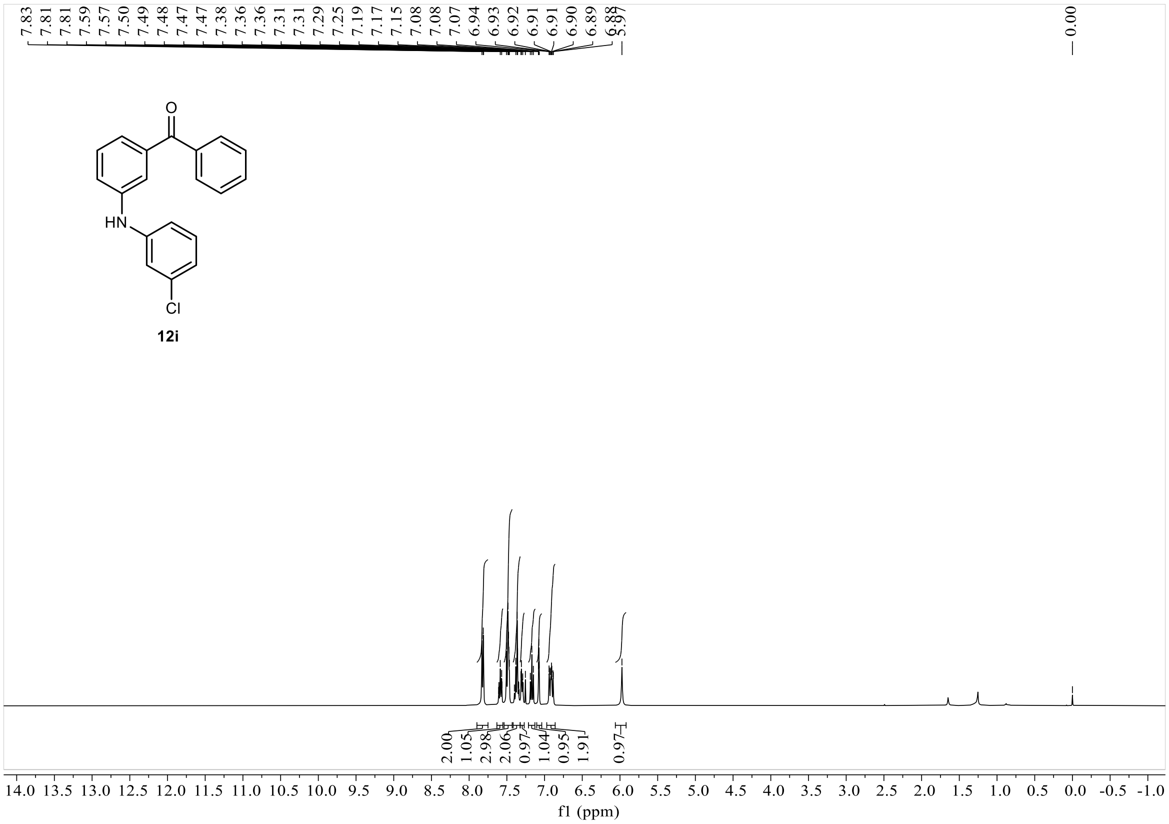

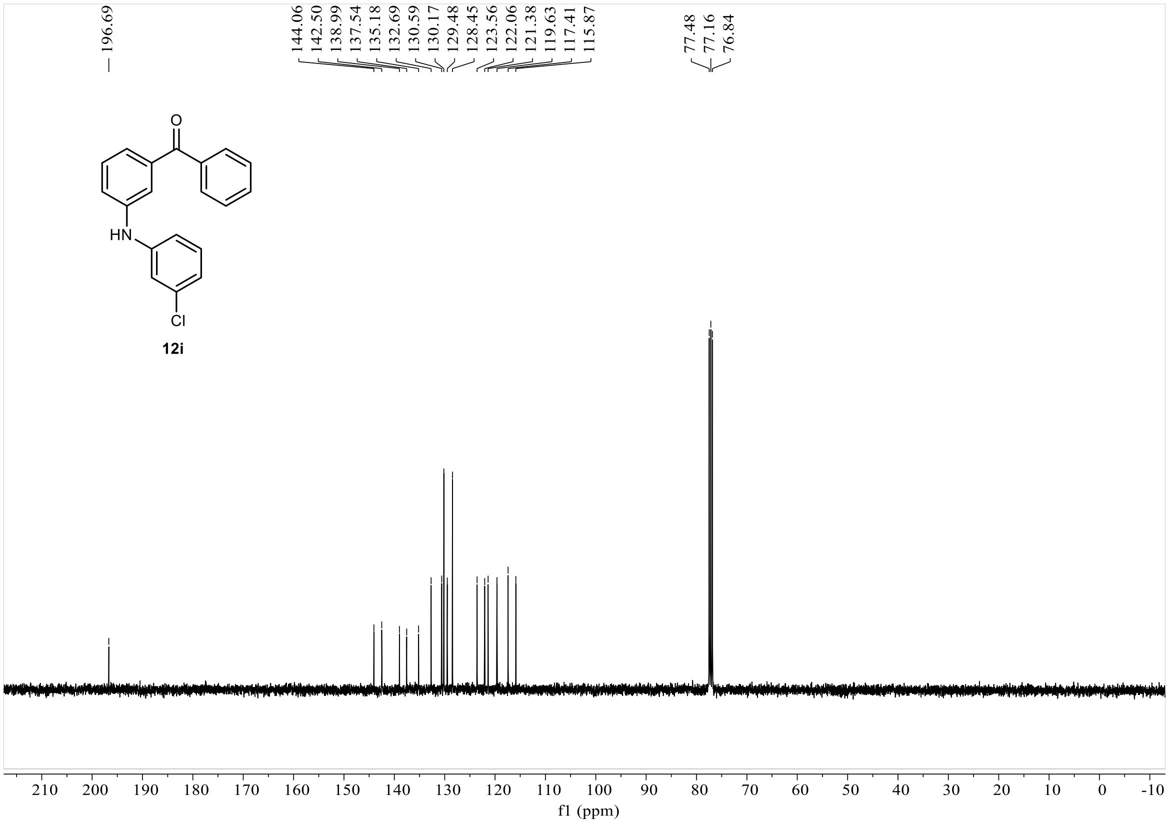

^{1}H NMR (400 MHz, $CDCl_3$) and ^{13}C NMR (101 MHz, $CDCl_3$) spectra of **12j**

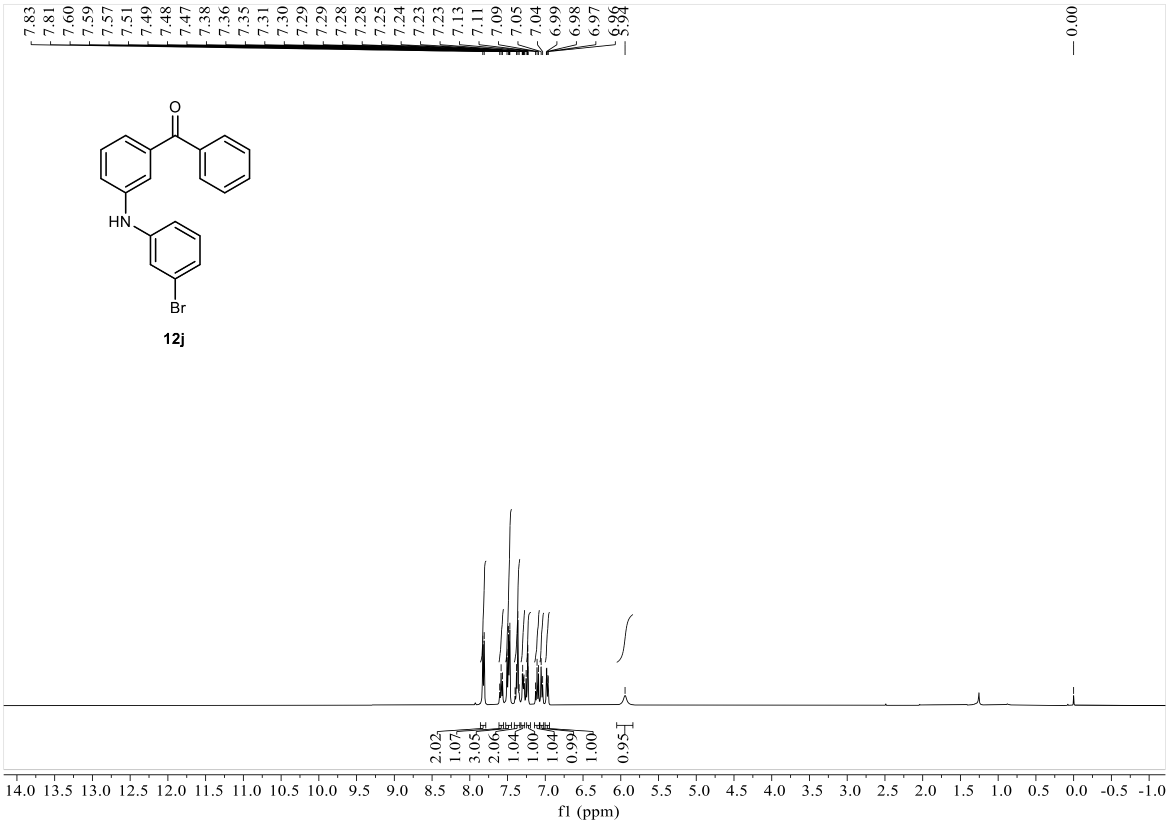

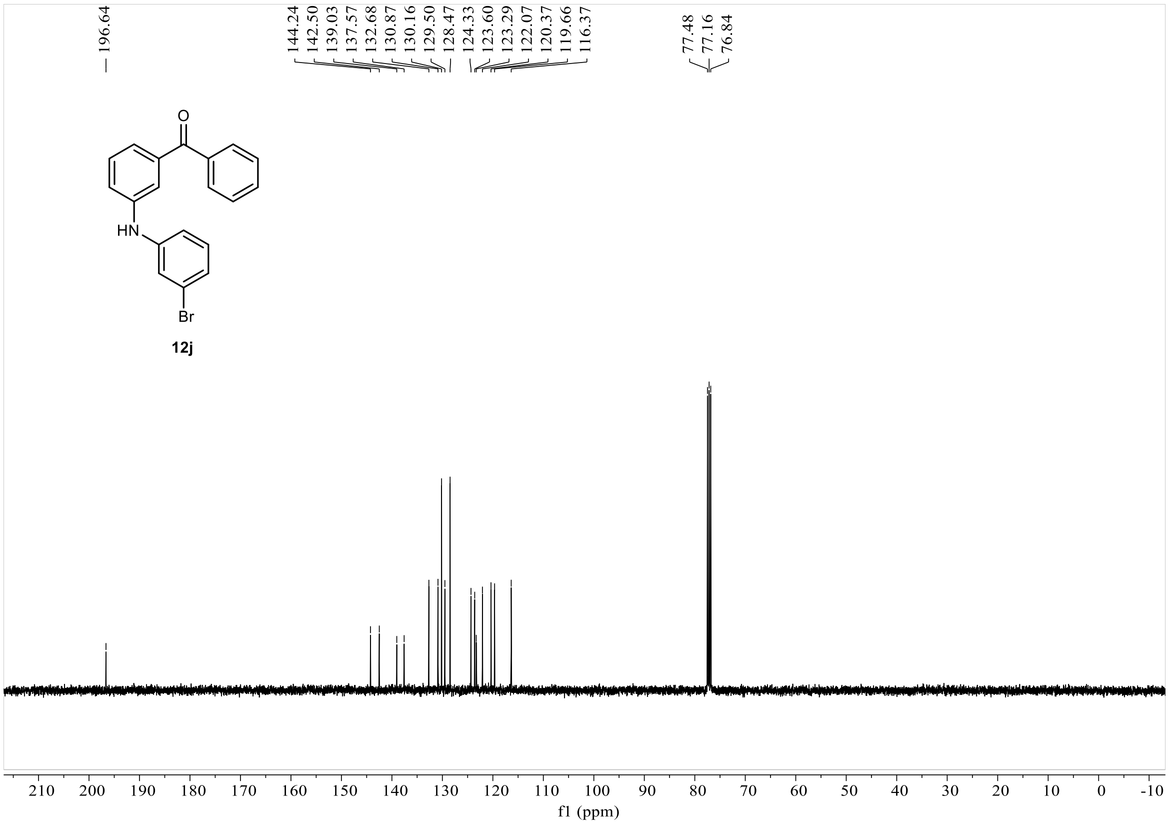

^{1}H NMR (400 MHz, $CDCl_3$) and ^{13}C NMR (101 MHz, $CDCl_3$) spectra of **12k**

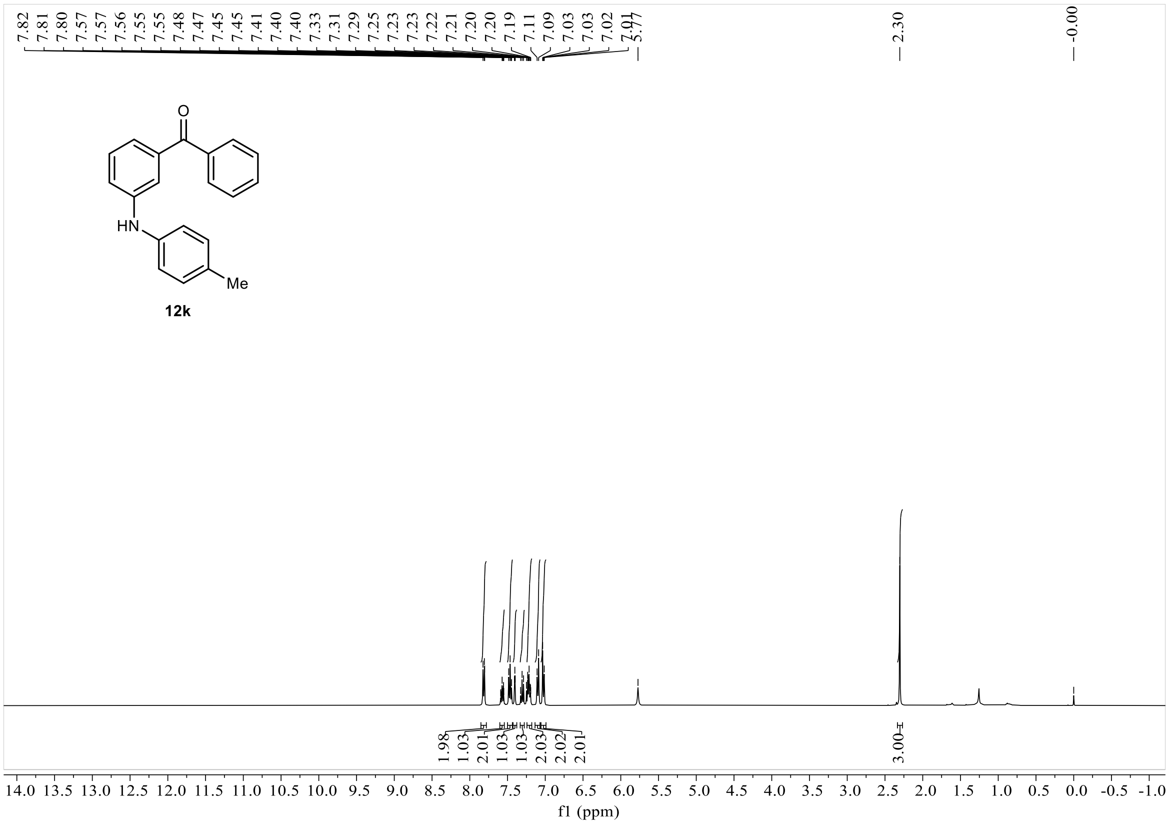

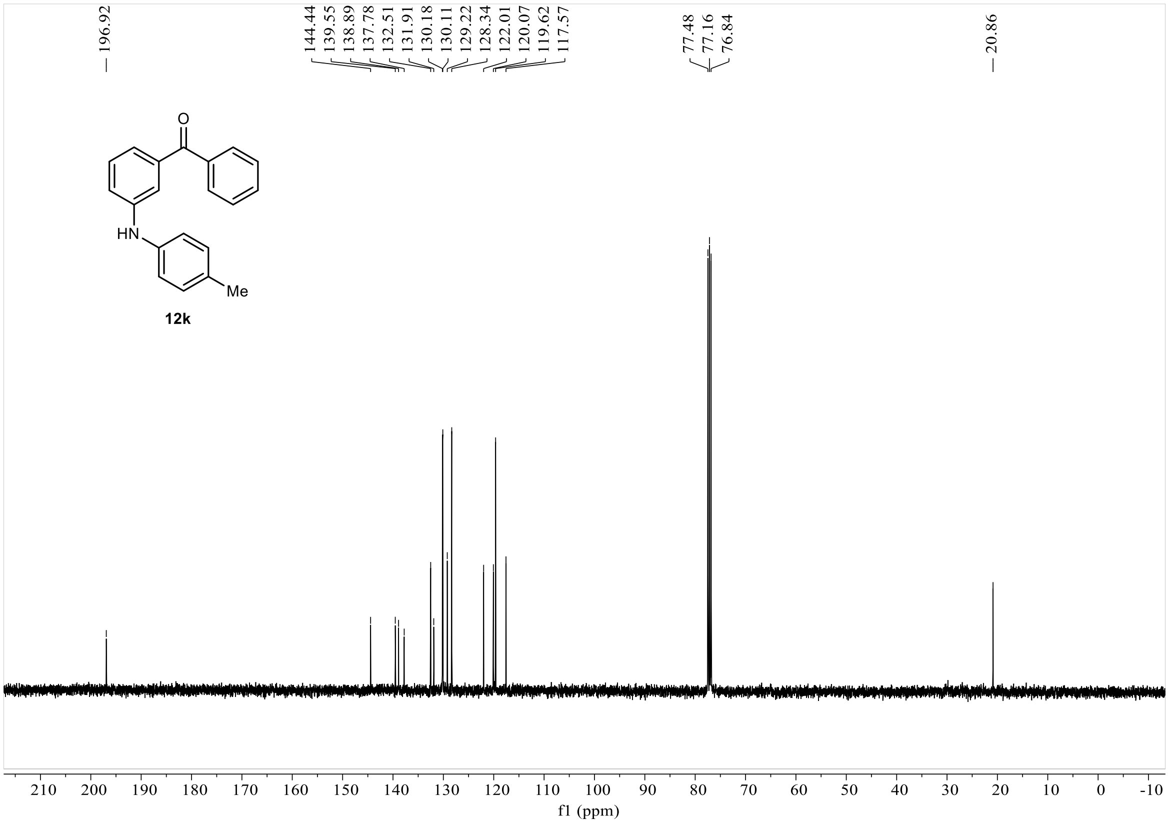

^{1}H NMR (400 MHz, $CDCl_3$) and ^{13}C NMR (101 MHz, $CDCl_3$) spectra of **12l**

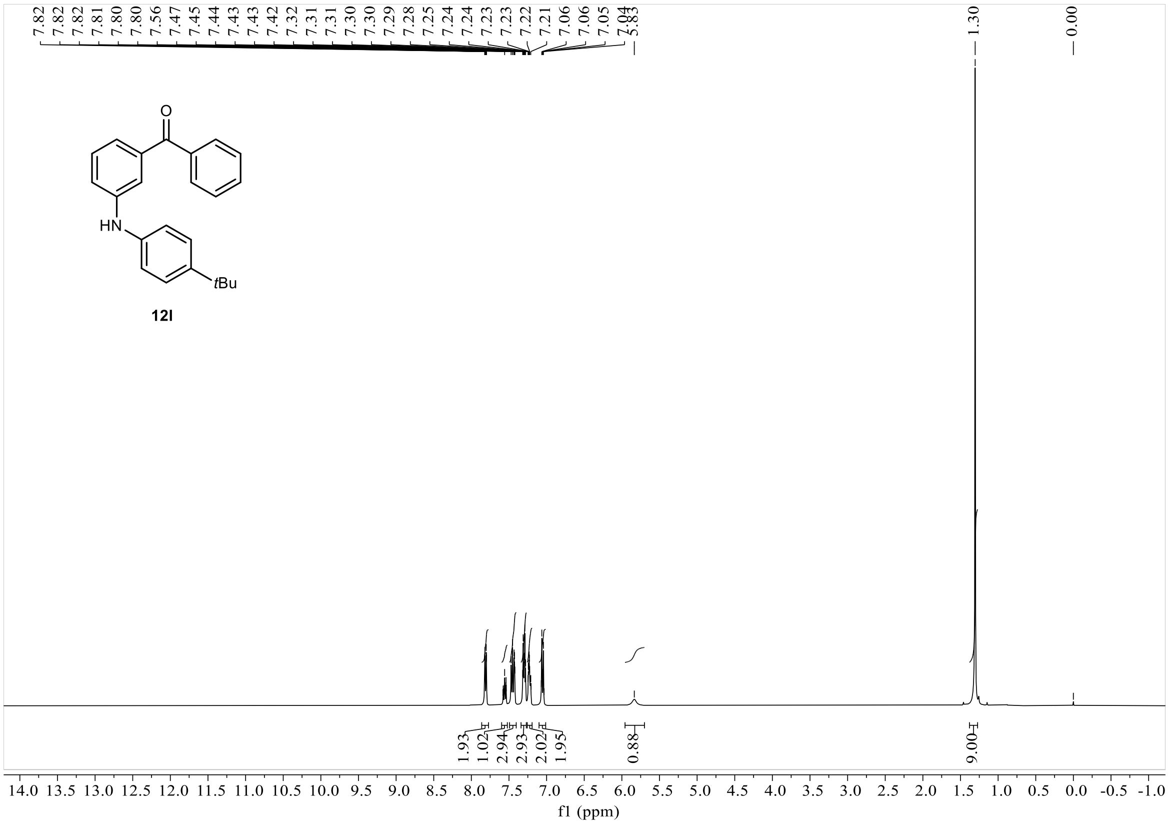

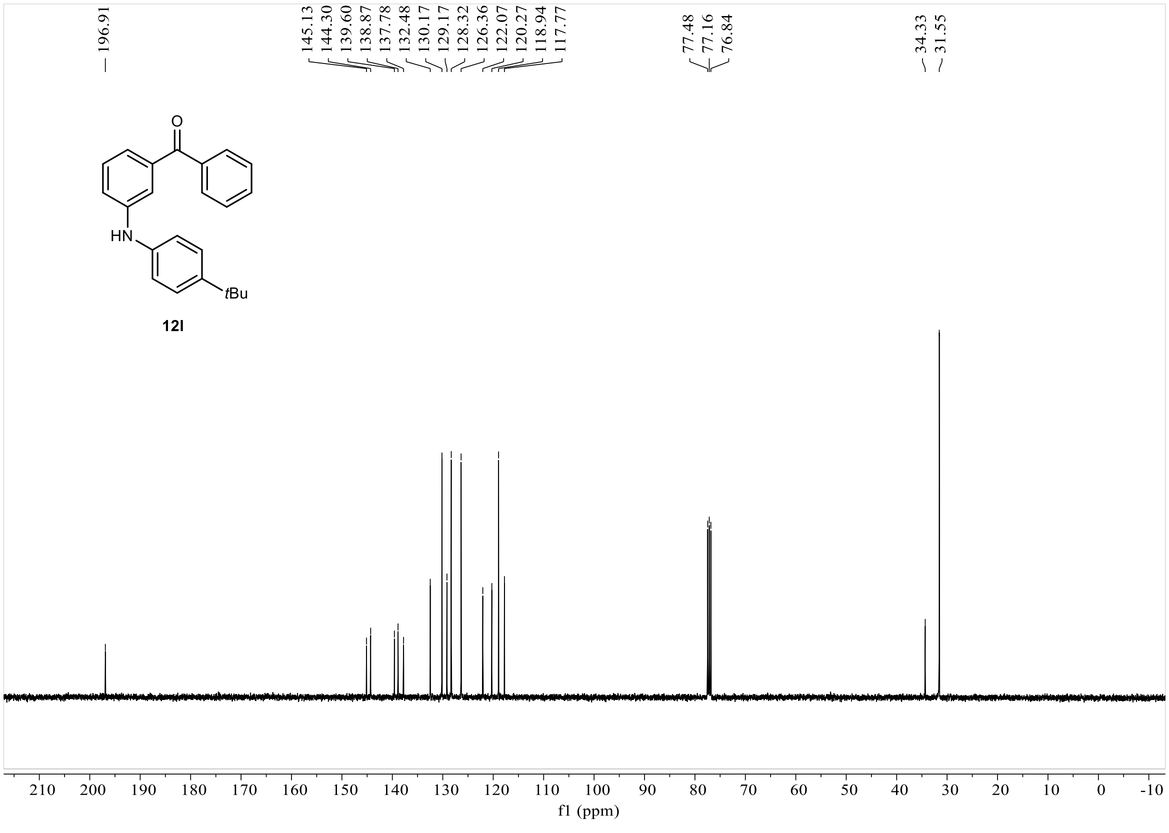

^{1}H NMR (400 MHz, $CDCl_3$) and ^{13}C NMR (101 MHz, $CDCl_3$) spectra of **12m**

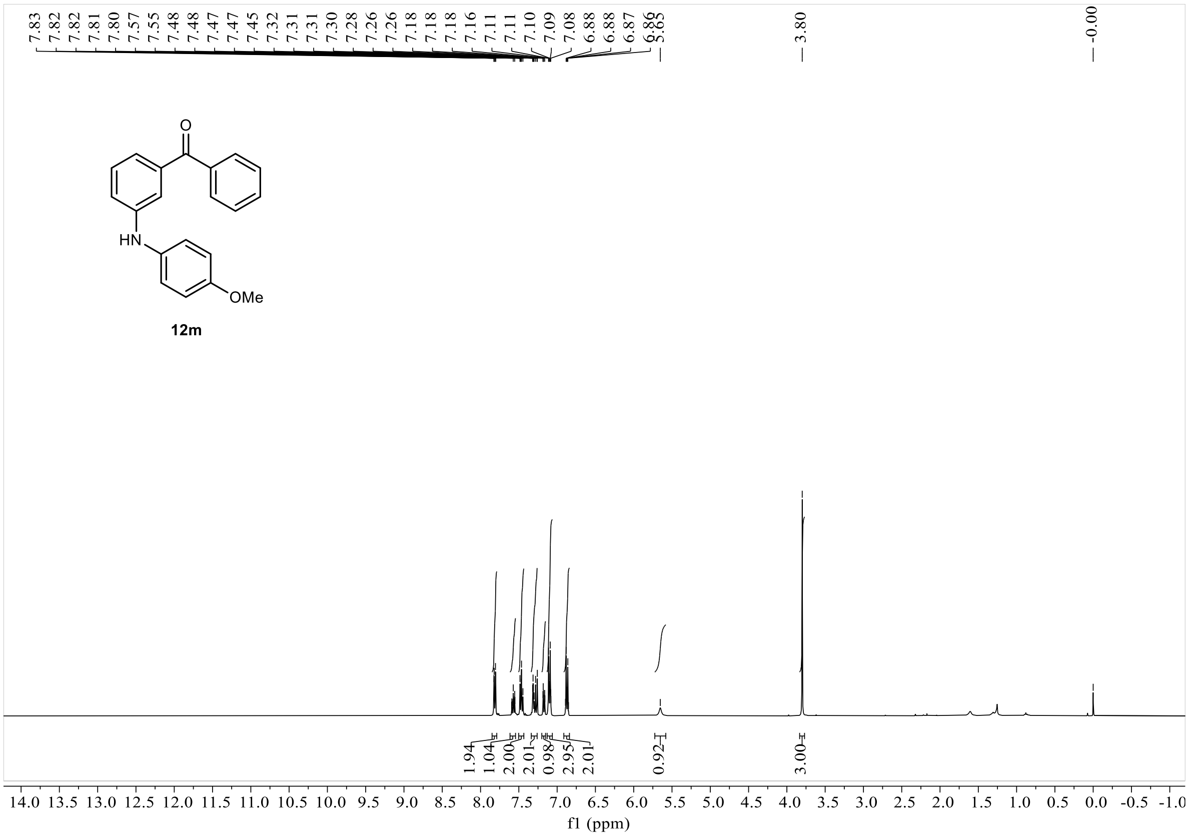

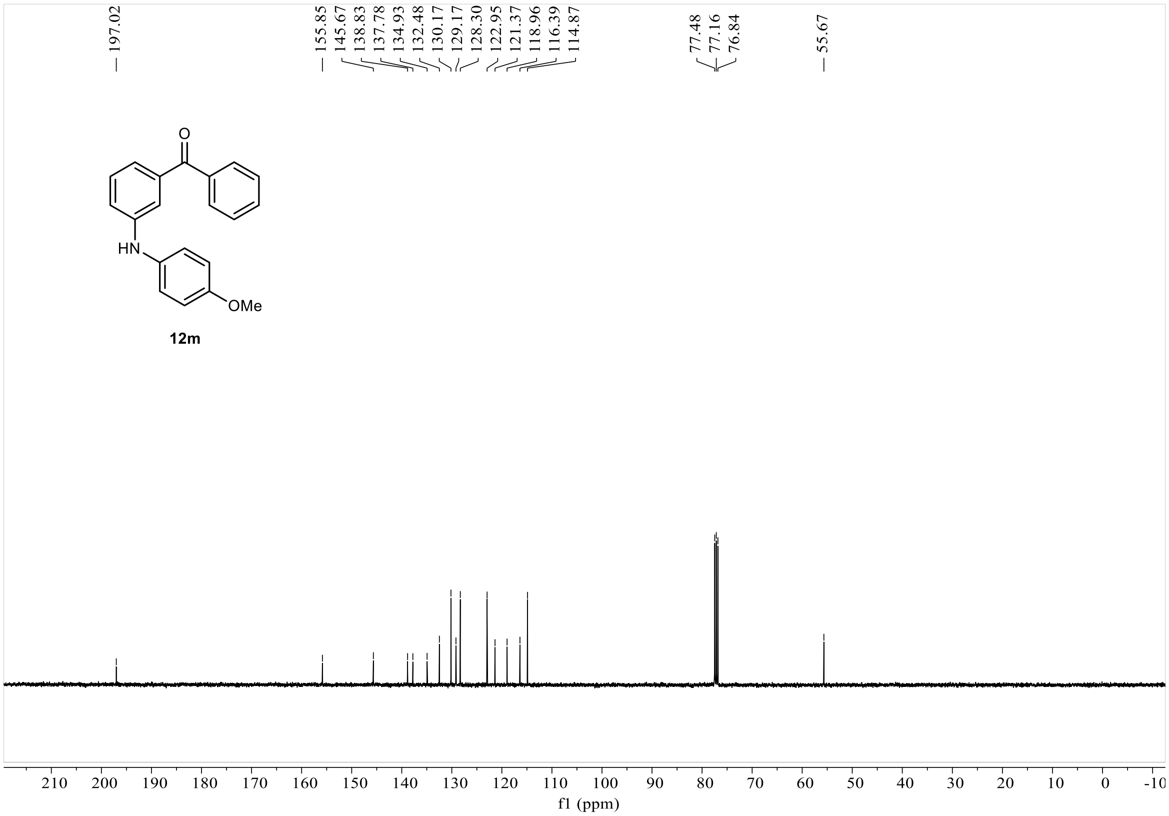

^{1}H NMR (400 MHz, $CDCl_3$) and ^{13}C NMR (101 MHz, $CDCl_3$) spectra of **12n**

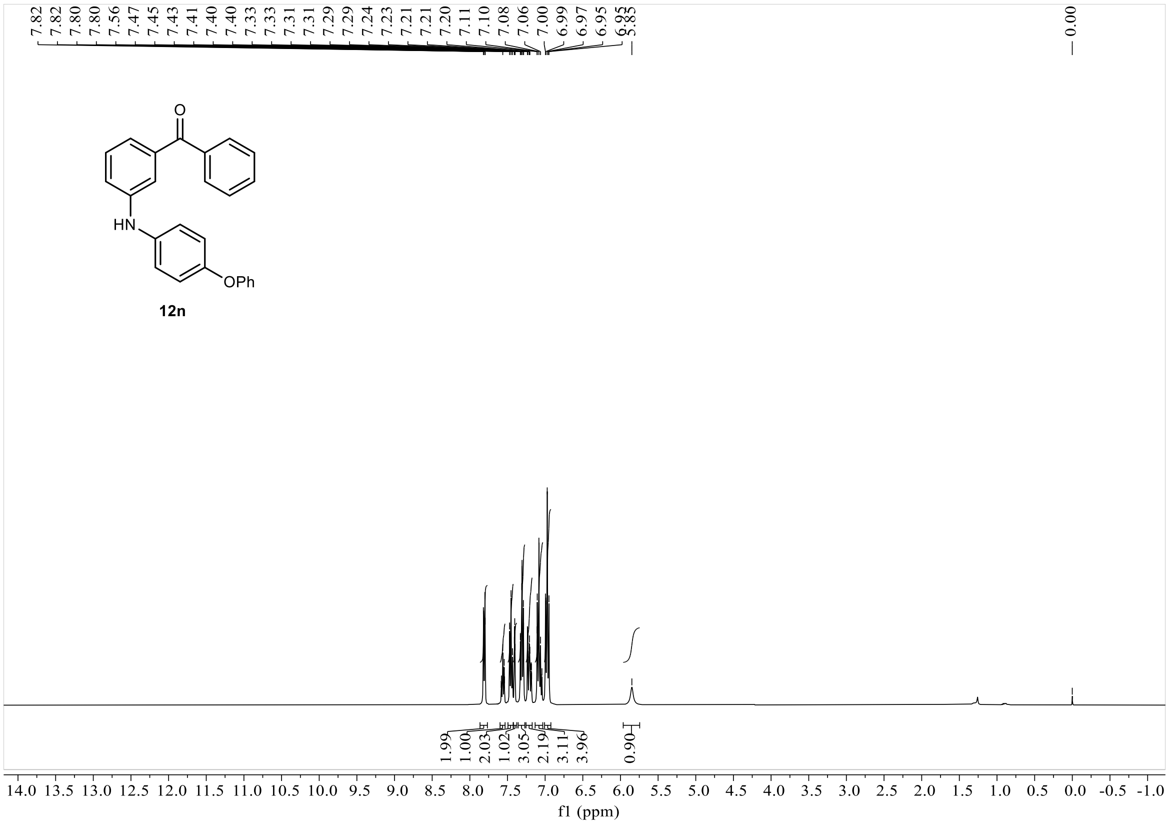

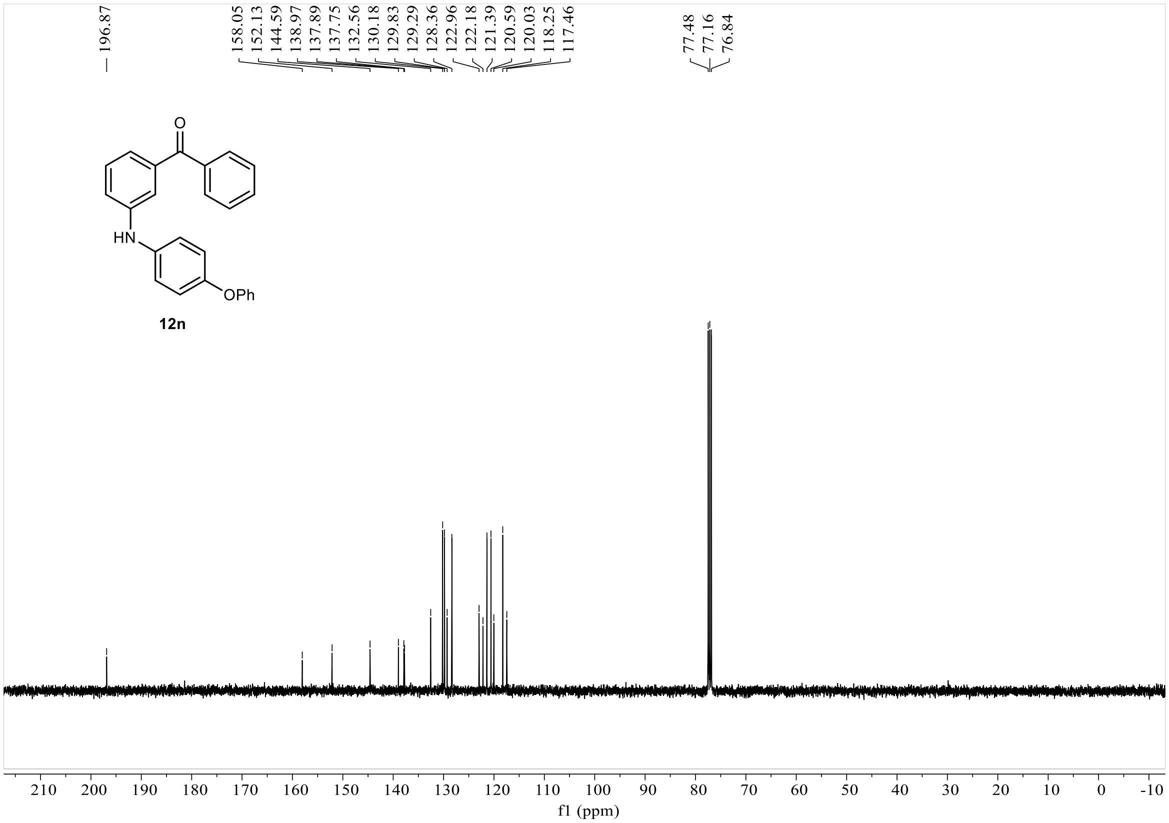

^{1}H NMR (400 MHz, $CDCl_3$) and ^{13}C NMR (101 MHz, $CDCl_3$) spectra of **12o**

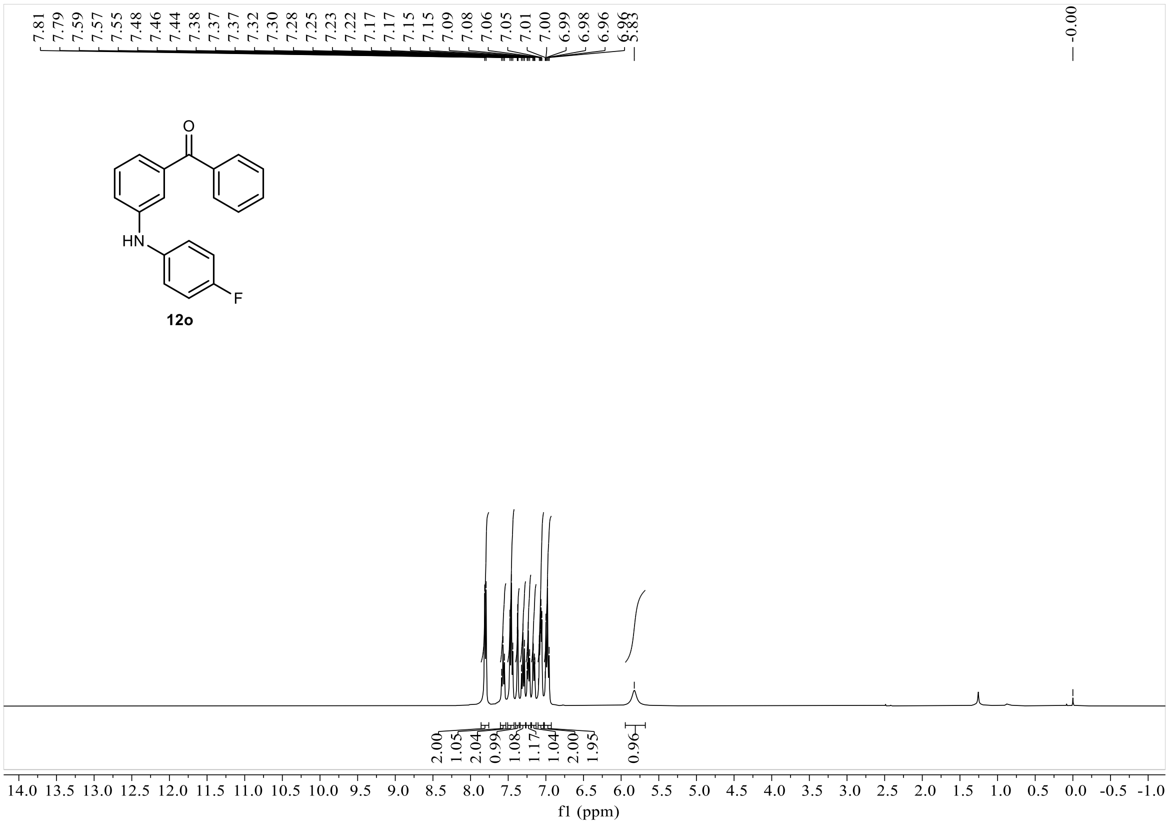

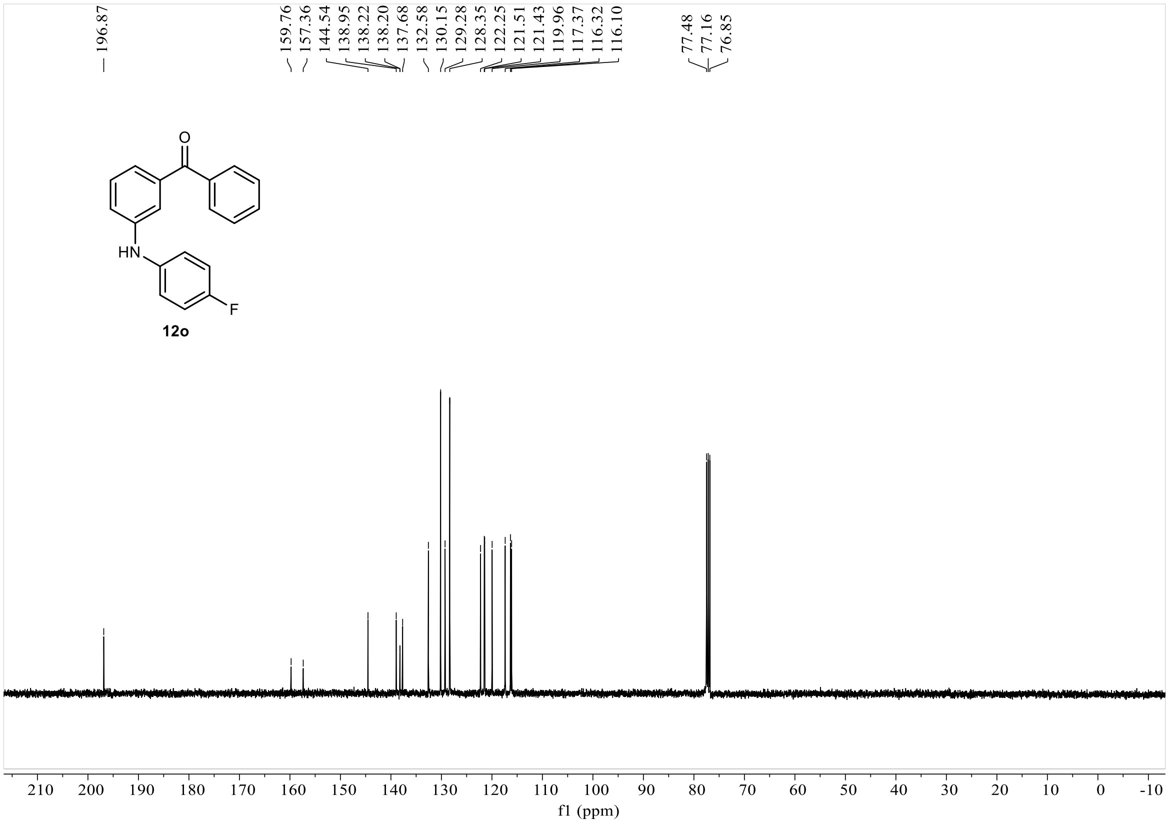

^{19}F NMR (376 MHz, $CDCl_3$) spectrum of **12o**

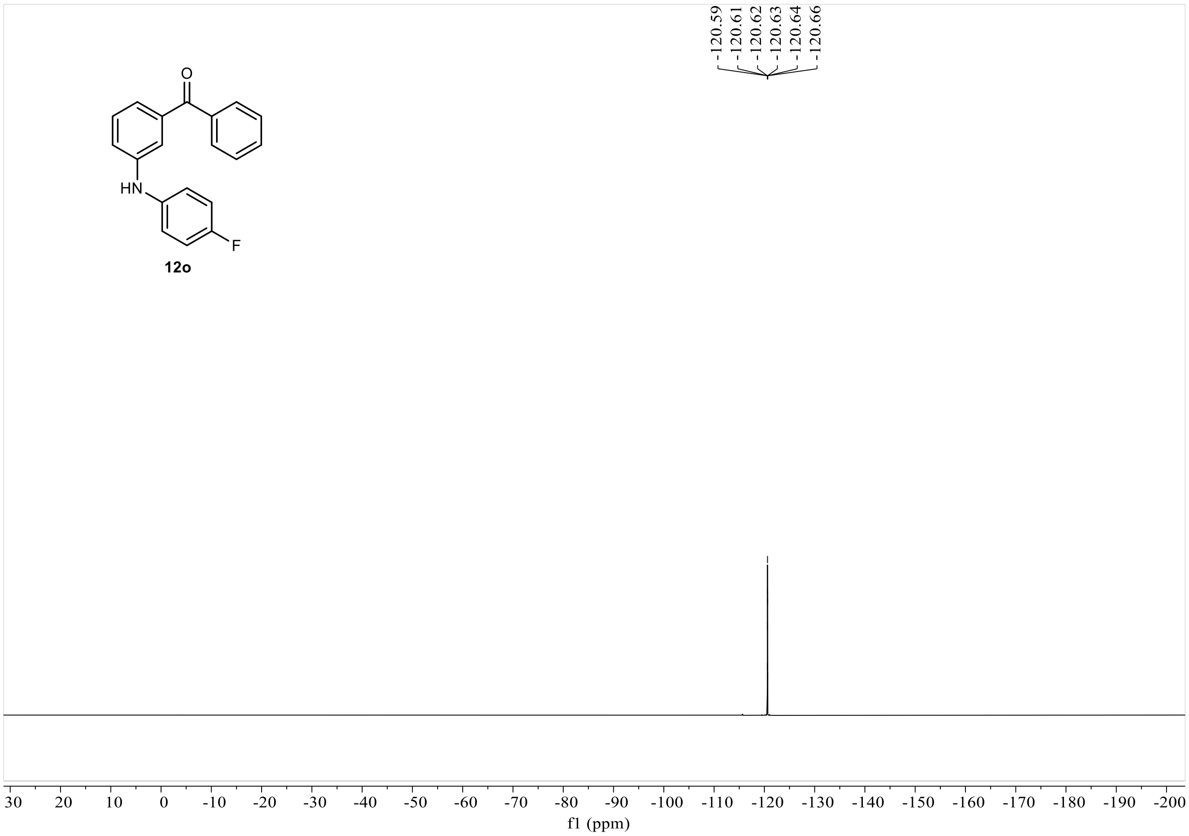

^{1}H NMR (400 MHz, $CDCl_3$) and ^{13}C NMR (101 MHz, $CDCl_3$) spectra of **12p**

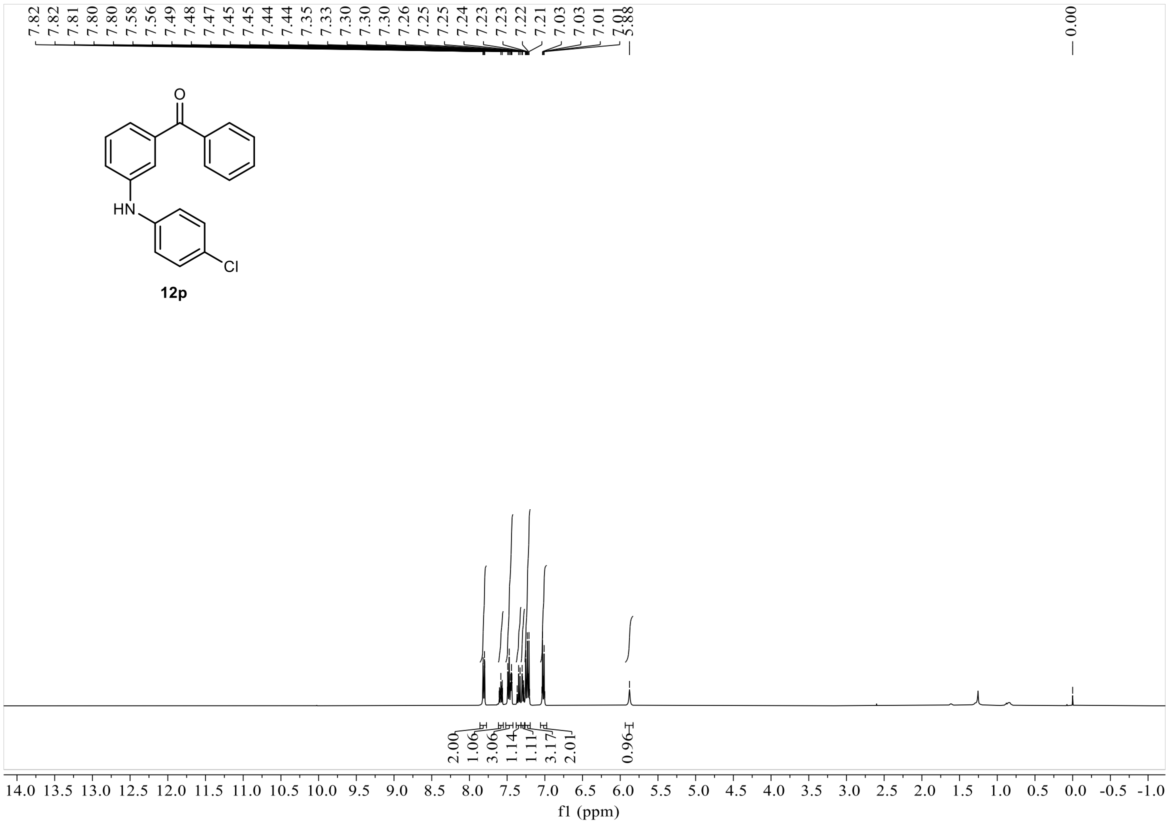

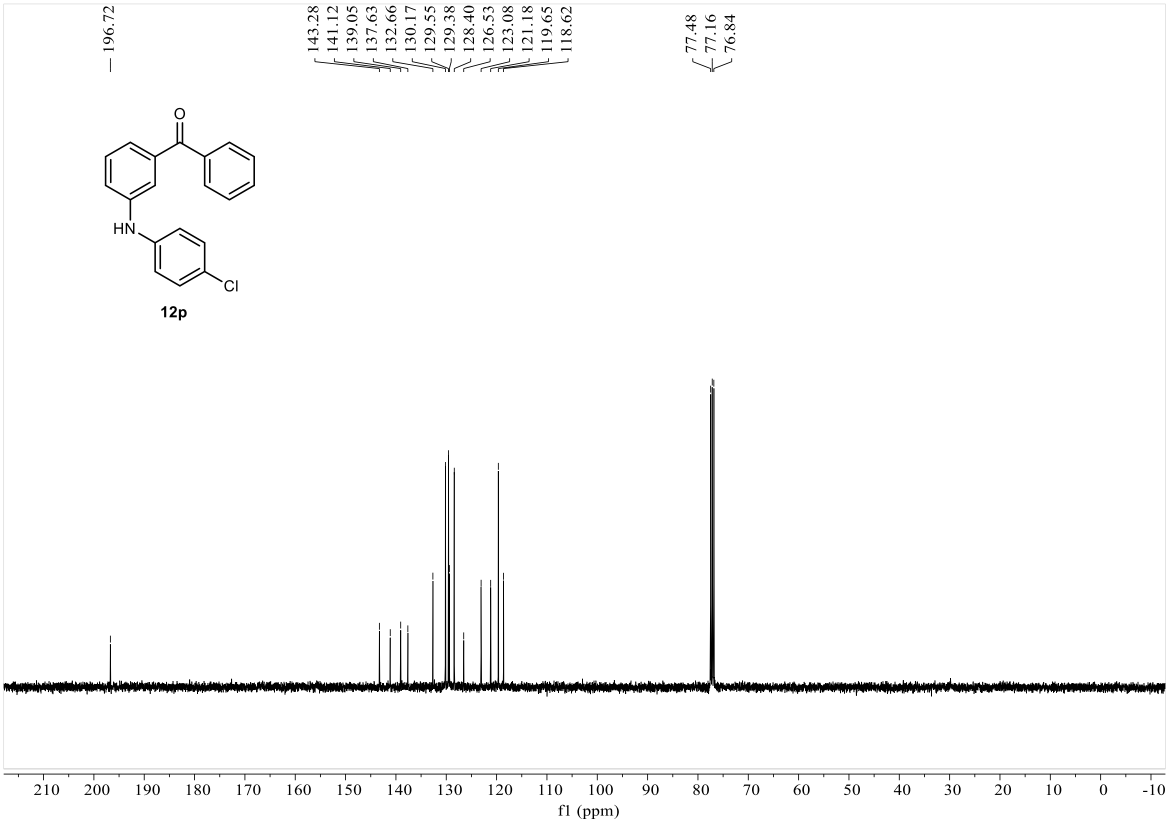

^{1}H NMR (400 MHz, $CDCl_3$) and ^{13}C NMR (101 MHz, $CDCl_3$) spectra of **12q**

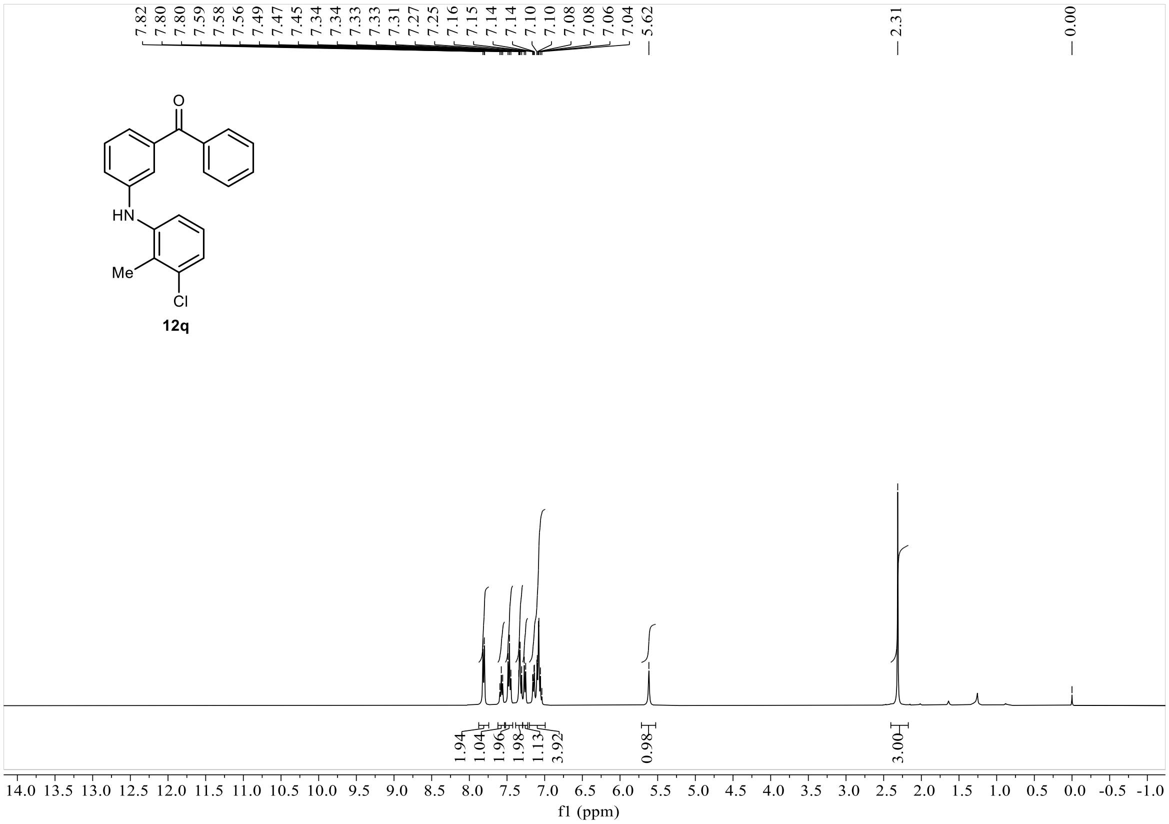

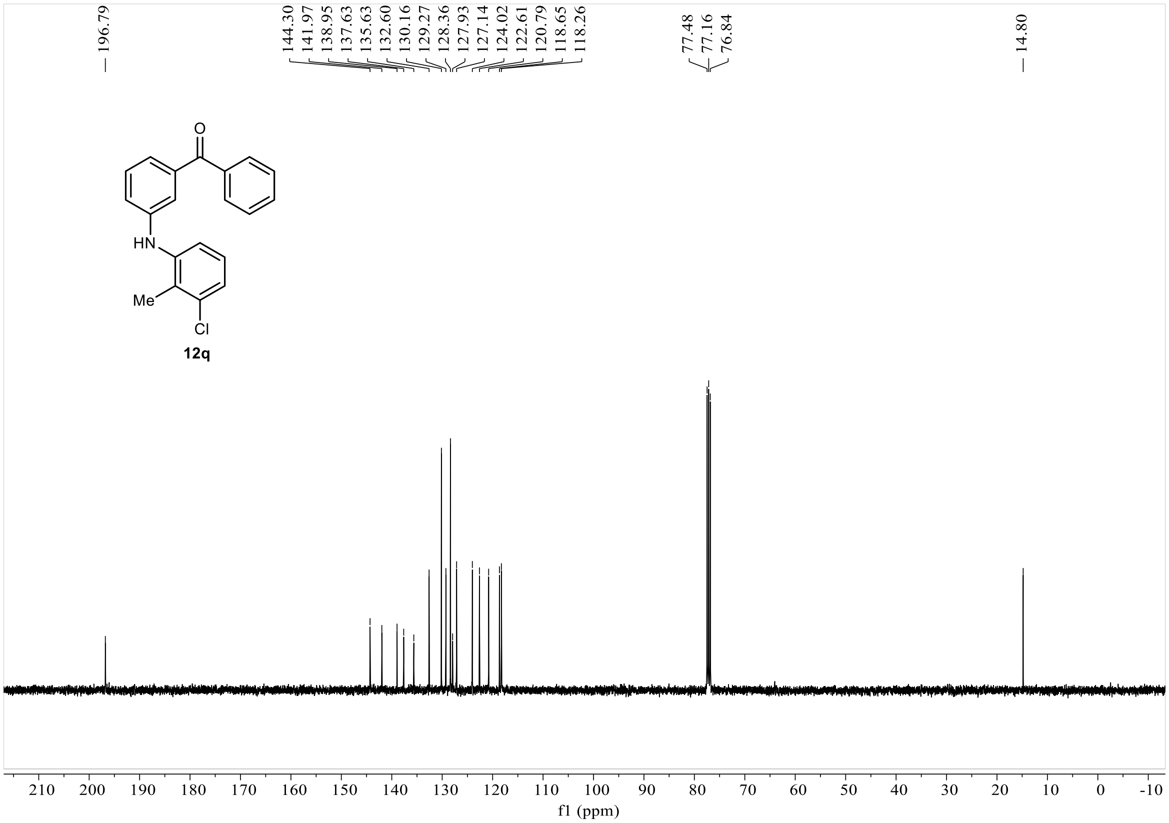

^{1}H NMR (400 MHz, $CDCl_3$) and ^{13}C NMR (101 MHz, $CDCl_3$) spectra of **12r**

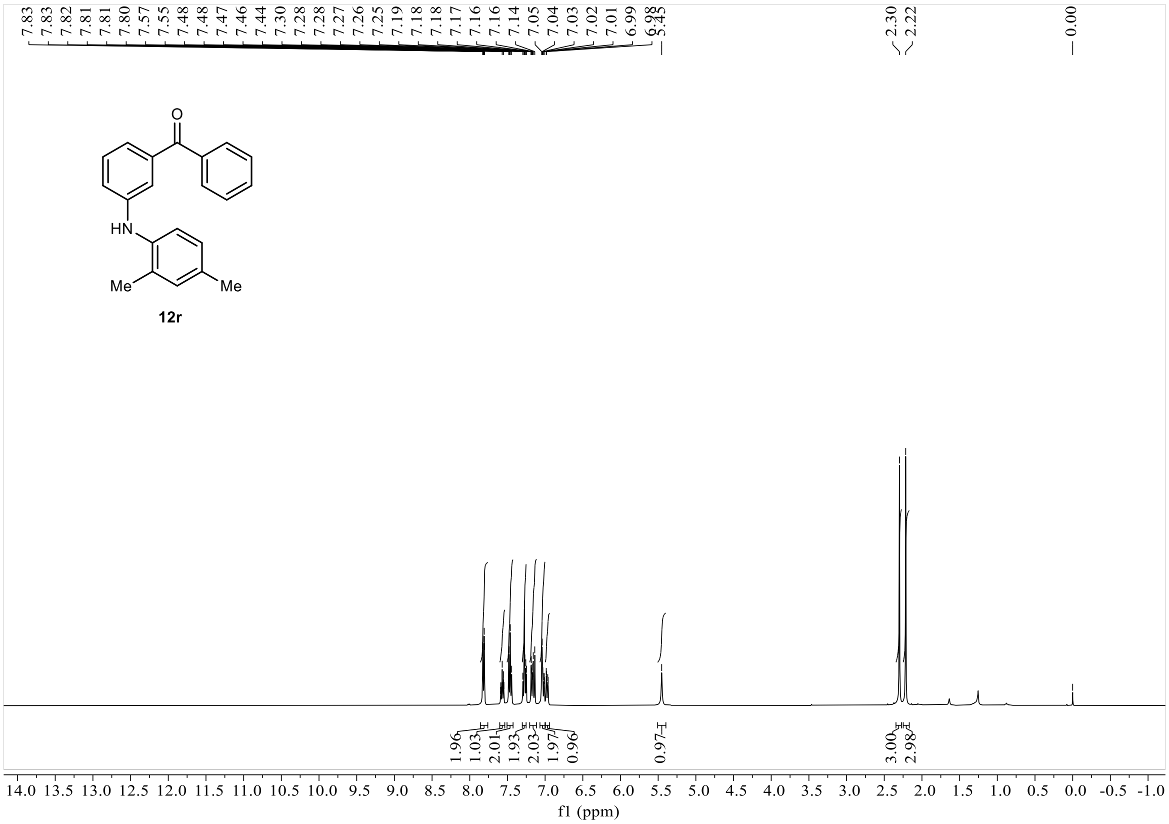

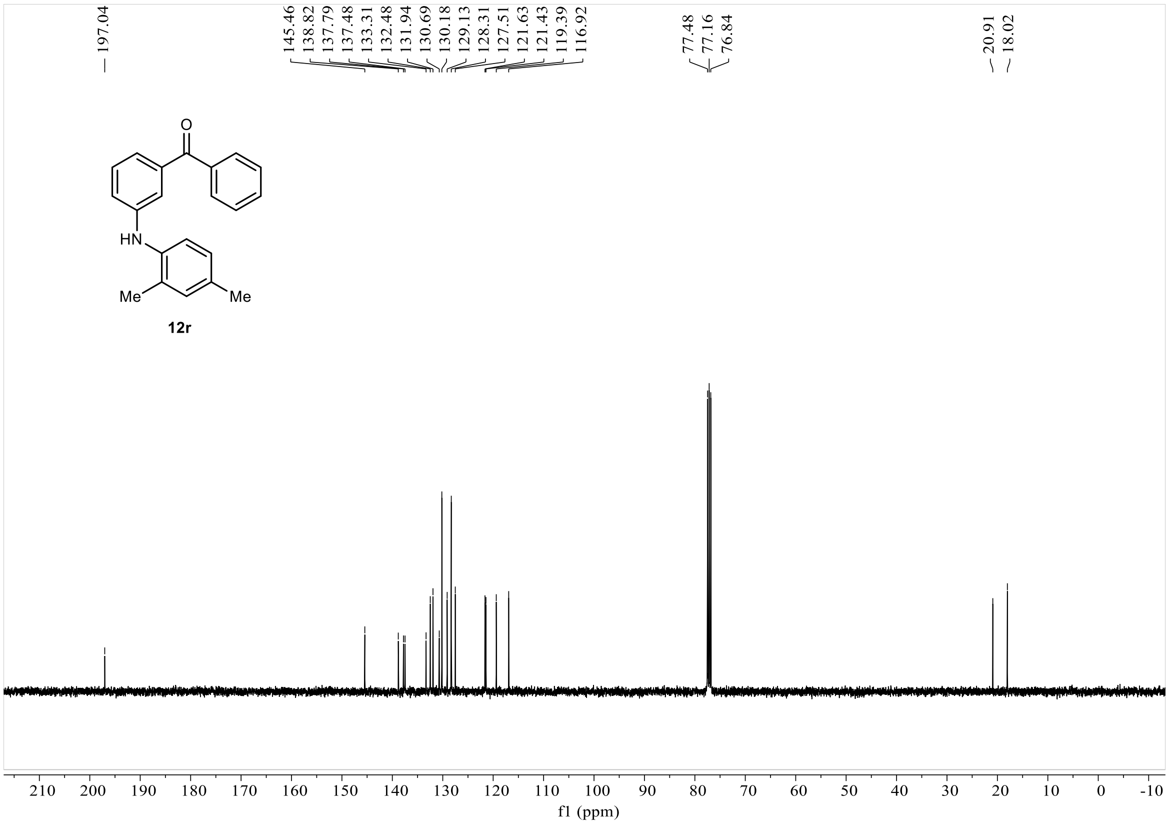

^{1}H NMR (400 MHz, $CDCl_3$) and ^{13}C NMR (101 MHz, $CDCl_3$) spectra of **12s**

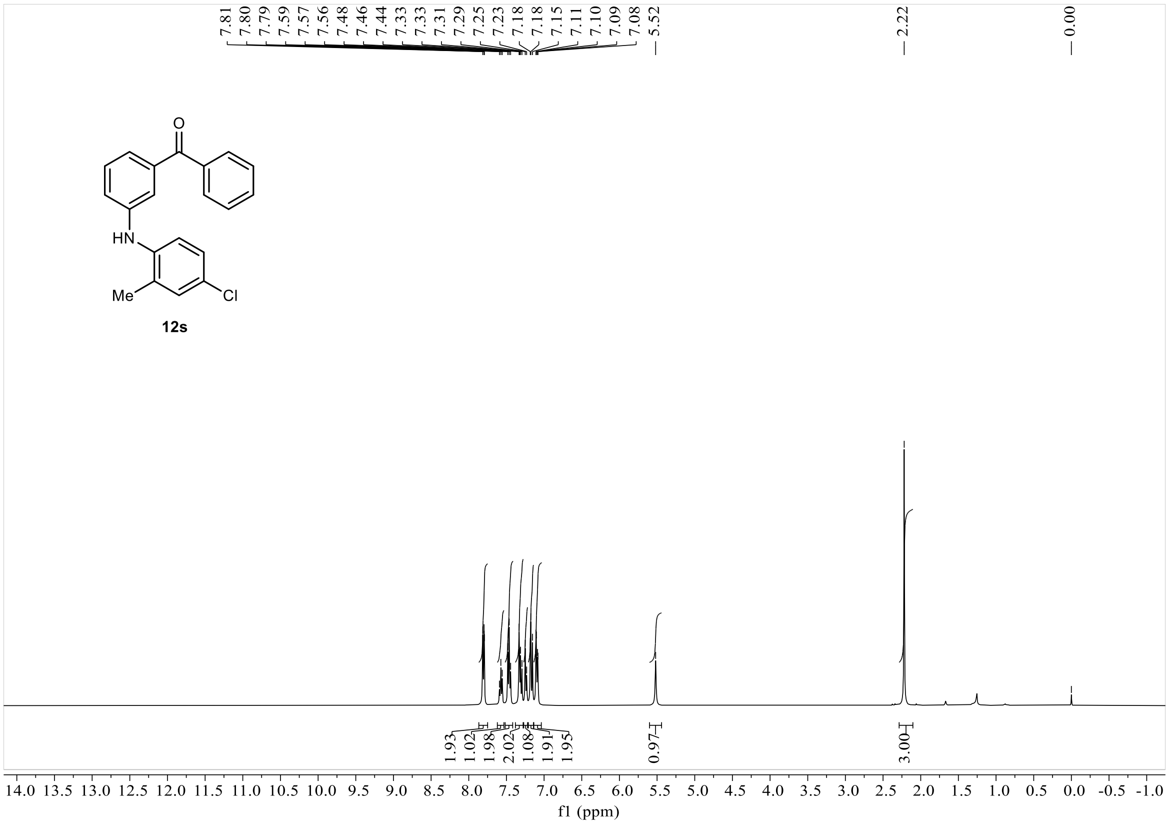

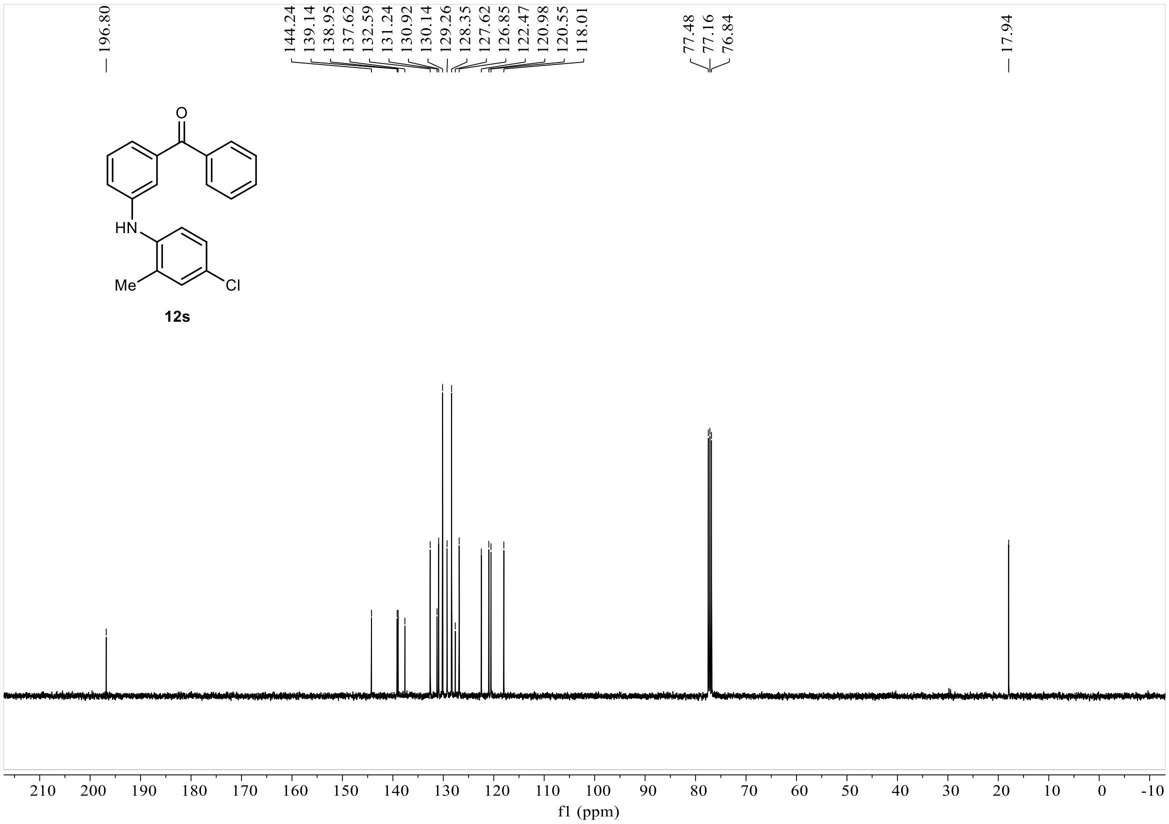

^{1}H NMR (400 MHz, $CDCl_3$) and ^{13}C NMR (101 MHz, $CDCl_3$) spectra of **12t**

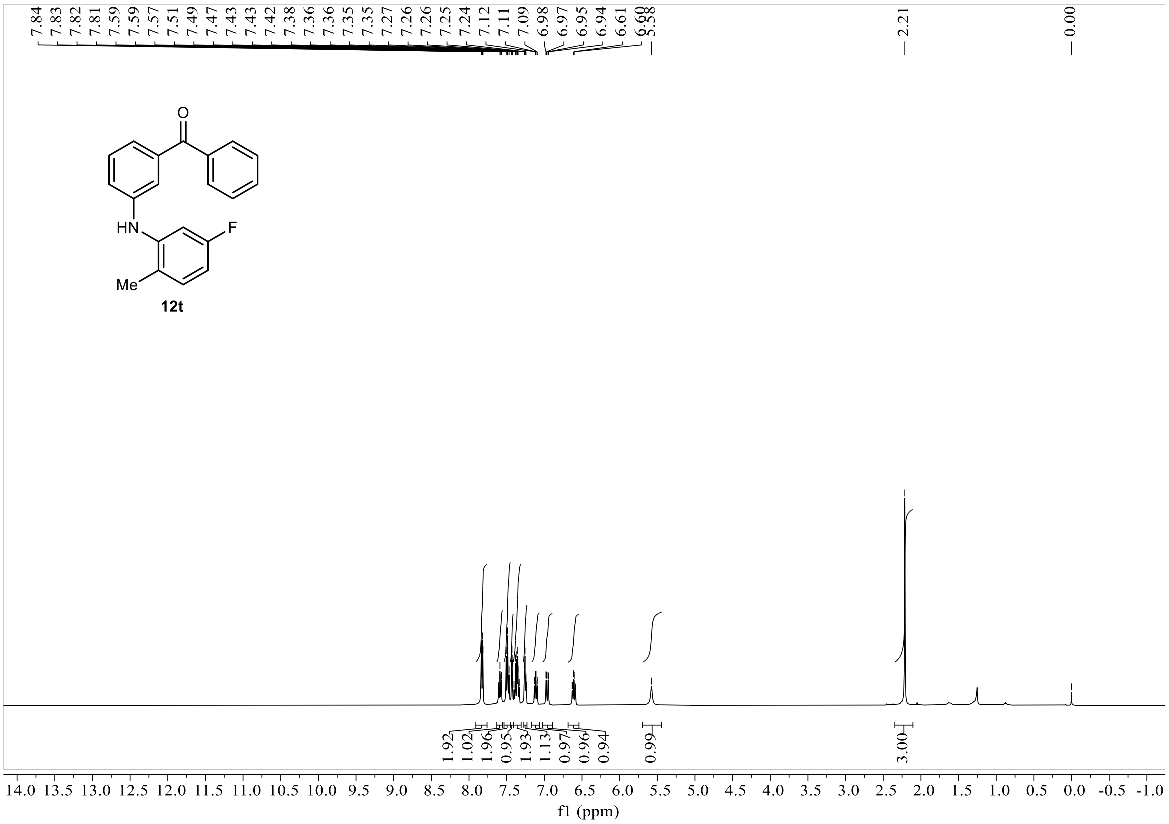

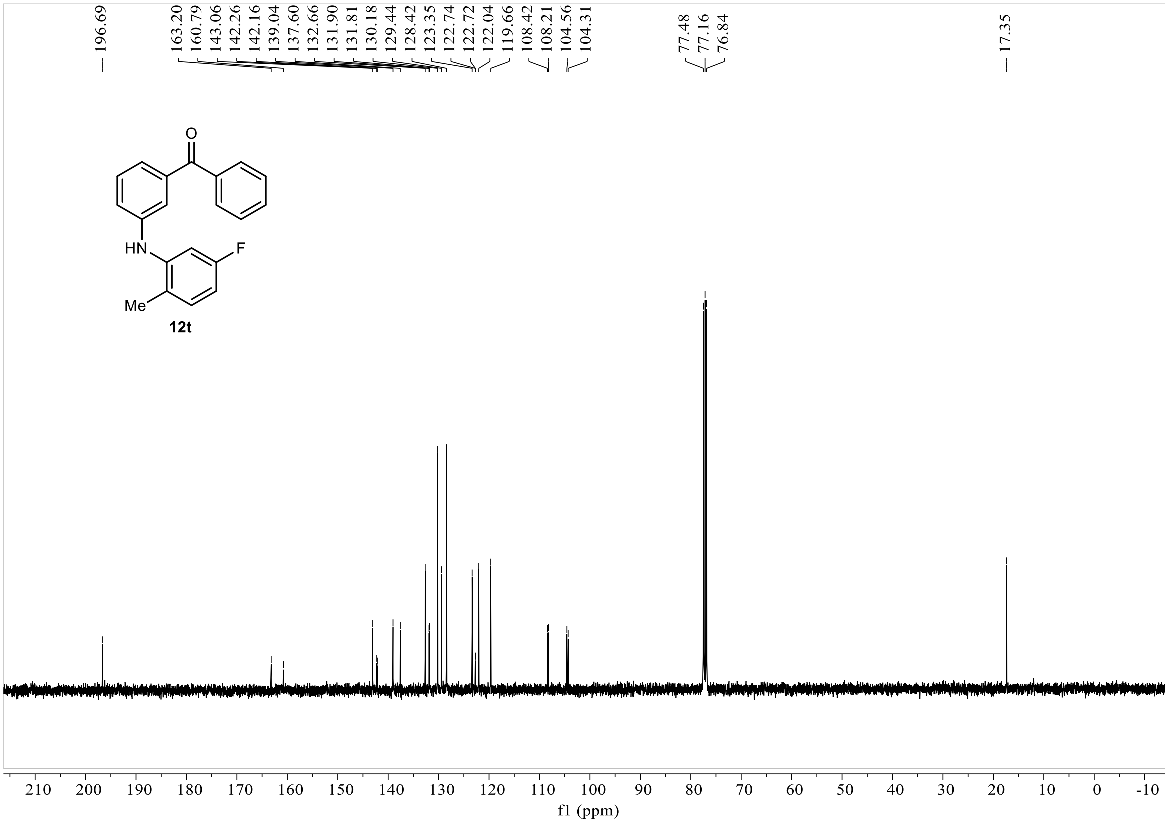

^{19}F NMR (376 MHz, $CDCl_3$) spectrum of **12t**

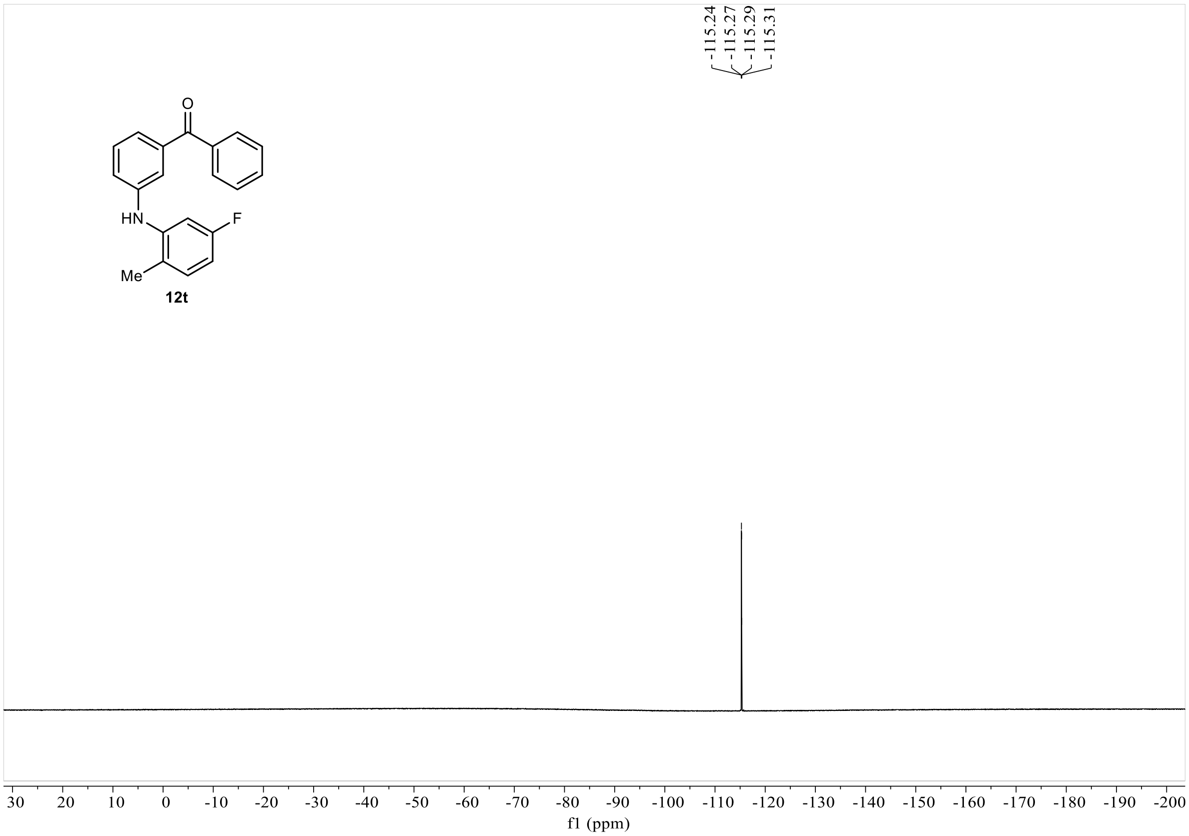

^{1}H NMR (400 MHz, $CDCl_3$) and ^{13}C NMR (101 MHz, $CDCl_3$) spectra of **12u**

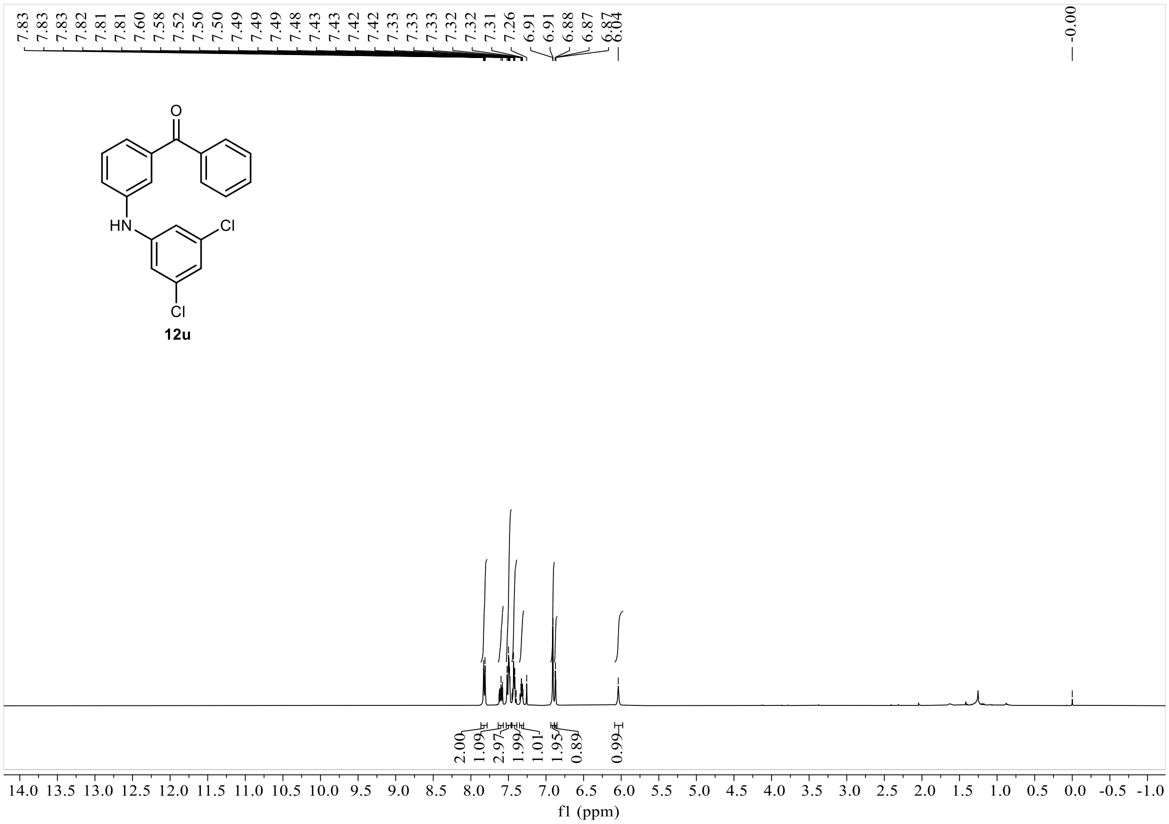

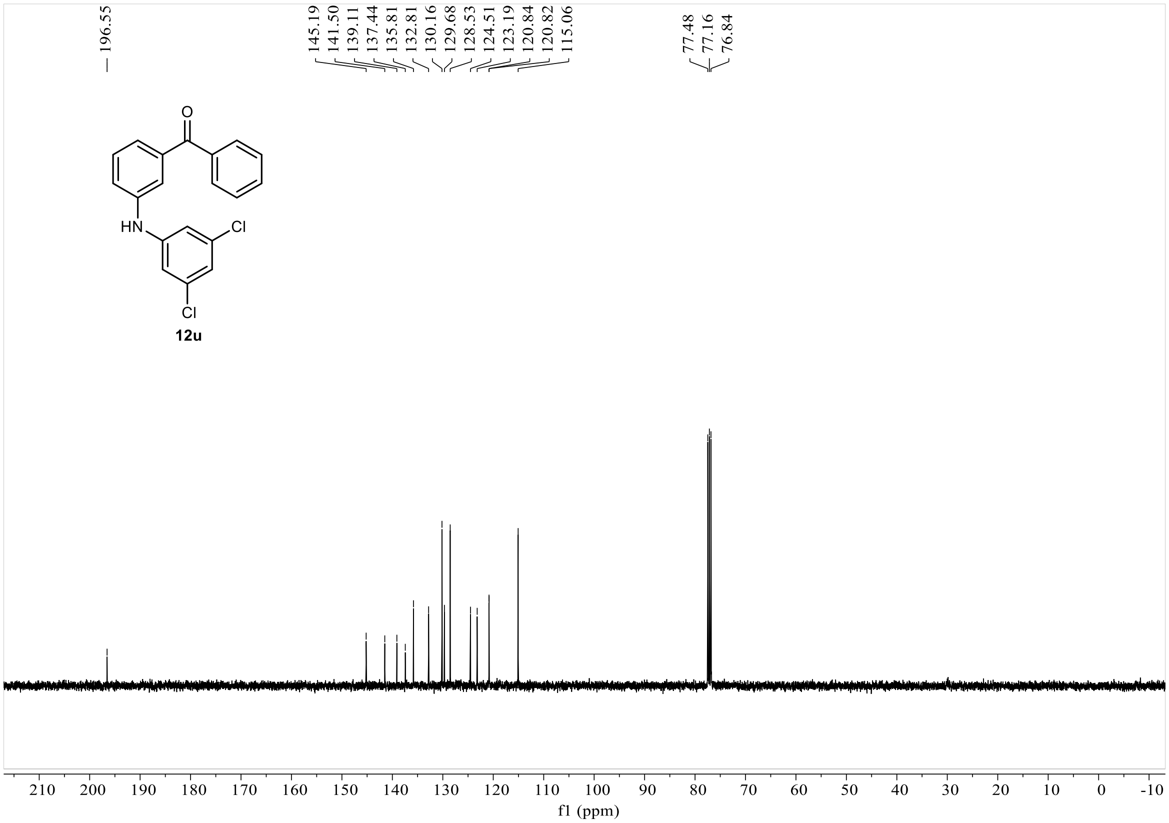

^{1}H NMR (400 MHz, $CDCl_3$) and ^{13}C NMR (101 MHz, $CDCl_3$) spectra of **12v**

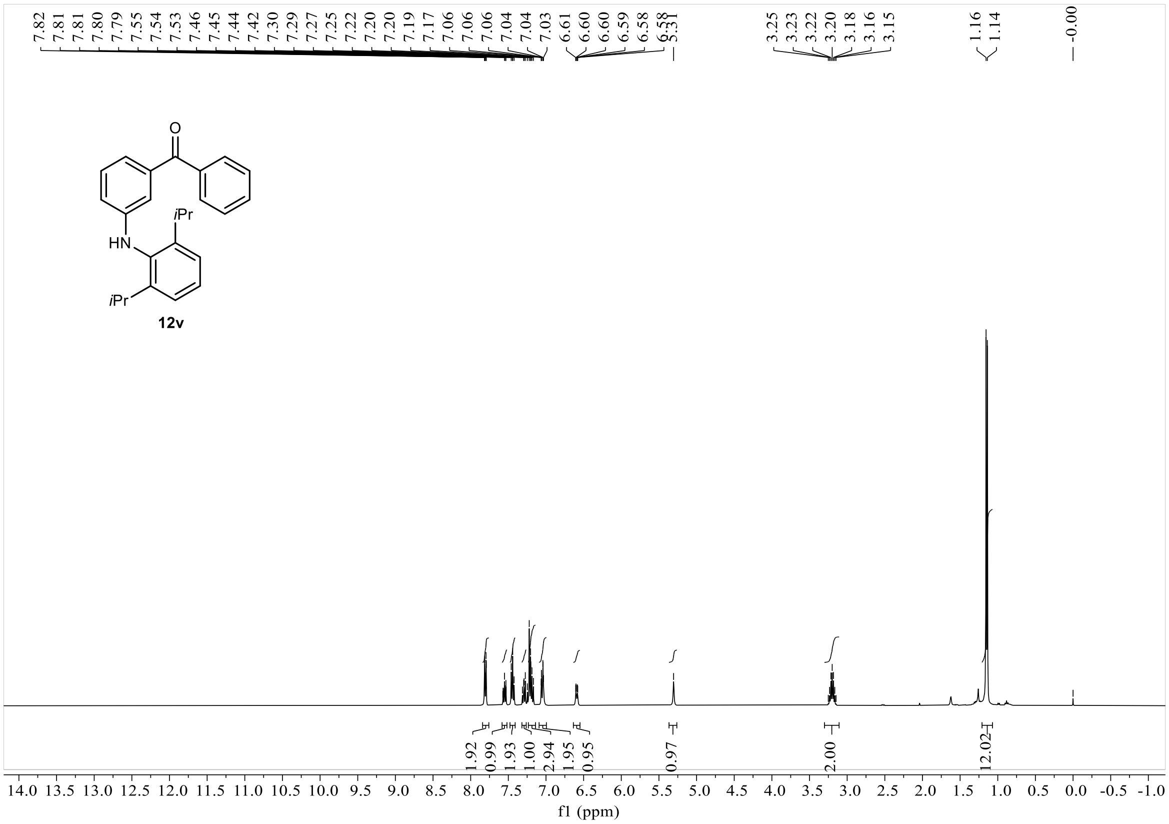

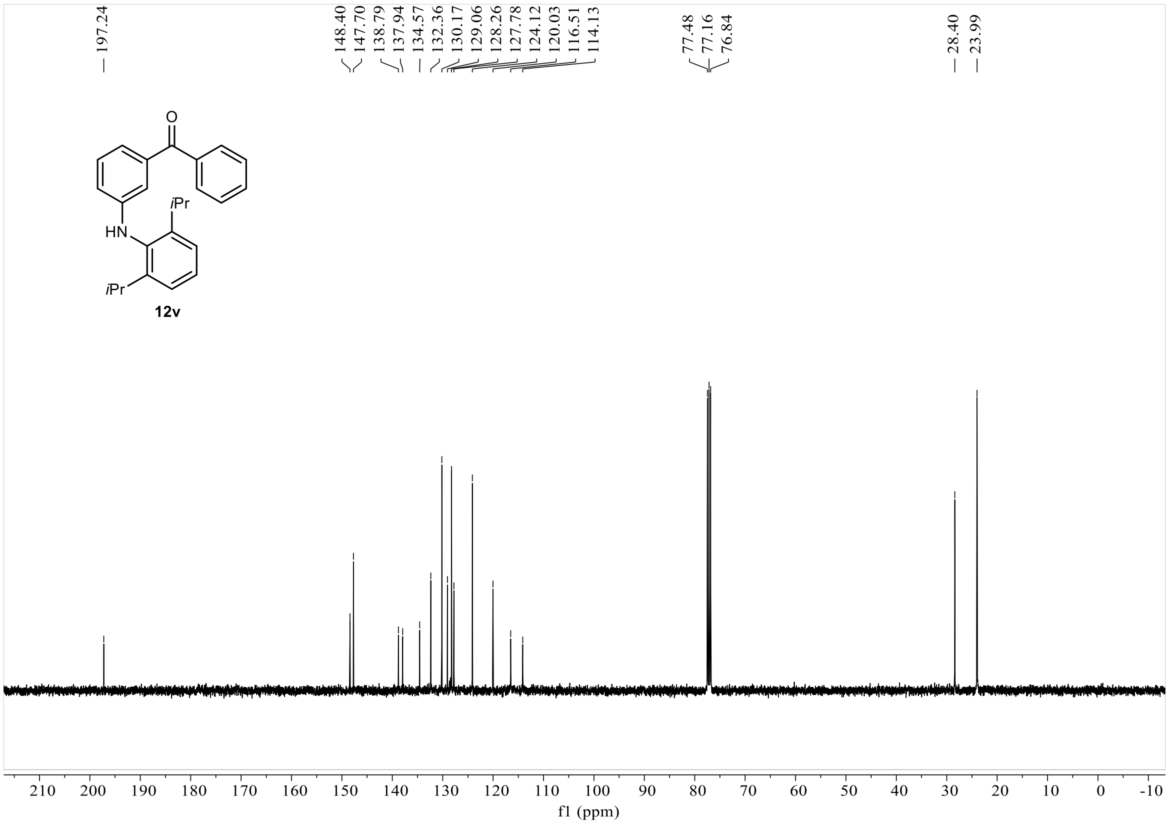

^{1}H NMR (400 MHz, $CDCl_3$) and ^{13}C NMR (101 MHz, $CDCl_3$) spectra of **12w**

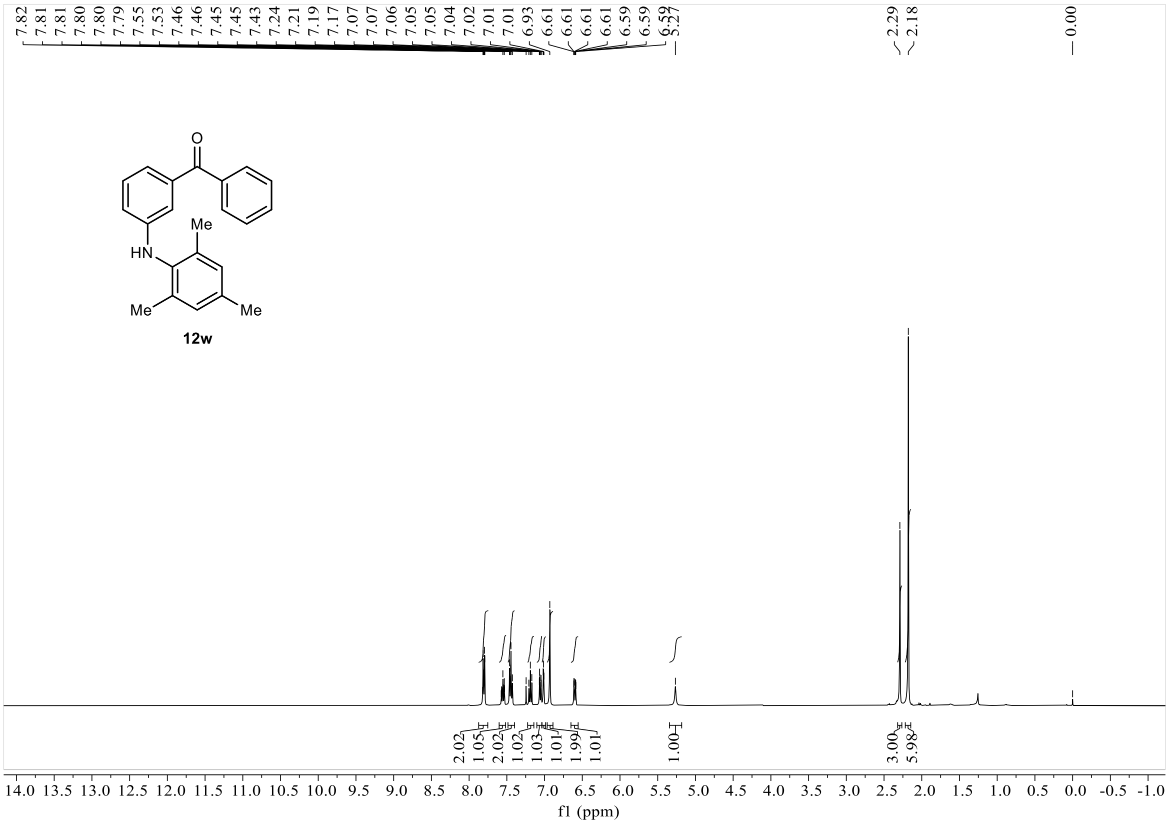

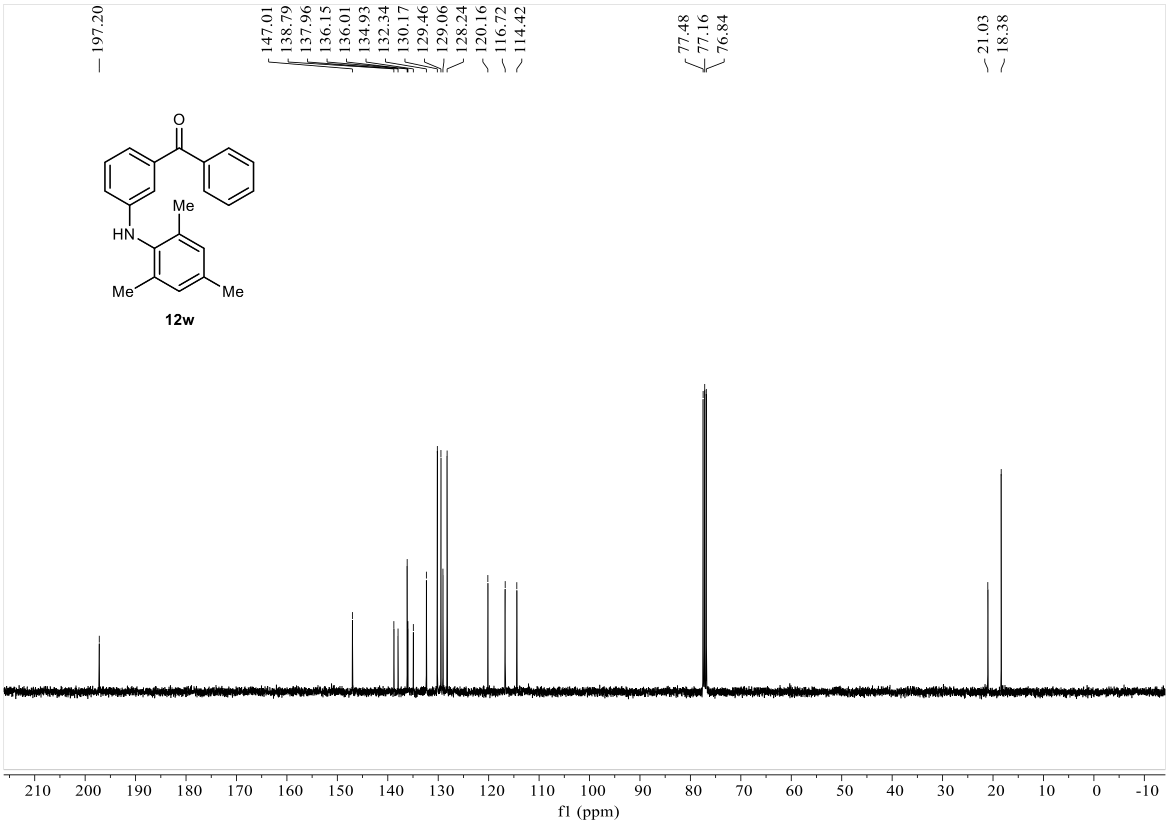

^1H NMR (400 MHz, $CDCl_3$) and ^{13}C NMR (101 MHz, $CDCl_3$) spectra of **12x**

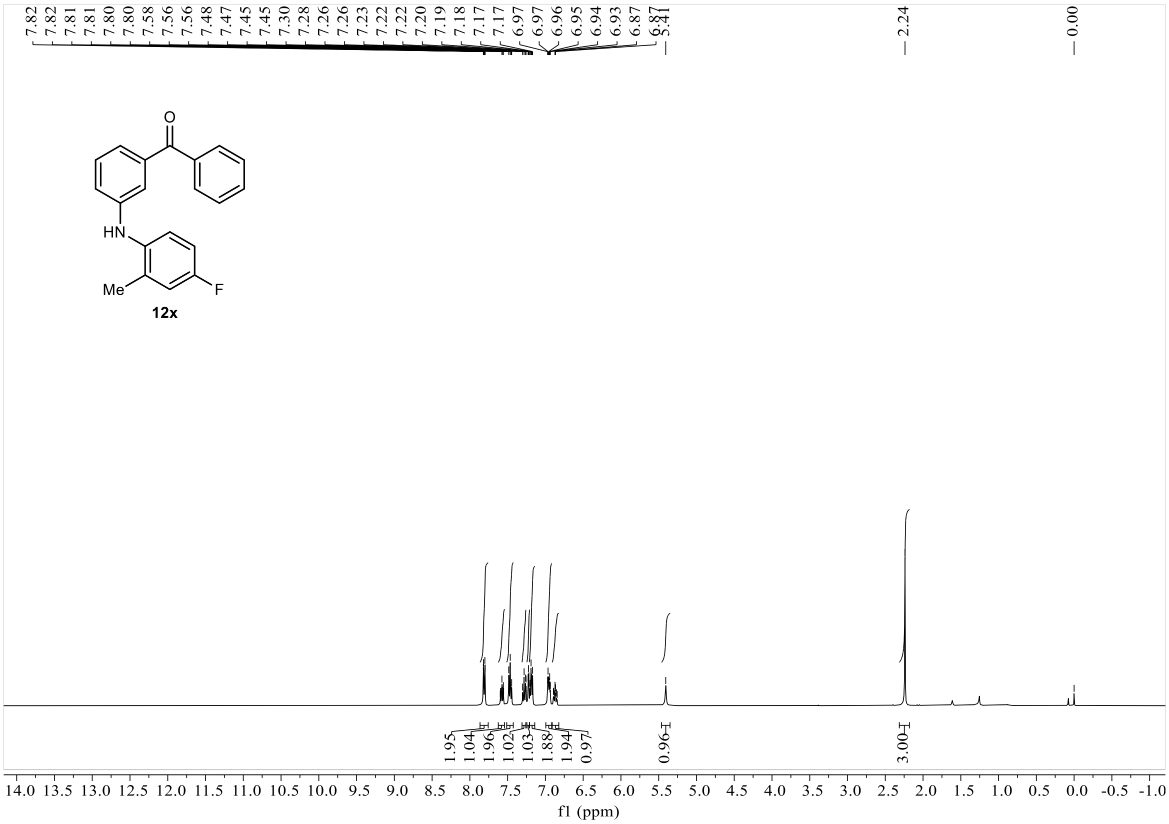

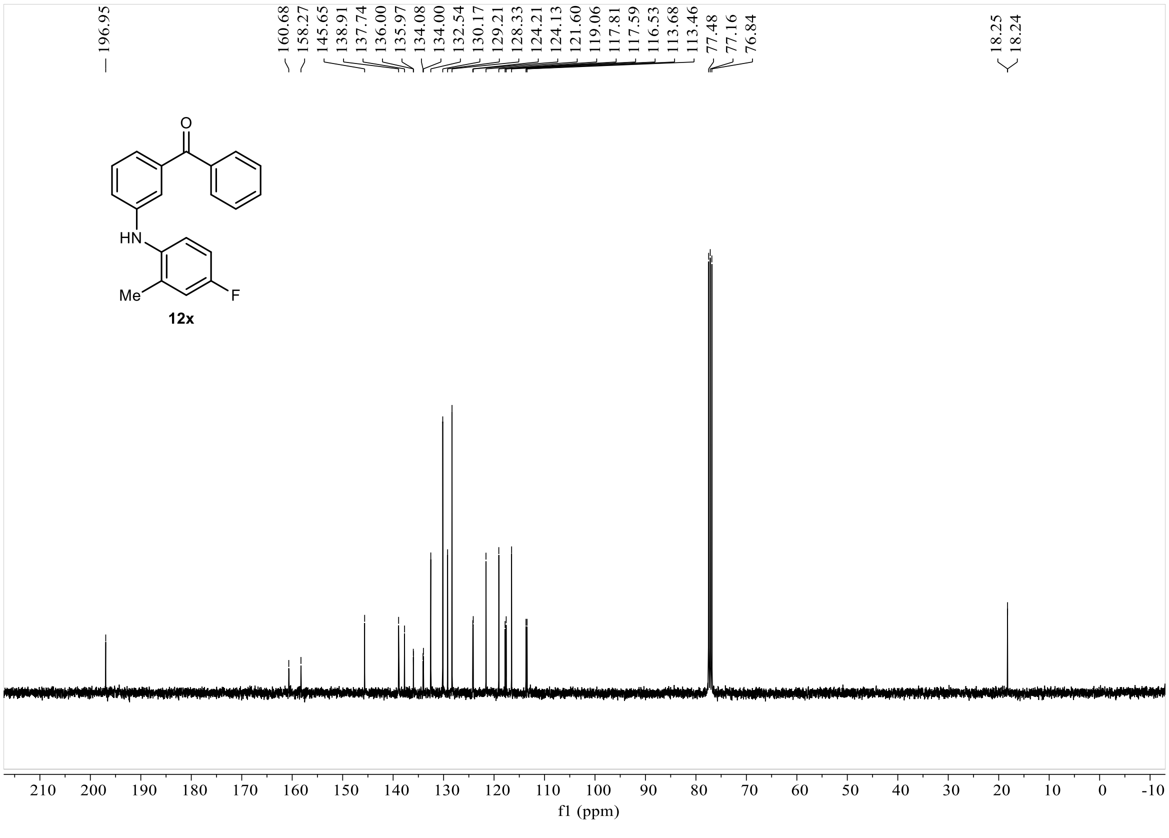

^{19}F NMR (376 MHz, $CDCl_3$) spectrum of **12x**

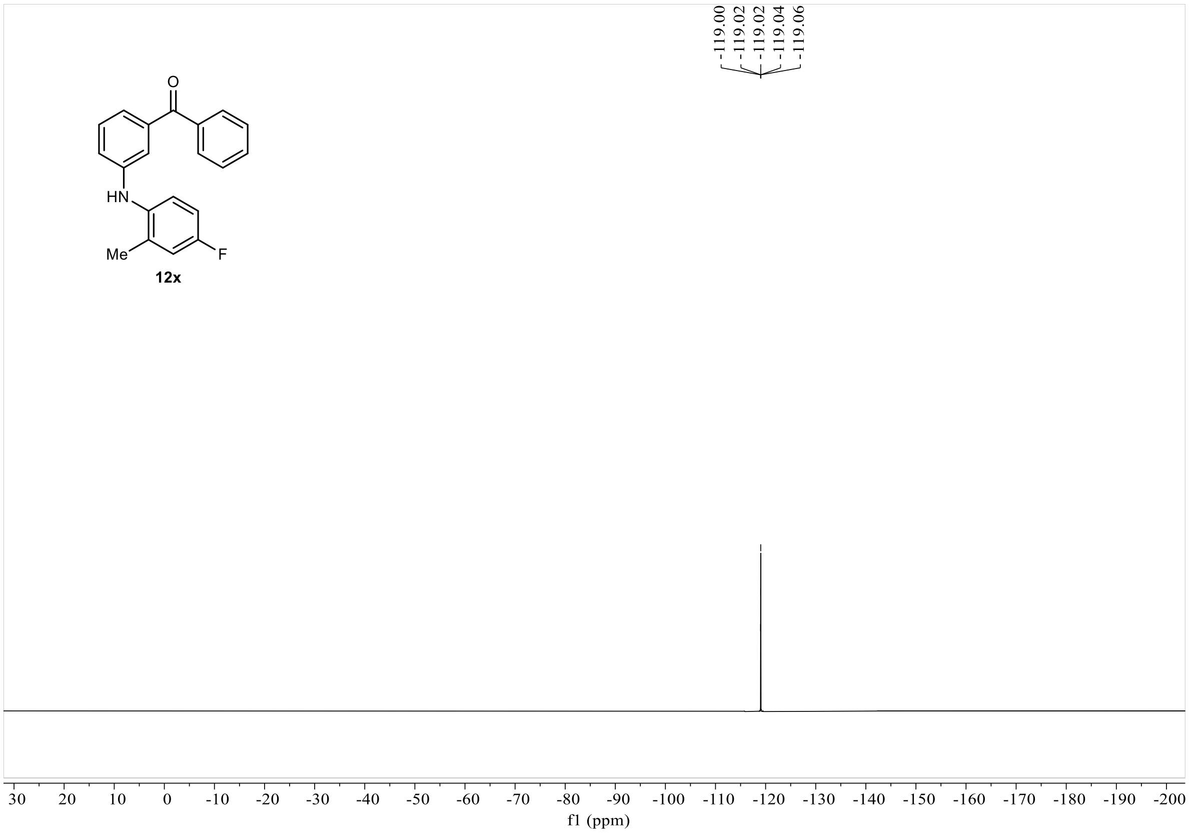

^{1}H NMR (400 MHz, $CDCl_3$) and ^{13}C NMR (101 MHz, $CDCl_3$) spectra of **12y**

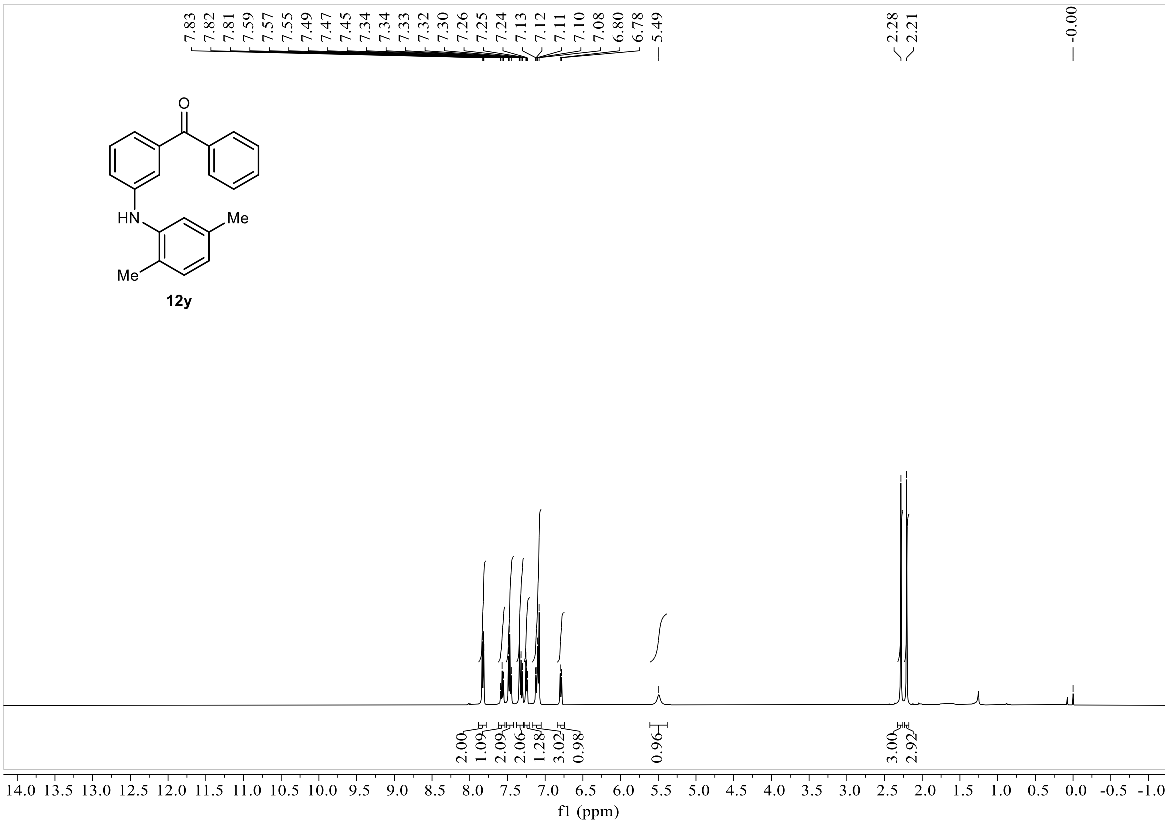

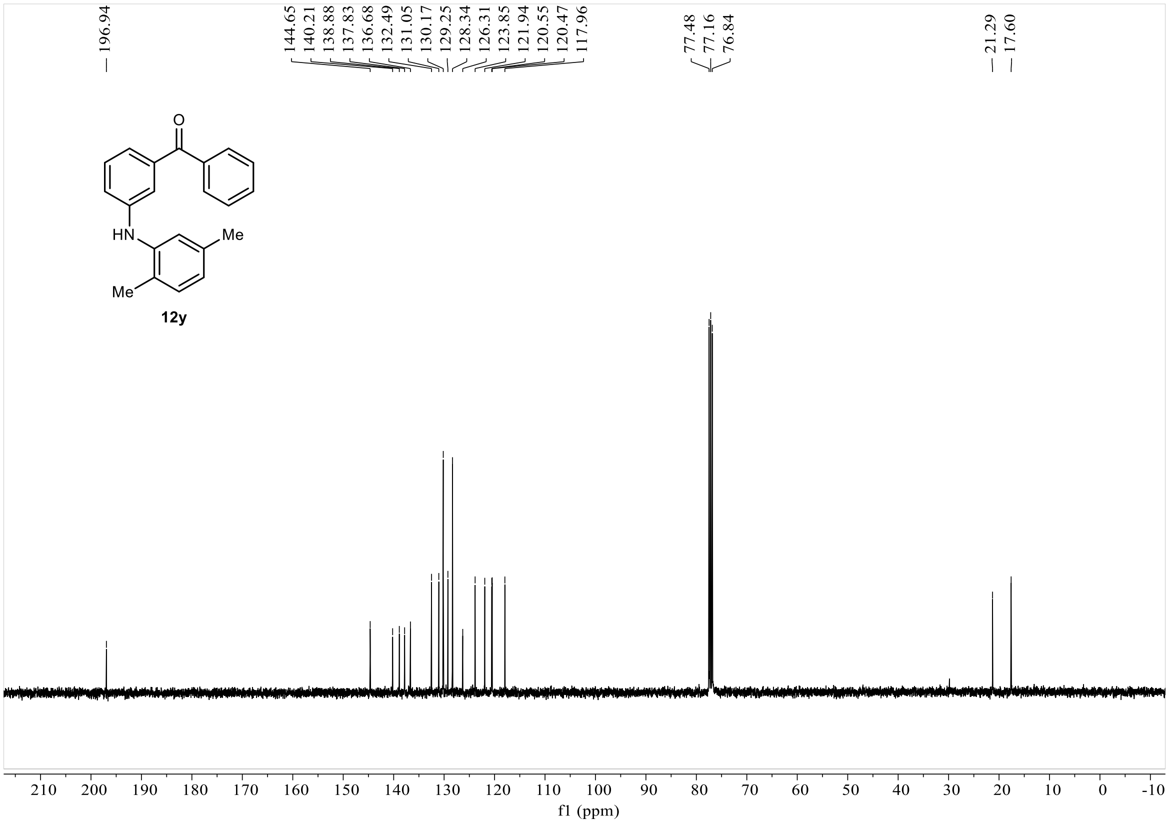

^{1}H NMR (400 MHz, $CDCl_3$) and ^{13}C NMR (101 MHz, $CDCl_3$) spectra of **13b**

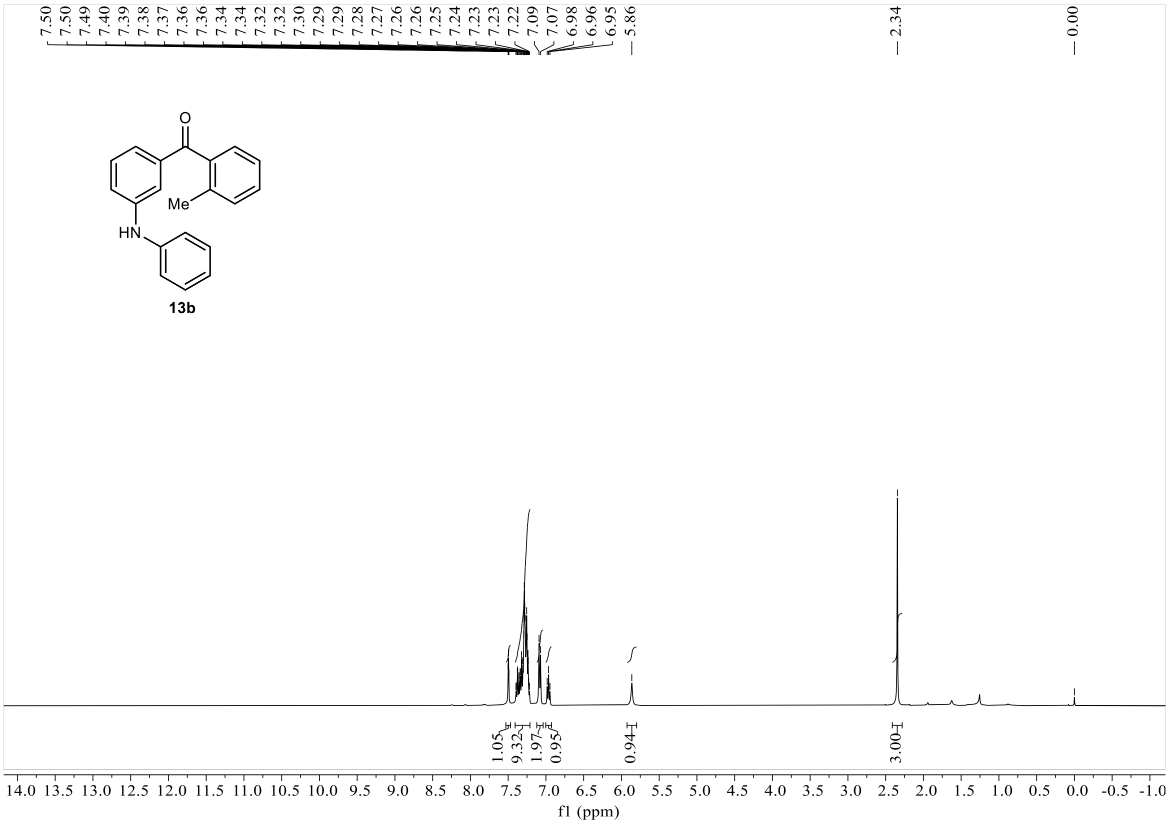

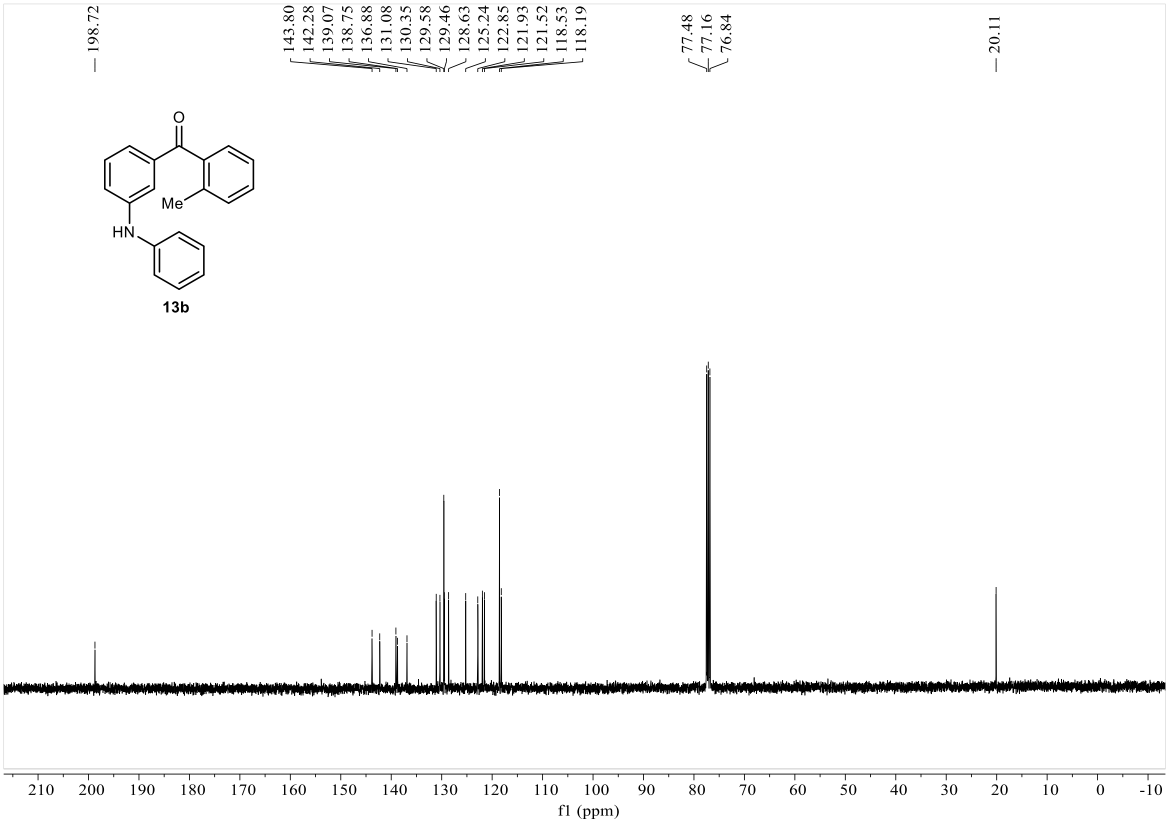

^1H NMR (400 MHz, $CDCl_3$) and ^{13}C NMR (101 MHz, $CDCl_3$) spectra of **13c**

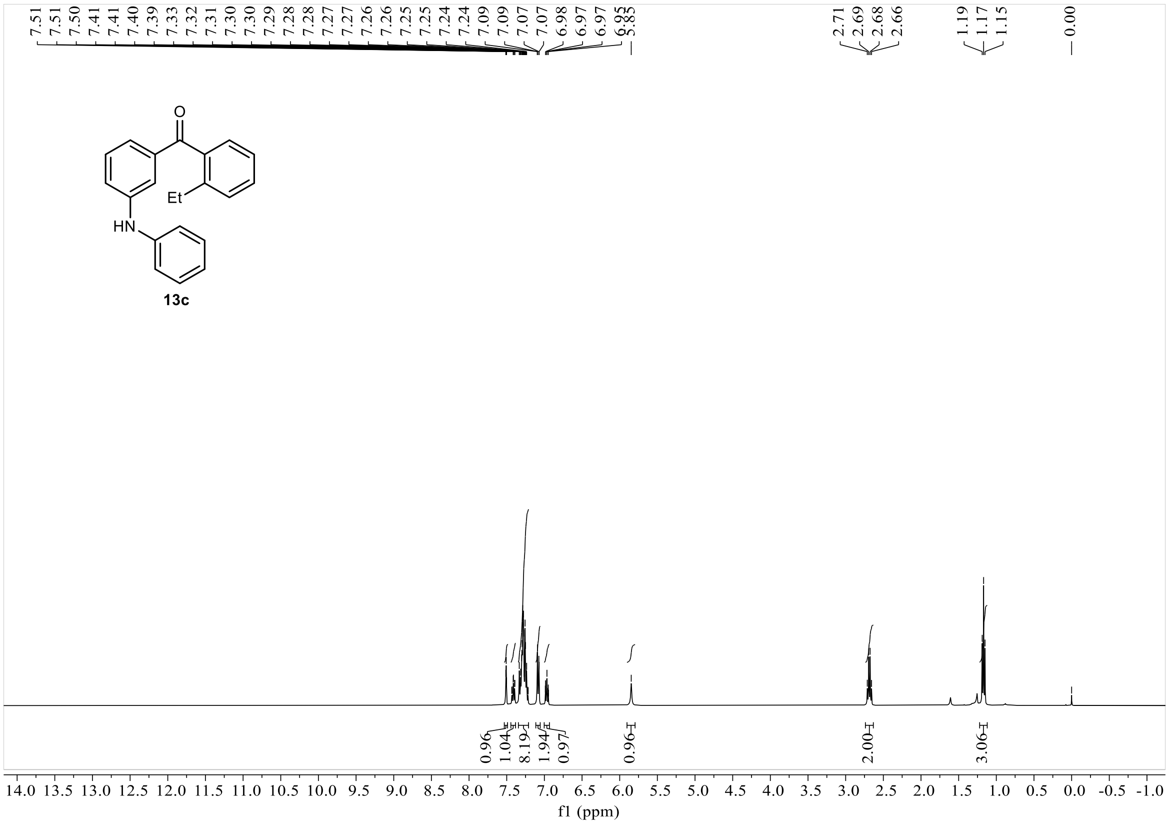

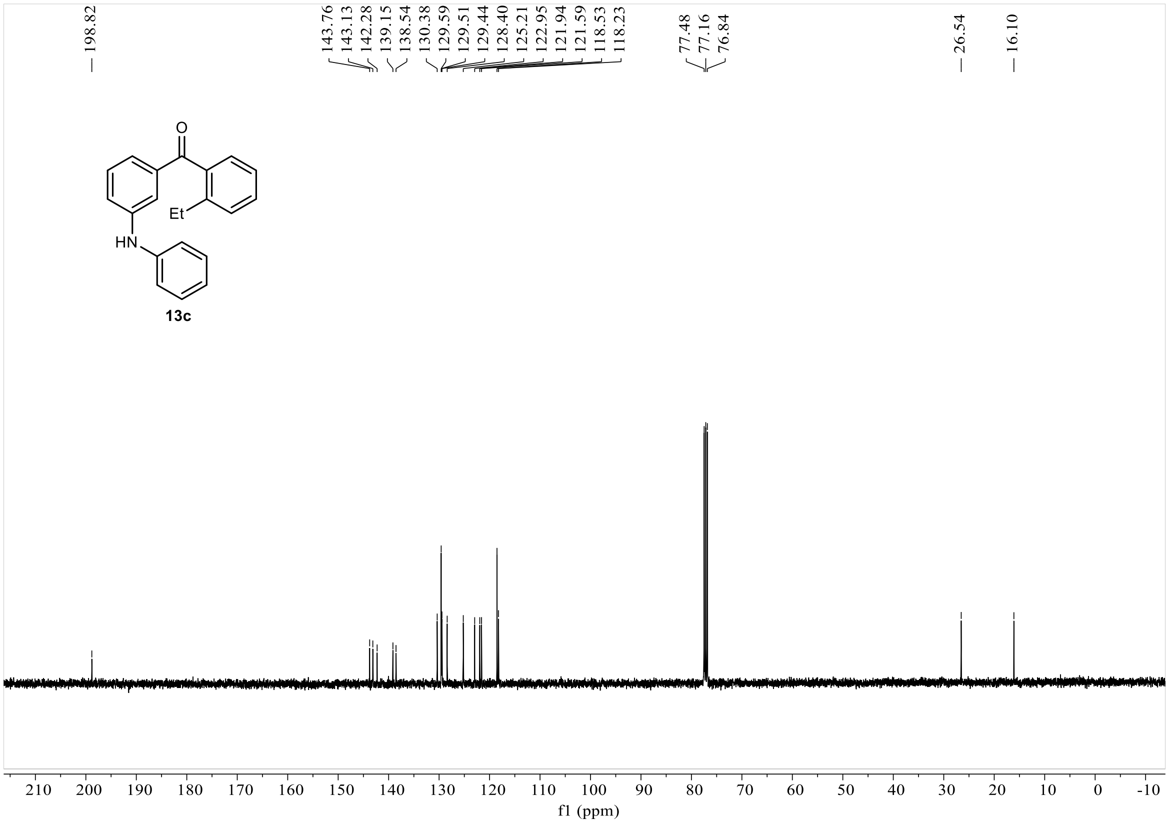

^{1}H NMR (400 MHz, $CDCl_3$) and ^{13}C NMR (101 MHz, $CDCl_3$) spectra of **13h**

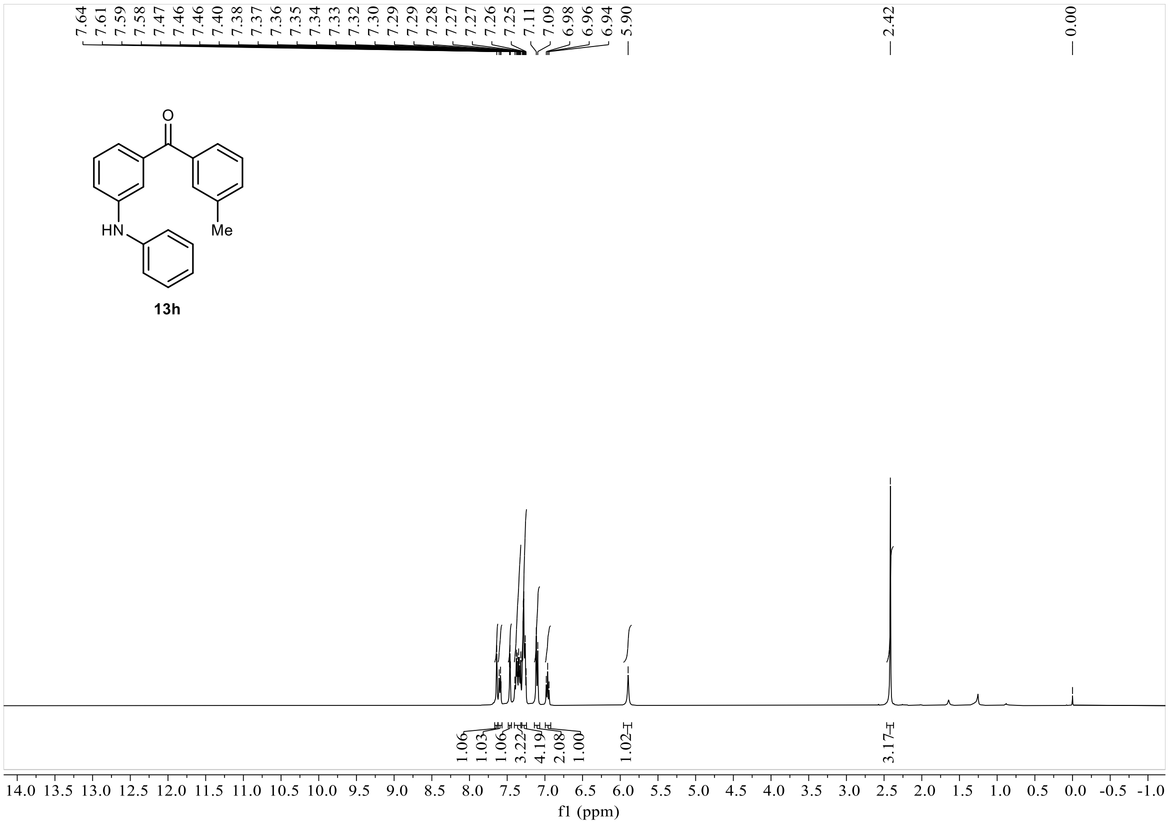

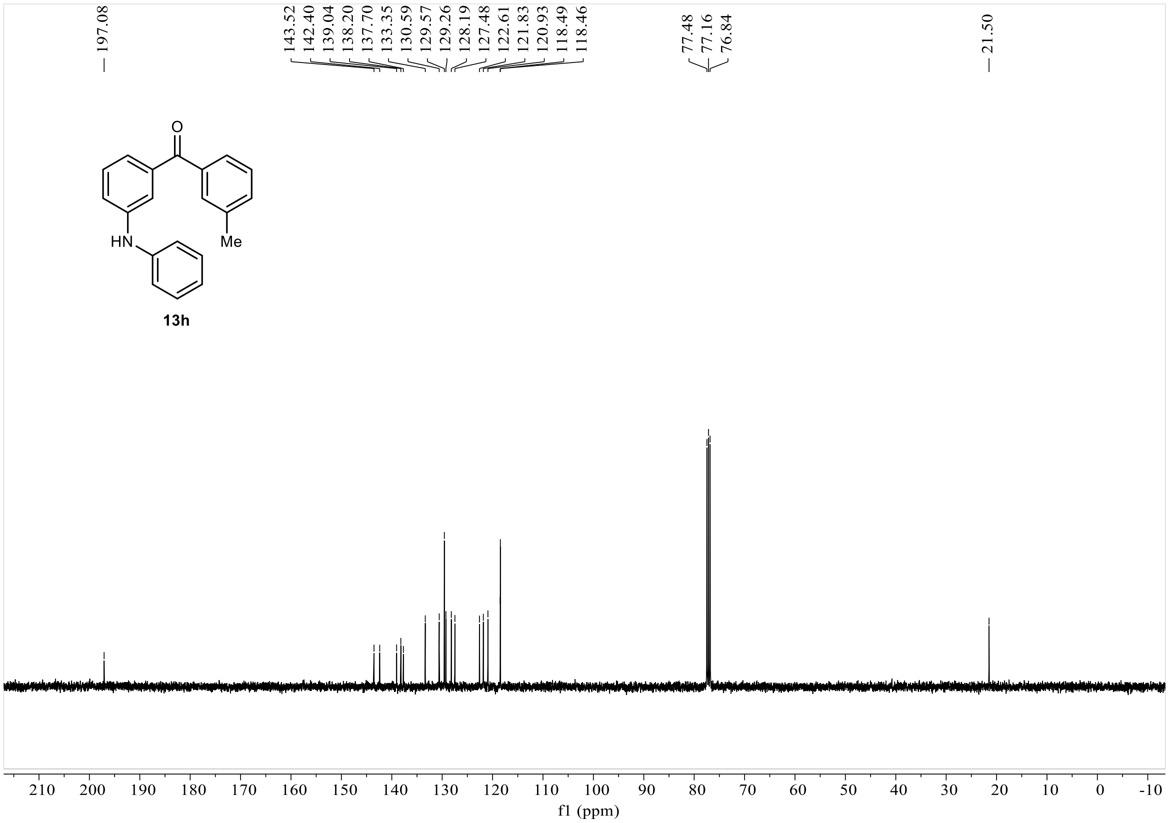

^{1}H NMR (400 MHz, $CDCl_3$) and ^{13}C NMR (101 MHz, $CDCl_3$) spectra of **13n**

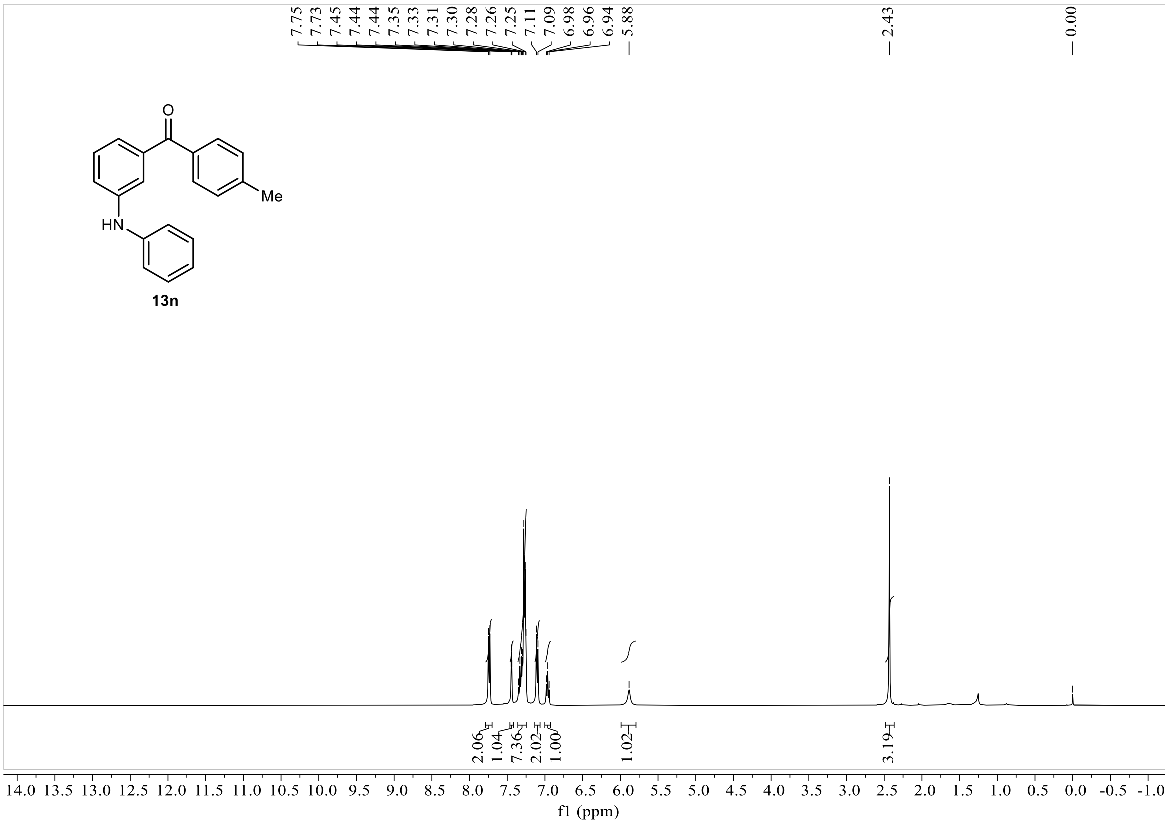

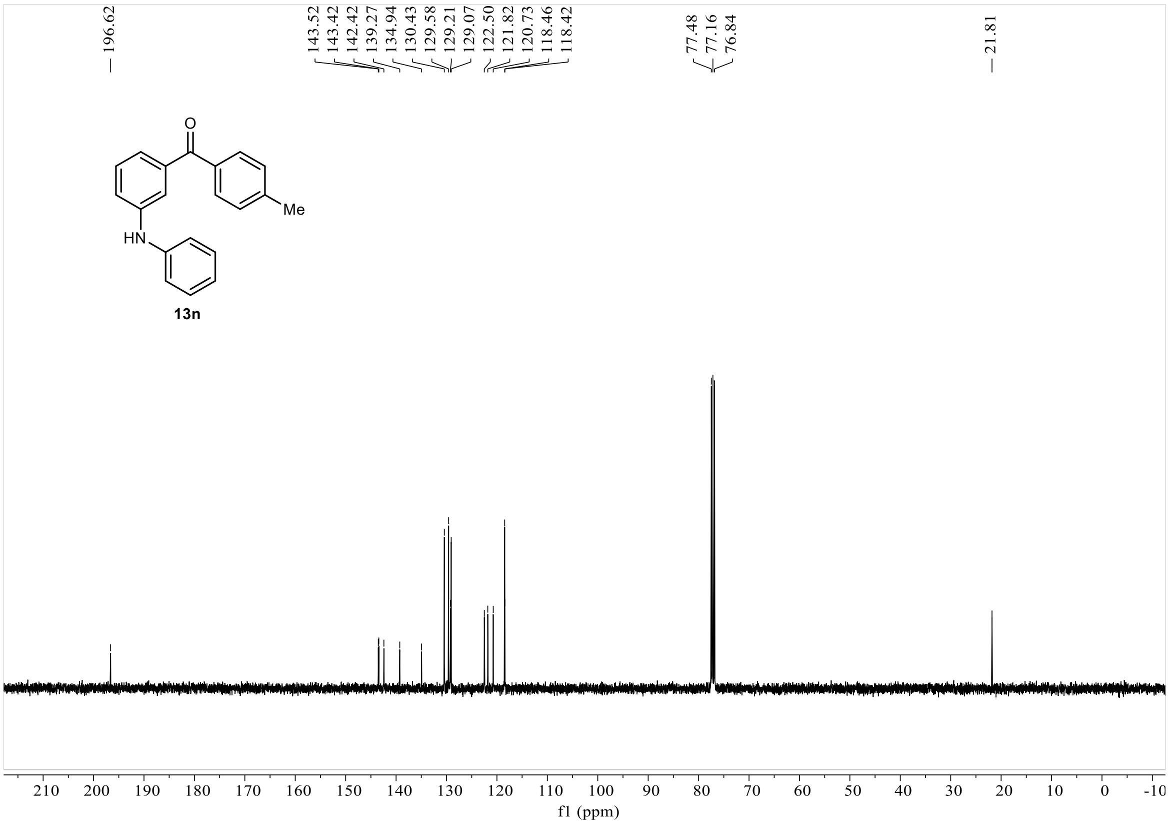

^{1}H NMR (400 MHz, $CDCl_3$) and ^{13}C NMR (101 MHz, $CDCl_3$) spectra of **13o**

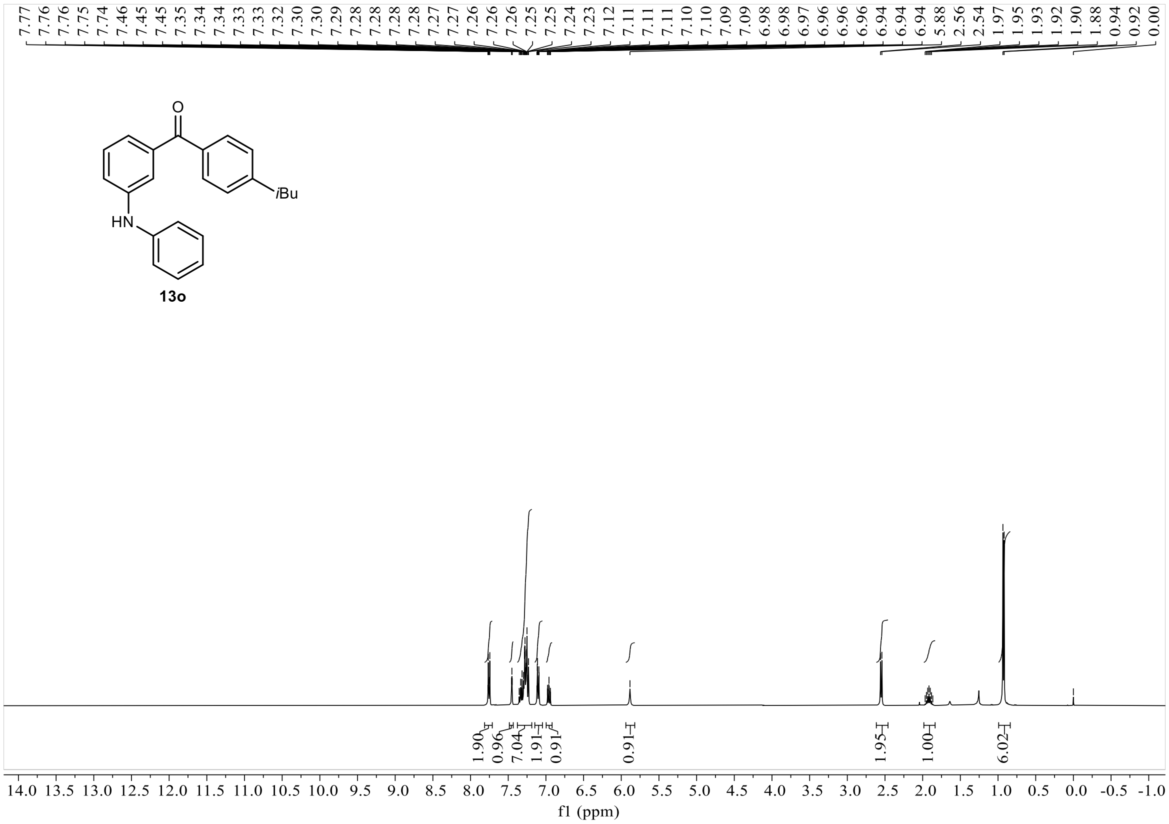

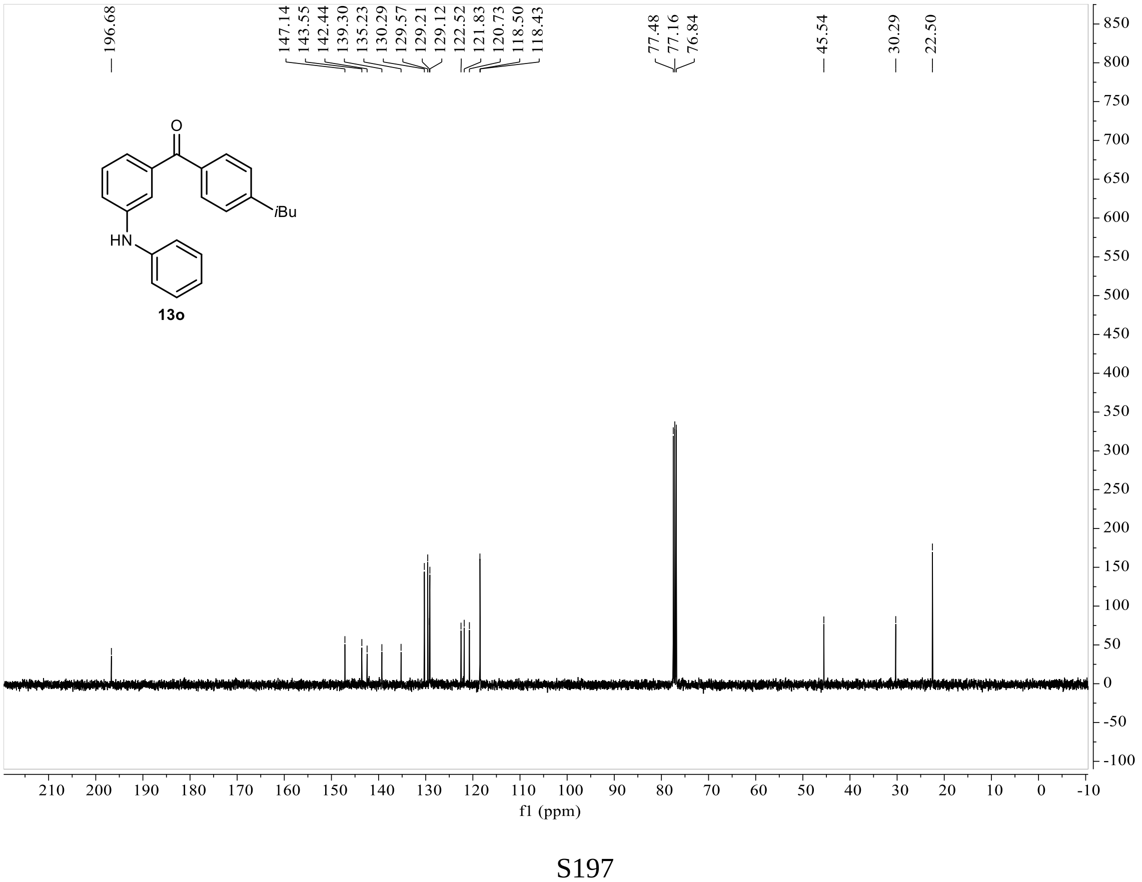

^{1}H NMR (400 MHz, $CDCl_3$) and ^{13}C NMR (101 MHz, $CDCl_3$) spectra of **13p**

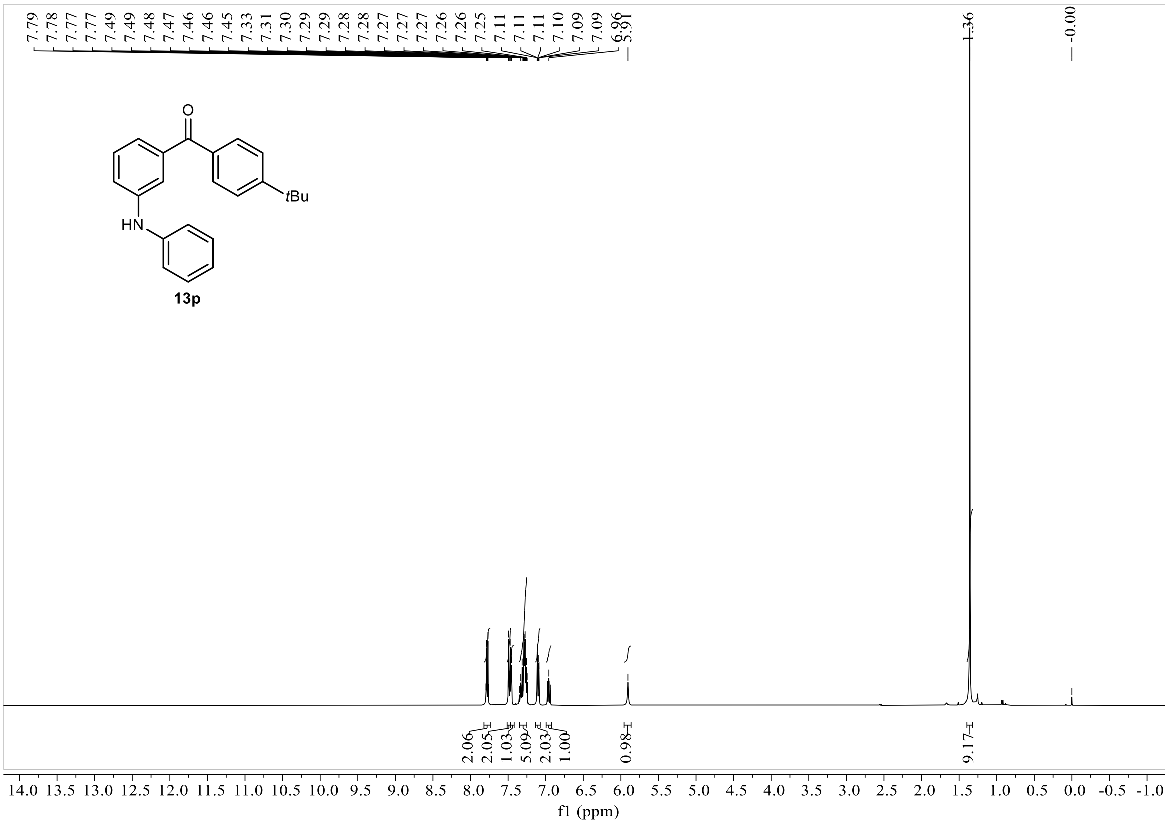

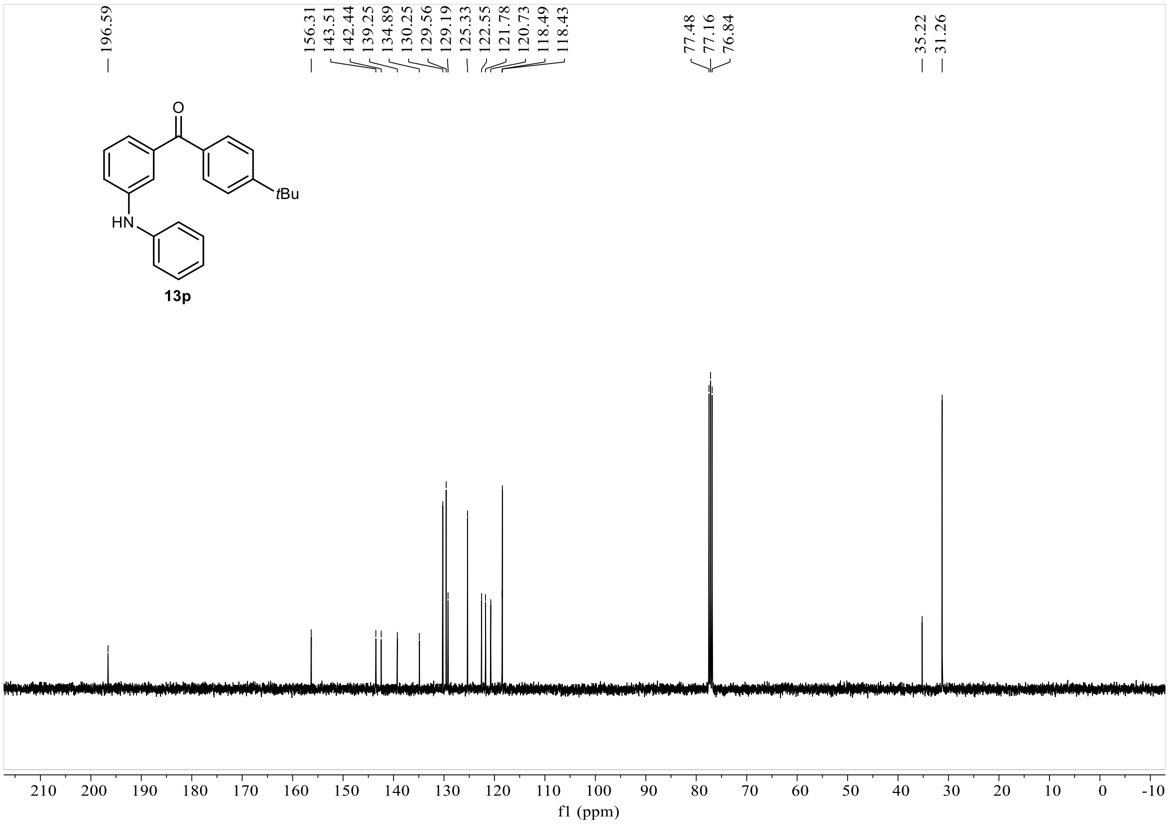

^{1}H NMR (400 MHz, $CDCl_3$) and ^{13}C NMR (101 MHz, $CDCl_3$) spectra of **13r**

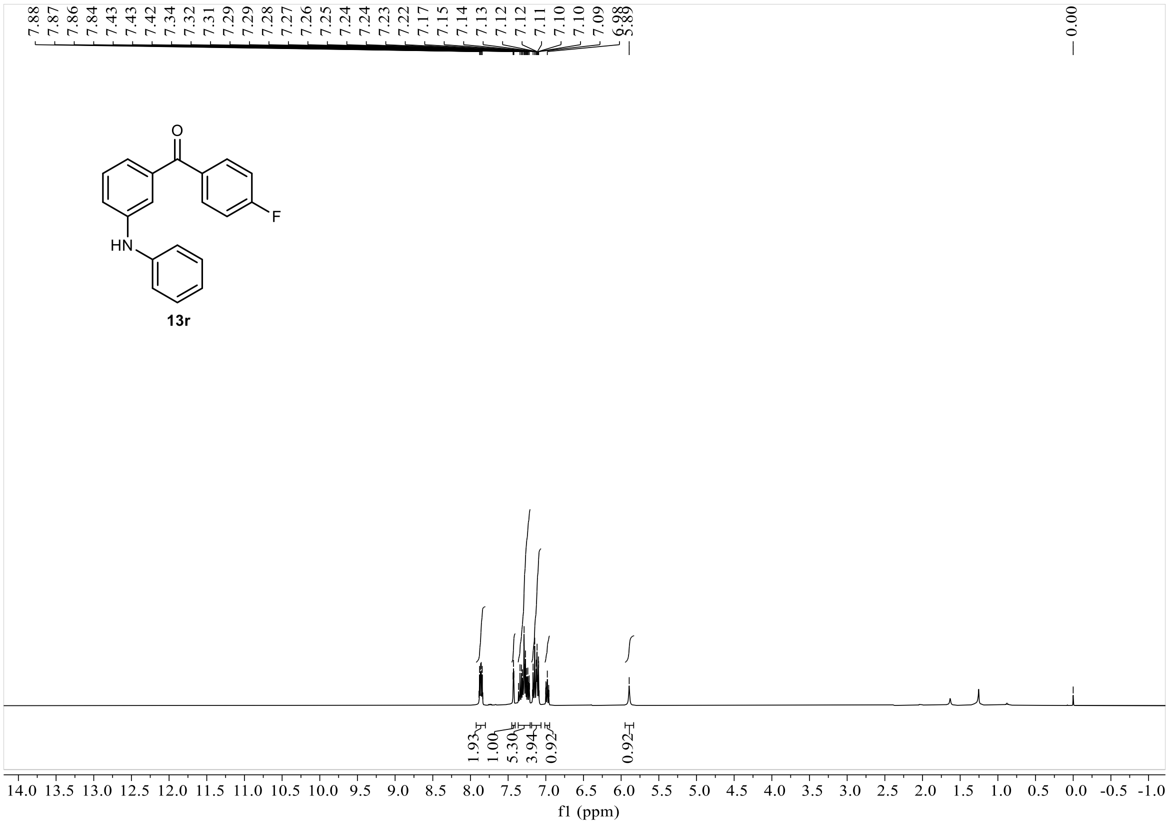

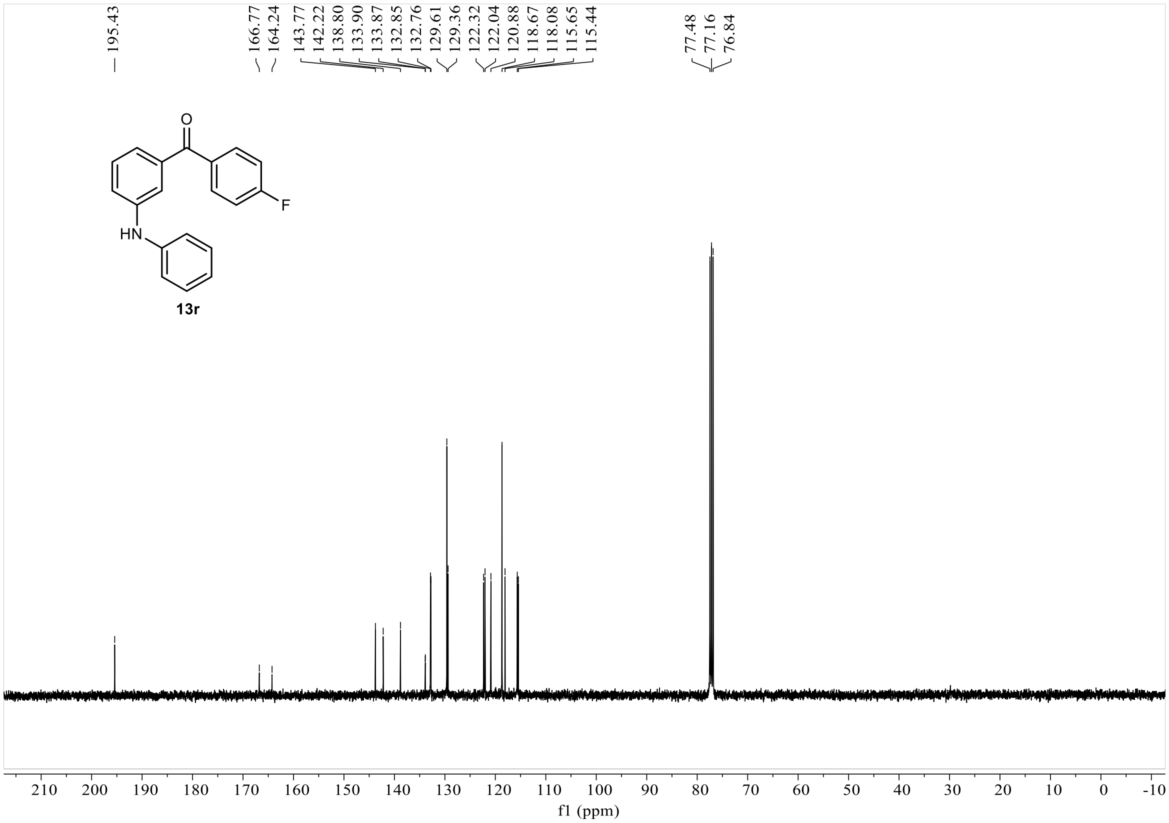

^{19}F NMR (376 MHz, $CDCl_3$) spectrum of **13r**

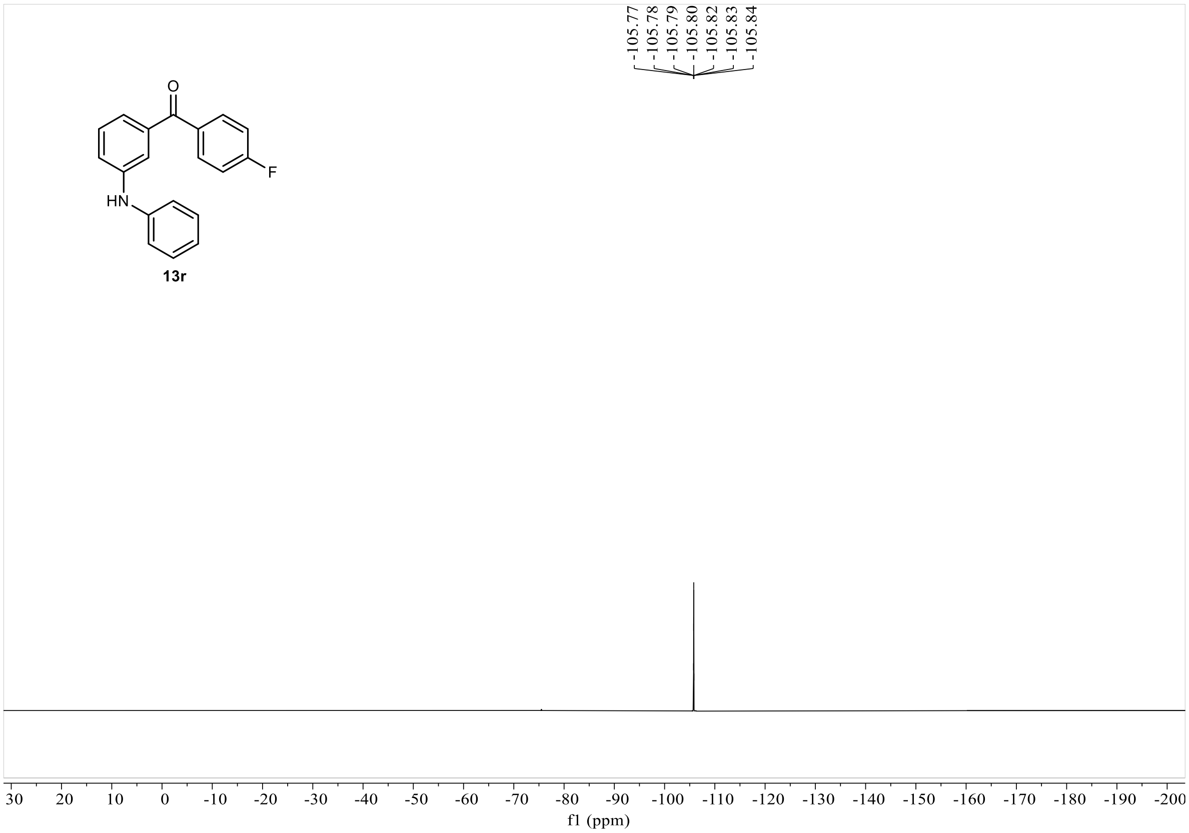

^{1}H NMR (400 MHz, $CDCl_3$) and ^{13}C NMR (101 MHz, $CDCl_3$) spectra of **13w**

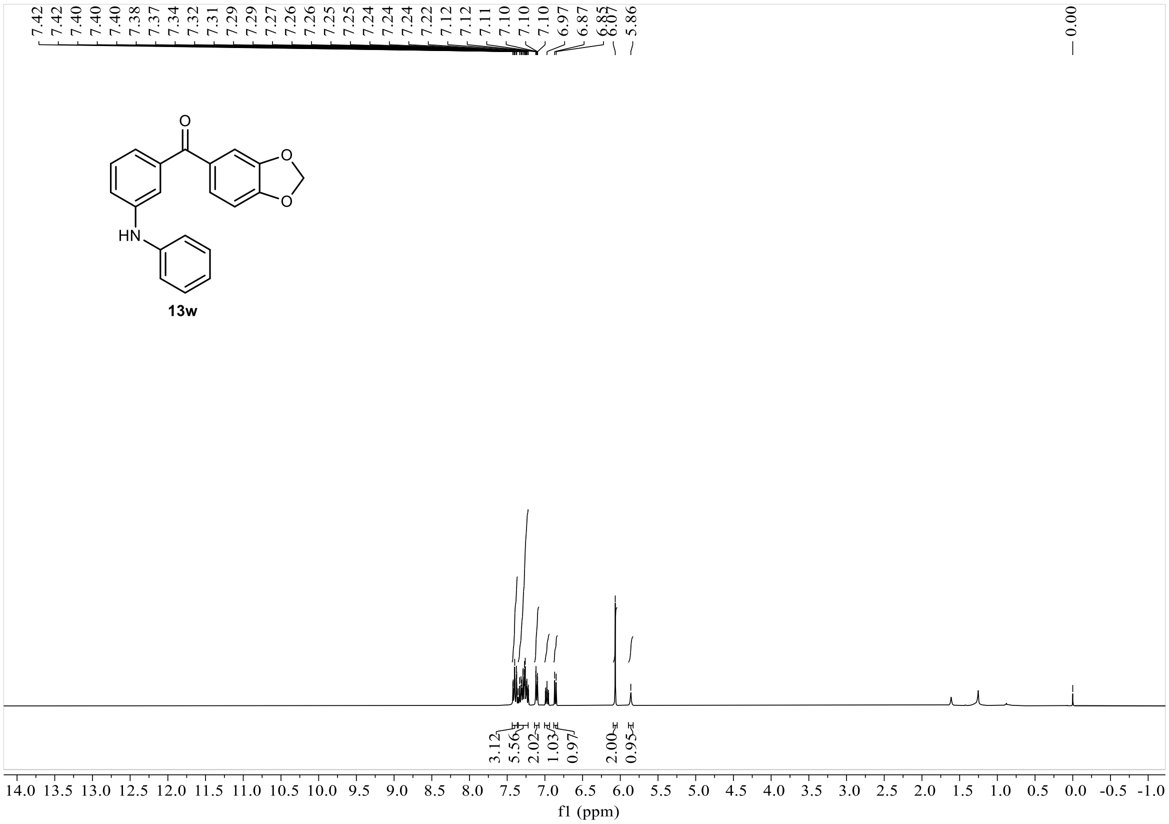

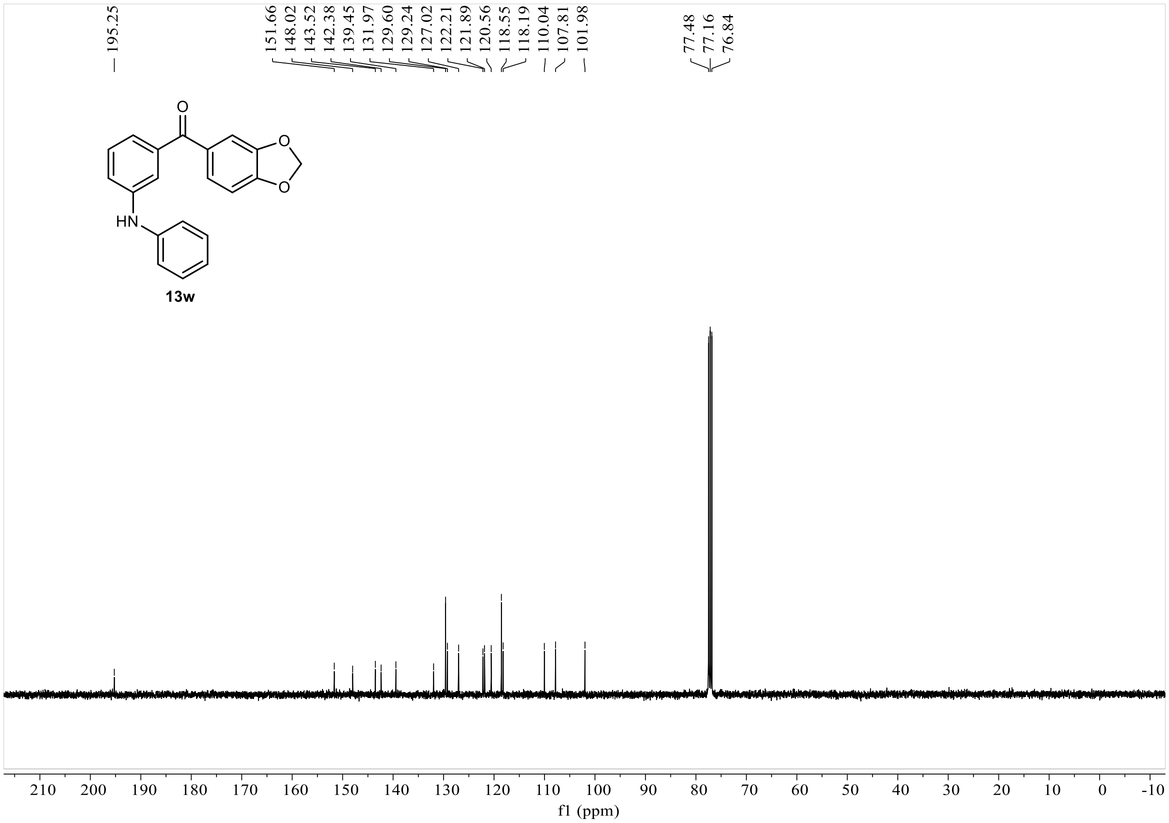

^{1}H NMR (400 MHz, $CDCl_3$) and ^{13}C NMR (101 MHz, $CDCl_3$) spectra of **13z**

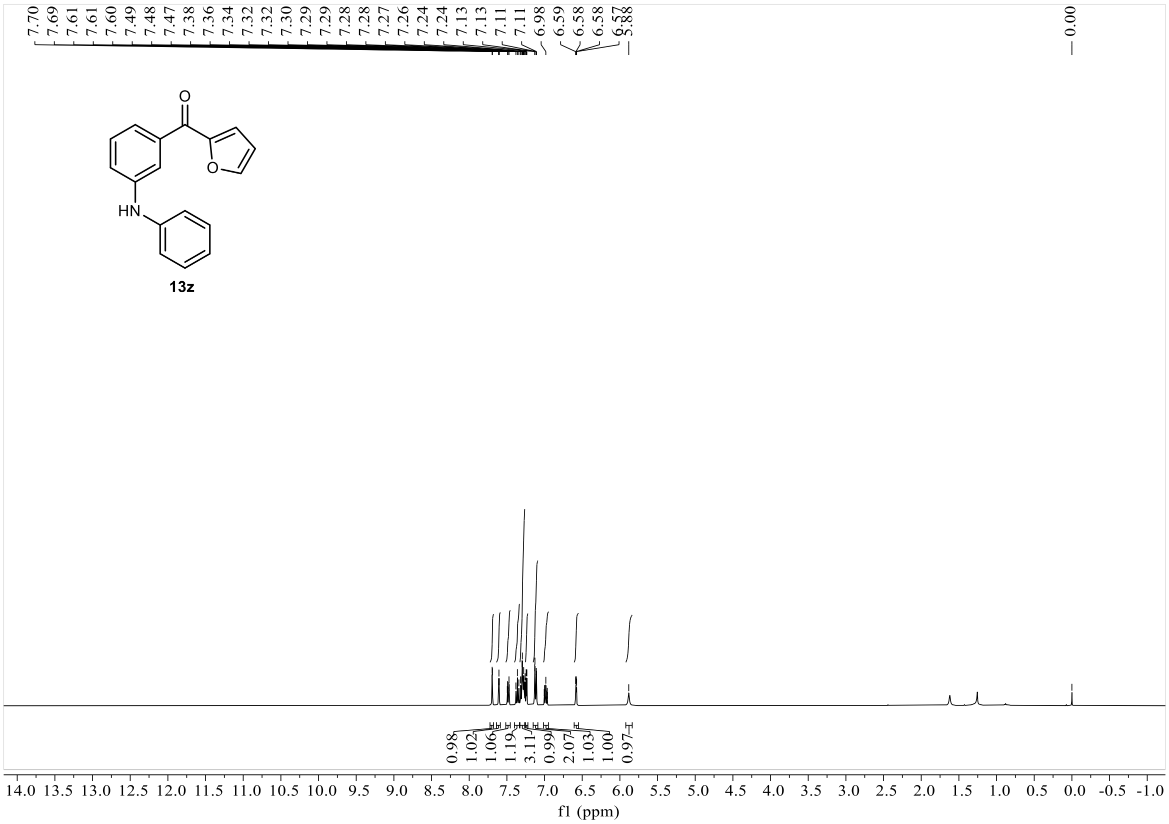

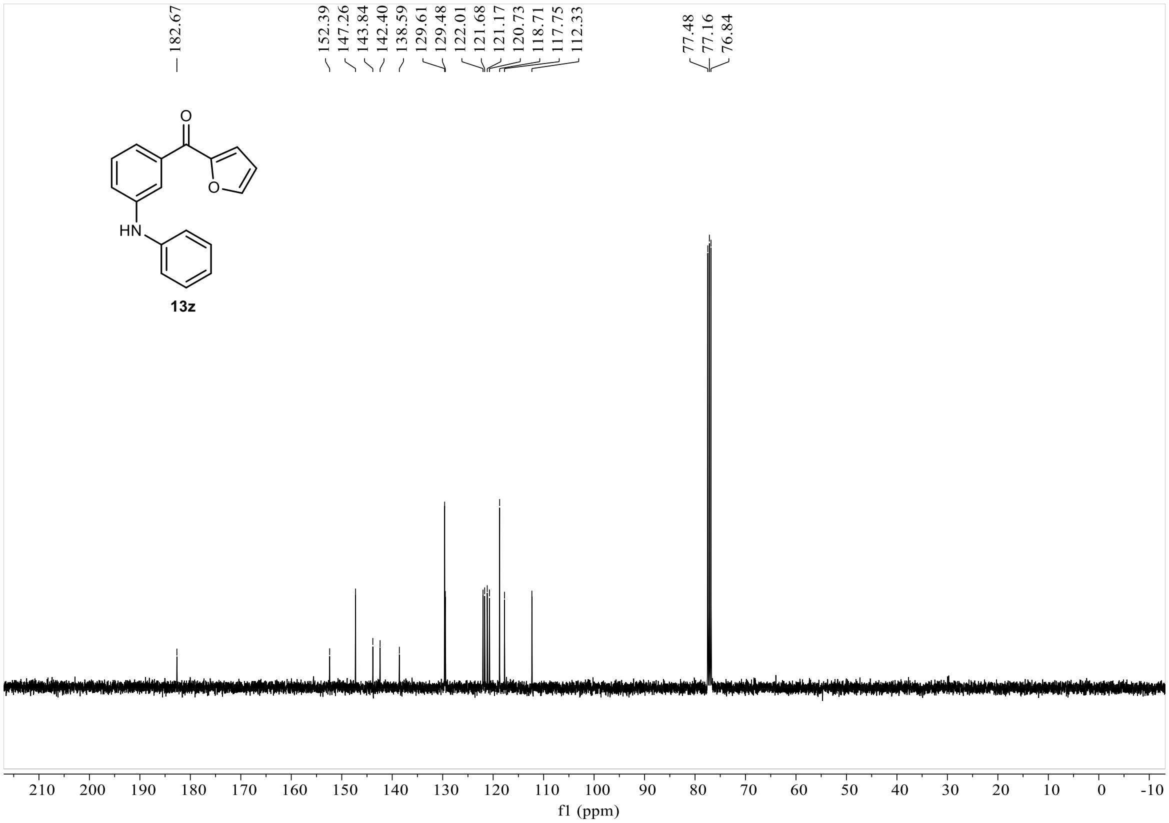

^{1}H NMR (400 MHz, $CDCl_3$) and ^{13}C NMR (101 MHz, $CDCl_3$) spectra of **13aa**

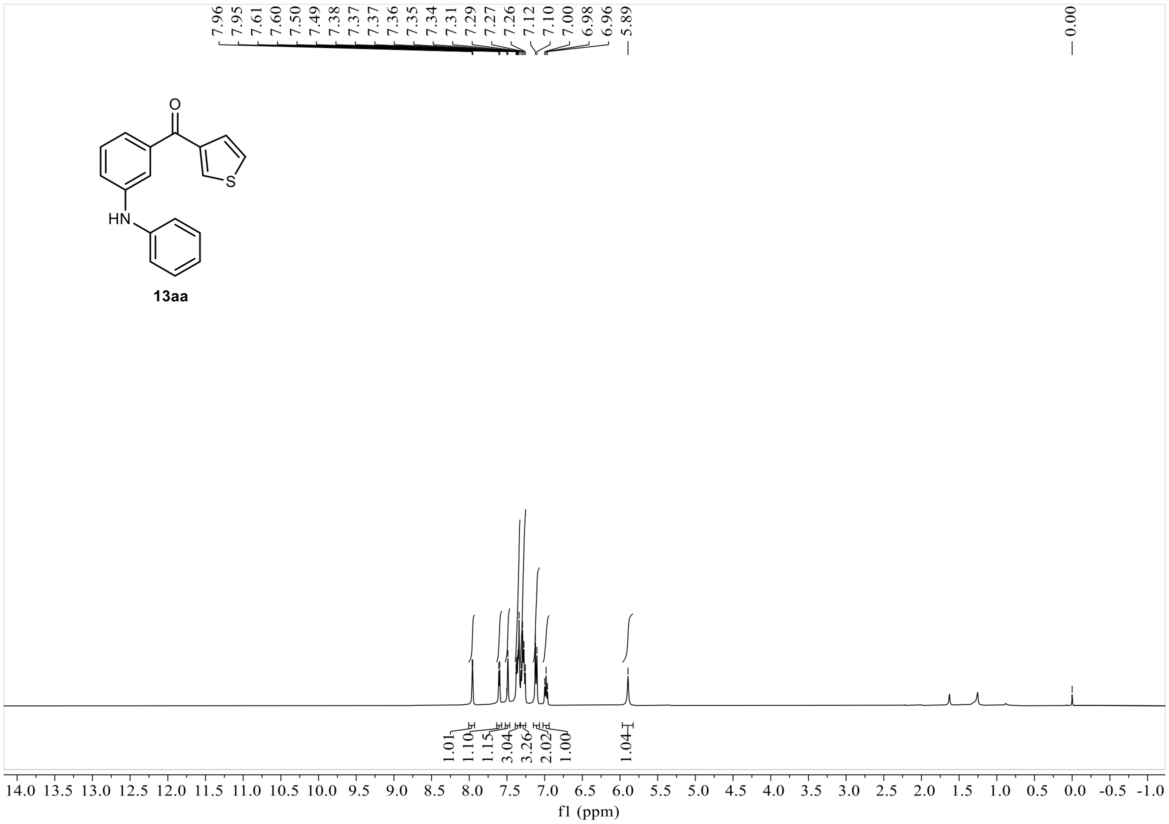

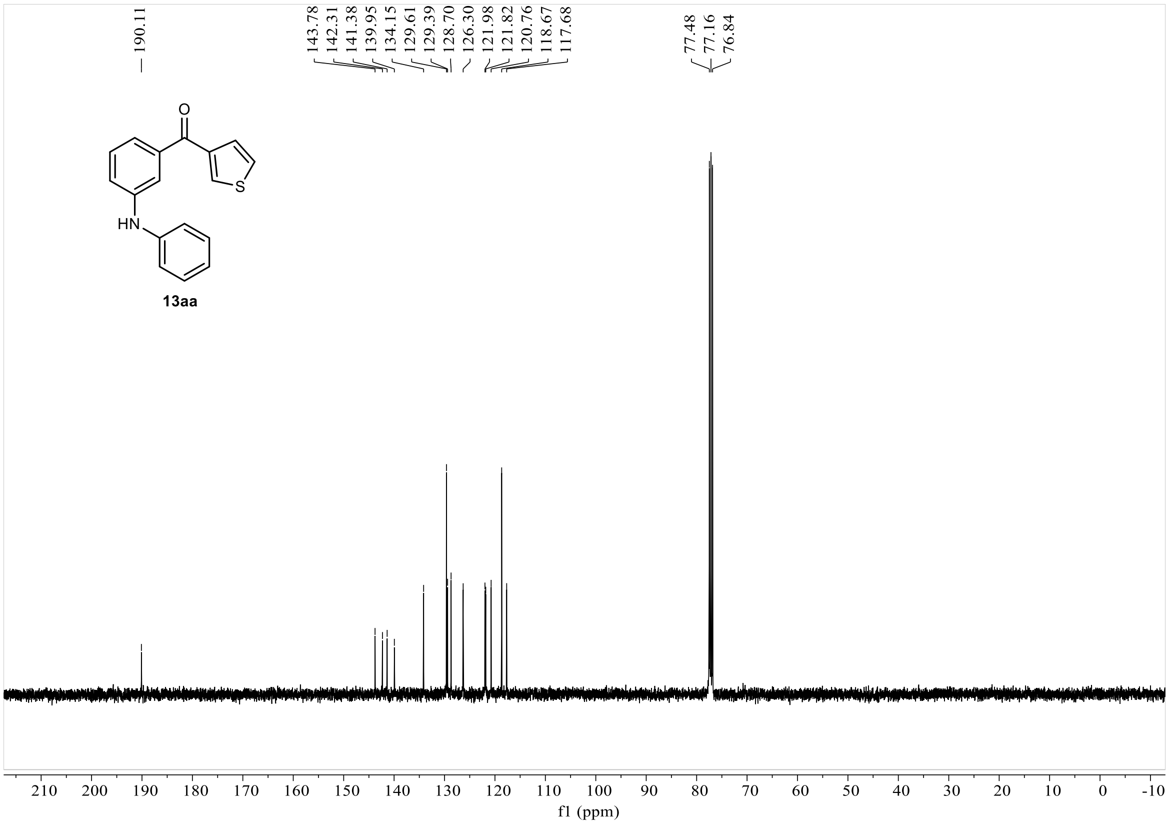

^{1}H NMR (400 MHz, $CDCl_3$) and ^{13}C NMR (101 MHz, $CDCl_3$) spectra of **3-benzylphenol**

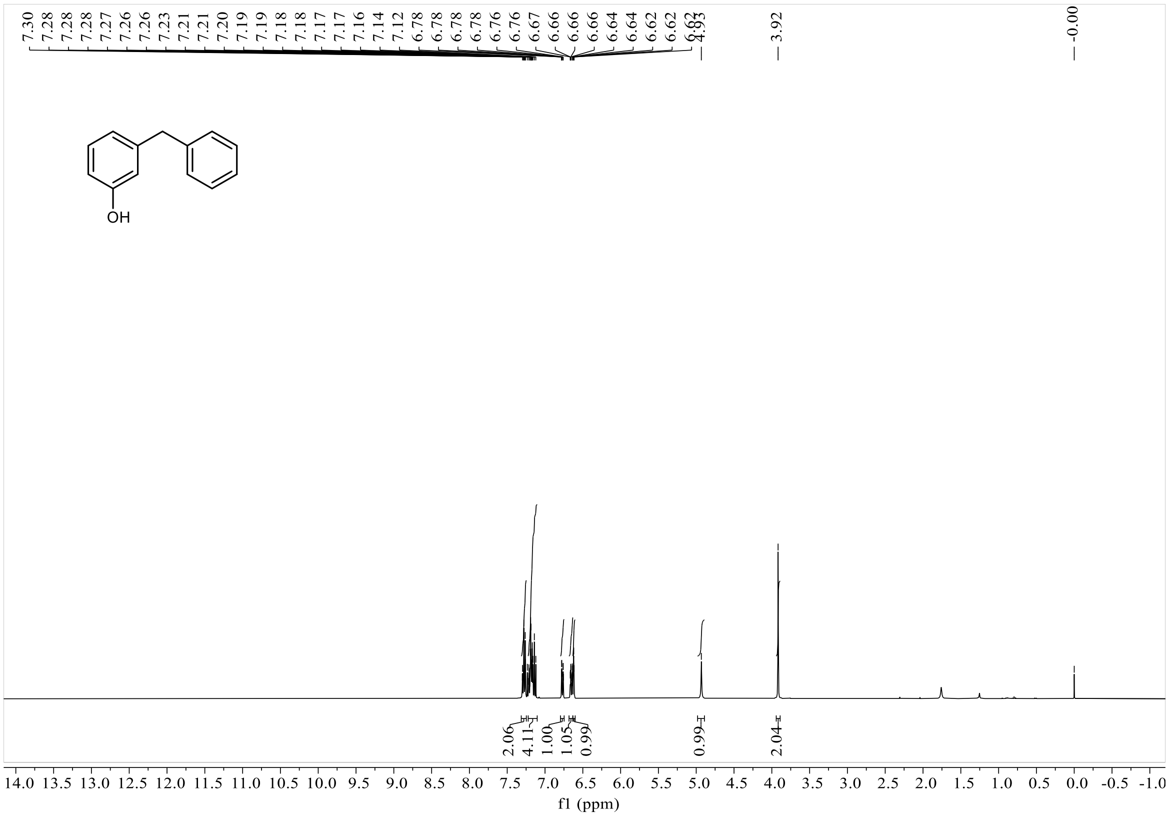

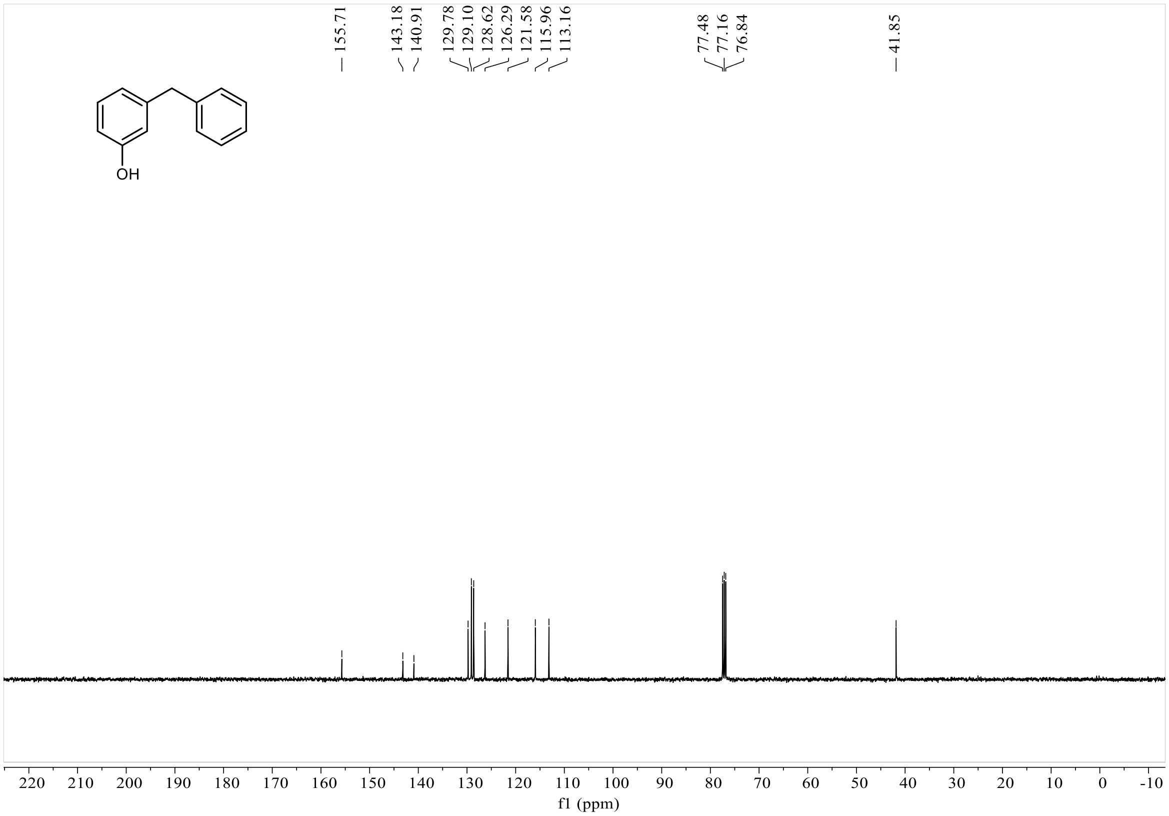

^{1}H NMR (400 MHz, DMSO-d_6) and ^{13}C NMR (101 MHz, DMSO-d_6) spectra of **3-(hydroxymethyl)phenol**

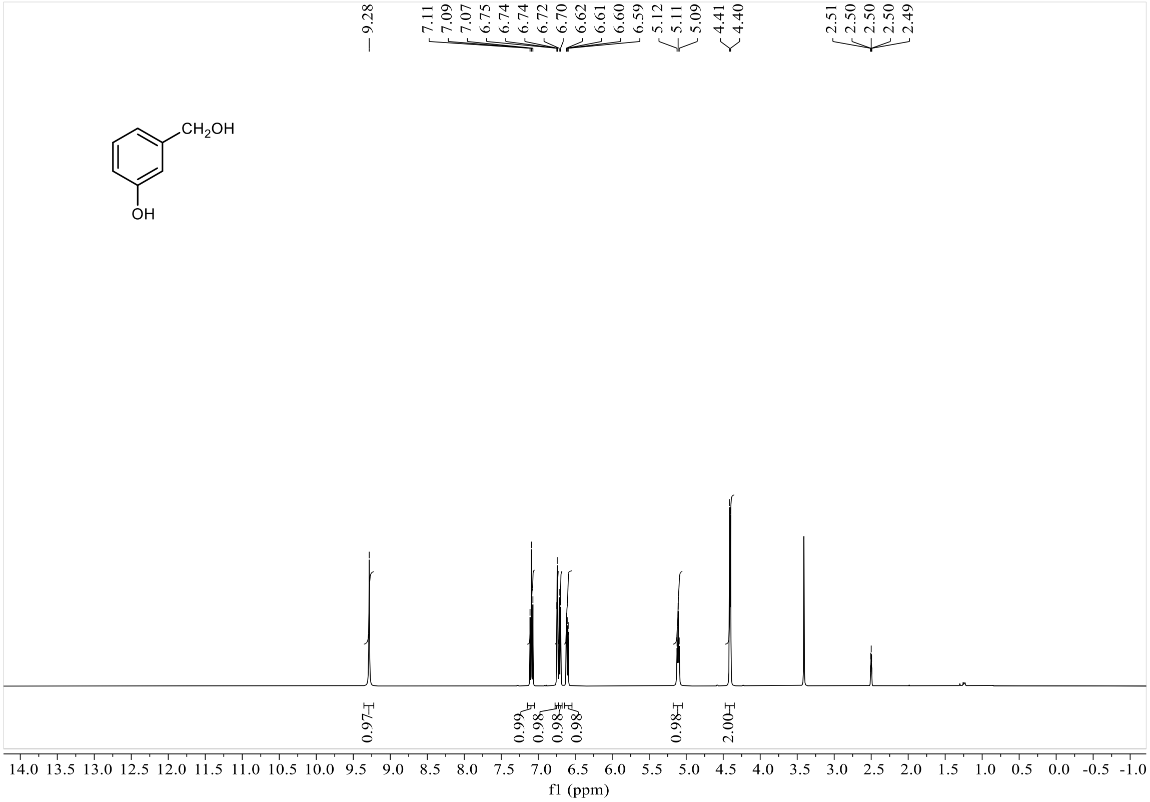

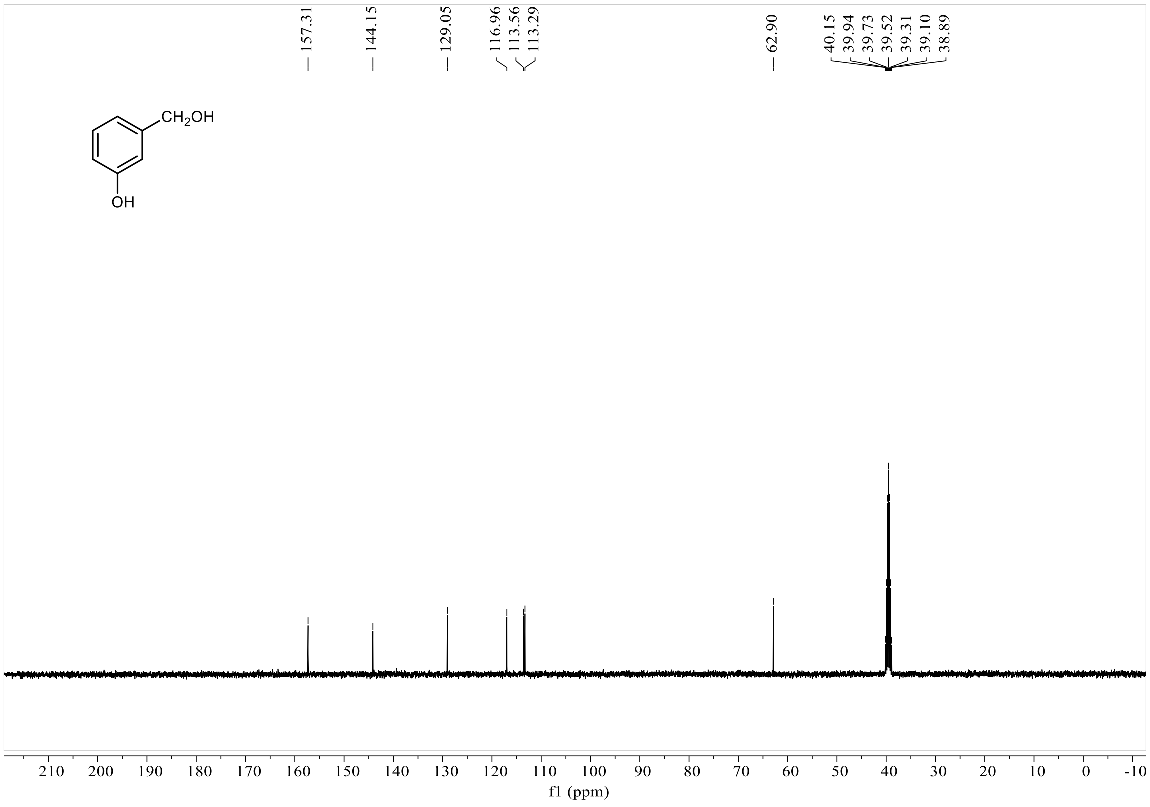

^{1}H NMR (400 MHz, DMSO-d_6) and ^{13}C NMR (101 MHz, DMSO-d_6) spectra of **3-hydroxybenzoic acid**

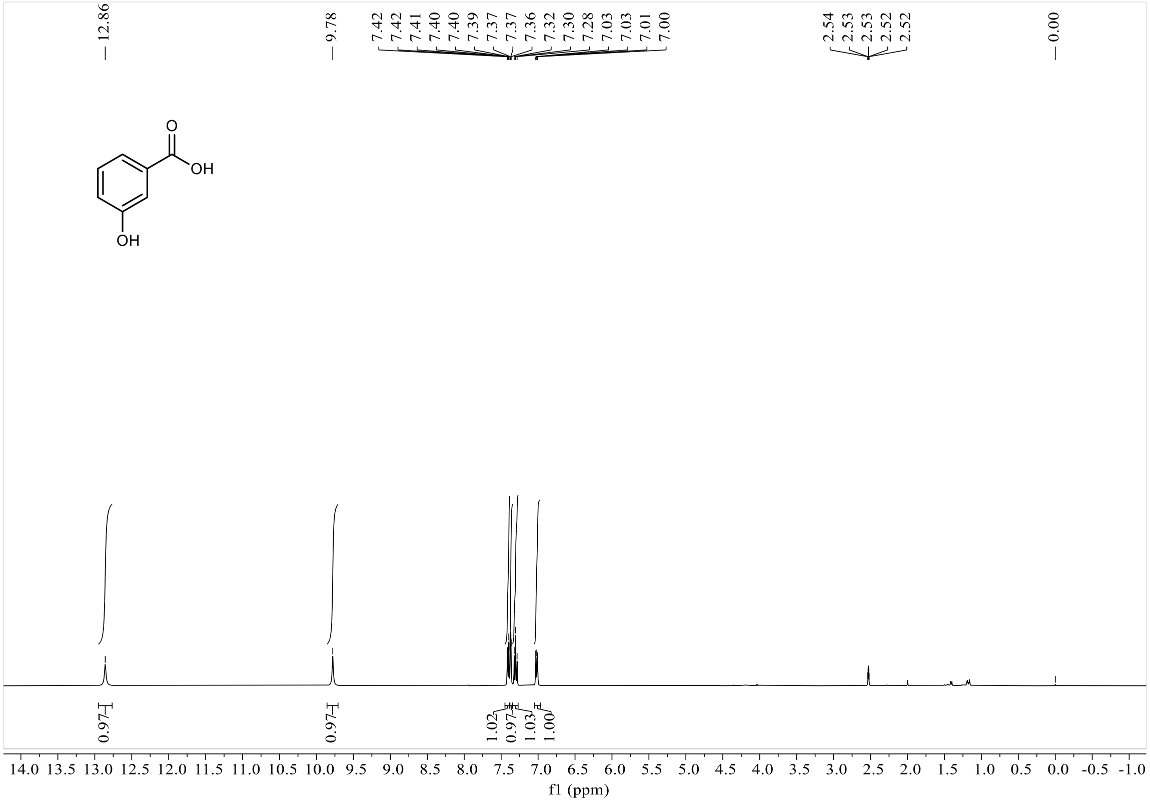

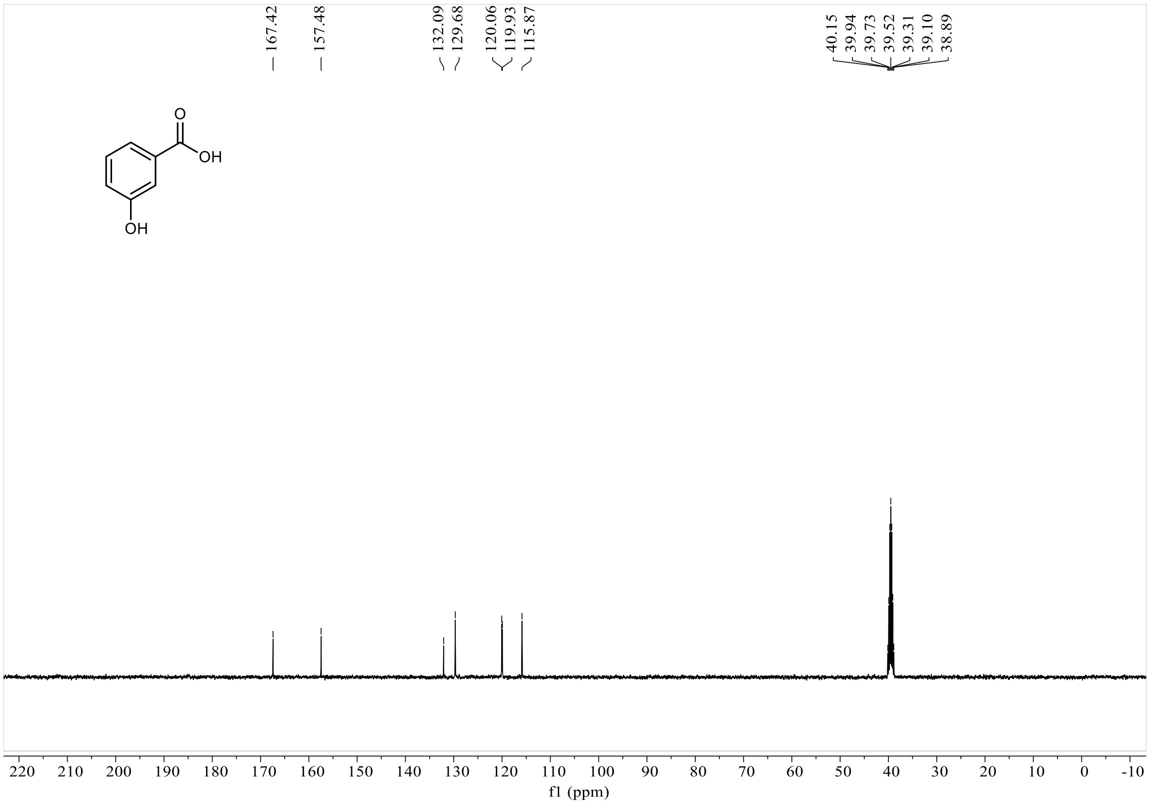

^{1}H NMR (400 MHz, DMSO-d_6) and ^{13}C NMR (101 MHz, DMSO-d_6) spectra of
(*E*)-3-(3-hydroxyphenyl)-1-phenylprop-2-en-1-one

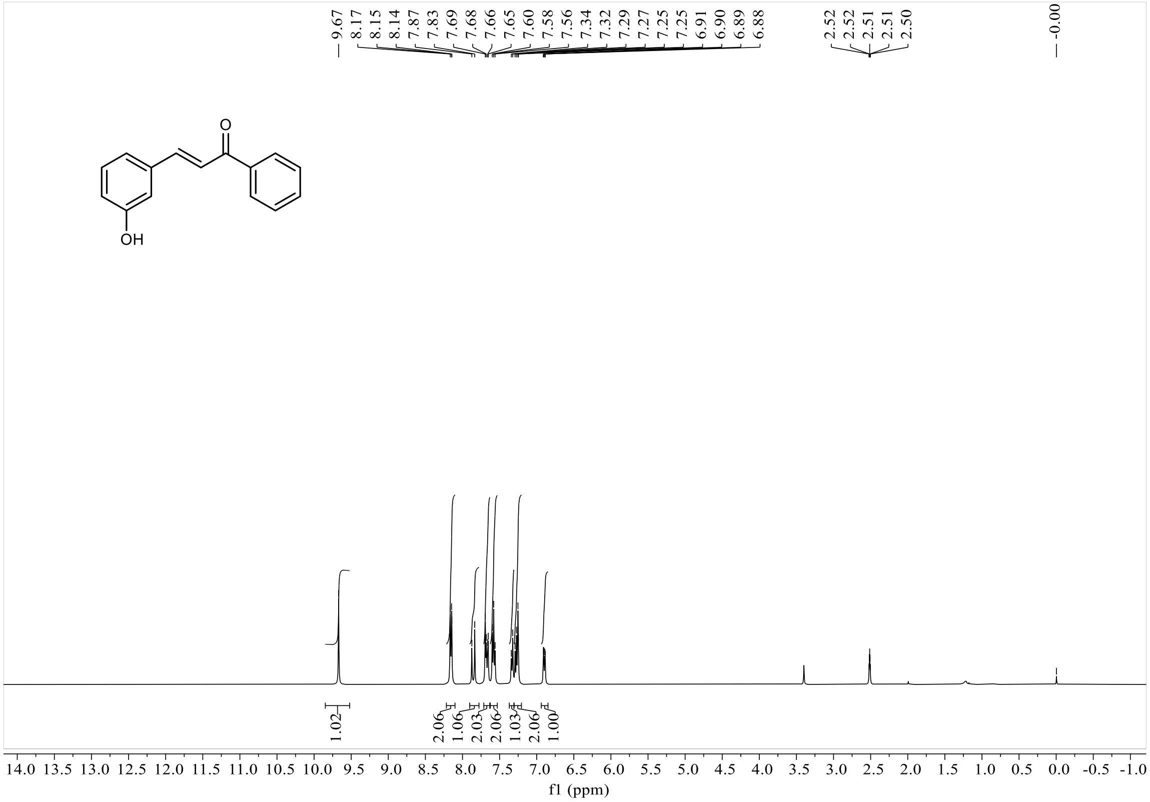

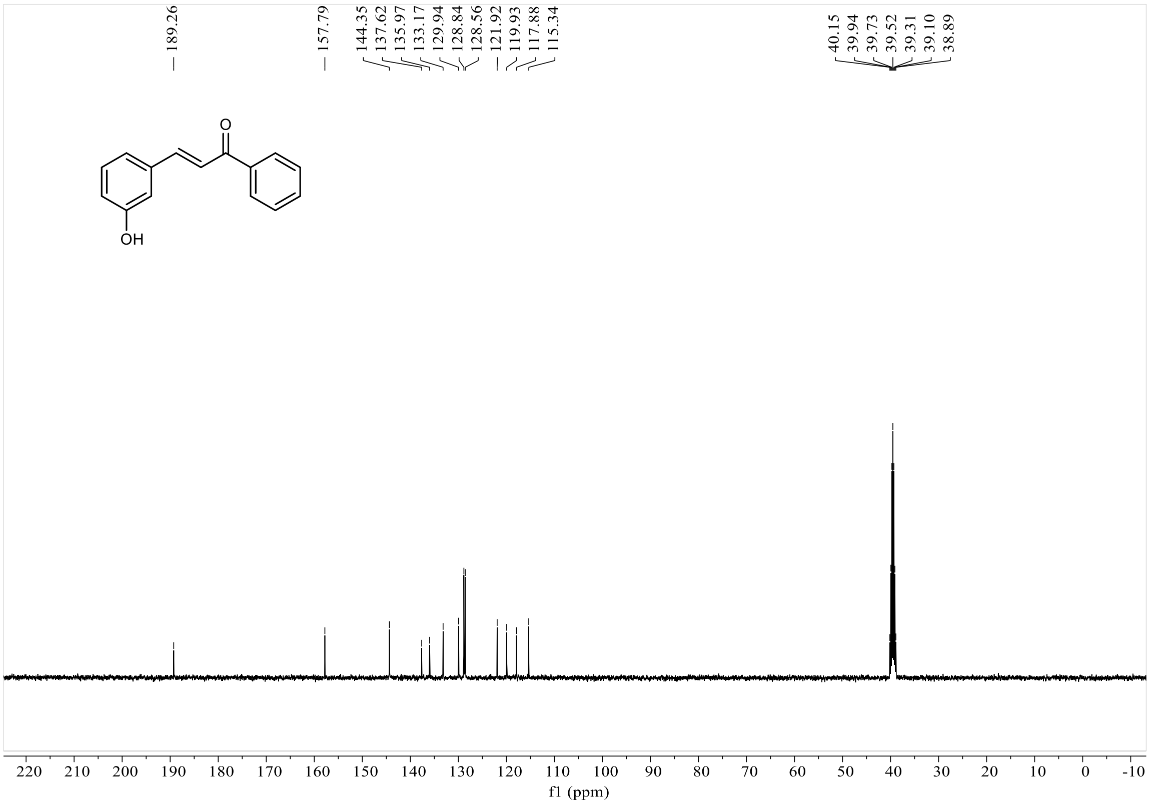

^{1}H NMR (400 MHz, $CDCl_3$) and ^{13}C NMR (101 MHz, $CDCl_3$) spectra of
(3-aminophenyl)(phenyl)methanone

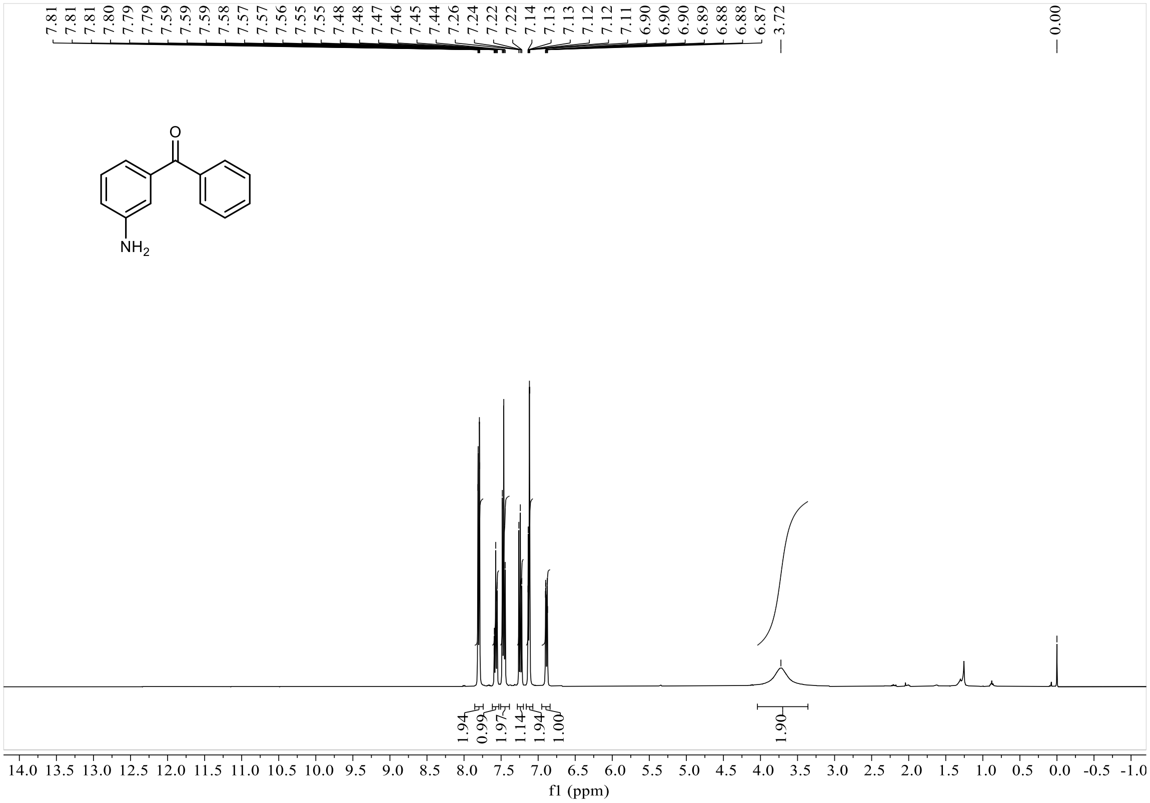

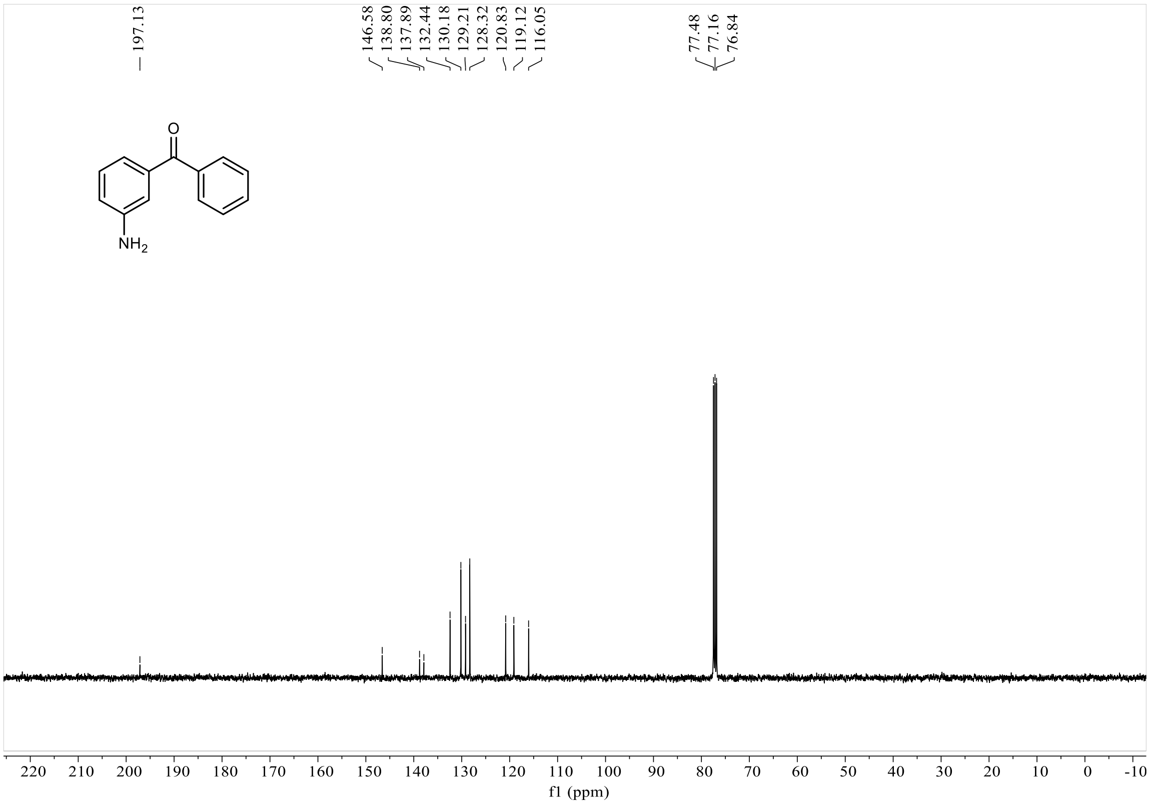

8. References

1. Zhao, B.-Y. et al. Aerobic oxidative dehydrogenation of ketones to 1,4-enediones. *Org. Lett.* **23**, 1216−1221 (2021).
2. Gutiérrez-Bonet, Á., Flores-Gaspar, A. & Martin, R. Fe-catalyzed regiodivergent [1,2]-shift of *α*-aryl aldehydes. *J. Am. Chem. Soc.* **135**, 12576–12579 (2013).
3. Li, X. & Zou, G. Palladium-catalyzed acylative cross-coupling of amides with diarylborinic acids and sodium tetraarylborates. *J. Organomet. Chem.* **794**, 136–145 (2015).
4. Wu, Q. et al. Pd-catalysed direct C(sp^2)–H fluorination of aromatic ketones: concise access to anacetrapib. *Chem. Commun.* **57**, 4544–4547 (2021).
5. Wang, L. et al. Direct C–H arylation of aldehydes by merging photocatalyzed hydrogen atom transfer with palladium catalysis. *ACS Catal.* **10**, 7543–7551 (2020).
6. Wang, C.-A. et al. Palladium-NHC (NHC = N-heterocyclic carbene)-catalyzed Suzuki−Miyaura cross-coupling of alkyl amides. *ACS Catal.* **12**, 2426–2433 (2022).
7. Amani, J. & Molander, G. A. Synergistic photoredox/nickel coupling of acyl chlorides with secondary alkyltrifluoroborates: dialkyl ketone synthesis. *J. Org. Chem.* **82**, 1856–1863 (2017).
8. Lee, G. S., Won, J., Choi, S., Baik, M.-H. & Hong, S. H. Synergistic activation of amides and hydrocarbons for direct C(sp^3)−H acylation enabled by metallaphotoredox catalysis. *Angew. Chem. Int. Ed.* **59**, 16933–16942 (2020).
9. Jana, A. et al. A phosphine-free manganese catalyst enables stereoselective synthesis of (1 + *n*)-membered cycloalkanes from methyl ketones and 1,*n*-Diols. *ACS Catal.* **10**, 2615–2626 (2020).
10. Ardkhean, R., Mortimore, M., Paton, R. S. & Fletcher, S. P. Formation of quaternary centres by copper catalysed asymmetric conjugate addition to *β*-substituted cyclopentenones with the aid of a quantitative structure–selectivity relationship. *Chem. Sci.* **9**, 2628–2632 (2018).
11. Mazzarella, D., Pulcinella, A., Bovy, L., Broersma, R. & Noël, T. Rapid and direct photocatalytic C(sp^3)−H acylation and arylation in flow. *Angew. Chem. Int. Ed.* **60**, 21277–21282 (2021).
12. Liu, M. T., Na, M., Li, Y., Biscoe, M. R. & Ryan, K. Conformational sensing by a mammalian olfactory receptor. *Chem. Eur. J.* **26**, 11462–11469 (2020).
13. Michailidou, F. et al. Pac13 is a small, monomeric dehydratase that mediates the formation of the 3'-deoxy nucleoside of pacidamycins. *Angew. Chem. Int. Ed.* **56**, 12492–12497 (2017).
14. Zhang, X., Wang, Z., Fan, X. & Wang, J. Synthesis of ketones through microwave irradiation promoted metal-free alkylation of aldehydes by activation of C(sp^3)−H bond. *J. Org. Chem.* **80**, 10660–10667 (2015).
15. Zheng, Z. et al. Copper-catalyzed synthesis of 2-acylbenzo[*b*]thiophenes from 3-(2-iodophenyl)-1-arylpropan-1-ones and potassium sulfide under aerobic conditions. *Org. Biomol. Chem.* **16**, 8020–8024 (2018).
16. Liu, Q. et al. Decatungstate catalyzed photochemical acetylation of C(sp^3)−H bonds. *Org. Lett.* **24**, 7983–7987 (2022).
17. Huang, B., Li, Y., Yang, C. & Xia, W. Electrochemical 1,4-reduction of *α,β*-unsaturated ketones with methanol and ammonium chloride as hydrogen sources. *Chem. Commun.* **55**, 6731–6734 (2019).
18. Sumino, S., Ui, T., Hamada, Y., Fukuyama, T. & Ryu, I. Carbonylative Mizoroki−Heck reaction of alkyl iodides with arylalkenes using a Pd/photoirradiation system. *Org. Lett.* **17**, 4952–4955 (2015).

19. Abdel-Halim, M. et al. From celecoxib to a novel class of phosphodiesterase 5 inhibitors: trisubstituted pyrazolines as novel phosphodiesterase 5 inhibitors with extremely high potency and phosphodiesterase isozyme selectivity. *J. Med. Chem.* **64**, 4462–4477 (2021).
20. Chaturvedi, J., Haldar, C., Bisht, R., Pandey, G. & Chattopadhyay, B. Meta selective C−H borylation of sterically biased and unbiased substrates directed by electrostatic interaction. *J. Am. Chem. Soc.* **143**, 7604–7611 (2021).
21. Sun, X., Wang, M., Li, P., Zhang, X. & Wang, L. H_2O_2-mediated oxidative formation of amides from aromatic amines and 1,3-diketones as novel acylation agents via C−C bond cleavage at room temperature in water under metal-free conditions. *Green Chem.* **15**, 3289–3294 (2013).
22. Diao, T., Wadzinski, T. J. & Stahl, S. S. Direct aerobic *α,β*-dehydrogenation of aldehydes and ketones with a $Pd(TFA)_2$/4,5-diazafluorenone catalyst. *Chem. Sci.* **3**, 887–891 (2012).
23. Xu, L., Wang, C., Gao, Z. & Zhao, Y.-M. Total synthesis of (±)-cephanolides B and C via a palladium-catalyzed cascade cyclization and late-stage sp^3 C−H bond oxidation. *J. Am. Chem. Soc.* **140**, 5653−5658 (2018).
24. Whitaker, L., Harb, H. Y. & Pulis, A. P. One-pot borylation/Suzuki–Miyaura sp^2–sp^3 cross-coupling. *Chem. Commun.* **53**, 9364−9367 (2017).
25. Sun, Z. et al. Enantioselective total synthesis of (−)-cephalotanin B. *Angew. Chem. Int. Ed.* **62**, e202312599 (2023).
26. Murai, N., Yonaga, M. & Tanaka, K. Palladium-catalyzed direct hydroxymethylation of aryl halides and triflates with potassium acetoxymethyltrifluoroborate. *Org. Lett.* **14**, 1278−1281 (2012).
27. Sang, D., Yue, H., Fu, Y. & Tian, J. Cleavage of carboxylic esters by aluminum and iodine. *J. Org. Chem.* **86**, 4254−4261 (2021).
28. Plasch, K. et al. Regioselective enzymatic carboxylation of bioactive (poly)phenols. *Adv. Synth. Catal.* **359**, 959–965 (2017).
29. Yue, J.-P. et al. Metallaphotoredox-enabled aminocarboxylation of alkenes with CO_2. *Nat. Catal.* **6**, 959–968 (2023).
30. Thakur, K. G., Ganapathy, D. & Sekar, G. D-Glucosamine as a green ligand for copper catalyzed synthesis of primary aryl amines from aryl halides and ammonia. *Chem. Commun.* **47**, 5076–5078 (2011).
